# (4+3) Annulation of Donor‐Acceptor Cyclopropanes and Azadienes: Highly Stereoselective Synthesis of Azepanones

**DOI:** 10.1002/anie.202209006

**Published:** 2022-07-28

**Authors:** Stefano Nicolai, Jérôme Waser

**Affiliations:** ^1^ Laboratory of Catalysis and Organic Synthesis Institute of Chemical Sciences and Engineering Ecole Polytechnique Fédérale de Lausanne 1015 Lausanne Switzerland

**Keywords:** Azadienes, Azepanones, Cycloadditions, Cyclopropanes, Tox-Ligands

## Abstract

Azepanes are important seven‐membered heterocycles, which are present in numerous natural and synthetic compounds. However, the development of convergent synthetic methods to access them remains challenging. Herein, we report the Lewis acid catalyzed (4+3) annulative addition of aryl and amino donor‐acceptor cyclopropanes with 2‐aza‐1,3‐dienes. Densely substituted azepane derivatives were obtained in good to excellent yields and with high diastereoselectivity. The reaction occurred under mild conditions with ytterbium triflate as the catalyst. The use of copper triflate with a trisoxazoline (Tox) ligand led to an enantioselective transformation. The obtained cycloadducts were convenient substrates for a series of further modifications, showing the synthetic utility of these compounds.

## Graphical abstract

Azepanones are important and widespread seven‐membered heterocycles, but their synthesis is challenging. A convergent method to access these scaffolds was developed relying on a (4+3) annulation reaction of azadienes and donor‐acceptor cyclopropanes. Good to excellent yields and high diastereoselectivity were achieved using Yb(OTf)_3_ as the catalyst. Asymmetric induction was possible with Cu(OTf)_2_ in the presence of a trisoxazoline (Tox) ligand.

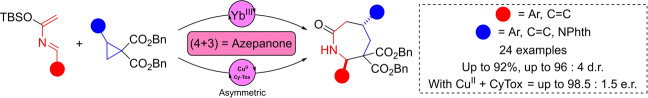

Medium‐sized (hetero)cycles are widespread motifs in natural and synthetic bioactive substances.^[1]^ In particular, seven membered azacycles (azepanes) are well known therapeutic agents.^[2]^ When compared to five‐ and six‐membered rings, the more challenging synthesis of seven‐membered rings has however led to a scarcity of methods for accessing them.^[3]^ One of the most attractive strategy towards medium‐sized rings relies on convergent intermolecular annulations.^[4]^ Although broadly exploited for the synthesis of seven‐membered carbocycles,^[5]^ extending this approach to the construction of azepanes is more difficult and has been poorly explored.^[6]^

As readily available equivalents of three‐carbon zwitterionic synthons, Donor‐Acceptor Cyclopropanes (DACs) have been widely used to generate five‐ and six‐membered (hetero)cycles by (3+2) and (3+3) annulations.[^7^, ^8^] Applying these compounds in (4+3) annulative reactions provides a powerful tool for the assemblage of seven‐membered rings.^[9]^ However, only few of such methods have been developed so far.^[10]^ Recently, the synthesis of benzoazepines has been accomplished using DACs in (4+3) annulations under Lewis acid or palladium catalysis with 2‐amino benzaldehydes^[10h]^ and anthranils[^10d^, ^10e^] as 1,4‐dipolarophiles (Scheme 1A). Despite these advances, annulations giving access to saturated azepane scaffolds have been elusive so far.^[11]^

**Scheme 1:**
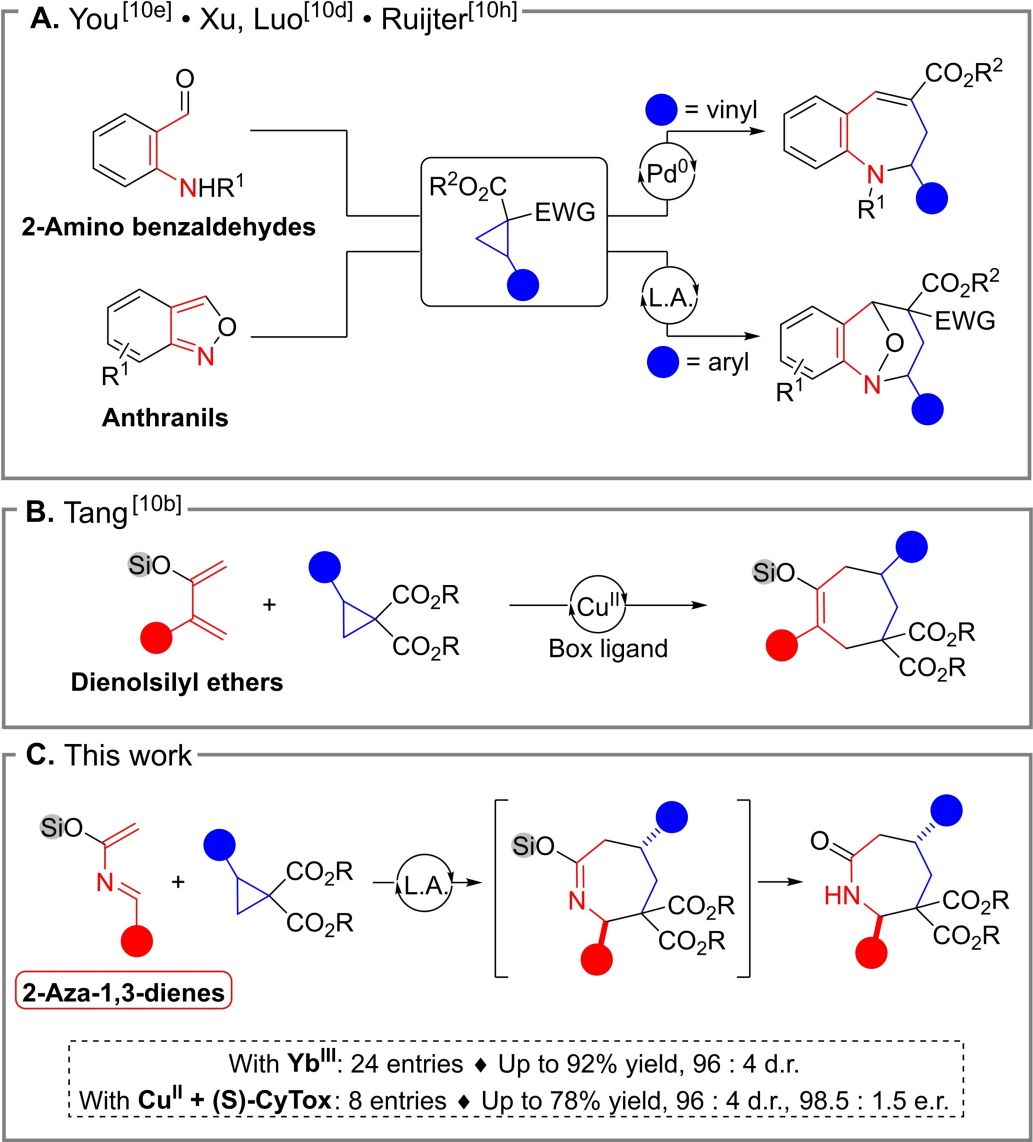
(4+3) Annulations for the synthesis of: A) benzoazepines; B) Seven‐membered carbocycles; C) Saturated azepanes scaffolds (This work).

Recently, Tang and co‐workers described the synthesis of seven‐membered carbocycles through a Lewis acid‐catalyzed (4+3) process involving DACs and dienolsilyl ethers (Scheme 1B).^[10b]^ Ghosez and co‐workers introduced in the 1980s highly reactive azadienes incorporating both an imine and a silyl enol ether moieties, and used them in hetero‐Diels Alder reactions.^[12]^ We reasoned that azadienes could be competent aza‐1,4‐dipolarophiles to react with DACs.^[13]^ Desilylation and tautomerization of the labile silyl imidate intermediates would lead to seven‐membered lactams (Scheme 1C). Herein, we describe the first, highly diastereoselective (4+3) annulation of aryl and amino DA cyclopropanes with azadienes, and our preliminary results in the development of the corresponding enantioselective variant.

To start our investigation, we focused on more stable and easily accessible alkoxy azadiene **1** (Scheme 2).^[12f]^ Azadiene **1** reacted with dibenzyl cyclopropane dicarboxylate **2 a** to provide ϵ‐lactam **3** in up to 72 % yield. No product was formed with diesters **2 a′** and **2 a′′**. Best results were provided by Yb(OTf)_3_ as the catalyst. Other Lewis acids were not or less effective (see Supporting Information). The reaction took place in DCM at room temperature. However, both yield and d.r. were poorly reproducible. This may be due to the low stability of the N,O acetal function in **3**, likely prone to undergo hydrolysis and isomerization under acidic conditions.

**Scheme 2:**
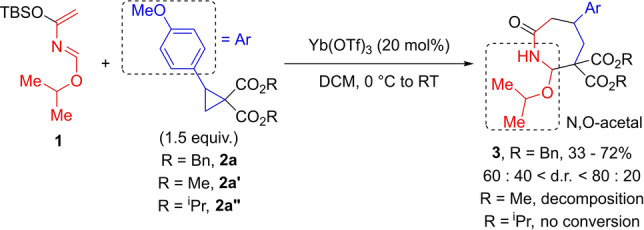
Preliminary investigation of the (4+3) annulation using azadiene **1**.

To avoid the issue of the sensitive N,O acetal function, phenyl substituted azadiene **4 a** was examined. When **1 a** was replaced by **4 a**, cyclopropane **2 a** was converted into azepanone **5 a.a** with excellent diastereoselectivity, and in a reproducible 80 % yield (Table 1, entry 1). Dibenzyl diester **2 a** was confirmed as the best DA cyclopropane, whereas other esters underwent decomposition or led to lower yields (entries 2–4). Other catalysts were not or less effective (entries 5–7). Moreover, the choice of the Lewis acid strongly affected the diastereoselectivity of the process. Interestingly, when Cu(OTf)_2_ was used with racemic Box ligand **L1** (see below), **5 a.a** was delivered with high yield but lower d.r. than in the absence of the ligand (entry 8). A similar Cu^II^‐Box had been used by Tang and co‐workers in their (4+3) annulation with dienolsilyl ethers.^[10b]^ The addition of molecular sieves (3Å MS) was beneficial to the reaction: the annulation with Yb(OTf)_3_ (20 mol %) and 1.5 equivalents of **4 a** gave **5 a.a** in 90 % yield and 94 : 6 d.r. (entry 9). X‐Ray diffraction of a single crystal obtained from the major diastereoisomer permitted at this point to assign the relative configuration of the latter as *trans* (Scheme 3A).[^14^, ^15^] A lower 10 mol % catalytic loading led to a diminished yield even in combination with a larger amount of azadiene **4 a** (entry 10). High yields and d.r. were obtained when the reaction was performed starting with 1 mmol or even 2.4 mmol (1.0 g) of cyclopropane **2 a** (entries 11 and 12).

**Table 1:** Optimization of the (4+3) annulation with azadiene **2 a**.

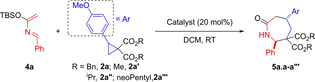				
Entry	R group	Catalyst	Yield^[a]^	d.r.
1	Bn	Yb(OTf)_3_	80 %	95 : 5
2	Me	Yb(OTf)_3_	decomp.	–
3	^i^Pr	Yb(OTf)_3_	40 %	>95 : 5
4	neoPentyl	Yb(OTf)_3_	35 %	>95 : 5
5	Bn	Dy(OTf)_3_	53 %	95 : 5
6	Bn	MgI_2_	57 %	63 : 37
7	Bn	Cu(OTf)_2_	71 %	89 : 11
8^[b]^	Bn	Cu(OTf)_2_+**L1**	83 %	70 : 30
9^[b]^	Bn	Yb(OTf)_3_	90 %	94 : 6
10^[b,c]^	Bn	Yb(OTf)_3_	77 %	>95 : 5
11^[b,d]^	Bn	Yb(OTf)_3_	89 %^[e]^	96.5 : 3.5
12^[b,f]^	Bn	Yb(OTf)_3_	90 %	≥95 : 5

Reaction conditions: 1.0 equiv cyclopropane **2 a**–**a′′′**, 1.5 equiv azadiene **4 a**, 20 mol % catalyst, 0.10–0.14 M in DCM, at RT, overnight. [a] Isolated yield upon column chromatography. [b] With 60–70 mg 3 Å MS per 0.1 mmol **2 a**. [c] Using 2.0 equiv **4 a**, 10 mol % catalyst. [d] Starting from 1.0 mmol **2 a**. [e] Average on two reiterations. [f] Starting from 1.0 g (2.4 mmol) **2 a**.

**Scheme 3:**
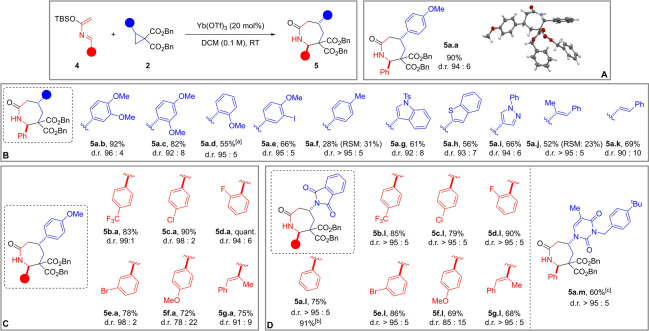
Scope of the reaction. A) Product **5 a.a**, obtained from model substrate **2 a** and azadiene **4 a**; X‐Ray diffraction of **5 a.a**. B) Products obtained from diverse (hetero)aryl and alkenyl DACs **2**. C) Products obtained from diverse azadienes **4**. D) Products obtained from cyclopropanes containing a phthalimide (**4 l**) or a thymine (**4 m**) substituent. General conditions: 0.20 mmol (1.0 equiv) cyclopropane **2**, 0.30 mmol (1.5 equiv) azadiene **4**, 20 mol % Yb(OTf)_3_, 140–150 mg 3 Å MS, DCM (0.1 M), RT, overnight. [a] Performed on 0.10 mmol scale. [b] Average yield over two reiterations. [c] With 0.50 mmol (2.5 equiv) azadiene **4 a**.

With an optimized protocol in hands, the scope of the reaction was first investigated with diverse dibenzyl cyclopropane dicarboxylates **2** together with azadiene **4 a** (Scheme 3B).

Starting from dimethoxy phenyl cyclopropanes, cycloadducts **5 a.b**–**c** were formed in 92 % and 82 % yield and with very high selectivity. By contrast, less electron‐rich substrates worked less effectively (**5 a.d**–**f**). These results were not surprising because annulations of DACs are known to be sensitive to the electron density on the donor substituent of the cyclopropane.^[7b]^ Heteroaromatic groups on the three‐membered ring were well tolerated, and cycloadducts **5 a.g**–**i** were accessed in 56–66 % yields. The transformation was also effective with alkenyl cyclopropanes: products **5 a.j**–**k** were synthesized in over 50 % yield. To test the scope with respect to the diene component, cyclopropane **2 a** was submitted to our protocol with a variety of azadienes **4** (Scheme 3C). The transformation proceeded smoothly in the presence of a *p*‐trifluoromethyl or a halogen substituent on the phenyl ring delivering azepanones **5 b**–**e.a** in more than 78 % yields and with high diastereoselectivity. With an electron‐rich *p*‐anisyl substituent on the azadiene, a loss of efficiency was observed and the d.r. was lower (product **5 f.a**). A methyl styryl containing azadiene gave alkenyl azepanone **5 g.a** in 75 % yield and 91 : 9 d.r.

We then turned our attention to DA cyclopropanes containing an amido substituent.^[16]^ Our optimized procedure worked effectively also with this class of substrates (Scheme 3D). Starting from model azadiene **4 a**, phthalimido‐containing cycloadduct **5 a.l** was formed in 75 % yield and almost complete diastereoselectivity. A scale‐up to 1.0 mmol was possible with no diminution of d.r. and with yield increasing up to 91 %. Other azadienes worked equally well: the best results were obtained with trifluoromethylphenyl‐ and halophenyl azadienes (**5 b**–**e.l**). Finally, we could also accomplish the synthesis of azepanone **5 a.m** from the corresponding DAC bearing a protected thymine.^[16b]^

Controlling the absolute configuration of newly generated stereocenters is highly desirable when developing a new synthetic method. Numerous examples of enantioselective annulations of DACs have been reported, mostly supposed to proceed through a DyKAT mechanism.[^7a^, ^10b^, ^16c^, ^17^] Preliminary investigations using Yb^III^‐ or other lanthanide‐based catalysts were not successful (see Supporting Information). We then examined MgI_2_ in the presence of PyBox ligands.^[8d]^ While these complexes indeed gave asymmetric induction, we could not exceed a 31 : 69 e.r., with (*S*)‐CyPyBox **L2** (Table 2, entry 1). The result previously obtained with Cu(OTf)_2_ and *rac*‐Box **L1** then urged us to focus on this class of complexes. Cu^II^/Box catalysis had been successfully applied by Ghosez and co‐workers to the enantioselective [4+2] cycloaddition of azadienes and olefins.^[12e]^ Cyclohexyl‐containing bisoxazoline **L3** provided encouraging results (entry 2). Increasing the steric hindrance at the bridging position of the bisoxazoline was beneficial for the enantioselectivity. With diethyl substituted **L4**, up to 98 : 2 e.r. could be achieved in chlorobenzene (entry 3). Unfortunately, these conditions led to poor yield reproducibility. Trisoxazolines ligands (Tox), developed by Tang and co‐workers,[^10b^, ^17^, ^18^] were then examined. (*S*)‐CyTox **L5** stood out as optimal. Upon a solvent screening (entries 4–7), a good compromise between yield, diastereo‐ and enantioselectivity was found by running the reaction in a 6 : 4 mixture of toluene and DCM (entry 7). Under these conditions, the desired enantioenriched lactam was isolated in 75 % yield, 93 : 7 d.r. and excellent 97 : 3 e.r. (94 % ee). The Competing Enantioselective Conversion (CEC) method developed by Rychnovsky and co‐workers for cyclic secondary amines^[19]^ was applied on derivative **10** (see below and Supporting Information) to determine the absolute configuration of the major enantiomer as **(2*S*,5*R*)‐5 a.a**.

**Table 2:** Optimization and of asymmetric (4+3) annulation with azadiene **4 a**.

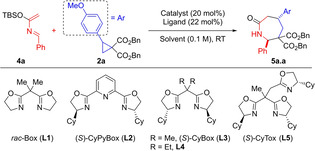						
Entry	Catalyst	Lig.	Solvent	Yield^[a]^	d.r.^[b]^	e.r.^[b]^
1	MgI_2_	**L2**	DCM	67 %	98 : 2	31 : 69
2	Cu(OTf)_2_	**L3**	DCM	85 %	89 : 11	88 : 12
3	Cu(OTf)_2_	**L4**	PhCl	35–90 %	95 : 5	98 : 2
4	Cu(OTf)_2_	**L5**	DCM	84 %	78 : 22	96 : 4
5	Cu(OTf)_2_	**L5**	PhCl	78 %	91 : 9	93 : 7
6	Cu(OTf)_2_	**L5**	Toluene	55 %	98 : 2	98 : 2
7^[c]^	Cu(OTf)_2_	**L5**	Tol./DCM (6/4)	75 %	93 : 7	97 : 3

Reaction conditions: 1.0 equiv cyclopropane **2 a**, 1.5 equiv azadiene **4 a**, 20 mol % catalyst, 22 mol % ligand, 60–70 mg 3 Å MS 0.10 M, at RT, overnight. [b] Isolated yield upon column chromatography. [c] d.r. and e.r. were measured by HPLC analysis.

The generality of this procedure was then tested on a selection of aryl and alkenyl cyclopropanes (Scheme 4). Full conversion and high levels of enantioinduction but lower yields were observed with other substrates (**5 a.a**, **b**, **e**, **k**). A scale‐up of the process could be done without any diminution of yield or stereoselectivity (**5 a.a**). Our enantioselective protocol proved effective also with different azadienes, delivering the corresponding azepanones with very good d.r. and excellent e.r. (**5 b**–**e.a**).^[20]^

**Scheme 4:**
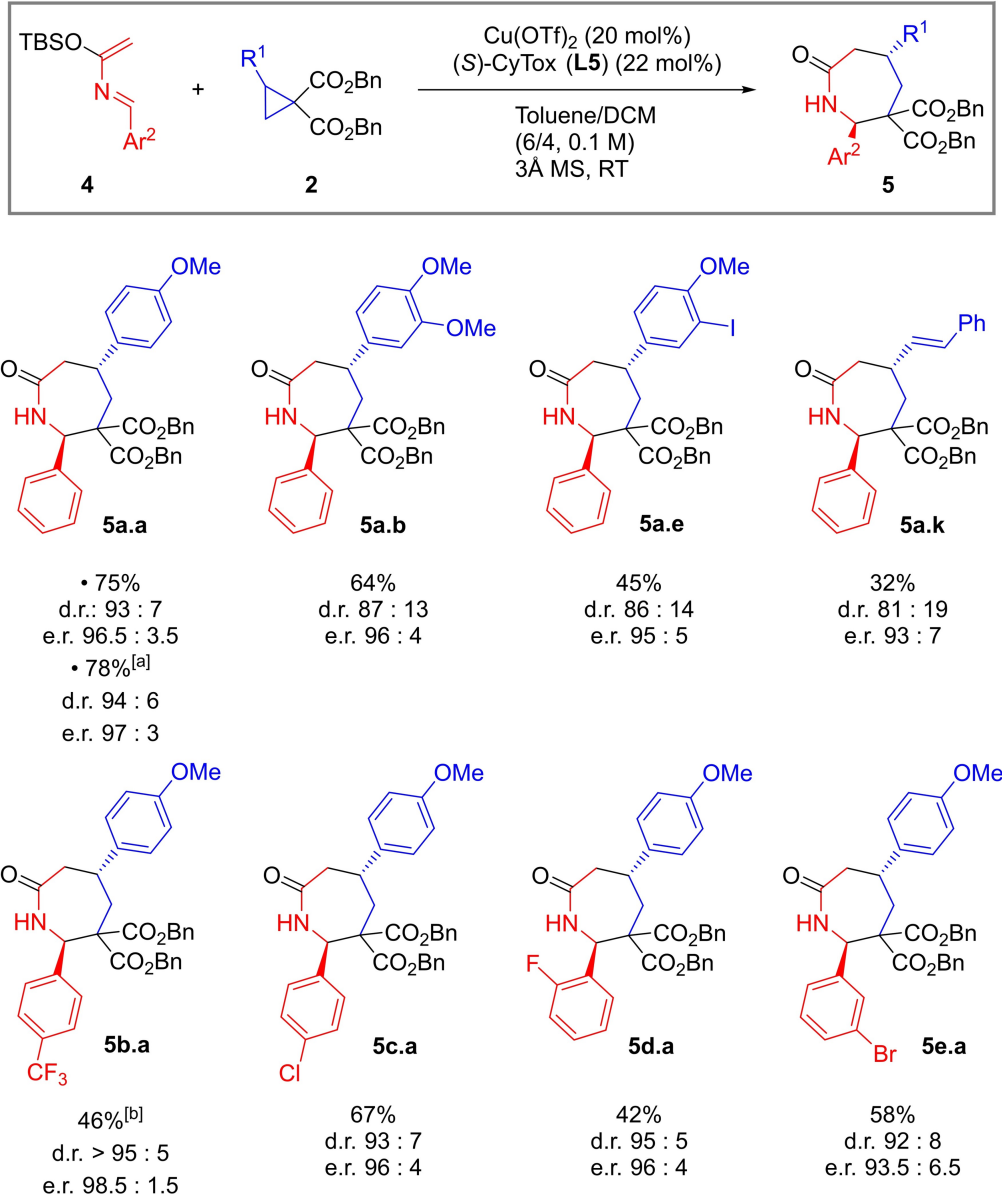
Scope of the enantioselective version of the (4+3) annulation. General conditions: 0.10 mmol (1.0 equiv) cyclopropane **2**, 0.15 mmol (1.5 equiv) azadiene **4**, 20 mol % Cu(OTf)_2_, 22 mol % (*S*)‐CyTox (**L5**), 60–70 mg 3 Å MS, Toluene (0.6 mL)/DCM (0.4 mL), RT, overnight. [a] Performed on 0.6 mmol scale. [b] 10 mol % Cu(OTf)_2_, 11 mol % (*S*)‐CyTox (**L5**).

We then examined synthetic modifications of the products (Scheme 5). Monocarboxylic acid **6** was easily obtained from diester **5 a.a** through a hydrogenolysis/decarboxylation sequence.^[21]^ It could be then converted into alkyne **7** in good yield, using a photoredox organocatalytic decarboxylative alkynylation.^[22]^ Alternatively, the complete decarboxylation of **6** was achieved under Barton conditions to give lactam **8**.^[23]^ The reduction of the tertiary amide obtained by N‐methylation of **5 a.a** was achieved via sequential treatment of the latter with Meerwein salt and sodium borohydride.^[24]^ Under these conditions, fragmentation was observed in addition to reduction, and acyclic benzylamine **9** was formed in moderate yield. The completely saturated azepane **10** was obtained by reduction of **8** with LiAlH_4_. Interestingly, when **5 a.l** was reacted with ethylenediamine,^[25]^ bicyclic dilactam **11** was obtained in very good yield though an amidation reaction of the newly formed free amino group and the *syn*‐oriented ester.

**Scheme 5:**
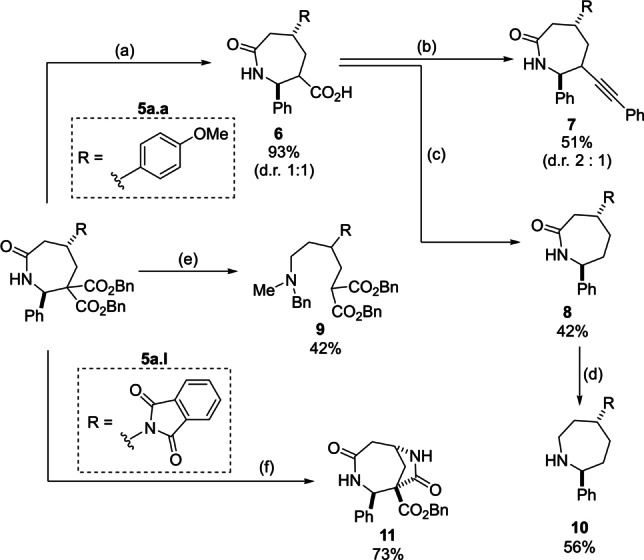
Modification of products **5**. Reaction conditions: a) 1. H_2_, Pd/C (10 mol %), MeOH/EtOAc (1/1); 2. Cu_2_O, MeCN, 80 °C. b) 4‐CzIBn (5 mol %), Ph‐EBX (1.5 equiv), Cs_2_CO_3_ (1.5 equiv), DCM, 25 °C, Kessil lamp (440 nm). c) 1. 2‐Mercaptopyridine N‐oxide (1.25 equiv), EDCI⋅HCl (2.0 equiv), DMAP (20 mol %), DCM, 0–25 °C; 2. ^n^Bu_3_SnH (3.0 equiv), AIBN (10 mol %), toluene, 80 °C. Yield provided over 2 steps. d) LiAlH_4_ (2.5 equiv), THF, 75–50 °C. e) 1. NaH (1.2 equiv), MeI (3.0 equiv), DMF/THF, 0 to 25 °C; 2. Me_3_OBF_4_ (3.0 equiv), 2,6‐di‐*tert*Bu‐Py (3.3 equiv), DCM, 25 °C then NaBH_4_ (10 equiv) and MeOH, 0 °C. f) Ethylenediamine (5.0 equiv), DCM/MeOH, 38 °C.

In summary, a (4+3) annulation of donor‐acceptor cyclopropanes with azadienes was disclosed. This easily scalable transformation occurred under mild conditions, using Yb(OTf)_3_ as the catalyst. Densely substituted azepanones could be synthesized in a single step in good to excellent yields and with high degrees of diastereoselectivity. The scope of the reaction included both electron‐rich (hetero)aryl and alkenyl, and amino‐substituted cyclopropanes. The development of an asymmetric version was possible using Cu(OTf)_2_ as catalyst and trisoxazoline ligand (S)‐CyTox (**L5**). While our method gives access to products of high interest for synthetic and medicinal chemistry, it also highlights the synthetic utility of azadienes in organic synthesis, which has been only scarcely investigated in the past. Further applications of these reagents are currently under investigation in our laboratories.

## Conflict of interest

The authors declare no conflict of interest.

## Supporting information

As a service to our authors and readers, this journal provides supporting information supplied by the authors. Such materials are peer reviewed and may be re‐organized for online delivery, but are not copy‐edited or typeset. Technical support issues arising from supporting information (other than missing files) should be addressed to the authors.

Supporting Information

Supporting Information

## Data Availability

The data that support the findings of this study are available in the Supporting Information of this article.
