# Supplementary material for: (4+3) Annulation of Donor‐Acceptor Cyclopropanes and Azadienes: Highly Stereoselective Synthesis of Azepanones
Source: Angew Chem Int Ed Engl. 2022 Jul 28;61(36):e202209006. doi: 10.1002/anie.202209006 (PMC9545371; doi:10.1002/anie.202209006)

checkCIF/PLATON report

Structure factors have been supplied for datablock(s) ste-20-131bni

THIS REPORT IS FOR GUIDANCE ONLY. IF USED AS PART OF A REVIEW PROCEDURE FOR PUBLICATION, IT SHOULD NOT REPLACE THE EXPERTISE OF AN EXPERIENCED CRYSTALLOGRAPHIC REFEREE.

No syntax errors found. CIF dictionary Interpreting this report

Datablock: ste-20-131bni

```
Bond precision:  C-C = 0.0020 A          Wavelength=1.54184

Cell:           a=10.2442(3)      b=12.3459(3)      c=12.6199(3)
                alpha=96.478(2)   beta=109.519(3)   gamma=103.169(2)
Temperature:    140 K

                Calculated                Reported
Volume          1433.42(7)                1433.43(7)
Space group     P -1                      P -1
Hall group      -P 1                      -P 1
Moiety formula  C35 H33 N O6              C35 H33 N O6
Sum formula     C35 H33 N O6              C35 H33 N O6
Mr              563.62                    563.62
Dx,g cm-3       1.306                     1.306
Z               2                         2
Mu (mm-1)       0.721                     0.721
F000            596.0                     596.0
F000'           597.84
h,k,lmax        12,15,15                  12,15,15
Nref            5986                      5924
Tmin,Tmax       0.753,0.847               0.785,0.885
Tmin'           0.657

Correction method= # Reported T Limits: Tmin=0.785 Tmax=0.885
AbsCorr = ANALYTICAL

Data completeness= 0.990          Theta(max)= 75.914

R(reflections)= 0.0403( 5708)     wR2(reflections)= 0.1050( 5924)

S = 1.043                  Npar= 548
```

The following ALERTS were generated. Each ALERT has the format
test-name_ALERT_alert-type_alert-level.
Click on the hyperlinks for more details of the test.

Alert level C

```
PLAT906_ALERT_3_C Large K Value in the Analysis of Variance ......      2.184 Check
PLAT911_ALERT_3_C Missing FCF Refl Between Thmin & STh/L=    0.600          3 Report
```

Alert level G

```
PLAT002_ALERT_2_G Number of Distance or Angle Restraints on AtSite          5 Note
PLAT003_ALERT_2_G Number of Uiso or Uij Restrained non-H Atoms ...         15 Report
PLAT176_ALERT_4_G The CIF-Embedded .res File Contains SADI Records          2 Report
PLAT178_ALERT_4_G The CIF-Embedded .res File Contains SIMU Records          1 Report
PLAT301_ALERT_3_G Main Residue  Disorder ..............(Resd  1  )        17% Note
PLAT793_ALERT_4_G Model has Chirality at C3          (Centro SPGR)          R Verify
PLAT793_ALERT_4_G Model has Chirality at C6          (Centro SPGR)          R Verify
PLAT860_ALERT_3_G Number of Least-Squares Restraints .............        224 Note
PLAT912_ALERT_4_G Missing # of FCF Reflections Above STh/L=  0.600         59 Note
PLAT933_ALERT_2_G Number of OMIT Records in Embedded .res File ...          1 Note
PLAT941_ALERT_3_G Average HKL Measurement Multiplicity ...........        2.6 Low
PLAT978_ALERT_2_G Number C-C Bonds with Positive Residual Density.         14 Info
PLAT992_ALERT_5_G Repd & Actual _reflns_number_gt Values Differ by          4 Check
```

0 **ALERT level A** = Most likely a serious problem - resolve or explain
0 **ALERT level B** = A potentially serious problem, consider carefully
2 **ALERT level C** = Check. Ensure it is not caused by an omission or oversight
13 **ALERT level G** = General information/check it is not something unexpected

0 ALERT type 1 CIF construction/syntax error, inconsistent or missing data
4 ALERT type 2 Indicator that the structure model may be wrong or deficient
5 ALERT type 3 Indicator that the structure quality may be low
5 ALERT type 4 Improvement, methodology, query or suggestion
1 ALERT type 5 Informative message, check

It is advisable to attempt to resolve as many as possible of the alerts in all categories. Often the minor alerts point to easily fixed oversights, errors and omissions in your CIF or refinement strategy, so attention to these fine details can be worthwhile. In order to resolve some of the more serious problems it may be necessary to carry out additional measurements or structure refinements. However, the purpose of your study may justify the reported deviations and the more serious of these should normally be commented upon in the discussion or experimental section of a paper or in the "special_details" fields of the CIF. checkCIF was carefully designed to identify outliers and unusual parameters, but every test has its limitations and alerts that are not important in a particular case may appear. Conversely, the absence of alerts does not guarantee there are no aspects of the results needing attention. It is up to the individual to critically assess their own results and, if necessary, seek expert advice.

Publication of your CIF in IUCr journals

A basic structural check has been run on your CIF. These basic checks will be run on all CIFs submitted for publication in IUCr journals (*Acta Crystallographica*, *Journal of Applied Crystallography*, *Journal of Synchrotron Radiation*); however, if you intend to submit to *Acta Crystallographica Section C* or *E* or *IUCrData*, you should make sure that full publication checks are run on the final version of your CIF prior to submission.

Publication of your CIF in other journals

Please refer to the *Notes for Authors* of the relevant journal for any special instructions relating to CIF submission.

PLATON version of 18/09/2020; check.def file version of 20/08/2020

Datablock ste-20-131bni - ellipsoid plot

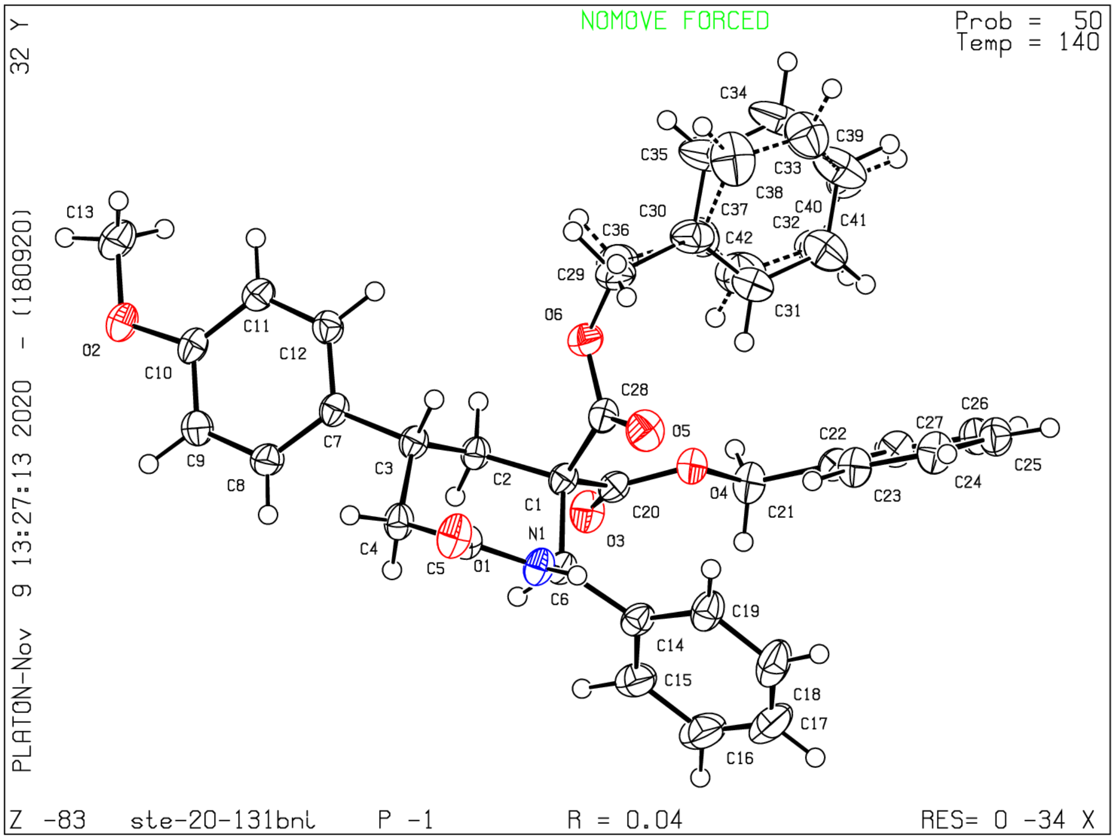

Supplement: Supplementary file 1 — Supporting Information [file ANIE-61-0-s002.pdf]
