# Supplementary material for: (4+3) Annulation of Donor‐Acceptor Cyclopropanes and Azadienes: Highly Stereoselective Synthesis of Azepanones
Source: Angew Chem Int Ed Engl. 2022 Jul 28;61(36):e202209006. doi: 10.1002/anie.202209006 (PMC9545371; doi:10.1002/anie.202209006)
Supplement: Supplementary file 2 — Supporting Information [file ANIE-61-0-s001.pdf]

Angewandte Chemie
Eine Zeitschrift der Gesellschaft Deutscher Chemiker

Supporting Information

(4+3) Annulation of Donor-Acceptor Cyclopropanes and Azadienes: Highly Stereoselective Synthesis of Azepanones

S. Nicolai, J. Waser**

Supporting Information
©Wiley-VCH 2021
69451 Weinheim, Germany

(4+3) Annulation of Donor-Acceptor Cyclopropane and Azadienes: Highly Stereoselective Synthesis of Azepanones

Stefano Nicolai* and Jérôme Waser*

Abstract: Azepanes are important seven-membered heterocyclic scaffolds, which are present in many natural and bioactive compounds. However, the development of convergent synthetic methods to access them remains challenging. Herein, we report the Lewis acid catalyzed (4+3) annulative addition of aryl and amino donor-acceptor cyclopropanes with 2-aza-1,3-dienes. Densely substituted azepane derivatives were obtained in good to excellent yields and with high diastereoselectivity. The reaction occurred under mild conditions with ytterbium triflate as the catalyst. The use of copper triflate with a trisoxazoline (Tox) ligand led to an enantioselective version of the transformation. The obtained cycloadducts were convenient substrates for a series of further modifications, showing the synthetic utility of these compounds.

DOI: 10.1002/anie.202209006

Table of Contents

Experimental Procedures	S1
Results and discussion	S2
1. Preparation of the Azadienes (Compounds **1** and **4**)	S2
2. Preparation of the Cyclopropropanes (Compounds **2**)	S12
3. Optimization of the reaction	S19
3.1 Speculative model for diastereoselectivity	S24
4. Scope of the reaction and characterization of the (4+3) annulation products	S24
5. Optimization and scope of the asymmetric version of the reaction	S34
5.1 Synthesis of the ligands	S34
5.1.1 PyBox ligands: Tested ones and preparation of the non-commercially availables ligands	S34
5.1.2 Box ligands: Tested ones and preparation of the non-commercially availables ligands	S35
5.1.3 Preparation of other ligands that were tested during the optimization	S47
5.2 Optimization of the Enantioselective reaction	S47
5.3 Scope of the Enantioselective reaction	S50
6. Modifications of the (4+3) annulation products	S57
7. Determination of the absolute configuration of the enantioenriched cycloadduct by CEC	S61
References	S66
Crystal Data and Experimental for **5a.a**	S68
NMR Spectra of new compounds	S76

Experimental Procedures

The NMR spectra were recorded on a Brucker DPX-400 spectrometer at 400 MHz for ^{1}H, 101 MHz for ^{13}C, 376 MHz for ^{19}F. The chemical shift (δ) for ^{1}H and ^{13}C are given in ppm relative to residual signals of the solvents (chloroform-*d* - 7.26 ppm ^{1}H NMR and 77.12 ppm ^{13}C NMR; methylene chloride-d_2 5.32 ppm ^{1}H NMR and 53.8 ppm ^{13}C NMR; acetonitrile-d_3 1.92 ppm ^{1}H NMR and 1.4 and 118.7 ppm ^{13}C NMR; dmso-d_6 2.50 ppm ^{1}H NMR and 39.52 ppm ^{13}C NMR). ^{13}C and ^{19}F spectra have been measured using broadband {1H} decoupling. Coupling constants are given in Hertz. The following abbreviations are used to indicate the multiplicity: s, singlet; d, doublet; q, quartet; m, multiplet or massive; bs, broad signal). Infrared spectra of selected compounds were recorded on a JASCO FT-IR B4100 spectrophotometer with an ATR PRO410-S and a ZnSe prisma and are reported as cm-1 (w = weak, m = medium, s = strong, br = broad). High resolution mass spectrometric measurements were performed by the mass spectrometry service of ISIC at the EPFL on a MICROMASS (ESI) Q-TOF Ultima API. The raw data obtained from the Q-TOF Waters instrument does not take into account the mass of the electron for the ion, the obtained raw data has been therefore corrected by removing the mass of the electron (5 mDa). Mass spectrometry for CEC reactions was performed on UPLC-MS system consisting of a Waters Acquity UPLC and a Waters VION IMS QTOF. Samples were analyzed using Waters Acquity-I-UPLC Classsystem (Waters Corporation, Milford, MA, USA) coupled with a Waters Vion IMS-QTof Mass Spectrometer equipped with LockSpray. The instrument was controlled by Waters UNIFI 1.9.4 (3.1.0, Waters Corporation, Milford, MA, USA). The diffraction data for crystal structures were collected by mass spectrometry service of ISIC at the EPFL at low temperature using Cu (323) or Mo (520) Ka radiation on a Rigaku SuperNova dual system in combination with Atlas type CCD detector. The data reduction and correction were carried out by CrysAlisPro (Rigaku Oxford Diffraction, release 1.171.40.68a, 2019). The solutions and refinements were performed by SHELXT1 and SHELXL2, respectively. The crystal structures were refined using full-matrix least-squares based on F2 with all non-H atoms defined in anisotropic manner. Hydrogen atoms were placed in calculated positions by means of the “riding” model. Yields of isolated products refer to materials of >95% purity as determined by ^{1}H NMR.

The authors are indebted to the team of the research support service of ISIC at EPFL, particularly to the NMR, X-Ray, and the High Resolution Mass Spectrometry Units.

General Procedures. All reactions were set up under a nitrogen atmosphere in oven-dried glassware using standard Schlenk techniques, unless otherwise stated. Synthesis grade solvents were used as purchased; anhydrous solvents (THF, Et_2O, Toluene, DCM and MeCN) were taken from a commercial SPS solvent dispenser (H_2O content < 10 ppm, Karl-Fischer titration). Chromatographic purification of products was accomplished using flash chromatography (FC) on SilicaFlash P60 silica gel (230 - 400 mesh). When specified, Ultra was used for elution (Ultra: 3/1 DCM/MeOH + 5% aq. NH_3 (25% v/v)). For thin layer chromatography (TLC) analysis throughout this work, Pre-coated Suplaco silica gel 60 F_{254} TLC glass plates were employed, using UV light as the visualizing agent and acidic ethanolic p-anisaldehyde or basic aqueous potassium permanganate ($KMnO_4$) stain solutions, and heat as developing agents. Organic solutions were concentrated under reduced pressure on a Büchi rotatory evaporator. Determination of Enantiomeric Purity: HPLC analysis on chiral stationary phase was performed on an Agilent Acquity instrument using a Daicel CHIRALPAK IA chiral column. The exact conditions for the analyses are specified within the characterization section. HPLC traces were compared to racemic samples prepared according to the procedure **GP6**.

Materials. Most of the starting materials used in this study are commercial and were purchased in the highest purity available from Sigma-Aldrich, Fluka, Alfa Aesar, Fluorochem. Synthesis grade solvents were used as purchased; anhydrous solvents (THF, Et_2O, Toluene, DCM and MeCN) were taken from a commercial SPS solvent dispenser (H_2O content < 10 ppm, Karl-Fischer titration).

Results and Discussion

1. Preparation of the Azadienes (Compounds 1 and 4)

Isopropyl (*E*)-N-(1-((tert-butyldimethylsilyl)oxy)vinyl)formimidate (1)

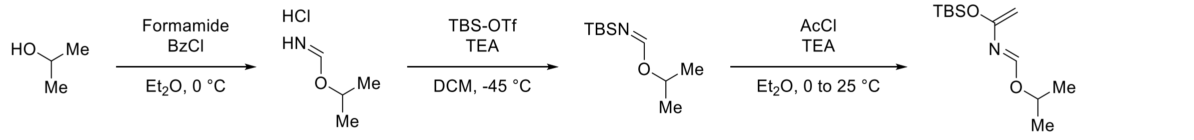

S1 **S2** **1**

Following a reported procedure,[1] in a 100 mL, two-necked, round-bottomed flask, benzoyl chloride (5.2 mL, 45 mmol, 1.0 equiv.) was dissolved in Et_2O (dry; 37 mL). The solution was kept at ca. 20 °C using a water bath. A solution of formamide (1.8 mL, 45 mmol, 1.01 equiv.) in isopropanol (dry; 3.5 mL, 45 mmol, 1.01 equiv.) was then added drop-wise over a period of 45 minutes, using a syringe pump. The mixture became immediately turbid and a solid started precipitating after ca. 50% of the addition. Once the addition was completed, the suspension was stirred at room temperature for another 2 hours. The crystalline white solid was then collected by filtration and washed with Et_2O (60 mL) to provide pure isopropyl formimidate hydrochloride (**S1**) (3.60 g, 29.1 mmol, 90% yield) as a white solid (stored at -20 °C).

^{1}H NMR (400 MHz, DMSO-d_6) δ 11.79 (s, 2H, NH_2^+), 8.80 (s, 1H, NC*H*), 5.10 (p, *J* = 6.2 Hz, 1H, OC*H*), 1.37 (d, *J* = 6.3 Hz, 6H, CH(*Me*)$_2$).
^{1}H-NMR data corresponded to the reported values.[1]

Following a reported procedure,[2] in a 25 mL, two-necked, round-bottomed flask, isopropyl formimidate hydrochloride (1.20 g, 9.71 mmol, 1.0 equiv.) was suspended in DCM (12 mL). The suspension was chilled to -45 °C (dry ice - acetone bath). Triethylamine (recently distilled over CaH_2 and stocked under Ar; 3.0 mL, 21 mmol, 2.2 equiv.) was added rapidly in a single portion, resulting in the formation of a thicker suspension (difficult to stirr). A solution of *tert*-butyldimethylsilyl trifluoromethanesulfonate (2.2 mL, 9.7 mmol, 1.0 equiv.) in DCM (6.0 mL) was added drop-wise at the same temperature: the suspension became slightly yellow and clearer. Once the addition was completed, hexane (15 mL) was added and the cooling bath was removed and the mixture was allowed to warm up. When it was at room temperature, the solids were filtered off through a pad of celite, which was then washed with several portions of hexane. The filtrate was concentrated under reduced pressure. Some solid appeared during the evaporation of the solvent. Dry Et_2O (ca. 5 mL) was added and the solid was allowed to separate by decantation. The etheral solution was collected by syringe, transferred into a vial, and concentrated under reduced pressure. Crude (*E*)-isopropyl N-(*tert*-butyldimethylsilyl)formimidate (**S2**) (1.95 g, 9.71 mmol, quantitative yield) was obtained as a colorless oil, in high purity, and used directly for the following step.

^{1}H NMR (400 MHz, Methylene Chloride-d_2) δ 7.64 (s, 1H, NC*HO*), 5.09 (pd, *J* = 6.2, 1.0 Hz, 1H, OC*H*), 1.21 (d, *J* = 6.3 Hz, 6H, CH(*Me*)$_2$), 0.88 (s, 9H, SiC*Me*$_3$), 0.07 (s, 6H, Si*Me*$_2$).
^{1}H-NMR data corresponded to the reported values.[2]

Following a reported procedure,[2] in a 25 mL vial, (*E*)-isopropyl N-(tert-butyldimethylsilyl)formimidate (2.01 g, 9.98 mmol, 1.0 equiv.) was dissolved in Et_2O (dry; 10 mL). The solution was cooled to 0 °C (ice - water bath) prior to the addition of triethylamine (5.6 mL, 40 mmol, 4.0 equiv.) in one portion. A solution of acetyl chloride (0.72 mL, 10 mmol, 1.0 equiv.) in Et_2O (2.0 mL) was

then added drop-wise, resulting in the immediate formation of a thick suspension. Stirring was maintained for two hours at room temperature. After this time, the mixture had turned from white to pale yellow. The precipitate (triethylamine hydrochloride) was filtered off on a pad celite and washed with two portions of ether. The filtrate was concentrated under reduced pressure to provide a yellow orange crude oil. The latter was submitted to kugelrohr distillation (70-90 °C; 0.85 mbar) to afford (*E*)-isopropyl N-(1-((*tert*-butyldimethylsilyl)oxy)vinyl)formimidate (**1**) (1.34 g, 5.52 mmol, 55% yield) as a colorless oil, which was stored under argon at -80 °C.

^{1}H NMR (400 MHz, Methylene Chloride-d_2) δ 7.90 (s, 1H, NC*H*), 5.03 (m, 1H, OC*H*), 3.92 (s, 1H, C=C*H₂*), 3.75 (s, 1H, C=C*H₂*), 1.27 (d, *J* = 6.2 Hz, 6H, CH(*Me*)$_2$), 0.95 (s, 9H, SiC*Me*$_3$), 0.20 (s, 6H, Si*Me*$_2$).
^{13}C NMR (101 MHz, Methylene Chloride-d_2) δ 155.6, 82.7, 70.1, 26.1, 22.2, 18.7, -4.4.
^{1}H-NMR data corresponded to the reported values.[2]

General procedures for the synthesis of aryl-substituted azadienes

GP1: N-Silylation of benzyl amines

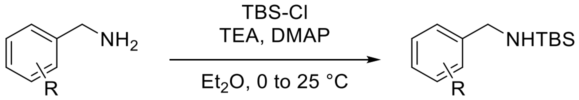

Following a reported procedure,[3] in a 100 mL, two-necked, round-bottomed flask, the benzyl amine (1.0 equiv.) was dissolved in Et_2O (2.0 mL per mmol of amine). The resulting solution was cooled to 0 °C (ice - water bath); triethylamine (1.15 equiv.) was added together with a catalytic amount of DMAP (2 mol%). The solution was stirred for another 3-4 minutes at 0 °C, and then a solution of *tert*-butyldimethylsilyl chloride (1.05 equiv.) in Et_2O (2.0 mL per mmol of amine) was added drop-wise over a period of about 5 minutes. Immediately, a white solid started to precipitate. The mixture was stirred over 12-60 hours, while progressively allowing to reach room temperature. The solids were then filtered off on paper, and washed with dry diethyl ether. The slightly turbid filtrate was concentrated under reduced pressure and then triturated with dry pentane, which led to further precipitation. The precipitate was again removed by filtration on paper, followed by washing with additional dry pentane. The resulting solution was concentrated under vacuum, and then submitted to bulb-to-bulb distillation, to provide the desired N-TBS amine as an oil.

GP2: Oxidation of N-TBS amine to the corresponding N-TBS imine.

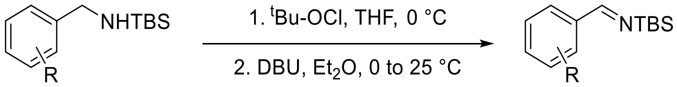

Following a slightly modified version of a reported procedure in two steps:[3]
First step: in a 100 mL, two-necked, round-bottomed flask, the N-TBS benzyl amine (1.0 equiv.) was dissolved in THF (1.5 mL per mmol of the N-TBS benzyl imine). The solution was cooled to 0 °C (ice - water bath). Under stirring, freshly prepared *tert*-butyl hypochlorite (1.0-1.05 equiv.)[1] was added drop-wise. Once the addition was finished, the resulting pale yellow mixture was stirred at 0 °C for 2-3 hours. The resulting pale yellow solution was concentrated under reduced pressure. To the residue, Et_2O (0.27 mL per mmol of the N-TBS benzyl imine) was added, which led to the precipitation of a sticky solid. The latter was removed by filtration (using a 25 mm syringe filter, 22 μm). [2]
Second step: The clear filtrate was directly collected a 50-100 mL, two-necked, round-bottom flask, and cooled to 0 °C (ice - water bath). A solution of DBU (1.05 equiv.) in Et_2O (1-4 mL) was added drop-wise. Some heterogeneous precipitation was initially observed; by the end of the addition, the mixture looked like a homogeneous suspension. The cooling bath was removed and stirring was continued at room temperature overnight. After 14-18 hours, the suspension looked yellow and thicker. The solids were removed by filtration on paper and washed with dry ether. The filtrate was concentrated under vacuum, and the pale yellow residue was redissolved in pentane (ca. 15 mL). The turbid organic solution was rapidly washed with ice-cold brine, dried over Na_2SO_4, and filtered. Upon concentration under reduced pressure, the N-TBS imine was collected as a (pale) yellow oil, which was used directly in the following step.

GP3: Conversion of the N-TBS imine into the aryl azadiene

[1] *tert*-Butyl hypochlorite was prepared each time following the protocol reported by Sonnet and co-workers:[60] In a 100 mL, single-necked, round-bottomed flask, bleach (50 mL) was cooled to 0 °C. The flask was covered with aluminium foil. ^{t}BuOH (3.7 mL, 39 mmol, 1 equiv.) and glacial AcOH (2.5 mL, 43 mmol, 1.1 equiv.) were then rapidly added. The resulting mixture was stirred for 5 minutes, then the organic layer was separated and washed with sat. aq. $NaHCO_3$ (5 mL) and then with water (5 mL). The organic layer was collected and dried over $CaCl_2$. *tert*-Butyl hypochlorite (2.56 g, 23.6 mmol, 61% yield) was obtained as a bright yellow oil, and it was stored at -20 °C in the presence of some grains of $CaCl_2$ prior to being used (few hours at longest).
[2] In some cases, the initially clear filtrate became slightly turbid upon being cooled to 0 °C. A second filtration was performed in such situations.

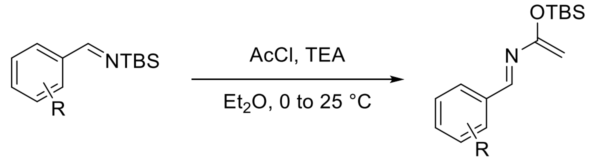

Following a reported procedure,[4] a 100 mL, single-necked, round bottom flask was charged with the N-TBS imine (1.0 equiv.) and a large stirring bar; it was then sealed with a septum, evacuated and backfilled with nitrogen (3 times). Et_2O (1 mL per mmol of the N-TBS imine) was added by syringe and the resulting yellow solution was cooled to 0 °C (ice - water bath). Triethylamine (4.0 equiv.) was added in a single portion. Finally, a solution of acetyl chloride (2.1 equiv.) in Et_2O (0.9 mL mmol of the N-TBS imine) was added slowly (but not drop-wise). Immediately, the solution converted into a thick, pale yellow suspension, which was stirred at 0 °C for 5 minutes, and then at room temperature for 3 hours, lightly darkening to pale orange during this time. The solids were then removed by rapid filtration through a pad of celite, which was then washed with several portions of pentane. The bright yellow filtrate was concentrated under reduced pressure, to give a turbid, orange crude oil. The latter was diluted with pentane and the organic solution was washed rapidly with ice-cold water (20 mL), ice-cold brine, dried over Na_2SO_4, filtered, and concentrated under vacuum. The now clear orange oil was submitted to column chromatography (Biotage flash chromatographer, 25-80 g SiO_2; the column was pre-treated with 200-400 mL of NEt_3 in pentane, 2.5% v/v; equilibration was then done with a 97/3 mixture of pentane and the aforementioned solution; elution was done with pure pentane). The azadiene was obtained as a yellow oil, which was stored under nitrogen at -80 °C.[3,4]

N-Benzylidene-1-((*tert*-butyldimethylsilyl)oxy)ethenamine (4a)

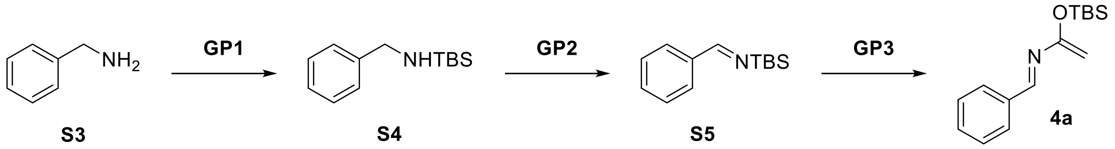

Starting from benzyl amine (**S3**) (3.0 mL, 27 mmol, 1.0 equiv.) with triethylamine (4.4 mL, 32 mmol, 1.15 equiv.), DMAP (0.067 g, 0.55 mmol, 2 mol%) and *tert*-butyldimethylsilyl chloride (4.34 g, 28.8 mmol, 1.05 equiv.), and following **GP1**. N-Benzyl-1-(*tert*-butyl)-1,1-dimethylsilanamine (**S4**) (5.3 g, 24 mmol) was obtained in 87% yield as a colorless oil upon kugelrohr distillation (110-120 °C, 1.0-0.9 mbar).

^{1}H NMR (400 MHz, Chloroform-*d*) δ 7.33 (m, 2H, Ar*H*), 7.32 (d, *J* = 2.3 Hz, 2H, Ar*H*), 7.22 (ddt, *J* = 6.1, 4.9, 2.6 Hz, 1H, Ar*H*), 3.98 (s, 2H, CH_2Ar), 3.01 (s, 1H, TBS-N*H*), 0.93 (s, 9H, SiCMe_3), 0.05 (s, 6H, SiMe_2).
^{1}H-NMR data corresponded to the reported value.[5]

The oxidation of N-benzyl-1-(*tert*-butyl)-1,1-dimethylsilanamine (**S4**) (5.33 g, 24.1 mmol, 1.0 equiv.) to N-(*tert*-butyldimethylsilyl)-1-phenylmethanimine (**S5**) was performed following the **GP2**, and using *tert*-butyl hypochlorite (2.7 mL, 26 mmol, 1.05 equiv.) in the first step, and DBU (3.8 mL, 25 mmol, 1.05 equiv.) in the second. Upon quick aqueous work-up, **S5** (5.23 g, 23.8 mmol, 99% yield) was collected as a yellow oil, which was not further purified.

Following **GP3**, N-(*tert*-butyldimethylsilyl)-1-phenylmethanimine (**S5**) (4.34 g, 19.8 mmol, 1.0 equiv.) was reacted with triethylamine (11.3 mL, 81.1 mmol, 4.1 equiv.) and acetyl chloride (3.0 mL, 42 mmol, 2.1 equiv.). Upon quick aqueous work-up, the crude orange oil was submitted to column chromatography (Biotage flash chromatographer, 80 g SiO_2; the column was primed with 300 mL of NEt_3 in pentane, 2.5% v/v; equilibration was then done with a mixture of pentane and the aforementioned solution 95/5; elution was done with pure pentane) to provide N-benzylidene-1-((tert-butyldimethylsilyl)oxy)ethenamine (**4a**) (3.76 g, 14.4 mmol, 73% yield) as a bright yellow oil (stable at -80 °C for at least 4 months).

^{1}H NMR (400 MHz, Methylene Chloride-d_2) δ 8.55 (s, 1H, N*CH*Ar), 7.85 – 7.77 (m, 2H, Ar*H*), 7.50 – 7.38 (m, 3H, Ar*H*), 4.60 (s, 1H, C=CH_2), 4.30 (s, 1H, C=CH_2), 1.02 (s, 9H, SiCMe_3), 0.25 (s, 6H, SiMe_2).
^{13}C NMR (101 MHz, Methylene Chloride-d_2) δ 157.5, 156.8, 136.6, 131.7, 129.4, 129.2, 92.4, 26.1, 18.9, -4.5.
^{1}H-NMR data corresponded to the reported value.[6] ^{13}C NMR signals are systematically shifted of around -4.5 ppm compared to the same reference.

N-(1-((*tert*-Butyldimethylsilyl)oxy)vinyl)-1-(4-(trifluoromethyl)phenyl)methanimine (4b)

3 Most of azadienes could be stored at -80 °C without significant decomposition for at least 3-4 months. By contrast, with chloro-aryl, bromo-aryl and trifluoromethyl-aryl substituted azadienes, polymerization and conversion into gels were observed after a few days since their purification: these azadienes should be used within 3 days after their synthesis at latest.

4 Azadienes were too unstable under ambient conditions to undergo HRMS or elemental analyses. This characterization data could not be therefore acquired.

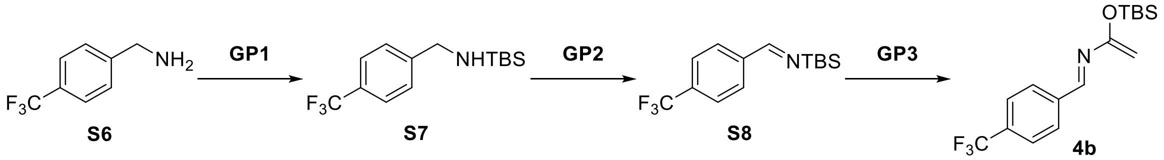

Starting from (4-(trifluoromethyl)phenyl)methanamine (**S6**) (1.7 mL, 12 mmol, 1.0 equiv.) with triethylamine (1.9 mL, 14 mmol, 1.15 equiv.), DMAP (0.029 g, 0.24 mmol, 2 mol%) and *tert*-butyldimethylsilyl chloride (1.9 g, 13 mmol, 1.05 equiv.), and following **GP1**. 1-*tert*-Butyl-1,1-dimethyl-N-(4-(trifluoromethyl)benzyl)silanamine (**S7**) (2.87 g, 9.91 mmol) was obtained in 83% yield as a colorless oil upon kugelrohr distillation (105-110 °C, 0.84 mbar).

^{1}H NMR (400 MHz, Chloroform-*d*) δ 7.56 (m, 2H, Ar*H*), 7.43 (m, 2H, Ar*H*), 4.03 (s, 2H, *CH₂*Ar), 0.92 (s, 9H, SiC*Me*$_3$), 0.75 (br s, 1H, TBS-N*H*), 0.05 (s, 6H, Si*Me*$_2$).
^{13}C NMR (101 MHz, Chloroform-*d*) δ 148.7, 128.7 (d, *J* = 32.0 Hz), 127.0, 125.1 (q, *J* = 4.0 Hz), 124.4 (d, *J* = 271.8 Hz), 46.2, 26.4, 18.4, -5.0.
^{19}F NMR (376 MHz, Chloroform-*d*) δ -62.2.

The oxidation of 1-*tert*-butyl-1,1-dimethyl-N-(4-(trifluoromethyl)benzyl)silanamine (**S7**) (2.80 g, 9.67 mmol, 1.0 equiv.) to N-(tert-butyldimethylsilyl)-1-(4-(trifluoromethyl)phenyl)methanimine (**S8**) was performed following the **GP2**, and using *tert*-butyl hypochlorite (1.1 mL, 10 mmol, 1.05 equiv.) in the first step, and DBU (1.5 mL, 10 mmol, 1.05 equiv.) in the second. Upon quick aqueous work-up, **S8** (2.02 g, 7.02 mmol, 73% yield) was collected as a gold yellow oil, which was not further purified.

Following **GP3**, N-(tert-butyldimethylsilyl)-1-(4-(trifluoromethyl)phenyl)methanimine (**S8**) (2.02 g, 7.02 mmol, 1.0 equiv.) was reacted with triethylamine (4.0 mL, 29 mmol, 4.1 equiv.) and acetyl chloride (1.0 mL, 15 mmol, 2.1 equiv.). Upon quick aqueous work-up, the crude orange oil was submitted to column chromatography (Biotage flash chromatographer, 25 g SiO_2; the column was pre-treated with 250 mL of NEt_3 in pentane, 2.5% v/v; equilibration was then done with a mixture of pentane and the aforementioned solution 97/3; elution was done with pure pentane) to provide N-(1-((*tert*-butyldimethylsilyl)oxy)vinyl)-1-(4-(trifluoromethyl)phenyl)methanimine (**4b**) (1.56 g, 4.75 mmol, 68% yield) as a bright yellow oil (stable at -80 °C for 3-4 days).

^{1}H NMR (400 MHz, Chloroform-*d*) δ 8.58 (d, *J* = 1.3 Hz, 1H, N*CH*Ar), 7.94 (m, 2H, Ar*H*), 7.68 (m, 2H, Ar*H*), 4.75 (d, *J* = 1.5 Hz, 1H, C=*CH₂*), 4.41 (d, *J* = 1.5 Hz, 1H, C=*CH₂*), 1.02 (s, 9H, SiC*Me*$_3$), 0.26 (d, *J* = 1.8 Hz, 6H, Si*Me*$_2$).
^{13}C NMR (101 MHz, Chloroform-*d*) δ 156.3, 154.6, 139.1, 132.5 (q, *J* = 32.4 Hz), 129.1, 125.6 (q, *J* = 4.0 Hz), 123.9 (q, *J* = 272.1 Hz), 94.1, 25.8, 18.4, -4.8.
^{19}F NMR (376 MHz, Chloroform-*d*) δ -62.8.

N-(1-((*tert*-Butyldimethylsilyl)oxy)vinyl)-1-(4-chlorophenyl)methanimine (4c)

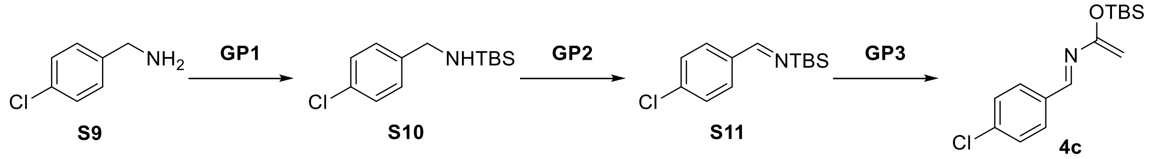

Starting from (4-chlorophenyl)methanamine (**S9**) (2.4 mL, 20 mmol, 1.0 equiv.) with triethylamine (3.2 mL, 21 mmol, 1.15 equiv.), DMAP (0.049 g, 0.40 mmol, 2 mol%) and *tert*-butyldimethylsilyl chloride (3.16 g, 21.0 mmol, 1.05 equiv.), and following **GP1**. 1-*tert*-Butyl-N-(4-chlorobenzyl)-1,1-dimethylsilanamine (**S10**) (4.40 g, 17.2 mmol) was obtained in 86% yield as a colorless oil upon kugelrohr distillation (130 °C, 1.2 mbar).

^{1}H NMR (400 MHz, Chloroform-*d*) δ 7.35 – 7.19 (m, 4H, Ar*H*), 3.94 (d, *J* = 7.9 Hz, 2H, *CH₂*Ar), 0.95 (s, 1 H, TBS-N*H*), 0.91 (s, 9H, SiC*Me*$_3$), 0.04 (s, 6H, Si*Me*$_2$).
^{13}C NMR (101 MHz, Chloroform-*d*) δ 143.1, 131.9, 128.3, 128.1, 45.9, 26.4, 18.4, -5.0.

The oxidation of 1-*tert*-butyl-N-(4-chlorobenzyl)-1,1-dimethylsilanamine (**S10**) (4.40 g, 17.2 mmol, 1.0 equiv.) to N-(*tert*-butyldimethylsilyl)-1-(4-chlorophenyl)methanimine (**S11**) was performed following the **GP2**, and using *tert*-butyl hypochlorite (2.2 mL, 19 mmol, 1.1 equiv.) in the first step, and DBU (2.7 mL, 18 mmol, 1.05 equiv.) in the second. Upon quick aqueous work-up, **S11** (3.03 g, 11.9 mmol, 69% yield) was collected as a gold yellow oil, which was not further purified.

Following **GP3**, N-(*tert*-butyldimethylsilyl)-1-(4-chlorophenyl)methanimine (**S11**) (3.03 g, 11.9 mmol, 1.0 equiv.) was reacted with triethylamine (6.8 mL, 49 mmol, 4.1 equiv.) and acetyl chloride (1.8 mL, 25 mmol, 2.1 equiv.). Upon quick aqueous work-up, the crude orange oil was submitted to column chromatography (25 g SiO_2; the column was primed with 250 mL of NEt_3 in pentane, 2.5% v/v; equilibration was then done with a mixture of pentane and the aforementioned solution 97/3; elution was done with pure pentane) to provide N-(1-((*tert*-butyldimethylsilyl)oxy)vinyl)-1-(4-chlorophenyl)methanimine (**4c**) (2.53 g, 8.55 mmol, 72% yield) as a bright yellow oil (stable at -80 °C for 3-4 days).

^{1}H NMR (400 MHz, Chloroform-*d*) δ 8.51 (s, 1H, NC*H*Ar), 7.76 (m, 2H, Ar*H*), 7.40 (m, 2H, Ar*H*), 4.67 (d, *J* = 0.7 Hz, 1H, C=C*H₂*), 4.34 (s, 1H, C=C*H₂*), 1.01 (s, 9H, SiC*Me₃*), 0.25 (s, 6H, Si*Me₂*).
^{13}C NMR (101 MHz, Chloroform-*d*) δ 156.4, 154.9, 137.1, 134.5, 130.1, 129.0, 93.0, 25.8, 18.5, -4.7.

N-(1-((*tert*-Butyldimethylsilyl)oxy)vinyl)-1-(2-fluorophenyl)methanimine (4d)

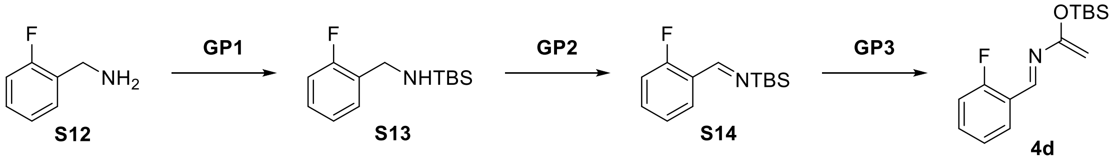

Starting from (2-fluorophenyl)methanamine (**S12**) (2.0 mL, 17 mmol, 1.0 equiv.) with triethylamine (2.8 mL, 20 mmol, 1.15 equiv.), DMAP (0.043 g, 0.35 mmol, 2 mol%) and *tert*-butyldimethylsilyl chloride (2.77 g, 18.4 mmol, 1.05 equiv.), and following **GP1**. 1-*tert*-Butyl-N-(2-fluorobenzyl)-1,1-dimethylsilanamine (**S13**) (4.40 g, 17.2 mmol) was obtained in 86% yield as a colorless oil upon kugelrohr distillation (130 °C, 1.2 mbar).

^{1}H NMR (400 MHz, Chloroform-*d*) δ 7.47 – 7.38 (m, 1H, Ar*H*), 7.20 (ddt, *J* = 9.9, 5.3, 2.6 Hz, 1H, Ar*H*), 7.12 (td, *J* = 7.5, 1.3 Hz, 1H, Ar*H*), 7.00 (ddd, *J* = 10.4, 8.1, 1.3 Hz, 1H, Ar*H*), 4.04 (d, *J* = 7.8 Hz, 2H, CH_2Ar), 0.95 (s, 1 H, TBS-N*H*), 0.93 (s, 9H, SiCMe_3), 0.06 (s, 6H, SiMe_2).
^{13}C NMR (101 MHz, Chloroform-*d*) δ 160.6 (d, *J* = 245.0 Hz), 131.4 (d, *J* = 14.7 Hz), 128.8 (d, *J* = 5.1 Hz), 127.8 (d, *J* = 8.1 Hz), 123.9 (d, *J* = 3.3 Hz), 114.9 (d, *J* = 21.6 Hz), 40.1 (d, *J* = 4.4 Hz), 26.4, 18.4, -5.0.
^{19}F NMR (376 MHz, Chloroform-*d*) δ -120.0.

The oxidation of 1-*tert*-butyl-N-(2-fluorobenzyl)-1,1-dimethylsilanamine (**S13**) (4.40 g, 17.2 mmol, 1.0 equiv.) to N-(*tert*-butyldimethylsilyl)-1-(2-fluorophenyl)methanimine (**S14**) was performed following the **GP2**, and using *tert*-butyl hypochlorite (1.25 mL, 11.0 mmol, 1.1 equiv.) in the first step, and DBU (1.7 mL, 11 mmol, 1.1 equiv.) in the second. Upon quick aqueous work-up, **S14** (2.28 g, 9.61 mmol, 96% yield) was collected as a pale yellow oil, which was not further purified.

Following **GP3**, N-(*tert*-butyldimethylsilyl)-1-(2-fluorophenyl)methanimine (**S14**) (2.28 g, 9.61 mmol, 1.0 equiv.) was reacted with triethylamine (5.5 mL, 39 mmol, 4.1 equiv.) and acetyl chloride (1.4 mL, 20 mmol, 2.1 equiv.). Upon quick aqueous work-up, the crude orange oil was submitted to column chromatography (25 g SiO_2; the column was primed with 250 mL of NEt_3 in pentane, 2.5% v/v; equilibration was then done with a mixture of pentane and the aforementioned solution 97/3; elution was done with pure pentane) to provide N-(1-((*tert*-butyldimethylsilyl)oxy)vinyl)-1-(2-fluorophenyl)methanimine (**4d**) (2.06 g, 7.37 mmol, 77% yield) as a bright yellow oil (stable at -80 °C for at least one week).

^{1}H NMR (400 MHz, Chloroform-*d*) δ 8.89 (m, 1H, NC*H*Ar), 8.11 (td, *J* = 7.5, 1.8 Hz, 1H, Ar*H*), 7.41 (dddd, *J* = 8.3, 7.3, 5.4, 1.9 Hz, 1H, Ar*H*), 7.20 (m, 1H, Ar*H*), 7.09 (ddd, *J* = 10.5, 8.2, 1.1 Hz, 1H, Ar*H*), 4.72 (d, *J* = 0.7 Hz, 1H, C=CH_2), 4.38 (d, *J* = 0.6 Hz, 1H, C=CH_2), 1.02 (s, 9H, SiCMe_3), 0.25 (s, 6H, SiMe_2).
^{13}C NMR (101 MHz, Chloroform-*d*) δ 163.0 (d, *J* = 253.9 Hz), 156.8, 149.6 (d, *J* = 5.8 Hz), 132.7 (d, *J* = 8.7 Hz), 127.8 (d, *J* = 2.5 Hz), 124.4 (d, *J* = 3.3 Hz), 123.8 (d, *J* = 9.1 Hz), 115.8 (d, *J* = 21.1 Hz), 93.5, 25.8, 18.4, -4.8.
^{19}F NMR (376 MHz, Chloroform-*d*) δ -121.0.

1-(3-Bromophenyl)-N-(1-((*tert*-butyldimethylsilyl)oxy)vinyl)methanimine (4e)

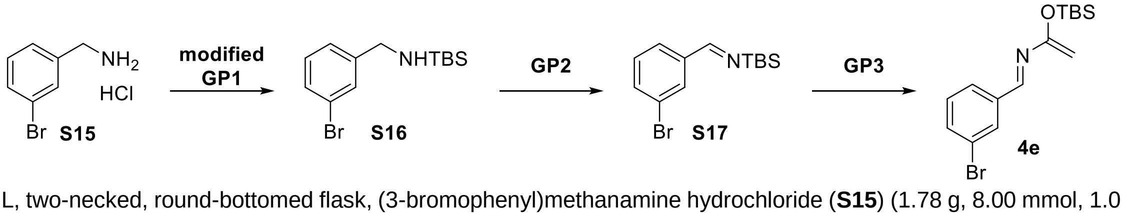

In a 50 mL, two-necked, round-bottomed flask, (3-bromophenyl)methanamine hydrochloride (**S15**) (1.78 g, 8.00 mmol, 1.0 equiv.) was suspended in Et_2O (dry; 4.0 mL). The suspension was cooled to 0 °C (ice - water bath); triethylamine (2.4 mL, 17 mmol, 2.1 equiv.) was added, and the mixture was stirred at this temperature for 10 minutes. A catalytic amount of DMAP (0.020 g, 0.16 mmol, 2 mol%) was also added. The mixture was stirred for another 3-4 minutes at 0 °C and then a solution of *tert*-butyldimethylsilyl chloride (1.27 g, 8.40 mmol, 1.05 equiv.) in Et_2O (dry; 4.0 mL) was added drop-wise over a period of about 5 minutes. The suspension was stirred over night at room temperature. After 17 hours, solids were filtered off on paper, and washed with dry diethyl ether. The filtrate was concentrated under reduced pressure to provide a pale-yellow oil, which was treated with dry pentane (10 mL) in order to induce further precipitation. The solid was again removed by filtration on paper, followed by washing with additional pentane (dry; 20 mL). The pale-yellow filtrate was concentrated under vacuum, and then submitted to kugelrohr distillation (165 °C, 1.0 mbar), N-(3-bromobenzyl)-1-(*tert*-butyl)-1,1-dimethylsilanamine (**S16**) (2.87 g, 9.91 mmol, 83% yield) as a colorless liquid.

^{1}H NMR (400 MHz, Chloroform-*d*) δ 7.48 (m, 1H, Ar*H*), 7.34 (m, 1H, Ar*H*), 7.24 (dt, *J* = 7.6, 1.3 Hz, 1H, Ar*H*), 7.17 (t, *J* = 7.7 Hz, 1H, Ar*H*), 3.95 (d, *J* = 8.0 Hz, 2H, CH_2Ar), 0.92 (s, 9H, SiCMe_3), 0.70 (br s, 1H, TBS-N*H*), 0.05 (s, 6H, SiMe_2).
^{13}C NMR (101 MHz, Chloroform-*d*) δ 147.1, 129.9, 129.8, 129.4, 125.4, 122.5, 46.1, 26.4, 18.4, -5.0.

The oxidation of N-(3-bromobenzyl)-1-(*tert*-butyl)-1,1-dimethylsilanamine (**S16**) (1.89 g, 6.29 mmol, 1.0 equiv.) to 1-(3-bromophenyl)-N-(*tert*-butyldimethylsilyl)methanimine (**S17**) was performed following the **GP2**, and using *tert*-butyl hypochlorite (0.75 mL, 6.6 mmol, 1.05 equiv.) in the first step, and DBU (1.0 mL, 6.7 mmol, 1.04 equiv.) in the second. Upon quick aqueous work-up, **S17** (1.7 g, 1.5 mmol, 91% yield) was collected as a golden oil, which was not further purified.

Following **GP3**, 1-(3-bromophenyl)-N-(*tert*-butyldimethylsilyl)methanimine (**S17**) (2.28 g, 9.61 mmol, 1.0 equiv.) was reacted with triethylamine (5.5 mL, 39 mmol, 4.1 equiv.) and acetyl chloride (1.4 mL, 20 mmol, 2.1 equiv.). Upon quick aqueous work-up, the crude orange oil was submitted to column chromatography (25 g SiO_2; the column was primed with 250 mL of NEt_3 in pentane, 2.5% v/v; equilibration was then done with a mixture of pentane and the aforementioned solution 97/3; elution was done with pure pentane) to provide N-(1-((*tert*-butyldimethylsilyl)oxy)vinyl)-1-(2-fluorophenyl)methanimine (**4e**) (2.06 g, 7.37 mmol, 77% yield) as a bright yellow oil (stable at -80 °C for 3-4 days).

^{1}H NMR (400 MHz, Chloroform-*d*) δ 8.47 (s, 1H, N*CH*Ar), 8.00 (t, *J* = 1.8 Hz, 1H, Ar*H*), 7.72 (dt, *J* = 7.7, 1.4 Hz, 1H, Ar*H*), 7.55 (ddd, *J* = 7.9, 2.0, 1.0 Hz, 1H, Ar*H*), 7.30 (t, *J* = 7.8 Hz, 1H, Ar*H*), 4.70 (d, *J* = 0.7 Hz, 1H, C=CH_2), 4.37 (s, 1H, C=CH_2), 1.01 (s, 9H, SiCMe_3), 0.25 (s, 6H, SiMe_2).
^{13}C NMR (101 MHz, Chloroform-*d*) δ 156.3, 154.6, 138.0, 133.9, 131.4, 130.2, 127.7, 123.0, 93.6, 25.8, 18.4, -4.8.

N-(1-((*tert*-Butyldimethylsilyl)oxy)vinyl)-1-(4-methoxyphenyl)methanimine (4f)

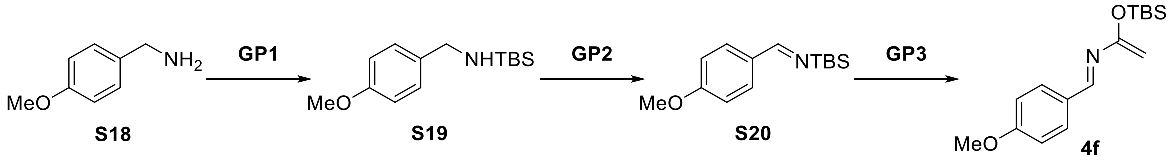

Starting from (4-methoxyphenyl)methanamine (**S18**) (2.0 mL, 15 mmol, 1.0 equiv.) with triethylamine (2.5 mL, 18 mmol, 1.17 equiv.), DMAP (0.037 g, 0.31 mmol, 2 mol%) and *tert*-butyldimethylsilyl chloride (1.81 g, 17.9 mmol, 1.05 equiv.), and following **GP1**. 1-*tert*-Butyl-N-(4-methoxybenzyl)-1,1-dimethylsilanamine (**S19**) (2.51 g, 10.0 mmol) was obtained in 65% yield as a colorless oil upon kugelrohr distillation (150 °C, 1.3 mbar).

^{1}H NMR (400 MHz, Chloroform-*d*) δ 7.32 – 7.19 (m, 2H, Ar*H*), 6.90 (d, *J* = 8.7 Hz, 2H, Ar*H*), 3.95 (d, *J* = 7.7 Hz, 2H, CH_2Ar), 3.84 (s, 3H, O*Me*), 0.96 (s, 9H, SiCMe_3), 0.67 (br s, 1H, TBS-N*H*), 0.08 (s, 6H, SiMe_2).
^{13}C NMR (101 MHz, Chloroform-*d*) δ 158.3, 136.8, 128.0, 113.7, 55.3, 45.9, 26.5, 18.5, -4.9.

The oxidation of 1-*tert*-butyl-N-(4-methoxybenzyl)-1,1-dimethylsilanamine (**S19**) (2.52 g, 10.0 mmol, 1.0 equiv.) to N-(*tert*-butyldimethylsilyl)-1-(4-methoxyphenyl)methanimine (**S20**) was performed following the **GP2**, and using *tert*-butyl hypochlorite (1.25 mL, 11.0 mmol, 1.1 equiv.) in the first step, and DBU (1.7 mL, 11 mmol, 1.1 equiv.) in the second. Upon quick aqueous work-up, **S20** (2.07 g, 8.29 mmol, 83% yield) was collected as a yellow oil, which was not further purified.

Following **GP3**, N-(*tert*-butyldimethylsilyl)-1-(4-methoxyphenyl)methanimine (**S20**) (3.03 g, 11.9 mmol, 1.0 equiv.) was reacted with triethylamine (4.7 mL, 34 mmol, 4.1 equiv.) and acetyl chloride (1.2 mL, 17 mmol, 2.1 equiv.). Upon quick aqueous work-up, the crude orange oil was submitted to column chromatography (25 g SiO_2; the column was primed with 250 mL of NEt_3 in pentane, 2.5% v/v; equilibration was then done with a mixture of pentane and the aforementioned solution 97/3; elution was done with pure pentane) to provide N-(1-((*tert*-butyldimethylsilyl)oxy)vinyl)-1-(4-chlorophenyl)methanimine (**4f**) (1.07 g, 3.67 mmol, 44% yield) as a pale yellow oil, which became an off-white solid on standing at -80 °C (stable at this temperature for at least 4 months).

^{1}H NMR (400 MHz, Chloroform-*d*) δ 8.51 (s, 1H, N*CH*Ar), 7.77 (m, 2H, Ar*H*), 6.94 (m, 2H, Ar*H*), 4.58 (s, 1H, C=CH_2), 4.25 (s, 1H, C=CH_2), 3.85 (s, 3H, O*Me*), 1.01 (s, 9H, SiCMe_3), 0.24 (s, 6H, SiMe_2).
^{13}C NMR (101 MHz, Chloroform-*d*) δ 162.2, 157.0, 155.9, 130.7, 128.9, 114.1, 90.8, 55.4, 25.9, 18.5, -4.7.

N-(1-((*tert*-Butyldimethylsilyl)oxy)vinyl)-2-methyl-3-phenylprop-2-en-1-imine (4g)

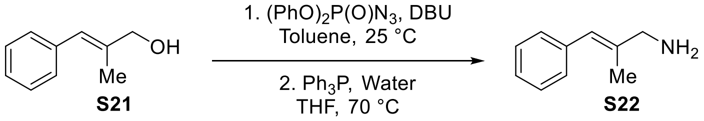

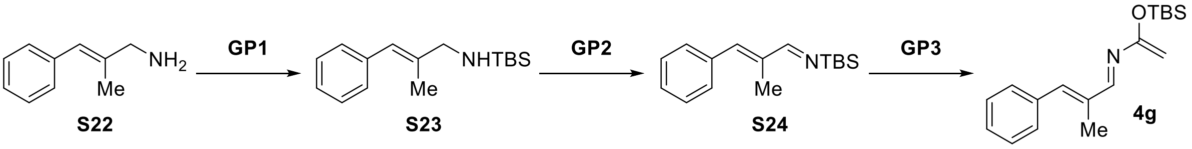

Following an adapted version of a reported procedure,[7] in a 250 mL, two-necked, round-bottomed flask, (*E*)-2-methyl-3-phenylprop-2-en-1-ol (**S21**) (3.0 g, 20 mmol, 1.0 equiv.) was dissolved in toluene (dry; 60 mL). At room temperature, diphenyl phosphoryl azide (6.5 mL, 30 mmol, 1.5 equiv.) was added leading to the formation of a clear, colorless solution. Finally, DBU (4.5 mL, 30 mmol, 1.5 equiv.) was also added (slowly) under stirring: the mixture became turbid, then gradually turned to yellow and, finally, to brown. It was stirred at room temperature for 5 hours. The reaction was then quenched by addition of sat. aq. NH_4Cl (60 mL), which made the mixture become orange. Upon separation of the layers, the aqueous one was extracted with Et_2O (3 x 50 mL). The combined organic layers were washed with brine, dried over $MgSO_4$, filtered, and concentrated under vacuum. The resulting orange crude oil was submitted to column chromatography (Biotage flash chromatographer, 80 g SiO_2; EtOAc in pentane, 0 to 5%) to provide (*E*)-(3-azido-2-methylprop-1-en-1-yl)benzene (2.79 g, 16.1 mmol, 79% yield) as a colorless oil.

^{1}H NMR (400 MHz, Chloroform-*d*) δ 7.36 (t, *J* = 7.6 Hz, 2H, Ar*H*), 7.32 – 7.22 (m, 3H, Ar*H*), 6.53 (s, 1H, Ar*CH*=C), 3.87 (s, 2H, *CH₂*N₃), 1.95 (d, *J* = 1.5 Hz, 3H, C=C*Me*).
^{1}H-NMR data corresponded to the reported values.[8]

Following an adapted version of a reported procedure,[7] inside an open 250 mL, single-necked, round-bottomed flask, equipped with a Liebig condenser, [(*E*)-3-azido-2-methylprop-1-enyl]benzene (2.79 g, 16.1 mmol, 1.0 equiv.) and triphenylphosphine (7.38 g, 28.2 mmol, 1.75 equiv.) were dissolved in THF (160 mL). Upon the addition of water (1.5 mL, 83 mmol, 5.2 equiv.), the pale yellow mixture was refluxed (heating bath at 70 °C) for 5 hours. After this time, TLC analysis (DCM/MeOH 95/5) showed the complete consumption of the starting azide. The mixture was allowed to cool down to room temperature, and subsequently concentrated under reduced pressure. The resulting viscous, pale yellow oil was treated with Et_2O (100 mL), which led to the massive precipitation of a white solid. The latter was filtered off through a plug of celite, which was then washed with additional diethyl ether. The filtrate was extracted with aq. HCl (2.0 N; 120 mL). The aqueous layer was washed with Et_2O (3 x 80 mL), cooled to 0 °C (ice-water bath), and then basified by addition of solid NaOH under stirring until pH > 10. Finally, it was extracted with Et_2O (3 x 80 mL). The combined organic layers were dried over Na_2SO_4, filtered, and concentrated under reduced pressure. The resulting yellow, crude oil was submitted to column chromatography (SiO_2; DCM/Ultra 11/1 to 7/1 to 5/1 to 2/1) to furnish (*E*)-2-methyl-3-phenyl-prop-2-en-1-amine (**S22**) (1.46 g, 9.95 mmol, 62% yield) as a yellow oil.

^{1}H NMR (400 MHz, Chloroform-*d*) δ 7.36 – 7.30 (m, 2H, Ar*H*), 7.26 (dt, *J* = 6.0, 1.7 Hz, 2H, Ar*H*), 7.23 – 7.17 (m, 1H, Ar*H*), 6.43 (m, 1H), 3.37 (d, *J* = 1.7 Hz, 2H, CH_2NH₂), 1.89 (d, *J* = 1.5 Hz, 3H, C=C*Me*), 1.44 (br s, 2H, NH_2).
^{1}H-NMR data corresponded to the reported values.[9]

Starting from (E)-2-methyl-3-phenyl-prop-2-en-1-amine (**S22**) (1.46 g, 9.95 mmol, 1.0 equiv.) with triethylamine (1.6 mL, 11 mmol, 1.15 equiv.), DMAP (0.024 g, 0.20 mmol, 2 mol%) and *tert*-butyldimethylsilyl chloride (1.57 g, 10.4 mmol, 1.05 equiv.), and following **GP1**. (*E*)-1-*tert*-Butyl-1,1-dimethyl-N-(2-methyl-3-phenylallyl)silanamine (**S23**) (1.28 g, 4.91 mmol) was obtained in 49% yield as a yellow oil upon kugelrohr distillation (140-150 °C, 0.73 mbar).

^{1}H NMR (400 MHz, Methylene Chloride-d_2) δ 7.32 (m, 2H, Ar*H*), 7.27 (m, 2H, Ar*H*), 7.19 (m, 1H, Ar*H*), 6.48 (m, 1H, Ar*CH*=C), 3.44 (d, *J* = 6.7 Hz, 2H, CH_2NH-TBS), 1.85 (d, *J* = 1.5 Hz, 3H, C=C*Me*), 0.93 (s, 9H, SiCMe_3), 0.62 (br s, 1H, TBS-N*H*), 0.06 (s, 6H, SiMe_2).
^{13}C NMR (101 MHz, Methylene Chloride-d_2) δ 141.1, 138.6, 128.7, 128.0, 125.8, 122.9, 50.7, 26.2, 18.3, 16.0, -5.3.

The oxidation of (*E*)-1-*tert*-butyl-1,1-dimethyl-N-(2-methyl-3-phenylallyl)silanamine (**S23**) (1.28 g, 4.89 mmol, 1.0 equiv.) to N-(*tert*-butyldimethylsilyl)-2-methyl-3-phenylprop-2-en-1-imine (**S24**) was performed following the **GP2**, and using *tert*-butyl hypochlorite (0.72 mL, 6.4 mmol, 1.3 equiv.) in the first step, and DBU (0.77 mL, 5.1 mmol, 1.05 equiv.) in the second. Upon quick aqueous work-up, **S24** (1.04 g, 4.02 mmol, 82% yield) was collected as a gold yellow oil, which was not further purified.

Following **GP3**, N-(*tert*-butyldimethylsilyl)-2-methyl-3-phenylprop-2-en-1-imine (**S24**) (1.04 g, 4.01 mmol, 1.0 equiv.) was reacted with triethylamine (2.3 mL, 17 mmol, 4.1 equiv.) and acetyl chloride (0.60 mL, 8.4 mmol, 2.1 equiv.). Upon quick aqueous work-up, the crude orange oil was submitted to column chromatography (25 g SiO_2; the column was primed with 250 mL of NEt_3 in pentane, 2.5% v/v; equilibration was then done with a mixture of pentane and the aforementioned solution 97/3; elution was done

with pure pentane) to provide N-(1-((*tert*-butyldimethylsilyl)oxy)vinyl)-2-methyl-3-phenylprop-2-en-1-imine (**4g**) (0.610 g, 2.02 mmol, 50% yield) as a pale yellow oil (stable at -80 °C for at least 4 months).

^{1}H NMR (400 MHz, Chloroform-*d*) δ 8.31 (d, J = 0.6 Hz, 1H, N*CH*Ar), 7.49 – 7.43 (m, 2H, Ar*H*), 7.40 (ddd, J = 7.9, 6.8, 1.2 Hz, 2H, Ar*H*), 7.31 (m, 1H, Ar*H*), 6.92 (s, 1H, Ar*CH*=C), 4.57 (s, 1H, C=*CH$_2$*), 4.25 (s, 1H, C=*CH$_2$*), 2.23 (d, *J* = 1.3 Hz, 3H, C=C*Me*), 1.02 (s, 9H, SiC*Me$_3$*), 0.25 (s, 6H, Si*Me$_2$*).
^{13}C NMR (101 MHz, Chloroform-d) δ 161.6, 157.1, 141.6, 137.2, 136.7, 129.6, 128.4, 128.0, 91.2, 25.9, 18.5, 13.1, -4.7.

2. Preparation of the Cyclopropropanes (Compounds 2)

General procedures for the synthesis of dibenzyl cyclopropane-1,1-dicarboxylates

GP4: Thermal Knoevenagel condensation of dibenzyl malonate with aldehydes

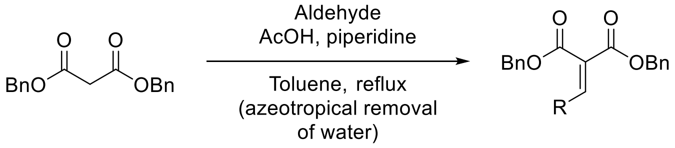

In a 25 mL, round-bottomed, one-necked flask equipped with a Dean-Stark apparatus, the aldehyde (1.0 equiv.) and dibenzyl malonate (1.0 equiv.) were dissolved in toluene. Acetic acid (6 drops per mmol of the aldehyde) and piperidine (4 drops per mmol of the aldehyde) were added. The resulting colorless, clear solution was stirred at reflux with azeotropical removal of water for 4-18 hours, darkening to yellow and then to orange. The mixture was allowed to cool down to room temperature and then directly submitted to column chromatography.

GP5: Corey-Chaykovsky cyclopropanation

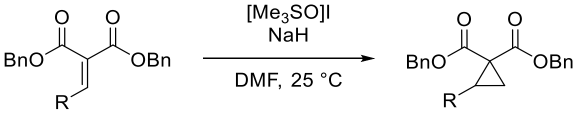

Following a reported procedure,[10] NaH (60% dispersion in mineral oil; 1.1-1.25 equiv.) was suspended in DMF. Trimethylsulfoxonium iodide (1.05-1.2 equiv.) was then added in one single portion. Immediately, gas release was observed. The mixture was stirred at room temperature for 30-45 minutes, progressively becoming a clear, pale-yellow solution. The Knoevenagel condensation product (1.0 equiv.) was then added (neat or as a solution in DMF). The resulting mixture was stirred at room temperature for 1-3 hours. The reaction was quenched by addition of water (20 mL). The aqueous solution was extracted with EtOAc (5 x 20 mL), and the combined organic layers were washed with brine (3 x 20 mL), dried over $MgSO_4$, filtered, and concentrated under vacuum. The resulting crude oil was purified by column chromatography.

Dibenzyl 2-(4-methoxyphenyl)cyclopropane-1,1-dicarboxylate (2a)

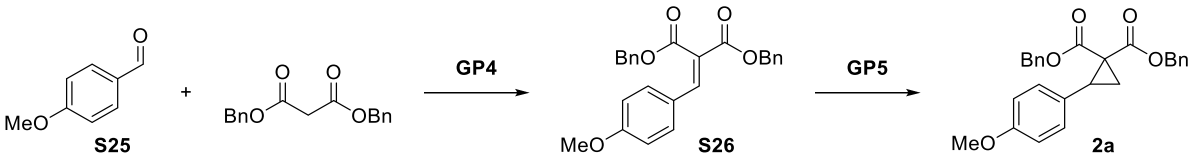

Following the **GP4**, *p*-anisaldehyde (**S25**) (1.5 mL, 12 mmol, 1.0 equiv.) and dibenzyl malonate (3.0 mL, 12 mmol, 1.0 equiv.) were reacted with acetic acid (24 drops) and piperidine (16 drops) in toluene (4.0 mL) at reflux and with azeotropical removal of water for 5.5 hours. Upon cooling to room temperature, the mixture was submitted to column chromatography (Biotage flash chromatographer, 25 g SiO_2; 80 g SiO_2; EtOAc in pentane, 0 to 25%) to afford dibenzyl 2-(4-methoxybenzylidene)malonate (**S26**) (3.26 g, 8.01 mmol, 68% yield) as a pale yellow, crystalline solid.

Following the **GP5**, dibenzyl 2-(4-methoxybenzylidene)malonate (**S26**) (2.80 g, 6.96 mmol, 1.0 equiv.) was added in a single portion to a mixture of trimethylsulfoxonium iodide (1.38 g, 7.65 mmol, 1.2 equiv.) and NaH (60% dispersion in mineral oil; 0.320 g, 8.00 mmol, 1.25 equiv.) in DMF (27 mL). After 2 hours, aqueous work-up followed by column chromatography (Biotage flash chromatographer, 25 g SiO_2; EtOAc in pentane, 13%) afforded dibenzyl 2-(4-methoxyphenyl)cyclopropane-1,1-dicarboxylate (**2a**) (1.25 g, 3.00 mmol, 43% yield) as a white solid.

^{1}H NMR (400 MHz, Chloroform-*d*) δ 7.37 – 7.28 (m, 5H, Ar*H*), 7.26 – 7.16 (m, 3H, Ar*H*), 7.12 – 7.07 (m, 2H, Ar*H*), 7.02 – 6.96 (m, 2H, Ar*H*), 6.77 – 6.67 (m, 2H, Ar*H*), 5.25 (d, *J* = 12.4 Hz, 1H, O*CH$_2$*Ph), 5.15 (d, *J* = 12.4 Hz, 1H, O*CH$_2$*Ph), 4.81 (d, *J* = 12.3 Hz, 1H, O*CH$_2$*Ph), 4.77 (d, *J* = 12.2 Hz, 1H, O*CH$_2$*Ph), 3.78 (s, 3H, O*Me*), 3.23 (t, *J* = 8.7 Hz, 1H, Ar*CH*CH$_2$), 2.18 (dd, *J* = 8.1, 5.1 Hz, 1H, ArCH*CH$_2$*), 1.74 (dd, *J* = 9.3, 5.1 Hz, 1H, ArCH*CH$_2$*).
^{1}H-NMR data corresponded to the reported values.[11]

Dimethyl 2-(4-methoxyphenyl)cyclopropane-1,1-dicarboxylate (2a')

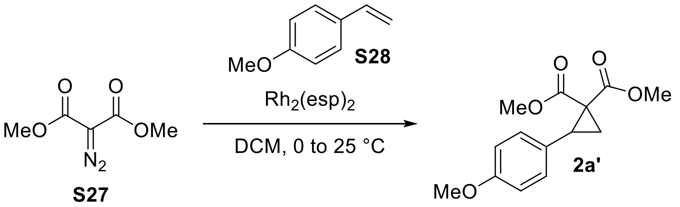

Following a reported procedure,[12] inside a glove-box, a 25 mL, round-bottomed vial was charged with $Rh_2(esp)_2$ (0.014 g, 0.019 mmol, 0.1 mol%). The vial was capped with a PTFE septum and taken out of the glove-box. A solution of 1-methoxy-4-vinylbenzene (**S28**) (freshly filtered over a pad of aluminum oxide; 0.30 mL, 2.3 mmol, 1.2 equiv.) in DCM (1.6 mL) was then added and the reaction mixture was stirred at 0 °C. After 5 minutes, a solution of dimethyl diazomalonate (**S27**) (0.300 g, 1.90 mmol, 1.0 equiv.) in DCM (1.6 mL) was added. The resulting green, clear solution was stirred at 0 °C for 10 minutes and then overnight at room temperature. The reaction mixture was then concentrated under reduced pressure and purified directly by column chromatography (Biotage flash chromatographer, 24 g SiO_2; EtOAc in pentane, 2 to 20%) to afford dimethyl 2-(4-methoxyphenyl)cyclopropane-1,1-dicarboxylate (**2a'**) (0.414 g, 1.57 mmol, 83% yield) as a viscous, colorless oil.

^{1}H NMR (400 MHz, Chloroform-*d*) δ 7.15 - 7.06 (m, 2H), 6.80 (d, *J* = 8.8 Hz, 2H), 3.78 (s, 3H), 3.77 (s, 3H), 3.38 (s, 3H), 3.18 (t, *J* = 8.6 Hz, 1H), 2.15 (dd, *J* = 8.0, 5.1 Hz, 1H), 1.72 (dd, *J* = 9.2, 5.2 Hz, 1H).
^{1}H-NMR data corresponded to the reported values.[12]

Di*iso*propyl 2-(4-methoxyphenyl)cyclopropane-1,1-dicarboxylate (2a")

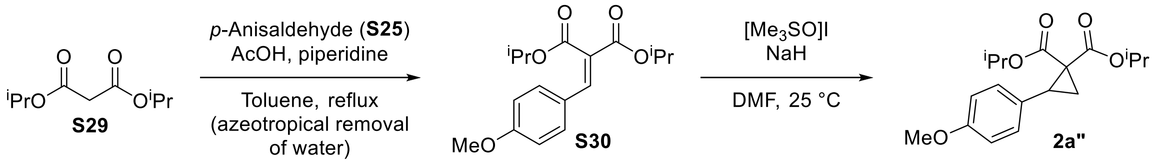

In a 50 mL, two-necked, round-bottomed flask, equipped with a Dean-Stark apparatus, *p*-anisaldehyde (1.2 mL, 10 mmol, 1.0 equiv.) and di(*iso*propyl) malonate (**S29**) (2.5 mL, 13 mmol, 1.3 equiv.) were dissolved in toluene (dry; 20 mL). Piperidine (0.20 mL, 20 mmol, 0.2 equiv.) and AcOH (0.12 mL, 2.0 mmol, 0.20 equiv.) were then added. The resulting clear, colorless solution was stirred at 130-140 °C overnight, with azeotropical removal of water. After 15 h, the mixture looked like an orange, clear solution. The latter was concentrated under vacuum to provide a red-brown crude oil, which was submitted to column chromatography (Biotage flash chromatographer, 40 SiO_2; EtOAc in pentane, 5 to 10%). Di(*iso*propyl)-2-(4-methoxybenzylidene)malonate (**S30**) (ca. 90% pure because of the presence of residual unreacted *p*-anisaldehyde; 1.41 g, 4.16 mmol, 42% yield) was collected as a pale yellow oil.

^{1}H NMR (400 MHz, Chloroform-*d*) δ 7.61 (s, 1H, C=C*H*), 7.48 – 7.41 (m, 2H, Ar*H*), 6.92 – 6.83 (m, 2H, Ar*H*), 5.27 (hept, *J* = 6.3 Hz, 1H, OC*H*Me$_2$), 5.14 (hept, *J* = 6.3 Hz, 1H, OC*H*Me$_2$), 3.83 (s, 3H, O*Me*), 1.31 (dd, *J* = 6.2, 5.5 Hz, 12H, OCH*Me*$_2$).

Following a slightly modified version of a reported procedure,[13] in a 25 mL, two-necked, round-bottomed flask, trimethylsulfoxonium iodide (1.10 g, 4.99 mmol, 1.08 equiv.) was dissolved in DMF (dry; 18.0 mL). NaH (60% dispersion in mineral oil; 0.203 g, 5.08 mmol, 1.10 equiv.) was then added in one single portion. Immediately, gas release was observed. The mixture was stirred at room temperature for 45 minutes, progressively becoming a less turbid, pale yellow solution. A solution of dibenzyl 2-(4-di*iso*propyl)malonate (**S30**) (1.41 g, 4.62 mmol, 1.0 equiv.) in DMF (7.0 mL) was then added. The resulting clear, yellow solution was stirred at room temperature for 3 hours. After this time, TLC analysis showed full conversion. The reaction was quenched by addition of water (20 mL) and brine (20 mL). The aqueous solution was extracted with Et_2O (4 x 20 mL), and the combined organic layers were washed with brine (2 x 20 mL), dried over $MgSO_4$, filtered, and concentrated under vacuum. The resulting crude oil was submitted to column chromatography (Biotage 40 g SiO_2; EtOAc in pentane, 2 to 20%) to furnish 2-(4-methoxyphenyl)cyclopropane-1,1-dicarboxylic acid bis(*iso*propyl) ester (**3a"**) (0.826 g, 2.58 mmol, 56% yield) as a pale yellow oil.

R_f (pentane/EtOAc 7/3) 0.80.
^{1}H NMR (400 MHz, Chloroform-*d*) δ 7.17 – 7.08 (m, 2H, Ar*H*), 6.83 – 6.76 (m, 2H, Ar*H*), 5.08 (hept, *J* = 6.3 Hz, 1H, OC*H*Me$_2$), 4.73 (hept, *J* = 6.2 Hz, 1H, OC*H*Me$_2$), 3.76 (s, 3H, O*Me*), 3.13 (t, *J* = 8.6 Hz, 1H, ArC*H*CH$_2$), 2.07 (dd, J = 7.9, 5.1 Hz, 1H, ArCHC*H$_2$*), 1.61 (dd, *J* = 9.2, 5.1 Hz, 1H, ArCHC*H$_2$*), 1.28 (d, *J* = 6.2 Hz, 3H, OCH*Me*$_2$), 1.26 (d, *J* = 6.2 Hz, 3H, OCH*Me*$_2$), 1.07 (d, *J* = 6.2 Hz, 3H, OCH*Me*$_2$), 0.75 (d, *J* = 6.3 Hz, 3H, OCH*Me*$_2$).
^{13}C NMR (101 MHz, Chloroform-*d*) δ 169.6, 166.4, 158.9, 129.8, 126.7, 113.5, 69.1, 68.5, 55.3, 37.7, 31.3, 21.8, 21.7, 21.4, 21.3, 18.5.
HRMS (ESI/QTOF) m/z: [M + Na]$^+$ Calcd for $C_{18}H_{24}NaO_5^+$ 343.1516; Found 343.1518.

Dineopentyl 2-(4-methoxyphenyl)cyclopropane-1,1-dicarboxylate (2a''')

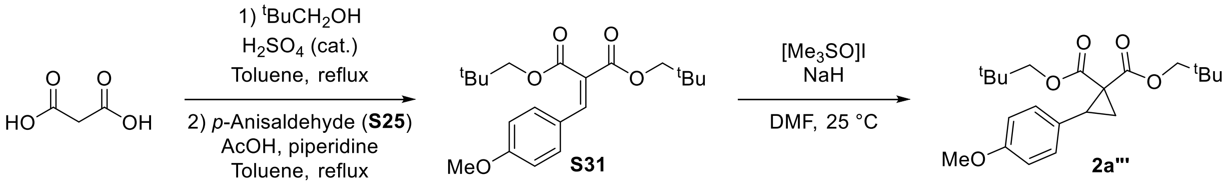

A 100 mL, two-necked, round-bottomed flask, equipped with a Dean-Stark apparatus, was charged with malonic acid (1.50 g, 14.4 mmol, 1.0 equiv.) and neopentyl alcohol (2.92 g, 33.1 mmol, 2.3 equiv.). Toluene (41 mL) was added, followed by sulfuric acid (0.20 mL). The resulting colorless, clear solution was heated to reflux while removing water azeotropically for 2 hours. The mixture became darker over this time, from colorless to yellow, then orange, and finally violet. It was then allowed to cool down to room temperature, diluted with EtOAc (40 mL), washed with water (3 x 40 mL), brine, dried over $MgSO_4$, filtered, and concentrated under reduced pressure. The resulting dark crude oil was submitted to column chromatography (Biotage flash chromatographer, 40 g SiO_2; EtOAc in pentane 2 to 15%) to afford dineopentyl malonate (2.08 g, 8.50 mmol, 59% yield) as a yellow oil.

^{1}H NMR (400 MHz, Chloroform-*d*) δ 3.87 (s, 4H, O*CH₂*), 3.44 (s, 2H, (CO)*CH₂*(CO)), 0.96 (s, 18H, *ᵗBu*).

Following a reported procedure,[14] in a sealed 25 mL, round-bottomed test tube, *p*-anisaldehyde (**S25**) (0.80 mL, 6.6 mmol, 1.0 equiv.) and dineopentyl malonate (1.77 g, 7.25 mmol, 1.1 equiv.) were added by syringe, followed by piperidine (0.20 mL, 2.0 mmol, 0.3 equiv.) and AcOH (10 drops). The resulting pale-yellow suspension was stirred at 110 °C (reflux) for 14 hours. After this time, the mixture was cooled to room temperature, diluted with EtOAc (50 mL) and washed with aq. HCl (1.0 M; 2 × 50 mL). The organic layer was then washed with brine, dried over $MgSO_4$, filtered. and concentrated *in vacuo*. Purification by flash column chromatography of the resulting yellow crude oil (Biotage flash chromatographer, 40 g SiO_2; EtOAc in pentane, 2 to 20%) afforded dineopentyl-2-(4-methoxybenzylidene)malonate (**S31**) (0.95 g, 2.6 mmol, 40% yield) as a pale yellow oil.

^{1}H NMR (400 MHz, Chloroform-*d*) δ 7.68 (s, 1H, C=*CH*), 7.45 – 7.38 (m, 2H, Ar*H*), 6.92 – 6.83 (m, 2H, Ar*H*), 3.95 (s, 2H, O*CH₂*), 3.93 (s, 2H, O*CH₂*), 3.83 (s, 3H, O*Me*), 0.96 (s, 9H, *ᵗBu*), 0.92 (s, 9H, *ᵗBu*).

Following a slightly modified version of a reported procedure,[10] in a 25 mL, two-necked, round-bottomed flask, trimethylsulfoxonium iodide (0.68 g, 3.1 mmol, 1.25 equiv.) was dissolved in DMF (9.0 mL). NaH (60% dispersion in mineral oil; 0.141 g, 3.23 mmol, 1.30 equiv.) was then added in one single portion. Immediately, gas release was observed. The mixture was stirred at room temperature for 45 minutes, progressively becoming a clear, pale-yellow solution. A solution of dineopently 2-(4-methoxybenzylidene)malonate (**S31**) (0.90 g, 2.5 mmol, 1.0 equiv.) in DMF (2.0 mL) was then added. The resulting clear, yellow solution was stirred at room temperature overnight. After 16 hours, TLC analysis showed full conversion. The reaction was quenched by addition of water (20 mL). The aqueous solution was extracted with EtOAc (4 x 20 mL), and the combined organic layers were washed with brine (2 x 20 mL), dried over $MgSO_4$, filtered, and concentrated under vacuum. The resulting crude oil was submitted to column chromatography (Biotage flash chromatographer, 25 g SiO_2; EtOAc in pentane, 2 to 20%) to furnish dineopentyl 2-(4-methoxyphenyl)cyclopropane-1,1-dicarboxylate (**2a'''**) (0.50 g, 1.3 mmol, 53% yield) as a white solid.

^{1}H NMR (400 MHz, Chloroform-*d*) δ 7.16 – 7.08 (m, 2H, Ar*H*), 6.83 – 6.75 (m, 2H, Ar*H*), 3.89 (d, *J* = 10.5 Hz, 1H), 3.86 (d, *J* = 10.5 Hz, 1H, O*CH₂*), 3.76 (s, 3H, O*Me*), 3.53 (d, *J* = 10.4 Hz, 1H, O*CH₂*), 3.43 (d, *J* = 10.4 Hz, 1H, O*CH₂*), 3.22 – 3.13 (m, 1H, Ar*CH*CH₂), 2.12 (dd, *J* = 8.0, 5.1 Hz, 1H, ArCH*CH₂*), 1.70 (dd, *J* = 9.3, 5.1 Hz, 1H, ArCH*CH₂*), 0.95 (s, 9H, *ᵗBu*), 0.71 (s, 9H, *ᵗBu*). ^{1}H-NMR data corresponded to the reported values.[10]

Dibenzyl 2-(3,4-dimethoxyphenyl)cyclopropane-1,1-dicarboxylate (2b)

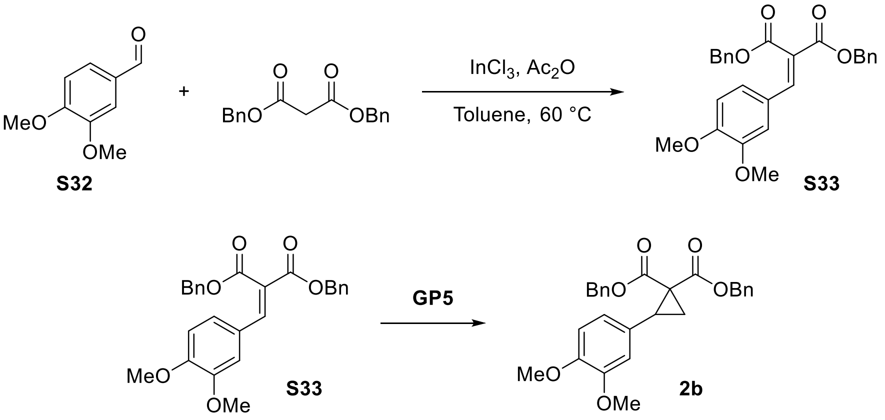

Following a reported procedure,[15] inside a glovebox, a 25 mL, round-bottomed vial was charged with Indium trichloride (44 mg, 0.20 mmol, 10 mol%). The vial was capped with a PTFE septum and taken out of the glovebox. Toluene (2.0 mL) was added by syringe, followed by 3,4-dimethoxybenzaldehyde (**S32**) (0.33 g, 2.0 mmol, 1.0 equiv.) and dibenzyl malonate (0.55 mL, 2.2 mmol,

1.1 equiv.). Finally, acetic anhydride (0.26 mL, 2.0 mmol,) was also added. The resulting clear solution was heated to 60 °C under stirring. The mixture was allowed to cool down to room temperature, diluted with EtOAc (20 mL) and washed with sat. aq. $NaHCO_3$ (20 mL). The aqueous layer was extracted once with EtOAc (20 mL). The combined organic layers were washed with brine, dried over Na_2SO_4, filtered, and concentrated under vacuum. The resulting crude oil was purified by column chromatography (Biotage flash chromatographer, 25 g SiO_2; EtOAc in pentane, 2 to 20%), to provide dibenzyl 2-(3,4-dimethoxybenzylidene)malonate (**S33**) (0.567 g 1.31 mmol, 66% yield) as a white solid.

Following the **GP5**, a solution of dibenzyl 2-(3,4-dimethoxybenzylidene)malonate (**S33**) (0.56 g, 1.3 mmol, 1.0 equiv.) in DMF (3.0 mL) was added to a mixture of trimethylsulfoxonium iodide (0.34 g, 1.6 mmol, 1.2 equiv.) and NaH (60% dispersion in mineral oil; 0.065 g, 1.6 mmol, 1.2 equiv.) in DMF (9.0 mL). After 1.5 hours, aqueous work-up followed by column chromatography (Biotage flash chromatographer, 25 g SiO_2; EtOAc in pentane, 7 to 15%) afforded an off-white solid. Recrystallization from hexane (1.5 mL) and EtOAc (0.5 mL) provided dibenzyl 2-(3,4-dimethoxyphenyl)cyclopropane-1,1-dicarboxylate (**2b**) (0.21 g, 0.47 mmol, 36% yield) as a crystalline, white solid.

Melting point: 86.5 – 87.0 °C.
R_f (pentane/EtOAc 85/15) = 0.20.
^{1}H NMR (400 MHz, Chloroform-*d*) δ 7.38 – 7.29 (m, 5H, Ar*H*), 7.26 (m, 1H, Ar*H*), 7.23 (m, 1H, ArH), 7.20 (m, 1H, Ar*H*), 6.94 (d, *J* = 7.2 Hz, 2H, Ar*H*), 6.72 – 6.68 (m, 3H, Ar*H*), 5.27 (d, *J* = 12.4 Hz, 1H, OCH_2Ph), 5.16 (d, *J* = 12.4 Hz, 1H, OCH_2Ph), 4.83 (d, *J* = 12.2 Hz, 1H, OCH_2Ph), 4.77 (d, *J* = 12.2 Hz, 1H, OCH_2Ph), 3.85 (s, 3H, O*Me*), 3.73 (s, 3H, O*Me*), 3.23 (t, *J* = 8.6 Hz, 1H, Ar*CH*CH_2), 2.24 – 2.15 (m, 1H, ArCHCH_2), 1.75 (m, 1H, ArCHCH_2).
^{13}C NMR (101 MHz, Chloroform-*d*; the signal corresponding to one aromatic C is not resolved) δ 169.7, 166.6, 148.6, 148.4, 135.5, 135.2, 128.6, 128.3, 128.1, 128.1, 128.0, 126.7, 120.4, 112.1, 110.6, 67.4, 67.2, 55.8, 55.8, 37.4, 32.7, 19.4.
HRMS (ESI/QTOF) m/z: $[M + Na]^+$ Calcd for $C_{27}H_{26}NaO_6^+$ 469.1622; Found 469.1626.

Dibenzyl 2-(2,4-dimethoxyphenyl)cyclopropane-1,1-dicarboxylate (2c)

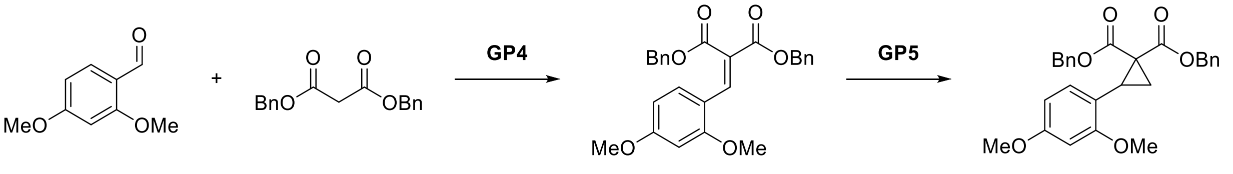

S34 **S35** **2c**

Following the **GP4**, 2,4-dimethoxybenzaldehyde (**S34**) (0.500 g, 3.01 mmol, 1.0 equiv.) and dibenzyl malonate (0.75 mL, 3.0 mmol, 1.0 equiv.) were reacted with acetic acid (6 drops) and piperidine (4 drops) in toluene (1.0 mL) at reflux and with azeotropical removal of water for 6 hours. Upon cooling to room temperature, the mixture was submitted to column chromatography (Biotage flash chromatographer, 25 g SiO_2; EtOAc in pentane, 5 to 32%) to afford dibenzyl 2-(2,4-dimethoxybenzylidene)malonate (**S35**) (1.01 g, 2.33 mmol, 78% yield) as a viscous, pale yellow oil.

Following the **GP5**, a solution of dibenzyl 2-(2,4-dimethoxybenzylidene)malonate (**S35**) (1.01 g, 2.33 mmol, 1.0 equiv.) in DMF (6.0 mL) was added to a mixture of trimethylsulfoxonium iodide (0.617 g, 2.80 mmol, 1.2 equiv.) and NaH (60% dispersion in mineral oil; 0.117 g, 2.92 mmol, 1.25 equiv.) in DMF (17 mL). After 1.0 hours, aqueous work-up followed by column chromatography (Biotage flash chromatographer, 25 g SiO_2; EtOAc in pentane, 10%) afforded dibenzyl 2-(2,4-dimethoxyphenyl)cyclopropane-1,1-dicarboxylate (**2c**) (0.635 g, 1.42 mmol, 61% yield) as a colorless, viscous oil.

R_f (pentane/EtOAc 9/1) 0.26.
^{1}H NMR (400 MHz, Chloroform-*d*) δ 7.35 – 7.28 (m, 5H, Ph*H*), 7.26 – 7.16 (m, 3H, Ph*H*), 7.02 – 6.94 (m, 2H, Ph*H*), 6.88 (dd, *J* = 8.4, 0.8 Hz, 1H, Ar*H*), 6.33 (dd, *J* = 8.3, 2.4 Hz, 1H, Ar*H*), 6.29 (d, *J* = 2.4 Hz, 1H, Ar*H*), 5.30 (d, *J* = 12.5 Hz, 1H, CH_2Ph), 5.18 (d, *J* = 12.5 Hz, 1H, CH_2), 4.83 (d, *J* = 12.3 Hz, 1H, CH_2), 4.74 (d, *J* = 12.3 Hz, 1H, CH_2), 3.78 (s, 3H, O*Me*), 3.60 (s, 3H, O*Me*), 3.28 (t, *J* = 9.2 Hz, 1H, Ar*CH*CH_2), 2.20 (dd, *J* = 8.5, 5.0 Hz, 1H, ArCHCH_2), 1.74 (dd, *J* = 9.2, 5.0 Hz, 1H, ArCHCH_2).
^{13}C NMR (101 MHz, Chloroform-*d*) δ 169.9, 167.0, 160.4, 160.1, 135.9, 135.6, 128.5, 128.5, 128.2, 128.2, 128.0, 127.9, 127.7, 115.4, 103.3, 98.4, 66.9, 66.9, 55.3, 55.3, 36.4, 28.9, 18.6.
HRMS (ESI/QTOF) m/z: $[M + Na]^+$ Calcd for $C_{27}H_{26}NaO_6^+$ 469.1622; Found 469.1622.

Dibenzyl 2-(2-methoxyphenyl)cyclopropane-1,1-dicarboxylate (2d)

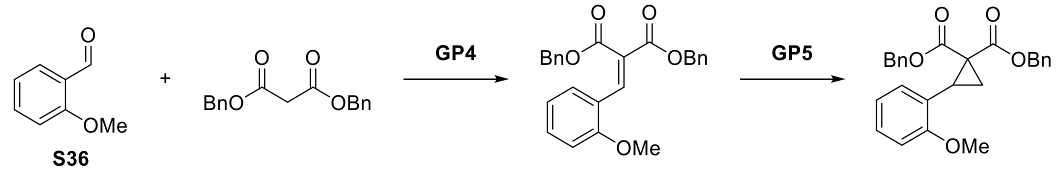

S36 **S37** **2d**

Following the **GP4**, 2-methoxybenzaldehyde (**S36**) (0400 g, 2.94 mmol, 1.0 equiv.) and dibenzyl malonate (0.73 mL, 2.9 mmol, 1.0 equiv.) were reacted with acetic acid (6 drops) and piperidine (4 drops) in toluene (1.0 mL) at reflux and with azeotropical removal of water for 6 hours. Upon cooling to room temperature, the mixture was submitted to column chromatography (Biotage flash chromatographer, 25 g SiO_2; EtOAc in pentane, 12%) to afford dibenzyl 2-(2-methoxybenzylidene)malonate (**S37**) (0.915 g, 2.27 mmol, 77% yield) as a viscous, colorless oil.

Following the **GP5**, a solution of dibenzyl 2-(2-methoxybenzylidene)malonate (**S37**) (0.915 g, 2.27 mmol, 1.0 equiv.) in DMF (dry; 6.0 mL) was added to a mixture of trimethylsulfoxonium iodide (0.663 g, 3.01 mmol, 1.2 equiv.) and NaH (60% dispersion in mineral oil; 0.125 g, 3.14 mmol, 1.25 equiv.) in DMF (19 mL). After 1.0 hours, aqueous work-up followed by column chromatography (Biotage flash chromatographer, 25 g SiO_2; EtOAc in pentane, 13%) afforded dibenzyl 2-(2-methoxyphenyl)cyclopropane-1,1-dicarboxylate (**3d**) (0.440 g, 1.07 mmol, 47% yield) as a viscous, colorless oil.

R_f (pentane/EtOAc 6/4) = 0.85.
^{1}H NMR (400 MHz, Chloroform-*d*) δ 7.37 – 7.30 (m, 5H, Ph*H*), 7.25 – 7.17 (m, 4H, Ph*H*), 6.99 (dd, *J* = 7.6, 2.2 Hz, 1H, Ar*H*), 6.98 – 6.92 (m, 2H, Ar*H*), 6.83 (td, *J* = 7.5, 1.3 Hz, 1H, Ar*H*), 6.73 (dd, *J* = 8.2, 1.2 Hz, 1H, Ar*H*), 5.31 (d, *J* = 12.6 Hz, 1H, *CH₂*Ph), 5.19 (d, *J* = 12.5 Hz, 1H, *CH₂*Ph), 4.76 (d, *J* = 12.4 Hz, 1H, *CH₂*Ph), 4.70 (d, *J* = 12.3 Hz, 1H, *CH₂*Ph), 3.63 (s, 3H, O*Me*), 3.35 (t, *J* = 8.9 Hz, 1H, Ar*CH*CH₂), 2.23 (dd, *J* = 8.5, 5.1 Hz, 1H, ArCH*CH₂*), 1.77 (dd, *J* = 9.2, 5.1 Hz, 1H, ArCH*CH₂*).
^{13}C NMR (101 MHz, Chloroform-*d*) δ 169.8, 166.8, 159.2, 135.9, 135.5, 128.7, 128.5, 128.3, 128.2, 128.1, 128.0, 127.9, 127.7, 123.0, 119.9, 110.1, 67.0, 66.9, 55.3, 36.5, 29.0, 18.6.
HRMS (ESI/QTOF) m/z: $[M + Na]^+$ Calcd for $C_{26}H_{24}NaO_5^+$ 439.1516; Found 439.1530.

Dibenzyl 2-(3-iodo-4-methoxyphenyl)cyclopropane-1,1-dicarboxylate (2e)

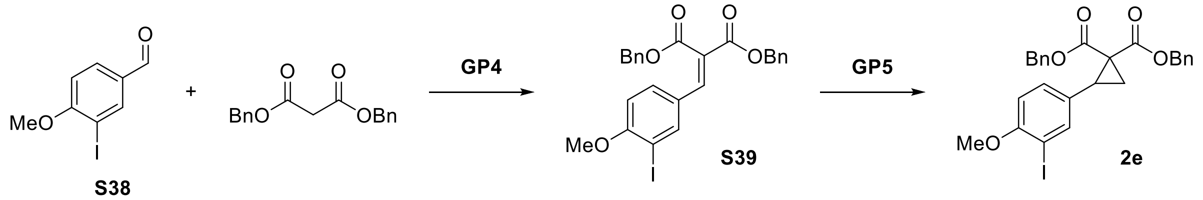

S38

Following the **GP4**, 3-iodo-4-methoxybenzaldehyde (**S38**) (0.50 g, 1.9 mmol, 1.0 equiv.) and dibenzyl malonate (0.48 mL, 1.9 mmol, 1.0 equiv.) were reacted with acetic acid (12 drops) and piperidine (8 drops) in toluene (2.0 mL) at reflux and with azeotropical removal of water for 17 hours. Upon cooling to room temperature, the mixture was submitted to column chromatography (Biotage flash chromatographer, 25 g SiO_2; EtOAc in pentane, 5 to 25%) to afford dibenzyl 2-(3-iodo-4-methoxybenzylidene)malonate (**S39**) (0.716 g, 1.36 mmol, 71% yield) as a viscous, pale yellow oil, which became a solid upon standing at 4 °C.

Following the **GP5**, dibenzyl 2-(3-iodo-4-methoxybenzylidene)malonate (**S39**) (0.716 g, 1.35 mmol, 1.0 equiv.) was added in a single portion to a mixture of trimethylsulfoxonium iodide (0.358 g, 1.63 mmol, 1.2 equiv.) and NaH (60% dispersion in mineral oil; 0.068 g, 1.7 mmol, 1.25 equiv.) in DMF (13 mL). After 1.5 hours, aqueous work-up followed by column chromatography (Biotage flash chromatographer, 25 g SiO_2; EtOAc in pentane, 2 to 20%) afforded dibenzyl 2-(3-iodo-4-methoxyphenyl)cyclopropane-1,1-dicarboxylate (**2e**) (0.400 g, 0.738 mmol, 54% yield) as a colorless, viscous oil.

R_f (pentane/EtOAc 9/1) 0.29.
^{1}H NMR (400 MHz, Chloroform-*d*) δ 7.65 (d, *J* = 2.4 Hz, 1H, Ar*H*), 7.32 (m, 5H, Ar*H*), 7.28 – 7.17 (m, 3H, Ar*H*), 7.07 (dd, *J* = 8.5, 2.6 Hz, 1H, Ar*H*), 7.04 – 6.97 (m, 2H, Ar*H*), 6.60 (d, *J* = 8.5 Hz, 1H, Ar*H*), 5.25 (d, *J* = 12.4 Hz, 1H, O*CH₂*Ph), 5.15 (d, *J* = 12.4 Hz, 1H, O*CH₂*Ph), 4.85 (d, *J* = 12.2 Hz, 1H, O*CH₂*Ph), 4.80 (d, *J* = 12.2 Hz, 1H, O*CH₂*Ph), 3.83 (s, 3H, O*Me*), 3.18 (t, *J* = 8.7 Hz, 1H, Ar*CH*CH₂), 2.15 (dd, *J* = 8.0, 5.2 Hz, 1H, ArCH*CH₂*), 1.73 (dd, *J* = 9.3, 5.2 Hz, 1H, ArCH*CH₂*).
^{13}C NMR (101 MHz, Chloroform-*d*) δ 169.4, 166.4, 157.5, 139.9, 135.4, 135.1, 129.5, 128.6, 128.5, 128.4, 128.3, 128.3, 128.1, 128.0, 110.2, 85.6, 67.4, 67.3, 56.3, 37.3, 31.3, 19.2.
HRMS (ESI/QTOF) m/z: $[M + Na]^+$ Calcd for $C_{26}H_{23}INaO_5^+$ 565.0482; Found 565.0490.

Dibenzyl 2-(*p*-tolyl)cyclopropane-1,1-dicarboxylate (2f)

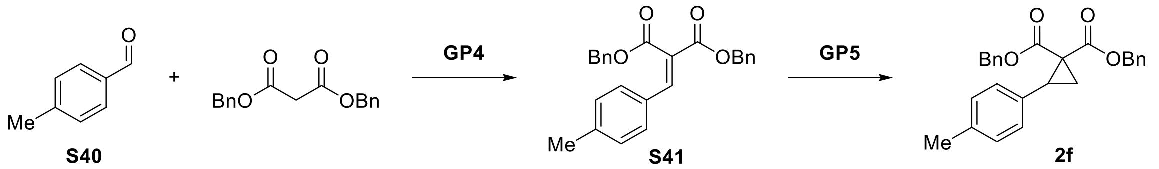

Following the **GP4**, 4-methylbenzaldehyde (**S40**) (0.21 mL, 1.8 mmol, 1.0 equiv.) and dibenzyl malonate (0.50 mL, 2.0 mmol, 1.1 equiv.) were reacted with indium trichloride (40 mg, 0.18 mmol, 10 mol%) and acetic anhydride (0.19 mL, 2.0 mmol, 1.0 equiv.) at 60 °C in toluene (18 mL) for 17 hours. Upon aqueous work-up and column chromatography (Biotage flash chromatographer, 25

g SiO_2; EtOAc in pentane, 2 to 20%), dibenzyl 2-(4-methylbenzylidene)malonate (**S41**) (0.38 g, 0.98 mmol, 55% yield) was obtained as a pale-yellow oil.

Following the **GP5**, dibenzyl 2-(4-methylbenzylidene)malonate (**S41**) (0.38 g, 0.98 mmol, 1.0 equiv.) was added to a mixture of trimethylsulfoxonium iodide (0.26 g, 1.2 mmol, 1.2 equiv.) and NaH (60% dispersion in mineral oil; 0.049 g, 1.2 mmol, 1.2 equiv.) in DMF (9.8 mL). After 1.5 hours, aqueous work-up followed by column chromatography (Biotage flash chromatographer, 25 g SiO_2; EtOAc in pentane, 2 to 15%; then 25 g SiO_2; EtOAc in pentane, 5 to 12%) afforded an off-white solid. Recrystallization from hexane (2.0 mL) provided dibenzyl 2-(p-tolyl)cyclopropane-1,1-dicarboxylate (**3f**) (0.129 g, 0.322 mmol, 33% yield) as white crystalline solid.

^{1}H NMR (400 MHz, Chloroform-*d*) δ 7.36–7.27 (m, 5H, Ar*H*), 7.24–7.16 (m, 3H, Ar*H*), 7.06 (d, *J* = 8.2 Hz, 2H, Ar*H*), 7.02 (d, *J* = 8.0 Hz, 2H, Ar*H*), 6.98–6.91 (m, 2H, Ar*H*), 5.25 (d, *J* = 12.4 Hz, 1H, OCH_2Ph), 5.15 (d, *J* = 12.5 Hz, 1H, OCH_2Ph), 4.80 (d, *J* = 12.3 Hz, 1H, OCH_2Ph), 4.76 (d, *J* = 12.3 Hz, 1H, OCH_2Ph), 3.23 (t, *J* = 8.7 Hz, 1H, Ar*CH*CH_2), 2.31 (s, 3H, Ar*Me*), 2.20 (dd, *J* = 8.1, 5.1 Hz, 1H, ArCHCH_2), 1.74 (dd, *J* = 9.3, 5.1 Hz, 1H, ArCHCH_2).
^{1}H-NMR data corresponded to the reported values.[13]

Dibenzyl 2-(1-tosyl-1*H*-indol-3-yl)cyclopropane-1,1-dicarboxylate (2g)

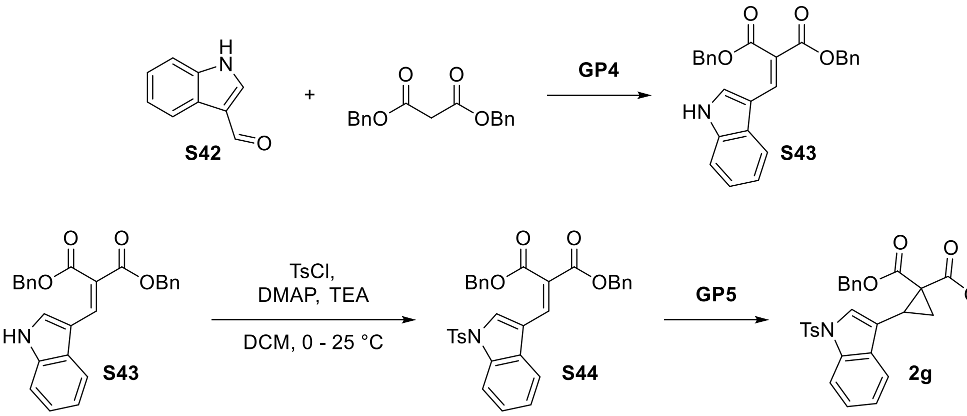

Following the **GP4**, 1*H*-indole-3-carbaldehyde (**S42**) (0.87 g, 6.0 mmol, 1.0 equiv.) and dibenzyl malonate (0.75 mL, 3.0 mmol, 1.0 equiv.) were reacted with acetic acid (12 drops) and piperidine (8 drops) in toluene (2.0 mL) at reflux and with azeotropical removal of water for 5.5 hours. Upon cooling to room temperature, the mixture was submitted to column chromatography (Büchi flash chromatographer, 25 g SiO_2; EtOAc in pentane, 1 to 22%) to afford afford dibenzyl 2-((1*H*-indol-3-yl)methylene)malonate (**S43**) (1.00 g, 2.43 mmol, 41% yield) as a pale yellow solid.

Following a reported procedure,[16] in a 50 mL, two-necked, round-bottomed flask, dibenzyl 2-((1*H*-indol-3-yl)methylene)malonate (**S43**) (0.500 g, 1.21 mmol, 1.0 equiv.) and DMAP (0.027 g, 0.24 mmol, 20 mol%) were dissolved in DCM (12 mL). DIPEA (0.30 mL, 1.7 mmol, 1.4 equiv.) was added, and the mixture was cooled to 0 °C (ice - water bath). Finally, *p*-tosyl chloride (0.0.278 g, 1.46 mmol, 1.2 equiv.) was added. The resulting solution was stirred at room temperature for 4 hours. After this time, the mixture was diluted with DCM (20 mL) and the reaction was quenched by addition of sat. aq. NH_4Cl (20 mL). The layers were separated, and the aqueous one was extracted with DCM (3 x 20 mL). The combined organic layers were washed with brine, dried over $MgSO_4$, filtered, and concentrated under vacuum. The resulting yellow crude became a yellow solid upon standing overnight, which was was submitted to recrystallization from hexanes (2.0 mL) and EtOAc (2.8 mL) to provide dibenzyl 2-((1-tosyl-1*H*-indol-3-yl)methylene)malonate (**S44**) (0.550 g, 0.949 mmol, 78% yield) as a pale yellow, sticky solid.

Following the **GP5**, a solution of dibenzyl 2-((1-tosyl-1*H*-indol-3-yl)methylene)malonate (**S44**) (0.298 g, 0.527 mmol, 1.0 equiv.) in DMF (1.2 mL) was added to a mixture of trimethylsulfoxonium iodide (0.139 g, 0.632 mmol, 1.2 equiv.) and NaH (60% dispersion in mineral oil; 0.26 g, 0.65 mmol, 1.2 equiv.) in DMF (4.2 mL). After 1.5 hours, aqueous work-up followed by column chromatography (Biotage flash chromatographer, 12 g SiO_2; EtOAc in pentane, 7 to 15%) afforded dibenzyl 2-(1-tosyl-1*H*-indol-3-yl)cyclopropane-1,1-dicarboxylate (**2g**) (0.176 g, 0.304 mmol, 58% yield) as a colorless oil, which became a white solid upon standing at 4 °C overnight.

Melting point: 83.4 – 86.6 °C.
R_f (pentane/EtOAc 85/15) = 0.34.
^{1}H NMR (400 MHz, Chloroform-*d*) δ 7.97 (dt, *J* = 8.3, 0.9 Hz, 1H, Ar*H*), 7.71 (d, *J* = 8.6 Hz, 2H, Ar*H*), 7.52 (dt, *J* = 7.8, 1.0 Hz, 1H, Ar*H*), 7.37 (m, 1H, Ar*H*), 7.35 – 7.27 (m, 7H, Ar*H*), 7.22 – 7.13 (m, 2H, Ar*H*), 7.12 (m, 1H, Ar*H*), 7.05 – 6.94 (m, 2H, Ar*H*), 6.52 – 6.46 (m, 2H, Ar*H*), 5.26 (d, *J* = 12.3 Hz, 1H, OCH_2Ph), 5.15 (d, *J* = 12.3 Hz, 1H, OCH_2Ph), 4.50 (d, *J* = 12.1 Hz, 1H, OCH_2Ph), 4.21 (d, *J* = 12.2 Hz, 1H, OCH_2Ph), 3.19 (ddd, *J* = 9.2, 7.9, 1.2 Hz, 1H, Ar*CH*CH_2), 2.20 (s, 3H, Ar*Me*), 2.16 (dd, *J* = 7.9, 5.0 Hz, 1H, ArCHCH_2), 1.82 (dd, *J* = 9.1, 5.0 Hz, 1H, ArCHCH_2).

^{13}C NMR (101 MHz, Chloroform-*d*; the signal corresponding to one aromatic C is not resolved) δ 169.4, 166.3, 144.9, 135.4, 135.1, 134.9, 134.7, 130.9, 129.8, 128.6, 128.3, 128.2, 127.9, 127.7, 126.9, 125.1, 124.2, 123.5, 119.8, 117.4, 113.7, 67.5, 67.0, 37.2, 23.3, 21.5, 18.9.
HRMS (ESI/QTOF) m/z: $[M + Na]^+$ Calcd for $C_{34}H_{29}NNaO_6S^+$ 602.1608; Found 602.1626.

Dibenzyl 2-(benzo[b]thiophen-2-yl)cyclopropane-1,1-dicarboxylate (2h)

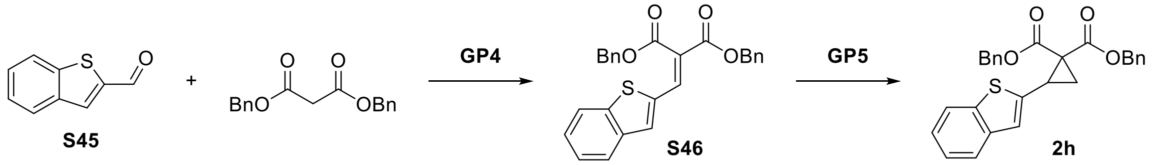

Following the **GP4**, benzo[b]thiophene-2-carbaldehyde (**S45**) (0.487 g, 3.00 mmol, 1.0 equiv.) and dibenzyl malonate (0.75 mL, 3.0 mmol, 1.0 equiv.) were reacted with acetic acid (6 drops) and piperidine (4 drops) in toluene (1.0 mL) at reflux and with azeotropical removal of water for 7 hours. Upon cooling to room temperature, the mixture was submitted to column chromatography (Biotage flash chromatographer, 25 g SiO_2; EtOAc in pentane, 0 to 20%) to afford dibenzyl 2-(benzo[b]thiophen-2-ylmethylene)malonate (**S46**) (ca. 90% pure; 1.01 g, 2.12 mmol, 71% yield) as a viscous yellow oil.

Following the **GP5**, a solution of dibenzyl 2-(benzo[b]thiophen-2-ylmethylene)malonate (**S46**) (ca. 90% pure; 1.01 g, 2.12 mmol, 1.0 equiv.) in DMF (1.0 mL) was added to a mixture of trimethylsulfoxonium iodide (0.54 g, 2.5 mmol, 1.2 equiv.) and NaH (60% dispersion in mineral oil; 0.10 g, 2.6 mmol, 1.22 equiv.) in DMF (8.5 mL). After 1.5 hours, aqueous work-up followed by column chromatography (Biotage flash chromatographer, 25 g SiO_2; EtOAc in pentane, 1 to 20%) afforded dibenzyl 2-(benzo[b]thiophen-2-yl)cyclopropane-1,1-dicarboxylate (**2h**) (0.230 g, 0.520 mmol, 22% yield) as a pale yellow solid.

Melting point: 100.1 – 103.5 °C.
R_f (pentane/EtOAc 85/15) = 0.58.
^{1}H NMR (400 MHz, Chloroform-*d*) δ 7.72 (m, 1H, Ar*H*), 7.63 (m, 1H, ArH), 7.39–7.27 (m, 7H, Ar*H*), 7.13 (m, 1H, Ar*H*), 7.06–6.98 (m, 3H, Ar*H*), 6.92–6.87 (m, 2H, ArH), 5.27 (d, *J* = 12.4 Hz, 1H, CH_2Ph), 5.17 (d, *J* = 12.4 Hz, 1H, CH_2Ph), 4.89 (d, *J* = 12.2 Hz, 1H, CH_2Ph), 4.83 (d, *J* = 12.2 Hz, 1H, CH_2Ph), 3.38 (ddd, *J* = 9.0, 7.8, 1.1 Hz, 1H, Ar*CH*CH_2), 2.26 (dd, *J* = 7.8, 5.2 Hz, 1H, ArCHCH_2), 1.90 (dd, *J* = 9.2, 5.3 Hz, 1H, ArCHCH_2).
^{13}C NMR (101 MHz, Chloroform-*d*; the signals corresponding to two aromatic Cs are not resolved) δ 169.0, 166.1, 139.7, 139.4, 138.7, 135.3, 135.0, 128.6, 128.4, 128.2, 128.0, 124.3, 124.2, 123.5, 122.8, 122.1, 67.6, 67.6, 38.3, 27.9, 20.9.
HRMS (ESI/QTOF) m/z: $[M + Na]^+$ Calcd for $C_{27}H_{22}NaO_4S^+$ 465.1131; Found 465.1138.

Dibenzyl 2-(1-phenyl-1H-pyrazol-4-yl)cyclopropane-1,1-dicarboxylate (2i)

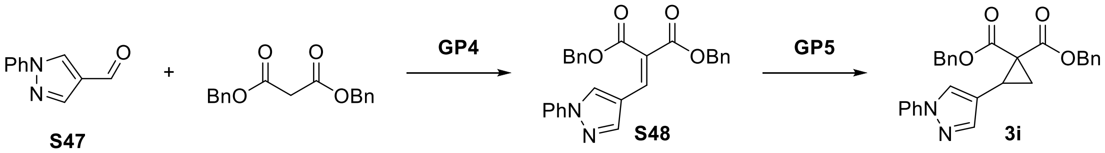

Following the **GP4**, 1-phenyl-1H-pyrazole-4-carbaldehyde (**S47**) (0.225 g, 1.31 mmol, 1.0 equiv.) and dibenzyl malonate (0.32 mL, 1.3 mmol, 1.0 equiv.) were reacted with acetic acid (6 drops) and piperidine (4 drops) in toluene (0.45 mL) at reflux and with azeotropical removal of water for 4 hours. Upon cooling to room temperature, the mixture was submitted to column chromatography (Biotage flash chromatographer, 12 g SiO_2; EtOAc in pentane, 10 to 50%) to afford dibenzyl 2-((1-phenyl-1H-pyrazol-4-yl)methylene)malonate (**S48**) (0.461 g, 0.405 mmol, 80% yield) as a pale yellow solid.
Following the **GP5**, a solution of dibenzyl 2-((1-phenyl-1H-pyrazol-4-yl)methylene)malonate (**S48**) (0.461 g, 0.405 mmol, 1.0 equiv.) in DMF (1.0 mL) was added to a mixture of trimethylsulfoxonium iodide (0.245 g, 1.16 mmol, 1.1 equiv.) and NaH (60% dispersion in mineral oil; 0.48 g, 1.2 mmol, 1.15 equiv.) in DMF (4.2 mL). After 3 hours, aqueous work-up followed by column chromatography (Biotage flash chromatographer, 4 g SiO_2; EtOAc in pentane, 10 to 50%) afforded dibenzyl 2-(1-phenyl-1H-pyrazol-4-yl)cyclopropane-1,1-dicarboxylate (**2i**) (0.095 g, 0.21 mmol, 20% yield) as a pale yellow, amorphous solid.

R_f (pentane / EtOAc 6 : 4) = 0.85.
^{1}H NMR (400 MHz, Chloroform-*d*) δ 7.70 (d, *J* = 0.7 Hz, 1H, Ar*H*), 7.64 – 7.54 (m, 3H, Ar*H*), 7.45 (dd, *J* = 8.5, 7.5 Hz, 1H, Ar*H*), 7.40 – 7.34 (m, 5H, Ar*H*), 7.31 (m, 1H, Ar*H*), 7.24 – 7.12 (m, 3H, Ar*H*), 7.10–7.00 (m, 3H, Ar*H*), 5.29 (d, *J* = 12.3 Hz, 1H, CH_2Ph), 5.18 (d, *J* = 12.3 Hz, 1H, CH_2Ph), 4.96 (d, *J* = 12.2 Hz, 1H, CH_2Ph), 4.91 (d, *J* = 12.2 Hz, 1H, CH_2Ph), 3.12 (dd, *J* = 9.4, 7.8 Hz, 1H, Ar*CH*CH_2), 2.07 (m, 1H, ArCHCH_2), 1.84 (dd, *J* = 9.3, 5.0 Hz, 1H, ArCHCH_2).
^{13}C NMR (101 MHz, Chloroform-*d*; the signal corresponding to one aromatic C is not resolved) δ 169.4, 166.6, 141.0, 139.8, 135.4, 135.2, 129.4, 128.6, 128.4, 128.3, 128.2, 128.1, 126.5, 125.9, 118.9, 117.7, 67.5, 67.5, 37.3, 23.7, 20.5.
HRMS (ESI/QTOF) m/z: $[M + H]^+$ Calcd for $C_{28}H_{25}N_2O_4^+$ 453.1809; Found 453.1819.

(E)-2-(1-phenylprop-1-en-2-yl)cyclopropane-1,1-dicarboxylate (2j)

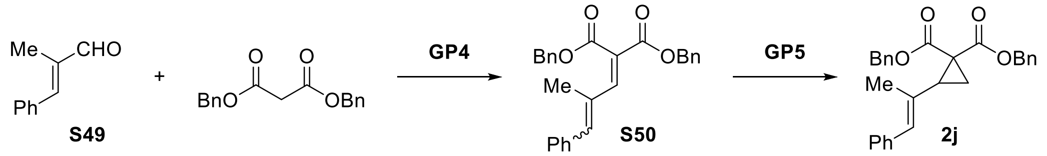

Following the **GP4**, (E)-2-methyl-3-phenylacrylaldehyde (**S49**) (0.43 mL, 3.1 mmol, 1.0 equiv.) and dibenzyl malonate (0.75 mL, 3.0 mmol, 1.0 equiv.) were reacted with acetic acid (6 drops) and piperidine (4 drops) in toluene (1.0 mL) at reflux and with azeotropical removal of water for 4 hours. Upon cooling to room temperature, the mixture was submitted to column chromatography (Biotage flash chromatographer, 25 g SiO_2; EtOAc in pentane, 6%) to afford dibenzyl 2-(2-methyl-3-phenylallylidene)malonate (**S50**) (0.823 g, 2.00 mmol, 65% yield; mixture of *E* and *Z* isomers) as a viscous, orange oil.

Following the **GP5**, a solution of dibenzyl 2-(2-methyl-3-phenylallylidene)malonate (**S50**) (mixture of *E* and *Z* isomers; 0.56 g, 1.3 mmol, 1.0 equiv.) in DMF (4.0 mL) was added to a mixture of trimethylsulfoxonium iodide (0.41 g, 1.9 mmol, 1.2 equiv.) and NaH (60% dispersion in mineral oil; 0.078 g, 1.9 mmol, 1.2 equiv.) in DMF (12 mL). After 1.0 hours, aqueous work-up followed by column chromatography (Biotage flash chromatographer, 25 g SiO_2; EtOAc in pentane, 13%) afforded 2-(1-phenylprop-1-en-2-yl)cyclopropane-1,1-dicarboxylate (mixture of *E* and *Z* isomers; 0.340 g, 0.299 mmol, 51% yield) as a viscous oil. Trituration of the latter with hexane, followed by recrystallization from hexane (2.0 mL) permitted to collect pure (*E*)-2-(1-phenylprop-1-en-2-yl)cyclopropane-1,1-dicarboxylate (**2j**) (0.20 g, 0.47 mmol, 30% yield) as a white solid.

Melting point: 70.9 – 72.8 °C.
R_f (pentane/EtOAc 6/4) 0.92.
^{1}H NMR (400 MHz, Chloroform-*d*) δ 7.38 – 7.28 (m, 7H, Ph*H*), 7.28 – 7.17 (m, 6H, Ph*H*), 7.12 – 7.07 (m, 2H, Ph*H*), 6.29 (s, 1H, PhC*H*=C), 5.25 (d, *J* = 12.4 Hz, 1H, O*CH₂*Ph), 5.15 (d, *J* = 12.5 Hz, 1H, *CH₂*Ph), 5.11 (d, *J* = 12.2 Hz, 1H, O*CH₂*Ph), 5.01 (d, *J* = 12.2 Hz, 1H, *CH₂*Ph), 2.74 (t, *J* = 8.6 Hz, 1H, C=C*CH*CH_2), 2.11 (dd, *J* = 8.2, 5.1 Hz, 1H, C=CCH*CH₂*), 1.82 (m, 3H, C=C*Me*), 1.58 (dd, *J* = 8.9, 5.1 Hz, 1H, C=CCH*CH₂*).
^{13}C NMR (101 MHz, Chloroform-*d*) δ 170.0, 166.8, 137.2, 135.6, 135.4, 131.6, 128.9, 128.6, 128.5, 128.4, 128.3, 128.2, 128.2, 128.1, 127.9, 127.8, 126.5, 67.4, 67.3, 36.8, 36.5, 18.7, 18.7.
HRMS (ESI/QTOF) m/z: $[M + Na]^+$ Calcd for $C_{28}H_{26}NaO_4^+$ 449.1723; Found 449.1718.

Dibenzyl (E)-2-styrylcyclopropane-1,1-dicarboxylate (2k)

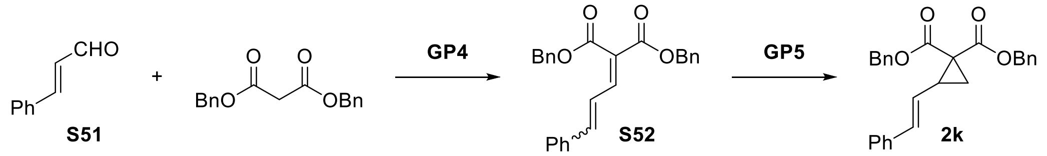

Following the **GP4**, *trans*-cinnamaldehyde (**S51**) (0.38 mL, 3.0 mmol, 1.0 equiv.) and dibenzyl malonate (0.75 mL, 3.0 mmol, 1.0 equiv.) were reacted with acetic acid (6 drops) and piperidine (4 drops) in toluene (1.0 mL) at reflux and with azeotropical removal of water for 4 hours. Upon cooling to room temperature, the mixture was submitted to column chromatography (Büchi flash chromatographer, 25 g SiO_2; EtOAc in pentane, 0 to 25%) to afford dibenzyl 2-(3-phenylallylidene)malonate (**S52**) (0.697 g, 1.75 mmol, 58% yield; mixture of *E* and *Z* isomers) as a viscous, orange oil.

Following the **GP5**, a solution of dibenzyl 2-(3-phenylallylidene)malonate (**S52**) (mixture of *E* and *Z* isomers; 0.69 g, 1.7 mmol, 1.0 equiv.) in DMF (1.0 mL) was added to a mixture of trimethylsulfoxonium iodide (0.46 g, 2.1 mmol, 1.2 equiv.) and NaH (60% dispersion in mineral oil; 0.087 mg, 2.2 mmol, 1.25 equiv.) in DMF (7.0 mL). After 1.5 hours, aqueous work-up followed by column chromatography (Büchi flash chromatographer, 25 g SiO_2; EtOAc in pentane, 0 to 25%) afforded dibenzyl (*E*)-2-styrylcyclopropane-1,1-dicarboxylate (**2k**) (0.20 g, 0.48 mmol, 28% yield) as a very viscous, yellow oil, which became a yellow, amorphous solid upon standing at 4 °C overnight.

Rf (pentane/EtOAc 85/15) = 0.63.
^{1}H NMR (400 MHz, Chloroform-*d*) δ 7.36 – 7.29 (m, 5H, Ar*H*), 7.28 – 7.14 (m, 10H, Ar*H*), 6.62 (d, *J* = 15.8 Hz, 1H, Ph*CH*=CH), 5.75 (dd, *J* = 15.8, 8.8 Hz, 1H, PhCH=*CH*), 5.22 (d, *J* = 12.5 Hz, 1H, *CH₂*Ph), 5.18 (d, *J* = 12.3 Hz, 1H, *CH₂*Ph), 5.13 (dd, *J* = 12.3, 10.1 Hz, 2H, *CH₂*Ph), 2.79 (tdd, *J* = 8.9, 7.7, 0.8 Hz, 1H, C=C*CH*CH_2), 1.88 (dd, *J* = 7.6, 5.0 Hz, 1H, C=CCH*CH₂*), 1.71 (dd, *J* = 9.0, 5.0 Hz, 1H, C=CCH*CH₂*).
^{13}C NMR (101 MHz, Chloroform-*d*; the signal corresponding to one aromatic C is not resolved) δ 169.4, 167.4, 136.6, 135.5, 134.0, 128.6, 128.5, 128.5, 128.3, 128.3, 128.2, 128.1, 127.6, 126.2, 124.5, 67.4, 67.4, 36.3, 31.9, 21.5.
HRMS (ESI/QTOF) m/z: $[M + Na]^+$ Calcd for $C_{27}H_{24}NaO_4^+$ 435.1567; Found 435.1566.

Dibenzyl 2-(1,3-dioxoisoindolin-2-yl)cyclopropane-1,1-dicarboxylate (2l)

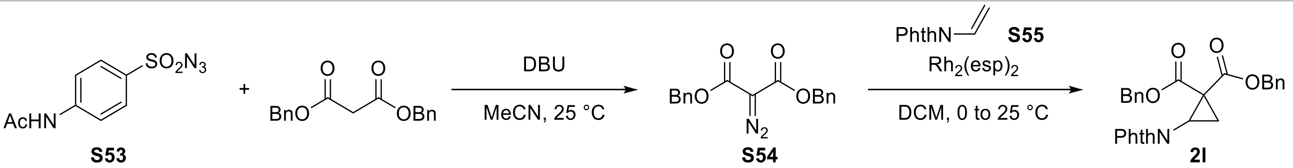

Following a reported procedure,[17] a 100 mL, two necked, round bottomed flask was charged with dibenzyl malonate (1.5 mL, 6.0 mmol, 1.0 equiv.) and acetamidobenzenesulfonyl azide (**S53**) (2.16 g, 9.00 mmol, 1.5 equiv.). MeCN (60 mL) was added, giving a colorless solution. DBU (1.3 mL, 9.0 mmol, 1.5 equiv.) was also added by syringe. The resulting mixture was stirred at room temperature for 3 hours. The reaction was then quenched by addition of sat. aq. NH_4Cl (30 mL), followed by brine. The aqueous layer was extracted with DCM (3 x 40 mL), the combined organic layers were washed with brine, dried over Na_2SO_4, filtered, and concentrated under reduced pressure in the presence of celite. The resulting crude product was submitted to column chromatography (dry load on SiO_2; Biotage flash chromatographer, 40 g SiO_2; EtOAc in pentane, 12 to 18%) to furnish dibenzyl 2-diazomalonate (**S54**) (1.73 g, 5.57 mmol, 93% yield) as a viscous, pale yellow oil, which became an off-white solid upon standing at 4 °C overnight.

^{1}H NMR (400 MHz, Chloroform-*d*) δ 7.40 – 7.30 (m, 10H), 5.28 (s, 4H).
^{1}H-NMR data corresponded to the reported values.[18]

Following a reported procedure,[19] a 100 mL, round-bottomed, two-necked flask was charged with $Rh_2(esp)_2$ (7.7 mg, 0.010 mmol, 0.2 mol%). The flask was sealed with a septum, and evacuated and refilled with nitrogen. DCM (dry; 11.4 mL) was added, followed by N-vinyl phthalimide (**S55**) (0.88 g, 5.1 mmol, 1.0 equiv.). The resulting green solution was cooled to 0 °C (ice - water cooling bath) and stirred for 10 minutes. Subsequently, a solution of dibenzyl diazomalonate (**S54**) (1.73 g, 5.57 mmol, 1.1 equiv.) in DCM (dry; 11.4 mL) was also added drop-wise at the same temperature over a period of 10 minutes. The mixture was then gradually allowed to warm to room temperature overnight, and then stirred for 3 days. After this time, the solution was concentrated under reduced pressure and submitted to column chromatography (Biotage flash chromatographer, 40 SiO_2; EtOAc in pentane, 15 to 30%) to provide dibenzyl 2-(1,3-dioxoisoindolin-2-yl)cyclopropane-1,1-dicarboxylate (**2l**) (0.974 g, 2.14 mmol, 42% yield) was obtained as a viscous oil, which slowly converted into a solid on standing at 4 °C.

^{1}H NMR (400 MHz, Chloroform-*d*) δ 7.80 – 7.73 (m, 2H, *Phth*N), 7.73 – 7.64 (m, 2H, *Phth*N), 7.32 (m, 5H, Ph*H*), 7.22 – 7.14 (m, 5H, Ph*H*), 5.27 (d, *J* = 12.3 Hz, 1H, *CH₂*Ph), 5.21 (d, *J* = 12.3 Hz, 1H, *CH₂*Ph), 5.02 (d, *J* = 12.2 Hz, 1H, *CH₂*Ph), 4.97 (d, *J* = 12.2 Hz, 1H, *CH₂*Ph), 3.74 (dd, *J* = 8.6, 6.6 Hz, 1H, N*CH*CH_2), 2.79 (t, *J* = 6.5 Hz, 1H, NCH*CH₂*), 2.05 (dd, *J* = 8.6, 6.4 Hz, 1H, NCH*CH₂*).
^{1}H-NMR data corresponded to the reported values.[19]

Dibenzyl 2-(3-(4-(*tert*-butyl)benzyl)-5-methyl-2,4-dioxo-3,4-dihydropyrimidin-1(2*H*)-yl)cyclopropane-1,1-dicarboxylate (2m)

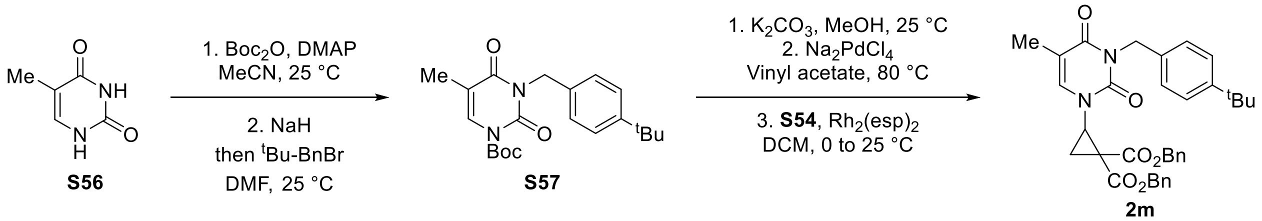

Following a reported procedure,[20] in a 100 mL, round-bottomed, single-necked flask, thymine (**S56**) (1.0 g, 7.9 mmol, 1.0 equiv.) and Boc-anhydride (1.73 g, 7.93 mmol, 1.0 equiv.) were suspended in acetonitrile (40 mL). DMAP (24 mg, 0.20 mmol, 2.5 mol%) was added under stirring at room temperature. Gradually, the suspension converted into a clear solution, which then became turbid after being stirred overnight. After 19 hours, the mixture was concentrated under reduced pressure. The resulting off-white, crude solid was submitted to column chromatography (SiO_2; DCM/MeOH 24/1 to 20/1) to provide , *tert*-butyl 5-methyl-2,4-dioxo-3,4-dihydropyrimidine-1(2*H*)-carboxylate (1.48 g, 6.54 mmol, 83% yield) as a white solid.

Following a reported procedure,[18] in a 100 mL, two-necked, round-bottomed flask, *tert*-butyl 5-methyl-2,4-dioxo-3,4-dihydropyrimidine-1(2*H*)-carboxylate (0.85 g, 3.8 mmol, 1.0 equiv.) was dissolved in DMF (dry; 23 mL). NaH (60% dispersion in mineral oil; 0.18 g, 4.5 mmol, 1.2 equiv.) was then added (release of gas), and the resulting mixture was stirred at room temperature for 30 minutes. After this time, the resulting clear solution was cooled to 0 °C (ice-water bath), and 1-(bromomethyl)-4-(*tert*-butyl)benzene (0.83 mL, 4.5 mmol, 1.2 equiv.) was added. The reaction mixture was then allowed to warm to room temperature, and stirred at room temperature for 1 hour, prior to being partitioned between AcOEt (30 mL) and water (30 mL). The aqueous layer was extracted with AcOEt (4 x 30 mL), and the organic layers were washed with aq. sat. NH_4Cl (30 mL), brine (3 x 30 mL), dried over K_2CO_3, filtered, and concentrated under reduced pressure. The resulting crude oil was submitted to column chromatography (SiO_2; DCM) to provide to provide an oil, from which *tert*-butyl 3-(4-(*tert*-butyl)benzyl)-5-methyl-2,4-dioxo-3,4-dihydropyrimidine-1(2*H*)- carboxylate (**S57**) (1.22 g, 3.28 mmol, 87% yield) precipitated as a whitish powder, upon trituration with pentane.

^{1}H NMR (400 MHz, Chloroform-*d*) δ 7.61 (q, *J* = 1.3 Hz, 1H, Thymine-*H*), 7.46 – 7.38 (m, 2H, Ar*H*), 7.35 – 7.28 (m, 2H, Ar*H*), 5.09 (s, 2H, CH_2Ar), 1.96 (d, *J* = 1.4 Hz, 3H, Thymine-*Me*), 1.60 (d, *J* = 2.5 Hz, 9H, *Boc*), 1.28 (s, 9H, Ar-^{t}*Bu*).
^{1}H-NMR data corresponded to the reported values.[18]

Following a reported procedure,[18] in a 100 mL, single-necked, round-bottomed flask, *tert*-butyl 3-(4-(*tert*-butyl)benzyl)-5-methyl-2,4-dioxo-3,4-dihydropyrimidine-1(2H)-carboxylate (**S57**) (1.22 g, 3.28 mmol, 1.0 equiv.) was suspended in methanol (33 mL). K_2CO_3 (0.453 g, 3.28 mmol, 1.0 equiv.) was added in a single portion. The resulting suspension was vigorously stirred at room temperature for 4.5 hours, progressively converting in a completely clear, colorless solution. Water (50 mL) was then added. The resulting aqueous solution was extracted with DCM (4 x 50 mL). The combined organic layers were washed once with sat. aq. NH_4Cl (50 mL), brine, dried over K_2CO_3, filtered, and concentrated under vacuum. 3-(4-(*tert*-Butyl)benzyl)-5-methylpyrimidine-2,4(1*H*,3*H*)-dione (0.724 g, 2.66 mmol, 81% yield) was obtained as a white solid, which was not submitted to further purification.

^{1}H NMR (400 MHz, Chloroform-*d*) δ 9.58 (s, 1H, Thymine-N*H*), 7.45 – 7.37 (m, 2H, Ar*H*), 7.37 – 7.29 (m, 2H, Ar*H*), 6.99 (dq, *J* = 5.6, 1.3 Hz, 1H, Thymine-*H*), 5.09 (s, 2H, CH_2Ar), 1.92 (d, *J* = 1.3 Hz, 3H, Thymine-*Me*), 1.29 (s, 9H, Ar-^{t}*Bu*).
^{1}H-NMR data corresponded to the reported values.[18]

Following a reported procedure,[18] in a sealed 25 mL, round-bottomed vial, 3-[(4-tert-Butylphenyl)methyl]-5-methyl-1H-pyrimidine-2,4-dione (0.724 g, 2.66 mmol, 1.0 equiv.) and sodium tetrachloropalladate (78 mg, 0.27 mmol, 10 mol%) were suspended in vinyl acetate (10 mL). The orange mixture was stirred at 80 °C for 24 hours. After this time, TLC analysis (DCM/MeOH 98/2) showed that the starting material had been entirely converted. The mixture was then allowed to cool down to room temperature, and then concentrated under reduced pressure. The resulting orange-brown crude oil was submitted to column chromatography (Biotage, 12 g SiO_2; EtOAc in DCM, 1 to 8%) to provide 3-(4-(*tert*-butyl)benzyl)-5-methyl-1-vinylpyrimidine-2,4(1*H*,3*H*)-dione (0.664 g, 2.22 mmol, 84% yield) as an off-white solid.

^{1}H NMR (400 MHz, Chloroform-*d*) δ 7.44 (d, *J* = 8.6 Hz, 2H, Ar*H*), 7.34 – 7.30 (m, 2H, Ar*H*), 7.30 (t, *J* = 1.3 Hz, 1H, Thymine-*H*), 7.23 (d, *J* = 9.0 Hz, 1H, Vinyl-*H*), 5.12 (s, 2H, CH_2Ar), 5.02 (dd, *J* = 16.0, 1.8 Hz, 1H, Vinyl-*H*), 4.88 (dd, *J* = 9.1, 2.1 Hz, 1H, Vinyl-*H*), 2.00 (d, *J* = 1.3 Hz, 3H, Thymine-*Me*), 1.29 (s, 9H, Ar-^{t}*Bu*).
^{1}H-NMR data corresponded to the reported values.[18]

Following a reported procedure,[19] a 100 mL, round-bottomed, two-necked flask was charged with $Rh_2(esp)_2$ (3.1 mg, 4.0 μmol, 0.3 mol%) and 3-(4-(*tert*-butyl)benzyl)-5-methyl-1-vinylpyrimidine-2,4(1*H*,3*H*)-dione (0.400 g, 1.34 mmol, 1.0 equiv.). The flask was sealed with a septum, and evacuated and refilled with nitrogen. DCM (dry; 12 mL) was added by syringe at room temperature. Subsequently, a solution of dibenzyl diazomalonate (**S54**) (0.624 g, 2.01 mmol, 1.5 equiv.) in DCM (dry; 4 mL) was also added drop-wise at the same temperature. The green solution was then stirred for 22 hours. After this time, the solution was concentrated under reduced pressure and submitted to column chromatography (Biotage, 25 g SiO_2; EtOAc in pentane, 7 to 50%) to provide dibenzyl 2-(3-(4-(*tert*-butyl)benzyl)-5-methyl-2,4-dioxo-3,4-dihydropyrimidin-1(2*H*)-yl)cyclopropane-1,1-dicarboxylate (**2m**) (0.281 g, 0.484 mmol, 36% yield) as an off-white foam that collapsed to a pale yellow, very viscous oil upon standing at room temperature.

^{1}H NMR (400 MHz, Chloroform-*d*) δ 7.40 – 7.35 (m, 2H, Ar*H*), 7.33 – 7.28 (m, 4H, Ar*H*), 7.28 – 7.21 (m, 6H, Ar*H*), 7.16 – 7.09 (m, 2H, Ar*H*), 6.85 (d, *J* = 1.3 Hz, 1H, Thymine-*H*), 5.24 (d, *J* = 12.4 Hz, 1H, CH_2Ar), 5.18 (d, *J* = 12.4 Hz, 1H, CH_2Ar), 5.02 – 4.94 (m, 2H, CH_2Ar), 4.91 (d, *J* = 12.2 Hz, 1H, CH_2Ar), 4.79 (d, *J* = 12.4 Hz, 1H, CH_2Ar), 4.08 (dd, *J* = 8.2, 6.6 Hz, 1H, N*CH*CH_2), 2.26 (t, *J* = 6.6 Hz, 1H, NCHCH_2), 1.91 (dd, *J* = 8.2, 6.4 Hz, 1H, NCHCH_2), 1.84 (d, *J* = 1.2 Hz, 3H, Thymine-*Me*), 1.22 (s, 9H, Ar-^{t}*Bu*).
^{13}C NMR (101 MHz, Chloroform-*d*) δ 167.3, 165.5, 163.2, 151.7, 150.5, 136.7, 135.1, 134.9, 133.8, 128.9, 128.6, 128.5, 128.5, 128.4, 128.4, 128.2, 125.3, 110.0, 67.9, 67.9, 44.2, 43.9, 35.3, 34.4, 31.3, 20.2, 13.2.
HRMS (ESI/QTOF) m/z: $[M + H]^+$ Calcd for $C_{35}H_{37}N_2O_6^+$ 581.2646; Found 581.2668.

3. Optimization of the reaction

Lewis acid catalyzed (4+3) annulation of dialkyl 2-(4-methoxyphenyl)cyclopropane-1,1-dicarboxylates 2a-a" with O^{i}Pr-substituted azadiene 1

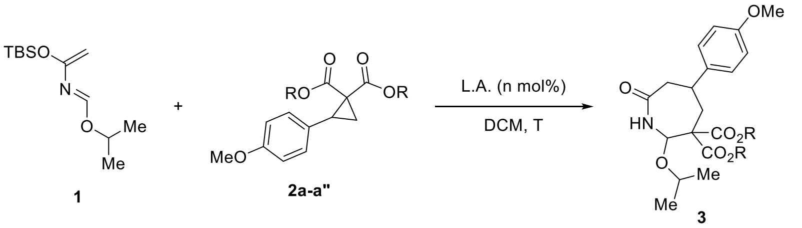

Inside a glove box, a 10 mL, round bottom vial was charged with the Lewis acid (0.050 mmol, 20 mol%) and the dialkyl 2-(4-methoxyphenyl)cyclopropane-1,1-dicarboxylates **2a-a"** (0.25 mmol, 1.0 equiv.). The vial was sealed with a PTFE cap and taken out of the glovebox. DCM (1.7 mL) was added by syringe to give a pale yellow solution, which was cooled to 0 °C (ice - water bath). Propan-2-yl (1E)-N-[1-[*tert*-butyl(dimethyl)silyl]oxyethenyl]methanimidate (**1**) (0.092 g, 0.37 mmol, 1.5 equiv.) was added by syringe. The mixture was stirred at 0 °C for 5 minutes. The cooling bath was then removed and stirring was continued at room temperature. The reaction was then quenched by addition of methanol (1 mL), followed by stirring for 15 minutes. The volatiles were then removed by evaporation under reduced pressure, and the resulting orange crude oil was submitted to column chromatography.
Full decomposition of the starting material was observed when dimethylester **2a'** was submitted to the reaction in the presence of $Yb(OTf)_3$ as the catalyst, with no product formation (Table 1, entry 1). Under the same conditions, di*iso*propylester **2a"** was recovered unreacted after 48 hours (entry 2). Dibenzyl ester **2a** could be successfully converted into the corresponding lactam **4** using either catalytic $Yb(OTf)_3$ (entries 3, 4) or $Sc(OTf)_3$ (entry 5). Both catalysts allowed to isolate the product in > 30% yield; better results were however gotten with the former (compare entry 3 with entry 5). Based on more than two reiterations, neither catalyst permitted to obtain reproducible yields. Significant variations of the d.r. (not reported in Table 1) were also observed. Noteworthy, addition of activated MS 3Å reduced significantly the reaction time, and led to a good yield (62%) when $Yb(OTf)_3$ was used as the catalyst (entry 4). With other catalytic Lewis acids, the reaction did not take place or proceeded sluggishly (entries 6-10).

Table S1. Tentative optimization of the reaction using O^{i}Pr-substituted azadiene **1**.

Entry	Cyclopropane (R)	Catalyst	Reaction time	Yield
1	**2a'** (Me)	$Yb(OTf)_3$	24 h	decomposition
2	**2a"** (^{i}Pr)	$Yb(OTf)_3$	48 h	n.c.[a]
3	**2a** (Bn)	$Yb(OTf)_3$	24 h	33 - 72%
4	**2a** (Bn)	$Yb(OTf)_3$[b]	3 h	62%
5	**2a** (Bn)	$Sc(OTf)_3$	24 h	33 - 46%
6	**2a** (Bn)	$SnCl_4$	24 h	n.c.
7	**2a** (Bn)	$GaCl_3$	24 h	n.c.
8	**2a** (Bn)	$Cu(OTf)_2$	24 h	n.c.
9	**2a** (Bn)	MgI_2	< 7 h	~20%[c]
10	**2a** (Bn)	$La(OTf)_3$	24 h	Traces

Reaction conditions: 0.25 mmol cyclopropane **2a-a"**, 0.37 mmol **1**; 0.050 mmol catalyst, in 1.7 mL DCM, at room temperature. Yield determined by isolation through column chromatography. [a] 0.50 mmol **1** (2.0 equiv.) were used; [b] with 100 mg MS 3Å; [c] yield estimated by ^{1}H-NMR, using trichloroethylene (0.0225 mL, ca. 0.250 mmol) as an internal standard

Dibenzyl 2-isopropoxy-5-(4-methoxyphenyl)-7-oxoazepane-3,3-dicarboxylate (3)

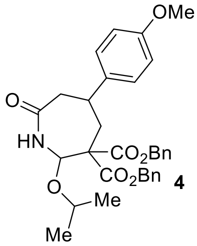

4

Upon column chromatography (Biotage flash chromatographer, 80 g SiO_2; EtOAc in pentane, 10 to 80%), the title compound was collected as a viscous, pale yellow oil. Mixture of diastereoisomers; over > 2 reiteration of the reaction using 20 mol% $Yb(OTf)_3$ as the catalyst, d.r. was found between 80 : 20 and 60 : 40. For characterization purposes, the batch of **4** obtained from one single experiment was considered.

R_f (pentane/EtOAc 6/4) 0.50.
^{1}H NMR (400 MHz, Acetonitrile-d_3; part of the signals corresponding to the *minor isomer* could not be resolved; the signals that could be assigned to the *minor isomer* are underlined; residual EtOAc peaks are visible in the spectrum) δ 7.40 – 7.28 (m, 8H, Ar*H*), 7.28 – 7.18 (m, 2H, Ar*H*), 7.13 – 7.09 (m, 2H, Ar*H*) 7.07 – 7.00 (m, 2H), 6.86 (br s, 1H. (CO)N*H*), 6.86 – 6.81 (m, 2H, Ar*H*), 5.27 (d, *J* = 12.3 Hz, 1H, *CH₂*Ph), 5.13 (m, 1H, *CH₂*Ph), 5.09 (d, *J* = 3.3 Hz, 1H, *CH₂*Ph), 5.05 (d, *J* = 12.3 Hz, 1H, *CH₂*Ph), 3.86 (p, *J* = 6.1 Hz, 1H, C*H*O^{i}Pr), 3.75 (s, 3H, O*Me*), 3.74 (s, 3H, O*Me*), 3.13 (dd, *J* = 13.7, 12.4 Hz, 1H, *CH* or *CH₂*), 2.89 (m, 1H, *CH* or *CH₂*), 2.48 (d, *J* = 12.0 Hz, 1H, *CH* or *CH₂*), 2.40 (dddd, *J*

= 14.1, 2.6, 1.8, 0.9 Hz, 1H, *CH* or CH_2), 2.26 (dq, *J* = 13.6, 2.0 Hz, 1H, *CH* or CH_2), 1.20 (t, *J* = 7.1 Hz, 3H, OCH(*Me*)$_2$), 1.20 – 1.13 (m, 3H, OCH(*Me*)$_2$), 1.01 (d, *J* = 6.1 Hz, 3H, OCH(*Me*)$_2$), 0.99 (d, *J* = 6.1 Hz, 3H, OCH(*Me*)$_2$).

^{13}C NMR (101 MHz, Acetonitrile-*d*$_3$; part of the signals corresponding to the *minor isomer* could not be resolved; the signals that could be assigned to the *minor isomer* are underlined; residual EtOAc peaks are present) δ 175.8, 169.7, 168.7, 168.6, 168.4, 159.2, 159.2, 139.7, 136.6, 136.5, 136.4, 136.4, 114.9, 114.8, 80.8, 70.0, 68.5, 68.3, 68.0, 62.7, 55.8, 44.8, 43.7, 38.7, 37.4, 23.4, 23.2, 21.1.

HRMS (ESI/QTOF) m/z: [M + Na]$^+$ Calcd for $C_{32}H_{35}NNaO_7^+$ 568.2306; Found 568.2314.

Lewis acid catalyzed (4+3) annulation of 2-(4-methoxyphenyl)cyclopropane-1,1-dicarboxylates 2a-a''' with Ph-substituted azadiene 3a: *General procedures*

GP5.[a]: (4+3) annulation of varying dialkyl diester DA cyclopropanes under Yb(OTf)$_3$ catalysis.

Inside a glove box, a 10 mL, round bottom vial was charged with Yb(OTf)$_3$ (31 mg, 0.050 mmol, 20 mol%) and the dialkyl 2-(4-methoxyphenyl)cyclopropane-1,1-dicarboxylates **2a-a'''** (0.25 mmol, 1.0 equiv.). The vial was sealed with a PTFE cap and taken out of the glovebox. DCM (1.7 mL) was added by syringe to give a pale yellow solution, which was cooled to 0 °C (ice - water bath). N-Benzylidene-1-((*tert*-butyldimethylsilyl)oxy)ethenamine (**4a**) (0.098 g, 0.37 mmol, 1.5 equiv.) was added by syringe. The mixture was stirred at 0 °C for 5 minutes. The cooling bath was then removed and stirring was continued at room temperature overnight (18 hours). The reaction mixture was then concentrated under reduced pressure. The crude product was submitted to column chromatography.

GP5.[b]: (4+3) annulation of dibenzyl diester DA cyclopropane with varying LA catalyst.

Inside a glove box, a 10 mL, round bottom vial was charged with the Lewis acid (0.020 mmol, 20 mol%) and dibenzyl 2-(4-methoxyphenyl)cyclopropane-1,1-dicarboxylates **2a** (0.042 g, 0.10 mmol, 1.0 equiv.). The vial was sealed with a PTFE cap, and taken out of the glovebox. DCM (0.8 mL) was added by syringe to give a pale yellow solution, which was cooled to 0 °C (ice - water bath). N-Benzylidene-1-((*tert*-butyldimethylsilyl)oxy)ethenamine (**4a**) (0.039 g, 0.15 mmol, 1.5 equiv. or 0.052 g, 0.20 mmol, 2.0 equiv.) was added by syringe. The mixture was stirred at 0 °C for 5 minutes. The cooling bath was then removed and stirring was continued at room temperature overnight (18 hours). The reaction mixture was then filtered through a short plug of SiO_2, which was then washed with several portions of DCM/MeOH 9/1. The filtrate was concentrated by evaporation under reduced pressure. Yield was determined upon isolation of the product through column chromatography, or estimated by ^{1}H-NMR analysis, using trichloroethylene (56 μL, ca. 0.100 mmol) as an internal standard

GP5.[f]: (4+3) annulation of dibenzyl diester DA cyclopropane in the presence of activated molecular sieves.

Inside a glove box, a 10 mL, round bottomed vial was charged with the Lewis acid (0.020 mmol, 20 mol%), the ligand (with Cu(OTf)$_2$; 0.022 mmol, 22 mol%), and molecular sieves 3Å (60-70 mg). The vial was sealed with a PTFE cap, and taken out of the glovebox. DCM (0.6 mL) was added by syringe, and the resulting suspension was stirred at room temperature for 30 minutes (3 hours with Cu(OTf)$_2$). Finally, a solution of dibenzyl 2-(4-methoxyphenyl)cyclopropane-1,1-dicarboxylates **2a** (0.042 g, 0.10 mmol, 1.0 equiv.) and N-Benzylidene-1-((*tert*-butyldimethylsilyl)oxy)ethenamine (**4a**) (0.039 g, 0.15 mmol, 1.5 equiv. or 0.052 g, 0.20 mmol, 2.0 equiv.) in DCM (0.4 mL) was also added. The mixture was stirred at room temperature overnight (18 hours). The solids were then filtered off through a short plug of celite, which was then washed with several portions of DCM/MeOH 9/1. The filtrate was concentrated by evaporation under reduced pressure. Yield was determined upon isolation of the product through column chromatography.

Synthesis of 2,2'-(propane-2,2-diyl)bis(4,5-dihydrooxazole) (*rac*-Box, L1)

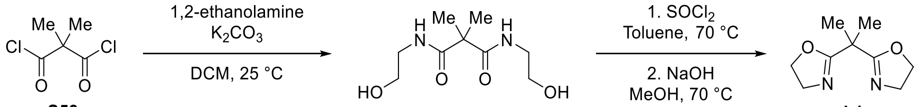

S58 **S59** **L1**

Following a reported procedure,[21] a 100 ml, two-necked, round-bottomed flask was charged with K_2CO_3 (finely ground in a mortar; 2.09 g, 15.1 mmol, 4.0 equiv.). DCM (37 mL) was then added, followed by ethanolamine (freshly distilled on Na_2SO_4; 0.48 mL, 7.9 mmol, 2.1 equiv.). The resulting suspension was cooled to 0 °C (ice - water bath). A solution of 2,2-dimethylmalonyl dichloride (**S58**) (0.50 mL, 3.8 mmol, 1.0 equiv.) in DCM (9 mL) was added drop-wise over a period of 10 minutes. The resulting white, homogeneous suspension was stirred for 24 hours, while allowing it to warm to room temperature. MeOH (38 mL) was then added and stirring was continued for 2 hours. After this time, the suspension was filtered through a pad of celite, which was then washed with MeOH (4 x 30 mL). The filtrate was concentrated under vacuum to afford N^1,N^3-bis(2-hydroxyethyl)-2,2-dimethylmalonamide (**S59**) (assumably, quantitative yield) as a waxy, off-white solid, which was directly used in the following step.

Following a reported procedure,[21] in a 50 mL, two-necked, round-bottomed flask, equipped with a Liebig condenser, crude N^1,N^3-bis(2-hydroxyethyl)-2,2-dimethylmalonamide (**S59**) (0.825 g, 3.78 mmol, 1.0 equiv.) was dissolved in toluene (22 mL). The suspension was heated to 70 °C. Thionyl chloride (1.1 mL, 15 mmol, 4.0 equiv.) was then added in a rapid drop-wise manner at this temperature, resulting in the mixture becoming a clear, pale yellow solution. The latter was then stirred at 70 °C for 5 hours. After this time, the mixture was cooled to 0 °C and the reaction quenched by treatment with sat. aq. $NaHCO_3$ (20 mL; gas release!).

The aqueous layer was extracted with DCM (5 x 20 mL). The combined organic layers were dried over Na_2SO_4, filtered, and concentrated under vacuum, to provide an off-white crude solid, which was directly used as such in the following step.

Following a reported procedure,[21] in a sealed 25 mL, round-bottomed vial, the crude N^1,N^3-bis(2-chloroethyl)-2,2-dimethylmalonamide obtained from the previous step was suspended in a solution of NaOH in MeOH (5% w/w; 20 mL). The suspension was heated to 70 °C. The solids were dissolved to give a clear colorless solution. Stirring was continued at the same temperature for 3 hours. The mixture was then allowed to cool down to room temperature and concentrated under reduced pressure. The so-obtained solid was partitioned between water (20 mL) and DCM (20 mL). The aqueous layer was extracted with DCM (5 x 20 mL). The combined organic layers were dried over Na_2SO_4, filtered, and concentrated under vacuum to provide pure 2,2'-(propane-2,2-diyl)bis(4,5-dihydrooxazole) (*rac*-Box, **L1**) (0.501 g, 2.75 mmol, 73% yield) as a colorless, viscous oil, which converted into an off-white solid on standing at 4 °C.

^{1}H NMR (400 MHz, Chloroform-*d*) δ 4.29 (t, *J* = 9.5 Hz, 4H, CH_2), 3.88 (t, *J* = 9.5 Hz, 4H, CH_2), 1.52 (s, 6H, 2 x *Me*).
^{1}H-NMR data corresponded to the reported values.[21]

Table S2. Optimization of the reaction using Ph-substituted azadiene **2a**.

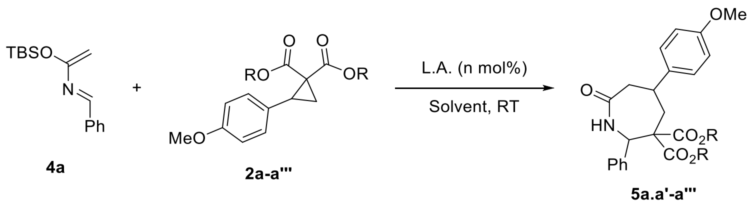

Entry	R	Catalyst (n mol%)	MS 3Å (60-70 mg)	Equiv. **3a**	Solvent	Yield	d.r.
1[a]	Me	$Yb(OTf)_3$ (20)	NO	1.5	DCM	decomp.	---
2[a]	^{i}Pr	$Yb(OTf)_3$ (20)	NO	1.5	DCM	40%	> 95 : 5
3[a]	neoPentyl	$Yb(OTf)_3$ (20)	NO	1.5	DCM	35%	> 95 : 5
4[a]	Bn	$Yb(OTf)_3$ (20)	NO	1.5	DCM	80%	95 : 5
5[b]	Bn	$Sc(OTf)_3$ (20)	NO	1.5	DCM	~50%[c]	90 : 10
6[b]	Bn	$Dy(OTf)_3$ (20)	NO	1.5	DCM	53%	> 95 : 5
7[b]	Bn	MgI_2 (20)	NO	1.5	DCM	57%	63 : 37
8[b]	Bn	$Mg(OTf)_3$ (20)	NO	1.5	DCM	n.r.	---
9[b]	Bn	$Cu(OTf)_3$ (20)	NO	1.5	DCM	71%	89 : 11
10[b]	Bn	$Sn(OTf)_2$ (20)	NO	1.5	DCM	n.r.	---
11[b]	Bn	$Hf(OTf)_4$ (20)	NO	1.5	DCM	traces	---
12[b]	Bn	$Yb(OTf)_3$ (20)	NO	1.5	DCE	67%	> 95 : 5
13[b]	Bn	$Yb(OTf)_3$ (20)	NO	1.5	THF	~45%[c]	> 95 : 5
14[b]	Bn	$Yb(OTf)_3$ (20)	NO	1.5	Toluene	~40%[c]	> 95 : 5
15[b]	Bn	$Yb(OTf)_3$ (10)	NO	1.5	DCM	41%	> 95 : 5
16[b]	Bn	$Yb(OTf)_3$ (10)	NO	2.0	DCM	48%	> 95 : 5
17[b]	Bn	$Yb(OTf)_3$ (10)	NO	2.0	DCE	59%[d]	87 : 13
18[b]	Bn	$Yb(OTf)_3$ (10)	NO	1.5	DCE	62%[e]	60 : 40
19	Bn	$Yb(OTf)_3$ (10)	Yes[f]	2.0	DCM	77%	> 95:5
20	Bn	$Yb(OTf)_3$ (20)	Yes[f]	1.5	DCM	89%	95 : 5
21	Bn	$Yb(OTf)_3$ (20)	Yes[f]	1.0[g]	DCM	84%	94 : 6
22	Bn	$Yb(OTf)_3$ (20)	Yes[f]	1.0[h]	DCM	75%	93 : 7
23	Bn	$Cu(OTf)_2$ (20) Box (**L1**)	Yes[f]	1.5	DCM	83%	70 : 30
24	Bn	$Cu(SbF_6)_2$[i] (20) Box (**L1**)	Yes[f]	1.5	DCM	96%	35 : 65

[a]Reaction conditions: 0.25 mmol cyclopropane **2a-a'''**, 0.37 mmol **4a**; 0.050 mmol catalyst, in 1.7 mL DCM, at room temperature. Yield determined by isolation through column chromatography; [b] reaction conditions: 0.10 mmol cyclopropane **2a**, 0.15 mmol **4a**; 20 mol % catalyst, in 0.8 – 1.0 mL DCM, at room temperature. Yield determined by isolation through column chromatography; [c] yield estimated by ^{1}H-NMR, using trichloroethylene (56 μL, ca. 0.100 mmol) as an internal standard; [d] stirred at 60 °C for 3 hours, then at 80 °C for another 2 hours; [e] stirred at 80 °C for 2 hours; [f] molecular sieves pre-stirred with **2a** in 0.6 mL DCM at room temperature for 30 minutes prior to the addition of a solution of **4a** in 0.4 mL DCM; [g] with 0.15 mmol (1.5 equiv.) cyclopropane **2a**, 0.10 mmol (1.0 equiv.) azadiene **4a**; [h] with 0.10 mmol (1.0 equiv.) cyclopropane **2a**, 0.10 mmol (1.0 equiv.) azadiene **4a**; [i] generated in situ by stirring $CuBr_2$ (20 mol%) with $AgSbF_6$ (40 mol%).

3.1 Speculative model for diastereoselectivity

A very speculative and not conclusive model tentatively explaining the observed *trans* diastereoselectivity is shown in Scheme S1. Two hypothetical pathways are proposed for the reaction:

Path A: The enolate portion of azadiene **4a** undergoes a (3+2) annulation with cyclopropane **2a**, leading to the formation of 5-membered intermediate **I** or **II**. The rearrangement of the latter would then occur with ring-expansion, and result in azepanone product **5a.a**. Stronger sterical repulsion in **II** between the diester moiety and the OTBS group would make such an intermediate less favorable than **I**, giving a rationale for the *trans* selectivity. This hypothesis would be more in agreement witht the mechanistic proposal of Tang and co-workers for their (4+3) annulation of DA cyclopropanes and dienolsilyl ethers (cfr. Ref. 10b in main part).

Path B: The nucleophilic attack of the enolate in **4a** to **2a** would occur in a non-annulative manner, leading to the formation of acyclic intermediate **III** or **IV**. In **III**, the imine moiety would adopt an s-trans conformation; the latter would be more sterically favorable compared to the s-cis conformation in **IV**, which would be subjected to stronger gauche interactions. Gauche interactions would be weaker in **III** because the lone pair of the imino-Nitrogen atom would be oriented towards the diester instead of the imino group itself.

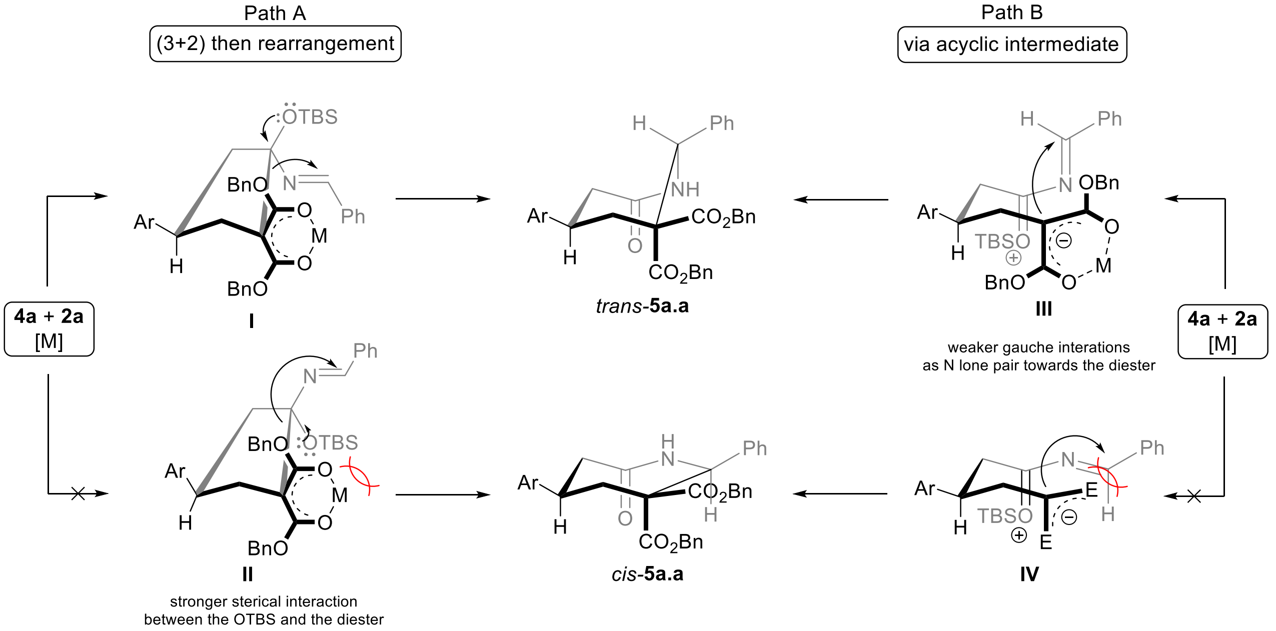

Scheme S1: Origin of the observed diastereoselectivity.

4. Scope of the reaction and characterization of the (4+3) annulation products

Di*iso*propyl 2,5-*trans*-5-(4-methoxyphenyl)-7-oxo-2-phenylazepane-3,3-dicarboxylate (5a.a'')

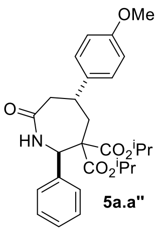

The title compound was prepared following the **GP5.[a]**, using di*iso*propyl 2-(4-methoxyphenyl)cyclopropane-1,1-dicarboxylate (**2a''**) (0.080 g, 0.25 mmol, 1.0 equiv.), benzylidene-1-((*tert*-butyldimethylsilyl)oxy)ethenamine (**4a**) (0.039 g, 0.15 mmol, 1.5 equiv.) and $Yb(OTf)_3$ (31 mg, 0.050 mmol, 20 mol%). Upon column chromatography (Biotage flash chromatographer, 4 g SiO_2; EtOAc in pentane, 15 to 60%), it was obtained (0.040 g, 0.10 mmol, 40% yield; one diastereoisomer) as a white, foamy solid.

R_f (pentane/EtOAc 6/4) 0.47.

^{1}H NMR (400 MHz, Chloroform-*d*) δ 7.70 – 7.62 (m, 2H, Ar*H*), 7.39 – 7.28 (m, 3H, Ar*H*), 7.17 – 7.10 (m, 2H, Ar*H*), 6.88 (d, *J* = 8.7 Hz, 2H, Ar*H*), 6.14 (d, *J* = 6.9 Hz, 1H, (CO)N*H*), 5.25 (p, *J* = 6.2 Hz, 1H, O*CH*(Me)$_2$), 5.01 (d, *J* = 7.3 Hz, 1H, NC*H*Ar), 4.52 (p, *J* = 6.2 Hz, 1H, O*CH*(Me)$_2$), 3.80 (s, 3H, O*Me*), 3.20 (m, 1H, C*H$_2$*), 2.94 (dd, *J* = 13.2, 10.7 Hz, 1H, C*H*), 2.75 (dt, *J* = 14.2, 2.2 Hz, 1H, C*H$_2$*), 2.70 (m, 1H, C*H$_2$*), 2.51 (dd, *J* = 14.2, 12.3 Hz, 1H, C*H$_2$*), 1.37 (d, *J* = 6.3 Hz, 3H, OCH(*Me*)$_2$), 1.28 (d, *J* = 6.2 Hz, 3H, OCH(*Me*)$_2$), 0.92 (d, *J* = 6.3 Hz, 3H, OCH(*Me*)$_2$), 0.56 (d, *J* = 6.2 Hz, 3H, OCH(*Me*)$_2$).

^{13}C NMR (101 MHz, Chloroform-*d*) δ 173.8, 168.8, 167.6, 158.4, 137.8, 137.0, 129.4, 128.7, 128.6, 127.3, 114.2, 69.6, 68.9, 62.9, 62.8, 55.3, 47.6, 42.8, 37.5, 21.9, 21.6, 21.2, 20.6.

HRMS (nanochip-ESI/LTQ-Orbitrap) m/z: $[M + Na]^+$ Calcd for $C_{27}H_{33}NNaO_6^+$ 490.2200; Found 490.2210.

Dineopentyl 2,5-*trans*-5-(4-methoxyphenyl)-7-oxo-2-phenylazepane-3,3-dicarboxylate (5a.a''')

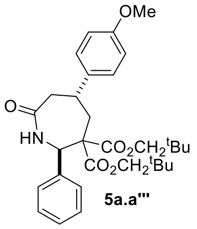
5a.a'''

The title compound was prepared following the **GP5.[a]**, using dineopentyl 2-(4-methoxyphenyl)cyclopropane-1,1-dicarboxylate (**2a'''**) (0.094 g, 0.25 mmol, 1.0 equiv.), benzylidene-1-((*tert*-butyldimethylsilyl)oxy)ethenamine (**4a**) (0.039 g, 0.15 mmol, 1.5 equiv.) and $Yb(OTf)_3$ (31 mg, 0.050 mmol, 20 mol%). Upon column chromatography (Biotage flash chromatographer, 12 g SiO_2; EtOAc in pentane, 5 to 70%), it was obtained (0.055 g, 0.088 mmol, 35% yield; one diastereoisomer) as a powdery, white solid.

Melting point: 250.8 – 252.2 °C

R_f (pentane/EtOAc 6/4) 0.55.

^{1}H NMR (400 MHz, Chloroform-*d*) δ 7.69 – 7.60 (m, 2H, Ar*H*), 7.36 – 7.27 (m, 3H, Ar*H*), 7.19 – 7.13 (m, 2H, Ar*H*), 6.87 (d, *J* = 8.7 Hz, 2H, Ar*H*), 6.23 (d, *J* = 6.1 Hz, 1H, (CO)N*H*), 5.04 (d, *J* = 7.3 Hz, 1H, NC*H*Ar), 4.06 (d, *J* = 10.5 Hz, 1H, C*H2*^{t}Bu), 3.80 (s, 3H, O*Me*), 3.74 (d, *J* = 10.5 Hz, 1H, C*H2*^{t}Bu), 3.42 (d, *J* = 10.4 Hz, 1H, C*H2*^{t}Bu), 3.19 (dd, *J* = 13.9, 12.1 Hz, 1H, C*H2*), 2.97 (d, *J* = 10.4 Hz, 1H, C*H2*^{t}Bu), 2.96 (m, 1H, C*H*), 2.82 (ddd, *J* = 14.2, 2.8, 1.6 Hz, 1H, C*H2*), 2.74 – 2.63 (m, 2H, C*H2*), 0.94 (s, 9H, ^{t}*Bu*), 0.62 (s, 9H, ^{t}*Bu*).

^{13}C NMR (101 MHz, Chloroform-*d*) δ 173.7, 169.4, 168.3, 158.5, 137.6, 136.8, 129.1, 128.7, 128.7, 127.3, 114.2, 75.4, 75.3, 63.4, 62.8, 55.3, 46.7, 43.5, 37.6, 31.2, 30.7, 26.7, 26.2.

HRMS (ESI/QTOF) m/z: $[M + Na]^+$ Calcd for $C_{31}H_{41}NNaO_6^+$ 546.2826; Found 546.2828.

GP6: *General procedure for the $Yb(OTf)_3$-catalyzed (4+3) cycloaddition of DA cyclopropanes 3 with aryl substituted azadienes 2.*

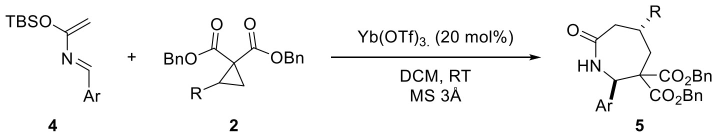

Inside a glove box, a 10 mL, round bottomed vial was charged with $Yb(OTf)_3$ (25 mg, 0.040 mmol, 20 mol%) and molecular sieves 3Å (140-150 mg). The vial was sealed with a PTFE cap, and taken out of the glovebox. DCM (1.2 mL) was added by syringe, and the resulting suspension was stirred at room temperature for 30 minutes. A solution of cyclopropane **2** (0.20 mmol, 1.0 equiv.) and azadiene **4** (0.30 mmol, 1.5 equiv.) in DCM (0.8 mL) was then added. The mixture was stirred at room temperature overnight (18 hours). The solids were then filtered off through a short plug of celite, which was then washed with several portions of DCM/MeOH 9/1. The filtrate was concentrated by evaporation under reduced pressure. Yield was determined upon isolation of the product through column chromatography.

Dibenzyl 2,5-*trans*-5-(4-methoxyphenyl)-7-oxo-2-phenylazepane-3,3-dicarboxylate (5a.a)

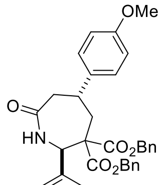
5a.a

The title compound was prepared following the **GP6**, using dibenzyl 2-(4-methoxyphenyl)cyclopropane-1,1-dicarboxylate (**2a**) (0.083 g, 0.20 mmol, 1.0 equiv.) and benzylidene-1-((*tert*-butyldimethylsilyl)oxy)ethenamine (**4a**) (0.078 g, 0.30 mmol, 1.5 equiv.). Upon column chromatography (Biotage flash chromatographer, 4 g SiO_2; EtOAc in pentane, 20 to 50%), it was obtained (0.099 g, 0.18 mmol, 90% yield – d.r. 94 : 6) as a white, foamy solid. Recrystallization from *n*-hexane and EtOAc permitted to obtain a single crystal suitable for X-Ray diffraction analysis, which was used to determine the relative configuration of the compound.

R_f (pentane/EtOAc 6/4) 0.37.

^{1}H NMR (400 MHz, Chloroform-*d*) δ 7.60 – 7.52 (m, 2H, Ar*H*), 7.38 – 7.18 (m, 11H, Ar*H*), 6.97 – 6.92 (m, 2H, Ar*H*), 6.90 – 6.85 (m, 2H, Ar*H*), 6.82 – 6.77 (m, 2H, Ar*H*), 6.17 (d, *J* = 6.9 Hz, 1H, (CO)N*H*), 5.37 (d, *J* = 11.9 Hz, 1H, *CH2*Ph), 5.08 – 5.02 (m, 2H, *CH2*Ph and NC*H*Ar), 4.66 (d, *J* = 12.2 Hz, 1H, *CH2*Ph), 4.41 (d, *J* = 12.3 Hz, 1H, *CH2*Ph), 3.79 (s, 3H, O*Me*), 3.16 (dd, *J* = 13.7, 12.0 Hz, 1H, C*H2*), 2.87 (t, *J* = 12.0 Hz, 1H, C*H*), 2.78 (dt, *J* = 14.1, 2.2 Hz, 1H, C*H2*), 2.66 (dd, *J* = 13.8, 1.5 Hz, 1H, C*H2*), 2.55 (dd, *J* = 14.2, 12.3 Hz, 1H, C*H2*).

^{13}C NMR (101 MHz, Chloroform-*d*; the two benzylic C signals are not revolved from each other) δ 173.8, 169.0, 167.9, 158.4, 137.4, 136.7, 135.0, 134.3, 129.0, 128.8, 128.8, 128.7, 128.7, 128.6, 128.5, 128.4, 128.1, 127.3, 114.1, 67.5, 63.3, 62.6, 55.3, 47.2, 42.7, 37.2.

HRMS (ESI/QTOF) m/z: $[M + Na]^+$ Calcd for $C_{35}H_{33}NNaO_6^+$ 586.2200; Found 586.2204.

Larger scale experiments:

Starting from 1.0 mmol of cyclopropane 2a: Inside a glove-box, a 25 mL round-bottom vial was charged with ytterbium(III) triflate (124 mg, 0.200 mmol, 20 mol%), and activated molecular sieves (3Å; ca. 0.7 mg). The vial was sealed with a septum, and taken out of the glove-box. DCM (6.0 mL) was added by syringe, and the resulting suspension was stirred at room temperature for 30 minutes. In a separate sealed 25 mL, round-bottomed vial, dibenzyl 2-(4-methoxyphenyl)cyclopropane-1,1-dicarboxylate (**2a**) (0.416 g, 1.00 mmol, 1.0 equiv.) was dissolved in DCM (4.0 mL). (*E*)-N-[1-[*tert*-Butyl(dimethyl)silyl]oxyethenyl]-1-phenylmethanimine (**4a**) (0.392 g, 1.50 mmol, 1.5 equiv.) was added by syringe under stirring. The resulting pale yellow, clear solution was transferred into the previously prepared suspension by syringe. A pale yellow mixture was formed, and was stirred at room temperature for 17 hours. After this time, TLC analysis (pentane/EtOAC 6/4) showed (almost) complete conversion, while the mixture looked like a milky suspension. The reaction was quenched by addition of methanol (ca. 2 mL), celite (ca. 10 g) was

added, and the volatiles were distilled off under reduced pressure. The resulting crude product was submitted to column chromatography (Biotage, 25 g SiO_2; MeOH in DCM, 0 to 2 to 5%). Dibenzyl (2,5-*trans*)-5-(4-methoxyphenyl)-7-oxo-2-phenylazepane-3,3-dicarboxylate (**5a.a**) (0.478 g, 0.848 mmol, 85% yield – d.r. 96.5 : 3.5) was collected as a white foam.
The same experiment was reiterated a second time to provide **5a.a** (0.530 g, 0.940 mmol) in 94% yield.
Average yield over two experiments: 89%.

Starting from 1.0 g of cyclopropane 2a: Inside a glove-box, a 50 mL round-bottomed flask was charged with ytterbium(III) triflate (297 mg, 0.480 mmol, 20 mol%), and activated molecular sieves (3Å; ca. 0.7 g). The vial was sealed with a septum, and taken out of the glove-box. DCM (15 mL) was added by syringe, and the resulting suspension was stirred at room temperature for 30 minutes. In a separate sealed 25 mL, round-bottomed vial, dibenzyl 2-(4-methoxyphenyl)cyclopropane-1,1-dicarboxylate (**2a**) (1.0 g, 2.4 mmol, 1.0 equiv.) was dissolved in DCM (10 mL). (*E*)-N-[1-[*tert*-Butyl(dimethyl)silyl]oxyethenyl]-1-phenylmethanimine (**4a**) (0.942 g, 3.60 mmol, 1.5 equiv.) was added by syringe under stirring. The resulting pale yellow, clear solution was transferred into the previously prepared suspension by syringe. A pale yellow mixture was formed, and was stirred at room temperature for 23 hours. After this time, TLC analysis (DCM/MeOH 95/5) showed complete conversion. Celite (ca. 10 g) was added, and the volatiles were distilled off under reduced pressure. The resulting crude product was submitted to column chromatography (dry load; Biotage, 25 g SiO_2; MeOH in DCM, 0 to 12%). Dibenzyl (2,5-*trans*)-5-(4-methoxyphenyl)-7-oxo-2-phenylazepane-3,3-dicarboxylate (**5a.a**) (1.22 g, 2.16 mmol, 90% yield – d.r. 95 : 5) was collected as a white foam.

Dibenzyl 2,5-*trans*-5-(3,4-dimethoxyphenyl)-7-oxo-2-phenylazepane-3,3-dicarboxylate (5a.b)

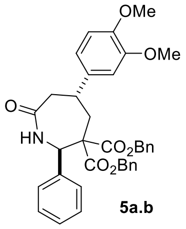

5a.b

The title compound was prepared following the **GP6**, using dibenzyl 2-(3,4-dimethoxyphenyl)cyclopropane-1,1-dicarboxylate (**2b**) (0.089 g, 0.20 mmol, 1.0 equiv.) and benzylidene-1-((*tert*-butyldimethylsilyl)oxy)ethenamine (**4a**) (0.078 g, 0.30 mmol, 1.5 equiv.). Upon column chromatography (Biotage flash chromatographer, 4 g SiO_2; EtOAc in pentane, 20 to 50%), it was obtained (109 mg, 0.184 mmol, 92% yield – d.r. 96 : 4) as a white, foamy solid.

R_f (pentane/EtOAc 6/4) 0.18.
^{1}H NMR (400 MHz, Chloroform-*d*) δ 7.62 – 7.52 (m, 2H, Ar*H*), 7.38 – 7.20 (m, 11H, Ar*H*), 6.92 – 6.85 (m, 2H, Ar*H*), 6.78 (d, *J* = 8.3 Hz, 1H, Ar*H*), 6.61 (dd, *J* = 8.2, 2.1 Hz, 1H, Ar*H*), 6.52 (d, *J* = 2.1 Hz, 1H, Ar*H*), 6.17 (m, 1H, (CO)N*H*), 5.36 (d, *J* = 12.0 Hz, 1H, *CH₂*Ph), 5.08 (d, *J* = 7.2 Hz, 1H, NC*H*Ar), 5.03 (d, *J* = 11.9 Hz, 1H, *CH₂*Ph), 4.68 (d, *J* = 12.2 Hz, 1H, *CH₂*Ph), 4.43 (d, *J* = 12.2 Hz, 1H, *CH₂*Ph), 3.85 (s, 3H, O*Me*), 3.76 (s, 3H, O*Me*), 3.17 (m, 1H, C*H₂*), 2.89 (t, *J* = 12.3 Hz, 1H, C*H*), 2.81 (m, 1H, C*H₂*), 2.70 (m, 1H, C*H₂*), 2.57 (dd, *J* = 14.1, 12.2 Hz, 1H, C*H₂*).
^{13}C NMR (101 MHz, Chloroform-*d*; the signal corresponding to one aromatic C is not resolved) δ 173.8, 169.0, 168.0, 149.0, 147.9, 137.9, 136.6, 135.0, 134.3, 129.0, 128.9, 128.7, 128.7, 128.6, 128.5, 128.4, 128.1, 117.9, 111.3, 109.967.6, 67.5, 63.3, 62.6, 55.9, 55.9, 47.0, 42.7, 37.6.
RMS (ESI/QTOF) m/z: [M + Na]$^+$ Calcd for $C_{36}H_{35}NNaO_7^+$ 616.2306; Found 616.2311.

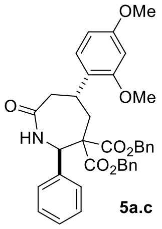

5a.c

Dibenzyl 2,5-*trans*-5-(2,4-dimethoxyphenyl)-7-oxo-2-phenylazepane-3,3-dicarboxylate (5a.c)

The title compound was prepared following the **GP6**, using dibenzyl 2-(2,4-dimethoxyphenyl)cyclopropane-1,1-dicarboxylate (**2c**) (0.089 g, 0.20 mmol, 1.0 equiv.) and benzylidene-1-((*tert*-butyldimethylsilyl)oxy)ethenamine (**4a**) (0.078 g, 0.30 mmol, 1.5 equiv.). Upon column chromatography (Biotage flash chromatographer, 4 g SiO_2; EtOAc in pentane, 15 to 60%), it was obtained (0.096 g, 0.16 mmol, 82% yield – dr 92 : 8) as a white, foamy solid.

R_f (pentane/EtOAc 6/4) = 0.30.
^{1}H NMR (400 MHz, Chloroform-*d*) δ 7.65 – 7.57 (m, 2H, Ar*H*), 7.34 – 7.26 (m, 7H, Ar*H*), 7 7.26 – 7.19 (m, 4H, Ar*H*), 6.97 (d, *J* = 7.9 Hz, 1H, Ar*H*), 6.92 – 6.84 (m, 2H, Ar*H*), 6.46 – 6.42 (m, 2H, Ar*H*), 6.27 (d, *J* = 6.1 Hz, 1H, (CO)N*H*), 5.24 (d, *J* = 12.3 Hz, 1H, *CH₂*Ph), 5.18 (d, *J* = 12.3 Hz, 1H, *CH₂*Ph), 5.12 (d, *J* = 7.1 Hz, 1H, NC*H*Ar), 4.66 (d, *J* = 12.1 Hz, 1H, *CH₂*Ph), 4.40 (d, *J* = 12.1 Hz, 1H, *CH₂*Ph), 3.82 (s, 3H, O*Me*), 3.67 (s, 3H, O*Me*), 3.38 – 3.26 (m, 2H, C*H₂*), 2.83 – 2.69 (m, 2H, C*H* and C*H₂*), 2.63 (m, 1H, C*H₂*).
^{13}C NMR (101 MHz, Chloroform-*d*; the two benzylic C signals are not revolved from each other; the signal corresponding to one aliphatic C is not resolved) δ 174.6, 169.2, 168.2, 159.6, 157.3, 136.8, 135.1, 134.4, 129.0, 128.8, 128.6, 128.5, 128.4, 128.3, 128.3, 128.3, 128.1, 127.4, 125.7, 104.1, 98.8, 67.4, 63.4, 55.4, 55.2, 44.8, 41.7.
HRMS (APCI/QTOF) m/z: [M + H]$^+$ Calcd for $C_{36}H_{36}NO_7^+$ 594.2486; Found 594.2489.

Dibenzyl 2,5-*trans*-5-(2-methoxyphenyl)-7-oxo-2-phenylazepane-3,3-dicarboxylate (5a.d)

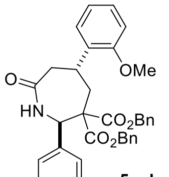

5a.d

The title compound was prepared following the **GP6**, but on a 0.1 mmol scale, using dibenzyl 2-(2-methoxyphenyl)cyclopropane-1,1-dicarboxylate (**2d**) (0.042 g, 0.10 mmol, 1.0 equiv.), benzylidene-1-((*tert*-butyldimethylsilyl)oxy)ethenamine (**4a**) (0.039 g, 0.15 mmol, 1.5 equiv.) and $Yb(OTf)_3$ (13 mg, 0.020 mmol, 20 mol%) with 65 mg MS 3Å. Upon column chromatography (Biotage flash chromatographer, 4 g SiO_2; EtOAc in pentane, 10 to 45%), it was obtained (0.031 g, 0.055 mmol, 55% yield – d.r. > 95 : 5) as a white, foamy solid. Recovered starting material: 0.010, 0.021 mmol, 21%.

R_f (pentane/EtOAc 6/4) 0.30.
^{1}H NMR (400 MHz, Chloroform-*d*) δ 7.60 – 7.55 (m, 2H, Ar*H*), 7.29 – 7.27 (m, 7H, Ar*H*), 7.25 – 7.17 (m, 5H, Ar*H*), 7.06 (d, *J* = 7.7 Hz, 1H, Ar*H*), 6.90 (m, 1H, Ar*H*), 6.87 – 6.81 (m, 3H, Ar*H*), 6.17 (d, *J* = 6.1 Hz, 1H, (CO)N*H*), 5.22 (d, *J* = 12.4 Hz, 1H, CH_2Ph), 5.17 (d, *J* = 12.2 Hz, 1H, CH_2Ph), 5.11 (d, *J* = 7.2 Hz, 1H, NC*H*Ar), 4.63 (d, *J* = 12.2 Hz, 1H, CH_2Ph), 4.38 (d, *J* = 12.2 Hz, 1H, CH_2Ph), 3.68 (s, 3H, O*Me*), 3.40 (t, *J* = 11.1 Hz, 1H, C*H* or CH_2), 3.32 (t, *J* = 12.6 Hz, 1H, C*H* or CH_2), 2.83 – 2.67 (m, 2H, C*H* or CH_2), 2.61 (d, *J* = 12.5 Hz, 1H, C*H* or CH_2).
^{13}C NMR (101 MHz, Chloroform-*d*; the signals corresponding to one aromatic C and one aliphatic C are not resolved) δ 174.4, 169.2, 168.3, 156.3, 136.9, 135.1, 134.4, 133.1, 129.0, 128.8, 128.6, 128.5, 128.4, 128.3, 128.3, 128.1, 127.9, 127.0, 120.7, 110.7, 67.4, 63.3, 62.6, 55.2, 44.6, 41.6, 32.1 (br s), .
HRMS (ESI/QTOF) m/z: $[M + H]^+$ Calcd for $C_{35}H_{34}NO_6^+$ 564.2381; Found 564.2369.

Dibenzyl 2,5-*trans*-5-(3-iodo-4-methoxyphenyl)-7-oxo-2-phenylazepane-3,3-dicarboxylate (5a.e)

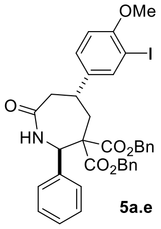

5a.e

The title compound was prepared following the **GP6**, using dibenzyl 2-(3-iodo-4-methoxyphenyl)cyclopropane-1,1-dicarboxylate (**2e**) (0.108 g, 0.200 mmol, 1.0 equiv.) and benzylidene-1-((*tert*-butyldimethylsilyl)oxy)ethenamine (**4a**) (0.078 g, 0.30 mmol, 1.5 equiv.). Upon column chromatography (Biotage flash chromatographer, 4 g SiO_2; EtOAc in pentane, 15 to 50%), it was obtained (0.091 g, 0.13 mmol, 66% yield – d.r. 95 : 5) as a white, foamy solid.

R_f (pentane/EtOAc 6/4) 0.29.
^{1}H NMR (400 MHz, Chloroform-*d*) δ 7.56 (dd, *J* = 7.6, 2.0 Hz, 1H, Ar*H*), 7.44 (d, *J* = 2.3 Hz, 1H, Ar*H*), 7.40 – 7.33 (m, 2H, Ar*H*), 7.33 – 7.18 (m, 10H, Ar*H*), 6.96 (dd, *J* = 8.5, 2.4 Hz, 1H, Ar*H*), 6.91 – 6.85 (m, 2H, Ar*H*), 6.71 (d, *J* = 8.5 Hz, 1H, Ar*H*), 6.15 (d, *J* = 6.4 Hz, 1H, (CO)N*H*), 5.41 (d, *J* = 11.9 Hz, 1H, CH_2Ph), 5.04 (d, *J* = 6.9 Hz, 1H, NC*H*Ar), 5.00 (d, *J* = 11.9 Hz, 1H, CH_2Ph), 4.68 (d, *J* = 12.2 Hz, 1H, CH_2Ph), 4.41 (d, *J* = 12.2 Hz, 1H, CH_2Ph), 3.86 (s, 3H, O*Me*), 3.13 (m, 1H, CH_2), 2.78 (dd, *J* = 19.8, 12.8 Hz, 2H, C*H* and CH_2), 2.62 (d, *J* = 12.3 Hz, 1H, CH_2), 2.53 (m, 1H, CH_2).
^{13}C NMR (101 MHz, Chloroform-*d*; the signals corresponding to two aromatic C are not resolved) δ 173.4, 168.9, 167.8, 157.0, 139.4, 137.3, 136.6, 134.9, 134.3, 129.0, 128.9, 128.9, 128.8, 128.7, 128.5, 128.4, 128.1, 127.3, 111.0, 86.2, 67.6, 63.2, 62.6, 56.4, 46.9, 42.6, 36.7.
HRMS (ESI/QTOF) m/z: $[M + Na]^+$ Calcd for $C_{35}H_{32}INNaO_6^+$ 712.1167; Found 712.1164.

Dibenzyl 2,5-*trans*-7-oxo-2-phenyl-5-(*p*-tolyl)azepane-3,3-dicarboxylate (5a.f)

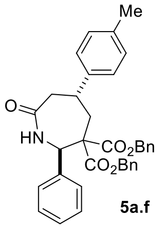

5a.f

The title compound was prepared following the **GP6**, using dibenzyl 2-(*p*-tolyl)cyclopropane-1,1-dicarboxylate (**2f**) (0.080 g, 0.20 mmol, 1.0 equiv.) and benzylidene-1-((*tert*-butyldimethylsilyl)oxy)ethenamine (**4a**) (0.078 g, 0.30 mmol, 1.5 equiv.). Upon column chromatography (Biotage flash chromatographer, 4 g SiO_2; EtOAc in pentane, 10 to 45%), it was obtained (0.031 g, 0.57 mmol, 28% yield – d.r. > 95 : 5) as a white, foamy solid. Recovered starting material: 0.034 g, 0.062 mmol, 31%.

R_f (pentane/EtOAc 6/4) 0.50.
^{1}H NMR (400 MHz, Chloroform-*d*) δ 7.59 – 7.54 (m, 2H, Ar*H*), 7.38 – 7.30 (m, 2H, Ar*H*), 7.30 – 7.19 (m, 9H, Ar*H*), 7.08 (d, *J* = 7.7 Hz, 2H, Ar*H*), 6.94 – 6.89 (m, 2H, Ar*H*), 6.89 – 6.85 (m, 2H, Ar*H*), 6.16 (d, *J* = 7.0 Hz, 1H, (CO)N*H*), 5.36 (d, *J* = 12.0 Hz, 1H, CH_2Ph), 5.09 – 5.01 (m, 2H, CH_2Ph and NC*H*Ar), 4.66 (d, *J* = 12.3 Hz, 1H, CH_2Ph), 4.41 (d, *J* = 12.2 Hz, 1H, CH_2Ph), 3.18 (dd, *J* = 13.7, 12.2 Hz, 1H, CH_2), 2.88 (t, *J* = 11.8 Hz, 1H, C*H*), 2.79 (dt, *J* = 14.1, 2.1 Hz, 1H, CH_2), 2.67 (dd, *J* = 13.8, 1.6 Hz, 1H, CH_2), 2.56 (dd, *J* = 14.1, 12.4 Hz, 1H, CH_2), 2.31 (s, 3H, Ar*Me*).
^{13}C NMR (101 MHz, Chloroform-*d*) δ 173.8, 169.0, 168.0, 142.2, 136.7, 136.5, 135.0, 134.4, 129.4, 129.0, 128.8, 128.8, 128.7, 128.7, 128.6, 128.5, 128.4, 128.1, 126.2, 67.5, 67.5, 63.3, 62.7, 47.1, 42.5, 37.6, 21.0.
HRMS (ESI/QTOF) m/z: $[M + Na]^+$ Calcd for $C_{35}H_{33}NNaO_5^+$ 570.2251; Found 570.2270.

Dibenzyl 2,5-*trans*-7-oxo-2-phenyl-5-(1-tosyl-1H-indol-3-yl)azepane-3,3-dicarboxylate (5a.g)

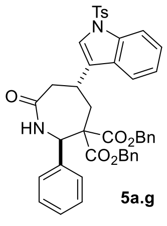

5a.g

The title compound was prepared following the **GP6**, using dibenzyl 2-(1-tosyl-1H-indol-3-yl)cyclopropane-1,1-dicarboxylate (**2g**) (0.12 g, 0.20 mmol, 1.0 equiv.) and benzylidene-1-((*tert*-butyldimethylsilyl)oxy)ethenamine (**4a**) (0.078 g, 0.30 mmol, 1.5 equiv.). Upon column chromatography (Biotage flash chromatographer, 4 g SiO_2; EtOAc in pentane, 15 to 55%), it was obtained (0.088 g, 0.12 mmol, 61% yield – d.r. 92 : 8) as a white, foamy solid.

R_f (pentane/EtOAc 6/4) 0.25.

^{1}H NMR (400 MHz, Chloroform-*d*) δ 7.95 (d, *J* = 8.3 Hz, 1H, Ar*H*), 7.79 – 7.68 (m, 2H, Ar*H*), 7.63 – 7.49 (m, 2H, Ar*H*), 7.39 – 7.15 (m, 16H, Ar*H*), 6.98 (t, *J* = 7.6 Hz, 1H, Ar*H*), 6.88 – 6.78 (m, 2H, Ar*H*), 6.22 (d, *J* = 7.0 Hz, 1H, (CO)N*H*), 5.27 (d, *J* = 11.8 Hz, 1H, *CH₂*Ph), 5.18 – 4.99 (m, 2H, *CH₂*Ph and NC*H*Ar), 4.58 (d, *J* = 12.6 Hz, 1H, *CH₂*Ph), 4.42 (d, *J* = 12.0 Hz, 1H, *CH₂*Ph), 3.29 – 3.16 (m, 2H, *CH* or C*H₂*), 3.03 (d, *J* = 14.5 Hz, 1H, *CH* or C*H₂*), 2.85 (d, *J* = 11.8 Hz, 1H, *CH* or C*H₂*), 2.49 (m, 1H, *CH* or C*H₂*), 2.34 (s, 3H, SO_2Ph-*Me*).

^{13}C NMR (101 MHz, Chloroform-*d*; the signal corresponding to one aliphatic C is not resolved) δ 173.3, 168.8, 168.1, 145.0, 136.5, 135.2, 135.1, 134.6, 134.2, 130.0, 129.0, 129.0, 129.0, 128.9, 128.8, 128.8, 128.7, 128.5, 128.5, 128.3, 126.8, 126.3, 125.0, 123.3, 121.6, 119.6, 113.7, 68.1, 67.6, 63.2, 62.6, 41.2 (br s), 29.3, 21.6.

IR (ν_{max}, cm^{-1}) 3034 (w), 2925 (w), 1725 (s), 1597 (w), 1496 (w), 1450 (m), 1373 (s), 1318 (s), 1271 (s), 1175 (s), 1132 (s), 1100 (m), 982 (m), 907 (w), 813 (w), 745 (s).

HRMS (ESI/QTOF) m/z: [M + H]$^+$ Calcd for $C_{43}H_{39}N_2O_7S^+$ 727.2472; Found 727.2468.

Dibenzyl 2,5-*trans*-5-(benzo[b]thiophen-2-yl)-7-oxo-2-phenylazepane-3,3-dicarboxylate (5a.h)

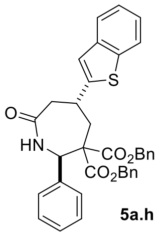

5a.h

The title compound was prepared following the **GP6**, using dibenzyl 2-(benzo[b]thiophen-2-yl)cyclopropane-1,1-dicarboxylate (**2h**) (0.088 g, 0.20 mmol, 1.0 equiv.) and benzylidene-1-((*tert*-butyldimethylsilyl)oxy)ethenamine (**4a**) (0.078 g, 0.30 mmol, 1.5 equiv.). Upon column chromatography (Biotage flash chromatographer, 4 g SiO_2; EtOAc in pentane, 15 to 55%), it was obtained (0.066 g, 0.11 mmol, 56% yield – d.r. 93 : 7) as a white, foamy solid.

R_f (pentane/EtOAc 6/4) 0.70.

^{1}H NMR (400 MHz, Chloroform-*d*) δ 7.78 (dd, *J* = 7.9, 1.2 Hz, 1H, Ar*H*), 7.67 (m, 1H, Ar*H*), 7.60 – 7.51 (m, 2H, Ar*H*), 7.41 – 7.18 (m, 13H, Ar*H*), 6.92 (s, 1H, SC=C*H*), 6.90 – 6.83 (m, 2H, Ar*H*), 6.25 (d, *J* = 6.1 Hz, 1H, (CO)N*H*), 5.33 (d, *J* = 12.0 Hz, 1H, *CH₂*Ph), 5.10 (d, *J* = 7.0 Hz, 1H, NC*H*Ar), 5.04 (d, *J* = 11.9 Hz, 1H, *CH₂*Ph), 4.63 (d, *J* = 12.2 Hz, 1H, *CH₂*Ph) , 4.44 (d, *J* = 12.2 Hz, 1H, *CH₂*Ph), 3.36 (m, 1H, C*H*), 3.23 (dd, *J* = 13.7, 12.0 Hz, 1H, C*H₂*), 3.05 (ddd, *J* = 14.1, 2.9, 1.5 Hz, 1H, C*H₂*), 2.91 (dt, *J* = 13.6, 1.7 Hz, 1H, C*H₂*), 2.72 (dd, *J* = 14.1, 11.6 Hz, 1H, C*H₂*).

^{13}C NMR (101 MHz, Chloroform-*d*; the signals corresponding to two aromatic C are not resolved) δ 172.7, 168.7, 167.7, 148.8, 139.5, 138.8, 136.4, 134.8, 134.2, 128.9, 128.8, 128.7, 128.7, 128.7, 128.5, 128.4, 128.1, 124.4, 123.3, 122.3, 119.8, 67.7, 67.6, 63.1, 62.5, 46.6, 43.2, 34.2.

HRMS (ESI/QTOF) m/z: [M + Na]$^+$ Calcd for $C_{36}H_{31}NNaO_5S^+$ 612.1815; Found 612.1838.

Dibenzyl 2,5-*trans* -7-oxo-2-phenyl-5-(1-phenyl-1*H*-pyrazol-4-yl)azepane-3,3-dicarboxylate (5a.i)

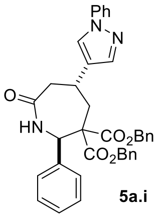

5a.i

The title compound was prepared following the **GP6**, using dibenzyl 2-(1-phenyl-1*H*-pyrazol-4-yl)cyclopropane-1,1-dicarboxylate (**2i**) (0.091 g, 0.20 mmol, 1.0 equiv.) and benzylidene-1-((*tert*-butyldimethylsilyl)oxy)ethenamine (**4a**) (0.078 g, 0.30 mmol, 1.5 equiv.). Upon column chromatography (Biotage flash chromatographer, 4 g SiO_2; EtOAc in pentane, 15 to 55%), it was obtained (0.079 g, 0.13 mmol, 66% yield – d.r. 94 : 6) as a white, foamy solid.

R_f (pentane/EtOAc 6/4) 0.30.

^{1}H NMR (400 MHz, Chloroform-*d*) δ 7.66 – 7.60 (m, 3H, Ar*H*), 7.57 – 7.52 (m, 2H, Ar*H*), 7.50 (d, *J* = 0.8 Hz, 1H, $Ar_{pyrazole}$H), 7.48 – 7.40 (m, 2H, Ar*H*), 7.39 – 7.17 (m, 12H, Ar*H*), 6.93 – 6.85 (m, 2H, Ar*H*), 6.16 (d, *J* = 6.0 Hz, 1H, (CO)N*H*), 5.33 (d, *J* = 11.9 Hz, 1H, *CH₂*Ph), 5.09 (d, *J* = 7.2 Hz, 1H, NC*H*$Ar_{pyrazole}$), 4.99 (d, *J* = 12.0 Hz, 1H, *CH₂*Ph), 4.66 (d, *J* = 12.0 Hz, 1H, *CH₂*Ph), 4.45 (d, *J* = 12.2 Hz, 1H, *CH₂*Ph), 3.12 (t, *J* = 11.9 Hz, 1H, C*H₂*), 3.03 (m, 1H, C*H*), 2.94 (ddd, *J* = 13.9, 2.7, 1.5 Hz, 1H, C*H₂*), 2.77 (m, 1H, C*H₂*), 2.58 (dd, *J* = 14.1, 11.4 Hz, 1H, C*H₂*).

^{13}C NMR (101 MHz, Chloroform-*d*; the signal corresponding to one aromatic C is not resolved) δ 173.2, 169.0, 167.8, 140.0, 138.9, 136.6, 134.9, 134.3, 129.5, 128.9, 128.9, 128.8, 128.7, 128.7, 128.5, 128.4, 128.2, 127.4, 126.5, 123.8, 119.0, 67.6, 67.6, 63.1, 62.4, 46.0, 43.0, 28.6.

IR (ν_{max}, cm^{-1}) 3405 (w), 3251 (w), 3065 (w), 3034 (w), 2952 (w), 1718 (s), 1663 (s), 1600 (m), 1501 (m), 1451 (m), 1399 (m), 1227 (s), 1155 (s), 1075 (m), 1033 (w), 954 (m), 909 (w), 738 (s).

HRMS (ESI/QTOF) m/z: [M + H]$^+$ Calcd for $C_{37}H_{34}N_3O_5^+$ 600.2493; Found 600.2496.

Dibenzyl 2,5-*trans*-7-oxo-2-phenyl-5-((E)-1-phenylprop-1-en-2-yl)azepane-3,3-dicarboxylate (5a.j)

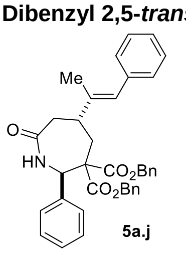

5a.j

The title compound was prepared following the **GP6**, using dibenzyl (*E*)-2-(1-phenylprop-1-en-2-yl)cyclopropane-1,1-dicarboxylate (**2j**) (0.085 g, 0.20 mmol, 1.0 equiv.) and benzylidene-1-((*tert*-butyldimethylsilyl)oxy)ethenamine (**4a**) (0.078 g, 0.30 mmol, 1.5 equiv.). Upon column chromatography (Biotage flash chromatographer, 4 g SiO_2; EtOAc in pentane, 20 to 50%), it was obtained (60 mg, 0.10 mmol, 52% yield – d.r. > 95 : 5) as a white, foamy solid. Recovered starting material: 0.020 g, 0.046 mmol, 23%.

R_f (pentane/EtOAc 6/4) 0.65.

^{1}H NMR (400 MHz, Chloroform-*d*) δ 7.49 – 7.42 (m, 2H, Ar*H*), 7.28 – 7.08 (m, 14H, Ar*H*), 7.08 – 7.02 (m, 2H, Ar*H*), 6.80 (dd, *J* = 7.9, 1.6 Hz, 2H, Ar*H*), 6.10 – 6.01 (m, 2H, C=C*H*Ph and (CO)N*H*), 5.24 (d, *J* = 11.9 Hz, 1H, *CH₂*Ph), 4.95 – 4.87 (m, 2H, *CH₂*Ph PhC*H*N), 4.62 (d, *J* = 12.2 Hz, 1H, *CH₂*Ph), 4.31 (d, *J* = 12.2 Hz, 1H, *CH₂*Ph), 2.93 (dd, *J* = 13.7, 11.7 Hz,

1H, C*H₂*), 2.67 (dt, *J* = 13.6, 1.9 Hz, 1H, C*H₂*), 2.50 (dq, *J* = 13.8, 1.7 Hz, 1H, C*H₂*), 2.40 (dd, *J* = 13.7, 12.0 Hz, 1H, C*H₂*), 2.30 (t, *J* = 11.9 Hz, 1H, C=CC*H*), 1.67 (d, *J* = 1.4 Hz, 3H, C=C*Me*).
^{13}C NMR (101 MHz, Chloroform-*d*; the signals corresponding to the aromatic C are not completely resolved; the two benzylic C signals are not revolved from each other) δ 174.2, 169.1, 168.0, 141.0, 137.5, 136.7, 134.9, 134.4, 129.0, 128.9, 128.8, 128.7, 128.6, 128.5, 128.4, 128.1, 126.4, 125.5, 67.5, 63.1, 62.7, 44.3, 41.2, 25.7, 15.4.
HRMS (ESI/QTOF) m/z: $[M + Na]^+$ Calcd for $C_{35}H_{33}NNaO_5^+$ 570.2251; Found 570.2270.

Dibenzyl 2,5-*trans*-7-oxo-2-phenyl-5-((E)-styryl)azepane-3,3-dicarboxylate (5a.k)

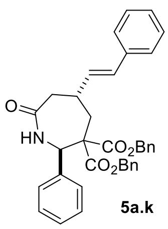
5a.k

The title compound was prepared following the **GP6**, using dibenzyl (*E*)-2-styrylcyclopropane-1,1-dicarboxylate (**2k**) (0.082 g, 0.20 mmol, 1.0 equiv.) and benzylidene-1-((*tert*-butyldimethylsilyl)oxy)ethenamine (**4a**) (0.078 g, 0.30 mmol, 1.5 equiv.). Upon column chromatography (Biotage flash chromatographer, 4 g SiO_2; EtOAc in pentane, 15 to 50%), it was obtained (0.077 mg, 0.14 mmol, 69% yield – d.r. 90 : 10) as a white, foamy solid.

R_f (pentane/EtOAc 6/4) 0.45.
^{1}H NMR (400 MHz, Chloroform-*d*) δ 7.57 – 7.50 (m, 2H, Ar*H*), 7.38 – 7.11 (m, 16H, Ar*H*), 6.93 – 6.85 (m, 2H, Ar*H*), 6.28 (d, *J* = 16.1 Hz, 1H, C*H*=C), 6.15 – 6.06 (m, 2H, C*H*=C and (CO)N*H*), 5.29 (d, *J* = 12.0 Hz, 1H, *CH₂*Ph), 5.03 (d, *J* = 6.9 Hz, 1H, PhC*H*N), 4.99 (d, *J* = 12.0 Hz, 1H, *CH₂*Ph), 4.68 (d, *J* = 12.2 Hz, 1H, *CH₂*Ph), 4.47 (d, *J* = 12.2 Hz, 1H, *CH₂*Ph), 2.90 (dd, *J* = 13.9, 11.9 Hz, 1H, C*H₂*), 2.80 (m, 1H, C*H₂*), 2.65 – 2.53 (m, 2H, C=CC*H* and C*H₂*), 2.41 (dd, *J* = 13.9, 11.3 Hz, 1H, C*H₂*).
^{13}C NMR (101 MHz, Chloroform-*d*; the signals corresponding to two aromatic C are not resolved) δ 173.5, 169.2, 168.0, 136.8, 136.7, 134.9, 134.3, 132.5, 129.8, 128.8, 128.7, 128.6, 128.6, 128.5, 128.4, 127.6, 126.2, 67.6, 67.5, 62.8, 62.3, 44.3, 41.5, 35.2.
HRMS (ESI/QTOF) m/z: $[M + H]^+$ Calcd for $C_{36}H_{34}NO_5^+$ 560.2431; Found 560.2449.

Dibenzyl 2,5-*trans*-5-(4-methoxyphenyl)-7-oxo-2-(4-(trifluoromethyl)phenyl)azepane-3,3-dicarboxylate (5b.a)

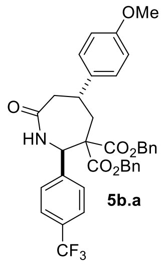
5b.a

The title compound was prepared following the **GP6**, using dibenzyl 2-(4-methoxyphenyl)cyclopropane-1,1-dicarboxylate (**2a**) (0.083 g, 0.20 mmol, 1.0 equiv.) and N-(1-((*tert*-Butyldimethylsilyl)oxy)vinyl)-1-(4-(trifluoromethyl)phenyl)methanimine (**4b**) (0.098 g, 0.30 mmol, 1.5 equiv.). Upon column chromatography (Biotage flash chromatographer, 4 g SiO_2; EtOAc in pentane, 15 to 60%), it was obtained (0.105 g, 0.166 mmol, 83% yield – dr 99 : 1) as a white, foamy solid.

R_f (pentane/EtOAc 6/4) = 0.56.
^{1}H NMR (400 MHz, Chloroform-*d*) δ 7.69 – 7.62 (m, 2H, Ar*H*), 7.49 – 7.43 (m, 2H, Ar*H*), 7.41 – 7.32 (m, 3H, Ar*H*), 7.31 – 7.25 (m, 3H, Ar*H*), 7.26 – 7.18 (m, 2H, Ar*H*), 6.97 – 6.92 (m, 2H, Ar*H*), 6.89 – 6.84 (m, 2H, Ar*H*), 6.84 – 6.77 (m, 2H, Ar*H*), 6.09 (d, *J* = 7.2 Hz, 1H, (CO)N*H*), 5.38 (d, *J* = 11.8 Hz, 1H, *CH₂*Ph), 5.13 (d, *J* = 7.2 Hz, 1H, NC*H*Ar), 5.09 (d, *J* = 11.9 Hz, 1H, *CH₂*Ph), 4.69 (d, *J* = 11.9 Hz, 1H, *CH₂*Ph), 4.53 (d, *J* = 12.0 Hz, 1H, *CH₂*Ph), 3.79 (s, 3H, O*Me*), 3.16 (m, 1H, C*H₂*), 2.89 (t, *J* = 12.2 Hz, 1H, C*H*), 2.81 (dt, *J* = 14.1, 2.1 Hz, 1H, C*H₂*), 2.68 (dd, *J* = 13.9, 1.7 Hz, 1H, C*H₂*), 2.53 (dd, *J* = 14.2, 12.2 Hz, 1H, C*H₂*).
^{13}C NMR (101 MHz, Chloroform-*d*) δ 173.8, 168.8, 167.8, 158.5, 140.3, 137.1, 134.8, 133.9, 130.9 (q, *J* = 33.7 Hz), 129.9, 129.6, 128.9, 128.8, 128.7, 128.5, 128.3, 127.3, 125.4 (q, *J* = 3.6 Hz), 123.7 (q, *J* = 272.5 Hz), 114.2, 67.8, 67.7, 63.1, 62.1, 55.3, 47.3, 42.6, 37.2.
^{19}F NMR (376 MHz, Chloroform-*d*) δ -62.7.
HRMS (ESI/QTOF) m/z: $[M + Na]^+$ Calcd for $C_{36}H_{32}F_3NNaO_6^+$ 654.2074; Found 654.2068.

Dibenzyl 2,5-*trans*-2-(4-chlorophenyl)-5-(4-methoxyphenyl)-7-oxoazepane-3,3-dicarboxylate (5c.a)

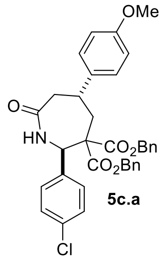
5c.a

The title compound was prepared following the **GP6**, using dibenzyl 2-(4-methoxyphenyl)cyclopropane-1,1-dicarboxylate (**2a**) (0.083 g, 0.20 mmol, 1.0 equiv.) and N-(1-((*tert*-Butyldimethylsilyl)oxy)vinyl)-1-(4-chlorophenyl)methanimine (**4c**) (0.089 g, 0.30 mmol, 1.5 equiv.). Upon column chromatography (Biotage flash chromatographer, 4 g SiO_2; EtOAc in pentane, 15 to 60%), it was obtained (0.108 g, 0.181 mmol, 90% yield – dr 98 : 2) as a white, foamy solid.

R_f (pentane/EtOAc 6/4) = 0.55.
^{1}H NMR (400 MHz, Chloroform-*d*) δ 7.51 – 7.44 (m, 2H, Ar*H*), 7.40 – 7.32 (m, 2H, Ar*H*), 7.33 – 7.20 (m, 6H, Ar*H*), 7.19 – 7.11 (m, 2H, Ar*H*), 6.97 – 6.91 (m, 2H, Ar*H*), 6.91 – 6.86 (m, 2H, Ar*H*), 6.83 – 6.77 (m, 2H, Ar*H*), 6.06 (d, *J* = 6.7 Hz, 1H, (CO)N*H*), 5.38 (d, *J* = 11.9 Hz, 1H, *CH₂*Ph), 5.08 (d, *J* = 12.0 Hz, 1H, *CH₂*Ph), 5.02 (d, *J* = 7.1 Hz, 1H, NC*H*Ar), 4.68 (d, *J* = 12.0 Hz, 1H, *CH₂*Ph), 4.58 (d, *J* = 12.0 Hz, 1H, *CH₂*Ph), 3.79 (s, 3H, O*Me*), 3.14 (dd, *J* = 13.6, 12.3 Hz, 1H, C*H₂*), 2.86 (t, *J* = 12.0 Hz, 1H, C*H*), 2.78 (m, 1H, C*H₂*), 2.66 (d, *J* = 12.6 Hz, 1H, C*H₂*), 2.51 (dd, *J* = 14.2, 12.3 Hz, 1H, C*H₂*).
^{13}C NMR (101 MHz, Chloroform-*d*; the signal corresponding to one aromatic C is not resolved; the two benzylic C signals are not resolved from each other) δ 173.9, 168.9, 167.9, 158.5, 137.2, 135.0, 134.8, 134.8, 134.1, 130.5, 128.9, 128.8, 128.7, 128.6, 128.5, 128.3, 127.3, 114.1, 67.7, 63.2, 62.0, 55.3, 47.2, 42.6, 37.1.
HRMS (nanochip-ESI/LTQ-Orbitrap) m/z: $[M + Na]^+$ Calcd for $C_{35}H_{32}ClNNaO_6^+$ 620.1810; 622.1767; Found 620.1804; 622.1768.

Dibenzyl 2,5-*trans*-2-(2-fluorophenyl)-5-(4-methoxyphenyl)-7-oxoazepane-3,3-dicarboxylate (5d.a)

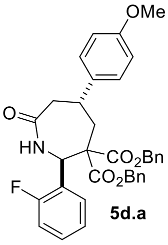

5d.a

The title compound was prepared following the **GP6**, using dibenzyl 2-(4-methoxyphenyl)cyclopropane-1,1-dicarboxylate (**2a**) (0.083 g, 0.20 mmol, 1.0 equiv.) and N-(1-((*tert*-butyldimethylsilyl)oxy)vinyl)-1-(2-fluorophenyl)methanimine (**4d**) (0.084 g, 0.30 mmol, 1.5 equiv.). Upon column chromatography (Biotage flash chromatographer, 4 g SiO_2; EtOAc in pentane, 15 to 60%), it was obtained (0.116 g, 0.199 mmol, quantitative – dr 94 : 6) as a white, foamy solid.

R_f (pentane/EtOAc 6/4) = 0.50.
^{1}H NMR (400 MHz, Chloroform-*d*) δ 8.00 (td, *J* = 7.7, 1.7 Hz, 1H, Ar*H*), 7.39 – 7.30 (m, 5H, Ar*H*), 7.30 – 7.17 (m, 4H, Ar*H*), 7.06 – 6.97 (m, 2H, Ar*H*), 6.97 – 6.89 (m, 4H, Ar*H*), 6.80 (s, 1H, Ar*H*), 5.98 (d, *J* = 7.5 Hz, 1H, (CO)N*H*), 5.58 (d, *J* = 7.5 Hz, 1H, NC*H*Ar), 5.44 (d, *J* = 12.0 Hz, 1H, C*H*$_2$Ph), 5.11 (d, *J* = 12.0 Hz, 1H, C*H*$_2$Ph), 4.71 (d, *J* = 12.1 Hz, 1H, C*H*$_2$Ph), 4.58 (d, *J* = 12.1 Hz, 1H, C*H*$_2$Ph), 3.78 (s, 3H, O*Me*), 3.23 (t, *J* = 13.0 Hz, 1H, C*H*$_2$), 2.88 (t, *J* = 12.2 Hz, 1H, C*H*), 2.78 (m, 1H, C*H*$_2$), 2.65 (d, *J* = 13.8 Hz, 1H, C*H*$_2$), 2.51 (dd, *J* = 14.1, 12.3 Hz, 1H, C*H*$_2$).
^{13}C NMR (101 MHz, Chloroform-*d*; the signals corresponding to two aromatic C are not resolved) δ 173.9, 168.8, 168.08, 159.9 (d, *J* = 248.7 Hz), 158.4, 137.3, 134.9, 134.2, 130.6 (d, *J* = 8.8 Hz), 129.7, 128.8, 128.8, 128.5, 128.5, 128.3, 124.7 (d, *J* = 3.7 Hz), 124.3 (d, *J* = 12.1 Hz), 115.5 (d, *J* = 23.4 Hz), 114.1, 67.71, 67.64, 62.82, 55.30, 53.15 (d, *J* = 6.0 Hz), 47.36, 42.46, 37.44.
^{19}F NMR (376 MHz, Chloroform-*d*; the signal that could be assigned to the *minor isomer* is underlined) δ <u>-113.2</u>, -115.4.
HRMS (ESI/QTOF) m/z: [M + Na]$^+$ Calcd for $C_{35}H_{32}FNNaO_6^+$ 604.2106; Found 604.2125.

Dibenzyl 2,5-*trans*-2-(3-bromophenyl)-5-(4-methoxyphenyl)-7-oxoazepane-3,3-dicarboxylate (5e.a)

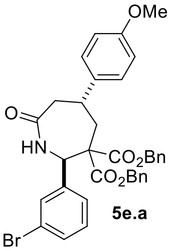

5e.a

The title compound was prepared following the **GP6**, using dibenzyl 2-(4-methoxyphenyl)cyclopropane-1,1-dicarboxylate (**2a**) (0.083 g, 0.20 mmol, 1.0 equiv.) and 1-(2-bromophenyl)-N-(1-((tert-butyldimethylsilyl)oxy)vinyl)methanimine (**4e**) (0.102 g, 0.300 mmol, 1.5 equiv.). Upon column chromatography (Biotage flash chromatographer, 4 g SiO_2; EtOAc in pentane, 15 to 55%), it was obtained (0.100 g, 0.155 mmol, 78% yield; d.r. 98 : 2) as a white, foamy solid.

R_f (pentane/EtOAc 6/4): 0.43.
^{1}H NMR (400 MHz, Chloroform-*d*) δ 7.76 (s, 1H, Ar*H*), 7.51 (d, *J* = 7.9 Hz, 1H, Ar*H*), 7.40 (d, *J* = 8.7 Hz, 1H, Ar*H*), 7.38 – 7.31 (m, 3H, Ar*H*), 7.32 – 7.23 (m, 7H, Ar*H*), 7.10 (t, *J* = 7.9 Hz, 1H, Ar*H*), 6.92 – 6.91 (m, 2H, Ar*H*), 6.84 – 6.76 (m, 2H, Ar*H*), 6.11 (d, *J* = 7.0 Hz, 1H, (CO)N*H*), 5.39 (dd, *J* = 12.0, 2.6 Hz, 1H, C*H*$_2$Ph), 5.07 (d, *J* = 12.1 Hz, 1H, C*H*$_2$Ph), 5.01 (d, *J* = 7.0 Hz, 1H, NC*H*Ar), 4.66 (d, *J* = 12.3 Hz, 1H, C*H*$_2$Ph), 4.55 (d, *J* = 12.1 Hz, 1H, C*H*$_2$Ph), 3.79 (d, *J* = 2.5 Hz, 3H, O*Me*), 3.14 (t, *J* = 13.0 Hz, 1H, C*H*$_2$), 2.85 (t, *J* = 12.2 Hz, 1H, C*H*), 2.77 (d, *J* = 14.3 Hz, 1H, C*H*$_2$), 2.66 (d, *J* = 13.8 Hz, 1H, C*H*$_2$), 2.50 (t, *J* = 13.3 Hz, 1H, C*H*$_2$).
^{13}C NMR (101 MHz, Chloroform-*d*) δ 173.7, 168.8, 167.8, 158.4, 138.8, 137.2, 134.9, 134.1, 132.2, 132.0, 130.2, 128.9, 128.8, 128.7, 128.6, 128.5, 128.2, 127.7, 127.3, 122.5, 114.1, 67.7, 67.7, 63.1, 62.1, 55.3, 47.2, 42.6, 37.1.
HRMS (ESI/QTOF) m/z: [M + H]$^+$ Calcd for $C_{35}H_{33}BrNO_6^+$ 642.1486, 644.1472; Found 642.1489, 644.1479.

Dibenzyl 2,5-*trans*-2,5-bis(4-methoxyphenyl)-7-oxoazepane-3,3-dicarboxylate (5f.a)

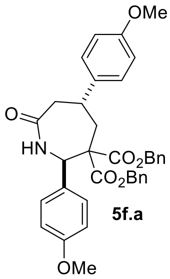

5f.a

The title compound was prepared following the **GP6**, using dibenzyl 2-(4-methoxyphenyl)cyclopropane-1,1-dicarboxylate (**2a**) (0.083 g, 0.20 mmol, 1.0 equiv.) and N-(1-((tert-butyldimethylsilyl)oxy)vinyl)-1-(4-methoxyphenyl)methanimine (**4f**) (0.087 g, 0.30 mmol, 1.5 equiv.). Upon column chromatography (Biotage flash chromatographer, 4 g SiO_2; EtOAc in pentane, 15 to 60%), it was obtained (0.086 g, 0.14 mmol, 72% yield – d.r. = 78 : 22) as a white, foamy solid.

R_f (pentane/EtOAc 6/4) = 0.39.
^{1}H NMR (400 MHz, Chloroform-*d*; the resolved signals corresponding to the minor diastereoisomer are underlined) δ 7.54 – 7.46 (m, 2H, Ar*H*), 7.39 – 7.33 (m, 2H, Ar*H*), 7.33 – 7.19 (m, 6H, Ar*H*), <u>7.05 – 6.99</u> (m), 6.98 – 6.89 (m, 4H, Ar*H*), 6.86 – 6.73 (m, 4H, Ar*H*), 6.12 (d, *J* = 6.9 Hz, 1H, (CO)N*H*), <u>6.04</u> (d, *J* = 6.7 Hz), <u>5.65</u> (d, *J* = 6.7 Hz), 5.40 (d, *J* = 12.0 Hz, 1H, C*H*$_2$Ph), 5.09 (d, *J* = 12.0 Hz, 1H, C*H*$_2$Ph), 5.03 (d, *J* = 7.0 Hz, 1H, NC*H*Ar), <u>5.00</u> (d, *J* = 12.4 Hz), <u>4.90</u> (d, *J* = 12.4 Hz), 4.70 (d, *J* = 12.2 Hz, 1H, C*H*$_2$Ph), 4.55 (d, *J* = 12.2 Hz, 1H, C*H*$_2$Ph), <u>4.48</u> (d, *J* = 12.3 Hz), 3.81 (s, 3H, O*Me*), 3.80 (s, 3H, O*Me*), <u>3.79</u> (s), 3.17 (dd, *J* = 13.7, 12.2 Hz, 1H, C*H*$_2$), <u>3.06</u> (dd, *J* = 14.5, 8.4 Hz), 2.88 (t, *J* = 12.1 Hz, 1H, C*H*), 2.79 (dt, *J* = 13.9, 2.1 Hz, 1H, C*H*$_2$), 2.75 – 2.62 (m, 1H, C*H*$_2$ + minor diast.), 2.55 (dd, *J* = 14.1, 12.3 Hz, 1H, C*H*$_2$), <u>2.47</u> (d, *J* = 11.5 Hz)
^{13}C NMR (101 MHz, Chloroform-*d*; mixture of diastereoisomers; the signals corresponding to the two diastereoisomers are only partially resolved) δ 173.7, 169.1, 168.0, 159.6, 158.4, 137.5, 135.0, 134.4, 130.3, 128.8, 128.8, 128.7, 128.7, 128.6, 128.5, 128.4, 128.4, 128.3, 128.1, 128.0, 127.7, 127.3, 114.1, 113.8, 67.5, 63.4, 62.1, 55.3, 47.2, 42.8, 37.2.
IR (ν_{max}, cm^{-1}) 3032 (w), 2952 (w), 1725 (s), 1600 (w), 1496 (w), 1452 (m), 1381 (m), 1314 (m), 1272 (s), 1200 (s), 1125 (s), 1029 (w), 978 (w), 903 (w), 867 (w), 741 (s), 698 (s).
HRMS (ESI/QTOF) m/z: [M + Na]$^+$ Calcd for $C_{36}H_{35}NNaO_7^+$ 616.2306; Found 616.2319.

Dibenzyl 2,5-*trans*-5-(4-methoxyphenyl)-7-oxo-2-((E)-1-phenylprop-1-en-2-yl)azepane-3,3-dicarboxylate (5g.a)

The title compound was prepared following the **GP6**, using dibenzyl 2-(4-methoxyphenyl)cyclopropane-1,1-dicarboxylate (**2a**) (0.083 g, 0.20 mmol, 1.0 equiv.) and N-(1-((*tert*-butyldimethylsilyl)oxy)vinyl)-2-methyl-3-phenylprop-2-en-1-imine (**4g**) (0.090 g, 0.30 mmol, 1.5 equiv.). Upon column chromatography (Biotage flash chromatographer, 4 g SiO_2; EtOAc in pentane, 15 to 60%), it was obtained (0.091 g, 0.15 mmol, 75% yield – dr 91 : 9) as a white, foamy solid.

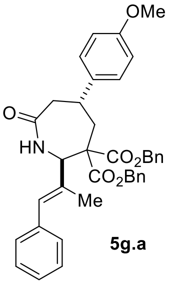

5g.a

R_f (pentane/EtOAc 6/4) 0.48.

^{1}H NMR (400 MHz, Chloroform-*d*) δ 7.40 – 7.28 (m, 9H, Ar*H*), 7.27 (m,1H, C=C*H*), 7.26 – 7.20 (m, 2H, Ar*H*), 7.08 (td, *J* = 8.5, 1.7 Hz, 4H, Ar*H*), 6.93 – 6.86 (m, 2H, Ar*H*), 6.83 – 6.74 (m, 2H, Ar*H*), 6.04 (d, *J* = 7.7 Hz, 1H, (CO)N*H*), 5.41 (d, *J* = 12.0 Hz, 1H, *CH2*Ph), 5.07 (d, *J* = 12.0 Hz, 1H, *CH2*Ph), 5.00 (d, *J* = 12.2 Hz, 1H, *CH2*Ph), 4.89 (d, *J* = 12.2 Hz, 1H, *CH2*Ph), 4.60 (d, *J* = 7.7 Hz, 1H, NC*H*Ar), 3.78 (s, 3H, O*Me*), 3.10 (t, *J* = 13.0 Hz, 1H, C*H2*), 2.84 – 2.73 (m, 2H, C*H* and C*H2*), 2.62 (d, *J* = 13.7 Hz, 1H, C*H2*), 2.47 (dd, *J* = 14.1, 12.4 Hz, 1H, C*H2*), 1.95 (d, *J* = 1.3 Hz, 3H, *Me*C=CH).

^{13}C NMR (101 MHz, Chloroform-*d*; *the two benzylic C signals are not resolved from each other*) δ 174.3, 169.2, 168.1, 158.4, 137.3, 136.6, 135.1, 134.5, 133.7, 132.1, 129.0, 128.8, 128.7, 128.7, 128.6, 128.5, 128.2, 128.2, 127.3, 127.1, 114.1, 67.7, 67.5, 65.1, 63.0, 55.3, 42.7, 37.3, 16.3.

HRMS (ESI/QTOF) m/z: $[M + Na]^+$ Calcd for $C_{38}H_{37}NNaO_6^+$ 626.2513; Found 626.2520.

Dibenzyl 2,5-*trans*-5-(1,3-dioxoisoindolin-2-yl)-7-oxo-2-phenylazepane-3,3-dicarboxylate (5a.l)

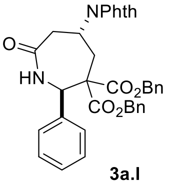

3a.l

The title compound was prepared following the **GP6**, using dibenzyl 2-(1,3-dioxoisoindol-2-yl)cyclopropane-1,1-dicarboxylate (**2l**) (0.091 g, 0.20 mmol, 1.0 equiv.), N-[1-[*tert*-butyl(dimethyl)silyl]oxyethenyl]-1-phenylmethanimine) (**4a**) (0.078 g, 0.30 mmol, 1.5 equiv.) and $Yb(OTf)_3$ (25 mg, 0.040 mmol, 20 mol%) with 150 mg MS 3Å. Upon column chromatography (Biotage flash chromatographer, 4 g SiO_2; EtOAc in pentane, 20 to 65%), it was obtained (0.090 g, 0.15 mmol, 75% yield – d.r. > 95 : 5) as a white, foamy solid.

R_f (pentane/EtOAc 6/4) 0.30.

^{1}H NMR (400 MHz, Chloroform-*d*) δ 7.92 – 7.82 (m, 2H, NPhth*H*), 7.79 – 7.70 (m, 2H, NPhth*H*), 7.61 – 7.51 (m, 2H, Ar*H*), 7.28 (d, *J* = 1.5 Hz, 7H, Ar*H*), 7.25 – 7.16 (m, 4H, Ar*H*), 6.88 – 6.80 (m, 2H, Ar*H*), 6.18 (d, *J* = 6.8 Hz, 1H, (CO)N*H*), 5.34 (d, *J* = 11.9 Hz, 1H, *CH2*Ph), 5.17 (d, *J* = 11.9 Hz, 1H, *CH2*Ph), 5.11 (d, *J* = 7.0 Hz, 1H, PhthNC*H*), 4.70 – 4.58 (m, 2H, *CH2*Ph and C*H*), 4.37 (d, *J* = 12.2 Hz, 1H, *CH2*Ph), 4.05 (dd, *J* = 14.3, 12.5 Hz, 1H, C*H2*), 3.29 (dd, *J* = 13.8, 12.3 Hz, 1H, C*H2*), 2.74 – 2.62 (m, 2H, C*H2*).

^{13}C NMR (101 MHz, Chloroform-*d*; the signals corresponding to the aromatic C are not completely resolved) δ 171.4, 168.1, 167.5, 167.2, 136.2, 134.7, 134.3, 134.2, 131.7, 129.0, 129.0, 128.9, 128.7, 128.5, 128.5, 128.4, 128.2, 123.5, 68.1, 67.6, 63.4, 62.3, 43.9, 42.3, 41.0.

HRMS (ESI/QTOF) m/z: $[M + Na]^+$ Calcd for $C_{36}H_{30}N_2NaO_7^+$ 625.1945; Found 625.1943.

Larger scale experiments:

Starting from 1.0 mmol of cyclopropane 2l: Inside a glove-box, a 25 mL round-bottomed vial was charged with ytterbium(III) triflate (124 mg, 0.200 mmol, 20 mol%), and activated molecular sieves (3Å; ca. 700 mg). The vial was sealed with a septum, and taken out of the glove-box. DCM (6.0 mL) was added by syringe, and the resulting suspension was stirred at room temperature for 30 minutes. In a separate sealed 25 mL, round-bottom vial, dibenzyl 2-(1,3-dioxoisoindol-2-yl)cyclopropane-1,1-dicarboxylate (**2l**) (0.455 g, 1.00 mmol, 1.0 equiv.) was dissolved in DCM (4.0 mL). (*E*)-N-[1-[*tert*-Butyl(dimethyl)silyl]oxyethenyl]-1-phenylmethanimine (**4a**) (0.392 g, 1.50 mmol, 1.5 equiv.) was added by syringe under stirring. The resulting pale yellow, clear solution was transferred into the previously prepared suspension by syringe. A pale yellow mixture was formed, and was stirred at room temperature for 17 hours. After this time, TLC analysis (DCM/MeOH 95/5) showed complete conversion, while the mixture looked like a yellow suspension. The reaction was quenched by addition of methanol (ca. 2 mL), celite (ca. 10 g) was added, and the volatiles were distilled off under reduced pressure. The resulting crude product was submitted to column chromatography (Biotage, 25 g SiO_2; MeOH in DCM, 0 to 5%). Dibenzyl (2,5-*trans*)-5-(1,3-dioxoisoindolin-2-yl)-7-oxo-2-phenylazepane-3,3-dicarboxylate (**5a.l**) (0.550 g, 0.913 mmol, 91% yield; d.r. > 95 : 5) was collected as a white foam.

The same experiment was reiterated a second time to provide **5a.l** (0.553 g, 0.918 mmol) in 92% yield.

Average yield over two experiments: 91%.

Dibenzyl 2,5-*trans*-5-(1,3-dioxoisoindolin-2-yl)-7-oxo-2-(4-(trifluoromethyl)phenyl)azepane-3,3-dicarboxylate (5b.l)

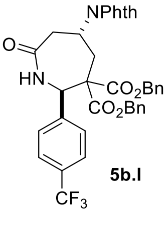

5b.l

The title compound was prepared following the **GP6**, using dibenzyl 2-(1,3-dioxoisoindol-2-yl)cyclopropane-1,1-dicarboxylate (**2l**) (0.091 g, 0.20 mmol, 1.0 equiv.) and N-(1-((*tert*-butyldimethylsilyl)oxy)vinyl)-1-(4-(trifluoromethyl)phenyl)methanimine (**4b**) (0.098 g, 0.30 mmol, 1.5 equiv.). Upon column chromatography (Biotage flash chromatographer, 4 g SiO_2; EtOAc in pentane, 15 to 60%), it was obtained (0.116 g, 0.170 mmol, 85% yield – d.r. > 95 : 5) as an off-white, foamy solid.

R_f (pentane/EtOAc 6/4) = 0.32.

^{1}H NMR (400 MHz, Chloroform-*d*) δ 7.87 (dd, *J* = 5.5, 3.0 Hz, 2H, NPhth*H*), 7.75 (dd, *J* = 5.5, 3.0 Hz, 2H, NPhth*H*), 7.68 – 7.64 (m, 2H, Ar*H*), 7.47 – 7.41 (m, 2H, Ar*H*), 7.37 – 7.28 (m, 5H), 7.28 – 7.24 (m, 1H), 7.24 – 7.16 (m, 2H, Ar*H*), 6.85 – 6.78 (m, 2H, Ar*H*), 6.07 (d, *J* = 7.0 Hz, 1H, (CO)N*H*), 5.38 (d, *J* = 11.9 Hz, 1H, CH_2Ph), 5.24 – 5.16 (m, 2H, CH_2Ph and PhthNC*H*), 4.73 – 4.62 (m, 2H, CH_2Ph and C*H*), 4.49 (d, *J* = 12.0 Hz, 1H, CH_2Ph), 4.04 (m, 1H, CH_2), 3.27 (m, 1H, CH_2), 2.77 – 2.64 (m, 2H, CH_2).
^{13}C NMR (101 MHz, Chloroform-*d*) δ 171.3, 167.9, 167.4, 167.2, 139.9, 134.6, 134.3, 133.8, 131.6, 131.0 (q, *J* = 32.6 Hz), 129.6, 129.0, 128.7, 128.6, 128.6, 128.5, 128.4, 126.1 (q, *J* = 11.1 Hz),125.5 (q, *J* = 3.6 Hz), 123.7 (q, J = 272.3 Hz), 68.4, 67.9, 63.2, 61.7, 43.8, 42.2, 40.8.
^{19}F NMR (376 MHz, Chloroform-*d*) δ -62.8.
HRMS (ESI/QTOF) m/z: $[M + Na]^+$ Calcd for $C_{37}H_{29}F_3N_2NaO_7^+$ 693.1819; Found 693.1835.

Dibenzyl 2,5-*trans*-2-(4-chlorophenyl)-5-(1,3-dioxoisoindolin-2-yl)-7-oxoazepane-3,3-dicarboxylate (5c.l)

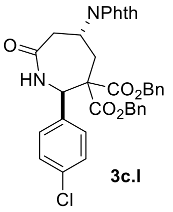

3c.l

The title compound was prepared following the **GP6**, using dibenzyl 2-(1,3-dioxoisoindol-2-yl)cyclopropane-1,1-dicarboxylate (**2l**) (0.091 g, 0.20 mmol, 1.0 equiv.) and N-(1-((*tert*-butyldimethylsilyl)oxy)vinyl)-1-(4-(trifluoromethyl)phenyl)methanimine (**4c**) (0.098 g, 0.30 mmol, 1.5 equiv.). Upon column chromatography (Biotage flash chromatographer, 4 g SiO_2; EtOAc in pentane, 15 to 75%), it was obtained (0.101 g, 0.185 mmol, 79% yield – d.r. = 95 : 5) as a white, foamy solid.

R_f (pentane/EtOAc 6/4) = 0.30.
^{1}H NMR (400 MHz, Chloroform-*d*) δ 7.91 – 7.80 (m, 2H, NPhth*H*), 7.79 – 7.70 (m, 2H, NPhth*H*), 7.51 – 7.42 (m, 2H, Ar*H*), 7.31 (m, 5H, Ar*H*), 7.28 (m, 1H, Ar*H*), 7.23 (ddt, *J* = 8.3, 6.8, 1.4 Hz, 2H, Ar*H*), 7.18 – 7.12 (m, 2H, Ar*H*), 6.90 – 6.82 (m, 2H, Ar*H*), 6.06 (d, *J* = 6.9 Hz, 1H, (CO)N*H*), 5.37 (d, *J* = 12.0 Hz, 1H, CH_2Ph), 5.19 (d, *J* = 11.9 Hz, 1H, CH_2Ph), 5.08 (d, *J* = 7.0 Hz, 1H, PhthNC*H*), 4.69 – 4.58 (m, 2H, CH_2Ph and C*H*), 4.53 (m, 1H, CH_2Ph), 4.02 (dd, *J* = 14.1, 12.5 Hz, 1H, CH_2), 3.26 (m, 1H, CH_2), 2.74 – 2.62 (m, 2H, CH_2).
^{13}C NMR (101 MHz, Chloroform-*d*; the two benzylic C signals are not revolved from each other) δ 171.3, 168.0, 167.5, 167.2, 134.9, 134.7, 134.6, 134.3, 133.9, 131.6, 130.9, 130.5, 129.5, 129.0, 128.7, 128.6, 128.5, 128.4, 123.5, 68.3, 67.8, 61.6, 43.9, 42.2, 40.9.
HRMS (ESI/QTOF) m/z: $[M + Na]^+$ Calcd for $C_{36}H_{29}ClN_2NaO_7^+$ 659.1555, 661.1547; Found 659.1554, 661.1551.

Dibenzyl 2,5-*trans*-5-(1,3-dioxoisoindolin-2-yl)-2-(2-fluorophenyl)-7-oxoazepane-3,3-dicarboxylate (5d.l)

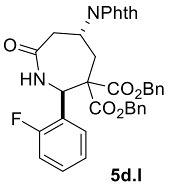

5d.l

The title compound was prepared following the **GP6**, using dibenzyl 2-(1,3-dioxoisoindol-2-yl)cyclopropane-1,1-dicarboxylate (**2l**) (0.091 g, 0.20 mmol, 1.0 equiv.) and (*E*)-N-(1-((*tert*-Butyldimethylsilyl)oxy)vinyl)-1-(2-fluorophenyl)methanimine (**4d**) (0.084 g, 0.30 mmol, 1.5 equiv.). Upon column chromatography (Biotage flash chromatographer, 4 g SiO_2; EtOAc in pentane, 15 to 75%), it was obtained (0.112 g, 0.180 mmol, 90% yield – d.r. > 95 : 5) as a white, foamy solid.

R_f (pentane/EtOAc 6/4) = 0.30.
^{1}H NMR (400 MHz, Chloroform-*d*) δ 7.95 (td, *J* = 7.9, 1.8 Hz, 1H, Ar*H*), 7.86 (dd, *J* = 5.5, 3.0 Hz, 2H, NPhth*H*), 7.74 (dd, *J* = 5.5, 3.0 Hz, 2H, NPhth*H*), 7.37 – 7.28 (m, 5H, Ar*H*), 7.28 – 7.18 (m, 4H, Ar*H*), 7.02 (m, 1H, Ar*H*), 7.97 (m, 1H, Ar*H*), 6.94 – 6.89 (m, 2H, Ar*H*), 5.99 (d, *J* = 7.3 Hz, 1H, (CO)N*H*), 5.63 (d, *J* = 7.3 Hz, 1H, PhthNC*H*), 5.41 (d, *J* = 12.0 Hz, 1H, CH_2Ph), 5.23 (d, *J* = 12.0 Hz, 1H, CH_2Ph), 4.77 – 4.60 (m, 2H CH_2Ph and C*H*), 4.56 (d, *J* = 12.1 Hz, 1H, CH_2Ph), 3.25 (dd, *J* = 13.5, 12.3 Hz, 1H, CH_2), 4.11 (m, 1H, CH_2), 2.69 (ddt, *J* = 18.0, 14.2, 2.1 Hz, 2H, CH_2).
^{13}C NMR (101 MHz, Chloroform-*d*; the signals corresponding to the aromatic C are not completely resolved) δ 171.5, 167.9, 167.7, 167.1, 159.9 (d, *J* = 249.3 Hz), 134.7, 134.2 (d, *J* = 19.1 Hz), 131.7, 130.7 (d, *J* = 8.8 Hz), 129.7, 128.9, 128.6 (d, *J* = 7.6 Hz), 128.56 (d, *J* = 6.6 Hz), 128.4, 124.6 (d, *J* = 3.9 Hz), 123.9 (d, *J* = 11.9 Hz), 123.5, 115.6 (d, *J* = 23.2 Hz), 68.3, 67.8, 62.9, 52.9 (d, *J* = 5.8 Hz), 44.1, 42.4, 40.8.
^{19}F NMR (376 MHz, Chloroform-*d*) δ -114.7.
HRMS (ESI/QTOF) m/z: $[M + Na]^+$ Calcd for $C_{36}H_{29}FN_2NaO_7^+$ 643.1851; Found 643.1838.

Dibenzyl 2,5-*trans*-2-(3-bromophenyl)-5-(1,3-dioxoisoindolin-2-yl)-7-oxoazepane-3,3-dicarboxylate (5e.l)

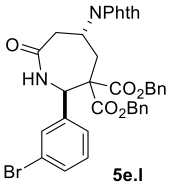

5e.l

The title compound was prepared following the **GP6**, using dibenzyl 2-(1,3-dioxoisoindol-2-yl)cyclopropane-1,1-dicarboxylate (**2l**) (0.091 g, 0.20 mmol, 1.0 equiv.) and N-(1-((*tert*-Butyldimethylsilyl)oxy)vinyl)-1-(4-chlorophenyl)methanimine (**4e**) (0.089 g, 0.30 mmol, 1.5 equiv.). Upon column chromatography (Biotage flash chromatographer, 4 g SiO_2; EtOAc in pentane, 15 to 75%), it was obtained (95% pure; 0.124 g, 0.172 mmol, 86% yield; d.r. > 95 : 5) as a white, foamy solid.

R_f (pentane/EtOAc 6/4): 0.22.
^{1}H NMR (400 MHz, Chloroform-*d*) δ 7.85 (tt, *J* = 5.2, 2.5 Hz, 2H, NPhth*H*), 7.78 (t, *J* = 1.9 Hz, 1H, Ar*H*), 7.74 (dd, *J* = 5.5, 3.1 Hz, 2H, NPhth*H*), 7.50 (ddd, *J* = 7.9, 1.9, 1.0 Hz, 1H, Ar*H*), 7.38 (ddd, *J* = 8.0, 2.0, 0.9 Hz, 1H, Ar*H*), 7.34 – 7.27 (m, 5H, Ar*H*), 7.27 – 7.21 (m, 3H, Ar*H*), 7.08 (t, *J* = 7.9 Hz, 1H, Ar*H*), 6.92 – 6.85 (m, 2H, Ar*H*), 6.15 (d, *J* = 7.0 Hz, 1H, (CO)N*H*), 5.37 (d, *J* = 11.9 Hz, 1H, CH_2Ph), 5.19 (d, *J* = 11.9 Hz, 1H, CH_2Ph), 5.08 (d, *J* = 7.1 Hz, 1H, PhthNC*H*), 4.72 – 4.58 (m, 2H, CH_2Ph and C*H*), 4.51 (d, *J* = 12.1 Hz, 1H, CH_2Ph), 4.02 (dd, *J* = 14.3, 12.5 Hz, 1H, CH_2), 3.26 (dd, *J* = 13.8, 12.3 Hz, 1H, CH_2), 2.74 – 2.61 (m, 2H, CH_2).

^{13}C NMR (101 MHz, Chloroform-*d*; the signal corresponding to one aromatic C is not resolved) δ 171.3, 167.9, 167.4, 167.2, 138.3, 134.7, 134.3, 134.0, 132.2, 132.1, 131.6, 130.1, 128.9, 128.6, 128.5, 128.5, 128.2, 127.8, 123.5, 122.6, 68.3, 67.8, 63.2, 61.7, 43.8, 42.2, 40.8.
HRMS (ESI/QTOF) m/z: [M + H]$^+$ Calcd for $C_{36}H_{30}BrN_2O_7^+$ 681.1231, 683.1218; Found 681.1224, 683.1214.

Dibenzyl 2,5-*trans*-5-(1,3-dioxoisoindolin-2-yl)-2-(4-methoxyphenyl)-7-oxoazepane-3,3-dicarboxylate (5f.l)

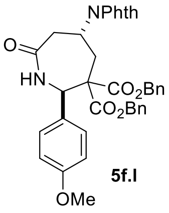

5f.l

The title compound was prepared following the **GP6**, using dibenzyl 2-(1,3-dioxoisoindol-2-yl)cyclopropane-1,1-dicarboxylate (**2l**) (0.091 g, 0.20 mmol, 1.0 equiv.) and N-(1-((tert-butyldimethylsilyl)oxy)vinyl)-1-(4-methoxyphenyl)methanimine (**4f**) (0.087 g, 0.30 mmol, 1.5 equiv.). Upon column chromatography (Biotage flash chromatographer, 4 g SiO_2; EtOAc in pentane, 15 to 70%), it was obtained (0.087 g, 0.14 mmol, 69% yield – d.r. 85 : 15) as a white, foamy solid.

R_f (pentane/EtOAc 6/4): 0.27.
^{1}H NMR (400 MHz, Chloroform-*d*; the resolved signals corresponding to the minor diastereoisomer are underlined) δ 7.91 – 7.80 (m, 2H, NPhth*H*), 7.80 – 7.68 (m, 2H, NPhth*H*), <u>7.57 – 7.51</u> (m), 7.50 – 7.44 (m, 2H, Ar*H*), 7.30 – 7.27 (m, 5H, Ar*H*), 7.25 – 7.18 (m, 3H, Ar*H*), <u>6.98</u> (d, *J* = 7.1 Hz), 6.91 – 6.84 (m, 2H, Ar*H*), <u>6.83 – 6.78</u> (m), 6.76 – 6.69 (m, 2H, Ar*H*), <u>6.17</u> (d, *J* = 7.1 Hz), 6.13 (d, *J* = 6.8 Hz, 1H, (CO)N*H*), <u>5.51</u> (d, *J* = 6.7 Hz), <u>5.47</u> (d, *J* = 12.2 Hz, *CH*2Ph), 5.36 (d, *J* = 11.9 Hz, 1H, *CH*2Ph), 5.18 (d, *J* = 11.9 Hz, 1H, *CH*2Ph), 5.06 (d, *J* = 6.8 Hz, 1H, PhthNC*H*), <u>4.93</u> (d, *J* = 12.2 Hz), 4.68 – 4.57 (m, 2H, *CH*2Ph and C*H*), 4.48 (d, *J* = 12.1 Hz, 1H, *CH*2Ph), 4.03 (dd, *J* = 14.3, 12.4 Hz, 1H, C*H*2), 3.28 (m, 1H, C*H*2), 2.73 – 2.61 (m, 2H, C*H*2).
^{13}C NMR (101 MHz, Chloroform-*d*; the signals corresponding to the minor diastereoisomer are not resolved; for the major diastereoisomer: the signals corresponding to the aromatic C are not completely resolved) δ 171.3, 168.2, 167.6, 167.2, 159.7, 134.8, 134.3, 131.7, 130.3, 128.9, 128.5, 128.5, 128.4, 128.3, 128.3, 128.2, 123.5, 113.8, 68.1, 67.6, 63.5, 55.2, 44.0, 42.2, 41.0.
HRMS (ESI/QTOF) m/z: [M + Na]$^+$ Calcd for $C_{37}H_{32}N_2NaO_8^+$ 655.2056; Found 655.2065.

Dibenzyl 2,5-*trans*-5-(1,3-dioxoisoindolin-2-yl)-7-oxo-2-((E)-1-phenylprop-1-en-2-yl)azepane-3,3-dicarboxylate (5g.l)

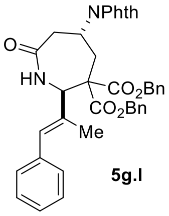

5g.l

The title compound was prepared following the **GP6**, using dibenzyl 2-(1,3-dioxoisoindol-2-yl)cyclopropane-1,1-dicarboxylate (**2l**) (0.091 g, 0.20 mmol, 1.0 equiv.) and N-(1-((*tert*-butyldimethylsilyl)oxy)vinyl)-2-methyl-3-phenylprop-2-en-1-imine (**4g**) (0.090 g, 0.30 mmol, 1.5 equiv.). Upon column chromatography (Biotage flash chromatographer, 4 g SiO_2; EtOAc in pentane, 15 to 60%), it was obtained (0.088 g, 0.14 mmol, 68% yield – dr > 95 : 5) as a pale yellow, foamy solid.

R_f (pentane/EtOAc 6/4) 0.29.
^{1}H NMR (400 MHz, Chloroform-*d*) δ 7.88 (dd, *J* = 5.5, 3.1 Hz, 2H, NPhth*H*), 7.77 (dd, *J* = 5.5, 3.1 Hz, 2H, NPhth*H*), 7.41 – 7.31 (m, 7*H*), 7.28 – 7.21 (m, 2H, Ar*H*), 7.22 – 7.12 (m, 2H, Ar*H*), 7.10 – 7.05 (m, 2*H*, ArH), 7.04 – 7.00 (m, 2H, Ar*H*), 6.81 (s, 1H, C=C*H*), 6.10 (d, *J* = 7.8 Hz, 1H, (CO)N*H*), 5.38 (d, *J* = 12.0 Hz, 1H, *CH*2Ph), 5.23 (d, *J* = 12.0 Hz, 1H, *CH*2Ph), 5.00 (d, *J* = 12.2 Hz, 1H, *CH*2Ph), 4.90 (d, *J* = 12.1 Hz, 1H, *CH*2Ph), 4.68 (d, *J* = 7.7 Hz, 1H, PhthNC*H*), 4.58 (t, *J* = 12.5 Hz, 1H, C*H* or C*H*2), 4.07 – 3.94 (m, 1H, C*H* or C*H*2), 3.24 (t, *J* = 12.9 Hz, 1H, C*H* or C*H*2), 2.71 – 2.58 (m, 2H, C*H* or C*H*2), 1.96 (d, *J* = 1.3 Hz, 3H, *Me*C=CH).
^{13}C NMR (101 MHz, Chloroform-*d*; the signals corresponding to the aromatic C are not completely resolved; the signal corresponding to one aliphatic C is not resolved) δ 171.2, 168.2, 167.1, 167.0, 149.0, 136.1, 134.7, 134.3, 134.2, 131.6, 128.9, 128.6, 128.5, 123.5, 112.5, 107.4, 68.3, 68.0, 62.8, 54.7, 43.7, 40.9, 40.6.
HRMS (ESI/QTOF) m/z: [M + Na]$^+$ Calcd for $C_{39}H_{34}N_2NaO_7^+$ 665.2258; Found 665.2273.

Dibenzyl 2,5-*trans*-5-(3-(4-(*tert*-butyl)benzyl)-5-methyl-2,4-dioxo-3,4-dihydropyrimidin-1(2*H*)-yl)-7-oxo-2-phenylazepane-3,3-dicarboxylate (5a.m)

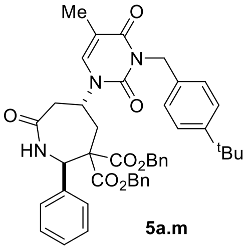

5a.m

The title compound was prepared following the **GP6**, using dibenzyl 2-(3-(4-(*tert*-butyl)benzyl)-5-methyl-2,4-dioxo-3,4-dihydropyrimidin-1(2*H*)-yl)cyclopropane-1,1-dicarboxylate (**2a**) (0.12 g, 0.20 mmol, 1.0 equiv.) and N-[1-[*tert*-Butyl(dimethyl)silyl]oxyethenyl]-1-phenylmethanimine (**4m**) (0.131 g, 0.500 mmol, 2.5 equiv.). Upon column chromatography (Biotage flash chromatographer, 4 g SiO_2; EtOAc in pentane, 20 to 70%), it was obtained (0.088 g, 0.12 mmol, 60% yield – d.r. > 95 : 5) as a white solid.

R_f (pentane/EtOAc 5/5) = 0.31.
Melting point: 191.2 - 194.9 °C.
^{1}H NMR (acquired at 339K, 400 MHz, DMSO-d_6) δ 7.70 (s, 1H, C*H*=CMe), 7.64 – 7.59 (m, 2H, Ar*H*), 7.51 (d, *J* = 6.7 Hz, 1H, (CO)NH), 7.35 – 7.29 (m, 5H, Ar*H*), 7.29 – 7.17 (m, 10H, Ar*H*), 6.95 – 6.91 (m, 2H, Ar*H*), 5.32 (d, *J* = 7.0 Hz, 1H, NC*H*Ph), 5.25 (d, *J* = 12.5 Hz, 1H, *CH*2Ar), 5.20 (d, *J* = 12.4 Hz, 1H, *CH*2Ar), 5.02 (d, *J* = 14.3 Hz, 1H, *CH*2Ar), 4.96 (d, *J* = 14.2 Hz, 1H, *CH*2Ar), 4.84 (br s, 1H, C*H*), 4.69 (d, *J* = 12.5 Hz, 1H, *CH*2Ar), 4.48 (d, *J* = 12.5 Hz, 1H, *CH*2Ar), 3.93 (t, *J* = 13.1 Hz, 1H, C*H*2), 2.95 (t, *J* = 12.7 Hz, 1H, C*H*2), 2.55 (m, 1H, C*H*2), 2.36 (d, *J* = 14.1 Hz, 1H, C*H*2), 1.88 (d, *J* = 1.2 Hz, 3H, CH=C*Me*), 1.27 (s, 9H, ^{t}*Bu*).

^{13}C NMR (acquired at 339K, 101 MHz, DMSO-d_6; the signals corresponding to three C(sp2) and one aliphatic C are not completely resolved) δ 171.1, 168.6, 167.9, 163.0, 150.9, 150.0, 137.0, 135.6, 135.1, 134.7, 130.1, 128.7, 128.7, 128.5, 128.4, 128.3, 128.2, 127.0, 125.5, 109.5, 67.7, 67.4, 63.6, 61.1, 44.2, 42.7, 34.6, 31.6, 13.2.
HRMS (ESI/QTOF) m/z: $[M + H]^+$ Calcd for $C_{44}H_{46}N_3O_7^+$ 728.3330; Found 728.3336.

5. Optimization and scope of the asymmetric version of reaction

5.1 Synthesis of the Ligands

5.1.1 PyBox ligands: Tested ones and preparation of the non-commercially availables ligands

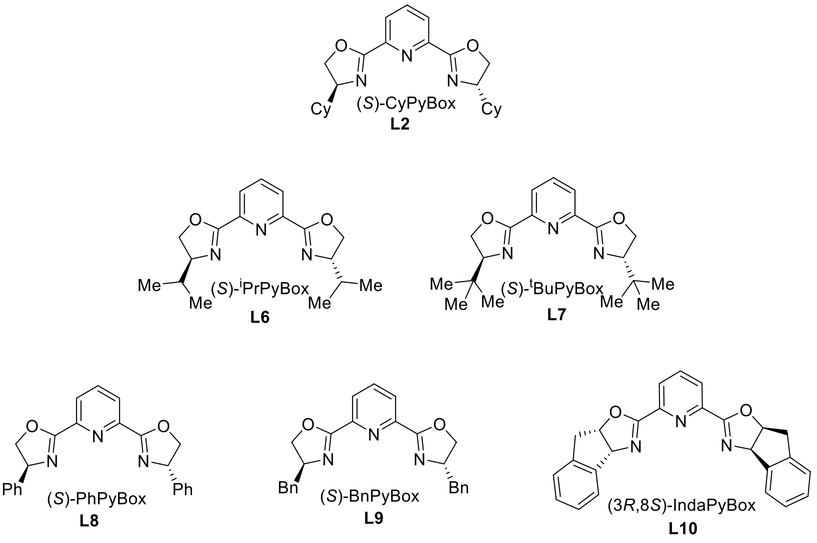

Figure S1. PyBox ligands that were tested during the optimization.

2,6-Bis((S)-4-cyclohexyl-4,5-dihydrooxazol-2-yl)pyridine ((*S*)-CyPYBox) (L2)

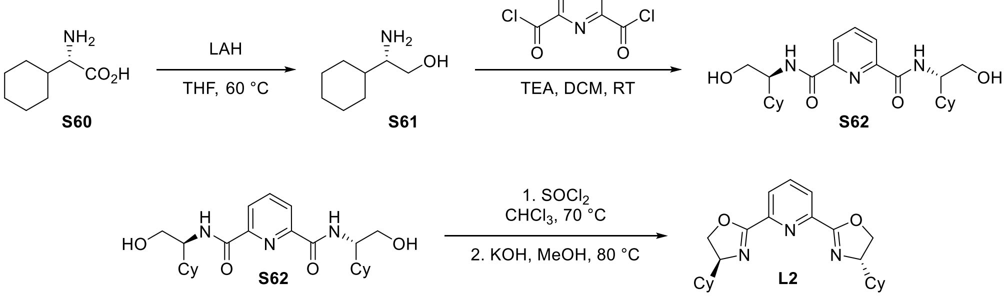

Following a reported procedure,[22] a 100 mL, two-necked, round-bottomed flask, equipped with a Liebig condenser, was charged with lithium aluminum hydride (1.1 g, 29 mmol, 3.0 equiv.). The flask was closed with a septum and taken out of the glove-box. THF (24 mL) was then added by syringe. Finally, (*S*)-2-amino-2-cyclohexylacetic acid (**S60**) (1.5 g, 9.5 mmol, 1.0 equiv.) was added in portions at room temperature (with significant gas release). The grey suspension was then heated to reflux (heating source at 80 °C), and stirred at this temperature for 5 hours. It was then allowed to cool down to 0 °C (ice - water bath), and the reaction was quenched by very cautions and slow addition of water (1.1 mL), aq. NaOH (1.0 M; 1.1 mL), and water (3.3 mL). The now white suspension was then stirred at room temperature for 20 minutes, diluted with DCM (40 mL), the solids were filtered off through a pad of celite and washed with several portions of DCM. The resulting turbid filtrate was washed with a mixture of aq. NaOH (1.0 M; 40 mL) and of sat. aq. sodium and potassium tartrate (40 mL). Upon separation, the aqueous layer was extracted once with DCM (50 mL). The combined organic layers were washed again with aq. NaOH (1.0 M; 50 mL), dried over Na_2SO_4, filtered, and concentrated under vacuum. The resulting white solid was triturated with pentane, and collected by suction filtration. (*S*)-2-Amino-2-cyclohexylethan-1-ol (**S61**) (1.6 g, 11 mmol, 59% yield) was obtained as a white, crystalline solid.

^{1}H NMR (400 MHz, Chloroform-*d*) δ 3.65 (dd, *J* = 10.4, 4.0 Hz, 1H, C*H₂*OH), 3.29 (dd, *J* = 10.6, 8.7 Hz, 1H, C*H₂*OH), 2.57 (ddd, *J* = 8.8, 6.6, 4.0 Hz, 1H, *CH*NH₂), 1.95 (s, 3H. O*H* and N*H₂*), 1.82 – 1.70 (m, 2H, Cy*H*), 1.70 – 1.59 (m, 2H, Cy*H*), 1.31 – 1.07 (m, 5H, Cy*H*), 1.00 (qdd, *J* = 13.4, 7.2, 3.8 Hz, 2H, Cy*H*).

Following a slightly modified version of a reported procedure,[23] a 25 mL, single neck, round bottom flask was charged with (*S*)-2-amino-2-cyclohexylethan-1-ol (**S61**) (0.20 g, 1.4 mmol, 2.0 equiv.) and DCM (2.6 mL). The solution was cooled to 0 °C (ice - water bath), prior to the addition of triethylamine (0.20 mL, 1.4 mmol, 2.0 equiv.). At 0 °C, pyridine-2,6-dicarbonyl dichloride (0.146 g, 0.716 mmol, 1.0 equiv.) was then added in a single portion. The colorless mixture was then stirred at room temperature for 8 hours, slightly darkening to pale yellow during this time. It was then concentrated under reduced pressure, and directly submitted to column chromatography (Biotage flash chromatography, 4 g SiO_2; MeOH in DCM, 0 to 10%) to provide N^2,N^6-bis((*S*)-1-cyclohexyl-2-hydroxyethyl)pyridine-2,6-dicarboxamide (**S62**) (0.185 g, 0.443 mmol, 62% yield) as a white foam.

In a sealed 25 mL, round-bottom vial, N^2,N^6-bis((*S*)-1-cyclohexyl-2-hydroxyethyl)pyridine-2,6-dicarboxamide (**S62**) (0.185 g, 0.443 mmol, 1.0 equiv.) was suspended in chloroform (4.0 mL). At room temperature, thionyl chloride (0.34 mL, 4.7 mmol, 10 equiv.) was added drop-wise under stirring. The initial suspension became a clear solution during this addition. The mixture was then stirred at reflux (70-80 °C) for 3 hours. It was then allowed to cool down to room temperature, and subsequently concentrated under reduced pressure. Exhaustive removal of residual thionyl chloride was accomplished by azeotropic co-evaporation with toluene (twice). The resulting white foam was then dissolved in MeOH (5.0 mL) in a round-bottomed, single-necked flask equipped with a Liebig condenser. Ground KOH (ca. 66 mg, 1.18 mmol, 2.5 equiv.) was added, leading to the formation of a pale yellow solution, which was stirred at 80 °C. After already 30 minutes, a solid started precipitating. After 6 hours overall, the reaction was stopped; the mixture was allowed to cool down to room temperature, and then concentrated under reduced pressure. The residue was dissolved in DCM (30 mL), and this organic solution was washed with water (20 mL), brine, dried over Na_2SO_4, filtered and concentrated under vacuum. The resulting crude solid was submitted to column chromatography (Biotage flash chromatographer, 12 g SiO_2; EtOAc in DCM, 2 to 15%) to furnish 2,6-bis((S)-4-benzyl-4,5-dihydrooxazol-2-yl)pyridine (**L2**, (*S*)-CyPyBox) (0.122 g, 0.307 mmol, 65% yield) as a whitish solid.

^{1}H NMR (400 MHz, Chloroform-*d*) δ 8.29 (m, 2H, Py*H*), 7.88 (t, *J* = 7.8 Hz, 1H, Py*H*), 4.55 (t, *J* = 9.0 Hz, 2H, C*H₂*O), 4.28 (t, *J* = 7.9 Hz, 2H, C*H₂*O), 4.17 (q, *J* = 8.9, Hz, 2H, C*H*N), 2.01 (d, *J* = 12.8 Hz, 2H, Cy*H*), 1.83 – 1.72 (m, 4H, Cy*H*), 1.72 – 1.51 (m, 6H, Cy*H*), 1.24 – 0.94 (m, 10H, Cy*H*).
^{1}H-NMR data corresponded to the reported values (with a systematic shift of +.0.04 ppm).[24]

5.1.2 Box ligands: Tested ones and preparation of the non-commercially availables ligands

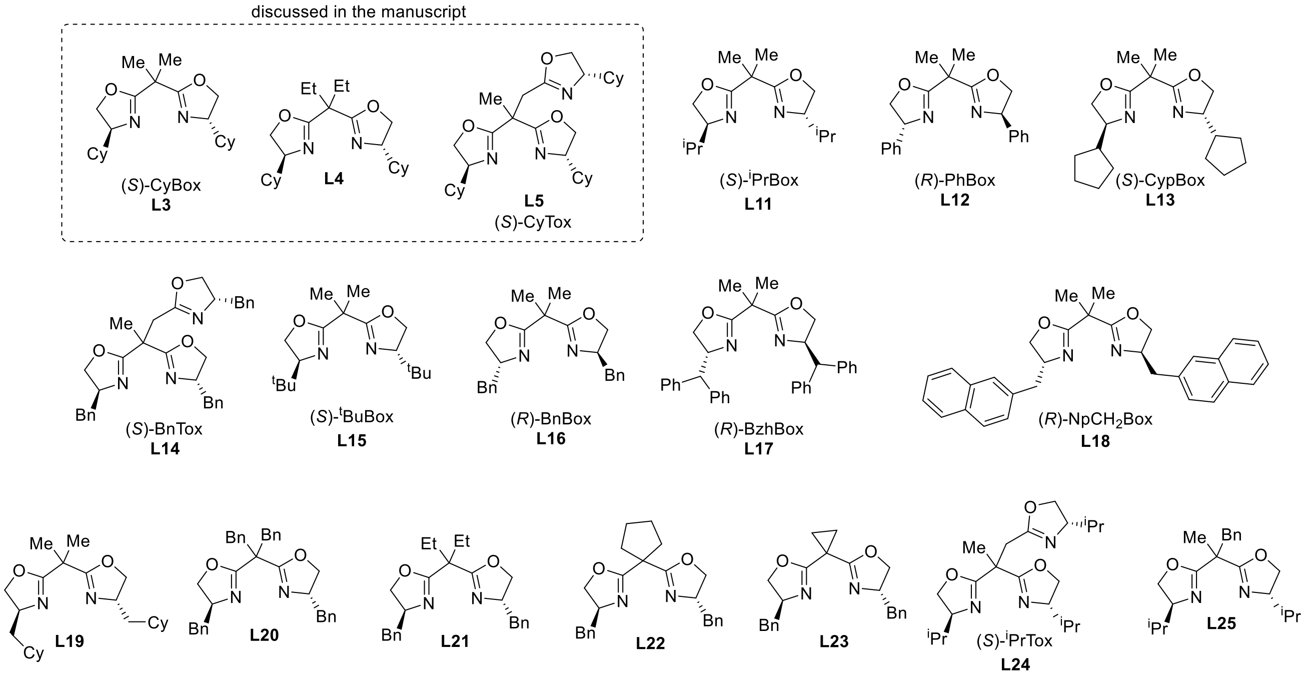

Figure S2. Bis- and Trisoxazoline (Box, Tox) ligands that were tested during the optimization.

(4S,4'S)-2,2'-(propane-2,2-diyl)bis(4-cyclohexyl-4,5-dihydrooxazole) ((*S*)-CyBox) (L3)

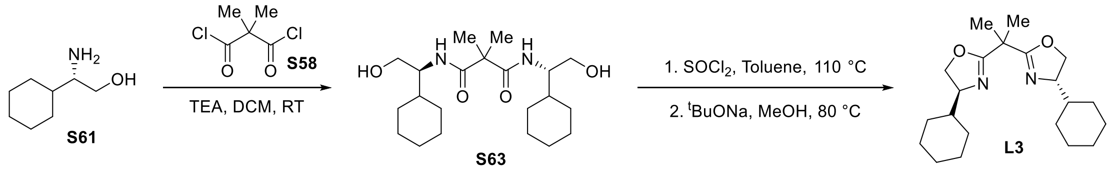

Following a slightly modified version of a reported procedure,[23] a 25 mL, single-necked, round-bottomed flask was charged with (*S*)-2-amino-2-cyclohexylethan-1-ol (**S61**) (0.341 g, 2.38 mmol, 2.1 equiv.). DCM (7 mL) was added, and the resulting suspension was cooled to 0 °C (ice - water bath), prior to the addition of triethylamine (0.40 mL, 2.8 mmol, 2.5 equiv.). Under stirring, a solution of dimethylmalonyl dichloride (**S58**) (0.15 mL, 1.1 mmol, 1.0 equiv.) in DCM (3.0 mL) was added slowly, at the same temperature. After the addition, the mixture was stirred at room temperature overnight, turning becoming clear, yellow solution. After 18 hours, the mixture was diluted with DCM (10 mL) and washed with sat. aq. $NaHCO_3$ (10 mL). The aqueous layer was extracted with DCM (3 x 15 mL). The combined organic layers were washed with brine, dried over Na_2SO_4, filtered, and concentrated under vacuum. The resulting pinkish crude solid was purified by column chromatography (Biotage flash chromatographer, 12 g SiO_2; MeOH in DCM, 1 to 12%) to afford (4*S*,4'*S*)-2,2'-(propane-2,2-diyl)bis(4-cyclohexyl-4,5-dihydrooxazole) (**S63**) (0.250 g, 0.635 mmol, 58% yield) as an off-white, foamy solid.
^{1}H NMR (400 MHz, Chloroform-*d*) δ 6.36 (d, *J* = 8.0 Hz, 2H, (CO)N*H*), 3.79 (d, *J* = 7.5 Hz, 2H, NC*H*), 3.74 (dd, *J* = 11.2, 3.6 Hz, 2H, C*H2*OH), 3.55 (dd, *J* = 11.6, 6.7 Hz, 2H, C*H2*OH), 1.79 – 1.52 (m, 6H, Cy*H*), 1.54 – 1.34 (m, 6H, Cy*H* and O*H*), 1,49 (s, 6H, Me), 1.31 – 1.10 (m, 8H, Cy*H*), 1.09 – 0.84 (m, 4H, Cy*H*).

Following a reported procedure,[23] N^1,N^3-bis((*S*)-1-cyclohexyl-2-hydroxyethyl)-2,2-dimethylmalonamide (**S63**) (0.25 g, 0.65 mmol, 1.0 equiv.) was suspended in toluene (5.5 mL) inside a sealed 25 mL, round-bottom vial. Thionyl chloride (0.12 mL, 1.6 mmol, 2.5 equiv.) was added by syringe at room temperature, leading to the dissolution of the solid. The clear solution was then heated at 110 °C (refluxed) for 3 hours, darkening from colorless to pale yellow during this time. It was then allowed to cool down to room temperature, diluted with DCM (15 mL) and poured onto ice and sat. aq. NH_4Cl. After the separation of the organic layer, the aqueous one was extracted with DCM (3 x 15 mL). The combined organic layers were washed with sat. aq. $NaHCO_3$ (15 mL), brine, dried over $MgSO_4$, filtered, and concentrated under vacuum. The resulting off-white crude solid was the desired N^1,N^3-bis((*S*)-2-chloro-1-cyclohexylethyl)-2,2-dimethylmalonamide (0.233 g, 0.555 mmol, 85% yield), which was found pure enough to be directly used for the next step without further purification.

Following a reported procedure,[23] inside a glove-box, a 25 mL round-bottom vial was charged with sodium *tert*-butoxide (0.15 g, 1.6 mmol, 2.8 equiv.), and subsequently sealed with a PTFE cap. The vial was taken out of the glove-box, and a suspension of N^1,N^3-bis((*S*)-2-chloro-1-cyclohexylethyl)-2,2-dimethylmalonamide (0.23 g, 0.56 mmol, 1.0 equiv.) in MeOH (4.8 mL) was added by syringe. The resulting yellow, turbid mixture was heated to 80 °C. The mixture was then stirred at 80 °C for 16 hours. After this time, TLC analysis (pentane/acetone 4/1) showed that the conversion was still incomplete. The reaction was stopped, and the mixture was concentrated under reduced pressure to give a pale-yellow paste. The latter was dissolved in DCM (ca. 15 mL), and washed with a solution of brine (ca. 15 mL) and water (ca. 15 mL). Upon separation, the aqueous layer was extacted with DCM (4 x 15 mL). All the organic layers were then combined, and washed with brine, dried over $MgSO_4$, filtered, and concentrated under vacuum. The resulting crude oil was dissolved in MeOH (dry; 4.8 mL); the so-obtained solution was transferred into a 25 mL, round-bottomed vial, sodium *tert*-butoxide (0.15 g, 1.6 mmol, 2.8 equiv.) was added, and - after sealing the vial with a PTFE septum - the mixture was stirred at 80 °C for another 16 hours. After this time, TLC analysis showed that the reaction had significantly progressed (with almost full conversion). The previously described work-up procedure was implemented again. The resulting crude oil was then submitted to column chromatography (SiO_2; Pentane/acetone 19/1 to 16/4) to provide (4*S*,4'*S*)-2,2'-(propane-2,2-diyl)bis(4-cyclohexyl-4,5-dihydrooxazole) (**L3**, (*S*)-CyBox) (0.095 g, 0.27 mmol, 49% yield) as a pale yellow solid.

^{1}H NMR (400 MHz, Chloroform-*d*) δ 4.19 (dd, *J* = 9.7, 8.2 Hz, 2H, C*H2*O), 4.02 (dd, *J* = 8.2, 7.2 Hz, 2H, C*H2*O), 3.93 (ddd, *J* = 9.8, 7.3, 5.8 Hz, 2H, C*H*N), 1.82 – 1.70 (m, 6H, Cy*H*), 1.70 – 1.52 (m, 4H, Cy*H*), 1.50 (s, 6H, *Me*), 1.45 (dddt, *J* = 12.2, 9.0, 5.9, 2.9 Hz, 2H, Cy*H*), 1.27 – 1.07 (m, 6H, Cy*H*), 0.97 (pd, *J* = 13.6, 12.8, 3.8 Hz, 4H, Cy*H*).
^{13}C NMR (101 MHz, Chloroform-*d*) δ 168.6, 70.9, 70.3, 42.4, 38.6, 29.2, 28.1, 26.5, 26.2, 26.0, 24.5.
NMR data corresponded to the reported values.[23]

(4S,4'S)-2,2'-(Pentane-3,3-diyl)bis(4-cyclohexyl-4,5-dihydrooxazole) (Et$_2$-(*S*)-CyTox) (L4)

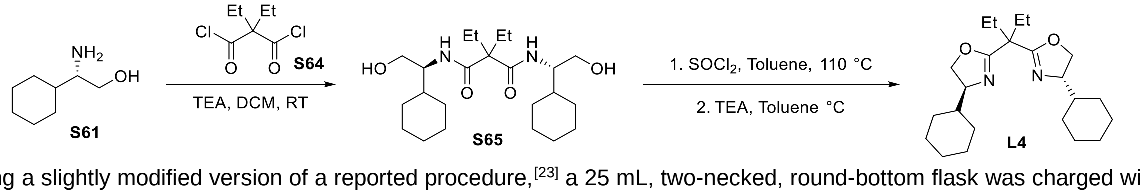

Following a slightly modified version of a reported procedure,[23] a 25 mL, two-necked, round-bottom flask was charged with (*S*)-2-amino-2-cyclohexylethan-1-ol (**S61**) (0.537 g, 3.75 mmol, 2.1 equiv.) and triethylamine (0.61 mL, 4.3 mmol, 2.5 equiv.) and DCM (13.0 mL). At room temperature, a solution of 1,1-diethyl malonyl dichloride (**S64**) (0.30 mL, 1.7 mmol, 1.0 equiv.) in DCM (3.3

mL) was then added drop-wise. After a few minutes, a white, fine precipitate started precipitating. The resulting off-white suspension was stirred overnight at room temperature. After 16 hours, the solution was diluted with additional DCM (30 mL), and washed with a 1:1 mixture of brine and sat. aq. NH_4Cl (50 mL). The aqueous layer was extracted with DCM (4 x 40 mL). The combined organic layers were washed with brine, dried over Na_2SO_4, filtered, and concentrated under vacuum. The resulting solid was washed with pentane, and collected by filtration. N^1,N^3-Bis((*S*)-1-cyclohexyl-2-hydroxyethyl)-2,2-diethylmalonamide (**S65**) (0.507 g, 1.23 mmol, 71% yield) was obtained as a white solid, which was used directly without further purification.

Following a slightly modified version of a reported procedure,[23] N^1,N^3-bis((*S*)-1-cyclohexyl-2-hydroxyethyl)-2,2-diethylmalonamide (**S65**) (0.507 g, 1.23 mmol, 1.0 equiv.) was dissolved in toluene (10 mL). At room temperature, thionyl chloride (0.23 mL, 3.1 mmol, 2.5 equiv.) was added drop-wise by syringe. The colorless, clear mixture was then heated to reflux (110 °C). Stirring was continued for 3.5 hours. The mixture was then allowed to cool down to room temperature, and it was concentrated under reduced pressure in order to remove the solvent and the excess of thionyl chloride. The solid residue was then partitioned between DCM (30 mL) and sat. aq. $NaHCO_3$ (20 mL). The aqueous layer was extracted with DCM (2 x 30 mL). The combined organic layers were washed brine, dried over Na_2SO_4, filtered, and concentrated under vacuum to provide a yellow, viscous oil. Trituration with pentane yielded N^1,N^3-bis((*S*)-2-chloro-1-cyclohexylethyl)-2,2-diethylmalonamide (0.40 g, 0.89 mmol, 72% yield) as a beige solid, which was used in the following step without further purification.

In a sealed 25 mL, round-bottomed vial, N^1,N^3-bis((*S*)-2-chloro-1-cyclohexylethyl)-2,2-diethylmalonamide (0.400 g, 0.894 mmol, 1.0 equiv.) was dissolved in toluene (dry; 11 mL). Et_3N (dry; 1.9 mL, 13 mmol, 15 equiv.) was then added by syringe, and the resulting mixture was heated to reflux (110 - 115 °C) and stirred for 16 hours. This resulted in the conversion of the initially clear, colorless solution into a yellow-beige suspension (due to the precipitation of a whitish solid). The mixture was allowed to cool down to room temperature, diluted with EtOAc (30 mL) and washed with sat. aq. NH_4Cl (30 mL). The aqueous layer was extracted with EtOAc (3 x 30 mL), and the combined organic layers were washed with brine, dried over Na_2SO_4, filtered, and concentrated under vacuum. The pale orange-brown crude oil was submitted to column chromatography (SiO_2; Pentane/acetone 19/1 to 9/1) to furnish (4*S*,4'*S*)-2,2'-(pentane-3,3-diyl)bis(4-cyclohexyl-4,5-dihydrooxazole) (**L4**, Et_2-(*S*)-CyBox; 0.334 g, 0.894 mmol, quantitative yield) as a very viscous, colorless oil, which became a colorless amorphous solid upon standing at 4 °C.

^1H NMR (400 MHz, Chloroform-*d*) δ 4.17 (td, *J* = 7.4, 1.5 Hz, 2H, C*H₂*O), 4.01 – 3.88 (m, 4H, C*H₂*O and C*H*N), 1.98 (ddt, *J* = 17.3, 14.2, 7.0 Hz, 4H, C*H₂*Me), 1.70 (ddd, *J* = 59.8, 31.7, 12.5 Hz, 10H, Cy*H*), 1.51 – 1.39 (m, 2H, Cy*H*), 1.30 – 1.06 (m, 6H, Cy*H*), 0.98 (ddt, *J* = 19.3, 15.6, 7.5 Hz, 4H, Cy*H*), 0.82 (t, *J* = 7.5 Hz, 6H, CH_2*Me*).
^{13}C NMR (101 MHz, Chloroform-*d*) δ 167.1, ^{13}C NMR (101 MHz, Chloroform-*d*) δ 71.1, 69.8, 46.6, 42.5, 29.5, 28.5, 26.6, 26.2, 25.2, 8.3.
HRMS (ESI/QTOF) m/z: $[M + H]^+$ Calcd for $C_{23}H_{39}N_2O_2^+$ 375.3006; Found 375.3011.
$[\alpha]_D^{20}$ = -116.4° (1 g / 100 mL chloroform)

(4*S*,4'*S*,4''*S*)-2,2',2''-(Propane-1,2,2-triyl)tris(4-cyclohexyl-4,5-dihydrooxazole) ((*S*)-CyTox) (L5)

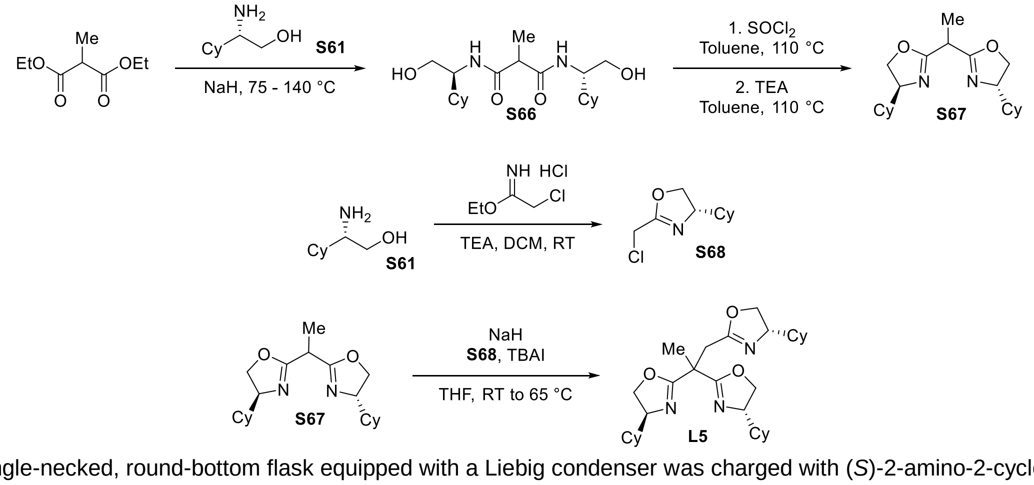

A 25 mL, single-necked, round-bottom flask equipped with a Liebig condenser was charged with (*S*)-2-amino-2-cyclohexylethan-1-ol (**S61**) (1.0 g, 7.0 mmol, 2.1 equiv.), diethyl methylmalonate (0.55 mL, 3.3 mmol, 1.0 equiv.) and a tip of a spatula of NaH (60% dispersion in mineral oil). The mixture was heated under stirring up to 75-80 °C: the solids melted down to form a yellow, oily mixture, which then converted into an off-white, solid mass (overall, this took ca. 15 minutes). Heating was continued up to 140 °C for overall 1 hour (stirring became increasingly difficult and was finally stopped a few minutes after the formation of the solid). Heating was then stopped, and the crude solid was allowed to cool down to room temperature. It was submitted to column chromatography (SiO_2; DCM/MeOH, 24/1 to 23/2) to afford N^1,N^3-bis((*S*)-1-cyclohexyl-2-hydroxyethyl)-2-methylmalonamide (**S66**) (0.95 g, 2.6 mmol, 77% yield) as a white powder.

^{1}H NMR (400 MHz, DMSO-d_6) δ 7.61 (d, *J* = 9.1 Hz, 1H, (CO)N*H*), 7.49 (d, *J* = 9.2 Hz, 1H, (CO)N*H*), 4.59 (dt, *J* = 11.0, 5.3 Hz, 2H, NC*H*Cy), 3.62 – 3.52 (m, 2H, C*H₂*OH), 3.48 – 3.26 (m, 2H, C*H₂*OH), 3.20 (q, *J* = 7.0 Hz, 1H, C*H*Me) 3.17 (m, 1H, O*H*), 3.16 (m, 1H, O*H*), 1.74 – 1.41 (m, 10H, Cy*H*), 1.48 (td, *J* = 7.7, 3.6 Hz, 2H, Cy*H*), 1.18 (d, *J* = 7.0 Hz, 3H, CH*Me*), 1.16 – 0.83 (m, 10H, Cy*H*).

Following a slightly modified version of a reported procedure,[23] In a sealed 25 mL, round-bottom vial, N^1,N^3-bis((*S*)-1-cyclohexyl-2-hydroxyethyl)-2,2-dimethylmalonamide (**S66**) (0.80 g, 2.2 mmol, 1.0 equiv.) was suspended in toluene (18.7 mL). To the resulting suspension, thionyl chloride (0.48 mL, 6.5 mmol, 3.0 equiv.) was added by syringe at room temperature, leading to the dissolution of the solid. The clear solution was then heated at 110 °C (refluxed) for 5 hours, darkening from colorless to pale yellow, and finally to orange during this time. It was then allowed to cool down to room temperature, diluted with chloroform (30 mL), and poured onto water. After the separation of the organic layer, the aqueous one was extracted with chloroform (3 x 30 mL). The combined organic layers were washed with sat. aq. K_2CO_3 (15 mL), brine, dried over Na_2SO_4, filtered, and concentrated under vacuum. The resulting pale-yellow solid was triturated with pentane. The yellow filtrate was discarted. N^1,N^3-bis((*S*)-2-chloro-1-cyclohexylethyl)-2,2-dimethylmalonamide (0.686 g, 1.69 mmol, 78% yield) was collected as an off-white powder, which was not further purified.

In a sealed 25 mL, round-bottom vial, N^1,N^3-bis((*S*)-2-chloro-1-cyclohexylethyl)-2,2-dimethylmalonamide (0.320 g, 0.789 mmol, 1.0 equiv.) was dissolved in toluene (dry; 10.3 mL). Et_3N (dry; 1.2 mL, 8.5 mmol, 10 equiv.) was then added by syringe, and the resulting mixture was stirred at room temperature overnight and then at 110 °C for 20 hours. This resulted in the conversion of the initially clear, pale yellow solution into a yellow-orange suspension. The mixture was allowed to cool down to room temperature, diluted with chloroform (30 mL) and washed with sat. aq. NH_4Cl (30 mL). The aqueous layer was extracted with chloroform (3 x 20 mL), and the combined organic layers were washed with brine, dried over $MgSO_4$, filtered, and concentrated under vacuum. The pale orange crude oil was submitted to column chromatography (Biotage flash chromatographer, 12 SiO_2; MeOH in DCM, 0 to 13%) to furnish (4*S*,4'*S*)-2,2'-(ethane-1,1-diyl)bis(4-cyclohexyl-4,5-dihydrooxazole) (**S67**) (0.253 g, 0.761 mmol, 96% yield) as a yellow, viscous oil.

^{1}H NMR (400 MHz, Chloroform-*d*) δ 4.22 (dd, *J* = 9.7, 8.2 Hz, 2H, NC*H*Cy), 4.01 (td, *J* = 8.0, 2.3 Hz, 2H, C*H₂*O), 3.92 (dtd, *J* = 9.9, 6.9, 3.3 Hz, 2H, C*H₂*O), 3.52 (q, *J* = 7.3 Hz, 1H, C*H*Me), 1.89 – 1.55 (m, 10H, Cy*H*), 1.47 (d, *J* = 7.3 Hz, 3H, CH*Me*), 1.42 (ddd, *J* = 11.8, 6.3, 3.1 Hz, 2H, Cy*H*), 1.30 – 1.09 (m, 6H, Cy*H*), 0.99 (pd, *J* = 12.3, 10.7, 2.6 Hz, 4H, Cy*H*).
^{1}H-NMR data corresponded to the reported values.[25]

Following a reported procedure,[26] in a 50 mL, two-necked, round-bottom flask, (2*S*)-2-amino-2-cyclohexylethanol (**S61**) (0.600 g, 4.20 mmol, 1.1 equiv.) and ethyl 2-chloroethanimidoate hydrochloride (0.600 g, 3.80 mmol, 1.0 equiv.) were suspended in DCM (13 mL) at room temperature. Triethylamine (0.64 mL, 4.6 mmol, 1.2 equiv.) was added to the resulting pink suspension: the suspension darkened to violet. The mixture was stirred at room temperature overnight. The volatiles were then removed under reduced pressure to give a dark paste, which was partitioned between sat. aq. NH_4Cl (20 mL) and EtOAc (20 mL). Upon separation, the aqueous layer was further extract with EtOAc (3 x 15 mL). The combined organic layers were washed with brine, dried over $MgSO_4$, filtered, and concentrated under vacuum. The resulting dark purple, crude oil was submitted to column chromatography (SiO_2; pentane/acetone, 19/1 to 7/3) to give (*S*)-2-(chloromethyl)-4-cyclohexyl-4,5-dihydrooxazole (**S68**) (0.530 g, 2.63 mmol, 69% yield) as a colorless oil.
^{1}H NMR (400 MHz, Chloroform-*d*) δ 4.37 (dd, *J* = 9.8, 8.4 Hz, 1H, C*H₂*O), 4.19 – 4.07 (m, 3H, C*H₂*O and C*H₂*Cl), 3.98 (ddd, *J* = 9.7, 8.4, 6.7 Hz, 1H, NC*H*Cy), 1.90 (dpd, *J* = 10.5, 3.4, 2.3 Hz, 1H, Cy*H*), 1.82 – 1.74 (m, 2H, Cy*H*), 1.70 (dtd, *J* = 9.0, 3.0, 1.5 Hz, 1H, Cy*H*), 1.64 – 1.55 (m, 1H, Cy*H*), 1.47 (tdt, *J* = 11.7, 6.7, 3.1 Hz, 1H, Cy*H*), 1.35 – 1.15 (m, 3H, Cy*H*), 1.14 – 0.92 (m, 2H, Cy*H*).

Following a slightly modified version of a reported procedure,[27] a 25 mL, round-bottom vial was charged with (4*S*,4'*S*)-2,2'-(ethane-1,1-diyl)bis(4-cyclohexyl-4,5-dihydrooxazole) (**S67**) (0.273 g, 0.861 mmol, 1.0 equiv.), followed by THF (6.6 mL). To the resulting solution was added NaH (60% dispersion in mineral oil; 98 mg, 2.5 mmol, 3.0 equiv.). The turbid, off-white mixture was stirred at room temperature for 60 minutes. TBAI (45 mg, 0.12 mmol, 15 mol%) was added, the vial was sealed with a PTFE cap, the mixture was then cooled to 0 °C (ice - water bath), and a (*S*)-2-(chloromethyl)-4-cyclohexyl-4,5-dihydrooxazole (**S68**) (0.331 g, 1.64 mmol, 2.0 equiv.) in THF (1.6 mL) was added slowly. The mixture was then heated to 65 °C. After 18 at this temperature, the mixture was allowed to cool down to room temperature, and poured into water/sat. aq. NH_4Cl (40 / 10 mL). The aqueous layer was extracted with DCM (4 x 15 mL). The combined organic layers were washed with brine, dried over Na_2SO_4, filtered, and concentrated under vacuum. The resulting pale yellow, crude oil was submitted to column chromatography (Biotage flash chromatographer, 4 g SiO_2; EtOAc in DCM, 20 to 100%) to provide (4*S*,4'*S*,4''*S*)-2,2',2''-(propane-1,2,2-triyl)tris(4-cyclohexyl-4,5-dihydrooxazole) (**L5**, (*S*)-CyTox) (0.180 g, 0.361 mmol, 44% yield) as a pale-yellow oil, which became an off-white solid on standing at 4 °C.

^{1}H NMR (400 MHz, Chloroform-*d*) δ 4.22 (ddd, *J* = 11.6, 9.7, 8.2 Hz, 2H, C*H₂*), 4.14 (m, 1H, C*H*), 4.04 (dt, *J* = 10.0, 7.6 Hz, 2H, C*H₂*), 3.93 (ddt, *J* = 9.7, 7.9, 6.0 Hz, 2H, C*H₂*), 3.85 (q, *J* = 8.3, 7.8 Hz, 2H, C*H₂*), 3.08 (d, *J* = 14.7 Hz, 1H, C*H₂*Ox), 2.97 (d, *J* =

14.7 Hz, 1H, C*H₂*Ox), 1.93 – 1.65 (m, 12H, Cy*H*), 1.61 (s, 3H, *Me*), 1.52 – 1.34 (m, 2H, Cy*H*), 1.33 – 1.11 (m, 10H, Cy*H*), 1.08 – 0.93 (m, 6H, Cy*H*).
^{1}H-NMR data corresponded to the reported values.[25]

(4*S*,4'*S*)-2,2'-(Propane-2,2-diyl)bis(4-cyclopentyl-4,5-dihydrooxazole) ((*S*)-CypBox) (L13)

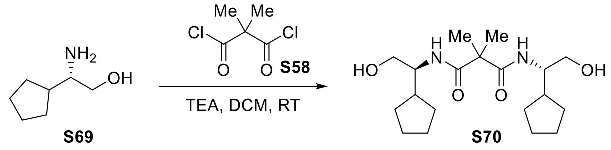

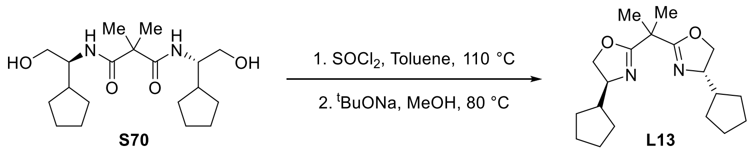

Following a slightly modified version of a reported procedure,[23] in a 25 mL, single-necked, round-bottom flask, (*S*)-2-amino-2-cyclopentylethan-1-ol (**S69**) (0.250 g, 1.93 mmol, 2.1 equiv.) was suspended in DCM (7 mL). The suspension was cooled to 0 °C (ice - water bath), prior to the addition of triethylamine (0.32 mL, 2.3 mmol, 2.5 equiv.). Under stirring, a solution of dimethylmalonyl dichloride (**S58**) (0.12 mL, 0.91 mmol, 1.0 equiv.) in DCM (2.0 mL) was added slowly, at the same temperature. After the addition, the mixture was stirred at room temperature overnight, turning into a clear, yellow solution. After 23 hours, the mixture was diluted with DCM (10 mL), and the organic solution was washed with sat. aq. $NaHCO_3$ (10 mL). The aqueous layer was extracted with DCM (3 x 15 mL). The combined organic layers were washed with brine, dried over Na_2SO_4, filtered, and concentrated under vacuum. The resulting pinkish crude solid was purified by column chromatography (SiO_2; DCM/MeOH 194/4 to 190/10) to afford N^1,N^3-bis((*S*)-1-cyclopentyl-2-hydroxyethyl)-2,2-dimethylmalonamide (**S70**) (0.225 g, 0.635 mmol, 70% yield) as an off-white, foamy solid.

Following a slightly modified version of a reported procedure,[23] N^1,N^3-bis((*S*)-1-cyclopentyl-2-hydroxyethyl)-2,2-dimethylmalonamide (**S70**) (0.210 g, 0.592 mmol, 1.0 equiv.) was dissolved in toluene (6 mL). At room temperature, thionyl chloride (0.11 mL, 1.5 mmol, 2.5 equiv.) was added drop-wise by syringe. The colorless, clear mixture was then heated to reflux (110 °C). Stirring was continued for 2.5 hours. The mixture was then allowed to cool down to room temperature, and it was concentrated under reduced pressure in order to remove the solvent and the excess of thionyl chloride. The solid residue was then partitioned between DCM (15 mL) and sat. aq. $NaHCO_3$ (10 mL). The aqueous layer was extracted with DCM (2 x 15 mL). The combined organic layers were washed brine, dried over Na_2SO_4, filtered, and concentrated under vacuum to provide a yellow, viscous oil. Trituration with pentane yielded N^1,N^3-bis((*S*)-2-chloro-1-cyclopentylethyl)-2,2-dimethylmalonamide (0.17 g, 0.43 mmol, 73% yield) as off-white solid, which was used in the following step without further purification.

Following a reported procedure,[23] inside a glove-box, a 25 mL round-bottom vial was charged with N^1,N^3-bis((*S*)-2-chloro-1-cyclopentylethyl)-2,2-dimethylmalonamide (0.16 g, 0.42 mmol, 1.0 equiv.) and sodium *tert*-butoxide (0.12 g, 1.3 mmol, 3.0 equiv.), and subsequently sealed with a PTFE cap. The vial was taken out of the glove-box, and MeOH (dry; 4.0 mL) was added by syringe. The resulting yellow, turbid mixture was heated to 80 °C for 16 hours. After this time, the reaction was stopped, and the mixture was concentrated under reduced pressure to give a pale-yellow paste. The latter was dissolved in DCM (ca. 15 mL), and washed with a solution of brine (ca. 15 mL) and water (ca. 15 mL). Upon separation, the aqueous layer was extracted with DCM (4 x 15 mL). All the organic layers were then combined, and washed with brine, dried over $MgSO_4$, filtered, and concentrated under vacuum. The resulting crude oil was dissolved in MeOH (dry; 4.0 mL); the so-obtained solution was transferred into a 25 mL, round-bottomed vial, sodium *tert*-butoxide (0.12 g, 1.3 mmol, 3.0 equiv.) was added, and - after sealing the vial with a PTFE septum - the mixture was stirred at 80 °C for another 20 hours. After this time, TLC analysis showed that the only trace amounts of the starting material were present. The previously described work-up procedure was implemented again. The resulting crude oil was then submitted to column chromatography (SiO_2; Pentane/acetone 19/1 to 16/4) to provide (4*S*,4'*S*)-2,2'-(propane-2,2-diyl)bis(4-cyclopentyl-4,5-dihydrooxazole) (**L13**, (*S*)-CypBox) (0.101 g, 0.317 mmol, 75% yield) as a pale yellow oil.

^{1}H NMR (400 MHz, Chloroform-*d*) δ 4.24 (ddt, *J* = 9.2, 8.0, 1.3 Hz, 2H, C*H₂*O), 4.13 – 4.02 (m, 2H, C*H*N), 3.96 (ddt, *J* = 8.0, 6.9, 1.3 Hz, 2H, C*H₂*O), 2.07 – 1.92 (m, 2H, Cyp*H*), 1.80 – 1.70 (m, 2H, Cyp*H*), 1.70 – 1.51 (m, 10H, Cyp*H*), 1.50 (d, *J* = 1.5 Hz, 6H, *Me*), 1.36 – 1.19 (m, 4H, Cyp*H*).
^{13}C NMR (101 MHz, Chloroform-*d*) δ 168.9, 71.5, 70.0, 44.7, 38.6, 28.8, 28.4, 25.5, 25.5, 24.5.
HRMS (ESI/QTOF) m/z: $[M + H]^+$ Calcd for $C_{19}H_{31}N_2O_2^+$ 319.2380; Found 319.2377.
$[\alpha]_D^{20}$ = -119.2° (1.09 g/ 100 mL chloroform)
As a reference, literature known (*R*)-CypBox has $[\alpha]_D^{21}$ = +119.9° (0.97 g/100 mL DCM).[28]

(4*S*,4'*S*,4''*S*)-2,2',2''-(Propane-1,2,2-triyl)tris(4-benzyl-4,5-dihydrooxazole) ((*S*)-BnTox) (L14)

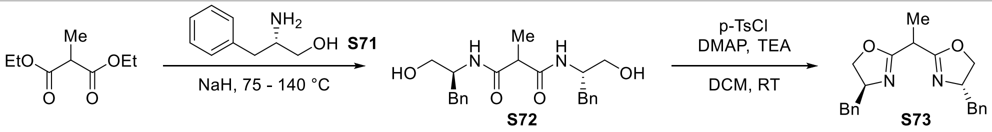

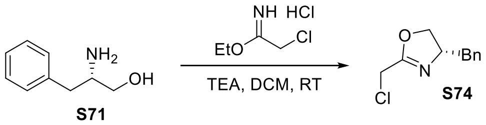

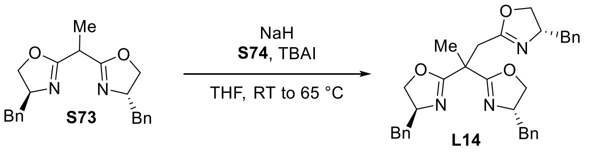

A 25 mL, single-necked, round-bottom flask equipped with a Liebig condenser was charged with (*S*)-2-amino-3-phenylpropan-1-ol (**S71**) (1.01 g, 6.68 mmol, 2.1 equiv.), diethyl methylmalonate (0.55 mL, 3.19 mmol, 1.0 equiv.), and a tip of a spatula of NaH (60% dispersion in mineral oil). The mixture was heated under stirring up to 75-80 °C: the solids melted down to form a yellow, oily mixture, which then converted into an off-white, solid mass (overall, this took ca. 15 minutes). The latter was submitted to vacuum in order to remove the formed EtOH. The solid was heated again to 140 °C for 1 hour. It was then allowed to cool down to room temperature. Recrystallization from EtOAc/EtOH (3 mL / 4 mL) gave N^1,N^3-bis((*S*)-1-hydroxy-3-phenylpropan-2-yl)-2-methylmalonamide (**S72**) (0.916 g, 2.38 mmol, 75% yield) as a white solid.

^{1}H NMR (400 MHz, DMSO-d_6; the OH signals are not resolved) δ 7.74 (d, *J* = 8.5 Hz, 1H, (CO)N*H*), 7.69 (d, *J* = 8.3 Hz, 1H, (CO)N*H*), 7.29 – 7.20 (m, 4H, Ph*H*), 7.22 – 7.13 (m, 6H, Ph*H*), 4.81 (dt, *J* = 16.8, 5.4 Hz, 2H, NC*H*Bn), 3.89 (d, *J* = 7.7 Hz, 2H, CH_2OH), 3.30 (m, 2H, CH_2OH), 3.04 (q, *J* = 7.2 Hz, 1H, C*H*Me), 2.80 (ddd, *J* = 16.5, 13.4, 6.0 Hz, 2H, CH_2Ph), 2.69 – 2.54 (m, 2H, CH_2Ph), 1.02 (d, *J* = 7.2 Hz, 3H, CH*Me*).

Following a reported procedure,[29] a 50 mL, single-necked, round-bottom flask was charged with N^1,N^3-bis((*S*)-1-hydroxy-3-phenylpropan-2-yl)-2-methylmalonamide (**S72**) (0.769 g, 2.00 mmol, 1.0 equiv.), DMAP (49 mg, 0.40 mmol, 20 mol%) and DCM (10 mL). Under stirring, triethylamine (1.4 mL, 10 mmol, 5.0 equiv.) was added by syringe. Finally, a solution of *p*-tosyl chloride (0.76 g, 4.0 mmol, 2.0 equiv.) in DCM (5.0 mL) was added drop-wise. The resulting pale-yellow suspension was then stirred for 3 days and 17 hours at room temperature; after this time, it looked like a yellow solution. The latter was then diluted with DCM (20 mL), and the organic solution was washed with sat. aq. NH_4Cl (20 mL). The aqueous layer was extracted with DCM (3 x 25 mL). The combined organic layers were washed with sat. aq. $NaHCO_3$ (20 mL), brine, dried over Na_2SO_4, filtered, and concentrated under vacuum. The residue was submitted to column chromatography (SiO_2; pentane/acetone 170/30 to 150/50) to furnish (4*S*,4'*S*)-2,2'-(ethane-1,1-diyl)bis(4-benzyl-4,5-dihydrooxazole) (**S73**) (0.229 g, 0.657 mmol, 33% yield) as a pale yellow, viscous oil.

^{1}H NMR (400 MHz, Chloroform-*d*) δ 7.34 – 7.26 (m, 4H, Ph*H*), 7.27 – 7.20 (m, 6H, Ph*H*), 4.45 (m, 2H, NC*H*Bn), 4.23 (td, *J* = 8.9, 3.7 Hz, 2H, CH_2O), 4.03 (ddd, *J* = 8.5, 7.1, 1.2 Hz, 2H, CH_2O), 3.50 (q, *J* = 7.2 Hz, 1H, C*H*Me), 3.14 (dd, *J* = 13.7, 5.1 Hz, 2H, CH_2Ph), 2.69 (dd, *J* = 13.7, 8.6 Hz, 2H, CH_2Ph), 1.48 (d, *J* = 7.2 Hz, 3H, CH*Me*).
^{1}H-NMR data corresponded to the reported values.[30]

Following a reported procedure,[26] in a 25 mL, two-necked, round-bottom flask, (*S*)-2-amino-3-phenylpropan-1-ol (**S71**) (0.287 g, 1.82 mmol, 1.1 equiv.) and ethyl 2-chloroethanimidoate hydrochloride (0.25 g, 1.6 mmol, 1.0 equiv.) were suspended in DCM (dry; 5.5 mL) at room temperature. Triethylamine (0.25 mL, 1.8 mmol, 1.1 equiv.) was added to the milky suspension: the suspension became less turbid at the beginning, and then precipitation started occurring with the colour turning from yellow to pink. The mixture was stirred at room temperature for 6 hours. The volatiles were then removed under reduced pressure to give a pinkish paste, which was partitioned between water (20 mL) and EtOAc (20 mL). Upon separation, the aqueous layer was further extract with EtOAc (3 x 15 mL). The combined organic layers were washed with brine, dried over $MgSO_4$, filtered, and concentrated under vacuum. The resulting brownish, crude oil was submitted to column chromatography (Biotage flash chromatographer, 4 g SiO_2; EtOAc in pentane, 5 to 35%) to give (*S*)-4-benzyl-2-(chloromethyl)-4,5-dihydrooxazole (**S74**) (0.260 g, 1.24 mmol, 75% yield) as a pale-yellow oil.

^{1}H NMR (400 MHz, Chloroform-*d*) δ 7.34 – 7.28 (m, 2H, Ph*H*), 7.26 – 7.18 (m, 3H, Ph*H*), 4.53 – 4.41 (m, 1H, NC*H*Bn), 4.31 (dd, *J* = 9.5, 8.5 Hz, 1H, CH_2O), 4.13 – 4.04 (m, 3H, CH_2O and CH_2Cl), 3.12 (dd, *J* = 13.8, 5.5 Hz, 1H, CH_2Ph), 2.71 (dd, *J* = 13.8, 8.3 Hz, 1H, CH_2Ph).
^{1}H-NMR data corresponded to the reported values.[26]

Following a slightly modified version of a reported procedure,[27] a 25 mL, round-bottom vial was charged with (4*S*,4'*S*)-2,2'-(ethane-1,1-diyl)bis(4-benzyl-4,5-dihydrooxazole) (**S73**) (0.195 g, 0.560 mmol, 1.0 equiv.), followed by THF (4.5 mL). To the resulting solution was added NaH (60% dispersion in mineral oil; 67 mg, 1.7 mmol, 3.0 equiv.). The turbid, off-white mixture was stirred at room temperature for 60 minutes. TBAI (31 mg, 0.084 mmol, 15 mol%) was added, the vial was sealed with a PTFE cap, the mixture was then cooled to 0 °C (ice - water bath), and a solution of (*S*)-4-benzyl-2-(chloromethyl)-4,5-dihydrooxazole (**S74**) (0.188 g, 0.895 mmol, 1.6 equiv.) in THF (1.1 mL) was added slowly. The mixture was then heated to 65 °C. After 18 at this temperature, the mixture was allowed to cool down to room temperature, and poured into water/sat. aq. NH_4Cl (40 / 10 mL). The aqueous layer was extracted with DCM (4 x 15 mL). The combined organic layers were washed with brine, dried over Na_2SO_4, filtered, and concentrated under vacuum. The resulting pale yellow, crude oil was submitted to column chromatography (Biotage flash chromatographer, 4 g SiO_2; EtOAc in DCM, 20 to 100%) to provide (4*S*,4'*S*,4''*S*)-2,2',2''-(propane-1,2,2-triyl)tris(4-benzyl-4,5-dihydrooxazole) (**L14**, (*S*)-BnTox) (0.224 g, 0.429 mmol, 77% yield) as a pale yellow oil.

^{1}H NMR (400 MHz, $CDCl_3$) δ 7.32 – 7.26 (m, 6H, Ph*H*), 7.25 – 7.14 (m, 9H, Ph*H*), 4.46 – 4.29 (m, 3H, NC*H*Bn), 4.18 (ddd, *J* = 9.3, 8.5, 6.0 Hz, 2H, C*H₂*O), 4.12 (dd, *J* = 9.4, 8.5 Hz, 1H, C*H₂*O), 4.02 (dt, *J* = 8.4, 6.9 Hz, 2H, C*H₂*O), 3.92 (dd, *J* = 8.5, 7.3 Hz, 1H, C*H₂*O), 3.10 (ddd, *J* = 18.6, 13.7, 5.0 Hz, 3H, C*H₂*Ph), 3.02 (dd, *J* = 14.8, 0.8 Hz, 1H, C*H₂*Ox), 2.97 (dd, *J* = 14.4, 0.8 Hz, 1H, C*H₂*Ox), 2.71 – 2.52 (m, 3H, C*H₂*Ph), 1.56 (s, 3H, *Me*).
^{13}C NMR (101 MHz, Chloroform-*d*; the signals corresponding to 3 aromatic C are not resolved) δ 167.7, 167.5, 164.2, 138.0, 137.7, 129.5, 129.3, 129.2, 128.6, 128.5, 128.5, 126.5, 72.5, 72.2, 71.6, 67.5, 67.4, 67.1, 41.8, 41.5, 41.2, 40.9, 35.0, 21.2.
HRMS (ESI/QTOF) m/z: $[M + H]^+$ Calcd for $C_{33}H_{36}N_3O_3^+$ 522.2751; Found 522.2748.
$[\alpha]_D^{20}$ = -21.6° (1 g/ 100 mL chloroform)

(4*R*,4'*R*)-2,2'-(Propane-2,2-diyl)bis(4-benzhydryl-4,5-dihydrooxazole) ((*R*)-BzhBox) (L17)

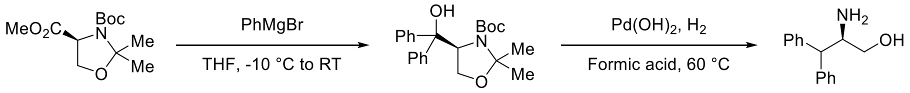

S75 **S76** **S77**

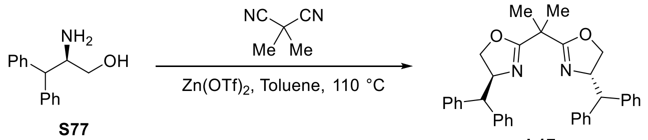

S77 **L17**

Following a modified version of a reported procedure,[31] in a 100 mL, two-necked, round-bottom flask, methyl-(*S*)-3-Boc-2,2-dimethyl-4-oxazolidincarboxylate (**S75**) (1.5 mL, 6.3 mmol, 1.0 equiv.) was dissolved in THF (dry; 54 mL). The resulting colorless solution was chilled to -10 °C (ice - brine bath). Phenylmagnesium bromide (3.0 M in Et_2O; 8.4 mL, 25 mmol, 4.0 equiv.) was then added drop-wise over a period of 10 minutes. The mixture was allowed to warm to room temperature, and it was stirred for 3 hours. The reaction was then quenched by pouring the mixture onto ice in sat. aq. NH_4Cl (50 mL). The aqueous layer was extracted with Et_2O (3 x 50 mL). The combined organic layers were washed with brine, dried over $MgSO_4$, filtered, and concentrated under vacuum. The resulting yellow crude oil was submitted to column chromatography (Biotage flash chromatographer, 40 g SiO_2; EtOAc in pentane, 5 to 30%) to furnish *tert*-butyl (*S*)-4-(hydroxydiphenylmethyl)-2,2-dimethyloxazolidine-3-carboxylate (**S76**) (1.58 g, 4.11 mmol, 66% yield) as a colorless oil (which was not submitted to analysis).

Following a reported procedure,[32] a 250 mL Schlenk flask was charged with *tert*-butyl (*S*)-4-(hydroxydiphenylmethyl)-2,2-dimethyloxazolidine-3-carboxylate (**S76**) (1.58 g, 4.11 mmol, 1.0 equiv.) and $Pd(OH)_2$ (20% w/w in Pd; 0.63 g, 40% of the substrate mass). The flask was closed with a septum, and evacuated. Formic acid (82 mL) was added. Under stirring, the black suspension was flushed with nitrogen for 5 minutes. After this, it was further flushed with hydrogen (using a balloon and a long needle) for 10 minutes. The needle was then lifted up in order to maintain the mixture under a hydrogen atmosphere, while being stirred under heating at 60 °C. After 16 hours, the mixture was allowed to cool down to room temperature. The solids were filtered off through a pad of celite, which was then washed with DCM (100 mL overall). Water (82 mL) was added. The aqueous layer was extracted with DCM (3 x 75 mL). The combined organic layers were then washed with sat. aq. $NaHCO_3$ until neutral pH; they were then dried over Na_2SO_4, filtered, and concentrated under vacuum. The pale yellow-grey, oily residue was then treated with water (50 mL), MeOH (50 mL), NaOH (1.50 g). The resulting reaction mixture was refluxed at 80 °C under air for 12 hours. It was then cooled to room temperature and concentrated under vacuum. The residual aqueous layer was extracted three times with a 3:1 mixture of $CHCl_3$: ^{i}PrOH. The organic layers were dried over $MgSO_4$, filtered and concentrated *in vacuo*. The crude amino alcohol was purified by recrystallization from hexane/EtOAc. (*S*)-2-Amino-1,1-diphenylpropane-1,3-diol (**S77**) (0.476 g, 2.09 mmol, 51% yield) was obtained as a white powder.

^{1}H NMR (400 MHz, Chloroform-*d*) δ 7.39 – 7.26 (m, 8H, Ph*H*), 7.25 – 7.14 (m, 2H, Ph*H*), 3.78 (d, *J* = 10.5 Hz, 1H, Ph_2CH), 3.67 (ddd, *J* = 10.4, 6.7, 3.3 Hz, 1H, C*H*NH_2), 3.55 (dd, *J* = 10.7, 3.4 Hz, 1H, C*H₂*OH), 3.29 (dd, *J* = 10.7, 6.7 Hz, 1H, C*H₂*OH), 1.53 (br s, 3H, O*H* and N*H₂*).

^{1}H-NMR data corresponded to the reported values.[33]

Following a reported procedure,[34] a 5 mL round-bottom vial was charged with 2,2-dimethylmalononitrile (0.023 g, 0.24 mmol, 1.0 equiv.) and Zinc(II) triflate (89 mg, 0.24 mmol, 1.0 equiv.). The vial was capped with a PTFE septum, and it was evacuated and backfilled with nitrogen (3 times). Toluene (dry; 2.5 mL) was added, and the resulting pale yellow, clear solution was stirred at room temperature for 5 minutes. (S)-2-Amino-1,1-diphenylpropane-1,3-diol (**S77**) (0.111 g, 0.489 mmol, 2.0 equiv.) was then added. The resulting mixture was refluxed (115 °C) for 3 days and 19 hours. It was then allowed to cool down to room temperature, and diluted with DCM (15 mL). The organic layer was washed with sat. aq. $NaHCO_3$. The aqueous layer was extracted with DCM (3 x 10 mL). The combined organic layers were washed with brine, dried over $MgSO_4$, filtered, and concentrated under reduced pressure. The resulting crude oil was submitted to preparative TLC (20 x 20 sq cm SiO_2 plate; elution with pentane/acetone 8/2) to provide (4R,4'R)-2,2'-(propane-2,2-diyl)bis(4-benzhydryl-4,5-dihydrooxazole) (**L17**, (R)-BzhBox) (0.084 g, 0.16 mmol, 67% yield) as a foamy, white solid.

^{1}H NMR (400 MHz, Chloroform-*d*) δ 7.27 (d, *J* = 4.4 Hz, 8H, Ph*H*), 7.25 – 7.14 (m, 12H, Ph*H*), 4.88 (dt, *J* = 9.7, 7.1 Hz, 2H, C*H*N), 4.27 (dd, *J* = 9.6, 8.6 Hz, 2H, C*H*$_2$O), 4.05 – 4.00 (m, 4H, C*H*$_2$O and Ph_2C*H*), 1.28 (s, 6H, *Me*).
^{1}H-NMR data corresponded to the reported values.[35]

(4R,4'R)-2,2'-(propane-2,2-diyl)bis(4-(naphthalen-2-ylmethyl)-4,5-dihydrooxazole) ((*R*)-NpCH$_2$Box) (L18)

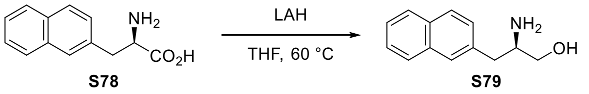

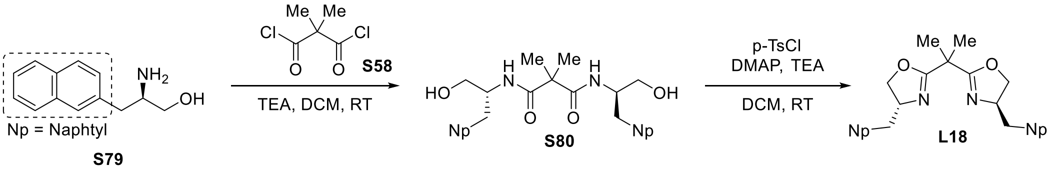

Following a reported procedure,[36] a 100 mL, two-necked, round bottomed flask equipped with a Liebig condenser was charged with LAH (0.441 g, 11.6 mmol, 5.0 equiv.). THF (dry; 15 mL) was added, and the resulting grey suspension was cooled to 0 °C (ice - water bath). (*R*)-2-Amino-3-(naphthalen-2-yl)propanoic acid (**S78**) (0.50 g, 2.3 mmol, 1.0 equiv.) was added as a solid in small portions (under a nitrogen stream). Gas release was observed. Once the addition was completed, the mixture was stirred at reflux (65 °C) overnight. After 16 hours, the mixture was cooled back to 0 °C, and the reaction was quenched by sequential addition of water (0.40 mL), aq. NaOH (15% w/w; 0.4 mL) and water (1.2 mL). Vigorous gas evolution occurred. The suspension was stirred at room temperature for 40 minutes, turning from grey to white. $MgSO_4$ was then added, and stirring continued for another 20 minutes. Finally, the solids were filtered off through a plug of celite, which was then washed with several portions of EtOAc. The filtrate was concentrated under reduced pressure to provide (*R*)-2-amino-3-(naphthalen-2-yl)propan-1-ol (**S79**) (0.429 g, 2.13 mmol, 92% yield) as a very pale-yellow solid, which was used in the following steps without further purification.

Following a slightly modified version of a reported procedure,[23] in a 10 mL, two-necked, round-bottomed flask, (*R*)-2-amino-3-(naphthalen-2-yl)propan-1-ol (**S79**) (0.234 g, 1.16 mmol, 2.05 equiv.) was suspended in DCM (dry; 2.2 mL). The mixture was cooled to 0 °C (ice - water bath), prior to the addition of triethylamine (0.20 mL, 1.4 mmol, 2.5 equiv.). A solution of dimethyl malonyl dichloride (**S58**) (0.075 mL, 0.57 mmol, 1.0 equiv.) in DCM (0.4 mL) was then added drop-wise at the same temperature. The solid was completely dissolved; after a few minutes, some homogeneous precipitation was observed. The mixture was stirred at 0 °C for 30 minutes. The cooling bath was afterwards removed and stirring was continued at room temperature overnight. After 18 hours, the reaction was quenched by addition of water (3.0 mL). The aqueous layer was extracted with DCM (4 x 15 mL). The combined organic layers were washed with brine, dried over Na_2SO_4, filtered, and concentrated under reduced pressure. The resulting pale yellow, sticky, crude oil was submitted to column chromatography (Biotage flash chromatographer, 4 g SiO_2; MeOH in DCM, 1 to 15%). The so obtained product was triturated at 60 °C with acetone and hexane, followed by collection by filtration of the precipitate. N^1,N^3-Bis((*R*)-1-hydroxy-3-(naphthalen-2-yl)propan-2-yl)-2,2-dimethylmalonamide (**S80**) (0.187 g, 0.375 mmol, 66% yield) was obtained as a yellow-greyish solid.

^{1}H NMR (400 MHz, Chloroform-*d*) δ 7.80 (t, *J* = 8.5 Hz, 6H, Np*H*), 7.61 (s, 2H, Np*H*), 7.46 (ddd, *J* = 6.6, 4.0, 1.7 Hz, 4H, Np*H*), 7.36 – 7.29 (m, 2H, Np*H*), 6.48 (d, *J* = 8.2 Hz, 2H, (CO)N*H*), 4.29 (m, 2H, C*H*NH), 3.76 (dd, *J* = 11.4, 3.4 Hz, 2H, C*H*$_2$OH), 3.51 (dd, *J* = 11.3, 5.9 Hz, 2H, C*H*$_2$OH), 2.94 (dd, *J* = 14.0, 6.9 Hz, 2H, C*H*$_2$Np), 2.88 (dd, *J* = 13.9, 7.7 Hz, 2H, C*H*$_2$Np), 1.62 (br s, 2H, O*H*), 1.23 (s, 6H, *Me*).

Following a protocol inspired by a reported procedure,[29] inside a 10 mL, flat-bottom vial capped with a septum and filled with nitrogen, diamide N^1,N^3-bis((*R*)-1-hydroxy-3-(naphthalen-2-yl)propan-2-yl)-2,2-dimethylmalonamide (**S80**) (0.187 g, 0.375 mmol, 1.0 equiv.) and DMAP (10 mg, 0.081 mmol, 22 mol%) were dissolved in DCM (dry; 1.5 mL). Triethylamine (0.30 mL, 2.2 mmol,

5.8 equiv.) was added under stirring. Finally, a solution of *p*-tosyl chloride (0.16 g, 0.84 g, 2.2 equiv.) in DCM (0.4 mL) was also added drop-wise. The mixture became a yellowish solution, which was stirred at room temperature for 4 days. After this time, the mixture was diluted with DCM, and the reaction was quenched by the addition of water. The aqueous layer was extracted with DCM (3 x 15 mL). The combined organic layers were washed with brine, dried over Na_2SO_4, filtered and concentrated under vacuum. The resulting crude product was submitted to column chromatography (Biotage flash chromatographer, 12 g SiO_2; MeOH in DCM, 1 to 12%) to provide (4*R*,4'*R*)-2,2'-(propane-2,2-diyl)bis(4-(naphthalen-2-ylmethyl)-4,5-dihydrooxazole) (**L18**, (*R*)-$NpCH_2Box$) (0.110 g, 0.238 mmol, 63% yield) as a pale yellow solid.

^{1}H NMR (400 MHz, Chloroform-*d*) δ 7.85 – 7.76 (m, 6H, Np*H*), 7.65 – 7.57 (m, 2H, Np*H*), 7.50 – 7.39 (m, 4H, Np*H*), 7.33 (dd, *J* = 8.4, 1.8 Hz, 2H, Np*H*), 4.49 (tdd, *J* = 8.5, 6.9, 4.7 Hz, 2H, C*H*N), 4.16 (t, *J* = 8.9 Hz, 2H, C*H*$_2$O), 4.00 (dd, *J* = 8.6, 6.9 Hz, 2H, C*H*$_2$O), 3.23 (dd, *J* = 13.7, 4.8 Hz, 2H, C*H*$_2$Np), 2.82 (dd, *J* = 13.8, 8.4 Hz, 2H, C*H*$_2$Np), 1.47 (s, 6H, *Me*).
^{1}H-NMR data corresponded to the reported values.[36]

(4S,4'S)-2,2'-(propane-2,2-diyl)bis(4-(cyclohexylmethyl)-4,5-dihydrooxazole) ((*S*)-$CyCH_2Box$) (L19)

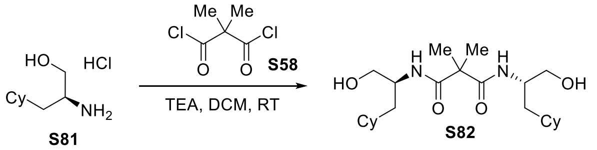

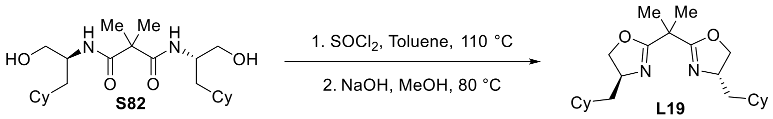

Following a modified version of a reported procedure,[37] a 25 mL, single-necked, round-bottom flask, was charged with (*S*)-3-cyclohexyl-2-aminopropanol hydrochloride (**S81**) (0.461 g, 2.38 mmol, 2.1 equiv.) and DCM (7 mL). The suspension was cooled to 0 °C (ice - water bath), prior to the addition of triethylamine (0.95 mL, 6.8 mmol, 6.0 equiv.). Under stirring, a solution of dimethylmalonyl dichloride (**S58**) (0.15 mL, 1.1 mmol, 1.0 equiv.) in DCM (3.0 mL) was added slowly, at the same temperature. After the addition, the mixture was stirred at room temperature overnight, turning into a homogeneous, pink-orange suspension. After 18 hours, the mixture was diluted with DCM (10 mL) and washed with aq. HCl (1.0 M; 10 mL). The aqueous layer was extracted with DCM (3 x 15 mL). The combined organic layers were washed with brine, dried over Na_2SO_4, filtered, and concentrated under vacuum. The resulting pinkish crude solid was recrystallized from hexane/EtOAc (3mL / 1.2 mL) to afford (4*S*,4'*S*)-2,2'-(propane-2,2-diyl)bis(4-(cyclohexylmethyl)-4,5-dihydrooxazole) (**S82**) (0.220 g, 0.536 mmol, 47% yield) as an off-white, crystalline solid.

^{1}H NMR (400 MHz, Chloroform-*d*) δ 6.23 (d, *J* = 8.2 Hz, 2H, (CO)N*H*), 4.10 (qd, *J* = 8.8, 8.0, 5.4 Hz, 2H, C*H*NH), 3.72 (dd, *J* = 11.4, 3.3 Hz, 2H, C*H*$_2$OH), 3.37 (dd, *J* = 11.4, 6.7 Hz, 2H, C*H*$_2$OH), 2.10 (br s, 2H, O*H*), 1.80 – 1.58 (m, 10H, Cy*H* and/or C*H*$_2$Cy), 1.46 (s, 6H, *Me*), 1.44 – 1.05 (m, 12 H, Cy*H* and/or C*H*$_2$Cy), 1.02 – 0.77 (m, 4H, Cy*H* and/or C*H*$_2$Cy).

Following a reported procedure,[37] in a 10 mL, round-bottom vial, N^1,N^3-bis((*S*)-1-cyclohexyl-3-hydroxypropan-2-yl)-2,2-dimethylmalonamide (**S82**) (0.220 g, 0.535 mmol, 1.0 equiv.) was dissolved in DCM (3.8 mL). The vial was sealed with a PTFE septum. Thionyl chloride (0.40 mL, 5.5 mmol, 10 equiv.) was added by syringe. The resulting colorless solution was then stirred at 45 °C for 4 hours. It was then allowed to cool down to room temperature, diluted with DCM (15 mL), and poured onto ice. Once the ice was molten, the aqueous layer was separated, and extracted with DCM (2 x 15 mL). The combined organic layers were washed with brine, with aq. K_2CO_3 (0.1 M; 20 mL), dried over $MgSO_4$, filtered, and concentrated under vacuum. The resulting pale yellow, crude oil was submitted to column chromatography (SiO_2; DCM/EtOAc 10/1 to 8/2), affording a colorless oil. The latter was dissolved in MeOH (5.0 mL), and the resulting solution was mixed with NaOH (0.19 g, 4.7 mmol, 8.9 equiv.) in water (5.0 mL). The initially formed milky suspension was heated to 100 °C under stirring, which made it convert into a turbid, off-white solution. Stirring was continued at the same temperature for 2 hours. The mixture was then allowed to cool to room temperature, diluted with brine, and extracted with DCM (4 x 20 mL). The combined organic layers were washed with brine, dried over $MgSO_4$, filtered, and concentrated under vacuum. The obtained crude product was purified via flash-chromatography (SiO_2; Pentane/EtOAc 8/2 to 7/3) to furnish (4*S*,4'*S*)-2,2'-(propane-2,2-diyl)bis(4-(cyclohexylmethyl)-4,5-dihydrooxazole) (**L19**) (0.066 g, 0.17 mmol, 31% yield) as a colorless, viscous oil.

^{1}H NMR (400 MHz, Chloroform-*d*) δ 4.31 (dd, *J* = 9.4, 7.9 Hz, 2H, C*H*N), 4.22 – 4.11 (m, 2H, C*H*$_2$O), 3.85 (t, *J* = 7.7 Hz, 2H, C*H*$_2$O), 1.77 – 1.64 (m, 6H, Cy*H* and/or C*H*$_2$Cy), 1.64 – 1.57 (m, 6H, Cy*H* and/or C*H*$_2$Cy), 1.49 (s, 6H, *Me*), 1.42 – 1.31 (m, 2H, Cy*H* and/or C*H*$_2$Cy), 1.31 – 1.08 (m, 8H, Cy*H* and/or C*H*$_2$Cy), 1.01 – 0.85 (m, 4H, Cy*H* and/or C*H*$_2$Cy).
^{1}H-NMR data corresponded to the reported values.[37]

(4*S*,4'*S*)-2,2'-(1,3-Diphenylpropane-2,2-diyl)bis(4-benzyl-4,5-dihydrooxazole) (L20)

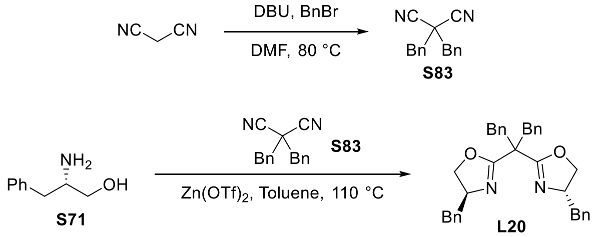

Following a reported procedure,[38] malononitrile (0.33 g, 5.0 mmol, 1.0 equiv.) was dissolved in DMF (10 mL). DBU (1.6 mL, 11 mmol, 2.2 equiv.) and benzyl bromide (1.3 mL, 11 mmol, 2.2 equiv.) were added by syringe at room temperature. The resulting mixture was then stirred under heating for 3 days. The mixture was then allowed to cool down to room temperature, and poured into water (20 mL). The aqueous layer was extracted with Et_2O (4 x 20 mL). The combined organic layers were washed with water (2 x 20 mL), brine, dried over $MgSO_4$, and concentrated under reduced pressure. The resulting yellowish, crude solid was submitted to recrystallization from hexane/EtOAc (10 mL / 4 mL) to provide dibenzylmalonitrile (**S83**) (0.825 g, 3.35 mmol, 67% yield) as a faded yellow, crystalline solid.

^{1}H NMR (400 MHz, Chloroform-*d*) δ 7.41 (s, 10H, Ph*H*), 3.25 (s, 4H, C*H*$_2$Ph).
^{13}C NMR (101 MHz, Chloroform-*d*) δ 132.0, 130.3, 129.0, 128.9, 114.9, 43.5, 41.2.

Following a slightly modified version of a reported procedure,[34] inside a glove-box, a 25 mL, round-bottom vial was charged with $Zn(OTf)_2$ (0.54 g, 1.5 mmol, 2.0 equiv.) and dibenzyl malonitrile (**S83**) (0.185 g, 0.750 mmol, 1.0 equiv.). The vial was closed with a septum, and taken out of the glove-box. Toluene (10 mL) was then added by syringe, and the resulting mixture was stirred at room temperature for 5 minutes. Finally, (*S*)-2-amino-3-phenylpropan-1-ol (**S71**) (0.283 g, 1.87 mmol, 2.5 equiv.) was also added in a single portion. The vial was sealed and heated at 110 °C for 3 days and 15 hours. During this time, a sticky precipitate was formed, which then converted into a whitish, finely dispersed solid. The mixture was allowed to cool down to room temperature, and diluted with EtOAc (20 mL). The organic solution was washed with sat. aq. $NaHCO_3$ (25 mL). Upon separation, the aqueous layer was back-extracted with DCM (3 x 25 mL). The combined organic layers were washed with brine, dried over Na_2SO_4, filtered, and concentrated under vacuum. The resulting pale yellow, crude oil was submitted to column chromatography (Biotage, 12 g SiO_2; EtOAc in pentane, 20 to 75%, with a plateau at 50%). (4*S*,4'*S*)-2,2'-(1,3-Diphenylpropane-2,2-diyl)bis(4-benzyl-4,5-dihydrooxazole) (**L20**) (0.23 g, 0.44 mmol, 60% yield) was collected as a sticky, highly viscous, colorless oil.

^{1}H NMR (400 MHz, Chloroform-*d*) δ 7.36 – 7.26 (m, 14H, Ph*H*), 7.26 – 7.18 (m, 2H, Ph*H*), 7.13 (dd, *J* = 6.8, 1.8 Hz, 4H, Ph*H*), 4.32 (tdd, *J* = 9.4, 7.4, 5.3 Hz, 2H, C*H*N), 4.13 (t, *J* = 8.9 Hz, 2H, C*H*$_2$O), 3.89 (dd, *J* = 8.5, 7.4 Hz, 2H, C*H*$_2$O), 3.37 (d, *J* = 1.5 Hz, 4H, C*H*$_2$Ph), 2.99 (dd, *J* = 13.6, 5.1 Hz, 2H, C*H*$_2$Ph), 2.32 (dd, *J* = 13.7, 9.3 Hz, 2H, C*H*$_2$Ph).
^{1}H-NMR data corresponded to the reported values.[39]

(4*S*,4'*S*)-2,2'-(cyclopropane-1,1-diyl)bis(4-benzyl-4,5-dihydrooxazole) (L23)

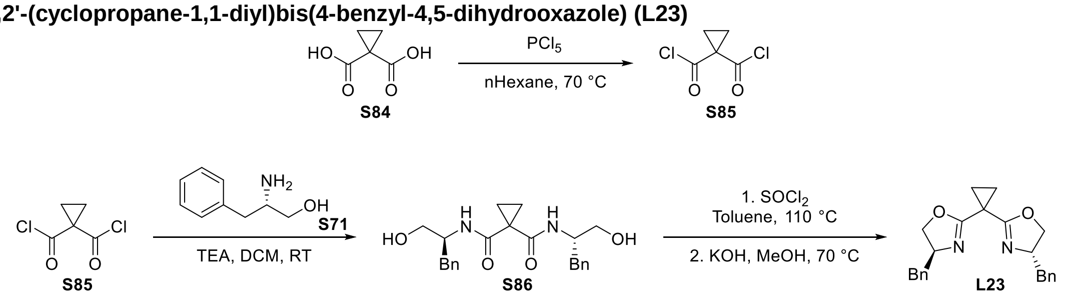

Following a reported procedure, a 25 mL, round-bottom vial was charged with cyclopropane-1,1-dicarboxylic acid (**S84**) (0.350 g, 2.69 mmol, 1.0 equiv.), PCl_5 (2.24 g, 10.8 mmol, 4.0 equiv.) and n-hexane (dry, over molecular sieves; 10.5 mL). The suspension was heated to 70 °C, converting from a suspension into a colourless solution, which was stirred at the same temperature for 17 hours. It was then allowed to cool down to room temperature, and concentrated under vacuum to remove the solvent and $POCl_3$ by-product. Anhydrous pentane (20 mL) was added to the residue and the resulting mixture, the precipate was filtered-off through a plug of celite (then washed with several portions of pentane), and the filtrate concentrated under vacuum to yield cyclopropane-1,1-dicarbonyl dichloride (**S85**) (0.445 g, 2.67 mmol, 99% yield) as a yellowish oil, which was used directly in the following step, without any purification.

Following a slightly modified version of a reported procedure,[23] in a 25 mL, two-necked, round-bottom flask, (*S*)-2-amino-3-phenylpropan-1-ol (**S71**) (0.815 g, 5.39 mmol, 2.0 equiv.) and triethylamine (0.94 mL, 6.7 mmol, 2.5 equiv.) were diluted in DCM (4.0 mL). The mixture was cooled to 0 °C (ice - water bath). A solution of cyclopropane-1,1-dicarbonyl dichloride (0.45 g, 2.7 mmol, 1.0 equiv.) in DCM (1.3 mL) was then added drop-wise. The cooling bath was removed, and the pale-yellow mixture was stirred overnight at room temperature. After 24 hours, the solution was diluted with additional DCM (20 mL), and washed with sat. aq. NH_4Cl (20 mL). The aqueous layer was extracted with DCM (2 x 20 mL). The combined organic layers were washed with sat. aq.

$NaHCO_3$ (20 mL), brine, dried over $MgSO_4$, filtered, and concentrated under vacuum. The resulting pale yellow crude oil was submitted to column chromatography (SiO_2; Pentane/Acetone 9/1 to 8/2) to afford N,N'-bis((*S*)-1-hydroxy-3-phenylpropan-2-yl)cyclopropane-1,1-dicarboxamide (**S86**) (0.456 g, 1.15 mmol, 43% yield) as a orange oil.

^{1}H NMR (400 MHz, Chloroform-*d*) δ 7.30 (t, *J* = 7.3 Hz, 4H, Ph*H*), 7.23 (d, *J* = 7.3 Hz, 2H, Ph*H*), 7.21 – 7.15 (m, 4H, Ph*H*), 7.04 (d, *J* = 8.0 Hz, 2H, (CO)N*H*), 4.17 (td, *J* = 11.1, 10.2, 5.8 Hz, 3H, C*H*NH), 3.70 (dd, *J* = 11.1, 3.3 Hz, 2H, C*H*$_2$OH), 3.53 (dd, *J* = 11.0, 5.7 Hz, 2H, C*H*$_2$OH), 2.88 – 2.76 (m, 4H, C*H*$_2$Ph), 2.73 (s, 2H, O*H*), 1.27 (q, *J* = 4.4, 3.8 Hz, 2H, C*H*$_{2\text{cyclopropane}}$), 1.14 (q, *J* = 5.2, 4.5 Hz, 2H, C*H*$_{2\text{cyclopropane}}$).

Following a reported procedure,[40] in a sealed 25 mL round-bottom vial, N,N'-bis((*S*)-1-hydroxy-3-phenylpropan-2-yl)cyclopropane-1,1-dicarboxamide (**S86**) (0.456 g, 1.15 mmol, 1.0 equiv.) was suspended in toluene (11.5 mL). At room temperature, thionyl chloride (0.21 mL, 2.9 mmol, 2.5 equiv.) was added drop-wise by syringe. The brown mixture was then heated to reflux (110 °C) with conversion to a clear, pale yellow solution. After 3 hours, the mixture looked like a brown-orange solution. The latter was allowed to cool down to room temperature and then poured onto a 1 : 1 mixture of sat. aqueous NH_4Cl and brine (20 mL, overall). The aqueous layer was extracted with DCM (3 x 15 mL). The combined organic layers were washed with sat. aq. $NaHCO_3$, brine, dried over Na_2SO_4, filtered, and concentrated under vacuum. In a 25 mL, round-bottomed vial, the resulting orange, crude oil was diluted in MeOH (11.5 mL) and KOH (pellets; 0.65 g, 12 mmol, 10 equiv.) was added. The resulting mixture was stirred at 80 °C for 4 hours, becoming a yellow-orange solution. The latter was afterwards allowed to cool down to room temperature, and diluted with water (25 mL). The aqueous layer was extracted with Et_2O (2 x 25 mL), dried over Na_2SO_4, filtered, and concentrated under vacuum. The brown residue was submitted to column chromatography (SiO_2; pentane/acetone 19/1 to 18/2) to provide (4*S*,4'*S*)-2,2'-(cyclopropane-1,1-diyl)bis(4-benzyl-4,5-dihydrooxazole) (**L23**) (74 mg, 0.21 mmol, 18% yield) as an orange oil.

^{1}H NMR (400 MHz, Chloroform-*d*) δ 7.29 (dd, *J* = 8.0, 6.4 Hz, 4H, Ph*H*), 7.25 – 7.15 (m, 6H, Ph*H*), 4.40 (tdd, *J* = 9.1, 6.9, 4.9 Hz, 2H, C*H*N), 4.20 (t, *J* = 8.9 Hz, 2H, C*H*$_2$O), 4.02 (dd, *J* = 8.5, 6.9 Hz, 2H, C*H*$_2$O), 3.10 (dd, *J* = 13.7, 4.8 Hz, 2H, C*H*$_2$Ph), 2.64 (dd, *J* = 13.8, 8.6 Hz, 2H, C*H*$_2$Ph), 1.43 – 1.36 (m, 2H, C*H*$_{2\text{cyclopropane}}$), 1.36 – 1.30 (m, 2H, C*H*$_{2\text{cyclopropane}}$).
^{1}H-NMR data corresponded to the reported values.[41]

(4*S*,4'*S*,4''*S*)-2,2',2''-(Propane-1,2,2-triyl)tris(4-*iso*propyl-4,5-dihydrooxazole) ((*S*)-^{i}PrTox) (L24)

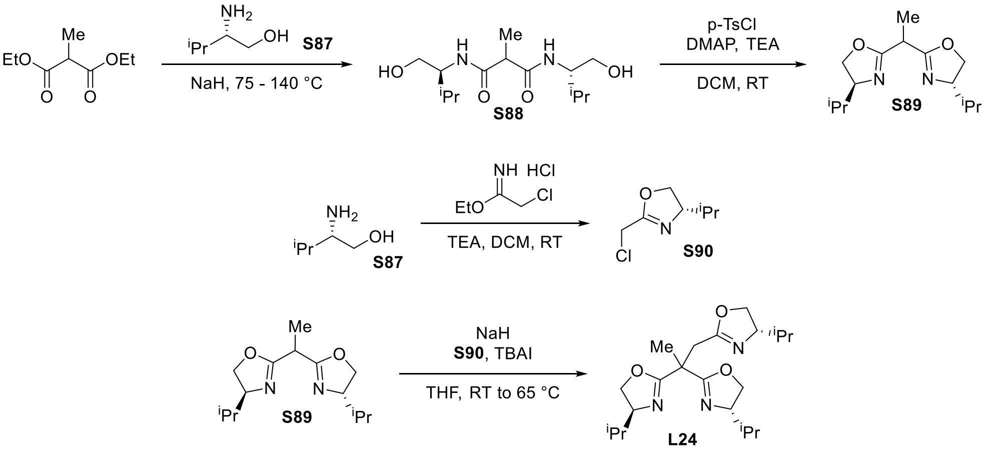

Following a modified version of a reported procedure,[42] a 25 mL, single-necked, round-bottomed flask, equipped with a Liebig condenser, was charged with (*S*)-2-amino-3-methylbutan-1-ol (**S87**) (2.8 mL, 25 mmol, 1.98 equiv.) and diethyl methylmalonate (2.2 mL, 13 mmol, 1.0 equiv.). The mixture was stirred at 130 °C for 9 hours. After this time, it looked like a viscous, yellow oil. The latter was allowed to cool down to room temperature. Residual ethanol was then removed under reduced pressure, resulting in the precipitation of a yellow and sticky crude solid. The latter was submitted to recrystallization from EtOAc (ca. 10 mL). Upon drying it under vacuum for 1 hour, N^1,N^3-Bis((*S*)-1-hydroxy-3-methylbutan-2-yl)-2-methylmalonamide (**S88**) (1.59 g, 5.51 mmol, 43% yield) was collected as a white, poudry solid.

Following a protocol inspired by a reported procedure,[29] a 50 mL, round-bottom, single-necked flask was charged with N^1,N^3-bis((*S*)-1-hydroxy-3-methylbutan-2-yl)-2-methylmalonamide (**S88**) (0.75 g, 2.6 mmol, 1.0 equiv.), DMAP (32 mg, 0.26 mmol, 10 mol%) and DCM (11 mL). Triethylamine (1.8 mL, 13 mmol, 5.0 equiv.) was added, and the resulting mixture was cooled to 0 °C (ice - water bath). Under stirring, a solution of *p*-TsCl (1.09 g, 5.72 mmol, 2.2 equiv.) in DCM (2.5 mL) was added drop-wise. The cooling bath was removed and the mixture was stirred at room temperature for 46 hours. The off-white suspension became initially a turbid, yellow solution before some precipitation started again. After the indicated time, the reaction was quenched by addition of water (25 mL) followed by dilution with DCM (25 mL). The aqueous layer was then extracted with DCM (3 x 30 mL). The combined organic layers were washed with brine, dried over Na_2SO_4, filtered, and concentrated under vacuum. The resulting

orange crude oil was submitted to column chromatography (SiO_2; pentane/acetone 10/1 to 8/2) to provide (4*S*,4'*S*)-2,2'-(ethane-1,1-diyl)bis(4-isopropyl-4,5-dihydrooxazole) (**S89**) (0.320 g, 1.27 mmol, 49% yield) as a pale yellow, viscous oil.

^{1}H NMR (400 MHz, Chloroform-*d*) δ 4.23 (td, *J* = 8.0, 1.0 Hz, 2H, CH_2O), 3.99 (t, *J* = 7.8 Hz, 2H, CH_2O), 3.94 (m, 2H, C*H*N), 3.53 (m, 1H, C*H*Me), 1.78 (m, 2H, $CH_{isopropyl}$), 1.48 (d, *J* = 7.3 Hz, 3H, CH*Me*), 0.94 (d, *J* = 2.3 Hz, 3H, $Me_{isopropyl}$), 0.92 (d, *J* = 2.3 Hz, 3H, $Me_{isopropyl}$), 0.89 – 0.83 (m, 6H, $Me_{isopropyl}$).
^{1}H-NMR data corresponded to the reported values (with a systematic shift of +.0.03 ppm).[43]

Following a reported procedure,[26] in a 25 mL, two-necked, round-bottom flask, (*S*)-2-amino-3-methylbutan-1-ol (**S87**) (0.54 mL, 4.8 mmol, 1.02 equiv.) and ethyl 2-chloroethanimidoate hydrochloride (0.75 g, 4.7 mmol, 1.0 equiv.) were suspended in DCM (dry; 7.0 mL) at room temperature. Triethylamine (0.86 mL, 6.2 mmol, 1.3 equiv.) was added: the suspension became less turbid at the beginning, and then precipitation started occurring with the color turning from yellowish to pink, and then to reddish. The mixture was stirred at room temperature for 5 hours. The mixture was stirred at room temperature overnight. The volatiles were then removed under reduced pressure to give a dark paste, which was partitioned between sat. aq. NH_4Cl (20 mL) and EtOAc (20 mL). Upon separation, the aqueous layer was further extract with EtOAc (3 x 15 mL). The combined organic layers were washed with brine, dried over $MgSO_4$, filtered, and concentrated under vacuum. The resulting reddish, crude oil was submitted to column chromatography (dry load on silica gel; SiO_2; EtOAc in pentane, 5 to 40% - containing 1% v/v NEt_3) to give (*S*)-2-(chloromethyl)-4-isopropyl-4,5-dihydrooxazole (**S90**) (0.365 g, 2.26 mmol, 48% yield) as a colorless oil.

^{1}H NMR (400 MHz, Chloroform-*d*) δ 4.35 (dd, *J* = 9.7, 8.4 Hz, 1H, CH_2O), 4.11 (s, 2H, CH_2Cl), 4.06 (t, *J* = 8.3 Hz, 1H, CH_2O), 4.00 – 3.93 (m, 1H, C*H*N), 1.78 (dq, *J* = 13.4, 6.7 Hz, 1H, $CH_{isopropyl}$), 0.97 (d, *J* = 6.7 Hz, 3H, $Me_{isopropyl}$), 0.89 (d, *J* = 6.7 Hz, 3H, $Me_{isopropyl}$).
^{1}H-NMR data corresponded to the reported values.[26]

Following a slightly modified version of a reported procedure,[27] a 25 mL, round-bottom vial was charged with (4*S*,4'*S*)-2,2'-(ethane-1,1-diyl)bis(4-isopropyl-4,5-dihydrooxazole) (**S89**) (0.127 g, 0.503 mmol, 1.0 equiv.), followed by THF (dry; 4.0 mL). To the resulting solution was added NaH (60% dispersion in mineral oil; 54 mg, 1.4 mmol, 2.8 equiv.). The turbid, off-white mixture was stirred at room temperature for 30 minutes. It was then cooled to 0 °C (ice - water bath). and a solution of (*S*)-2-(chloromethyl)-4-isopropyl-4,5-dihydrooxazole (**S90**) (0.130 g, 0.805 mmol, 1.6 equiv.) in THF (dry; 1.0 mL) was added slowly. The mixture was then heated to 50 °C. After 18 at this temperature, the mixture was allowed to cool down to room temperature, and poured into water/sat. aq. NH_4Cl (40 / 10 mL). The aqueous layer was extracted with DCM (4 x 15 mL). The combined organic layers were washed with brine, dried over Na_2SO_4, filtered, and concentrated under vacuum. The resulting pale yellow, crude oil was submitted to column chromatography (SiO_2; packed with pentane/acetone 9/1; eluted with pentane/acetone 7/1 to 9/2) to provide (4*S*,4'*S*,4''*S*)-2,2',2''-(propane-1,2,2-triyl)tris(4-isopropyl-4,5-dihydrooxazole) (**L24**, (*S*)-^{i}PrTox) (0.108 g, 0.286 mmol, 57% yield) as a pale yellow oil.

^{1}H NMR (400 MHz, Chloroform-*d*) δ 4.27 – 4.13 (m, 3H, C*H* and/or CH_2), 4.05 – 3.83 (m, 6H, C*H* and/or CH_2), 3.09 (d, *J* = 14.2 Hz, 1H, CH_2Ox), 2.95 (d, *J* = 14.5 Hz, 1H, CH_2Ox), 1.85 – 1.71 (m, 3H, $CH_{isopropyl}$), 1.66 (s, 3H, $C_{quat}Me$), 0.94 (d, *J* = 6.8 Hz, 3H, $Me_{isopropyl}$), 0.94 (d, *J* = 6.8 Hz, 3H, $Me_{isopropyl}$), 0.90 (d, *J* = 6.8 Hz, 3H, $Me_{isopropyl}$), 0.86 (d, *J* = 6.7 Hz, 6H, $Me_{isopropyl}$), 0.84 (d, *J* = 6.8 Hz, 3H, $Me_{isopropyl}$).
^{1}H-NMR data corresponded to the reported values.[44]

(4*S*,4'*S*)-2,2'-(1-Phenylpropane-2,2-diyl)bis(4-isopropyl-4,5-dihydrooxazole) (L25)

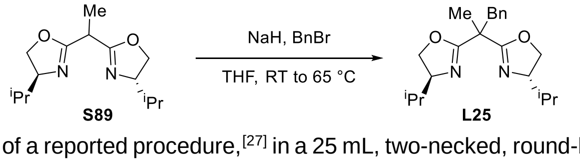

Following a slightly modified version of a reported procedure,[27] in a 25 mL, two-necked, round-bottom flask, (4*S*,4'*S*)-2,2'-(ethane-1,1-diyl)bis(4-isopropyl-4,5-dihydrooxazole) (**S89**) (0.125 g, 0.495 mmol, 1.0 equiv.) was dissolved in THF (dry; 2.5 mL). NaH (60% dirspersion in mineral oil; 36 mg, 0.89 mmol, 1.8 equiv.) was added in a single portion at room temperature, with immediate release of gas. The resulting suspension was stirred at room temperature for 1 hour, after which a solution of benzyl bromide (0.090 mL, 0.76 mmol, 1.5 equiv.) in THF (dry; 2.5 mL) was added drop-wise. The resulting mixture was stirred at room temperature for 7 hours. It was then poured onto water and aq. NH_4Cl (overall 15 mL). The aqueous layer was extracted with DCM (3 x 15 mL). The combined organic layers were washed with brine, dried over $MgSO_4$, filtered, and concentrated under reduced pressure. The pale yellow crude oil was submitted to column chromatography (SiO_2; pentane/acetone 10/1 to 8/2) to furnish (4*S*,4'*S*)-2,2'-(1-phenylpropane-2,2-diyl)bis(4-isopropyl-4,5-dihydrooxazole) (**L25**) (0.084 g, 0.25 mmol, 50% yield) as a pale yellow oil.

^{1}H NMR (400 MHz, Chloroform-*d*) δ 7.26 – 7.18 (m, 3H, Ph*H*), 7.18 – 7.13 (m, 2H, Ph*H*), 4.23 (ddd, *J* = 9.2, 8.0, 2.8 Hz, 2H, C*H*N or CH_2O), 4.00 (m, 2H, C*H*N or CH_2O), 3.93 (ddd, *J* = 9.9, 7.3, 5.7 Hz, 2H, C*H*N or CH_2O), 3.33 (d, *J* = 13.5 Hz, 1H, CH_2Ph), 3.25

(d, J = 13.5 Hz, 1H, CH_2Ph), 1.81 (m, 1H, C$H_{iso\text{propyl}}$), 1.72 (dq, J = 13.5, 6.7 Hz, 1H, C$H_{iso\text{propyl}}$), 1.42 (s, 3H, *Me*), 0.92 (d, J = 6.8 Hz, 3H, $Me_{iso\text{propyl}}$), 0.87 (t, J = 6.6 Hz, 6H, $Me_{iso\text{propyl}}$), 0.81 (d, J = 6.8 Hz, 3H, $Me_{iso\text{propyl}}$).
^{1}H-NMR data corresponded to the reported values.[45]

5.1.3 Preparation of other ligands that were tested during the optimization

Synthesis of non-commercially available (*R*)-N,N'-([1,1'-binaphthalene]-2,2'-diyl)bis(4-bromobenzamide) (L26)

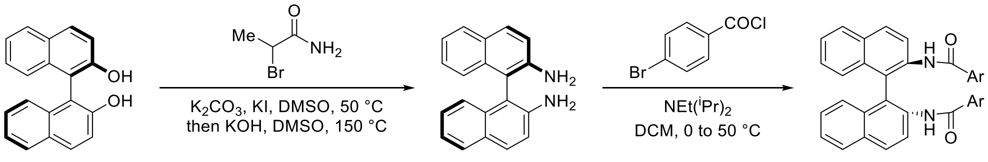

S91 **S92** **L26**
Ar = *p*-Br-Ph

Following a reported procedure,[46] a 100 mL, single-necked, round-bottom flask was charged with (*R*)-BINOL (**S91**) (0.785 g, 2.74 mmol, 1.0 equiv.), 2-bromopropanamide (1.25 g, 8.22 mmol, 3.0 equiv.), K_2CO_3 (1.14 g, 8.22 mmol, 3.0 equiv.) and KI (0.068 g, 0.41 mmol, 10 mol%). DMSO (27 mL) was then added. The resulting yellow suspension was stirred at 50 °C for 20 hours, darkening to green-yellow. After this time, TLC analysis (DCM/MeOH 98/2) showed the complete disappearance of (R)-BINOL. At this point, the mixture was allowed to cool down to room temperature, ground KOH (1.92 g, 34.3 mmol, 12.5 equiv.) was added, and stirring was continued at 150 °C for 4 hours. This led the mixture to darken to black-brown. The mixture was then cooled to room temperature and water (50 mL) was added to quench the reaction. At this point, the mixture became pale brown, looking like a fine suspension. The aqueous solution was extracted with EtOAc (3 x 40 mL). The combined organic layers were washed with brine, dried over anhydrous Na_2SO_4, filtered, and concentrated *in vacuo*. The residue was submitted to column chromatography (Biotage flash chromatographer, 25 g SiO_2; MeOH in DCM, 0 to 7%) to afford (*R*)-1,1'-binaphtyl-2,2'-diamine (**S92**) (0.480 g, 1.69 mmol, 62% yield) as an off-white solid.

^{13}C NMR (101 MHz, Chloroform-*d*) δ 142.8, 133.8, 129.6, 128.6, 128.3, 127.0, 124.1, 122.5, 118.4, 112.7.
^{13}C-NMR data corresponded to the reported values.[47]

Following a reported procedure,[48] a 25 mL, single necked, round-bottomed flask was charged with (*R*)-1,1'-binaphtyl-2,2'-diamine (**S92**) (0.105 g, 0.369 mmol, 1.0 equiv.) and DCM (3.7 mL). ^{i}Pr$_2$NEt (0.18 mL, 1.1 mmol, 3.0 equiv.) was added and the mixture was cooled to 0 °C (ice - water bath). Finally, 4-bromobenzoyl chloride (0.203 g, 0.923 mmol, 2.5 equiv.) was also added. The initially clear solution became turbid and converted into an off-white suspension. After being stirred for 2 hours at room temperature, the reaction was quenched by addition of aq. HCl (1.0 M; 2.0 mL). The aqueous layer was separated and extracted with DCM (3 x 20 mL). The combined organic layers were washed with brine, dried over $MgSO_4$, filtered, and concentrated under vacuum. The resulting residue was submitted to column chromatography (Biotage flash chromatographer, 12 g SiO_2; EtOAc in DCM, 0 to 12%) to provide (*R*)-N,N'-([1,1'-binaphthalene]-2,2'-diyl)bis(4-bromobenzamide) (**L26**) (0.187 g, 0.288 mmol, 78% yield) as a white solid.

^{1}H NMR (400 MHz, Chloroform-*d*) δ 8.62 (d, J = 9.0 Hz, 2H), 8.13 (d, J = 9.0 Hz, 2H), 8.00 (d, J = 8.2 Hz, 2H), 7.69 (s, 2H), 7.54 - 7.47 (m, 2H), 7.36 (dd, J = 8.2, 6.2 Hz, 6H), 7.25 (d, J = 8.4 Hz, 2H), 7.06 (d, J = 8.5 Hz, 2H).
^{1}H-NMR data corresponded to the reported values.[48]

5.2 Optimization of the Enantioselective reaction

General procedures:

Conditions for d.r. and e.r measurement based on HPLC analysis: Column IA, elution with n-hexane / ^{i}PrOH 6/4, flow 1.0 mL/min. The retention times for the two enantiomers of the major (*trans*) diastereoisomer were: 8.9 and 22.0 min. The retention times for the two enantiomers of the minor (*cis*) diastereoisomer were: 13.3 and 17.2 min.

Note on the sign (+/-) of the enantiomeric excess (e.e.): In the present document, e.e. values were determined consistently with the retention times. i.e. positive e.e. values were obtained when the larger integral area corresponded to the peak at the lower retention time and vice-versa.

GP6.[a.MgI$_2$]: enantioselective (4+3) annulation of varying dialkyl diester DA cyclopropanes under Mg(II) catalysis, with Box ligands.
Inside a glove box, a 10 mL, round bottomed vial was charged with MgI_2 (5.6 mg, 0.020 mmol, 20 mol%), the ligand (0.022 mmol, 22 mol%) and activated molecular sieves 3Å (60-70 mg). The vial was sealed with a PTFE cap and taken out of the glovebox. DCM (0.6 mL) was added, and the resulting suspension was stirred at room temperature for 1 hour (becoming bright yellow). A solution of dibenzyl 2-(4-methoxyphenyl)cyclopropane-1,1-dicarboxylate **2a** (0.042 g, 0.10 mmol, 1.0 equiv.) and N-Benzylidene-1-((*tert*-butyldimethylsilyl)oxy)ethenamine (**4a**) (0.039 g, 0.15 mmol, 1.5 equiv.) in DCM (0.4 mL) was then added by syringe. The

resulting yellow-green suspension was stirred at room temperature overnight (18 hours). The reaction mixture was then diluted with DCM (2 mL), and the solids were filtered off through a short plug of celite, which was washed with several portions of DCM/MeOH 9/1. The filtrate was concentrated under reduced pressure. The resulting green, viscous crude oil was submitted to column chromatography (Biotage flash chromatographer, 4 g SiO_2; EtOAc in pentane, 15 to 60%). The product was collected as a white foam, and submitted to HPLC analysis.

GP6.[a.Cu(II)]: enantioselective (4+3) annulation of varying dialkyl diester DA cyclopropanes under Cu(II) catalysis, with Box ligands.
Inside a glove box, a 10 mL, round bottom vial was charged with the Cu(II) catalyst (0.020 mmol, 20 mol%), the ligand (0.022 mmol, 22 mol%) and activated molecular sieves 3Å (60-70 mg). The vial was sealed with a PTFE cap and taken out of the glovebox. DCM (0.6 mL) was added, and the resulting suspension was stirred at room temperature for 3 hours (becoming green to turquoise, depending on the used ligand). A solution of dibenzyl 2-(4-methoxyphenyl)cyclopropane-1,1-dicarboxylate **2a** (0.042 g, 0.10 mmol, 1.0 equiv.) and N-Benzylidene-1-((*tert*-butyldimethylsilyl)oxy)ethenamine (**4a**) (0.039 g, 0.15 mmol, 1.5 equiv.) in DCM (0.4 mL) was then added by syringe. The resulting yellow-green suspension was stirred at room temperature overnight (18 hours). The reaction mixture was then diluted with DCM (2 mL), and the solids were filtered off through a short plug of celite, which was washed with several portions of DCM/MeOH 9/1. The filtrate was concentrated under reduced pressure. The resulting green, viscous crude oil was submitted to column chromatography (Biotage flash chromatographer, 4 g SiO_2; EtOAc in pentane, 15 to 60%). The product was collected as a white foam, and submitted to HPLC analysis.

GP6.[b,c] enantioselective (4+3) annulation of varying dialkyl diester DA cyclopropanes with Feng L3-PrPr$_2$ ligand.
Inside a glove box, a 10 mL, round bottomed vial was charged with the Lewis acid catalyst (0.020 mmol, 20 mol%) and Feng L3-PrPr$_2$ ligand (**L27**) (0.012 g, 0.20 mmol, 20 mol%) ([c] when $Tb(OTf)_3$ was used, molecular sieves 3Å (70 mg) and NaBarF (35 mg, 0.40 mmol, 40 mol%) were also added).[49] The vial was sealed with a PTFE cap and taken out of the glovebox. DCM (0.6 mL) was added, and the resulting mixture was stirred at room temperature for 2 hours (remaining colorless). A solution of dibenzyl 2-(4-methoxyphenyl)cyclopropane-1,1-dicarboxylate **2a** (0.042 g, 0.10 mmol, 1.0 equiv.) and N-Benzylidene-1-((*tert*-butyldimethylsilyl)oxy)ethenamine (**4a**) (0.039 g, 0.15 mmol, 1.5 equiv.) in DCM (0.4 mL) was then added by syringe. The reaction mixture was stirred at room temperature overnight (18 hours). It was then diluted with DCM (2 mL), and the solids were filtered off through a short plug of celite, which was washed with several portions of DCM/MeOH 9/1. The filtrate was concentrated under reduced pressure. The resulting green, viscous crude oil was submitted to column chromatography (Biotage flash chromatographer, 4 g SiO_2; EtOAc in pentane, 15 to 60%). The product was collected as a white foam, and submitted to HPLC analysis.

GP6.[d] enantioselective (4+3) annulation of varying dialkyl diester DA cyclopropanes under Yb(III) catalysis with BINAD ligand L26.
Inside a glove box, a 10 mL, round bottomed vial was charged with $Yb(OTf)_3$ 12.4 mg, 0.0200 mmol, 20 mol%), 4-bromo-N-[1-[2-[(4-bromobenzoyl)amino]naphthalen-1-yl]naphthalen-2-yl]benzamide (**L26**) (15.6 mg, 0.0240 mmol, 22 mol%), and activated molecular sieves 3Å (75 mg). The vial was sealed with a PTFE cap and taken out of the glovebox. A solution of DBU (7.0 µL, 0.047 mmol, 47 mol%) in DCM (dry; 0.4 mL) was added by syringe. The resulting colorless mixture was stirred at room temperature for 3 hours. A solution of A solution of dibenzyl 2-(4-methoxyphenyl)cyclopropane-1,1-dicarboxylate (**2a**) (0.042 g, 0.10 mmol, 1.0 equiv.) and (*E*)-N-[1-[*tert*-butyl(dimethyl)silyl]oxyethenyl]-1-phenylmethanimine (**4a**) (0.039 g, 0.15 mmol, 1.5 equiv.) in DCM (1.0 mL) was then added by syringe. The resulting mixture was then stirred at room temperature for 18 hours, turning pale yellow-green during this time. It was then diluted with DCM (2 mL), and the solids were filtered off through a short plug of celite, which was washed with several portions of DCM/MeOH 9/1. The filtrate was concentrated under reduced pressure. The resulting green, viscous crude oil was submitted to column chromatography (Biotage flash chromatographer, 4 g SiO_2; EtOAc in pentane, 15 to 60%). The product was collected as a white foam, and submitted to HPLC analysis.

Table S3. Optimization of the asymmetric version of the (4+3) annulation: *Ligand/L.A.*

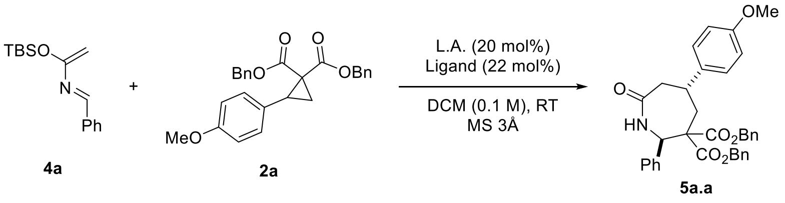

Entry[a]	Lewis Acid	Ligand	Yield	d.r.	e.r. (e.e.)
1[b]	$Yb(OTf)_3$	Feng L3-PrPr$_2$ (**L27**)	-	-	-
2[b]	$Dy(OTf)_3$	Feng L3-PrPr$_2$ (**L27**)	Traces	-	-

3[c]	$Tb(OTf)_3$	Feng L3-PrPr$_2$ (**L27**)	25%		Racemic
4[d]	$Yb(OTf)_3$	Ar-BINAD (**L26**)	36%	96 : 4	53 : 47 (6% ee)
5	MgI_2	(*S*)-CyPyBox (**L2**)	67%	98 : 2	69 : 31 (38% ee)
6	$Yb(OTf)_3$	(*S*)-CyPyBox (**L2**)	< 10%	-	-
7	MgI_2	(*S*)-^{i}PrPyBox (**L6**)	64%	97 : 3	55 : 45 (10% ee)
8	MgI_2	(*S*)-^{t}BuPyBox (**L7**)	64%	97 : 3	53 : 47 (5% ee)
9	MgI_2	(*S*)-PhPyBox (**L8**)	55%	89 : 11	61 : 39 (32% ee)
10	MgI_2	(*S*)-BnPyBox (**L9**)	69%	94 : 6	Racemic
11	MgI_2	(3*R*,8*S*)-IndaPyBox (**L10**)	50%	94 : 6	41 : 59 (-20% ee)
12	$Cu(OTf)_2$	(*S*)- ^{t}BuBox (**L15**)	Traces	-	-
13	$Cu(SbF_6)_2$	(*S*)- ^{t}BuBox (**L15**)	-	-	-
14	$Cu(OTf)_2$	(*R*)-BnBox (**L16**)	78%	92 : 8	82 : 18 (64% ee)
15	$Cu(ClO_4)_2 \bullet H_2O$	(*R*)-BnBox (**L16**)	76%	85 : 15	82 : 18 (64% ee)
16	$Cu(SbF_6)_2$	(*R*)-BnBox (**L16**)	68%	60 : 40	87 : 13 (74% ee)
17	$Cu(OTf)_2$	(*S*)-CyBox (**L3**)	85%	89 : 11	12 : 88 (-75% ee)
18	$Cu(OTf)_2$	(*S*)-BnTox (**L14**)	67%	97 : 3	6 : 94 (-88% ee)
19	$Cu(OTf)_2$	(*S*)-CyTox (**L5**)	84%	78 : 22	4 : 96 (-91% ee)
20[e]	$Cu(OTf)_2$	**L4**	62%	85 : 15	8 : 92 (-84% ee)
21	$Cu(OTf)_2$	(*S*)- ^{i}PrBox (**L11**)	77%	88 : 12	23 : 77 (-53% ee)
22	$Cu(SbF_6)_2$	(*S*)- ^{i}PrBox (**L11**)	87%	53 : 47	15 : 85 (-70% ee)
23	$Cu(OTf)_2$	(*R*)-PhBox (**L12**)	24%	93 : 7	80 : 20 (60% ee)
24	$Cu(OTf)_2$	(*S*)-CypBox (**L13**)	90%		20 : 80 (-60% ee)
25	$Cu(OTf)_2$	(*R*)-BzhBox (**L17**)	90%	97 : 3	83 : 17 (66% ee)
26	$Cu(OTf)_2$	(*R*)-NpBox (**L18**)	78%	90 : 10	74 : 26 (48% ee)
27	$Cu(OTf)_2$	**L19**	38%	75 : 25	15 : 85 (-70% ee)
28	$Cu(OTf)_2$	**L20**	Traces	-	-
39	$Cu(OTf)_2$	**L21**	73%	89 : 11	6 : 94 (-88% ee)
30	$Cu(OTf)_2$	**L22**	76%	88 : 12	20 : 80 (-60% ee)
31	$Cu(OTf)_2$	**L23**	85%	90 : 10	19 : 81 (-78% ee)
32	$Cu(OTf)_2$	(*S*)- ^{i}PrTox (**L24**)	75%	75 : 25	8 : 92 (-84% ee)
33	Cu(OTf)2	**L25**	37%	89 : 11	8 : 92 (-84% ee)

[a] Reaction conditions: 0.10 mmol cyclopropane **2a**, 0.15 mmol **4a**; 0.020 mmol Lewis acid catalyst, 0.022 mmol ligand; 60-70 mg MS 3Å, in 1.0 mL DCM, at room temperature. Yield determined by isolation through column chromatography. d.r. and e.r. determined by HPLC analysis; [b] Reaction conditions: 0.10 mmol cyclopropane **2a**, 0.15 mmol **4a**; 0.020 mmol Lewis acid catalyst, 0.022 mmol Feng L3-PrPr$_2$ ligand. Yield determined by isolation through column chromatography. d.r. and e.r. determined by HPLC analysis; [c] same conditions as in [b] but with 70 mg MS 3Å and 0.40 mmol NaBarF; [d] 0.10 mmol cyclopropane **2a**, 0.15 mmol **4a**; 0.020 mmol $Yb(OTf)_3$, 0.022 mmol BINAD ligand **L26**, 0.05 mmol DBU, 75 mg 3Å MS, in 1.6 mL DCM, at room temperature. Yield determined by isolation through column chromatography. d.r. and e.r. determined by HPLC analysis [e] with 0.20 mmol **4a**.

Table S4. Fine adjustment of the asymmetric version of the (4+3) annulation with Cu(II) and ligand **L4** and **L5**

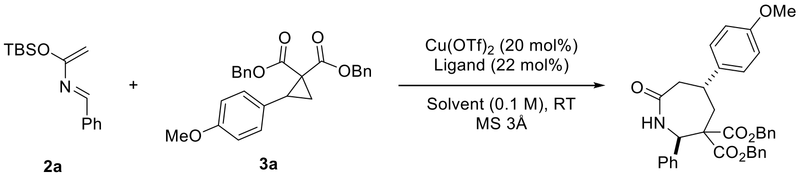

2a **3a** **5a.a**

Entry	Ligand (n mmol%) / Solvent	Equiv. **4a**	Yield	d.r.	e.r. (ee)
1	**L4** (11 mol%) / PhCl	1.5	35-90%	> 95 : 5	up to 2 : 98 (-96% ee)

2	**L4** (22 mol%) / PhCl	1.5	65-82%	> 95 : 5	up to 2 : 98 (-96% ee)
3	**L4** (30 mol%) / PhCl	1.5	51%	97 : 3	4.5 : 95.5 (-91% ee)
4	**L4** (24 mol%) / PhCl	2.0	67%	-	- not measured -
5	**L4** (24 mol%) / DCM	2.0	62%	85 : 15	8 : 92 (-85% ee)
6	**L4** (24 mol%) / $PhCF_3$	2.0	59%	93 : 7	4 : 96 (-92% ee)
7	**L4** (24 mol%) / (Toluene / DCM) (6/4)	2.0	69%	96 : 4	5 : 95 (-91% ee)
8	**L4** (24 mol%) / (Toluene / DCM) (6/4)	2.0	73-81%	96 : 4	5 : 95 (-90% ee)
9	**L5** (22 mol%) / DCM	1.5	84%	78 : 22	5 : 95 (-91% ee)
10	**L5** (22 mol%) / DCM	2.0	75%	79 : 21	6 : 94 (-88% ee)
11*	**L5** (22 mol%) / DCM	1.5	96%	66 : 34	10 : 90 (-80% ee)
12	**L5** (22 mol%) / DCE	1.5	78%	78 : 22	5 : 95 (-90% ee)
13	**L5** (22 mol%) / Et_2O	1.5	44%	95 : 5	3 : 97 (-94% ee)
14	**L5** (22 mol%) / $PhCF_3$	1.5	64%	89 : 11	3 : 97 (-94% ee)
15	**L5** (22 mol%) / Toluene	1.5	55%	98 : 2	2 : 98 (-96% ee)
16	**L5** (22 mol%) / PhCl	1.5	78%	91 : 9	7 : 93 (-86% ee)
17	**L5** (22 mol%) / PhCl	2.0	64%	-	- not measured -
18	**L5** (22 mol%) / (Toluene / DCM) (6/4)	1.5	75%	93 : 7	3 : 97 (-94% ee)
19	**L5** (22 mol%) / (Toluene / DCM) (6/4)	1.0[c] *vs* 1.5 **3a**	48%	93 : 7	7 : 93 (-84% ee)
20	**L5** (22 mol%) / (Toluene / DCM) (6/4)	2.0	71%	95 : 5	7 : 93 (-84% ee)
21	**L5** (22 mol%) / (Toluene / DCM) (4/6)	2.0	73%	91 : 9	7 : 93 (-84% ee)
22	**L5** (11mol%) / (Toluene / DCM) (6/4)	1.5	57%	93 : 7	4 : 96 (-93% ee)

[a] Reaction conditions: 0.10 mmol cyclopropane **2a**, 0.15 mmol **4a** in 0.4 mL solvent added to: 0.020 mmol $Cu(OTf)_2$, n mmol% ligand; 60-70 mg MS 3Å, in 0.6 mL solvent, at room temperature. Yield determined by isolation through column chromatography. d.r. and e.r. determined by HPLC analysis; [b] $Cu(ClO_4)_2 \bullet H_2O$ used instead of $Cu(OTf)_2$; [c] Reversed stoichiometry experiment: same conditions as in [a] but using 0.15 mmol **2a** with 0.10 mmol **4a**.

5.3 Scope of the enantioselective reaction

GP7: ***General procedure for the asymmetric (S)-CyTox/Cu(OTf)₂-catalyzed (4+3) annulation of DA cyclopropanes 3a with aryl substituted azadienes.***
Inside a glove box, a 10 mL, round bottom vial was charged with $Cu(OTf)_2$ (7.2 mg, 0.020 mmol, 20 mol%), (*S*)-CyTox (**L5**) (11 mg, 0.022 mmol, 22 mol%) and activated molecular sieves 3Å (60-70 mg). The vial was sealed with a PTFE cap and taken out of the glovebox. Toluene (0.6 mL) was added, and the resulting suspension was stirred at room temperature for 3 hours (becoming turquoise). A solution of cyclopropane **2** (0.10 mmol, 1.0 equiv.) and azadiene **4** (0.15 mmol, 1.5 equiv.) in DCM (0.4 mL) was then added by syringe. The resulting yellow-green suspension was stirred at room temperature overnight (18 hours). The reaction mixture was then diluted with DCM (2 mL), and the solids were filtered off through a short plug of celite, which was washed with several portions of DCM/MeOH 9/1. The filtrate was concentrated under reduced pressure. The resulting green, viscous crude oil was submitted to column chromatography (Biotage flash chromatographer, 4 g SiO_2; EtOAc in pentane, 15 to 60%). The product was collected as a white foam, and submitted to HPLC analysis.

Enantioenriched dibenzyl (2*R*,5*R*)-5-(4-methoxyphenyl)-7-oxo-2-phenylazepane-3,3-dicarboxylate (5a.a)
Following the **GP7** and starting from dibenzyl 2-(4-methoxyphenyl)cyclopropane-1,1-dicarboxylate (**2a**) (0.042 g, 0.10 mmol, 1.0 equiv.) and N-benzylidene-1-((*tert*-butyldimethylsilyl)oxy)ethenamine (**4a**) (0.039 g, 0.15 mmol, 1.5 equiv.), the title compound (0.042 g, 0.075 mmol; d.r. 93 : 7) was obtained in 75% yield and 3.5 : 96.5 e.r. (-93% ee).
The reaction was also performed on a larger scale using: 2-(4-methoxyphenyl)cyclopropane-1,1-dicarboxylates **2a** (0.25 g, 0.60 mmol, 1.0 equiv.) and N-benzylidene-1-((*tert*-butyldimethylsilyl)oxy)ethenamine (**4a**) (0.24 g, 0.90 mmol, 1.5 equiv.) in DCM (2.2 mL). this solution was added to a suspension of $Cu(OTf)_2$ (43 mg, 0.12 mmol, 20 mol%), (*S*)-CyTox (**L5**) (66 mg, 0.13 mmol, 22 mol%) and MS 3Å (0.45 g) in toluene (3.4 mL). The title compound (0.265 g, 0.470 mmol; d.r. 94 : 6) was obtained in 78% yield and 3 : 97 e.r. (-94% ee).
$[\alpha]_D^{20}$ = -34.3° (1 g/ 100 mL chloroform).

Experiments on 0.1 mmol scale:

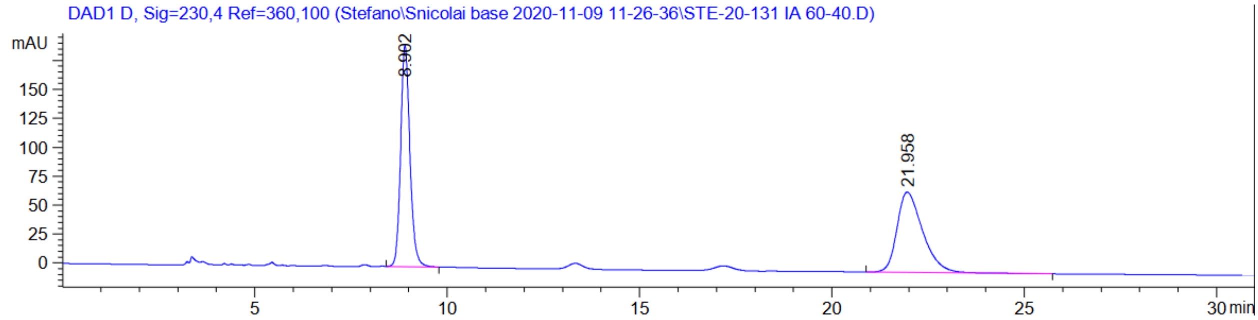

Signal 4: DAD1 D, Sig=230,4 Ref=360,100

Peak #	RetTime [min]	Type	Width [min]	Area [mAU*s]	Height [mAU]	Area %
1	8.902	BB	0.2571	3207.73413	191.78648	49.7441
2	21.958	BBA	0.7062	3240.73853	69.47930	50.2559
Totals :				6448.47266	261.26579	

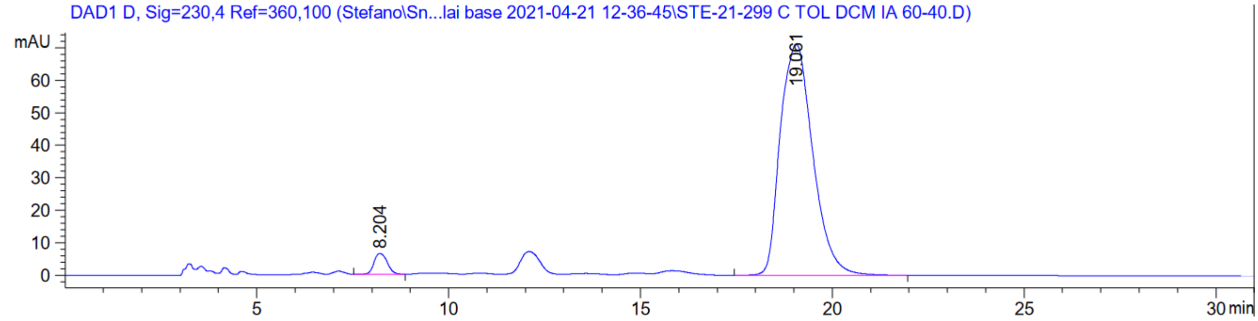

Peak #	RetTime [min]	Type	Width [min]	Area [mAU*s]	Height [mAU]	Area %
1	8.204	BB	0.3881	153.23845	6.38344	3.5213
2	19.061	BB	0.9376	4198.52539	71.24062	96.4787
Totals :				4351.76384	77.62405	

Reiteration on 0.6 mmol scale:

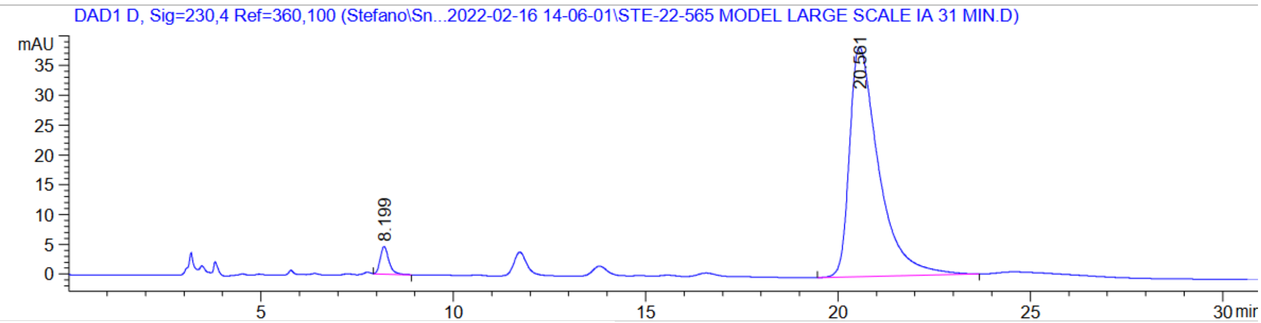

Signal 4: DAD1 D, Sig=230,4 Ref=360,100

Peak #	RetTime [min]	Type	Width [min]	Area [mAU*s]	Height [mAU]	Area %
1	8.199	BB	0.2357	71.74159	4.60630	3.4055
2	20.561	BB	0.7854	2034.88208	38.57898	96.5945
Totals :				2106.62367	43.18528	

Enantioenriched dibenzyl (2*R*,5*R*)-5-(3,4-dimethoxyphenyl)-7-oxo-2-phenylazepane-3,3-dicarboxylate (5a.b)

Following the **GP7** and starting from dibenzyl 2-(3,4-dimethoxyphenyl)cyclopropane-1,1-dicarboxylate (**2b**) (0.045 g, 0.10 mmol, 1.0 equiv.) and N-benzylidene-1-((*tert*-butyldimethylsilyl)oxy)ethenamine (**4a**) (0.039 g, 0.15 mmol, 1.5 equiv.), the title compound (0.038 g, 0.064 mmol; d.r. 87 : 13) was obtained in 64% yield and 4 : 96 e.r. (-92% ee).
Column IA, elution with n-hexane / ^{i}PrOH 6/4, flow 1.0 mL/min.
Retention times for the enantiomers of the major diastereoisomer: 8.5 min. and 21.2 min.
Retention times for the enantiomers of the minor diastereoisomer: 11.0 and 20.2 min.
$[\alpha]_D^{20}$ = -19.9° (1 g/ 100 mL chloroform).

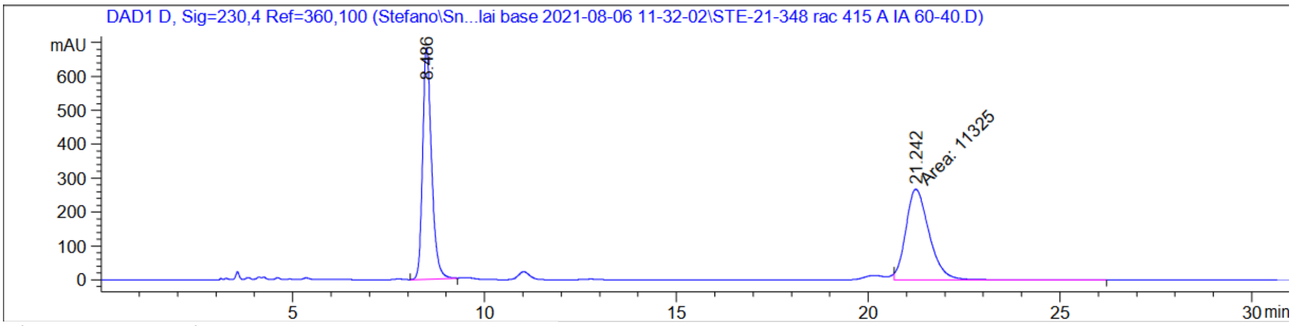

Signal 4: DAD1 D, Sig=230,4 Ref=360,100

Peak #	RetTime [min]	Type	Width [min]	Area [mAU*s]	Height [mAU]	Area %
1	8.486	BB	0.2471	1.10719e4	683.02692	49.4351
2	21.242	FM	0.7048	1.13250e4	267.79468	50.5649
Totals :				2.23968e4	950.82159	

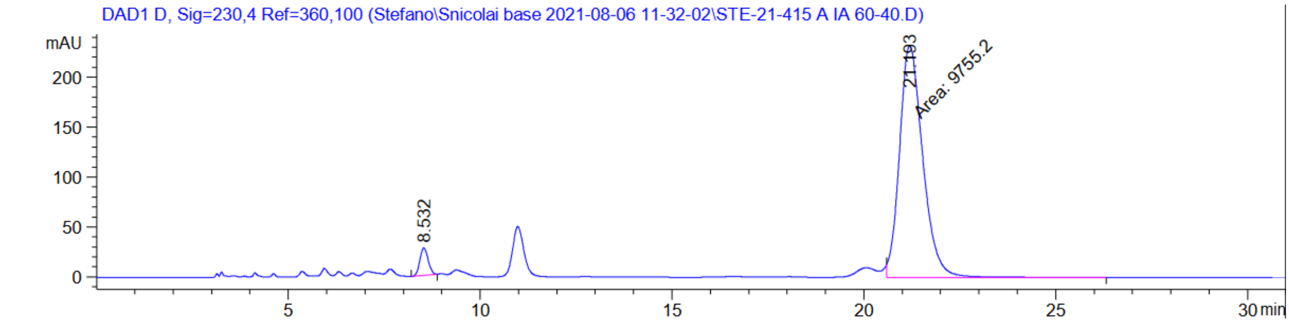

Signal 4: DAD1 D, Sig=230,4 Ref=360,100

Peak #	RetTime [min]	Type	Width [min]	Area [mAU*s]	Height [mAU]	Area %
1	8.532	BB	0.2319	411.70923	27.61604	4.0495
2	21.193	FM	0.7004	9755.20215	232.14400	95.9505
Totals :				1.01669e4	259.76004	

Enantioenriched dibenzyl (2*R*,5*R*)-5-(3-iodo-4-methoxyphenyl)-7-oxo-2-phenylazepane-3,3-dicarboxylate (5a.e)
Following the **GP7** and starting from dibenzyl 2-(3-iodo-4-methoxyphenyl)cyclopropane-1,1-dicarboxylate (**2e**) (0.054 g, 0.10 mmol, 1.0 equiv.) and N-benzylidene-1-((*tert*-butyldimethylsilyl)oxy)ethenamine (**4a**) (0.039 g, 0.15 mmol, 1.5 equiv.), the title compound (0.030 g, 0.045 mmol; d.r. 86 : 14) was obtained in 45% yield and 5 : 95 e.r. (-90% ee).
Column IA, elution with n-hexane / ^{i}PrOH 6/4, flow 1.0 mL/min.
Retention times for the enantiomers of the major diastereoisomer: 7.9 min. and 20.8 min.
Retention times for the enantiomers of the minor diastereoisomer: 10.3 and 16.3 min.
$[\alpha]_D^{20}$ = -19.2° (1 g/ 100 mL chloroform).

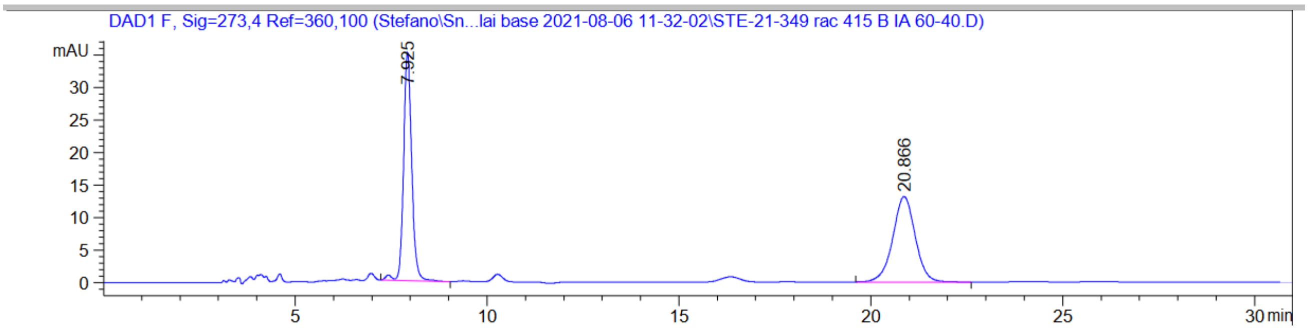

Signal 6: DAD1 F, Sig=273,4 Ref=360,100

Peak #	RetTime [min]	Type	Width [min]	Area [mAU*s]	Height [mAU]	Area %
1	7.925	VB R	0.2271	520.71826	35.10513	49.4112
2	20.866	BB	0.6199	533.12787	13.12455	50.5888
Totals :				1053.84613	48.22967	

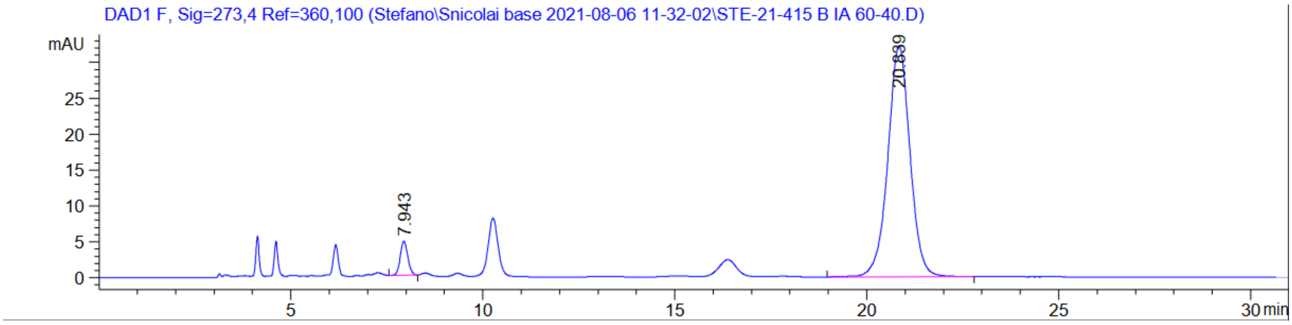

Signal 6: DAD1 F, Sig=273,4 Ref=360,100

Peak #	RetTime [min]	Type	Width [min]	Area [mAU*s]	Height [mAU]	Area %
1	7.943	BB	0.2094	65.02882	4.75830	4.7932
2	20.839	BB	0.6124	1291.65820	32.17176	95.2068
Totals :				1356.68702	36.93007	

Enantioenriched dibenzyl (2*R*,5*R*)-7-oxo-2-phenyl-5-((E)-styryl)azepane-3,3-dicarboxylate (5a.k)

Following the **GP7** and starting from dibenzyl (*E*)-2-styrylcyclopropane-1,1-dicarboxylate (**2k**) (0.041 g, 0.10 mmol, 1.0 equiv.).) and N-benzylidene-1-((*tert*-butyldimethylsilyl)oxy)ethenamine (**4a**) (0.039 g, 0.15 mmol, 1.5 equiv.), the title compound (0.018 g, 0.032 mmol; d.r. 81 : 19) was obtained in 32% yield and 7 : 93 e.r. (-86% ee).

Column IA, elution with n-hexane / ^{i}PrOH 6/4, flow 1.0 mL/min.

Retention times for the enantiomers of the major diastereoisomer: 7.8 min. and 14.9 min.

Retention time for minor diastereoisomer: 10.3 min.

$[\alpha]_D^{20}$ = -8.6° (0.75 g/100 mL in chloroform)

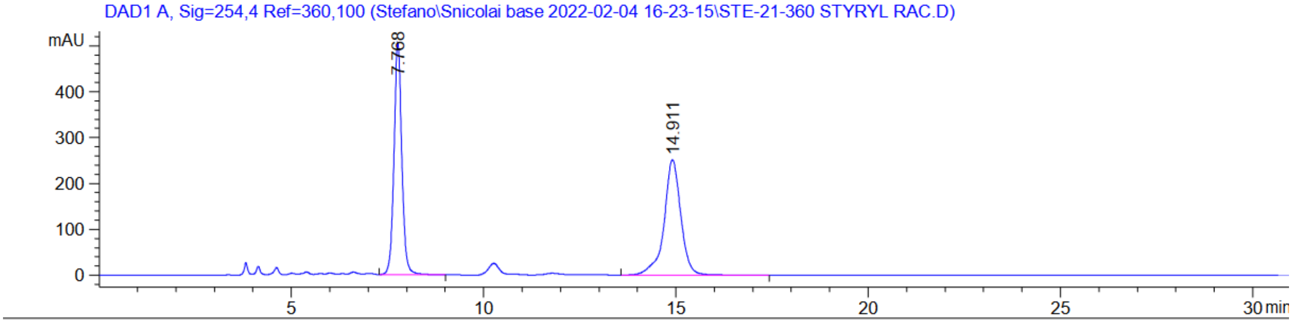

Signal 1: DAD1 A, Sig=254,4 Ref=360,100

Peak #	RetTime [min]	Type	Width [min]	Area [mAU*s]	Height [mAU]	Area %
1	7.768	BB	0.2144	7120.16846	505.30850	48.3217
2	14.911	BB	0.4519	7614.76123	251.05695	51.6783

Totals : 1.47349e4 756.36545

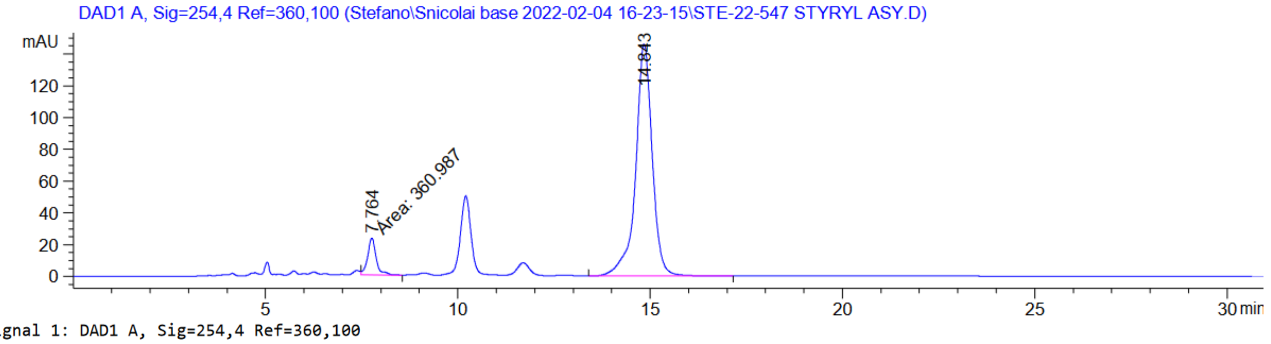

Signal 1: DAD1 A, Sig=254,4 Ref=360,100

Peak #	RetTime [min]	Type	Width [min]	Area [mAU*s]	Height [mAU]	Area %
1	7.764	FM	0.2593	360.98682	23.20094	7.5976
2	14.843	BB	0.4509	4390.33398	145.95595	92.4024

Totals : 4751.32080 169.15688

Enantioenriched dibenzyl (2*R*,5*R*)-5-(4-methoxyphenyl)-7-oxo-2-(4-(trifluoromethyl)phenyl)azepane-3,3-dicarboxylate (5b.a)

Following the **GP7** and starting from dibenzyl 2-(2,4-dimethoxyphenyl)cyclopropane-1,1-dicarboxylate (**2a**) (0.042 g, 0.10 mmol, 1.0 equiv.) and N-(1-((*tert*-butyldimethylsilyl)oxy)vinyl)-1-(4-trifluoromethylphenyl)methanimine (**4b**) (0.049 g, 0.15 mmol, 1.5 equiv.) but using $Cu(OTf)_2$ (3.6 mg, 0.010 mmol, 20 mol%) and (*S*)-CyTox (**L5**) (5.5 mg, 0.011 mmol, 11 mol%), the title compound (0.029 g, 0.046 mmol; d.r. > 95 : 5) was obtained in 46% yield and 1.5 : 98.5 e.r. (-97% ee).

Column IA, elution with n-hexane / ^{i}PrOH 6/4, flow 1.0 mL/min.

Retention times for enantiomers of the major diastereoisomer: 7.3 min. and 14.7 min.

$[\alpha]_D^{20}$ = -34.7° (1 g/ 100 mL chloroform).

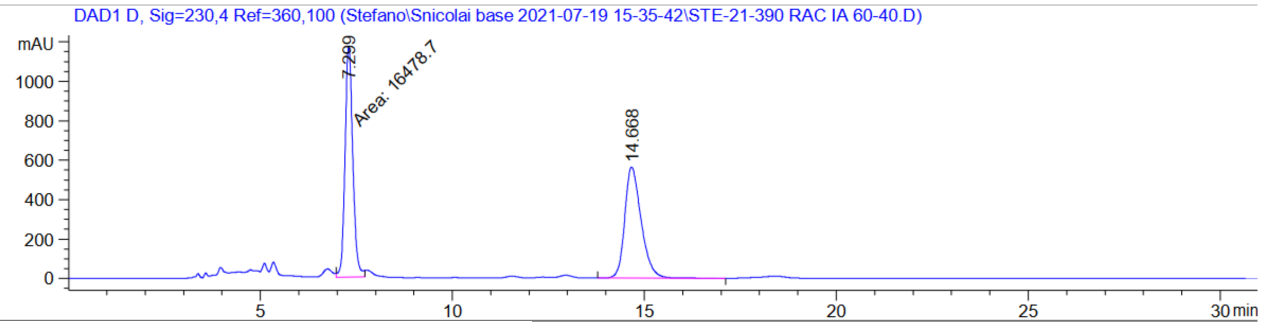

Signal 4: DAD1 D, Sig=230,4 Ref=360,100

Peak #	RetTime [min]	Type	Width [min]	Area [mAU*s]	Height [mAU]	Area %
1	7.299	MF	0.2349	1.64787e4	1169.17261	49.5827
2	14.668	BB	0.4532	1.67560e4	563.10956	50.4173

Totals : 3.32347e4 1732.28217

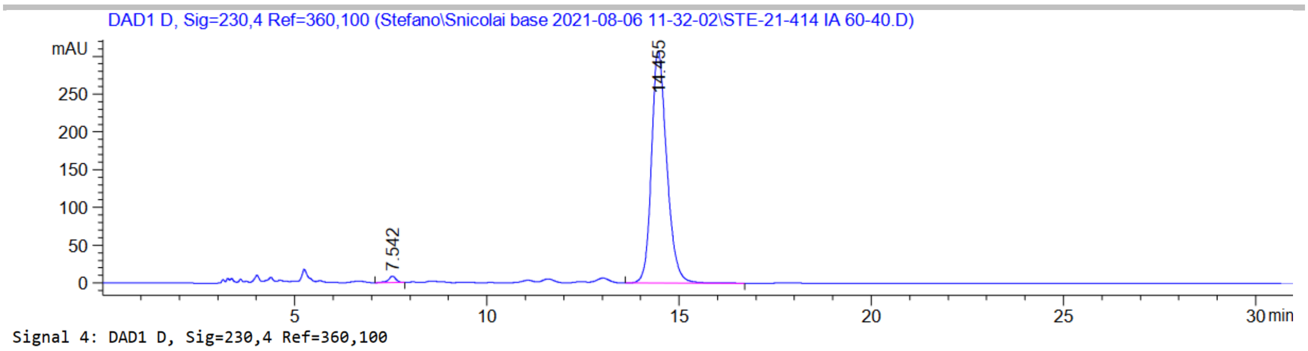

Signal 4: DAD1 D, Sig=230,4 Ref=360,100

Peak #	RetTime [min]	Type	Width [min]	Area [mAU*s]	Height [mAU]	Area %
1	7.542	BB	0.2057	117.63769	8.59404	1.3652
2	14.455	BB	0.4229	8499.43457	307.12939	98.6348
Totals :				8617.07226	315.72343	

Enantioenriched dibenzyl (2*R*,5*R*)-2-(4-chlorophenyl)-5-(4-methoxyphenyl)-7-oxoazepane-3,3-dicarboxylate (5c.a)

Following the **GP7** and starting from dibenzyl 2-(2,4-dimethoxyphenyl)cyclopropane-1,1-dicarboxylate (**2a**) (0.042 g, 0.10 mmol, 1.0 equiv.) and N-(1-((tert-butyldimethylsilyl)oxy)vinyl)-1-(4-chlorophenyl)methanimine (**4c**) (0.044 g, 0.15 mmol, 1.5 equiv.), the title compound (0.040 g, 0.067 mmol; d.r. 93 : 7) was obtained in 67% yield and 4 : 96 e.r. (-92% ee).

Column IA, elution with n-hexane / ^{i}PrOH 6/4, flow 1.0 mL/min.

Retention times for the enantiomers of the major diastereoisomer: 8.7 min. and 19.1 min.

Retention times for the enantiomers of the minor diastereoisomer: 8.0min. and 14.8 min.

$[\alpha]_D^{20}$ = -28.0 °C (1 g/ 100 mL chloroform)

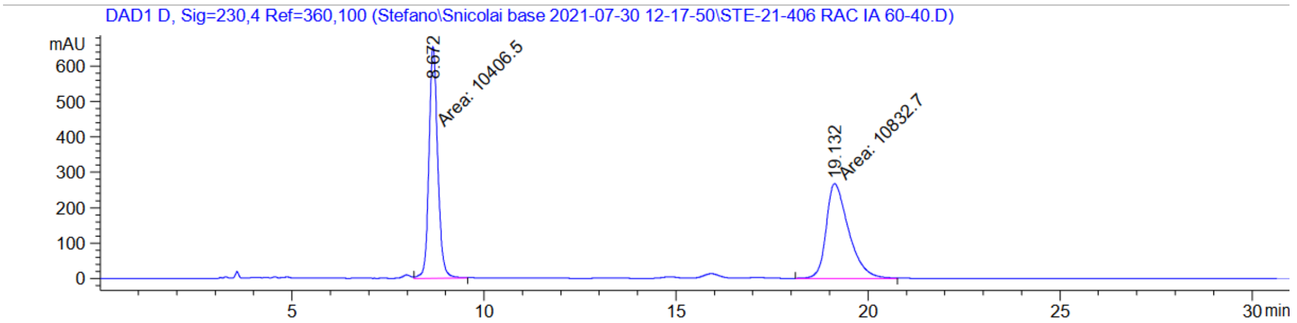

Signal 4: DAD1 D, Sig=230,4 Ref=360,100

Peak #	RetTime [min]	Type	Width [min]	Area [mAU*s]	Height [mAU]	Area %
1	8.672	FM	0.2647	1.04065e4	655.12988	48.9967
2	19.132	MM T	0.6754	1.08327e4	267.32855	51.0033
Totals :				2.12392e4	922.45844	

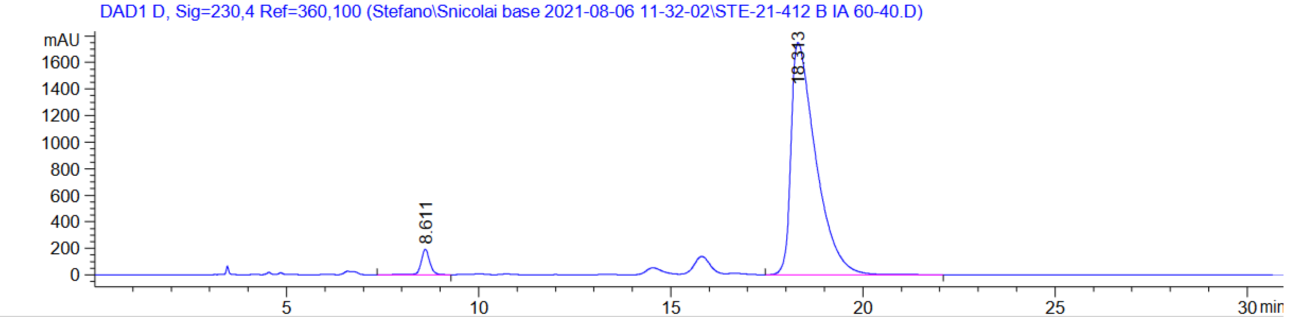

Signal 4: DAD1 D, Sig=230,4 Ref=360,100

Peak #	RetTime [min]	Type	Width [min]	Area [mAU*s]	Height [mAU]	Area %
1	8.611	VB R	0.2366	3007.94580	190.88040	3.6879
2	18.313	BB	0.6660	7.85537e4	1748.89294	96.3121
Totals :				8.15617e4	1939.77335	

Enantioenriched dibenzyl (2*S*,5*R*)-2-(2-fluorophenyl)-5-(4-methoxyphenyl)-7-oxoazepane-3,3-dicarboxylate (5d.a)
Following the **GP7** and starting from dibenzyl 2-(2,4-dimethoxyphenyl)cyclopropane-1,1-dicarboxylate (**2a**) (0.042 g, 0.10 mmol, 1.0 equiv.) and N-(1-((*tert*-butyldimethylsilyl)oxy)vinyl)-1-(2-fluorophenyl)methanimine (**4d**) (0.042 g, 0.15 mmol, 1.5 equiv.), the title compound (0.025 g, 0.042 mmol; d.r. 95 : 5) was obtained in 42% yield and 4 : 96 e.r. (-92% ee).
Column IA, elution with n-hexane / ^{i}PrOH 6/4, flow 1.0 mL/min.
Retention times for enantiomers of the major diastereoisomer: 7.2 min. and 13.6 min.
$[\alpha]_D^{20}$ = -50.4° (1 g/ 100 mL chloroform).

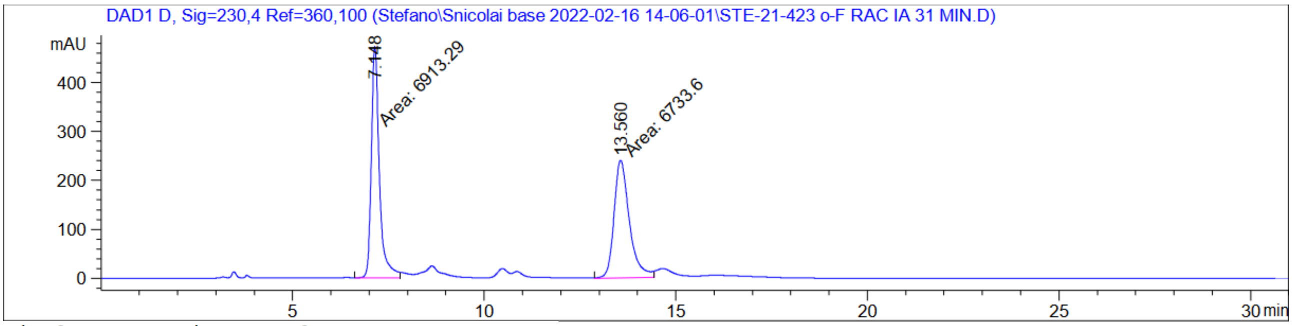

Signal 4: DAD1 D, Sig=230,4 Ref=360,100

Peak #	RetTime [min]	Type	Width [min]	Area [mAU*s]	Height [mAU]	Area %
1	7.148	MF	0.2439	6913.28564	472.43729	50.6583
2	13.560	MF	0.4673	6733.60059	240.14514	49.3417
Totals :				1.36469e4	712.58243	

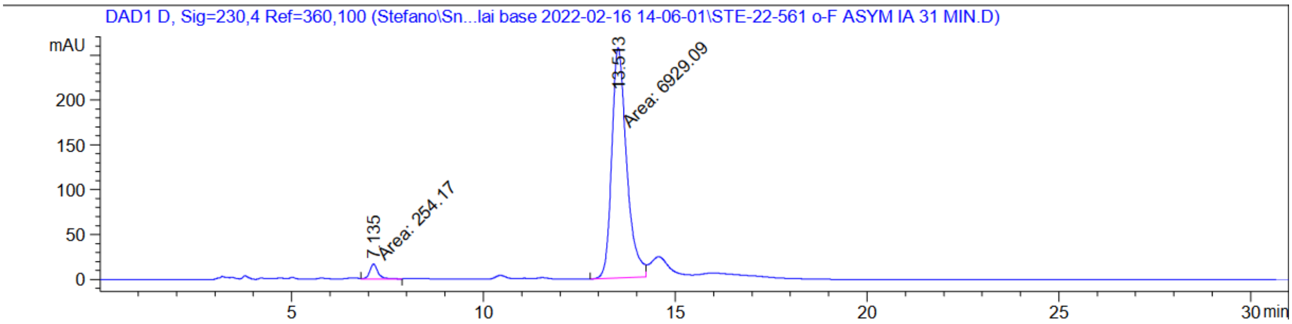

Signal 4: DAD1 D, Sig=230,4 Ref=360,100

Peak #	RetTime [min]	Type	Width [min]	Area [mAU*s]	Height [mAU]	Area %
1	7.135	MM	0.2511	254.16978	16.86932	3.5384
2	13.513	MF	0.4500	6929.08789	256.63550	96.4616
Totals :				7183.25768	273.50482	

Enantioenriched dibenzyl (2*R*,5*R*)-2-(3-bromophenyl)-5-(4-methoxyphenyl)-7-oxoazepane-3,3-dicarboxylate (5e.a)
Following the **GP7** and starting from dibenzyl dibenzyl 2-(2,4-dimethoxyphenyl)cyclopropane-1,1-dicarboxylate (**2a**) (0.042 g, 0.10 mmol, 1.0 equiv.) and (*E*)-N-(1-((*tert*-butyldimethylsilyl)oxy)vinyl)-1-(3-bromophenyl)methanimine (**4e**) (0.051 g, 0.15 mmol, 1.5 equiv.), the title compound (0.037 g, 0.058 mmol; d.r. 92 : 8) was obtained in 58% yield and 6.5 : 93.5 e.r. (-87% ee).
Column IA, elution with n-hexane / ^{i}PrOH 6/4, flow 1.0 mL/min.
Retention times for the enantiomers of the major diastereoisomer: 7.5 min. and 14.7 min.
Retention time for minor diastereoisomer: 12.2 min.
$[\alpha]_D^{20}$ = -156.5° (1 g / 100 mL chloroform)

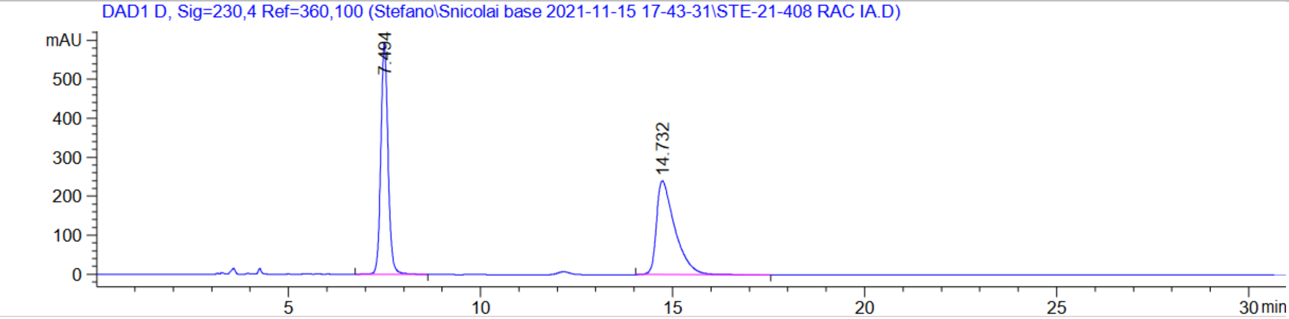

Signal 4: DAD1 D, Sig=230,4 Ref=360,100

Peak #	RetTime [min]	Type	Width [min]	Area [mAU*s]	Height [mAU]	Area %
1	7.494	BB	0.2006	7784.63281	595.24341	49.3103
2	14.732	BB	0.4876	8002.38721	240.86739	50.6897

Totals : 1.57870e4 836.11079

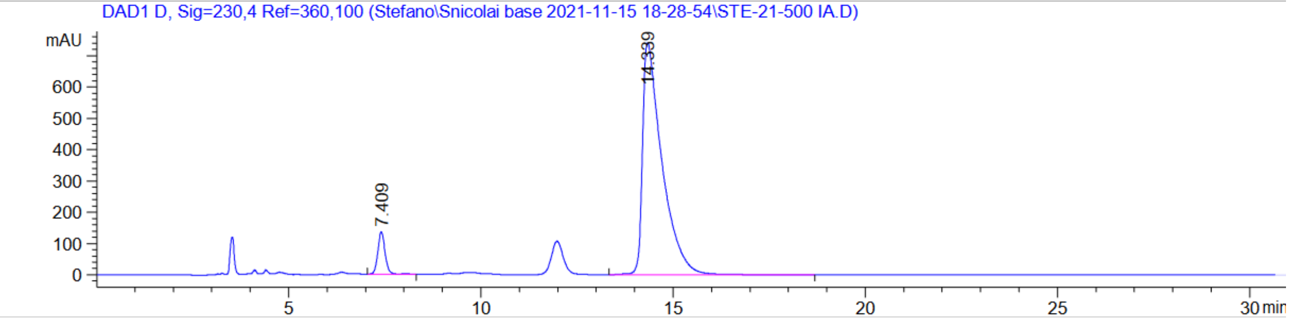

Signal 4: DAD1 D, Sig=230,4 Ref=360,100

Peak #	RetTime [min]	Type	Width [min]	Area [mAU*s]	Height [mAU]	Area %
1	7.409	BV R	0.1987	1772.92871	135.20879	6.3776
2	14.339	BB	0.5038	2.60263e4	740.90015	93.6224

Totals : 2.77993e4 876.10893

6. Modifications of the (4+3) annulation products

Hydrogenolysis/decarboxylation of the cycloadduct 5a.a: synthesis of 5-(4-methoxyphenyl)-7-oxo-2-phenylazepane-3-carboxylic acid (6)

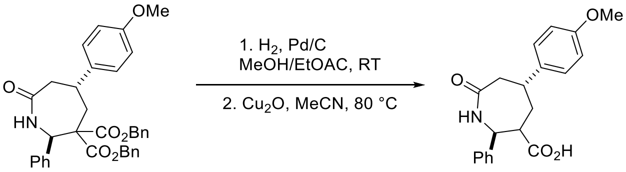

5a.a **6**

A 25 mL, single-necked, round-bottom flask was charged with Pd/C (5% Pd on carbon; 0.16 g, 0.076 mmol, 10 mol%) and dibenzyl 5-(4-methoxyphenyl)-7-oxo-2-phenylazepane-3,3-dicarboxylate (**5a.a**) (0.430 g, 0.763 mmol, 1.0 equiv.). The flask was sealed with a septum, evacuated and back-filled twice with nitrogen and then with hydrogen. Methanol (2.2 mL) and EtOAc (2.2 mL) were added, leading to the formation of a homogeneous suspension. The latter was flushed with hydrogen (balloon) for 15 minutes. The mixture was then stirred at room temperature under an atmosphere of hydrogen. After 3 hours, full conversion was observed according to TLC analysis (pentane/EtOAc 4/6). One single, much more polar new product was formed (DCM/MeOH 9/1). Celite was added to the mixture, and the solids were filtered-off through a pad of celite, which was then washed with DCM/MeOH (9/1). The filtrate was concentrated under reduced pressure, to provide an off-white, poudry solid (ca. 290 mg, corresponding to quantitative yield for the debenzylation reaction), which was not purified and used directly in the following step.

Following a reported procedure,[50] inside a glove-box, a 50 mL, two-necked, round-bottom vial was charged with copper(I) oxide (red powder; 11 mg, 0.076 mmol, 10 mol%). Outside the glovebox, the flask was rapidly equipped with a Liebig condenser, and the whole apparatus was evacuated and back-filled with nitrogen. Acetonitrile (27 mL) was then added, followed by the solid obtained from the previous step. The milky suspension was then heated to 80 °C, which initially led it to become a clear, colorless

solution. It was stirred at this temperature for 20 hours, turning to green-blueish during this time. The mixture was then allowed to cool to room temperature, and concentrated under vacuum. Water (10 mL) and aq. HCl (1 M; 10 mL) were then added. The aqueous layer was extracted with EtOAc (4 x 25 mL). The combined organic layers were washed with brine, dried over $MgSO_4$, and concentrated under vacuum. The resulting pale yellow solid was submitted to column chromatography (Biotage flash chromatographer, 12 g SiO_2; MeOH in DCM, 1 to 12%) to afford 5-(4-methoxyphenyl)-7-oxo-2-phenylazepane-3-carboxylic acid (**6**) (0.240 g, 0.707 mmol, 93% yield; mixture of two diastereoisomers, d.r. = 50 : 50) as an off-white solid.

R_f (DCM/MeOH 95/5) = 0.2 - 0.3
^{1}H NMR (400 MHz, DMSO-d_6; the resolution of the signals corresponding to the two diastereoisomers is not complete) δ 12.08 (br s, 1H, CO_2H), 7.51 (d, *J* = 7.5 Hz, 1H, Ar*H*), 7.45 (d, *J* = 6.9 Hz, 0.5H, (CO)N*H*), 7.40 – 7.31 (m, 3H, Ar*H*), 7.29 (m, 1H, Ar*H*), 7.22 (m, 1H, Ar*H*), 7.16 (m, 1H, Ar*H*), 7.12 (d, *J* = 3.8 Hz, 0.5H, (CO)N*H*), 6.89 (dd, *J* = 8.7, 1.5 Hz, 2H, Ar*H*), 5.20 (d, *J* = 7.0 Hz, 0.5H, NC*H*Ph), 4.79 (dd, *J* = 9.4, 4.8 Hz, 1H, NC*H*Ph), 3.73 (s, 3H, O*Me*), 3.33 (d, *J* = 6.8 Hz, 2H, CH_2), 3.15 – 3.06 (m, 1H, C*H*), 3.02 – 2.89 (m, 1H, C*H*), 2.22 (d, *J* = 13.2 Hz, 1H, CH_2), 2.18 – 2.11 (m, 1H, CH_2), 2.11 – 2.05 (m, 1H, CH_2).
^{13}C NMR (101 MHz, DMSO-d_6; the resolution of the signals corresponding to the two diastereoisomers is not complete) δ 174.6, 174.4, 173.6, 158.1, 158.1, 140.9, 140.4, 139.5, 128.7, 128.5, 128.2, 127.9, 127.8, 127.3, 114.4, 58.6, 56.9, 55.5, 53.2, 47.9, 43.4, 42.9, 38.8, 36.4.
HRMS (ESI/QTOF) m/z: $[M + H]^+$ Calcd for $C_{20}H_{22}NO_4^+$ 340.1543; Found 340.1542.

- **Hydrogenolysis/decarboxylation of the enantioenriched product 5a.a:**

The same procedure as the one described above was implemented on enantioenriched **5a.a** dibenzyl 5-(4-methoxyphenyl)-7-oxo-2-phenylazepane-3,3-dicarboxylate (0.52 g, 0.93 mmol, 1.0 equiv.), obtained followed the asymmetric protocol. Eantioenriched **6** (0.255 g, 0.751 mmol) was obtained in 81% yield (mixture of epimers, d.r. 50 : 50).

Decarboxylative alkynylation of carboxylic acid 6: synthesis of 4-(4-methoxyphenyl)-7-phenyl-6-(phenylethynyl)azepan-2-one (7)

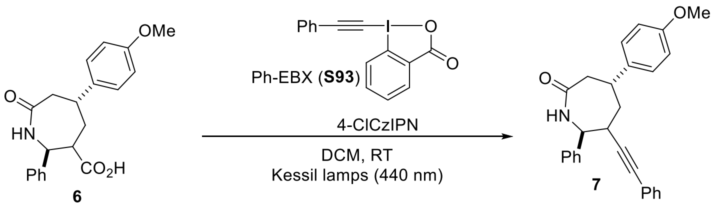

Following a modified version of a reported procedure,[51] inside a glove-box, a 10 mL, flat-bottomed vial was charged with cesium carbonate (75 mg, 0.23 mmol, 2.3 equiv.), 5-(4-methoxyphenyl)-7-oxo-2-phenylazepane-3-carboxylic acid (**6**) (35 mg, 0.10 mmol, 1.0 equiv.), 4-ClCzIPN (5.5 mg, 0.0052 mmol, 5 mol%) and Ph-EBX (**S93**) (63 mg, 0.18 mmol, 1.8 equiv.). The tube was closed with a septum, and taken out of the glove-box. DCM (degassed by freeze-pump-thaw method (3 times); 5.0 mL, 0.020 M) was added. The septum was rapidly replaced with a screw cap under a stream of nitrogen. The slightly turbid, bright yellow mixture was subsequently irradiated (Kessil lamp; 440 nm) at room temperature under stirring overnight. After 15 hours, the mixture looked like an orange suspension, with significant precipitation of a whitish solid. TLC analysis showed at this point that all the starting material had been consumed. Celite was added, and the volatiles were removed by distillation under reduced pressure. The crude product was submitted to column chromatography (dry load; Biotage flash chromatographer, 4 g; EtOAc in pentane, 15 to 45%) to provide 4-(4-methoxyphenyl)-7-phenyl-6-(phenylethynyl)azepan-2-one (**7**) (mixture of diastereoisomers. d.r. ca 2 : 1; 21 mg, 0.053 mmol, 51% yield) as a pale orange solid.

R_f (pentane/EtOAc 1 : 1) = 0.26.
^{1}H NMR (400 MHz, Chloroform-*d*; the signals that could be assigned to the *major isomer* are in bold) δ 7.50 – 7.28 (m, 10H, Ar*H*), 7.23 – 7.05 (m, 7H, Ar*H*), 6.96 – 6.84 (m, 5H, Ar*H*), **5.86** (d, *J* = 3.7 Hz, 1H, (CO)N*H*), 5.78 (s, 0.5H, (CO)N*H*), 4.61 – 4.53 (m, 1.5H, PhC*H*N), **4.58** (dd, *J* = 9.3, 4.2 Hz, 1H, PhC*H*N), **3.81** (s, 3H, O*Me*), 3.80 (s, 1.5H, O*Me*), 3.23 (ddd, *J* = 12.1, 9.2, 3.2 Hz, 1H, C*H* or CH_2), 3.15 – 2.92 (m, 3.5H, C*H* or CH_2), 2.75 – 2.66 (m, 2H, C*H* or CH_2), 2.61 (dt, *J* = 13.7, 2.8 Hz, 1H, C*H* or CH_2), 2.26 – 2.15 (m, 1.5H, C*H* or CH_2), 2.08 (td, *J* = 7.4, 4.0 Hz, 1H, C*H* or CH_2), 1.92 (m, 0.5H, C*H* or CH_2).
^{13}C NMR (101 MHz, Chloroform-*d*; the resolution of the signals corresponding to the two diastereoisomers is not complete) δ 175.5, 175.3, 158.4, 158.2, 142.3, 140.9, 139.1, 138.3, 131.3, 129.3, 129.0, 128.6, 128.4, 128.0, 127.8, 127.3, 127.3, 126.3, 122.9, 114.2, 114.0, 89.6, 84.8, 62.3, 58.7, 55.3, 46.4, 44.5, 43.6, 40.3, 39.9, 39.6, 39.3, 37.5.
IR (v_{max}, cm^{-1}) 3209 (w), 3069 (w), 2936 (w), 2835 (w), 1655 (s), 1512 (m), 1438 (m), 1410 (w), 1302 (w), 1248 (m), 1180 (w), 1033 (w), 911 (m), 832 (m), 760 (m), 734 (m), 698 (m)
HRMS (ESI/QTOF) m/z: $[M + Na]^+$ Calcd for $C_{27}H_{25}NNaO_2^+$ 418.1777; Found 418.1777.

Barton decarboxylation of carboxylic acid 6: synthesis of 4,7-*trans*-4-(4-methoxyphenyl)-7-phenylazepan-2-one (8)

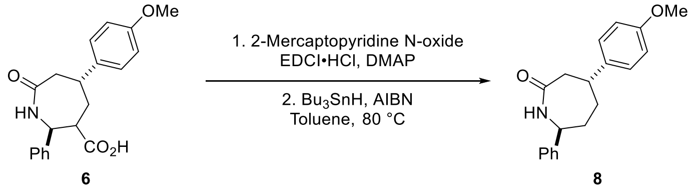

Following a reported procedure,[52] a 25 mL, two-necked, round-bottom flask was charged with 5-(4-methoxyphenyl)-7-oxo-2-phenylazepane-3-carboxylic acid (**6**) (51 mg, 0.15 mmol, 1.0 equiv.) and DCM (2.2 mL). The flask was wrapped with aluminium foil to protect the mixture from light, and the latter was cooled to 0 °C (ice - water bath). 2-Mercaptopyridine N-oxide (24 mg, 0.30 mmol, 2.0 equiv.), EDC●HCl (58 mg, 0.30 mmol, 2.0 equiv.), and a catalytic amount of DMAP (4 mg, 0.03 mmol, 20 mol%) were added. The resulting mixture was stirred at 0 °C for 4 hours, becoming a clear, yellow solution during this time. It was then diluted with DCM (15 mL) and washed with sat. aq. $NaHCO_3$ (15 mL). The aqueous layer was extracted with DCM (3 x 15 mL). The combined organic layers were washed with brine, dried over Na_2SO_4, filtered, and concentrated under vacuum. The resulting yellow-orange crude product was then submitted to column chromatography (Biotage flash chromatographer, 4 g SiO_2; MeOH in DCM, 0 to 12%) to furnish 2-thioxopyridin-1(2*H*)-yl 5-(4-methoxyphenyl)-7-oxo-2-phenylazepane-3-carboxylate (mixture of diastereoisomers; 63 mg, 0.14 mmol, 93% yield) as a greenish solid.

The solid obtained from the previous step was dissolved in toluene (3.0 mL), and the resulting grey-green solution was introduced by syringe into a sealed, 25 mL vial already containing AIBN (2.5 mg, 0.015 mmol, 10 mol%) under nitrogen. Finally, tri-*n*-butyl stannane (0.12 mL, 0.45 mmol, 3.0 equiv.) was also added, and the mixture was heated to 80 °C, in the dark. It converted into a pale yellow suspension, which then further turned into a colorless and clear solution. After 5 hours, full conversion was observed based on TLC analysis (pentane/EtOAc 5/5). The mixture was allowed to cool down to room temperature, diluted with EtOAc, and washed with an aq. saturated solution of KF. The aqueous layer was then extracted with EtOAc (4 x 10 mL). The combined organic layers were washed with brine, dried over Na_2SO_4, filtered, and concentrated under vacuum. The resulting crude solid was submitted to column chromatography (Biotage flash chromatographer, 4 g SiO_2; MeOH in DCM, 1 to 10%) to afford 4,7-*trans*-4-(4-methoxyphenyl)-7-phenylazepan-2-one (**8**) (0.020 g, 0.068 mmol, 45% yield; 42% yield over two steps from **6**) as a white solid.

R_f (pentane/EtOAc 5 : 5) 0.13.
^{1}H NMR (400 MHz, Chloroform-*d*) δ 7.42 – 7.36 (m, 2H, Ph*H*), 7.36 – 7.28 (m, 3H, Ph*H*), 7.18 – 7.10 (m, 2H, Ar*H*), 6.90 – 6.82 (m, 2H, Ar*H*), 5.77 (br s, 1H, (CO)N*H*), 4.55 (dt, *J* = 7.9, 4.1 Hz, 1H, NC*H*Ph), 3.80 (s, 3H, O*Me*), 3.07 – 2.90 (m, 2H, C*H* or C*H*₂), 2.68 (dt, *J* = 12.4, 2.2 Hz, 1H, C*H* or C*H*₂), 2.19 (m, 1H, C*H* or C*H*₂), 2.08 (dq, *J* = 7.7, 3.9 Hz, 2H, C*H* or C*H*₂), 1.92 (m, 1H, C*H* or C*H*₂).
^{13}C NMR (101 MHz, $CDCl_3$) δ 175.5, 158.2, 142.3, 139.1, 129.3, 128.4, 127.3, 126.3, 114.0, 58.7, 55.3, 44.5, 39.9, 39.3, 37.5.
IR ($ν_{max}$, cm^{-1}) 3383 (w), 3252 (w), 3064 (w), 2929 (m), 1652 (s), 1513 (s), 1444 (m), 1306 (w), 1247 (m), 1181 (w), 1035 (w), 911 (m), 827 (m), 759 (m), 736 (m)
HRMS (ESI/QTOF) m/z: $[M + Na]^+$ Calcd for $C_{19}H_{21}NNaO_2^+$ 318.1464; Found 318.1466.
Melting point: 188.7 - 190.6 °C.

- **Barton decarboxylation of enantioenriched carboxylic acid 6:**

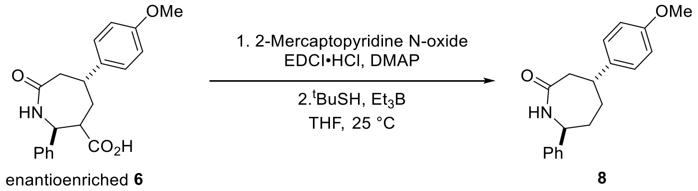

In a 25 mL, two-necked, round-bottomed flask, enantioenriched 5-(4-methoxyphenyl)-7-oxo-2-phenylazepane-3-carboxylic acid (**6**) (0.250 g, 0.736 mmol, 1.0 equiv.) was suspended in DCM (dry; 11 mL). The flask was wrapped with aluminium foil to protect the mixture from light, and the latter was cooled to 0 °C (ice - water bath). 2-Mercaptopyridine N-oxide (0.120 g, 0.944 mmol, 1.28 equiv.), EDC●HCl (0.28 g, 1.5 mmol, 2.0 equiv.), and a catalytic amount of DMAP (18 mg, 0.15 mmol, 20 mol%). The resulting mixture was stirred at 0 °C for 4 hours, becoming a clear, yellow solution during this time. The mixture was then diluted with DCM (15 mL) and washed with sat. aq. $NaHCO_3$ (15 mL). The aqueous layer was extracted with DCM (3 x 15 mL). The combined organic layers were washed with brine, dried over Na_2SO_4, filtered, and concentranted under vacuum. The resulting yellow-orange crude product was then submitted to column chromatography (Biotage, 4 g SiO_2; MeOH in DCM, 0 to 12%) to furnish 2-thioxopyridin-1(2*H*)-yl 5-(4-methoxyphenyl)-7-oxo-2-phenylazepane-3-carboxylate (mixture of diastereoisomers; 0.24 mg, 0.53 mmol, 77% yield) as a green solid.

The latter was dissolved in THF (dry; 3.5 mL), and to the resulting green solution was added 2-methylpropane-2-thiol (0.83 mL; 7.4 mmol, 10 equiv.). After protecting the flask from light (aluminium foil), triethyl borane (1.0 M in hexane; 0.22 mL, 0.22 mmol, 30 mol%) was added drop-wise in the presence of air: the green solution rapidly became pale yellow. The mixture was stirred at

room temperature for 2 hours. It was then treated with sat. aq. $NaHCO_3$ (10 mL). The aqueous layer was extracted with DCM (4 x 15 mL). The combined organic layers were washed with brine, dried over Na2SO4, filtered, and concentrated under vacuum. The resulting yellow crude solid was submitted to column chromatography (Biotage, 4 g SiO_2; EtOAc in pentane, 20 to 60%) to afford 4,7-trans-4-(4-methoxyphenyl)-7-phenylazepan-2-one (**8**) (0.070 g, 0.024 mmol, 32% yield; 25% yield over two steps from enantioenriched **6**) as a white solid.

Reduction of (enantioenriched) amide 8: synthesis of 2,5-*trans*-5-(4-methoxyphenyl)-2-phenylazepane (10)

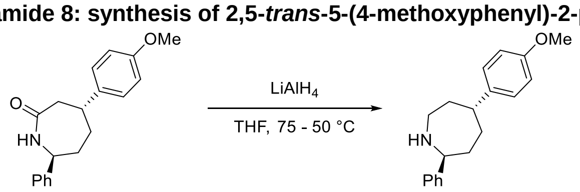

enantioenriched **8** **10**

In a 25 mL, two-necked, round-bottomed flask, equipped with an air-condenser (Findenser), lithium aluminium hydride (0.020 g, 0.53 mmol, 2.5 equiv.) was suspended in THF (0.5 mL). At room temperature, a solution of 4-(4-methoxyphenyl)-7-phenylazepan-2-one (0.062 g, 0.21 mmol, 1.0 equiv.) in THF (2.0 mL) was added drop-wise, with immediate release of gas after each drop. The grey suspension was then stirred at 75 °C for 4 hours, and at 50 °C overnight. When no further advance of conversion (after 20 hours since the beginning of the reaction) was observed according to TLC analysis (pentane/EtOAc 40/60), the mixture was cooled to 0 °C (ice - water), and water (0.020 mL), aq. NaOH (15% w/w; 0.020 mL), and water (0.060 mL) were added in sequence. The suspension was then stirred at room temperature for 20 minutes, turning from grey to yellow-grey. MgSO4 was added, stirring was continued for another 10 minutes, and the solids were filtered off through a pad of celite, and washed with DCM. The filtrate was concentrated under vacuum to provide a yellow crude oil. The latter was submitted to preparative TLC (20 x 20 sq cm SiO_2 plate; elution with DCM/Ultra 80/20). The compound eluted with R_f = 0.77 was deadsorbed from silica using Ultra (20 mL). The so-obtained solution was dried over Na_2SO_4, and concentrated under vacuum to furnish 5-(4-methoxyphenyl)-2-phenylazepane (**10**) (0.033 g, 0.12 mmol, 56% yield) as a white foam.

R_f (DCM/Ultra 80/20) = 0.77
^{1}H NMR (400 MHz, Chloroform-d) δ 7.54 (d, *J* = 7.2 Hz, 2H, Ar*H*), 7.35 (t, *J* = 7.3 Hz, 2H, Ar*H*), 7.29 (d, *J* = 6.9 Hz, 1H, Ar*H*), 7.18 (d, *J* = 8.1 Hz, 2H, Ar*H*), 6.88 (d, *J* = 8.2 Hz, 2H, Ar*H*), 4.74 (s, 1H, N*H*), 4.34 (d, *J* = 10.4 Hz, 1H, NC*H*Ph), 3.81 (s, 3H, O*Me*), 3.32 (d, *J* = 13.7 Hz, 1H, C*H*$_2$), 3.20 (m, 1H, C*H*$_2$), 2.89 (s, 1H, C*H*Ar), 2.39 (q, *J* = 12.0, 11.4 Hz, 1H, C*H*$_2$), 2.24 – 2.02 (m, 4H, CH2), 1.94 (q, *J* = 12.6, 12.1 Hz, 1H, C*H*$_2$).
^{13}C NMR (101 MHz, Chloroform-d) δ 158.1, 138.6, 137.8, 129.0, 129.0, 127.6, 127.6, 114.0, 62.3, 55.3, 45.1, 43.9, 35.4, 33.0, 32.5.
HRMS (ESI/QTOF) m/z: [M + H]+ Calcd for $C_{19}H_{24}NO^+$ 282.1852; Found 282.1854.
$[\alpha]_D^{20}$ = +7.8° (1 g/ 100 mL chloroform)

N-Methylation and reduction of the cycloadduct 6: synthesis of dibenzyl 2-(4-(benzyl(methyl)amino)-2-(4-methoxyphenyl)butyl)malonate (9)

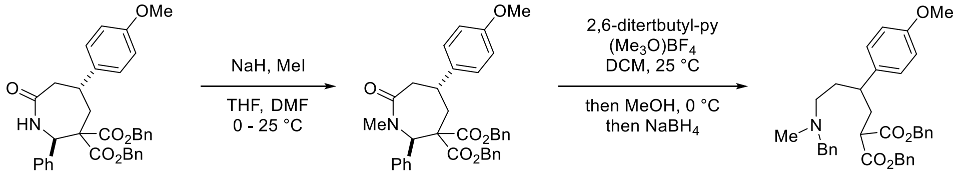

6 **S94** **9**

In a 25 mL, two-necked, round bottom flask, dibenzyl 5-(4-methoxyphenyl)-7-oxo-2-phenylazepane-3,3-dicarboxylate (**6**) (0.30 g, 0.53 mmol, 1.0 equiv.) was dissolved in a mixture of THF (1.5 mL) and DMF (0.7 mL). The clear, colorless solution was cooled to 0 °C (ice - water bath). Methyl iodide (0.10 mL, 1.6 mmol, 3.0 equiv.) was added by syringe. Finally, NaH (60% dispersion in mineral oil; 0.026 g, 0.64 mmol, 1.2 equiv.) was also added in a single portion, upon which the mixture immediately converted into a yellow suspension (some bubbling was observed at this point). The mixture was stirred at 0 °C for 5 minutes, and then at room temperature for 3 hours. The reaction was then quenched by addition of water (2 mL) and sat. aq. NH_4Cl (5 mL). Upon dilution with EtOAc (15 mL), the aqueous layer was separated and extracted with EtOAc (4 x 15 mL). The combined organic layers were washed with brine (3 times), dried over Na_2SO_4, filtered, and concentrated under vacuum. The resulting pale yellow, crude solid was submitted to column chromatography (Biotage flash chromatographer, 4 g SiO_2; MeOH in DCM, 0 to 8%) to provide dibenzyl 5-(4-methoxyphenyl)-1-methyl-7-oxo-2-phenylazepane-3,3-dicarboxylate (**S94**) (0.24 g, 0.41 mmol, 77% yield) as a white foam.

R_f (Pentane/EtOAc 6 : 4) = 0.50.
^{1}H NMR (400 MHz, Chloroform-*d*) δ 7.40 – 7.34 (m, 5H, Ar*H*), 7.34 – 7.20 (m, 7H, Ar*H*), 6.93 (dd, *J* = 11.4, 8.2 Hz, 5H, Ar*H*), 6.83 – 6.74 (m, 2H, Ar*H*), 5.53 (s, 1H, PhC*H*NMe), 5.41 (d, *J* = 12.0 Hz, 1H, *CH*$_2$Ph), 5.19 (d, *J* = 12.0 Hz, 1H, *CH*$_2$Ph), 4.76 (d, *J* = 12.1 Hz, 1H, *CH*$_2$Ph), 4.67 (d, *J* = 12.0 Hz, 1H, *CH*$_2$Ph), 3.77 (s, 3H, O*Me*), 3.31 (m, 1H, C*H*$_2$ or C*H*), 3.06 (m, 1H, C*H*$_2$ or C*H*), 2.87 (d, *J* = 14.5 Hz, 1H, C*H*$_2$ or C*H*), 2.77 (d, *J* = 11.7 Hz, 1H, C*H*$_2$ or C*H*), 2.59 (s, 3H, N*Me*), 2.33 (m, 1H, C*H*$_2$ or C*H*).

^{13}C NMR (101 MHz, Chloroform-*d*; the signals corresponding to the aromatic C are not completely resolved; the signal corresponding to one aliphatic C is not resolved) δ 173.9, 170.2, 168.4, 158.3, 137.7, 136.2, 134.9, 134.3, 128.8, 128.7, 128.6, 128.6, 128.5, 128.3, 127.4, 114.1, 68.0, 67.8, 66.7, 62.8, 55.3, 43.3, 37.3, 33.0.
HRMS (ESI/QTOF) m/z: $[M + H]^+$ Calcd for $C_{36}H_{36}NO_6^+$ 578.2537; Found 578.2550.

Following a reported procedure,[53] a 25 mL, two-necked, round-bottom flask was charged with dibenzyl 5-(4-methoxyphenyl)-1-methyl-7-oxo-2-phenylazepane-3,3-dicarboxylate (**S94**) (0.069 g, 0.12 mmol, 1.0 equiv.), 2,6-di*tert*butyl pyridine (0.092 mL, 0.40 mmol, 3.3 equiv.) and DCM (dry; 3.0 mL). To the resulting clear, colorless solution was added trimethyloxonium tetrafluoroborate (0.053 g, 0.036 mmol, 3.0 equiv.). The resulting suspension was stirred at room temperature for 20 hours. During this time, the salt was completely dissolved, which resulted in the formation of a clear, yellow solution. The latter was cooled to 0 °C (ice - water bath), and MeOH (dry; 1.5 mL) was added. The resulting turbid mixture was stirred at the same temperature for 20 minutes. Finally, sodium borhydride (0.045 g, 1.2 mmol, 10 equiv.) was added, followed by stirring at 0 °C for another 30 minutes. Sat. aq. $NaHCO_3$ (5 mL) and DCM (15 mL) were added. The layers were separated, and the aqueous one was extracted with DCM (5 x 15 mL). The combined organic layers were dried over Na_2SO_4, filtered, and concentrated under vacuum. The resulting crude, yellow oil was submitted to column chromatography (Biotage flash chromatographer, 4 g SiO_2; MeOH in DCM, 0 to 10%) to provide dibenzyl 2-(4-(benzyl(methyl)amino)-2-(4-methoxyphenyl)butyl)malonate (**9**) (90% pure; 0.032 g, 0.051 mmol, 42% yield) as a pale yellow oil. A high purity sample was obtained by preparative TLC (20 x 20 sq cm plate, SiO_2; elution with DCM/MeOH 19/1).

R_f (pentane/EtOAc 6 : 4) 0.45.
^{1}H NMR (400 MHz, Chloroform-*d*) δ 7.38 – 7.22 (m, 15H, Ph*H*), 6.97 (m, 2H, Ar*H*), 6.80 (m, 2H, Ar*H*), 5.20 (d, *J* = 12.3 Hz, 1H, O*CH₂*Ph), 5.16 (d, *J* = 12.3 Hz, 1H, O*CH₂*Ph), 5.07 (d, *J* = 12.4 Hz, 1H, O*CH₂*Ph), 5.02 (d, *J* = 12.4 Hz, 1H, O*CH₂*Ph), 3.80 (s, 3H, O*Me*), 3.45 (d, *J* = 12.8 Hz, 1H, N*CH₂*Ph), 3.39 (d, *J* = 12.9 Hz, 1H, N*CH₂*Ph), 3.25 (dd, *J* = 10.1, 4.9 Hz, 1H, $(BnO_2C)_2$C*H*), 2.58 (tt, *J* = 9.6, 4.7 Hz, 1H, C*H*), 2.36 (ddd, *J* = 14.4, 10.2, 4.5 Hz, 1H, C*H₂*), 2.27 (m, 1H, C*H₂*), 2.16 (m, 2H, C*H₂*), 2.12 (s, 3H, N*Me*), 1.89 (m, 1H, C*H₂*), 1.78 (m, 1H, C*H₂*).
^{13}C NMR (101 MHz, Chloroform-*d*; the signal corresponding to one aromatic C is not resolved; the two benzylic C signals are not resolved from each other) δ 169.2, 169.1, 158.2, 135.5, 135.4, 129.1, 128.7, 128.5, 128.5, 128.4, 128.3, 128.3, 128.2, 128.0, 114.0, 67.0, 62.1, 55.3, 55.2, 50.2, 41.9, 40.7, 35.8, 29.7.
HRMS (ESI/QTOF) m/z: $[M + H]^+$ Calcd for $C_{36}H_{40}NO_5^+$ 566.2901; Found 566.2909.

Cleavage of the phthalimido group of cycloadduct 5a.l: synthesis of benzyl-4,8-dioxo-2-phenyl-3,7-diazabicyclo[4.2.1]nonane-1-carboxylate (11)

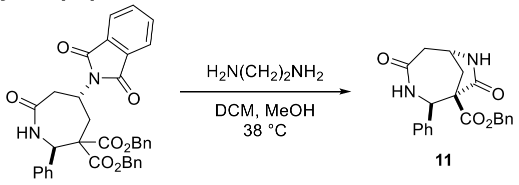

Following a reported procedure,[54] in a sealed 25 mL, round-bottom vial, dibenzyl 5-(1,3-dioxoisoindolin-2-yl)-7-oxo-2-phenylazepane-3,3-dicarboxylate (**5a.l**) (0.090 g, 0.15 mmol, 1.0 equiv.) was dissolved in DCM (dry; 0.75 mL) and MeOH (dry; 0.75 mL). Ethylene diamine (0.050 mL, 0.75 mmol, 5.0 equiv.) was added by syringe. The resulting pale yellow, clear solution was stirred at 38 °C overnight. After 20 hours, TLC analysis (DCM/MeOH 95/5) showed the complete conversion of the starting material. The mixture was concentrated under reduced pressure. The resulting yellow, crude oil was submitted to column chromatography (Biotage, 4 g SiO_2; MeOH in DCM, 0 to 8%) to provide benzyl 4,8-dioxo-2-phenyl-3,7-diazabicyclo[4.2.1]nonane-1-carboxylate (**11**) (0.039 g, 0.11 mmol, 73% yield) as a white, light powder.

Melting point: 156.5 - 159.8 °C.
R_f (DCM/MeOH 9 : 1) 0.31.
^{1}H NMR (400 MHz, Chloroform-*d*) δ 7.65 (s, 1H, (CO)N*H*), 7.59 (d, *J* = 7.4 Hz, 1H, (CO)N*H*), 7.41 – 7.32 (m, 6H, Ar*H*), 7.23 (m, Ar*H*), 7.11 (m, 2H, Ar*H*), 5.62 (d, *J* = 7.4 Hz, 1H, NC*H*Ph), 5.33 (d, *J* = 12.2 Hz, 1H, O*CH₂*Ph), 5.28 (d, *J* = 12.2 Hz, 1H, O*CH₂*Ph), 3.83 (t, *J* = 6.9 Hz, 1H, C*H*), 3.09 – 2.98 (m, 2H, C*H₂*), 2.73 (d, *J* = 17.1 Hz, 1H, C*H₂*), 1.98 (d, *J* = 13.1 Hz, 1H, C*H₂*).
^{13}C NMR (101 MHz, Chloroform-*d*: the signal corresponding to one carbonyl C is not resolved) δ 174.1, 168.8, 137.9, 135.0, 128.8, 128.7, 128.7, 128.6, 127.9, 126.8, 68.2, 59.5, 57.6, 46.8, 43.7, 34.5.
HRMS (ESI/QTOF) m/z: $[M + H]^+$ Calcd for $C_{21}H_{21}N_2O_4^+$ 365.1496; Found 365.1492.

7. Determination of the absolute configuration of the enantioenriched cycloadduct by CEC

No single crystal suitable for crystallographic analysis could be obtained from enantioenriched **5a.a** or its derivatives. The absolute configuration of **5a.a** was therefore determined from corresponding enantioenriched azepane **10** following the Competing Enantioselective Conversion (CEC) method developed by Rychnovsky and co-workers for secondary cyclic amines.[55][56]

Preparation of pseudo-enantiomeric Bode's acylatin reagents

Synthesis of oxodiperoxymolybdenum(pyridine)-(hexamethylphosphoric triamide) (MoOPh, S95)

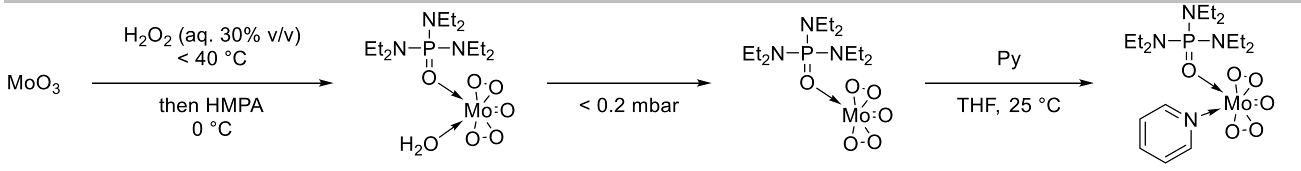

MoOPh, **S95**

Following a reported procedure,[57] in a 50 mL, round-bottomed, one-necked flask, MoO_3 (3.0 g, 21 mmol, 1.0 equiv.) was suspended in H_2O_2 (30% v/v; 15 mL). The off-white suspension was heated to 35-40 °C, checking its temperature by using a dipped-in termometer. According to the reported protocol, the internal temperature of the suspension was maintained below 40 °C during the first 30 minutes, during which the reaction is exothermal. Stirring was then ontinued at 40 °C for another 3.5 hours. After this time, the mixture looked like a yellow suspension, containing a small amount of a finely dispered whith solid. The latter was removed by filtration trhough a short pad of celite, giving a bright yellow, clear solution, which was cooled to 0 °C (ice - water bath). Addition of HMPA (3.7 mL, 21 mmol, 1.02 equiv.) over a period of 5 minutes, led to the precipitation of a crystalline solid. Stirring was continued at 0 °C for another 15 minutes. The solid was then collected on paper by suction filtration, maintained in the Büchner under suction for 10 minutes, and then transferred into a 50 mL single-necked flask with 2.0 mL MeOH. The suspension was heated to 40 °C, and further MeOH was added drop-wise under stirring until the complete dissolution of the solid. The bright yellow solution was then allowed to stand at a 4 °C (fridge) overnight, which led to the separation of $MoO_5.H_2O$.HMPA (3.57 g, 9.57 mmol, 46% yield) as large, bright yellow crystals. The latter were collected by filtration on paper, washed with ice-cold MeOH, and crashed to a fine powder. Drying over P_2O_5 under high vacuum (< 0.2 mbar) for 28 hours gave MoO_5.HMPA (3.30 g, 9.29 mmol, 45% yield relative to the initial amount of MoO_3) as a yellow powder. The latter was rapidly dissolved in dry THF (13.7 mL), and pyridine (dry; 0.75 mL, 9.3 mmol, 1.0 equiv. relative to MoO_5.HMPA) was added drop-wise. Massive precipitation was observed after the addition of ca. 75% of the mentioned amount of pyridine. After the complete addition of pyridine, the precipitate was collected by filtration on paper, washed with THF (dry; 2.0 mL), ether (dry; 25 mL), and dried under high vacuum for 4 hours (protected from light) to furnish oxodiperoxymolybdenum(pyridine)-(hexamethylphosphoric triamide) (MoOPH, **S95**) (3.26 g, 7.49 mmol, 36% yield relative to the initial amount of MoO_3) as a bright yellow solid, which was stored in a glove box at -20 °C, protected from light. No analysis was performed.

(4a*R*,9a*S*)-3-Oxo-2,3,9,9a-tetrahydroindeno[2,1-b][1,4]oxazin-4(4a*H*)-yl butyrate (acylating reagent (*R*,*S*)-C4, S96)

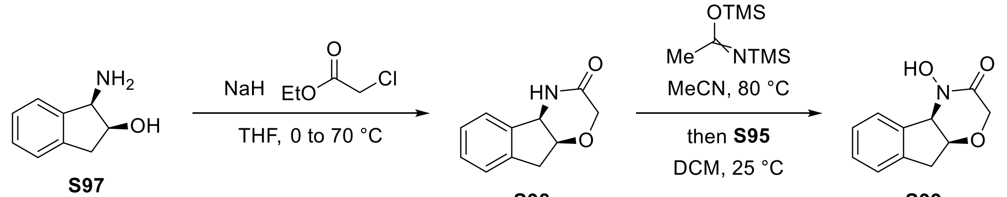

S97 **S98** **S99**

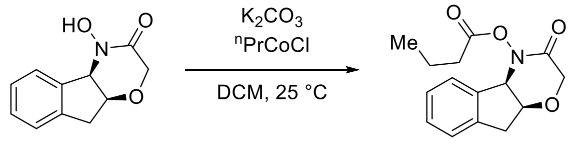

S99 **(*R*,*S*)-C4, S96**

Following a reported procedure,[58] in a 100 mL, two-necked, round-bottomed flask, equipped with an air condenser (Radley Findenser©), NaH (60% suspension in mineral oil, preliminarily washed twice with pentane; 0.21 g, 5.2 mmol, 1.3 equiv.) was suspended in THF (60 mL). The suspension was cooled to 0 °C (ice - water bath) prior to the addition of (1*R*, 2*S*)-(-)-cis-1-amino-2-indanol (**S98**) (0.60 g, 4.0 mmol, 1.0 equiv.) in two portions 15 minutes apart from each other. After the addition of the second portion, the mixture was heated to 70 °C for 40 minutes, turning from an off-white suspension into a purple-brown solution. The latter was cooled back to 0 °C, and ethyl chloroacetate (0.43 mL, 4.0 mmol, 1.0 equiv.) was added drop-wise over 10 minutes. The now darker solution was refluxed for 2 hours. It was then allowed to cool to room temperature, and washed twice with brine (2 x 30 mL). The combined aqueous layers were back-extracted with EtOAc (3 x 30 mL). The combined organic layers were vigorously stirred over $MgSO_4$ (ca. 5 g) overnight. Upon filtering and concentration under reduced pressure, pure (4a*R*,9a*S*)-4,4a,9,9a-tetrahydroindeno[2,1-b][1,4]oxazin-3(2H)-one (**S98**) (0.46 g, 2.4 mmol, 60% yield) was collected as a greysh solid.

^{1}H NMR (400 MHz, Acetone-d_6) δ 7.94 (br s, 1H, N*H*), 7.47 (m, 1H, Ar*H*), 7.33 – 7.16 (m, 3H, Ar*H*), 4.82 (m, 1H, ArC*H*N), 4.57 (t, *J* = 4.3 Hz, 1H, $ArCH_2CHO$), 4.05 (d, *J* = 16.2 Hz, 1H, (CO)CH_2O), 3.89 (dd, *J* = 16.2, 2.8 Hz, 1H, (CO)CH_2O), 3.23 (dd, *J* = 16.8, 4.7 Hz, 1H, ArCH_2CHO), 2.98 (d, *J* = 16.7 Hz, 1H, ArCH_2CHO).
^{1}H-NMR data corresponded to the reported values.[58]

Following a reported procedure,[59] in a 50 mL, single-necked, round-bottomed flask, (4a*R*,9a*S*)-4,4a,9,9a-tetrahydroindeno[2,1-b][1,4]oxazin-3(2H)-one (**S98**) (0.46 g, 2.4 mmol, 1.0 equiv.) was suspended in acetonitrile (4.6 mL). Trimethylsilyl N-(trimethylsilyl)acetimidate (0.65 mL) was added drop-wise to the suspension, which was consequently converted into a clear

yellow-grey solution. The latter was heated to 80 °C under stirring for 1 hour, becoming golden yellow. The mixture was then allowed to cool to room temperature, and the volatiles were removed under vacuum (Schlenk technique - rotary evaporation was avoided in order to avoid exposure to moisture). A solution of MoOPh (**S95**) (1.38 g, 3.16 mmol, 1.3 equiv.) in DCM (dry; 4.6 mL) was then added dorp-wise to the resulting crude oil. The bright golden mixture was then stirred at room temperature for 2 days and 8 hours, protected from light (aluminium foil). During this time, it became a brown suspension. Sat. aq. Na_4EDTA (9.2 mL) was then added, and the pH of the mixture was adjusted to 8 by drop-wise addition of aq. HCl (1.0 M). The aqueous layer was extracted with EtOAc (8 x 20 mL). All the organic layers were combined, washed with brine, dried over Na_2SO_4, filtered, and concentrated under vacuum in the presence of celite. The dark brown crude product, adsorbed on celite, was submitted to column chromatography (Biotage, 25 g SiO_2; EtOAc in pentane, 30 to 100%) to provide an orange solid. The latter was cristallized from EtOAc (25-30 mL) to give (4a*R*,9a*S*)-4-hydroxy-4,4a,9,9a-tetrahydroindeno[2,1-b][1,4]oxazin-3(2*H*)-one (*R*,*S* Bode's hydroxamic acid, **S99**; 0.19 g, 0.93 mmol, 38% yield) as an off-white solid.

^{1}H NMR (400 MHz, DMSO-*d*$_6$) δ 10.36 (m, 1H, N-O*H*), 7.63 (d, *J* = 7.1 Hz, 1H, Ar*H*), 7.32 – 7.17 (m, 3H, Ar*H*), 4.99 (d, *J* = 4.3 Hz, 1H, ArC*H*N), 4.66 (t, *J* = 4.6 Hz, 1H, ArCH$_2$C*H*O), 4.18 (d, *J* = 15.9 Hz, 1H, (CO)C*H$_2$*O), 4.04 (d, *J* = 15.9 Hz, 1H, (CO)C*H$_2$*O), 3.20 (dd, *J* = 16.9, 5.1 Hz, 1H, ArC*H$_2$*CHO), 2.90 (d, *J* = 16.9 Hz, 1H, ArC*H$_2$*CHO).
^{1}H-NMR data corresponded to the reported values.[59]

Following a reported procedure,[55] a 50 mL, round bottomed, single-necked flask was charged with K_2CO_3 (0.12 g, 0.88 mmol, 1.0 equiv.) and (4a*R*,9a*S*)-4-hydroxy-4,4a,9,9a-tetrahydroindeno[2,1-b][1,4]oxazin-3(2*H*)-one (**S99**) (0.18 g, 0.88 mmol, 1.0 equiv.). DCM (8.8 mL) was added. The resulting beige suspension was stirred at room temperature for 10 minutes; butiryl chloride was then added. The mixture was stirred at room temperature overnight. After 22 hours, the suspension looked slightly less turbid. The solids were removed by filtration though paper. The filtrate was concentrated under reduced pressure to give a yellow-orange crude oil. The latter was submitted to column chromatography (Büchi flash chromatographer; SiO_2, 12 g; EtOAc in pentane, 20 to 60%) to provide (4a*R*,9a*S*)-3-Oxo-2,3,9,9a-tetrahydroindeno[2,1-b][1,4]oxazin-4(4aH)-yl butyrate ((R,S)-C4, **S96**) (0.21 g, 0.76 mmol, 87% yield) as a pale yellow oil.

^{1}H NMR (400 MHz, Chloroform-*d*) δ 7.54 (d, *J* = 7.1 Hz, 1H, Ar*H*), 7.35 – 7.26 (m, 3H, Ar*H*), 5.04 (d, *J* = 4.4 Hz, 1H, ArC*H*N), 4.77 (m, 1H, ArCH$_2$C*H*O), 4.36 (d, *J* = 16.3 Hz, 1H, (CO)C*H$_2$*O), 4.30 (d, *J* = 16.3 Hz, 1H, (CO)C*H$_2$*O), 3.22 (dd, *J* = 16.9, 4.9 Hz, 1H, ArC*H$_2$*CHO), 3.13 (d, *J* = 16.8 Hz, 1H, ArC*H$_2$*CHO), 2.69 – 2.49 (m, 2H, C*H$_2$*CH$_2$CH$_3$), 1.83 (h, *J* = 7.4 Hz, 2H, CH$_2$C*H$_2$*CH$_3$), 1.06 (t, *J* = 7.4 Hz, 3H, CH$_2$CH$_2$C*H$_3$*).
^{1}H-NMR data corresponded to the reported values.[55]

(4a*S*,9a*R*)-3-oxo-2,3,9,9a-tetrahydroindeno[2,1-b][1,4]oxazin-4(4a*H*)-yl pentanoate (acylating reagent (*R*,*S*)-C5, S100)

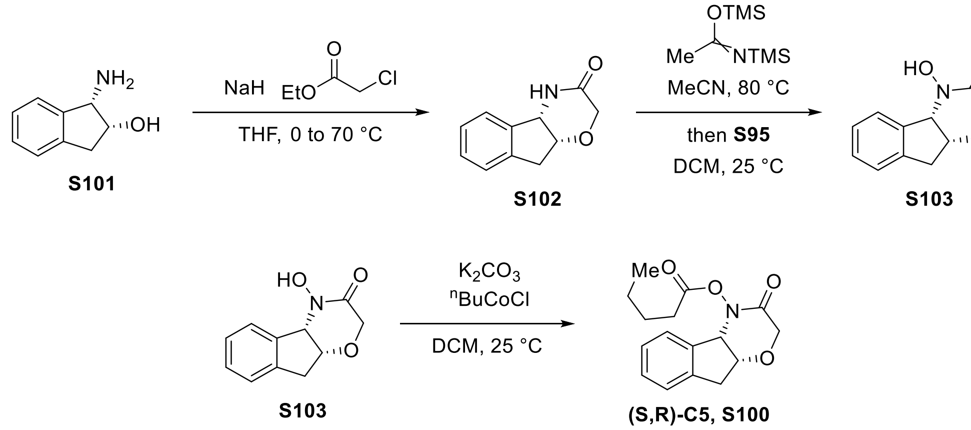

Following a reported procedure,[58] in a 100 mL, two-necked, round-bottomed flask, equipped with an air condenser (Radley Findenser©), NaH (60% suspension in mineral oil, preliminarily washed twice with pentane; 0.21 g, 5.2 mmol, 1.3 equiv.) was suspended in THF (60 mL). The suspension was cooled to 0 °C (ice - water bath) prior to the addition of (1*S*, 2*R*)-(-)-cis-1-amino-2-indanol (**S101**) (0.60 g, 4.0 mmol, 1.0 equiv.) in two portions 15 minutes apart from each other. After the addition of the second portion, the mixture was heated to 70 °C for 40 minutes, turning from an off-white suspension into a purple-brown solution. The latter was cooled back to 0 °C, and ethyl chloroacetate (0.43 mL, 4.0 mmol, 1.0 equiv.) was added drop-wise over 10 minutes. The now darker solution was refluxed for 2 hours. It was then allowed to cool to room temperature, and washed twice with brine (2 x 30 mL). The combined aqueous layers were back-extracted with EtOAc (3 x 30 mL). The combined organic layers were vigorously stirred over MgSO4 (ca. 5 g) overnight. Upon filtering and concentration under reduced pressure, pure (4a*S*,9a*R*)-4,4a,9,9a-tetrahydroindeno[2,1-b][1,4]oxazin-3(2H)-one (**S102**) (0.49 g, 2.6 mmol, 65% yield) was collected as a greysh solid.

Following a reported procedure,[59] in a 50 mL, single-necked, round-bottomed flask, (4a*S*,9a*R*)-4,4a,9,9a-tetrahydroindeno[2,1-b][1,4]oxazin-3(2H)-one (**S102**) (0.46 g, 2.4 mmol, 1.0 equiv.) was suspended in acetonitrile (4.9 mL). Trimethylsilyl N-(trimethylsilyl)acetimidate (0.70 mL) was added drop-wise to the suspension, which was consequently converted into a clear yellow-grey solution. The latter was heated to 80 °C under stirring for 1 hour, becoming golden yellow. The mixture was then allowed to cool to room temperature, and the volatiles were removed under vacuum (Schlenk technique - rotary evaporation was avoided in order to avoid exposure to moisture). A solution of MoOPh (**S95**) (1.46 g, 3.37 mmol, 1.3 equiv.) in DCM (dry; 4.9 mL) was then added dorp-wise to the resulting crude oil. The bright golden mixture was then stirred at room temperature for 2 days and 8 hours, protected from light (aluminium foil). During this time, it became a brown suspension. Sat. aq. Na_4EDTA (9.4 mL)

was then added, and the pH of the mixture was adjusted to 8 by drop-wise addition of aq. HCl (1.0 M). The aqueous layer was extracted with EtOAc (8 x 20 mL). All the organic layers were combined, washed with brine, dried over Na_2SO_4, filtered, and concentrated under vacuum in the presence of celite. The dark brown crude product, adsorbed on celite, was submitted to column chromatography (Biotage, 25 g SiO_2; EtOAc in pentane, 30 to 100%) to provide an orange solid. The latter was cristallized from EtOAc (25-30 mL) to give (4a*S*,9a*R*)-4-hydroxy-4,4a,9,9a-tetrahydroindeno[2,1-b][1,4]oxazin-3(2*H*)-one (*S*,*R* Bode's hydroxamic acid, **S103**; 0.21 g, 1.0 mmol, 39% yield) as an off-white solid.

Following a reported procedure,[55] a 50 mL, round bottomed, single-necked flask was charged with K_2CO_3 (0.14 g, 1.0 mmol, 1.0 equiv.) and (4a*S*,9a*R*)-4-hydroxy-4,4a,9,9a-tetrahydroindeno[2,1-b][1,4]oxazin-3(2*H*)-one (**S103**) (0.21 g, 1.0 mmol, 1.0 equiv.). DCM (10 mL) was added. The resulting beige suspension was stirred at room temperature for 10 minutes; butiryl chloride was then added. The mixture was stirred at room temperature overnight. After 22 hours, the suspension looked slightly less turbid. The solids were removed by filtration though paper. The filtrate was concentrated under reduced pressure to give a yellow-orange crude oil. The latter was submitted to column chromatography (Büchi flash chromatographer; SiO_2, 12 g; EtOAc in pentane, 20 to 60%) to provide (4a*S*,9a*R*)-3-oxo-2,3,9,9a-tetrahydroindeno[2,1-b][1,4]oxazin-4(4aH)-yl pentanoate ((*S*,*R*)-C5, **S100**) (0.27 g, 0.94 mmol, 94% yield) as a pale yellow oil.

^{1}H NMR (400 MHz, Chloroform-*d*) δ 7.54 (d, *J* = 7.2 Hz, 1H, Ar*H*), 7.37 – 7.28 (m, 3H, Ar*H*), 5.03 (d, *J* = 4.4 Hz, 1H, ArC*H*N), 4.78 (t, *J* = 4.6 Hz, 1H, ArCH$_2$C*H*O), 4.41 – 4.24 (m, 2H, (CO)C*H$_2$*O), 3.22 (dd, *J* = 16.9, 4.8 Hz, 1H, ArC*H$_2$*CHO), 3.13 (d, *J* = 16.8 Hz, 1H, ArC*H$_2$*CHO), 2.61 (h, *J* = 8.4 Hz, 2H, C*H$_2$*), 1.82 – 1.73 (m, 2H, C*H$_2$*), 1.46 (dq, *J* = 14.7, 7.4 Hz, 2H, C*H$_2$*), 0.96 (t, *J* = 7.4 Hz, 3H, C*H$_3$*).
^{1}H-NMR data corresponded to the reported values.[55]

CEC Experiment

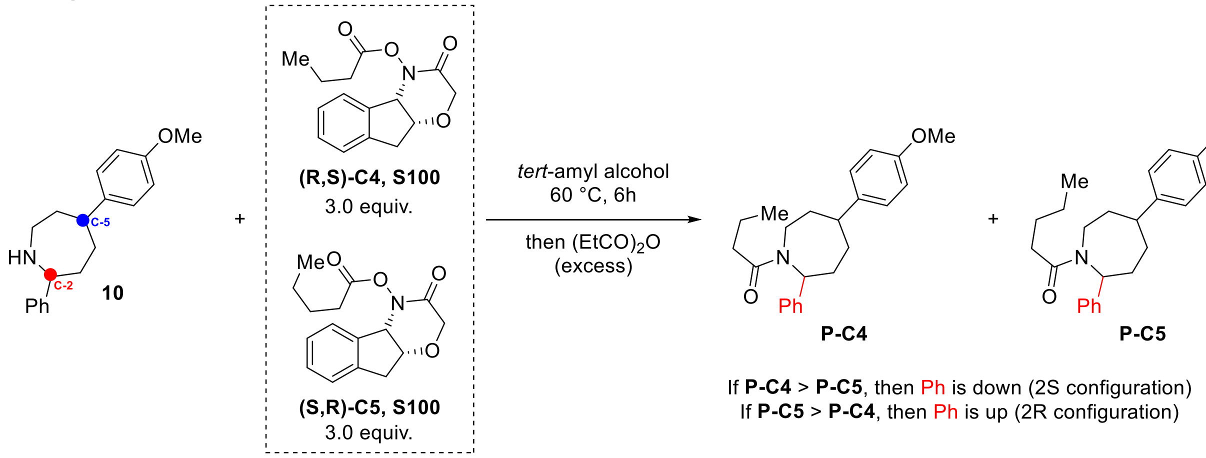

The experiment was executed following a slightly modified version (10 x scale-up; modified sampling of the acylating reagents) of a reported procedure.[56]
Cycloamine **10** (0.023 g, 8.2 mmol) was dissolved in MeOH (0.4 mL). tert-Amyl alcohol (7.8 mL) was then added, in order to obtain a 10 mM solution of **10** (solution **A**).
***(R,S)*-C4** (0.21 g) was dissolved in MeOH (0.21 g, 0.27 mL), in order to obtain a 50% w/w solution (solution **B-C4**).
***(S,R)*-C5** (0.27 g) was dissolved in MeOH (0.27 g, 0.35 mL), in order to obtain a 50% w/w solution (solution **B-C5**).
Three conic vials were charged each with:

- 16.5 mg of solution **B-C4** (corresponding to 8.2 mg of ***(R,S)*-C4**, 0.030 mmol, 3.0 equiv.);
- 17.4 mg of solution **B-C5** (corresponding to 8.7 mg of ***(S,R)*-C5**, 0.030 mmol, 3.0 equiv.);
- 1.0 mL of solution **A** (corresponding to 2.8 mg of **10**, 0.010 mmol, 1.0 equiv.).

The vials were sealed with PTFE caps, and delicately shaken in order to obtain completely homogeneous solutions (colorless) (experiments CEC-1 to CEC-3). The latter were then left to stand at 60 °C for 6 hours (protected from light with aluminium foil). After 6 hours, the solutions looked pale yellow. Propionic anhydride was added to the vials (0.13 mL, 1.0 mmol, 100 equiv. in each vial). The mixtures were left to stand for 15 minutes, before being submitted to UPLC-MS analysis.

For the UPLC-MS analysis, samples were prepared for injection from each of the aforementioned mixtures according to the following procedure: 10 µL of the mixture were diluted witn 990 µL of acidic MeOH (0.1% v/v formic acid in MeOH).

Samples were analyzed using Waters Acquity-I-UPLC Classsystem (Waters Corporation, Milford, MA, USA) coupled with a Waters Vion IMS-QTof Mass Spectrometer equipped with LockSpray. Analysis were performed on an ACQUITY UPLC® BEH C18 1.7µm column, 2.1 mm x 50 mm (Waters) heated at 30°C. The mobile phase was maintained at a flow rate of 0.4 mL/min and contained 0.1% (v/v) formic acid water solution (A), and 0.1% (v/v) formic acid acetonitrile solution (B). Over a 7 minutes total run, the gradient was: 0-0.5 min, 1% B; 0.5-5 min, 5-95% B; 5-6 min, 95% B; 6-6.1 min, 1% B and 6.1 to7 to re-equilibrate the system in initial conditions. The instrument was controlled by Waters UNIFI 1.9.4 (3.1.0, Waters Corporation, Milford, MA, USA). Injection volume was 5. The instrument was operated in positive polarity, sensitivity mode (33,000 FWHM at 556.2766 m/z). Data was acquired in HDMSe mode with a scan time of 0.036 s. The recorded mass range was from 50 to 1200 m/z for both low and high energy spectra. The collision energy was ramped from 20 to 40 V. The cone voltage was set to 30 V, capillary voltage was set to

2 kV and source offset was set to 50 V. Source temperature was set to 120 ◦C and desolvation temperature set to 500 ◦C. Cone gas flow rate was set to 50 L/h and desolvation gas flow rate was set to 1000 L/h.

Data concerning products **P-C4** and **PC5**:

P-C4 (1-(5-(4-Methoxyphenyl)-2-phenylazepan-1-yl)butan-1-one)
Retention time: 4.29 min.
CCS 192.20 A^2
10 ppm XIC
[M+H]$^+$: 352.2272, 0.7 ppm

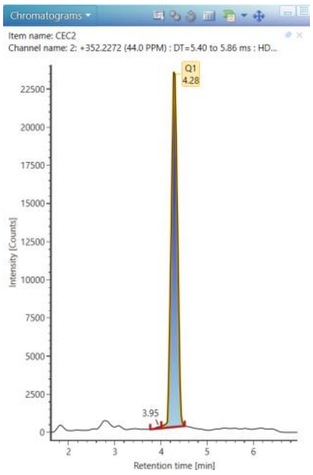

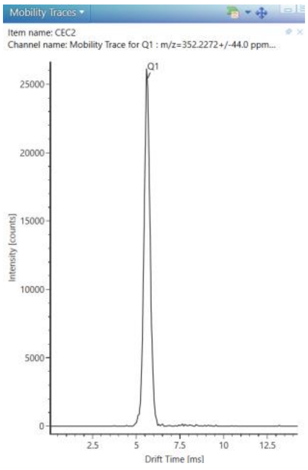

P-C5 (1-(5-(4-Methoxyphenyl)-2-phenylazepan-1-yl)pentan-1-one)
Retention time: 4.46 min.
CCS 303.04 A^2
10 ppm XIC
[M+H]$^+$: 366.2423, 1.1 ppm

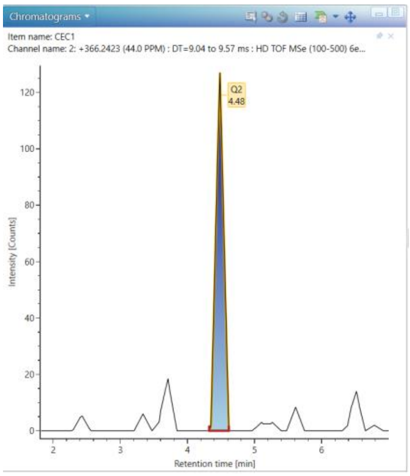

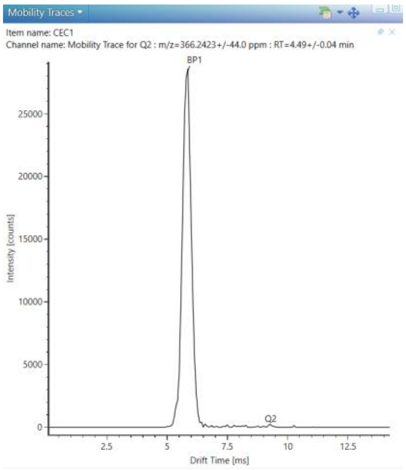

Quantification of P-C4 and P-C5:

Experiment	Integration residual **10**	Integration residual ***(R,S)*-C4**	Integration residual ***(S,R)*-C5**	Integration **P-C4**	Integration **P-C5**
CEC-1	324527.0	622.0	983.0	**4560.0**	**30084.0**
CEC-2	771360.7	1650.0	2791.0	**20251.3**	**136641.7**
CEC-3	898860.0	2115.3	3799.3	**28014.3**	**193166.7**

Values normalized to 100:

Experiment	Relative amount of **P-C4**	Relative amount of **P-C5**
CEC-1	13.16	86.84
CEC-2	12.91	87.09
CEC-3	12.67	87.33

Average percentual amounts:

P-C4: 12.9 ± 0.2%
P-C5: 87.1 ± 0.2%

Conclusions:
Formed amount of **P-C5** > Formed amount of **P-C4**
According to Rychnovsky and co-workers,[55][56] the Ph group on carbon C-2 in **10** is “up” in the major *trans* diastereoisomer obtained from **5a.a**. The aryl group on C-5 in 10 is consequently “down”. The absolute configuration is therefore determined as it follows:

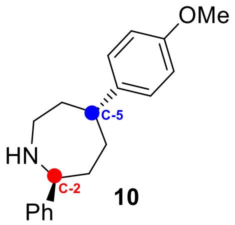

10

(2*S*,5*R*)-5-(4-methoxyphenyl)-2-phenylazepane

References

[1] G. Bold, A. Vaupel, M. Lang, **2008**, WO2008009487 (A1).
[2] L. Ghosez, P. Bayard, P. Nshimyumukiza, V. Gouverneur, F. Sainte, R. Beaudegnies, M. Rivera, A.-M. Frisque-Hesbain, C. Wynants, *Tetrahedron* **1995**, *51*, 11021–11042.
[3] E. W. Colvin, D. McGarry, M. J. Nugent, *Tetrahedron* **1988**, *44*, 4157–4172.
[4] Y. Watanabe, T. Washio, J. Krishnamurthi, M. Anada, S. Hashimoto, *Chem. Commun.* **2012**, *48*, 6969–6971.
[5] K. Yamamoto, M. Takemae, *Bull. Chem. Soc. Jpn.* **1989**, *62*, 2111–2113.
[6] S. Jayakumar, K. Louven, C. Strohmann, K. Kumar, *Angew. Chem. Int. Ed.* **2017**, *56*, 15945–15949.
[7] C. Gaul, J. T. Njardarson, D. Shan, D. C. Dorn, K.-D. Wu, W. P. Tong, X.-Y. Huang, M. A. S. Moore, S. J. Danishefsky, *J. Am. Chem. Soc.* **2004**, *126*, 11326–11337.
[8] S. A. Rogers, C. Melander, *Angew. Chem. Int. Ed.* **2008**, *47*, 5229–5231.
[9] Z.-J. Jia, S. Gao, F. H. Arnold, *J. Am. Chem. Soc.* **2020**, *142*, 10279–10283.
[10] Y.-Y. Zhou, L.-J. Wang, J. Li, X.-L. Sun, Y. Tang, *J. Am. Chem. Soc.* **2012**, *134*, 9066–9069.
[11] T. Nishikata, Y. Noda, R. Fujimoto, S. Ishikawa, *Chem. Commun.* **2015**, *51*, 12843–12846.
[12] S. Racine, B. Hegedüs, R. Scopelliti, J. Waser, *Chem. Eur. J.* **2016**, *22*, 11997–12001.
[13] H. Xu, J.-L. Hu, L. Wang, S. Liao, Y. Tang, *J. Am. Chem. Soc.* **2015**, *137*, 8006–8009.
[14] P. J. Black, G. Cami-Kobeci, M. G. Edwards, P. A. Slatford, M. K. Whittlesey, J. M. J. Williams, *Org. Biomol. Chem.* **2006**, *4*, 116–125.
[15] Y. Ogiwara, K. Takahashi, T. Kitazawa, N. Sakai, *J. Org. Chem.* **2015**, *80*, 3101–3110.
[16] H. Ohno, M. Yamamoto, M. Iuchi, N. Fujii, T. Tanaka, *Synthesis (Stuttg).* **2011**, 2567–2578.
[17] L. Meng, P. Wu, J. Fang, Y. Xiao, X. Xiao, G. Tu, X. Ma, S. Teng, J. Zeng, Q. Wan, *J. Am. Chem. Soc.* **2019**, *141*, 11775–11780.
[18] S. Racine, F. de Nanteuil, E. Serrano, J. Waser, *Angew. Chem. Int. Ed.* **2014**, *53*, 8484–8487.
[19] F. de Nanteuil, J. Waser, *Angew. Chem. Int. Ed.* **2011**, *50*, 12075–12079.
[20] S. Jaime-Figueroa, A. Zamilpa, A. Guzmán, D. J. Morgans, *Synth. Commun.* **2001**, *31*, 3739–3746.
[21] B. W. Turnpenny, S. R. Chemler, *Chem. Sci.* **2014**, *5*, 1786–1793.

[22] M. J. Hadd, M. D. Hocker, M. W. Holladay, G. Liu, M. W. Rowbottom, S. Xu, **2014**, EP2766359 (A2).

[23] S. M. Podhajsky, Y. Iwai, A. Cook-Sneathen, M. S. Sigman, *Tetrahedron* **2011**, *67*, 4435–4441.

[24] J.-S. Poh, S. Makai, T. von Keutz, D. N. Tran, C. Battilocchio, P. Pasau, S. V Ley, *Angew. Chem. Int. Ed.* **2017**, *56*, 1864–1868.

[25] Y. Tang, J.-L. Hu, Z. Xie, Q. Kang, Q. Liu, Y. Cheng, *No Title*, **2015**, CN104926747 (A).

[26] M.-C. Ye, B. Li, J. Zhou, X.-L. Sun, Y. Tang, *J. Org. Chem.* **2005**, *70*, 6108–6110.

[27] P. Wang, W.-J. Tao, X.-L. Sun, S. Liao, Y. Tang, *J. Am. Chem. Soc.* **2013**, *135*, 16849–16852.

[28] D. A. Alonso, S. K. Bertilsson, S. Y. Johnsson, S. J. M. Nordin, M. J. Södergren, P. G. Andersson, *J. Org. Chem.* **1999**, *64*, 2276–2280.

[29] J. Mao, F. Liu, M. Wang, L. Wu, B. Zheng, S. Liu, J. Zhong, Q. Bian, P. J. Walsh, *J. Am. Chem. Soc.* **2014**, *136*, 17662–17668.

[30] C. Foltz, M. Enders, S. Bellemin-Laponnaz, H. Wadepohl, L. H. Gade, *Chem. – A Eur. J.* **2007**, *13*, 5994–6008.

[31] R. Dave, N. A. Sasaki, *Tetrahedron: Asymmetry* **2006**, *17*, 388–401.

[32] M. G. Núñez, A. J. M. Farley, D. J. Dixon, *J. Am. Chem. Soc.* **2013**, *135*, 16348–16351.

[33] D. A. Klumpp, S. L. Aguirre, G. V Sanchez, S. J. de Leon, *Org. Lett.* **2001**, *3*, 2781–2784.

[34] M. Ju, C. D. Weatherly, I. A. Guzei, J. M. Schomaker, *Angew. Chem. Int. Ed.* **2017**, *56*, 9944–9948.

[35] S. Kanemasa, K. Adachi, H. Yamamoto, E. Wada, *Bull. Chem. Soc. Jpn.* **2000**, *73*, 681–687.

[36] C. N. Slattery, S. O'Keeffe, A. R. Maguire, *Tetrahedron: Asymmetry* **2013**, *24*, 1265–1275.

[37] P. Müller, C. Boléa, *Molecules* **2001**, *6*, 258–266.

[38] T. Sawada, M. Nakada, *Tetrahedron: Asymmetry* **2012**, *23*, 350–356.

[39] F. Liu, J. Zhong, Y. Zhou, Z. Gao, P. J. Walsh, X. Wang, S. Ma, S. Hou, S. Liu, M. Wang, et al., *Chem. – A Eur. J.* **2018**, *24*, 2059–2064.

[40] H. Zheng, M. P. Doyle, *Angew. Chem. Int. Ed.* **2019**, *58*, 12502–12506.

[41] S. K. Ginotra, V. K. Singh, *Org. Biomol. Chem.* **2007**, *5*, 3932–3937.

[42] S. Doherty, P. Goodrich, C. Hardacre, J. G. Knight, M. T. Nguyen, V. I. Pârvulescu, C. Paun, *Adv. Synth. Catal.* **2007**, *349*, 951–963.

[43] M. R. Castillo, S. Castillón, C. Claver, J. M. Fraile, A. Gual, M. Martín, J. A. Mayoral, E. Sola, *Tetrahedron* **2011**, *67*, 5402–5408.

[44] J. Zhou, Y. Tang, *J. Am. Chem. Soc.* **2002**, *124*, 9030–9031.

[45] J.-P. Qu, Z.-H. Xu, J. Zhou, C.-L. Cao, X.-L. Sun, L.-X. Dai, Y. Tang, *Adv. Synth. Catal.* **2009**, *351*, 308–312.

[46] X. Chang, Q. Zhang, C. Guo, *Org. Lett.* **2019**, *21*, 4915–4918.

[47] X. Chang, Q. Zhang, C. Guo, *Org. Lett.* **2019**, *21*, 4915–4918.

[48] Y. Sudo, D. Shirasaki, S. Harada, A. Nishida, *J. Am. Chem. Soc.* **2008**, *130*, 12588–12589.

[49] Q. Tan, H. Yu, Y. Luo, F. Chang, X. Liu, Y. Zhou, X. Feng, *Chem. Commun.* **2021**, *57*, 3018–3021.

[50] O. Toussaint, P. Capdevielle, M. Maumy, *Synthesis (Stuttg).* **1986**, *1986*, 1029–1031.

[51] M. Garreau, F. Le Vaillant, J. Waser, *Angew. Chem. Int. Ed.* **2019**, *58*, 8182–8186.

[52] T. M. Pimpalpalle, J. Yin, T. Linker, *Org. Biomol. Chem.* **2012**, *10*, 103–109.

[53] A. Deiters, M. Pettersson, S. F. Martin, *J. Org. Chem.* **2006**, *71*, 6547–6561.

[54] L. Nicke, P. Horx, K. Harms, A. Geyer, *Chem. Sci.* **2019**, *10*, 8634–8641.

[55] A. Burtea, S. D. Rychnovsky, *Org. Lett.* **2017**, *19*, 4195–4198.

[56] C. J. Dooley, A. Burtea, C. Mitilian, W. T. Dao, B. Qu, N. T. Salzameda, S. D. Rychnovsky, *J. Org. Chem.* **2020**, *85*, 10750–10759.

[57] E. Vedejs, S. Larsen, *Org. Synth.* **1986**, *64*, 127.

[58] H. U. Vora, S. P. Lathrop, N. T. Reynolds, M. S. Kerr, J. Read de Alaniz, T. Rovis, *Org. Synth.* **2010**, *87*, 350.

[59] M. Binanzer, S.-Y. Hsieh, J. W. Bode, *J. Am. Chem. Soc.* **2011**, *133*, 19698–19701.

[60] G. Bentzinger, W. De Souza, C. Mullié, P. Agnamey, A. Dassonville-Klimpt, P. Sonnet, *Tetrahedron: Asymmetry* **2016**, *27*, 1–11.

Crystal Data and Experimental for 5a.a

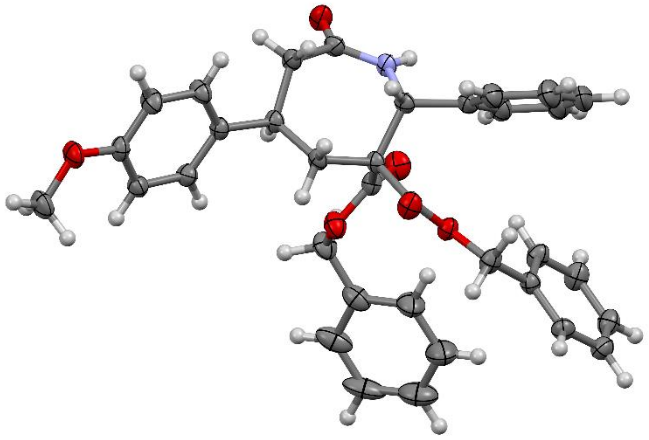

Recrystallization of 5a.a: 5a.a (ca. 0.11 g) were suspended in n-hexane (ca. 2.0 mL) and the mixture was heated to 70 °C. EtOAc was added drop-wise until a clean solution was formed. The latter was allowed to cool to room temperature over several hours.

Experimental. Single clear pale colourless irregular crystals of **5a.a** were used as supplied. A suitable crystal with dimensions 0.52 × 0.39 × 0.23 mm^3 was selected and mounted on a SuperNova, Dual, Cu at home/near, AtlasS2 diffractometer. The crystal was kept at a steady T = 140.00(10) K during data collection. The structure was solved with the ShelXT (Sheldrick, 2015) solution program using dual methods and by using Olex2 (Dolomanov et al., 2009) as the graphical interface. The model was refined with ShelXL 2018/3 (Sheldrick, 2015) using full matrix least squares minimisation on $\boldsymbol{F^2}$.

Crystal Data. $C_{35}H_{33}NO_6$, M_r = 563.62, triclinic, P-1 (No. 2), a = 10.2442(3) Å, b = 12.3459(3) Å, c = 12.6199(3) Å, α = 96.478(2)°, β = 109.519(3)°, γ = 103.169(2)°, V = 1433.43(7) $Å^3$, T = 140.00(10) K, Z = 2, Z' = 1, μ(Cu K_α) = 0.721, 15213 reflections measured, 5924 unique (R_{int} = 0.0117) which were used in all calculations. The final wR_2 was 0.1050 (all data) and R_1 was 0.0403 (I≥2 σ(I)).

**Compound**	**5a.a**
Formula	$C_{35}H_{33}NO_6$
$D_{calc.}$/ g cm^{-3}	1.306
μ/mm^{-1}	0.721
Formula Weight	563.62
Colour	clear pale colourless
Shape	irregular
Size/mm^3	0.52×0.39×0.23
T/K	140.00(10)
Crystal System	triclinic
Space Group	P-1
a/Å	10.2442(3)
b/Å	12.3459(3)
c/Å	12.6199(3)
α/°	96.478(2)
β/°	109.519(3)
γ/°	103.169(2)
V/$Å^3$	1433.43(7)
Z	2
Z'	1
Wavelength/Å	1.54184
Radiation type	Cu K□
Θ_{min}/°	3.757
Θ_{max}/°	75.914
Measured Refl's.	15213
Indep't Refl's	5924
Refl's I≥2 σ(I)	5708
R_{int}	0.0117
Parameters	548
Restraints	224
Largest Peak	0.366
Deepest Hole	-0.206
GooF	1.043
wR_2 (all data)	0.1050
wR_2	0.1041
R_1 (all data)	0.0415
R_1	0.0403

Structure Quality Indicators

**Reflections:**	d min (Cu) CIF 0.79	I/σ(I) CIF 80.6	Rint CIF 1.17%	complete 100%
**Refinement:**	Shift CIF 0.001	Max Peak CIF 0.4	Min Peak CIF -0.2	GooF CIF 1.043

A clear pale colourless irregular-shaped crystal with dimensions 0.52 × 0.39 × 0.23 mm^3 was mounted. Data were collected using a SuperNova, Dual, Cu at home/near, AtlasS2 diffractometer operating at T = 140.00(10) K.

Data were measured using ω scans using Cu K_α radiation. The diffraction pattern was indexed and the total number of runs and images was based on the strategy calculation from the program CrysAlisPro (Rigaku, V1.171.40.84a, 2020). The maximum resolution that was achieved was Θ = 75.914° (0.79 Å).

The unit cell was refined using CrysAlisPro (Rigaku, V1.171.40.84a, 2020) on 11170 reflections, 73% of the observed reflections.

Data reduction, scaling and absorption corrections were performed using CrysAlisPro (Rigaku, V1.171.40.84a, 2020). The final completeness is 99.90 % out to 75.914° in Θ. An analytical absorption correction was performed using CrysAlisPro 1.171.40.84a (Rigaku Oxford Diffraction, 2020). The analytical numeric absorption correction was done using a multifaceted crystal model based on expressions derived by R.C. Clark & J.S. Reid (Clark, R. C. & Reid, J. S. (1995). Acta Cryst. A51, 887-897). The empirical absorption correction was carried out using spherical harmonics, implemented in SCALE3 ABSPACK scaling algorithm. The absorption coefficient μ of this crystal is 0.721 mm^{-1} at this wavelength (λ = 1.54184Å) and the minimum and maximum transmissions are 0.785 and 0.885.

The structure was solved and the space group *P*-1 (# 2) determined by the ShelXT (Sheldrick, 2015) structure solution program using dual methods and refined by full matrix least squares minimisation on F^2 using version 2018/3 of ShelXL (Sheldrick, 2015). All non-hydrogen atoms were refined anisotropically. Most hydrogen atom positions were calculated geometrically and refined using the riding model, but some hydrogen atoms were refined freely.

There is a single molecule in the asymmetric unit, which is represented by the reported sum formula. In other words: Z is 2 and Z' is 1.

Data Plots: Diffraction Data

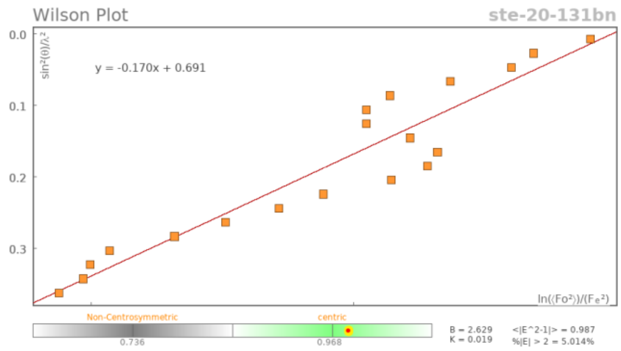

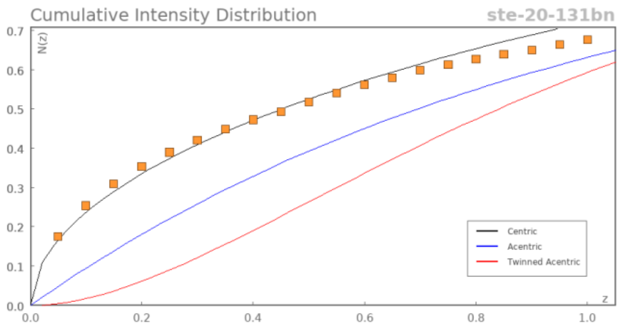

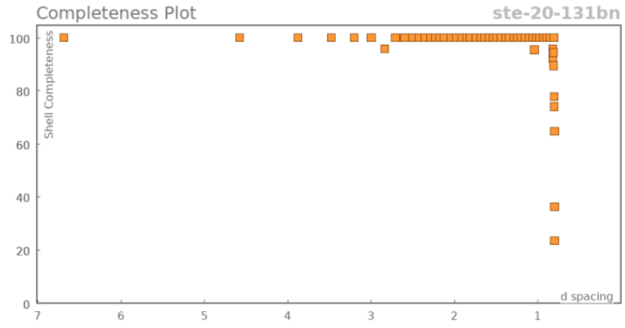

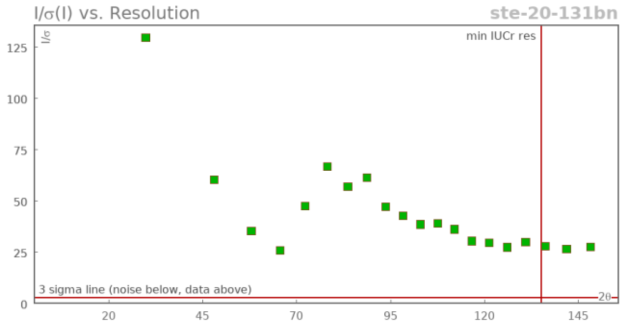

Data Plots: Refinement and Data

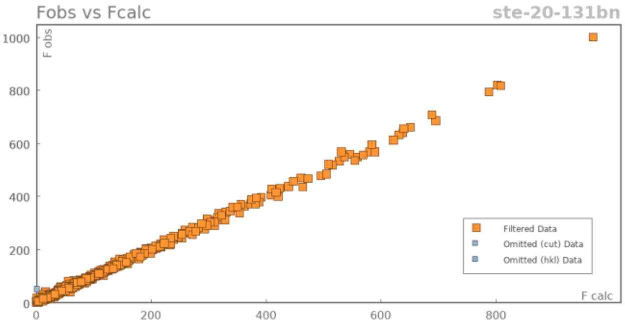

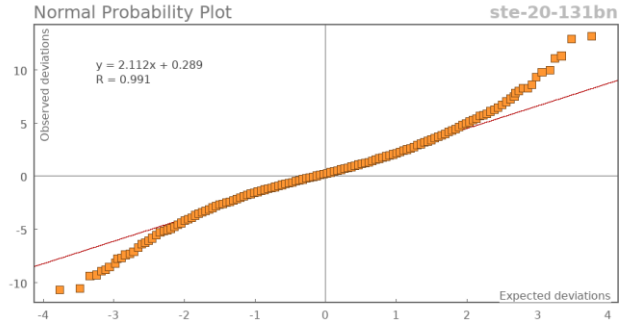

Reflection Statistics

Total reflections (after filtering)	15214	Unique reflections	5924
Completeness	0.99	Mean I/□	44.31
hkl_{max} collected	(12, 15, 15)	hkl_{min} collected	(-12, -10, -15)
hkl_{max} used	(11, 15, 15)	hkl_{min} used	(-12, -15, 0)
Lim d_{max} collected	100.0	Lim d_{min} collected	0.77
d_{max} used	11.76	d_{min} used	0.79
Friedel pairs	1311	Friedel pairs merged	1
Inconsistent equivalents	11	R_{int}	0.0117
R_{sigma}	0.0124	Intensity transformed	0
Omitted reflections	0	Omitted by user (OMIT hkl)	1
Multiplicity	(3265, 1905, 1086, 535, 208, 101, 52, 44, 27, 9, 2, 2)	Maximum multiplicity	12
Removed systematic absences	0	Filtered off (Shel/OMIT)	0

Table 1: Fractional Atomic Coordinates (×10^4) and Equivalent Isotropic Displacement Parameters (Å^2×10^3) for **5a.a**. U_{eq} is defined as 1/3 of the trace of the orthogonalised U_{ij}.

Atom	x	y	z	U_{eq}
O1	5016.2(10)	10782.5(8)	8898.8(8)	36.7(2)
O2	1553.6(11)	10529.2(9)	1779.0(8)	38.6(2)
O3	-43.5(10)	6191.8(8)	5970.7(8)	35.3(2)
O4	1695.4(9)	5577.3(7)	7143.1(7)	28.48(19)
O5	4633.0(10)	7437.2(9)	8358.9(8)	40.4(2)
O6	4144.5(11)	6974.5(9)	6467.3(9)	38.3(2)
N1	3326.3(11)	9163.9(9)	8676.6(9)	29.2(2)
C1	2336.3(12)	7503.2(10)	6988.8(9)	24.8(2)
C2	2071.9(13)	8093.3(10)	5950.1(10)	26.1(2)
C3	3114.4(13)	9276.1(10)	6139.2(10)	26.1(2)
C4	3070.7(14)	10162.7(10)	7079.5(10)	29.3(3)
C5	3878.7(13)	10057.1(10)	8290.0(10)	28.6(2)
C6	2075.9(13)	8213.7(10)	7986.7(10)	26.4(2)
C7	2750.3(12)	9655.1(10)	4994.1(10)	25.9(2)
C8	1919.1(14)	10403.8(12)	4710.6(11)	32.7(3)

Atom	x	y	z	U_{eq}
C9	1562.0(15)	10686.5(12)	3639.8(12)	35.5(3)
C10	2005.7(13)	10213.8(11)	2813.7(10)	29.7(3)
C11	2849.6(14)	9477.0(11)	3080.3(11)	30.4(3)
C12	3212.8(14)	9214.6(11)	4168.0(11)	29.7(3)
C13	1892.0(19)	10001.2(15)	877.2(12)	44.3(3)
C14	1531.8(14)	7534.6(10)	8761.8(10)	30.1(3)
C15	65.8(17)	7289.9(13)	8573.8(14)	40.9(3)
C16	-487(2)	6707.8(15)	9280.8(17)	54.0(4)
C17	419(2)	6387.6(14)	10183.4(16)	55.8(5)
C18	1876(2)	6617.1(13)	10373.9(13)	49.7(4)
C19	2439.6(17)	7185.9(11)	9661.8(11)	36.6(3)
C20	1181.0(13)	6351.8(10)	6616.4(9)	26.0(2)
C21	655.6(14)	4503.9(11)	7007.8(12)	33.5(3)
C22	1413.7(13)	3807.3(10)	7772.2(10)	28.6(2)
C23	2648.9(14)	4307.9(11)	8754.7(12)	33.7(3)
C24	3290.7(15)	3638.3(12)	9459.6(12)	37.8(3)
C25	2697.8(15)	2469.4(12)	9207.0(13)	36.5(3)
C26	1447.8(15)	1971.2(11)	8250.8(12)	35.2(3)
C27	817.3(14)	2631.7(11)	7527.9(11)	32.6(3)
C28	3835.3(13)	7309.5(10)	7381.5(11)	28.9(3)
C29	5614(3)	6855(3)	6755(3)	39.4(7)
C30	5607(5)	5639(4)	6698(5)	41.5(9)
C31	4720(3)	4909(2)	7102(3)	49.9(7)
C32	4732(5)	3781(3)	7085(3)	61.4(8)
C33	5664(5)	3377(5)	6682(5)	63.5(14)
C34	6528(5)	4085(4)	6263(3)	63.1(11)
C35	6517(5)	5219(4)	6294(3)	52.3(10)
C36	5369(13)	6584(10)	6441(10)	44(2)
C37	5357(18)	5406(12)	6589(17)	44(2)
C38	6597(16)	5019(13)	6566(12)	57(3)
C39	6543(15)	3942(11)	6647(10)	53(2)
C40	5277(15)	3185(14)	6616(15)	50(2)
C41	4115(11)	3486(8)	6564(10)	55.0(16)
C42	4116(11)	4595(8)	6476(11)	64.1(18)

Table 2: Anisotropic Displacement Parameters (×10[4]) for **5a.a**. The anisotropic displacement factor exponent takes the form: $-2\pi^2[h^2a^{*2} \times U_{11} + ... + 2hka^* \times b^* \times U_{12}]$

Atom	U_{11}	U_{22}	U_{33}	U_{23}	U_{13}	U_{12}
O1	37.9(5)	33.1(5)	24.3(4)	4.6(3)	1.3(4)	-1.8(4)
O2	48.0(6)	47.7(6)	25.5(4)	17.1(4)	13.4(4)	19.6(4)
O3	27.9(4)	37.0(5)	30.6(5)	8.6(4)	0.8(4)	3.8(4)
O4	26.4(4)	27.2(4)	27.7(4)	7.5(3)	6.5(3)	4.4(3)
O5	30.2(5)	51.7(6)	33.6(5)	11.7(4)	2.2(4)	13.9(4)
O6	38.7(5)	49.2(6)	40.0(5)	19.0(4)	22.0(4)	21.1(4)
N1	31.3(5)	30.3(5)	17.8(5)	4.6(4)	2.7(4)	3.2(4)
C1	24.0(5)	28.3(6)	19.0(5)	5.7(4)	5.0(4)	6.0(4)
C2	27.7(6)	29.0(6)	19.1(5)	6.2(4)	5.5(4)	7.8(5)
C3	25.6(6)	29.5(6)	20.8(5)	7.1(4)	5.3(4)	7.4(4)
C4	34.5(6)	27.1(6)	21.4(6)	5.7(4)	5.4(5)	6.8(5)
C5	31.8(6)	28.6(6)	20.9(5)	3.4(4)	5.7(5)	7.2(5)
C6	26.6(6)	28.7(6)	19.9(5)	4.0(4)	5.5(4)	5.5(4)
C7	23.8(5)	28.3(6)	22.3(5)	7.0(4)	5.5(4)	4.8(4)
C8	34.5(6)	42.7(7)	26.2(6)	10.9(5)	12.2(5)	17.7(5)
C9	38.6(7)	44.7(7)	30.7(6)	16.3(5)	12.7(5)	21.8(6)
C10	29.1(6)	33.8(6)	24.1(6)	10.8(5)	7.7(5)	5.9(5)
C11	33.1(6)	32.9(6)	26.2(6)	6.9(5)	12.2(5)	8.8(5)
C12	31.1(6)	31.0(6)	27.5(6)	8.4(5)	9.3(5)	11.4(5)
C13	54.9(9)	56.8(9)	25.6(7)	14.5(6)	16.4(6)	18.8(7)
C14	37.0(6)	28.1(6)	23.3(6)	2.0(4)	12.7(5)	4.6(5)
C15	42.1(8)	39.7(7)	45.0(8)	5.3(6)	24.8(7)	7.7(6)
C16	60.8(10)	49.7(9)	63.6(11)	10.2(8)	43.6(9)	8.0(8)
C17	86.2(13)	40.3(8)	50.2(9)	7.4(7)	47.9(10)	2.5(8)
C18	79.5(12)	36.1(7)	29.3(7)	8.5(6)	20.5(8)	7.2(7)
C19	47.5(8)	31.4(6)	24.8(6)	5.3(5)	10.7(6)	4.1(6)
C20	27.3(6)	29.3(6)	19.4(5)	4.1(4)	7.5(4)	6.4(4)

Atom	U_{11}	U_{22}	U_{33}	U_{23}	U_{13}	U_{12}
C21	29.0(6)	30.4(6)	31.2(6)	8.1(5)	3.5(5)	0.4(5)
C22	27.0(6)	30.3(6)	26.8(6)	6.5(5)	10.0(5)	4.9(5)
C23	29.3(6)	30.1(6)	34.1(7)	7.0(5)	6.3(5)	2.2(5)
C24	28.7(6)	39.7(7)	36.9(7)	10.9(6)	4.7(5)	4.5(5)
C25	34.3(7)	37.4(7)	40.1(7)	15.5(6)	13.0(6)	12.3(5)
C26	37.8(7)	27.8(6)	39.5(7)	6.3(5)	15.5(6)	6.8(5)
C27	31.2(6)	30.7(6)	29.3(6)	2.2(5)	8.1(5)	3.1(5)
C28	26.9(6)	30.6(6)	29.1(6)	11.0(5)	9.4(5)	6.9(5)
C29	31.3(13)	51.8(18)	44.8(18)	14.1(12)	21.8(12)	16.5(12)
C30	34.0(17)	58(2)	33.3(13)	4.8(14)	11.3(12)	18.6(14)
C31	60.8(16)	50.0(13)	57.3(16)	14.3(12)	34.1(13)	30.6(12)
C32	82(2)	51.6(16)	67.6(19)	14.6(13)	38.2(16)	35.2(15)
C33	62(3)	62(3)	66.6(17)	-5.2(19)	18(2)	35(2)
C34	48.9(14)	76(2)	56(2)	-21.0(18)	9.0(16)	35.7(15)
C35	33.5(12)	74(2)	42.1(17)	-10.4(15)	7.4(13)	22.0(13)
C36	46(4)	52(4)	42(4)	10(3)	22(3)	17(3)
C37	45(4)	46(3)	49(4)	17(3)	19(3)	21(3)
C38	37(3)	53(4)	60(4)	11(3)	-1(3)	3(3)
C39	39(3)	51(3)	63(4)	12(3)	11(4)	13(3)
C40	58(4)	45(3)	62(4)	19(3)	38(3)	14(3)
C41	60(3)	47(3)	77(4)	15(3)	47(3)	18(3)
C42	60(3)	60(3)	81(4)	12(3)	39(3)	16(3)

Table 3: Bond Lengths in Å for **5a.a**.

Atom	Atom	Length/Å
O1	C5	1.2334(15)
O2	C10	1.3653(15)
O2	C13	1.4281(18)
O3	C20	1.2018(15)
O4	C20	1.3315(15)
O4	C21	1.4506(14)
O5	C28	1.1991(16)
O6	C28	1.3413(16)
O6	C29	1.472(3)
O6	C36	1.452(11)
N1	C5	1.3401(16)
N1	C6	1.4541(15)
C1	C2	1.5462(15)
C1	C6	1.5839(16)
C1	C20	1.5340(16)
C1	C28	1.5318(16)
C2	C3	1.5396(16)
C3	C4	1.5405(17)
C3	C7	1.5225(15)
C4	C5	1.5116(16)
C6	C14	1.5188(17)
C7	C8	1.3918(17)
C7	C12	1.3881(17)
C8	C9	1.3843(18)
C9	C10	1.3907(19)
C10	C11	1.3859(18)
C11	C12	1.3935(17)
C14	C15	1.394(2)
C14	C19	1.3882(19)
C15	C16	1.389(2)
C16	C17	1.370(3)
C17	C18	1.385(3)
C18	C19	1.392(2)
C21	C22	1.5047(17)
C22	C23	1.3939(18)
C22	C27	1.3943(17)
C23	C24	1.3891(19)
C24	C25	1.386(2)
C25	C26	1.384(2)
C26	C27	1.3886(19)
C29	C30	1.494(5)
C30	C31	1.392(6)
C30	C35	1.369(7)
C31	C32	1.394(4)
C32	C33	1.380(6)
C33	C34	1.375(7)
C34	C35	1.399(7)
C36	C37	1.484(13)
C37	C38	1.46(3)
C37	C42	1.381(18)
C38	C39	1.335(18)
C39	C40	1.401(15)
C40	C41	1.310(19)
C41	C42	1.385(12)

Table 4: Bond Angles in ° for **5a.a**.

Atom	Atom	Atom	Angle/°
C10	O2	C13	117.53(11)
C20	O4	C21	116.68(9)
C28	O6	C29	114.23(18)
C28	O6	C36	128.5(5)
C5	N1	C6	125.12(10)
C2	C1	C6	109.50(9)
C20	C1	C2	107.74(9)
C20	C1	C6	105.97(9)
C28	C1	C2	111.50(10)
C28	C1	C6	112.63(9)
C28	C1	C20	109.23(9)
C3	C2	C1	116.45(9)

Atom	Atom	Atom	Angle/˚
C2	C3	C4	113.28(10)
C7	C3	C2	107.91(9)
C7	C3	C4	111.15(10)
C5	C4	C3	114.04(10)
O1	C5	N1	121.45(11)
O1	C5	C4	120.17(11)
N1	C5	C4	118.38(11)
N1	C6	C1	112.59(10)
N1	C6	C14	110.01(10)
C14	C6	C1	115.87(10)
C8	C7	C3	122.88(11)
C12	C7	C3	119.60(11)
C12	C7	C8	117.49(11)
C9	C8	C7	120.89(12)
C8	C9	C10	120.73(12)
O2	C10	C9	115.53(11)
O2	C10	C11	125.02(12)
C11	C10	C9	119.45(11)
C10	C11	C12	118.92(12)
C7	C12	C11	122.50(11)
C15	C14	C6	117.91(12)
C19	C14	C6	122.70(12)
C19	C14	C15	119.36(13)
C16	C15	C14	120.59(16)
C17	C16	C15	119.80(17)
C16	C17	C18	120.17(15)
C17	C18	C19	120.55(16)
C14	C19	C18	119.51(15)
O3	C20	O4	124.84(11)
O3	C20	C1	123.92(11)
O4	C20	C1	111.17(9)
O4	C21	C22	108.40(10)
C23	C22	C21	121.93(11)
C23	C22	C27	118.92(12)
C27	C22	C21	119.04(11)
C24	C23	C22	120.23(12)
C25	C24	C23	120.51(13)
C26	C25	C24	119.49(13)
C25	C26	C27	120.34(12)
C26	C27	C22	120.46(12)
O5	C28	O6	124.08(12)
O5	C28	C1	125.79(12)
O6	C28	C1	110.13(10)
O6	C29	C30	111.3(3)
C31	C30	C29	120.9(4)
C35	C30	C29	121.1(4)
C35	C30	C31	118.0(4)
C30	C31	C32	121.6(3)
C33	C32	C31	119.6(4)
C34	C33	C32	119.3(5)
C33	C34	C35	120.6(4)
C30	C35	C34	120.9(4)
O6	C36	C37	118.3(11)
C38	C37	C36	118.0(13)
C42	C37	C36	122.4(13)
C42	C37	C38	117.8(12)
C39	C38	C37	117.4(13)
C38	C39	C40	120.7(15)
C41	C40	C39	123.2(15)
C40	C41	C42	118.1(9)
C37	C42	C41	121.5(10)

Table 5: Torsion Angles in ˚ for **5a.a**.

Atom	Atom	Atom	Atom	Angle/˚
O2	C10	C11	C12	-178.96(12)
O4	C21	C22	C23	25.20(17)
O4	C21	C22	C27	-158.54(11)
O6	C29	C30	C31	-40.1(5)
O6	C29	C30	C35	142.8(4)
O6	C36	C37	C38	-179.7(13)
O6	C36	C37	C42	16(2)
N1	C6	C14	C15	129.53(12)
N1	C6	C14	C19	-48.35(16)
C1	C2	C3	C4	-61.38(13)
C1	C2	C3	C7	175.12(10)
C1	C6	C14	C15	-101.34(13)
C1	C6	C14	C19	80.78(14)
C2	C1	C6	N1	-82.47(12)
C2	C1	C6	C14	149.67(10)
C2	C1	C20	O3	-35.85(15)
C2	C1	C20	O4	147.13(10)
C2	C1	C28	O5	139.27(13)
C2	C1	C28	O6	-40.54(13)
C2	C3	C4	C5	77.59(13)
C2	C3	C7	C8	99.59(13)
C2	C3	C7	C12	-78.04(13)
C3	C4	C5	O1	109.63(13)
C3	C4	C5	N1	-70.50(15)
C3	C7	C8	C9	-177.16(12)
C3	C7	C12	C11	176.38(11)
C4	C3	C7	C8	-25.19(16)
C4	C3	C7	C12	157.18(11)
C5	N1	C6	C1	63.97(15)
C5	N1	C6	C14	-165.15(12)
C6	N1	C5	O1	-173.56(12)
C6	N1	C5	C4	6.58(18)

Atom	Atom	Atom	Atom	Angle/˚
C6	C1	C2	C3	64.83(13)
C6	C1	C20	O3	81.29(14)
C6	C1	C20	O4	-95.73(11)
C6	C1	C28	O5	15.69(17)
C6	C1	C28	O6	-164.12(10)
C6	C14	C15	C16	-177.64(13)
C6	C14	C19	C18	176.66(12)
C7	C3	C4	C5	-160.71(10)
C7	C8	C9	C10	1.1(2)
C8	C7	C12	C11	-1.38(19)
C8	C9	C10	O2	178.12(13)
C8	C9	C10	C11	-1.9(2)
C9	C10	C11	C12	1.02(19)
C10	C11	C12	C7	0.61(19)
C12	C7	C8	C9	0.53(19)
C13	O2	C10	C9	-175.47(13)
C13	O2	C10	C11	4.51(19)
C14	C15	C16	C17	1.1(2)
C15	C14	C19	C18	-1.2(2)
C15	C16	C17	C18	-1.7(3)
C16	C17	C18	C19	0.8(2)
C17	C18	C19	C14	0.7(2)
C19	C14	C15	C16	0.3(2)
C20	O4	C21	C22	-173.43(10)
C20	C1	C2	C3	179.64(10)
C20	C1	C6	N1	161.58(10)
C20	C1	C6	C14	33.72(13)
C20	C1	C28	O5	-101.77(14)
C20	C1	C28	O6	78.42(12)
C21	O4	C20	O3	-6.41(17)
C21	O4	C20	C1	170.58(10)
C21	C22	C23	C24	177.86(13)
C21	C22	C27	C26	-176.55(12)
C22	C23	C24	C25	-1.2(2)
C23	C22	C27	C26	-0.18(19)
C23	C24	C25	C26	-0.7(2)
C24	C25	C26	C27	2.1(2)
C25	C26	C27	C22	-1.7(2)
C27	C22	C23	C24	1.6(2)
C28	O6	C29	C30	100.8(3)
C28	O6	C36	C37	77.1(11)
C28	C1	C2	C3	-60.51(13)
C28	C1	C6	N1	42.21(13)
C28	C1	C6	C14	-85.65(12)
C28	C1	C20	O3	-157.12(12)
C28	C1	C20	O4	25.85(13)
C29	O6	C28	O5	-4.5(2)
C29	O6	C28	C1	175.30(19)
C29	C30	C31	C32	-178.1(3)
C29	C30	C35	C34	178.7(3)
C30	C31	C32	C33	1.4(6)
C31	C30	C35	C34	1.5(6)
C31	C32	C33	C34	-2.4(6)
C32	C33	C34	C35	3.0(7)
C33	C34	C35	C30	-2.6(6)
C35	C30	C31	C32	-0.9(6)
C36	O6	C28	O5	7.5(7)
C36	O6	C28	C1	-172.7(6)
C36	C37	C38	C39	-177.0(12)
C36	C37	C42	C41	176.9(12)
C37	C38	C39	C40	7(2)
C38	C37	C42	C41	12(2)
C38	C39	C40	C41	-3(2)
C39	C40	C41	C42	3(2)
C40	C41	C42	C37	-8.3(19)
C42	C37	C38	C39	-12(2)

Table 6: Hydrogen Fractional Atomic Coordinates (×10^4) and Equivalent Isotropic Displacement Parameters (Å^2×10^3) for **5a.a**. U_{eq} is defined as 1/3 of the trace of the orthogonalised U_{ij}.

Atom	x	y	z	U_{eq}
H29A	6230.63	7298.23	7539.28	47
H29B	6030.12	7167.96	6212.5	47
H31	4091.49	5187.6	7396.59	60
H32	4101.74	3292.73	7349.19	74
H33	5708.31	2618.04	6693.12	76
H34	7138.04	3801.45	5950.13	76
H35	7149.86	5705.27	6030.25	63
H36A	6247.88	7107.45	7049.78	53
H36B	5465.38	6660.24	5695.88	53
H38	7408.08	5514.72	6496.58	68
H39	7371.94	3687.35	6727.06	64
H40	5261.43	2414.43	6633.32	61
H41	3293.25	2958.81	6586.15	66
H42	3244.49	4801.2	6335.31	77
H6	1282(16)	8524(12)	7581(12)	25(3)
H3	4123(16)	9217(12)	6362(13)	29(4)
H2A	2110(16)	7601(13)	5304(14)	32(4)
H21A	-103(19)	4691(14)	7251(15)	41(4)
H2B	1079(17)	8165(13)	5744(13)	31(4)
H4A	3525(18)	10910(14)	7012(14)	35(4)
H12	3796(18)	8682(14)	4352(14)	38(4)
H4B	2037(18)	10107(13)	6993(13)	33(4)
H8	1596(18)	10726(14)	5232(15)	40(4)
H25	3126(19)	2023(15)	9692(15)	44(4)
H19	3422(19)	7312(14)	9782(15)	39(4)
H27	-68(19)	2253(14)	6853(15)	42(4)
H11	3176(18)	9162(14)	2527(15)	40(4)
H1	3832(19)	9104(14)	9355(16)	41(4)
H9	1030(20)	11265(16)	3476(16)	51(5)
H23	3037(18)	5131(14)	8946(14)	37(4)
H21B	254(19)	4119(15)	6203(16)	46(5)
H26	1011(19)	1163(15)	8067(15)	43(4)
H18	2540(20)	6397(16)	10961(17)	52(5)
H24	4160(20)	4006(16)	10129(17)	54(5)
H13A	1390(20)	10281(15)	204(16)	47(5)
H15	-570(20)	7614(17)	7948(18)	57(5)
H13B	2930(20)	10210(17)	1058(17)	58(5)
H13C	1490(20)	9143(19)	740(18)	62(6)
H16	-1510(30)	6590(20)	9200(20)	77(7)
H17	60(20)	6006(19)	10660(20)	71(6)

Table 7: Atomic Occupancies for all atoms that are not fully occupied in **5a.a**.

Atom	Occupancy	Atom	Occupancy
C29	0.741(6)	H38	0.259(6)
H29A	0.741(6)	C39	0.259(6)
H29B	0.741(6)	H39	0.259(6)
C30	0.741(6)	C40	0.259(6)
C31	0.741(6)	H40	0.259(6)
H31	0.741(6)	C41	0.259(6)
C32	0.741(6)	H41	0.259(6)
H32	0.741(6)	C42	0.259(6)
C33	0.741(6)	H42	0.259(6)
H33	0.741(6)		
C34	0.741(6)		
H34	0.741(6)		
C35	0.741(6)		
H35	0.741(6)		
C36	0.259(6)		
H36A	0.259(6)		
H36B	0.259(6)		
C37	0.259(6)		
C38	0.259(6)		

NMR spectra of new compounds

N-(1-((*tert*-Butyldimethylsilyl)oxy)vinyl)-1-(4-(trifluoromethyl)phenyl)methanimine (4b)

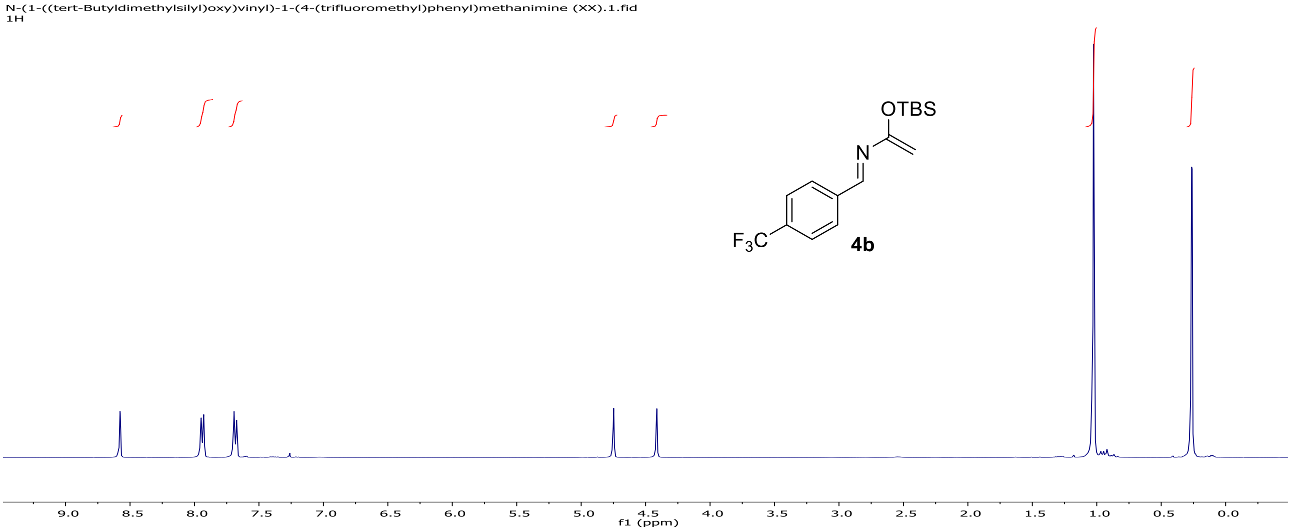

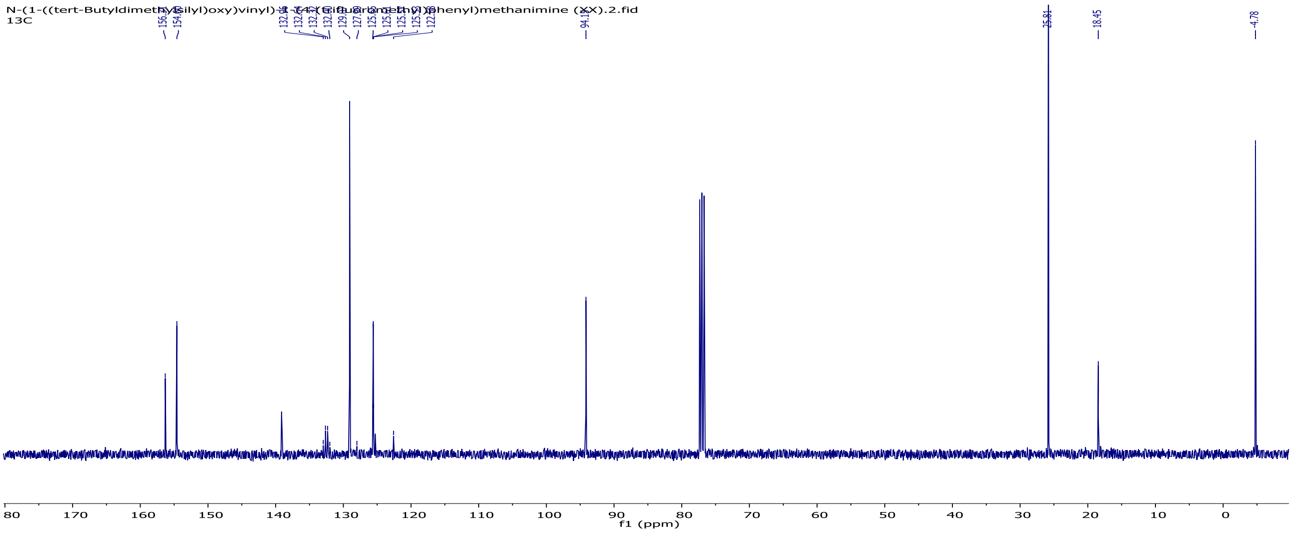

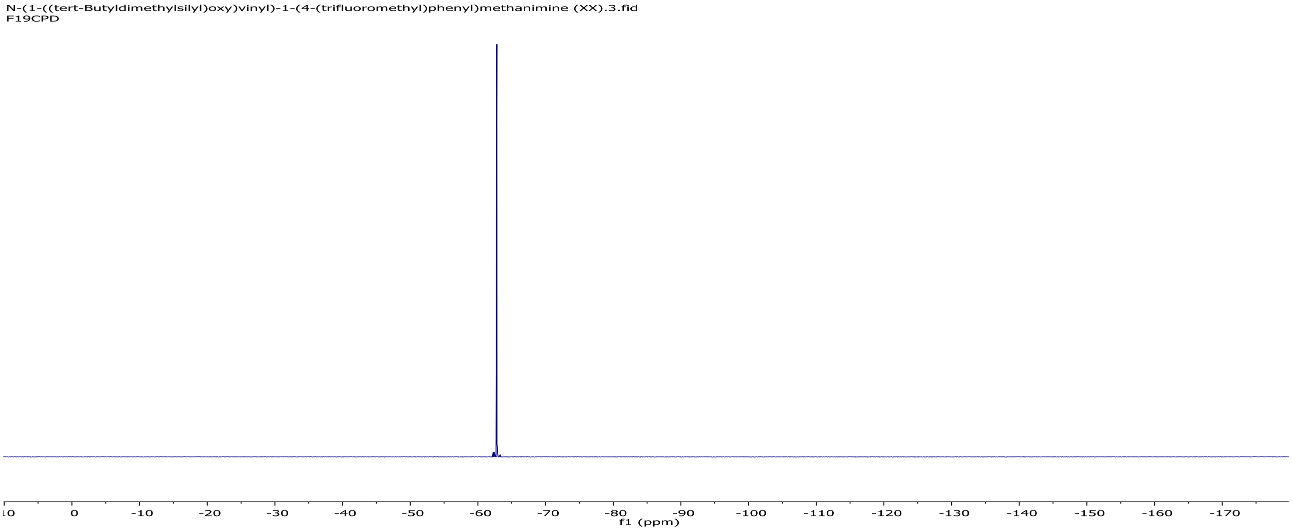

N-(1-((*tert*-Butyldimethylsilyl)oxy)vinyl)-1-(4-chlorophenyl)methanimine (4c)

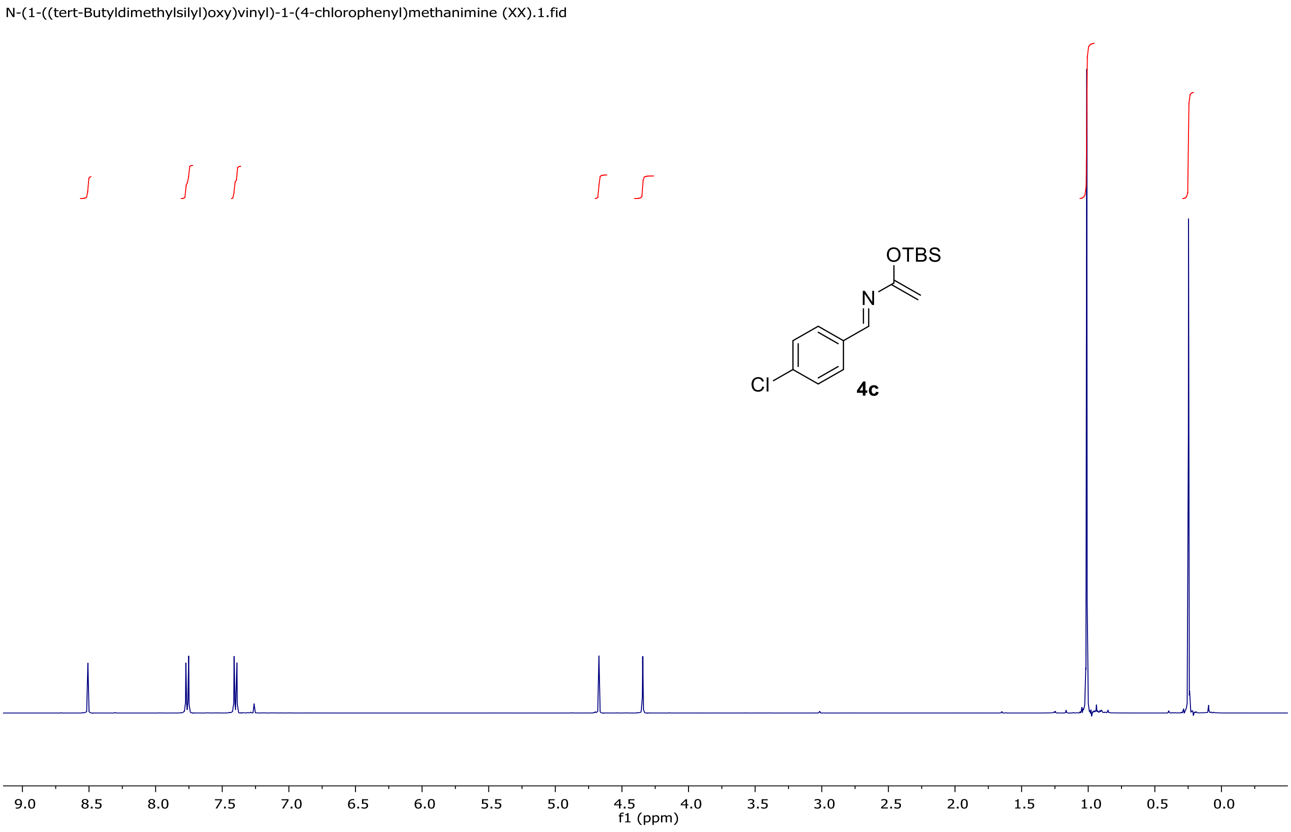

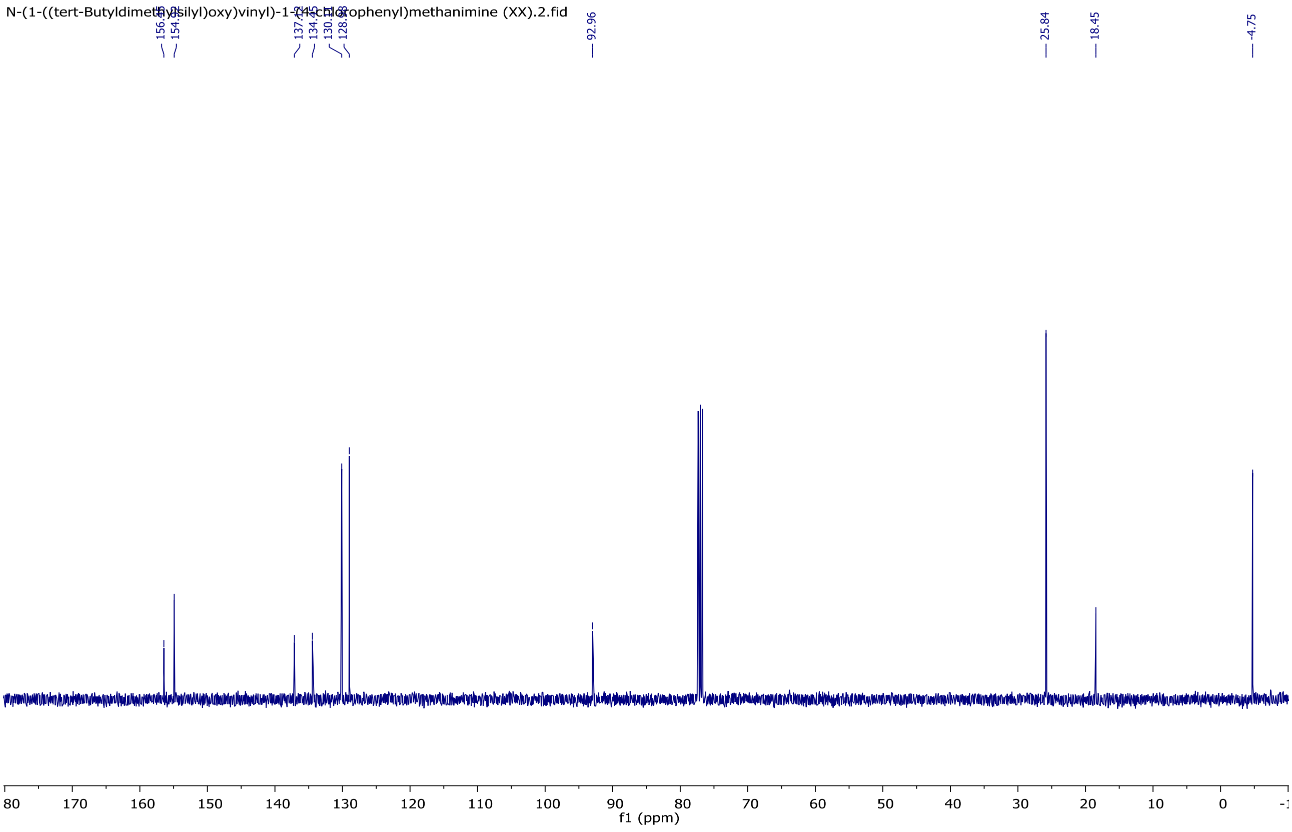

N-(1-((*tert*-Butyldimethylsilyl)oxy)vinyl)-1-(2-fluorophenyl)methanimine (4d)

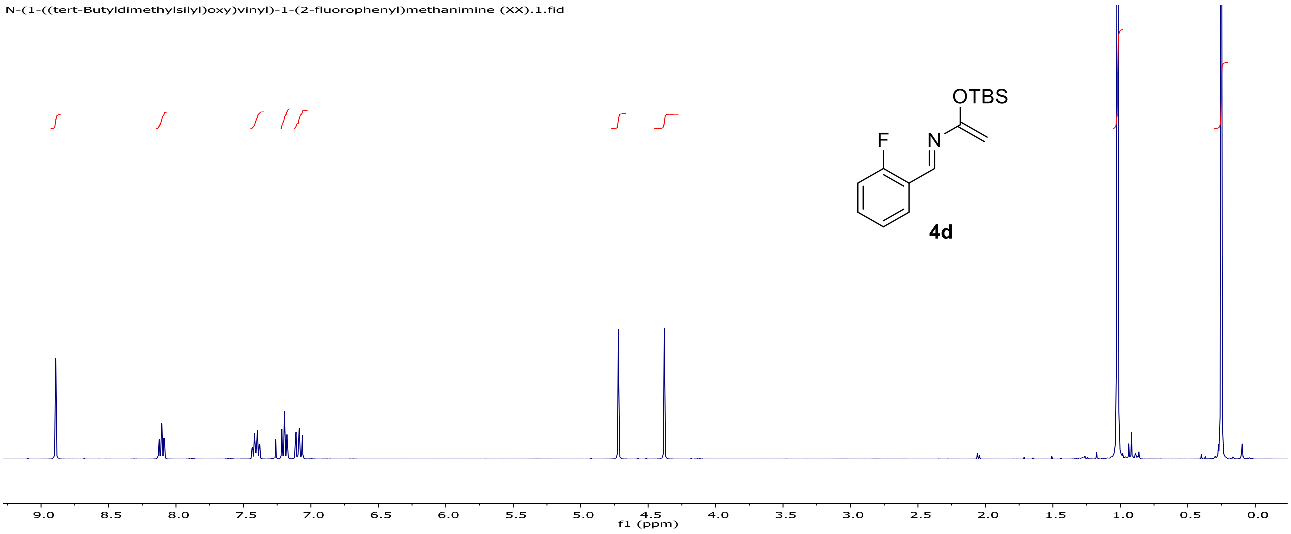

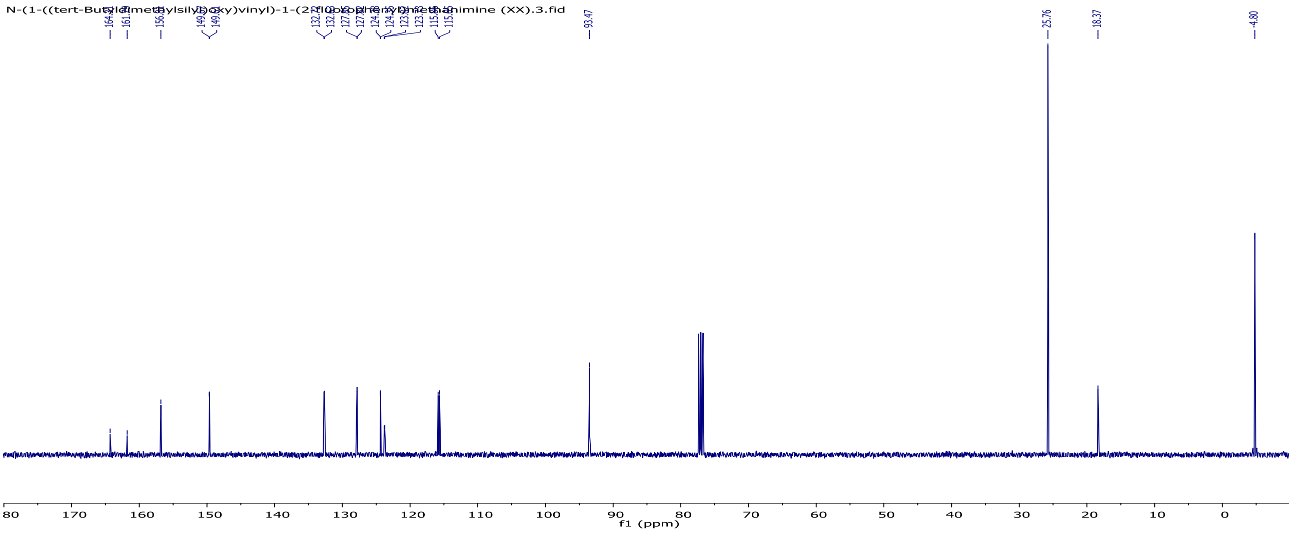

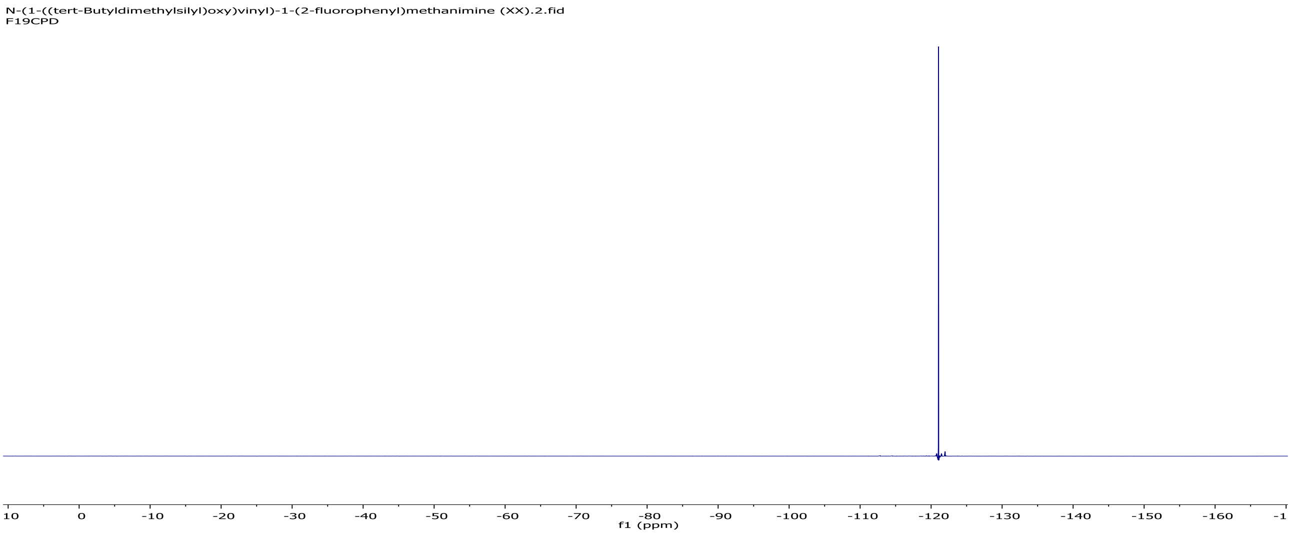

1-(3-Bromophenyl)-N-(1-((*tert*-butyldimethylsilyl)oxy)vinyl)methanimine (4e)

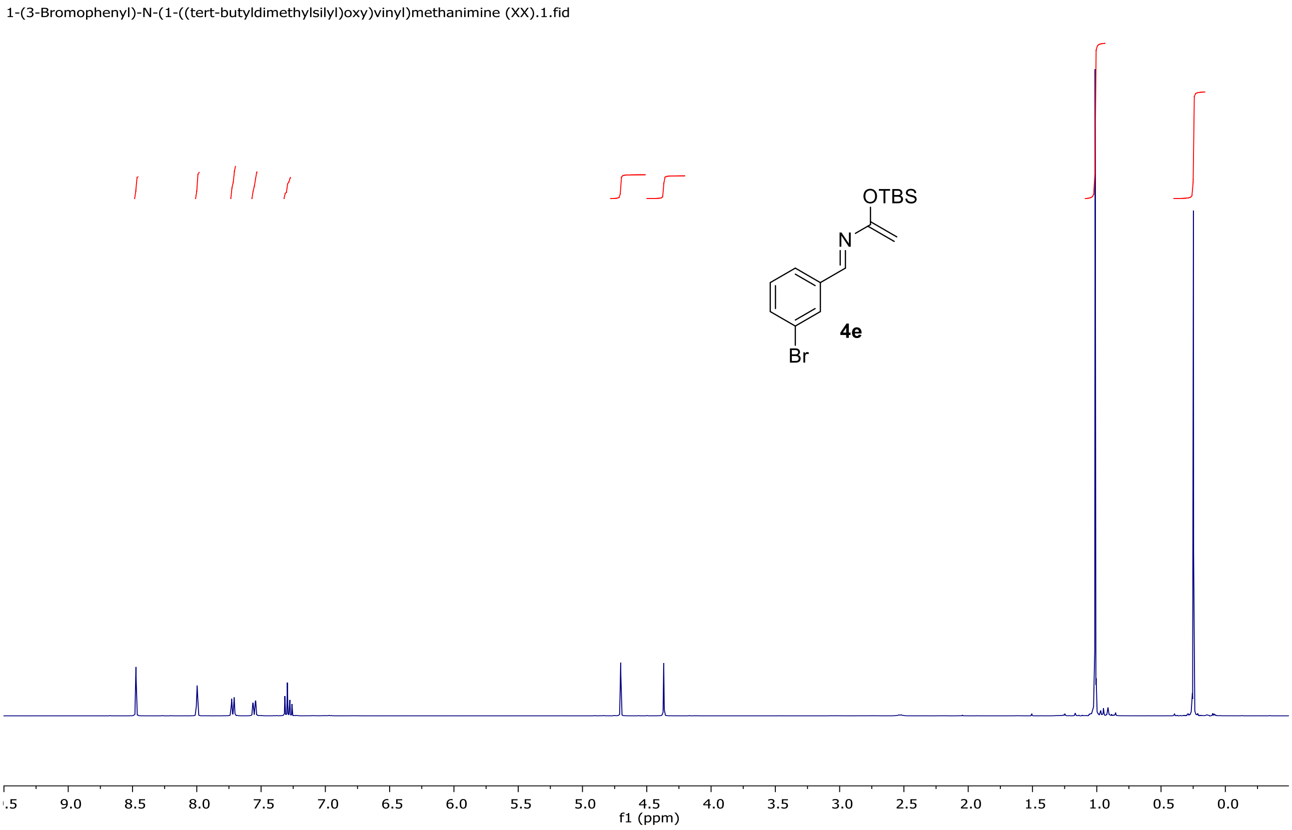

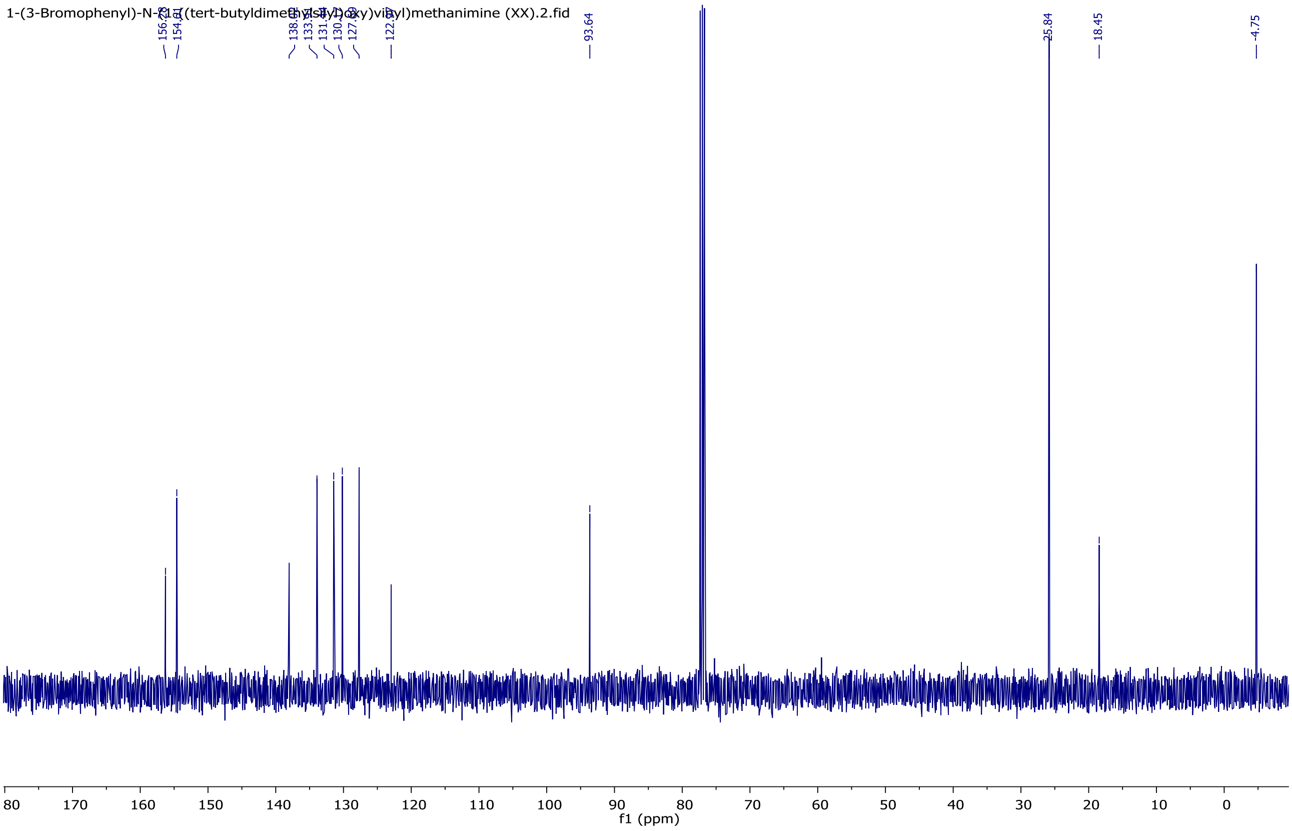

N-(1-((*tert*-Butyldimethylsilyl)oxy)vinyl)-1-(4-methoxyphenyl)methanimine (4f)

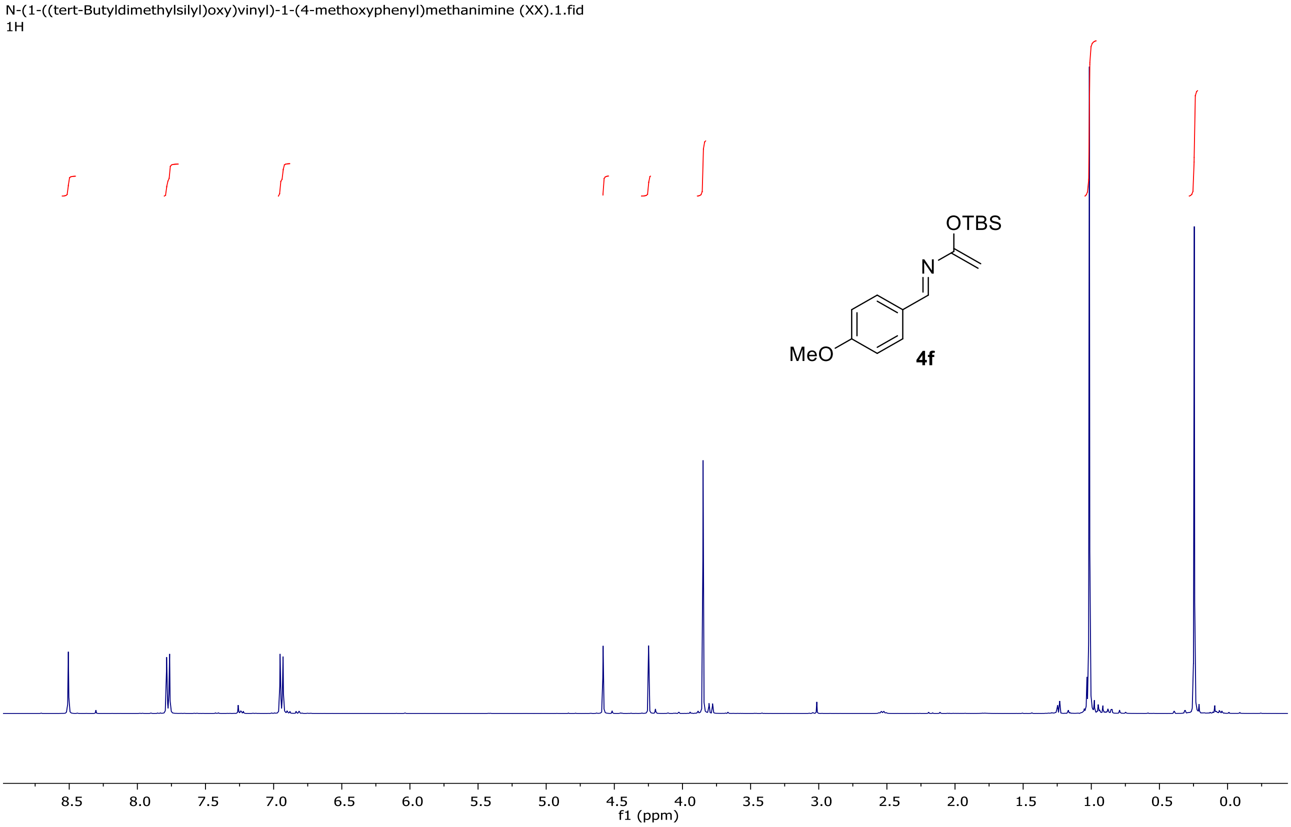

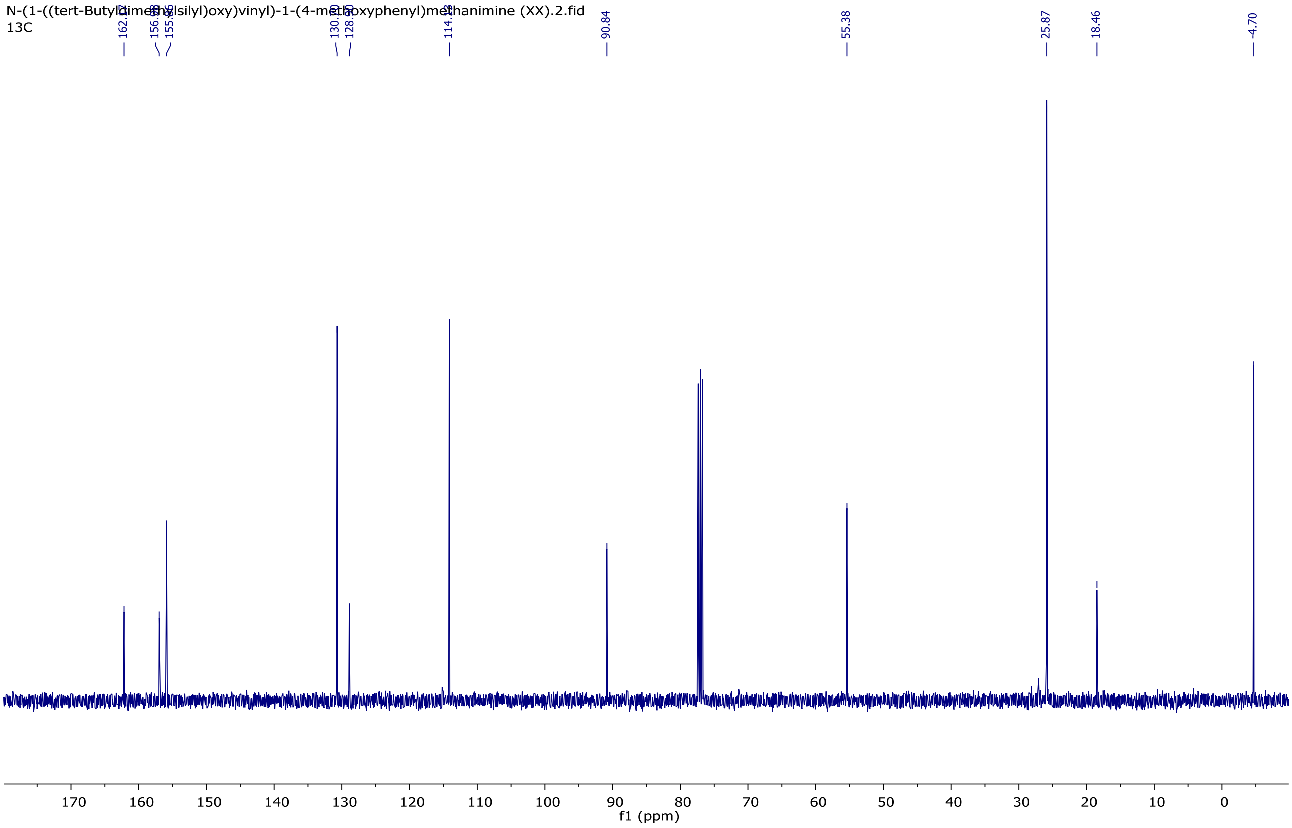

N-(1-((*tert*-Butyldimethylsilyl)oxy)vinyl)-2-methyl-3-phenylprop-2-en-1-imine (4g)

N-(1-((tert-Butyldimethylsilyl)oxy)vinyl)-2-methyl-3-phenylprop-2-en-1-imine (XX).1.fid

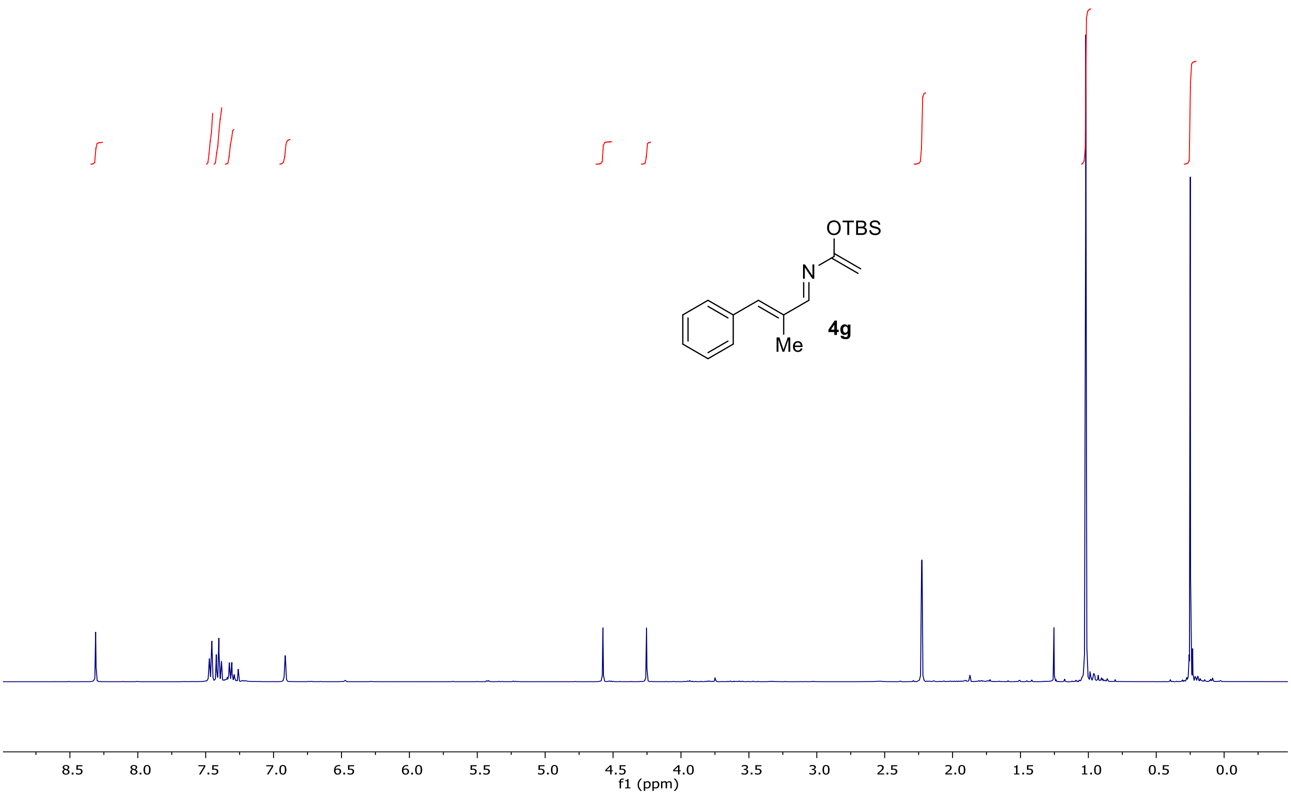

N-(1-((tert-Butyldimethylsilyl)oxy)vinyl)-2-methyl-3-phenylprop-2-en-1-imine (XX).2.fid

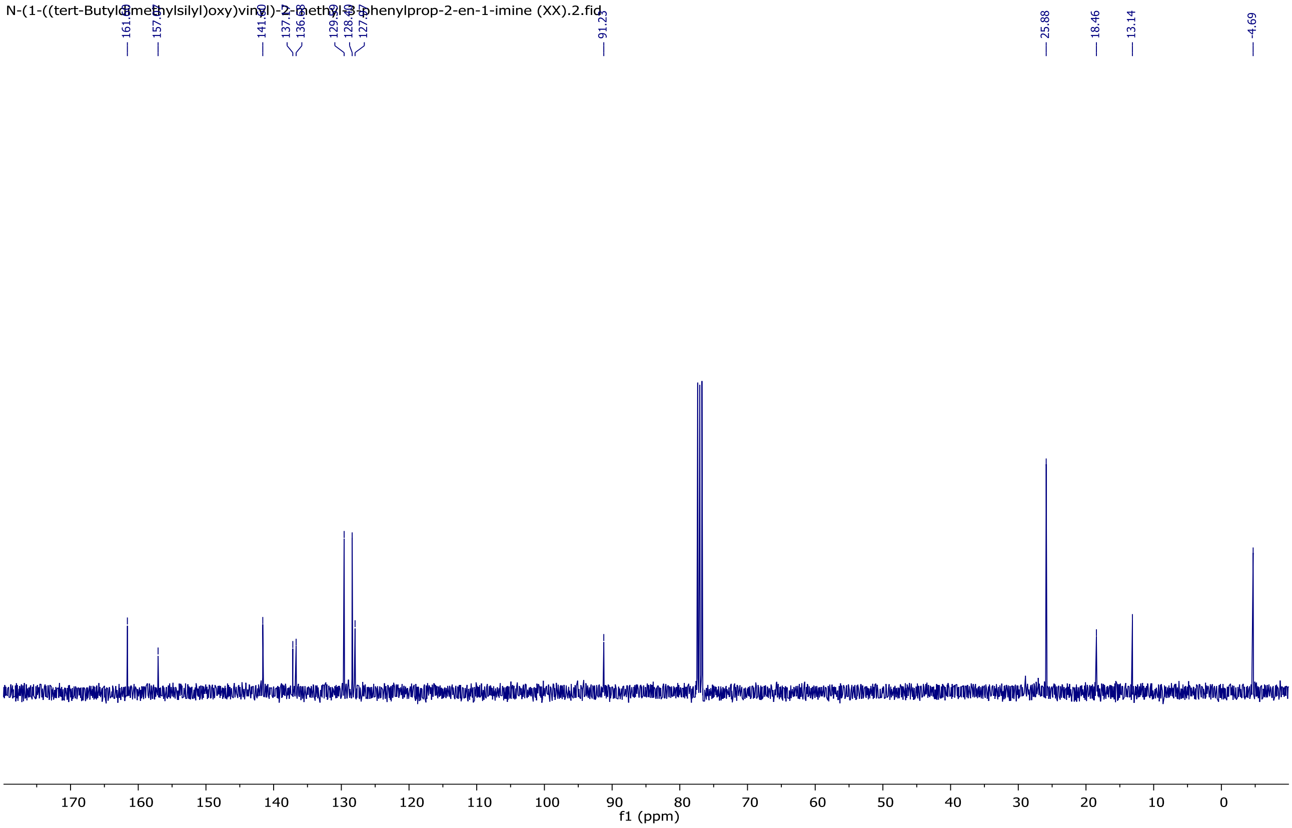

Di*iso*propyl 2-(4-methoxyphenyl)cyclopropane-1,1-dicarboxylate (2a'')

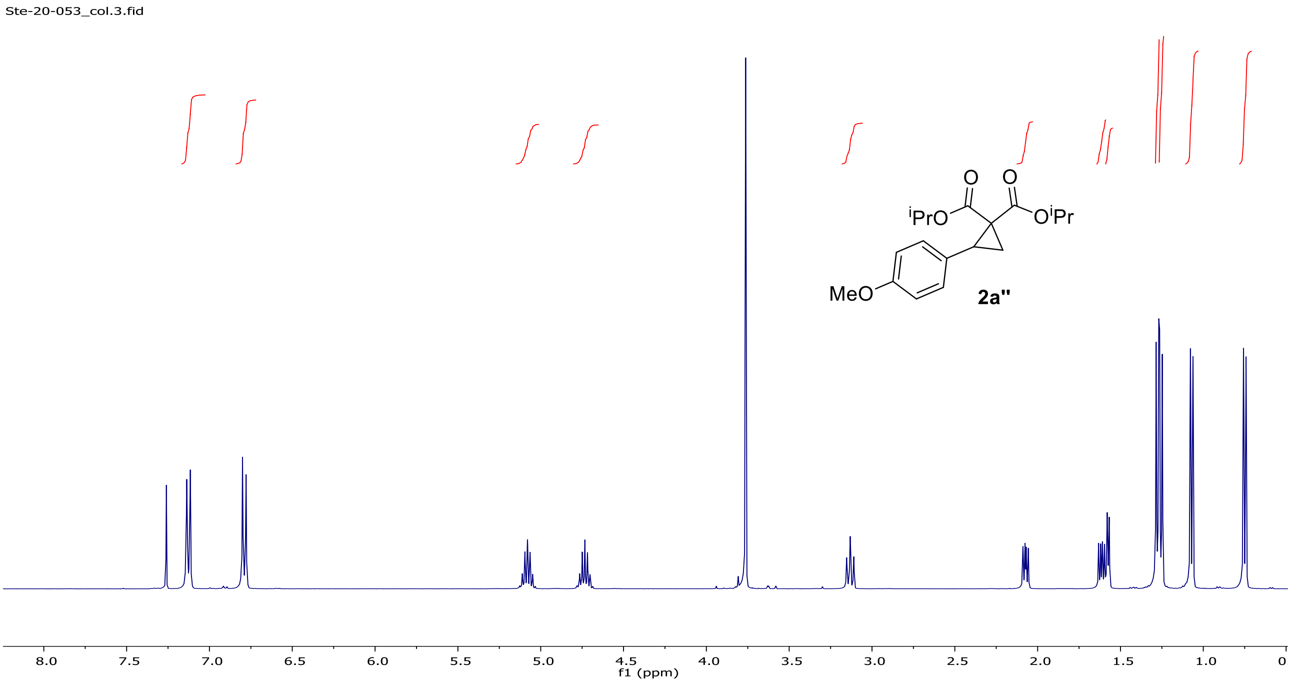

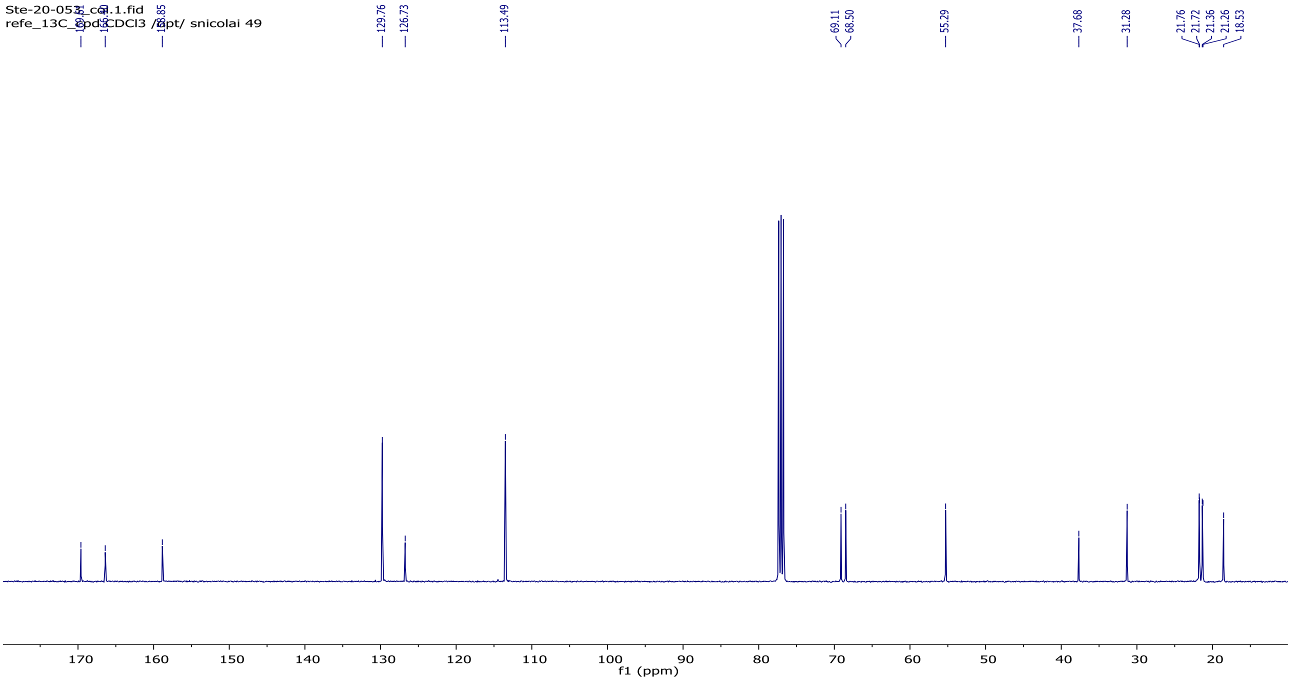

Dibenzyl 2-(3,4-dimethoxyphenyl)cyclopropane-1,1-dicarboxylate (2b)

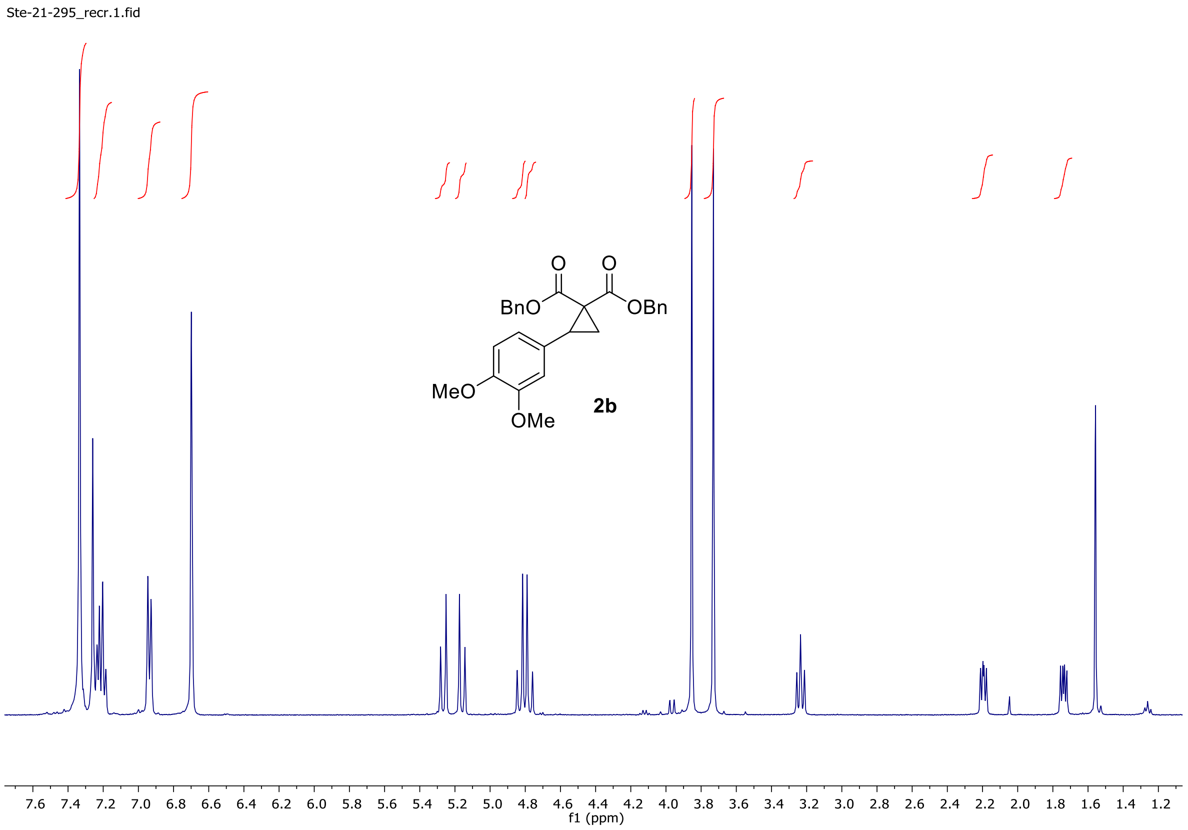

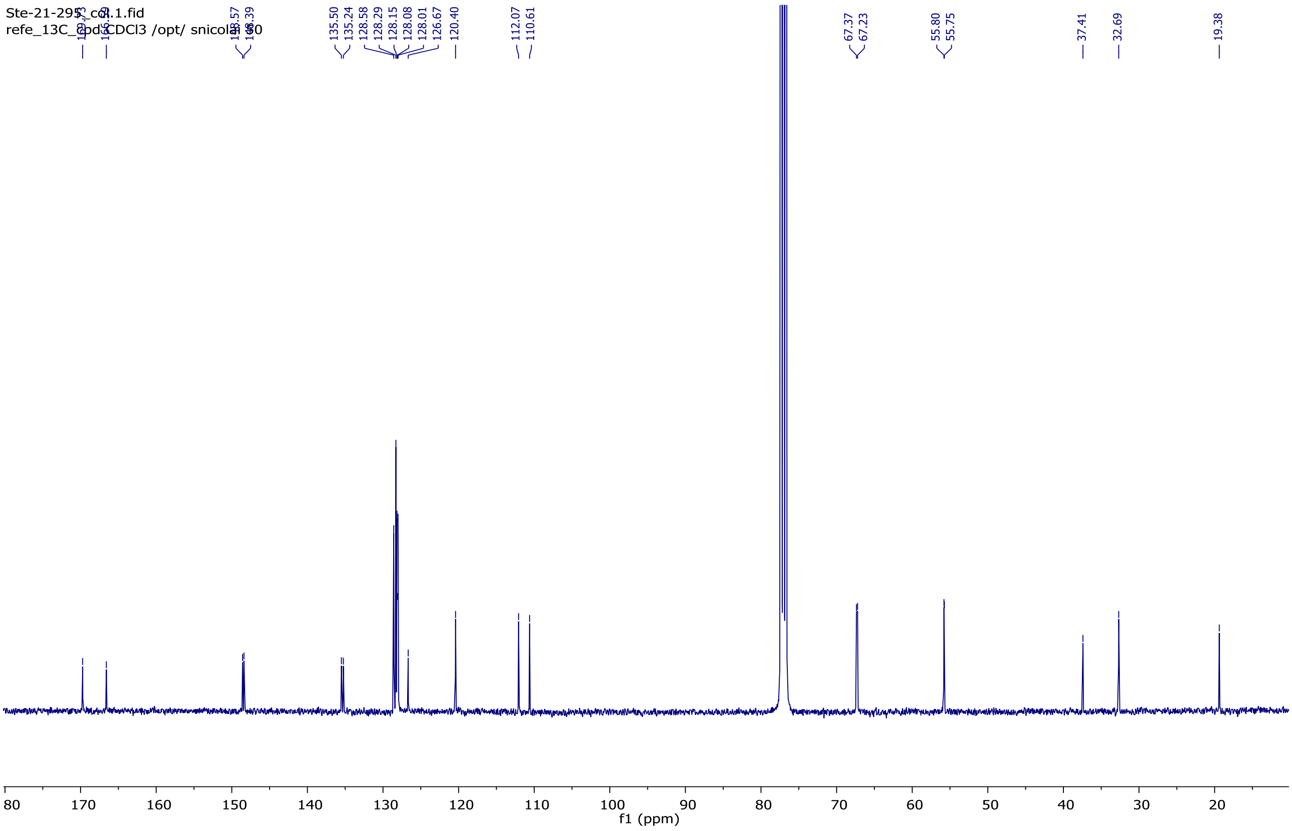

Dibenzyl 2-(2,4-dimethoxyphenyl)cyclopropane-1,1-dicarboxylate (2c)

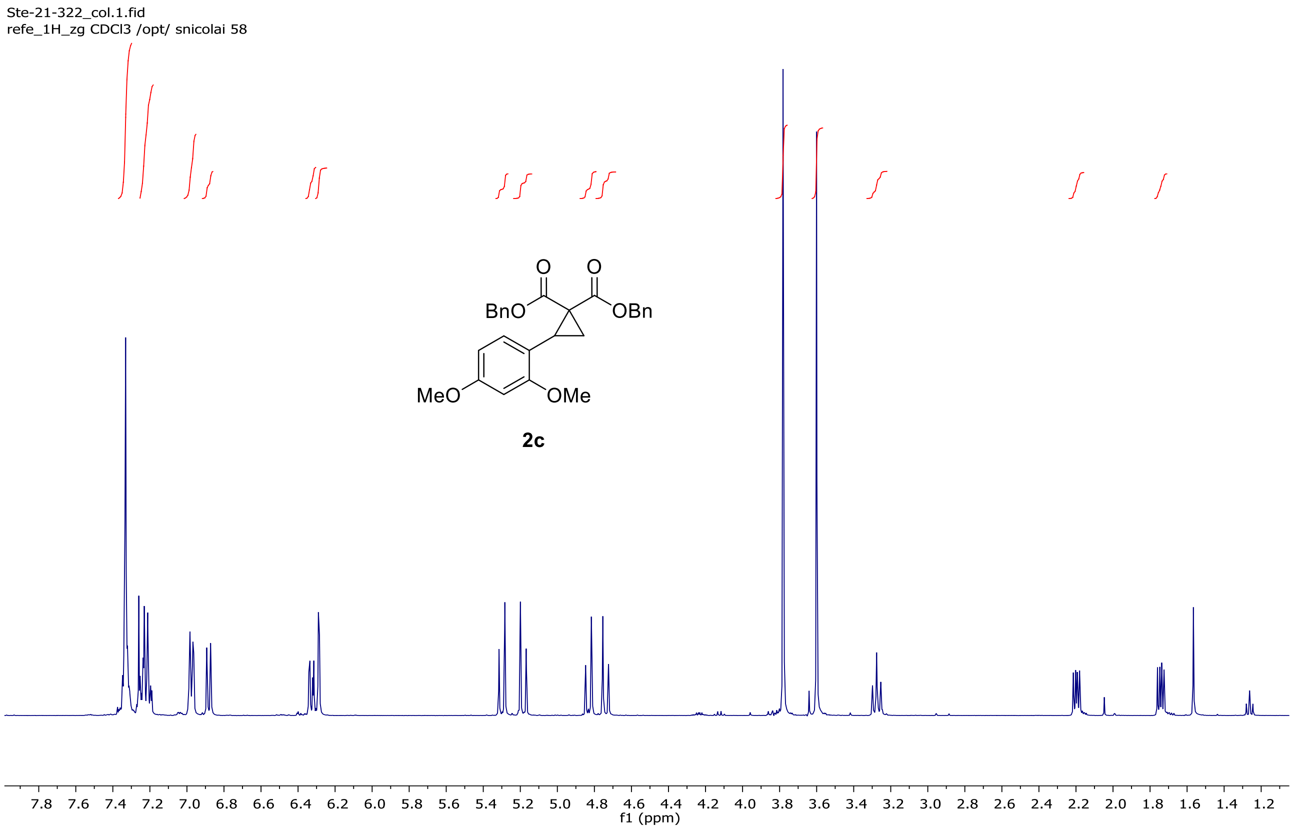

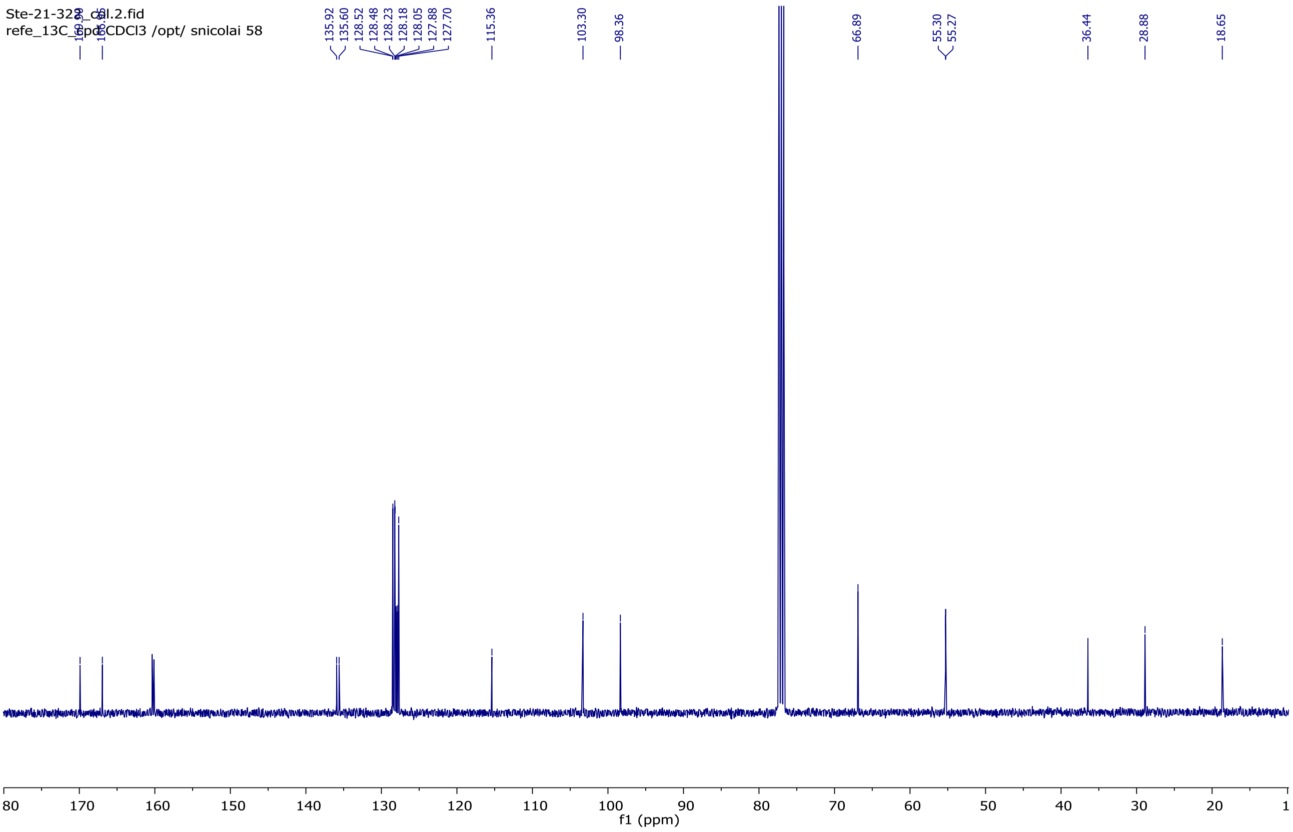

Dibenzyl 2-(2-methoxyphenyl)cyclopropane-1,1-dicarboxylate (2d)

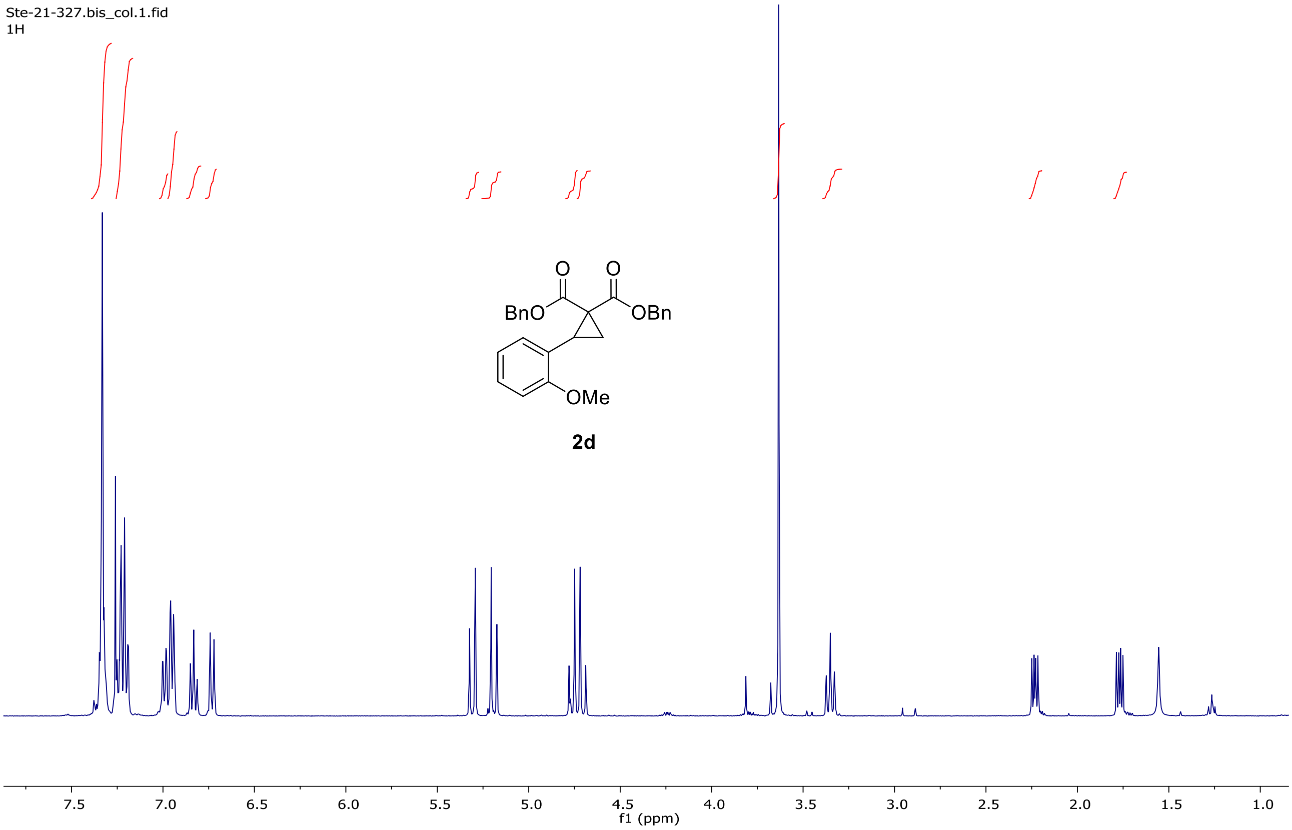

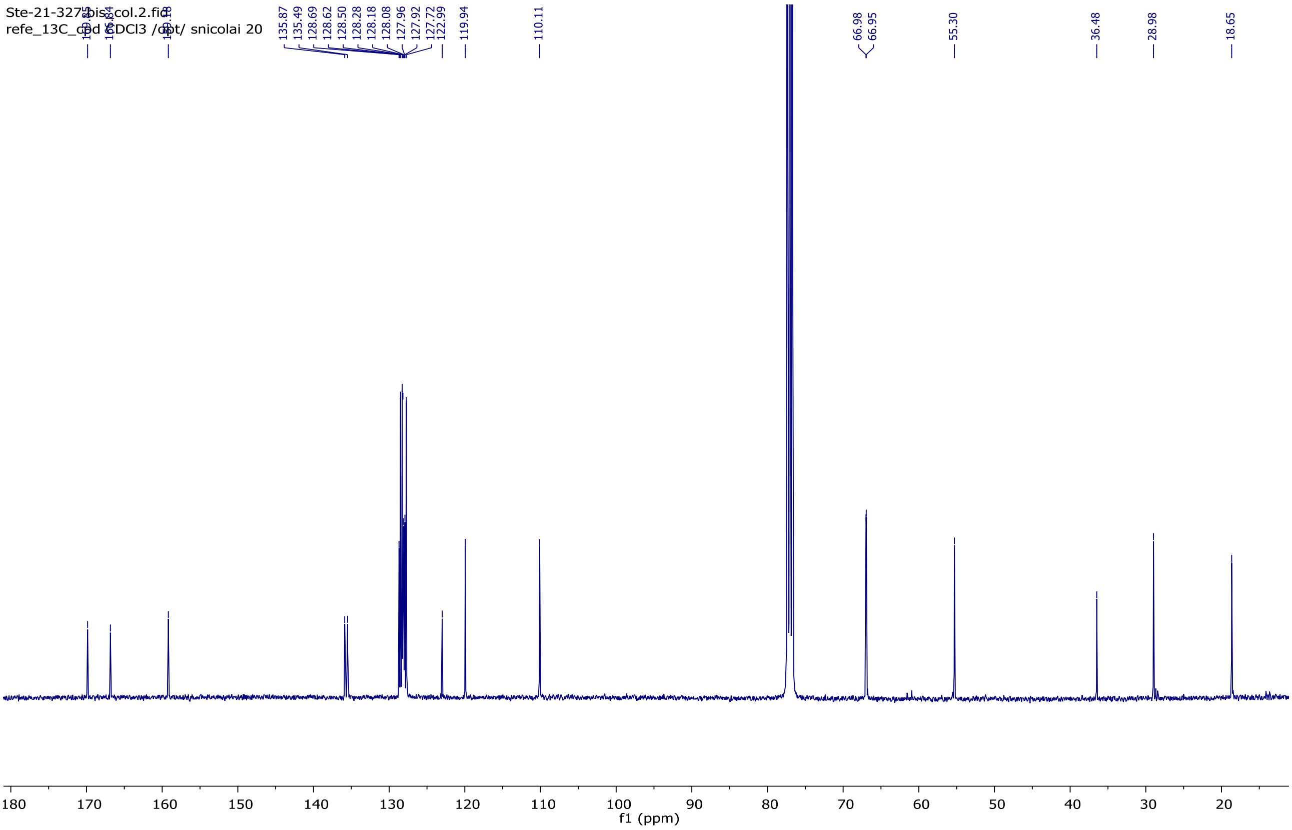

Dibenzyl 2-(3-iodo-4-methoxyphenyl)cyclopropane-1,1-dicarboxylate (2e)

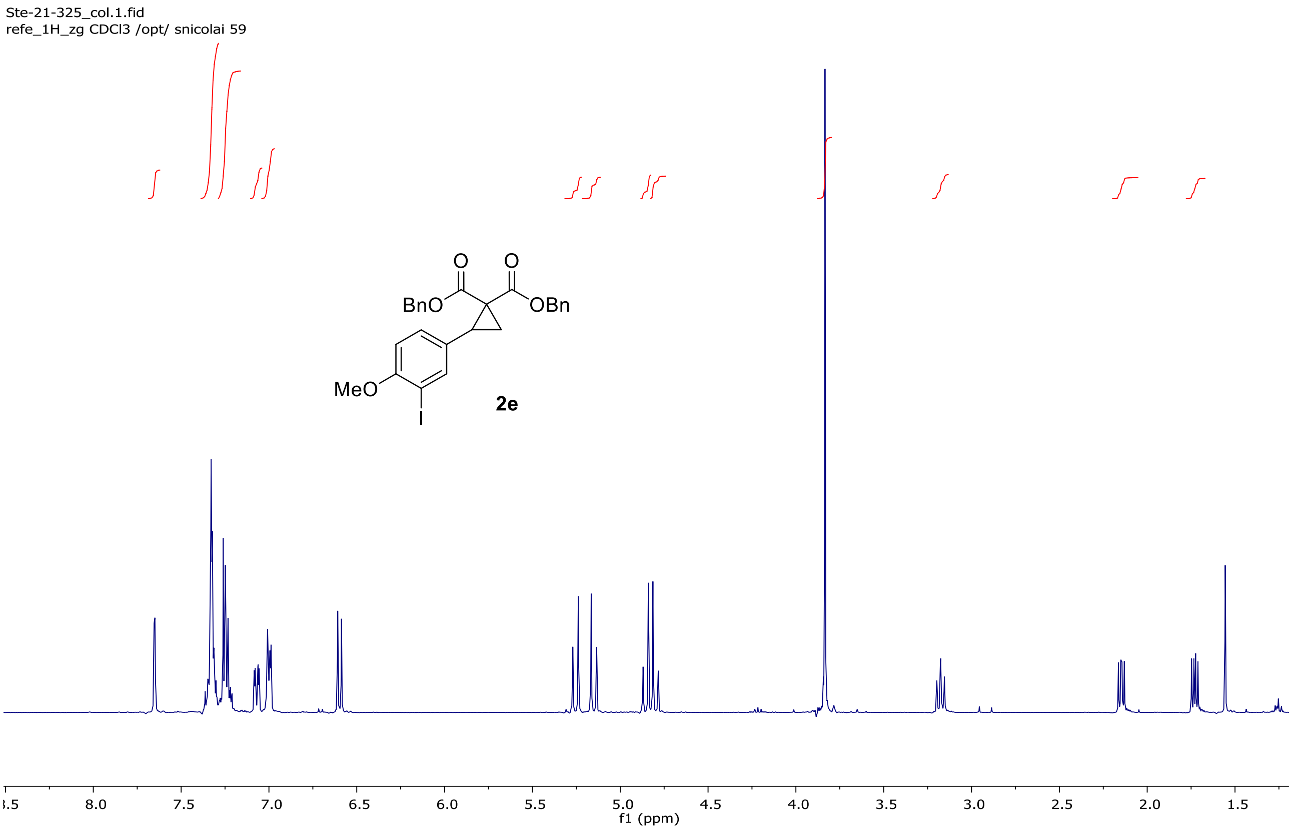

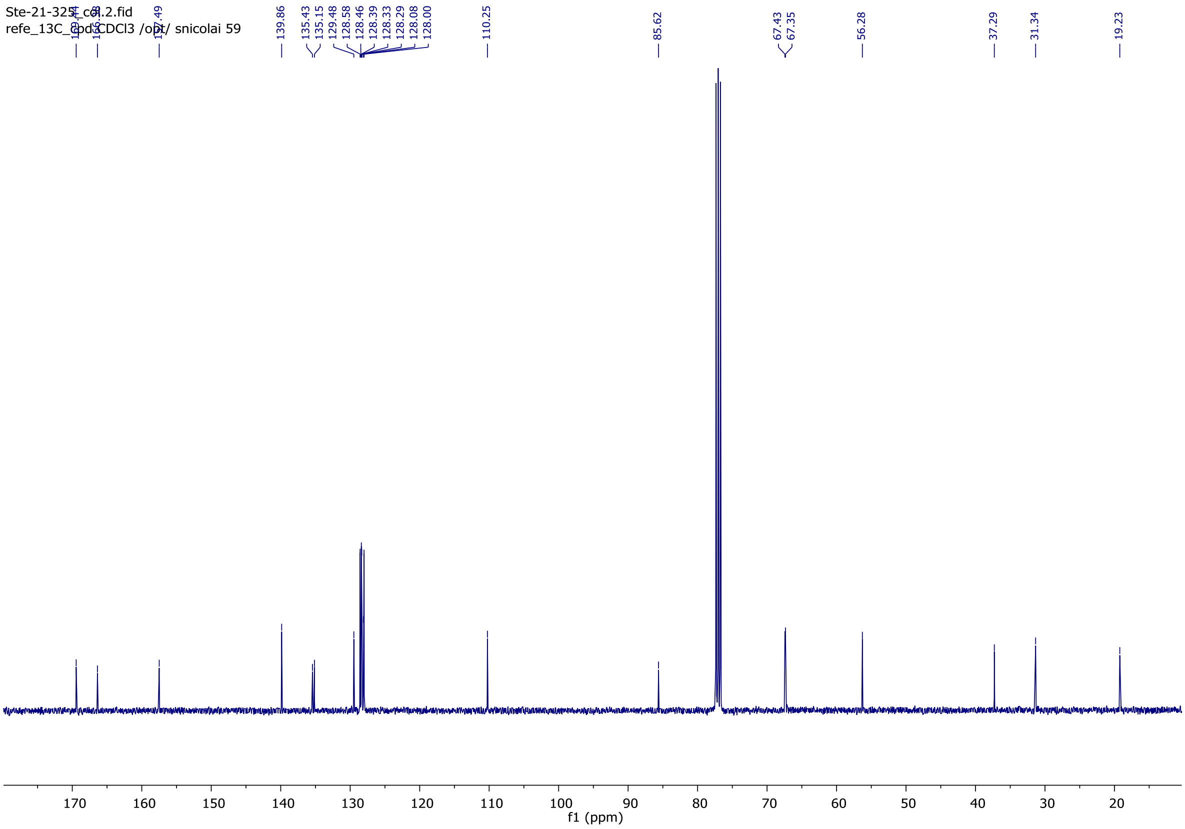

Dibenzyl 2-(1-tosyl-1*H*-indol-3-yl)cyclopropane-1,1-dicarboxylate (2g)

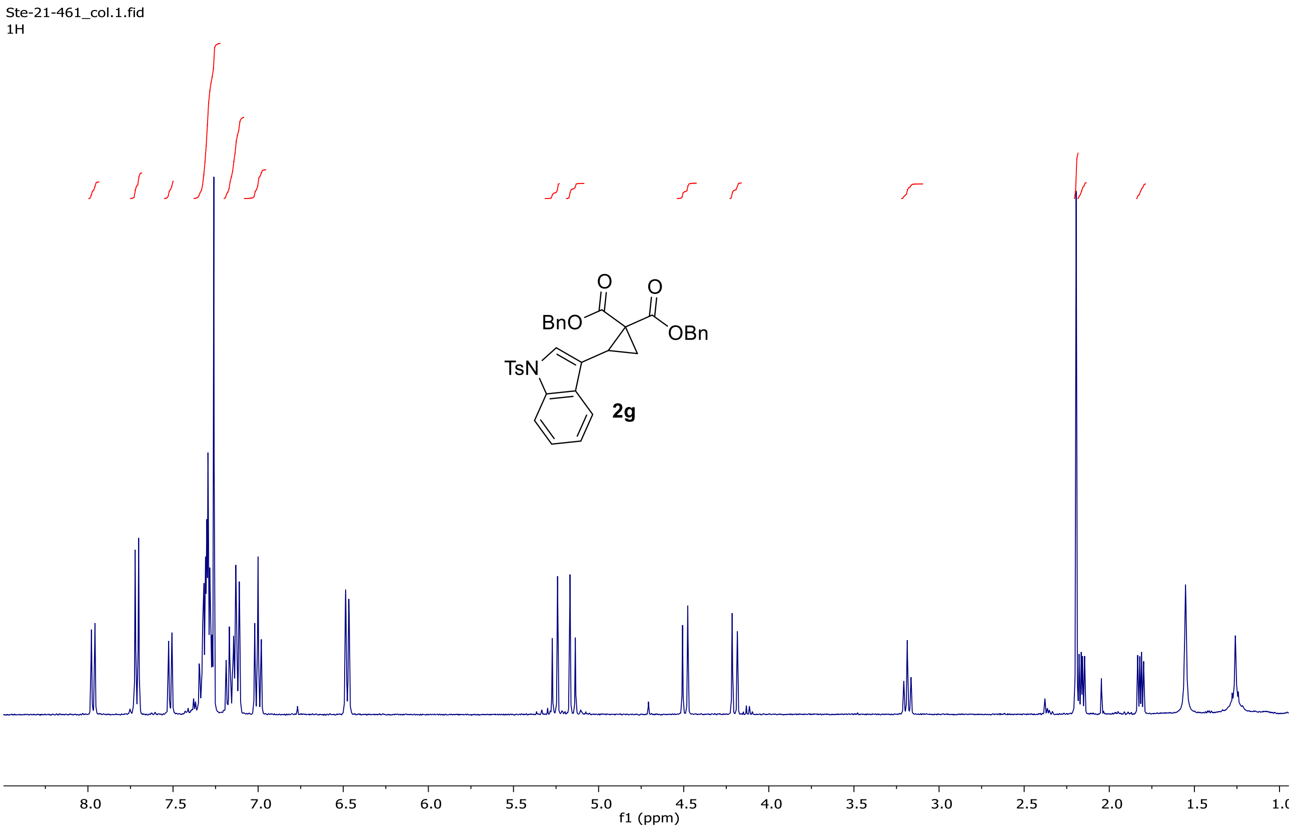

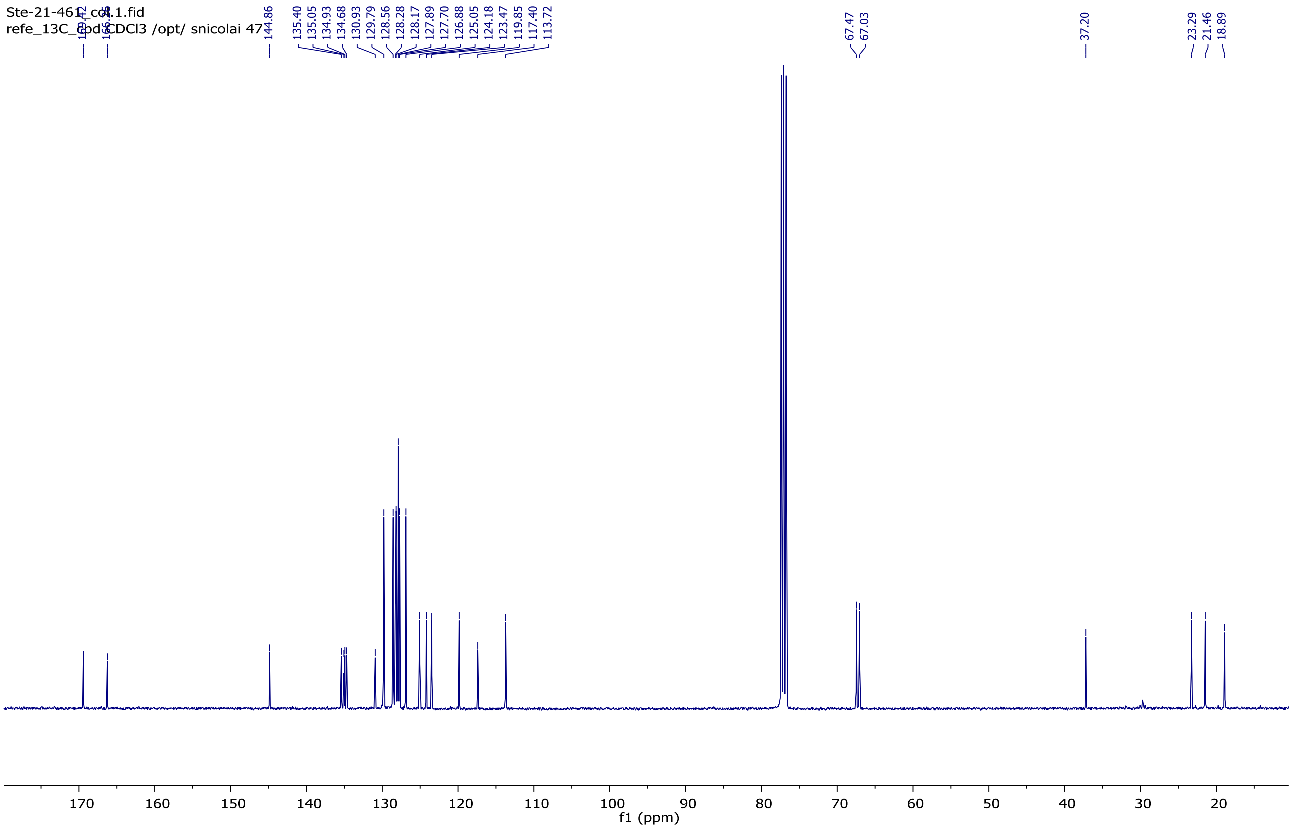

Dibenzyl 2-(benzo[b]thiophen-2-yl)cyclopropane-1,1-dicarboxylate (2h)

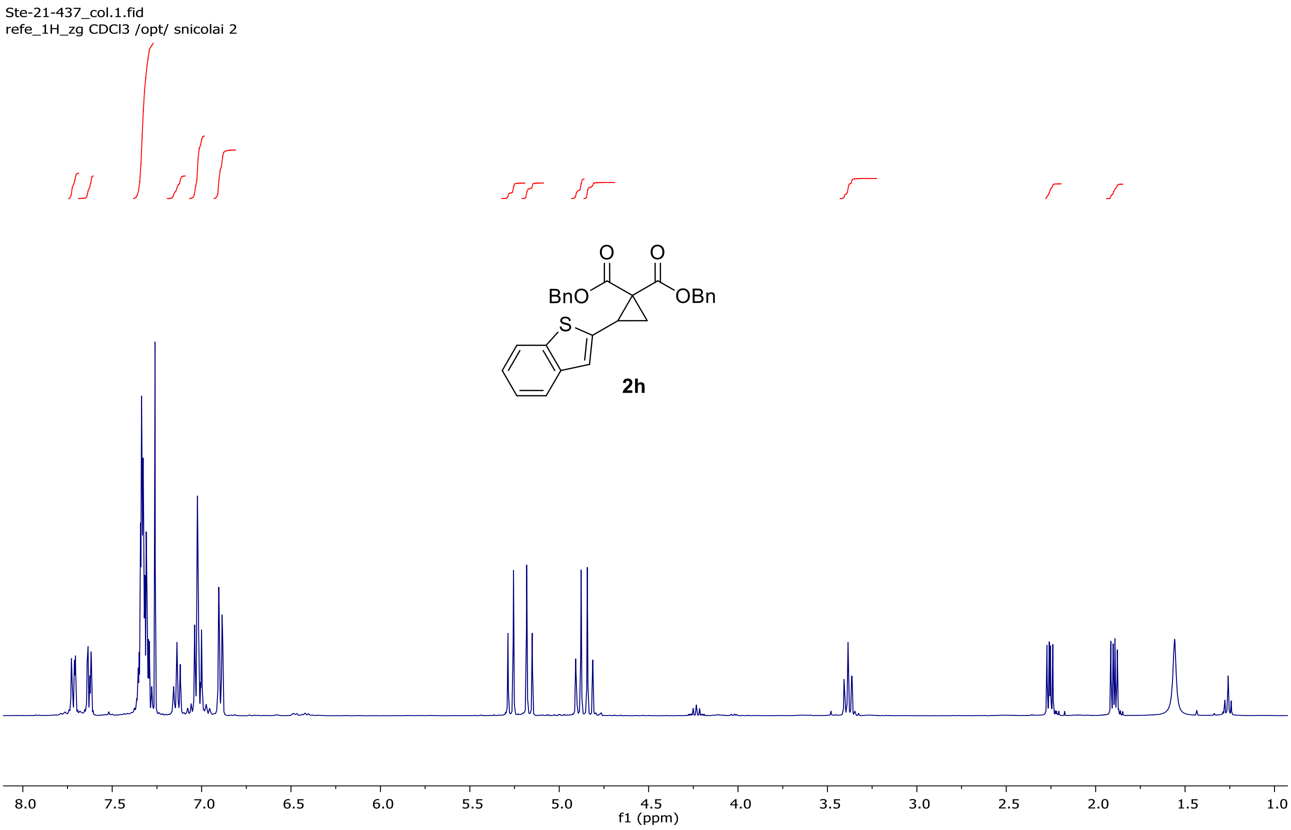

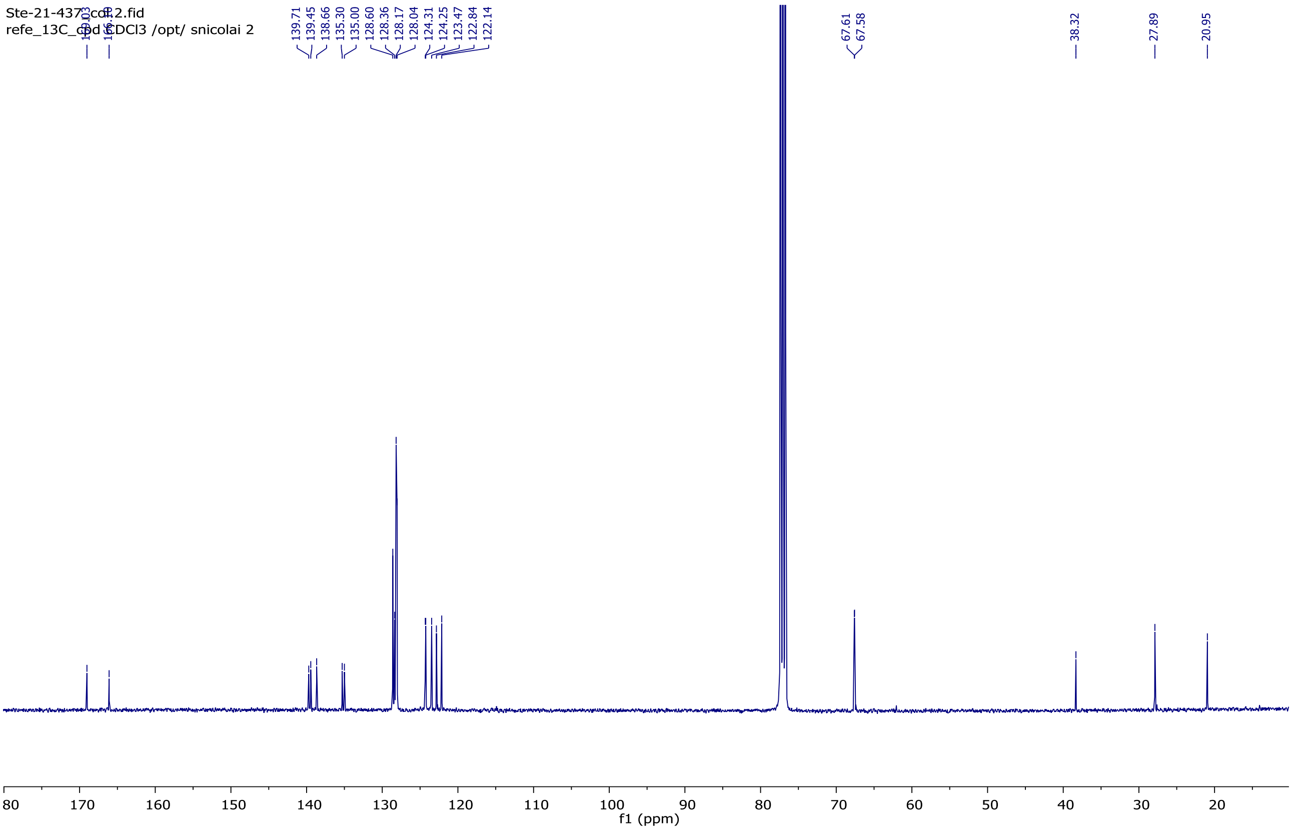

Dibenzyl 2-(1-phenyl-1*H*-pyrazol-4-yl)cyclopropane-1,1-dicarboxylate (2i)

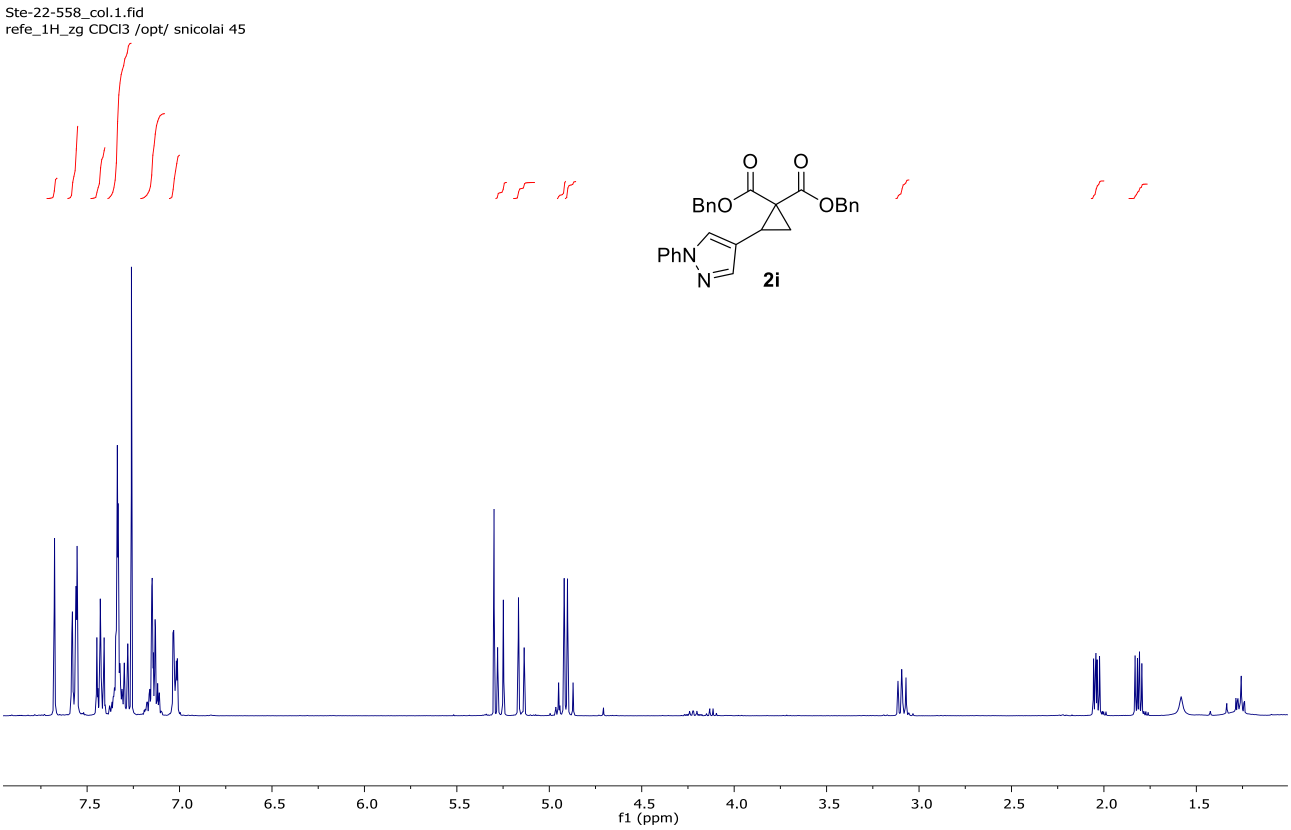

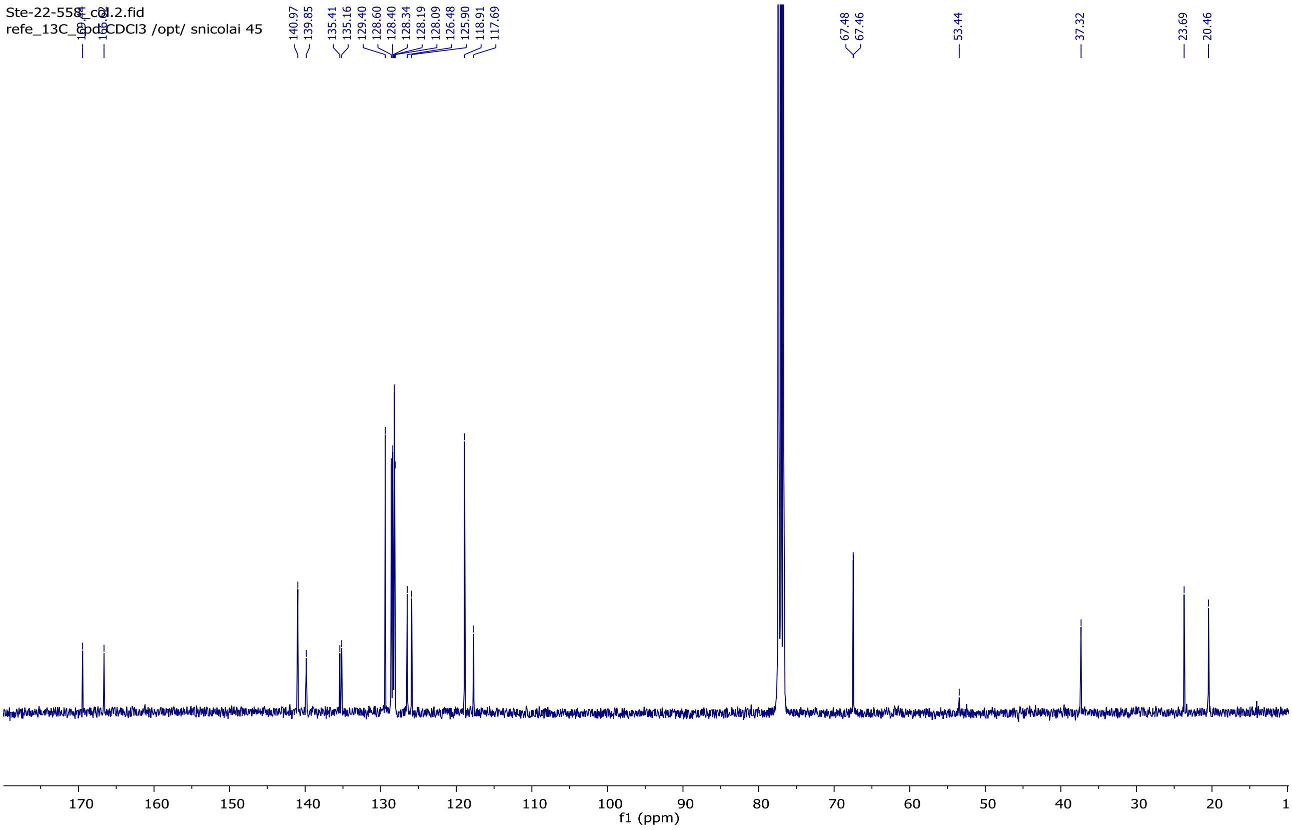

(E)-2-(1-phenylprop-1-en-2-yl)cyclopropane-1,1-dicarboxylate (2j)

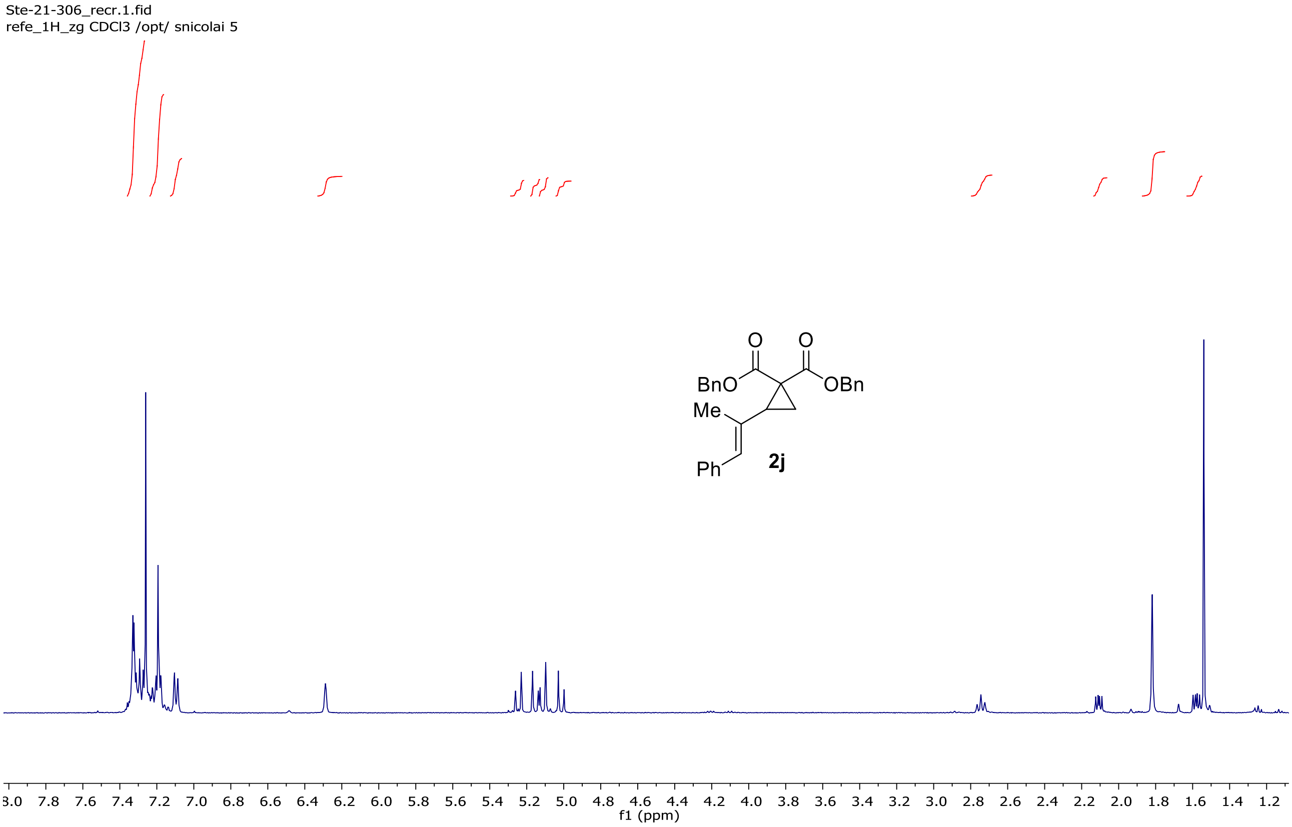

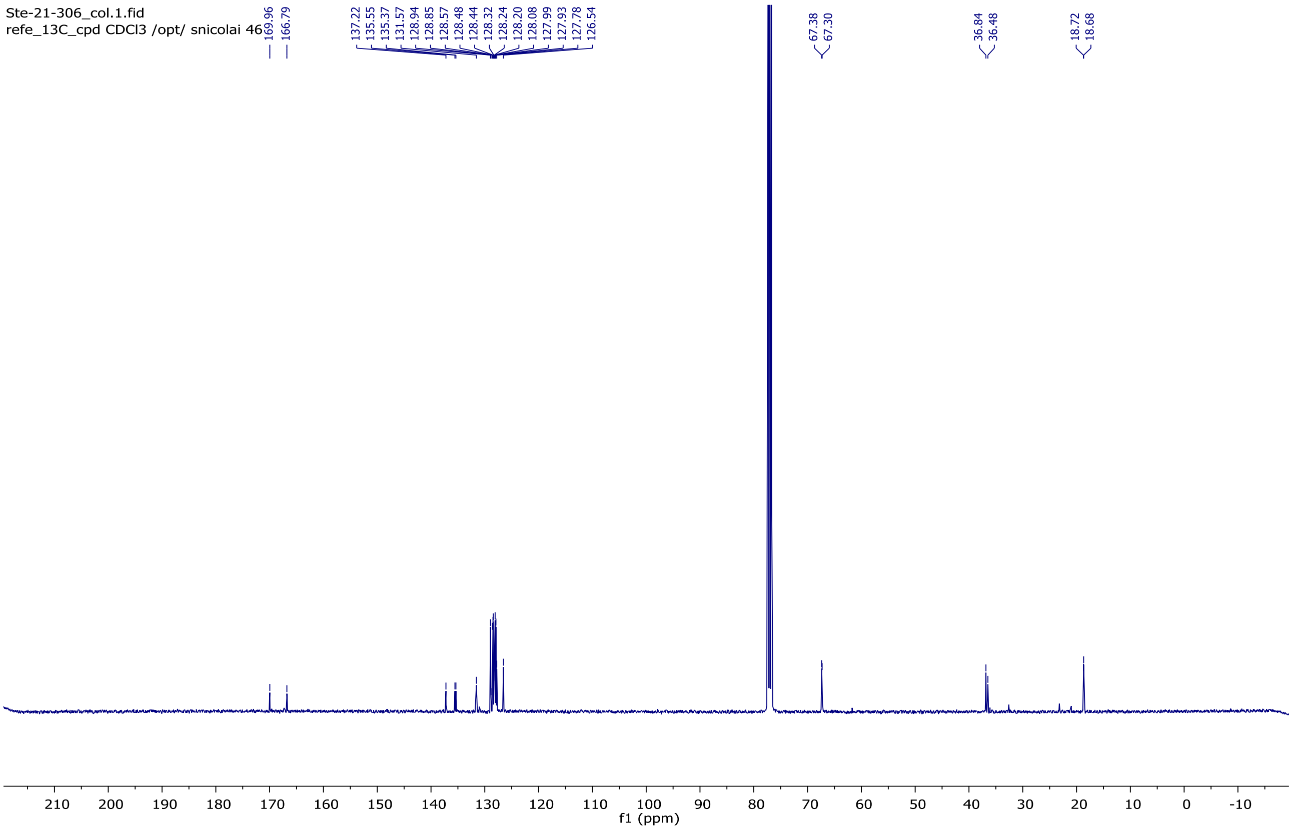

Dibenzyl (E)-2-styrylcyclopropane-1,1-dicarboxylate (2k)

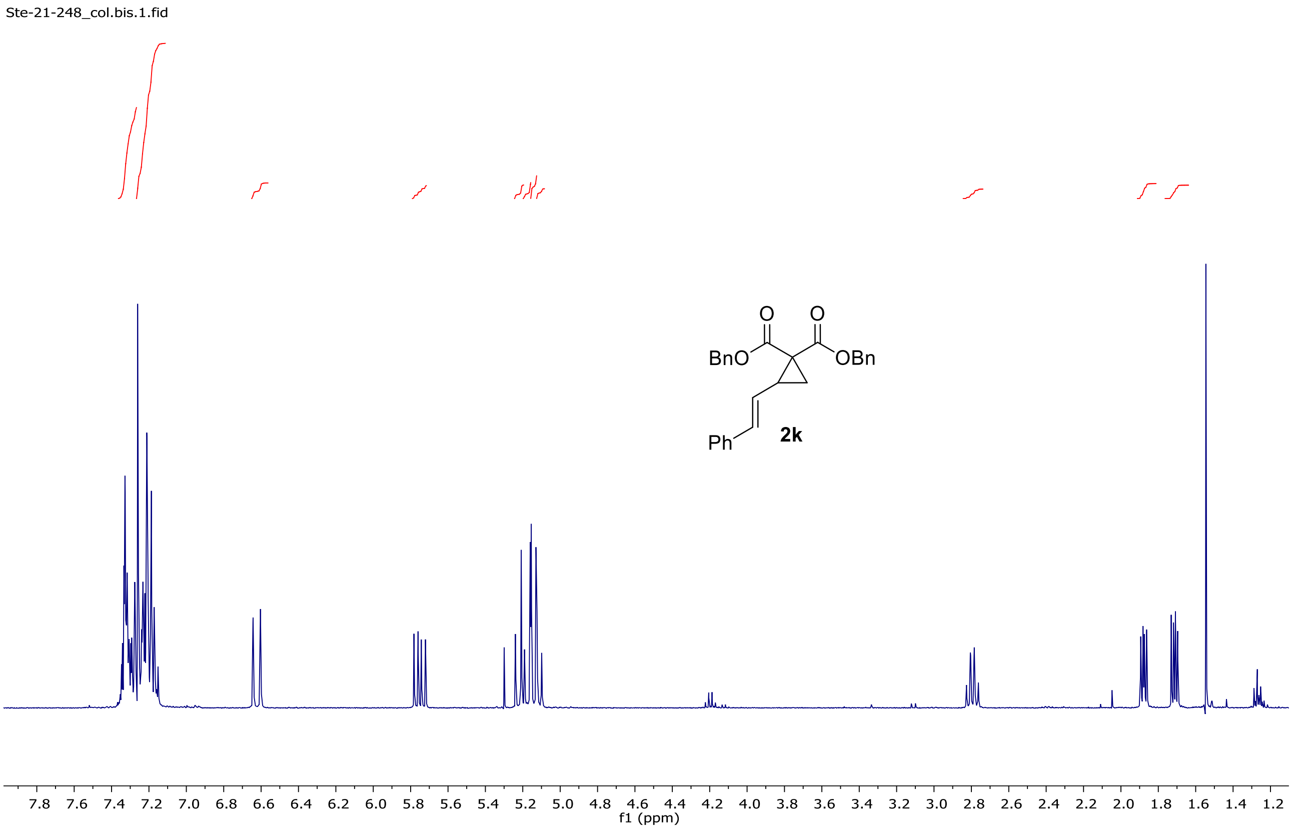

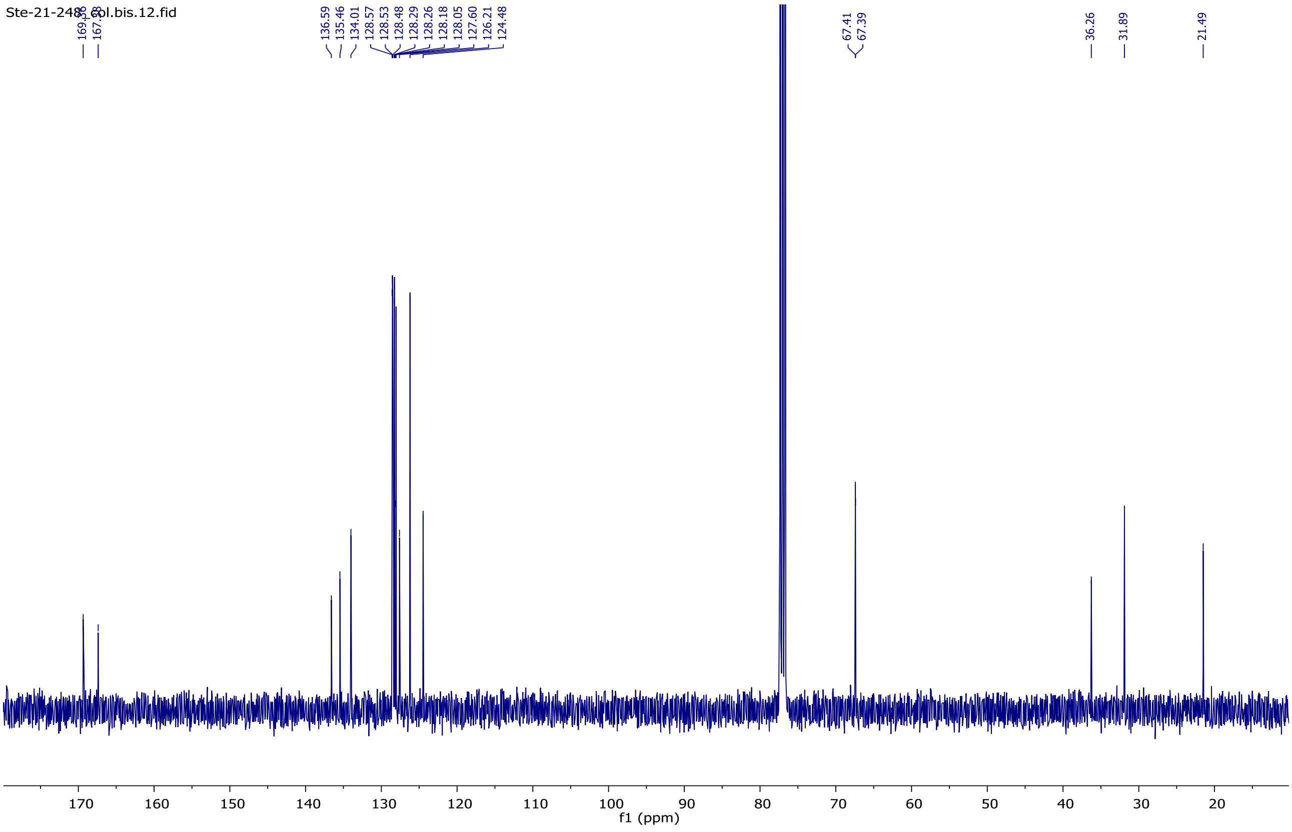

Dibenzyl 2-(3-(4-(*tert*-butyl)benzyl)-5-methyl-2,4-dioxo-3,4-dihydropyrimidin-1(2*H*)-yl)cyclopropane-1,1-dicarboxylate (2m)

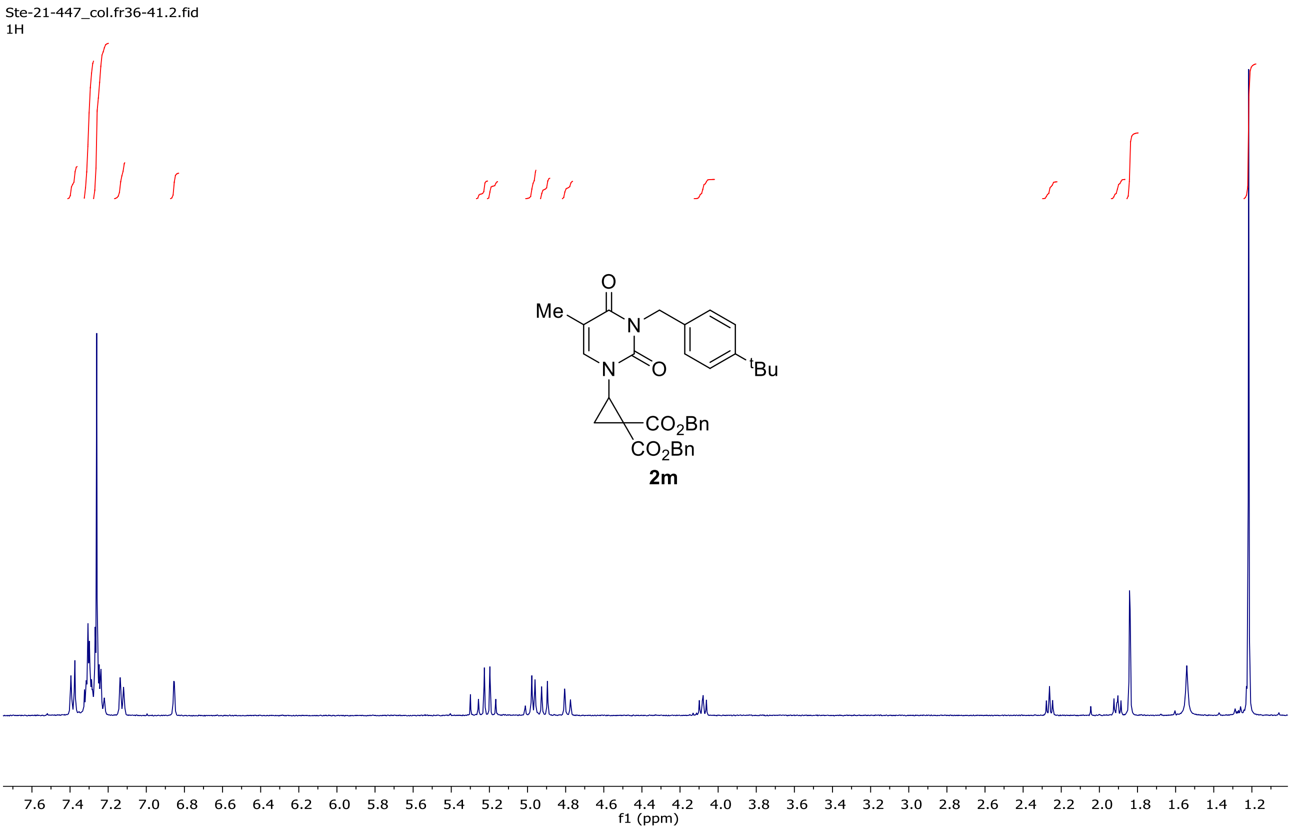

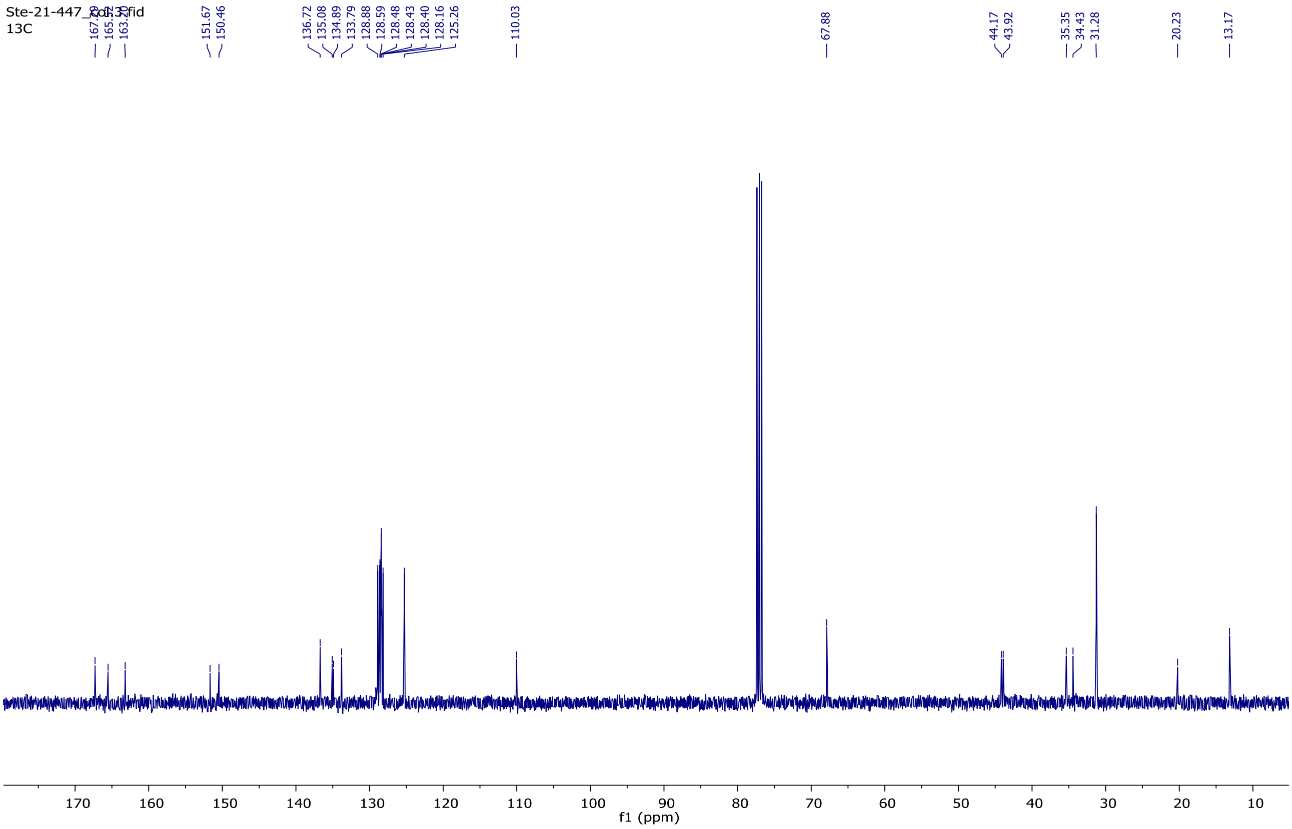

Dibenzyl 2-isopropoxy-5-(4-methoxyphenyl)-7-oxoazepane-3,3-dicarboxylate (3)

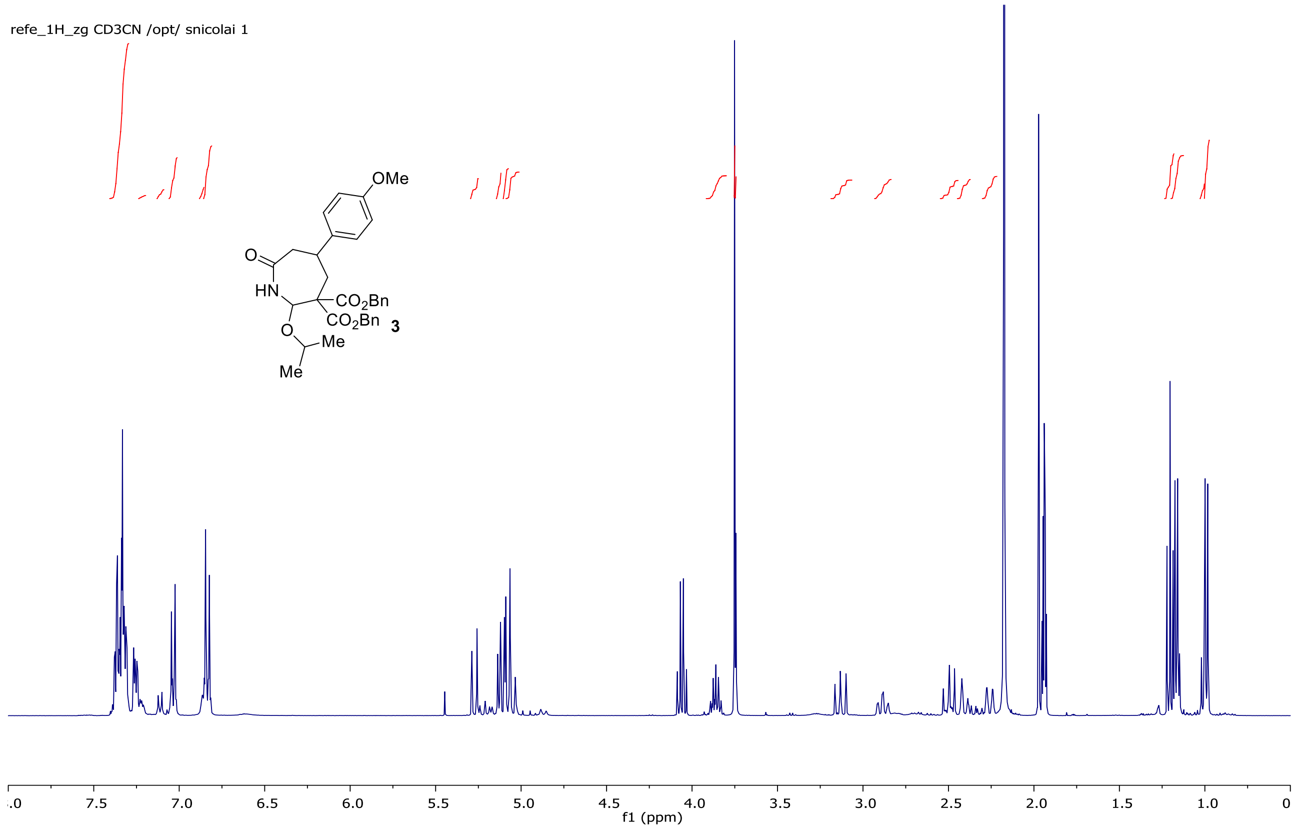

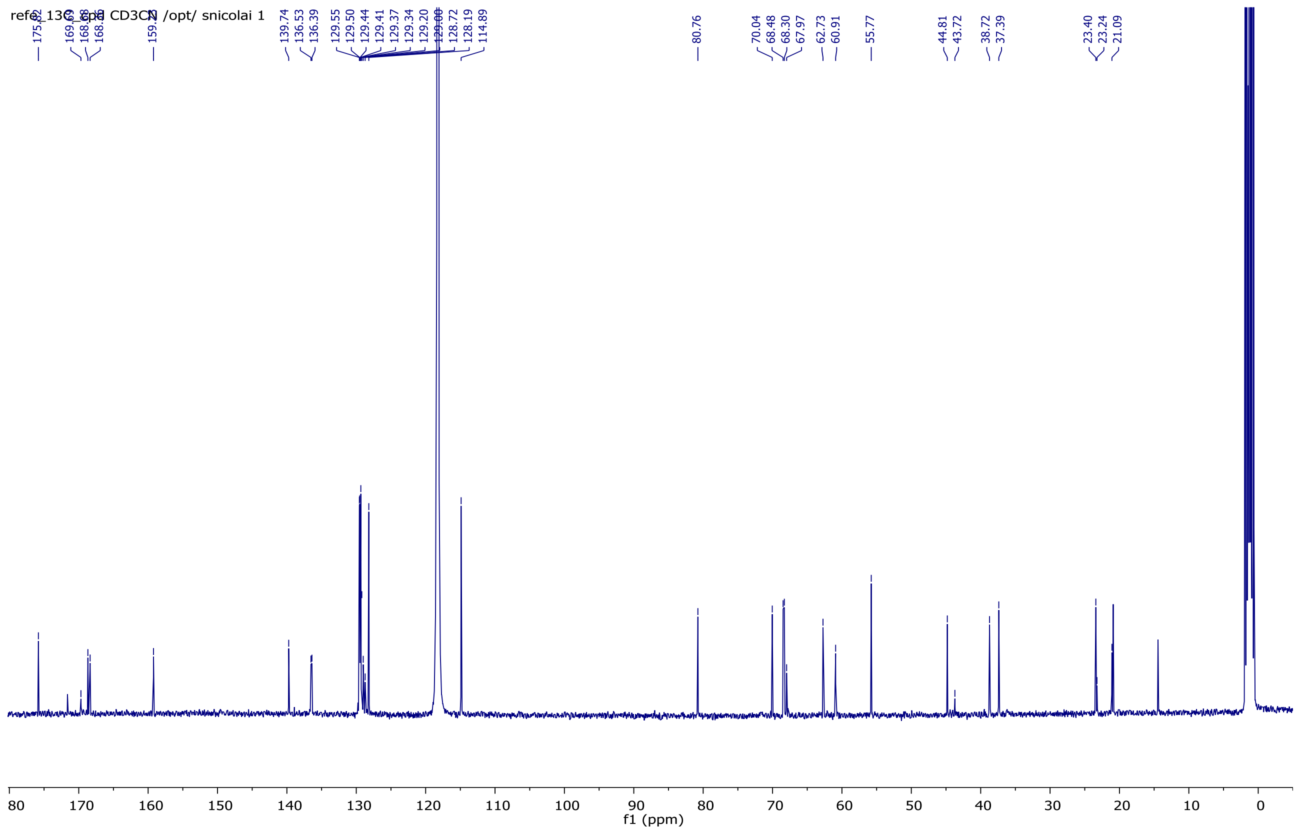

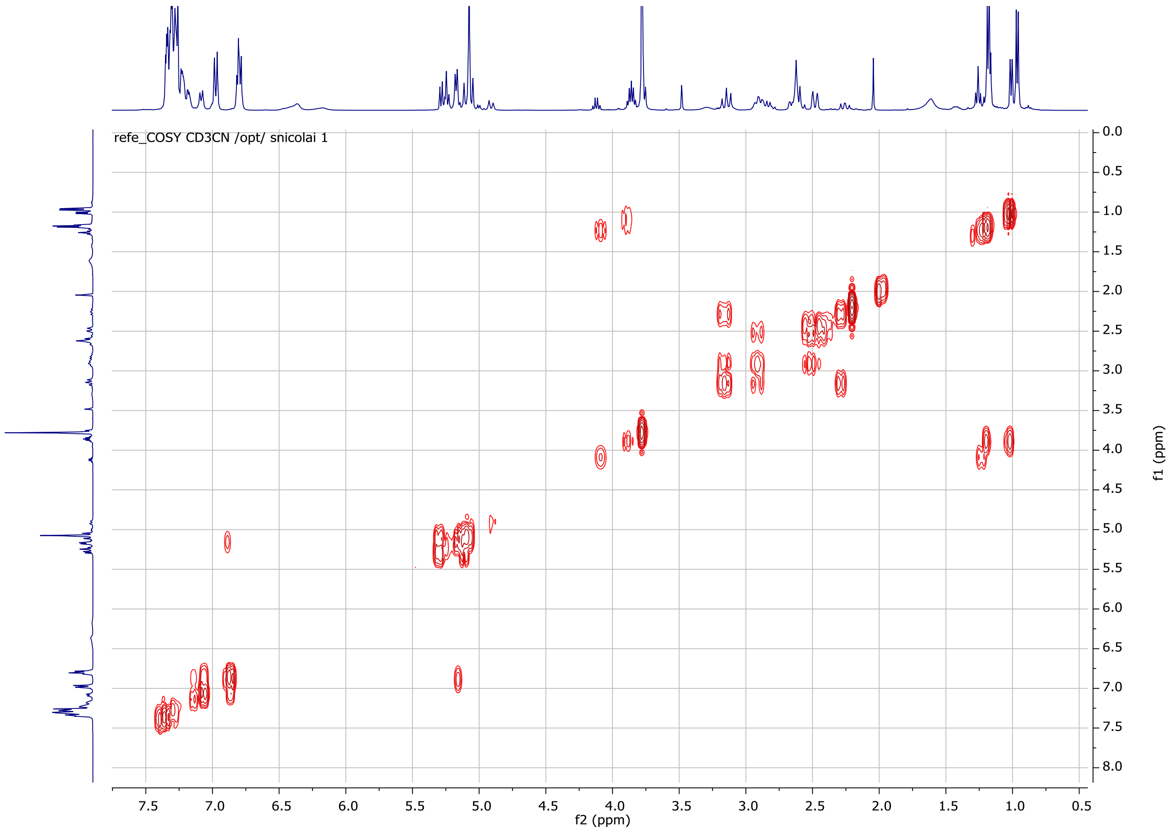
refe_COSY CD3CN /opt/ snicolai 1
f2 (ppm)
f1 (ppm)

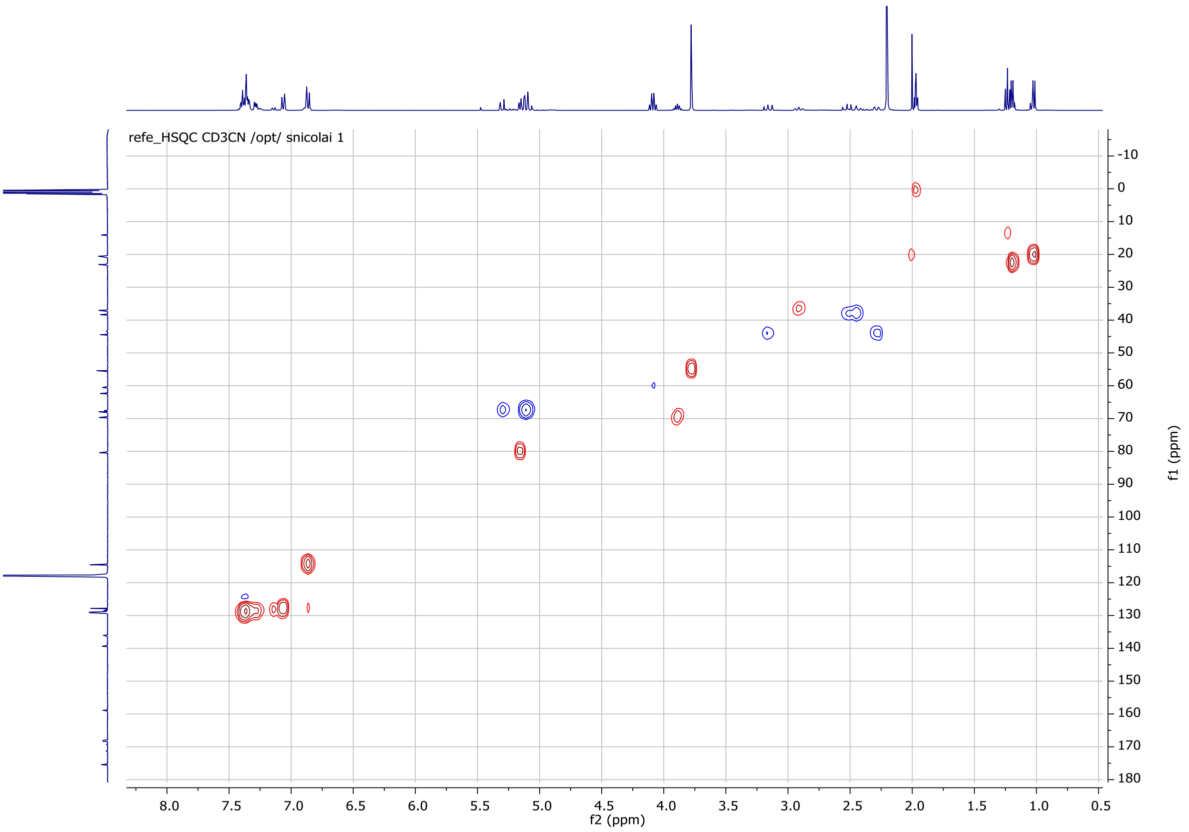
refe_HSQC CD3CN /opt/ snicolai 1
f2 (ppm)
f1 (ppm)

Di*iso*propyl 2,5-*trans*-5-(4-methoxyphenyl)-7-oxo-2-phenylazepane-3,3-dicarboxylate (5a.a'')

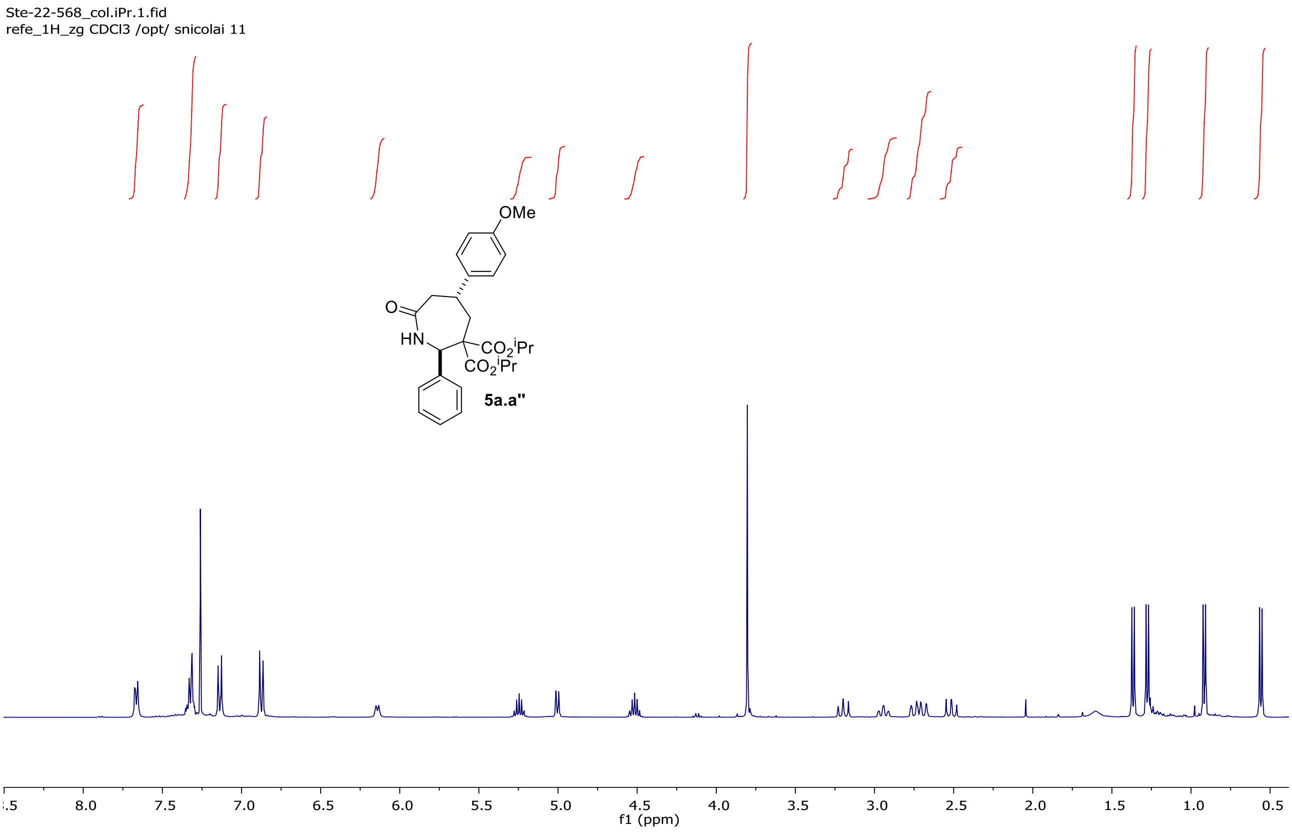

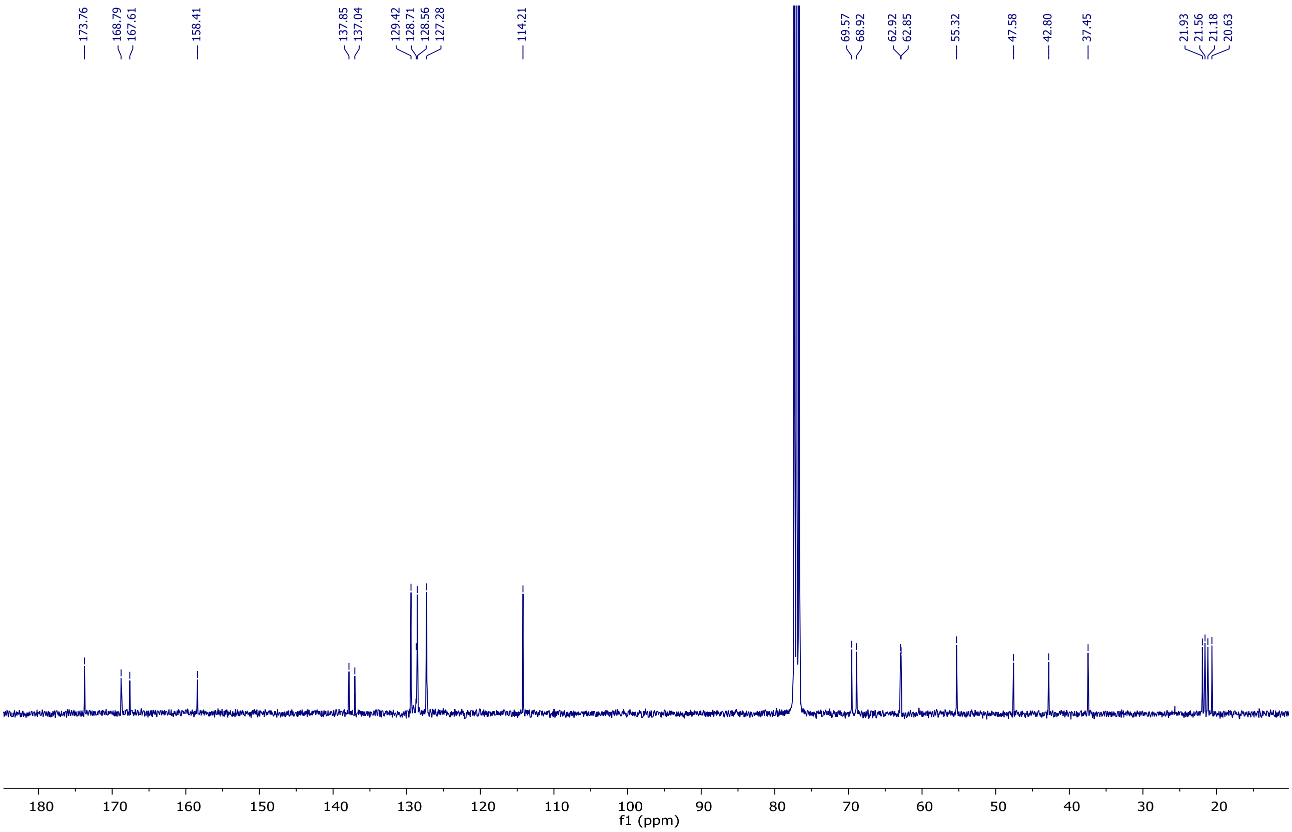

Dineopentyl 2,5-*trans*-5-(4-methoxyphenyl)-7-oxo-2-phenylazepane-3,3-dicarboxylate (5a.a''')

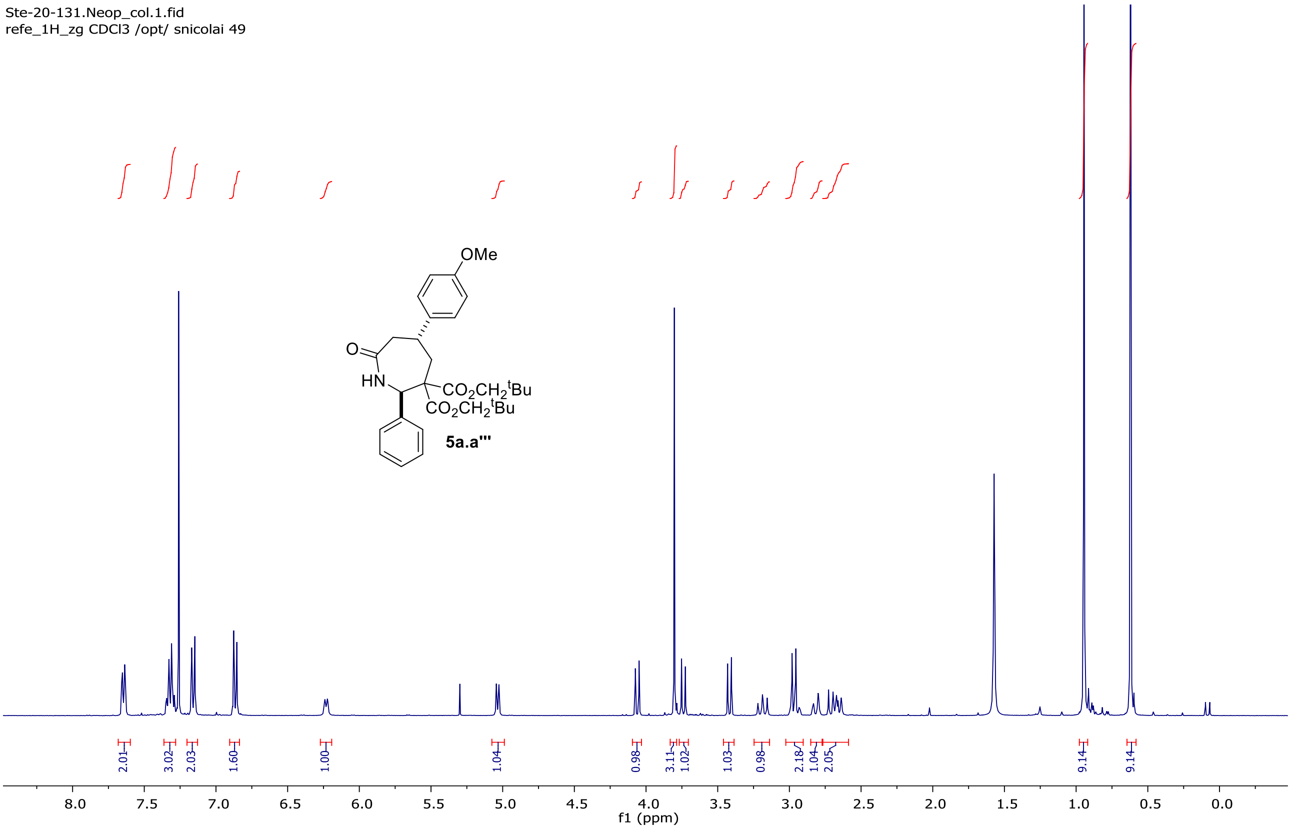

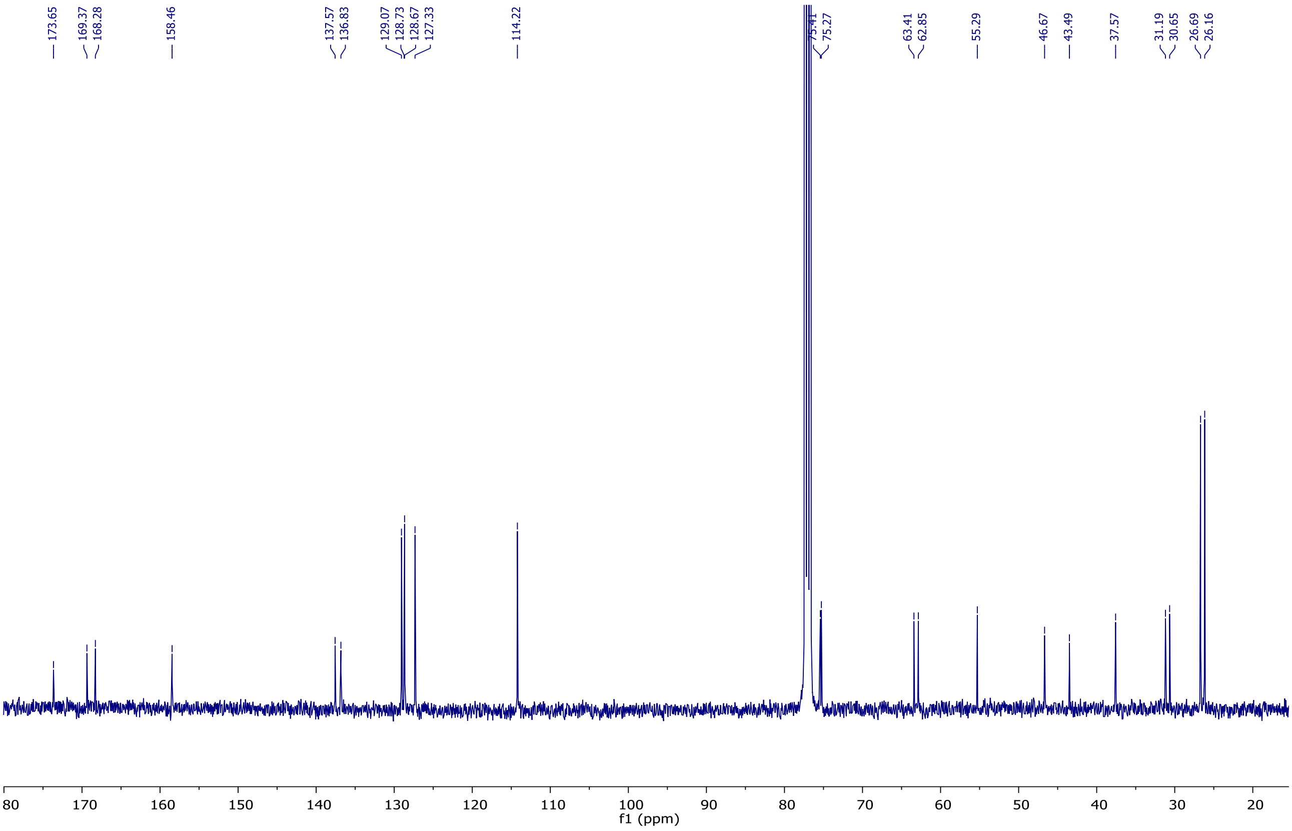

Dibenzyl 2,5-*trans*-5-(4-methoxyphenyl)-7-oxo-2-phenylazepane-3,3-dicarboxylate (5a.a)

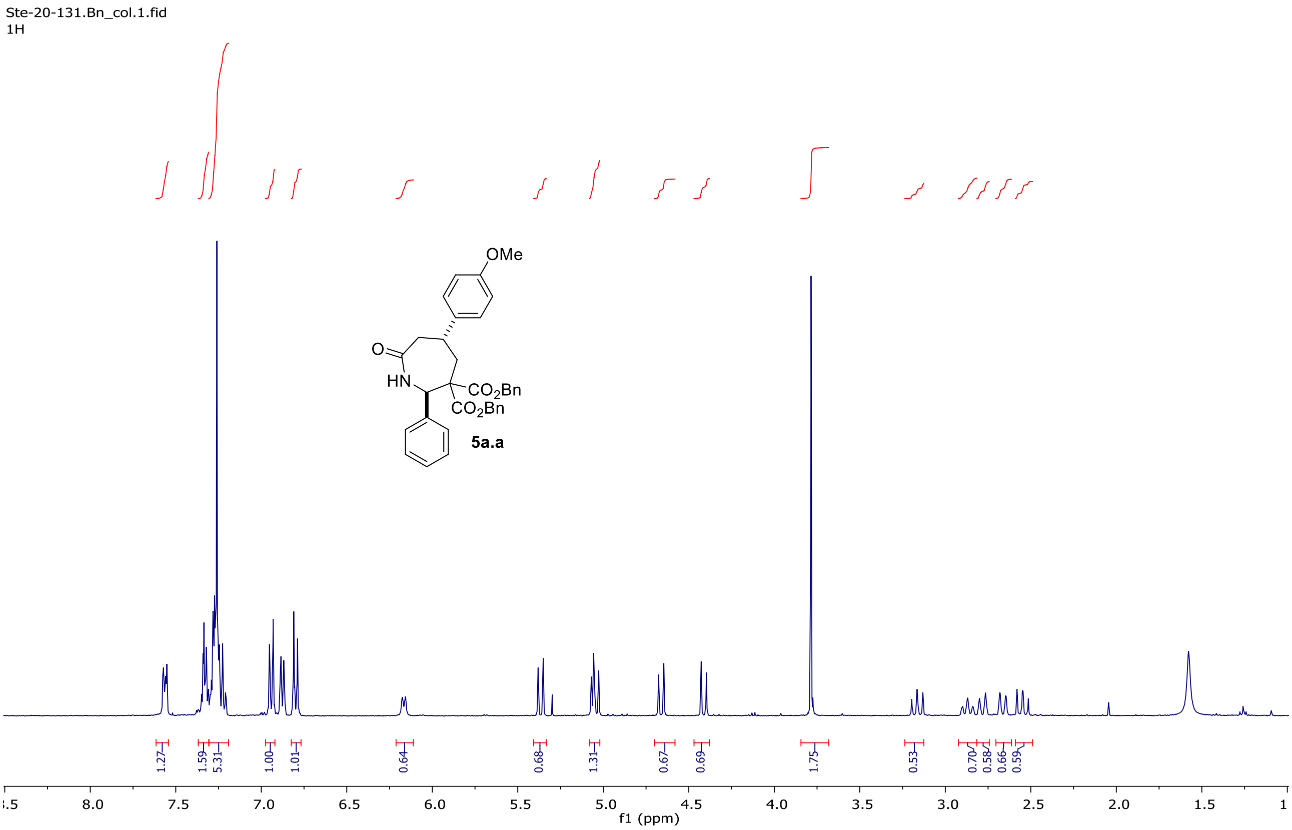

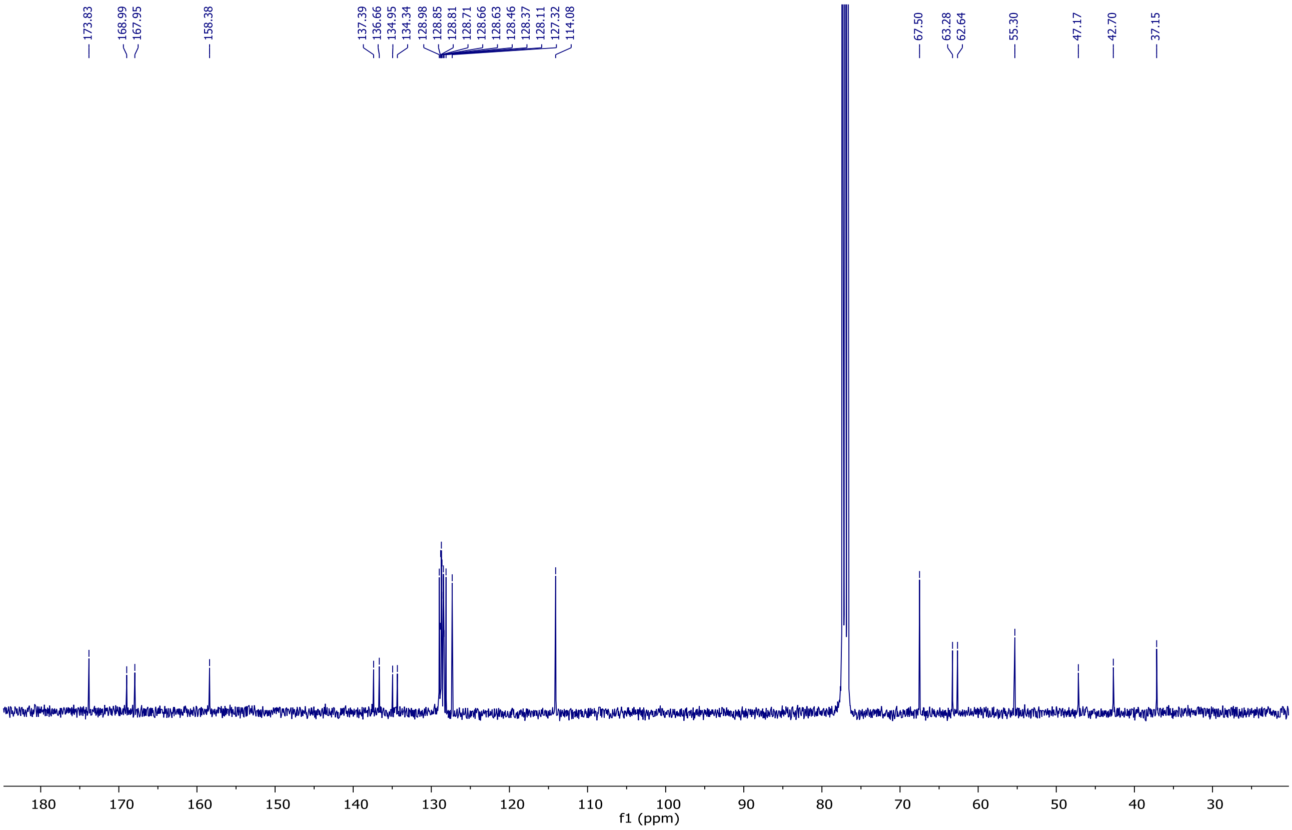

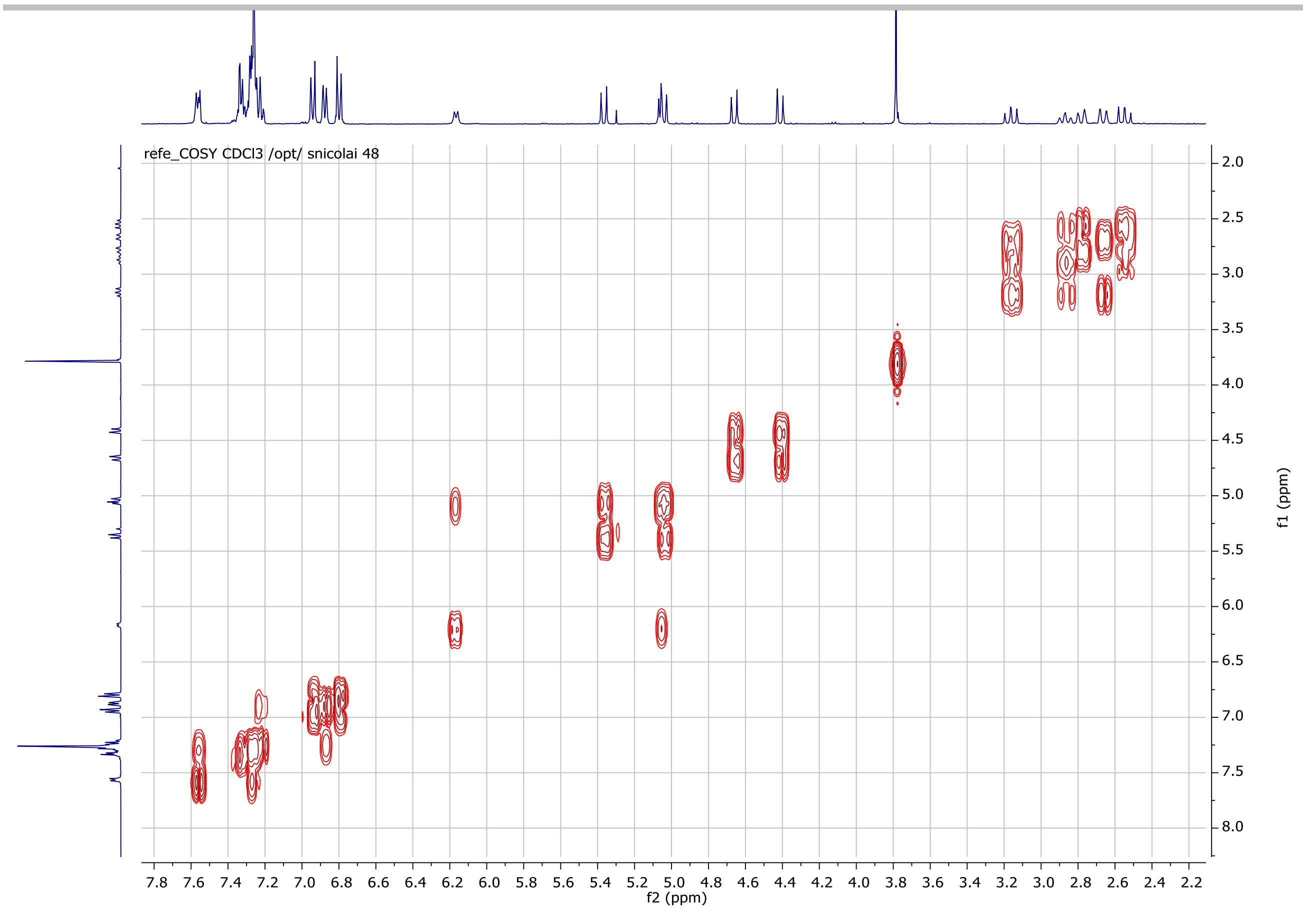
refe_COSY CDCl3 /opt/ snicolai 48
f2 (ppm)
f1 (ppm)

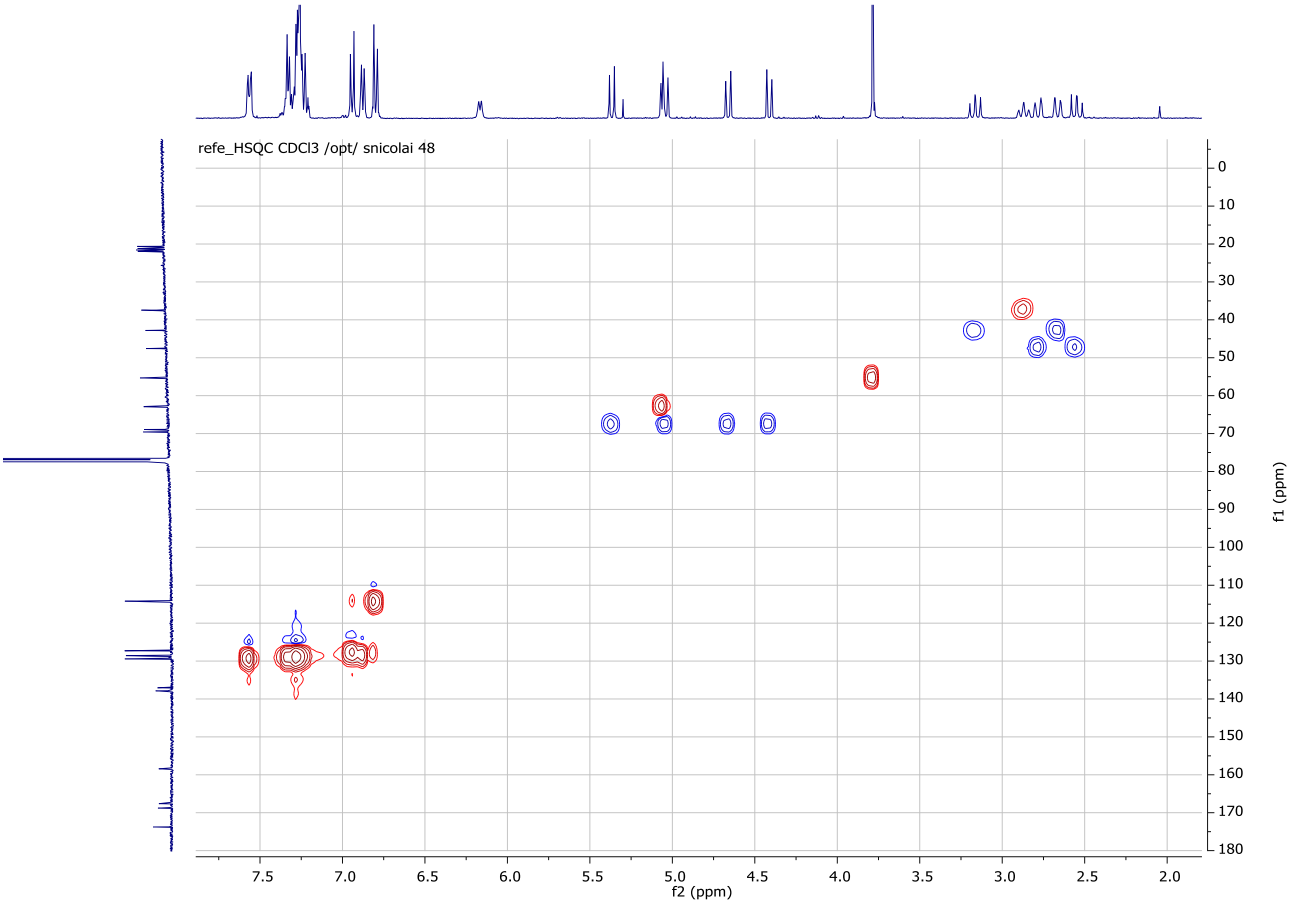
refe_HSQC CDCl3 /opt/ snicolai 48
f2 (ppm)
f1 (ppm)

Dibenzyl 2,5-*trans*-5-(3,4-dimethoxyphenyl)-7-oxo-2-phenylazepane-3,3-dicarboxylate (5a.b)

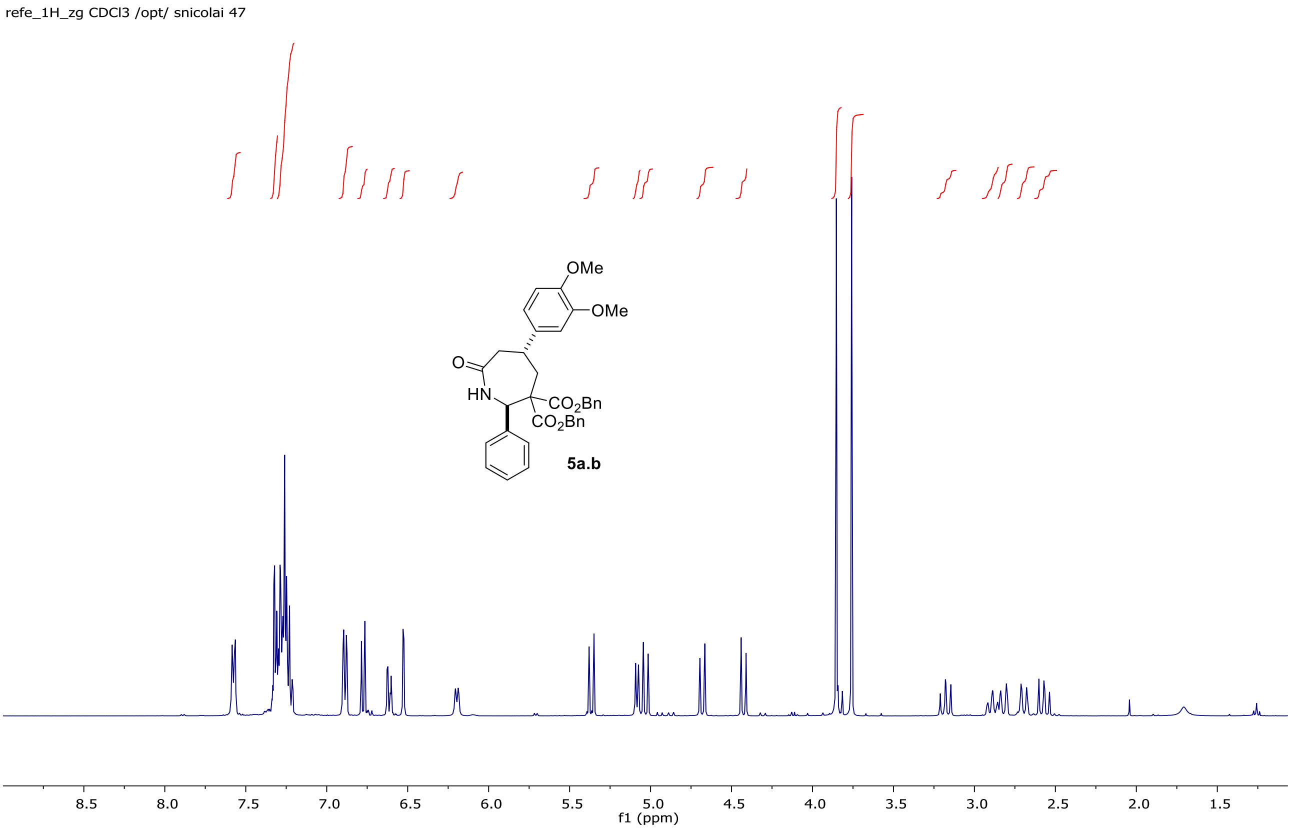

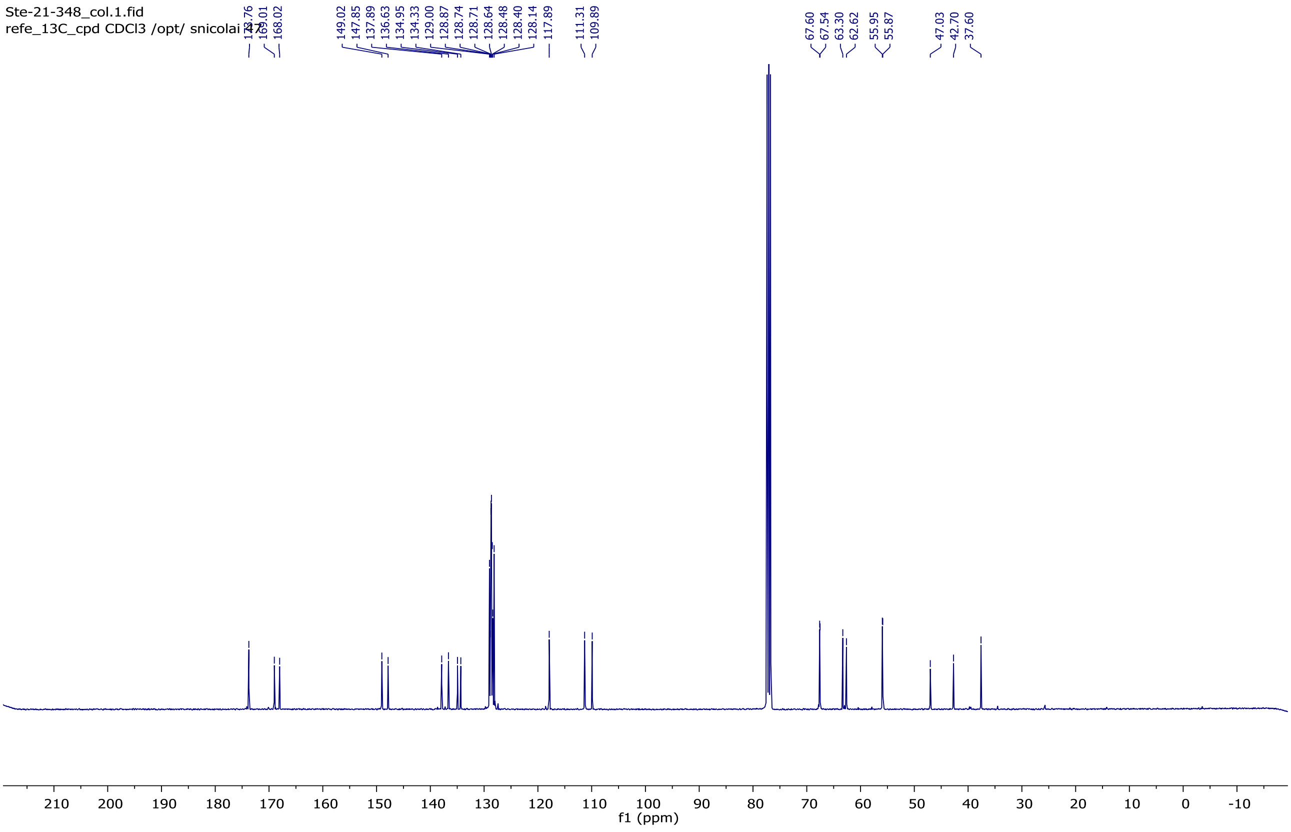

Dibenzyl 2,5-*trans*-5-(2,4-dimethoxyphenyl)-7-oxo-2-phenylazepane-3,3-dicarboxylate (5a.c)

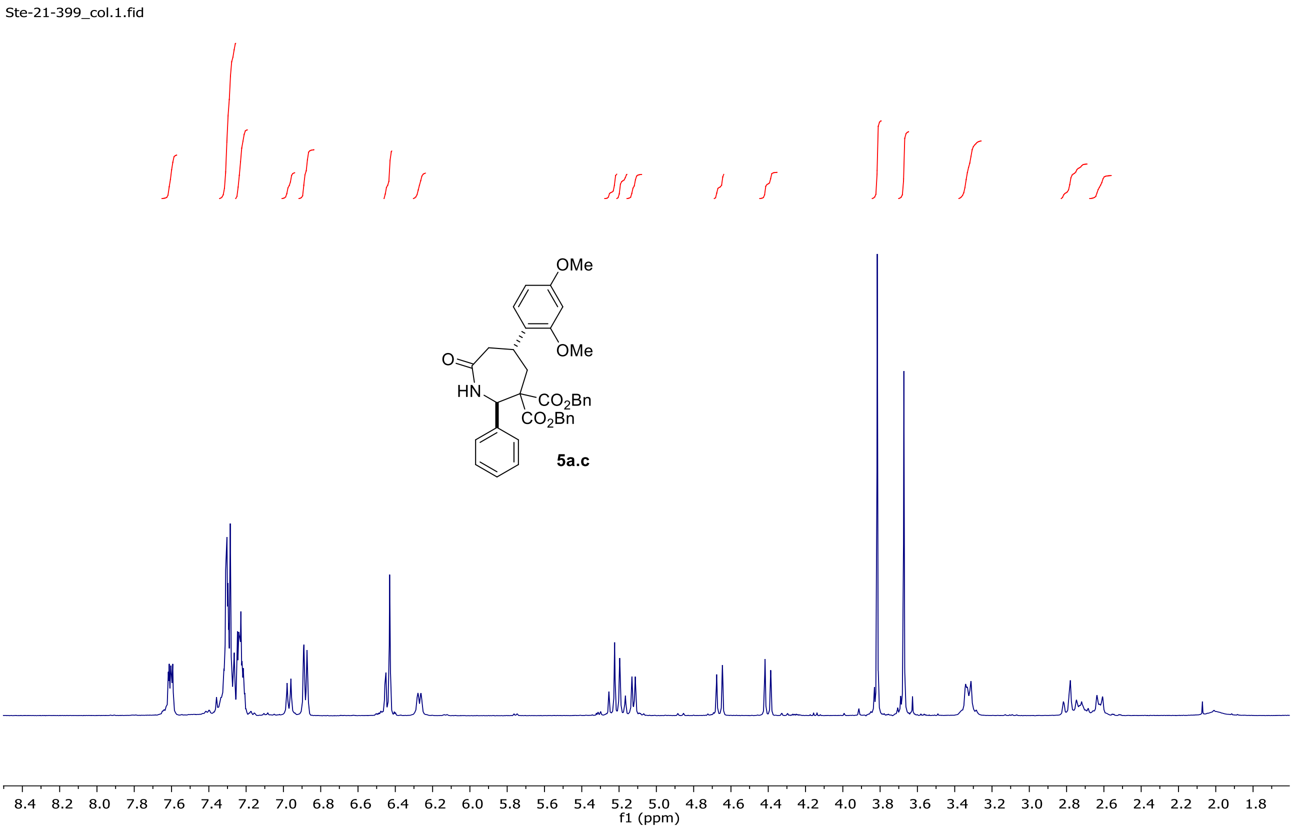

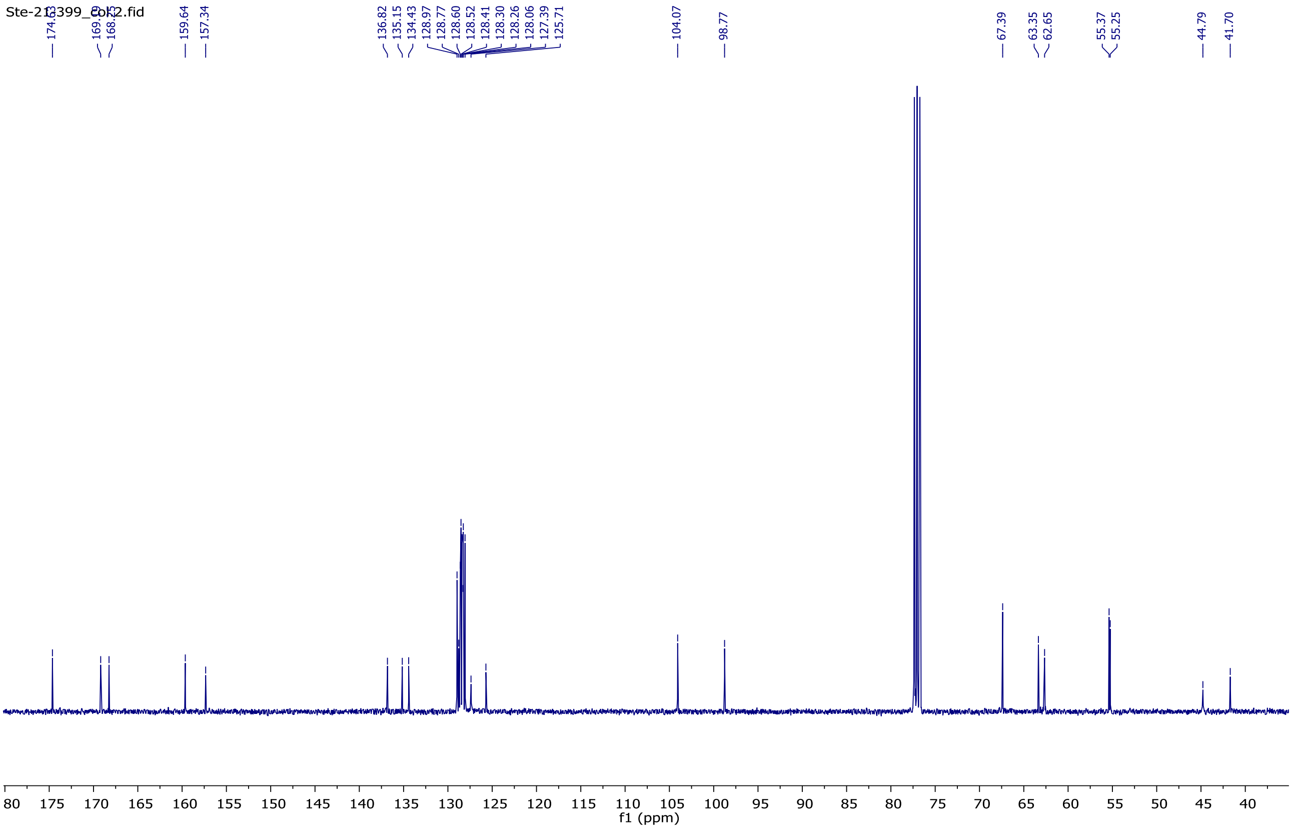

Dibenzyl 2,5-*trans*-5-(2-methoxyphenyl)-7-oxo-2-phenylazepane-3,3-dicarboxylate (5a.d)

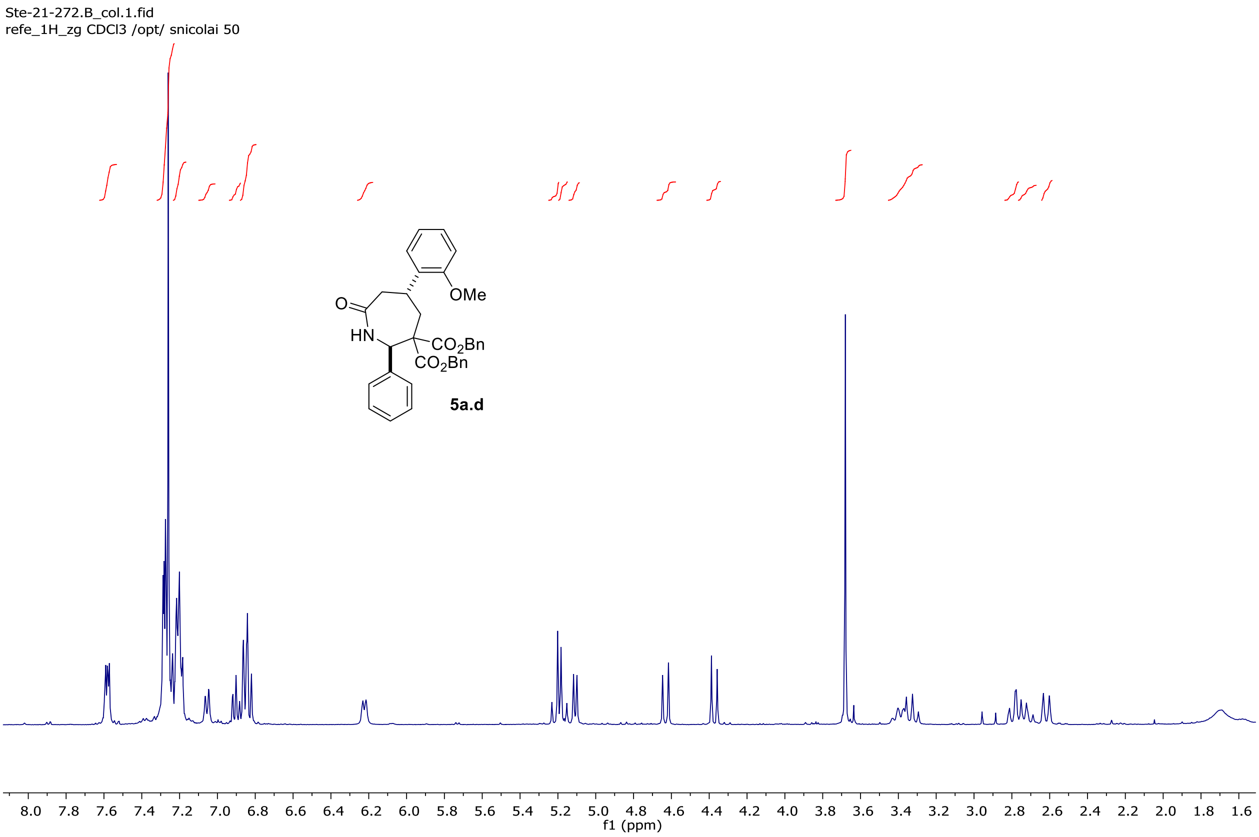

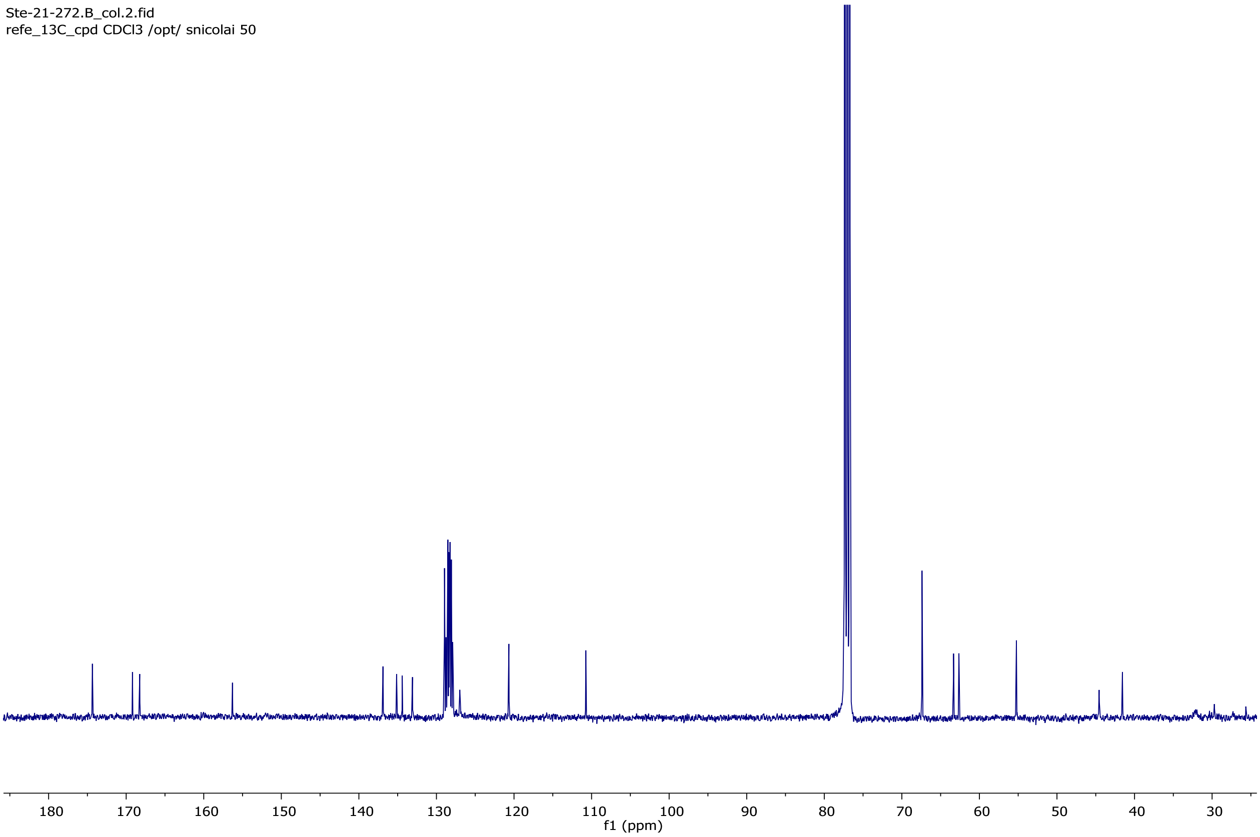

Dibenzyl 2,5-*trans*-5-(3-iodo-4-methoxyphenyl)-7-oxo-2-phenylazepane-3,3-dicarboxylate (5a.e)

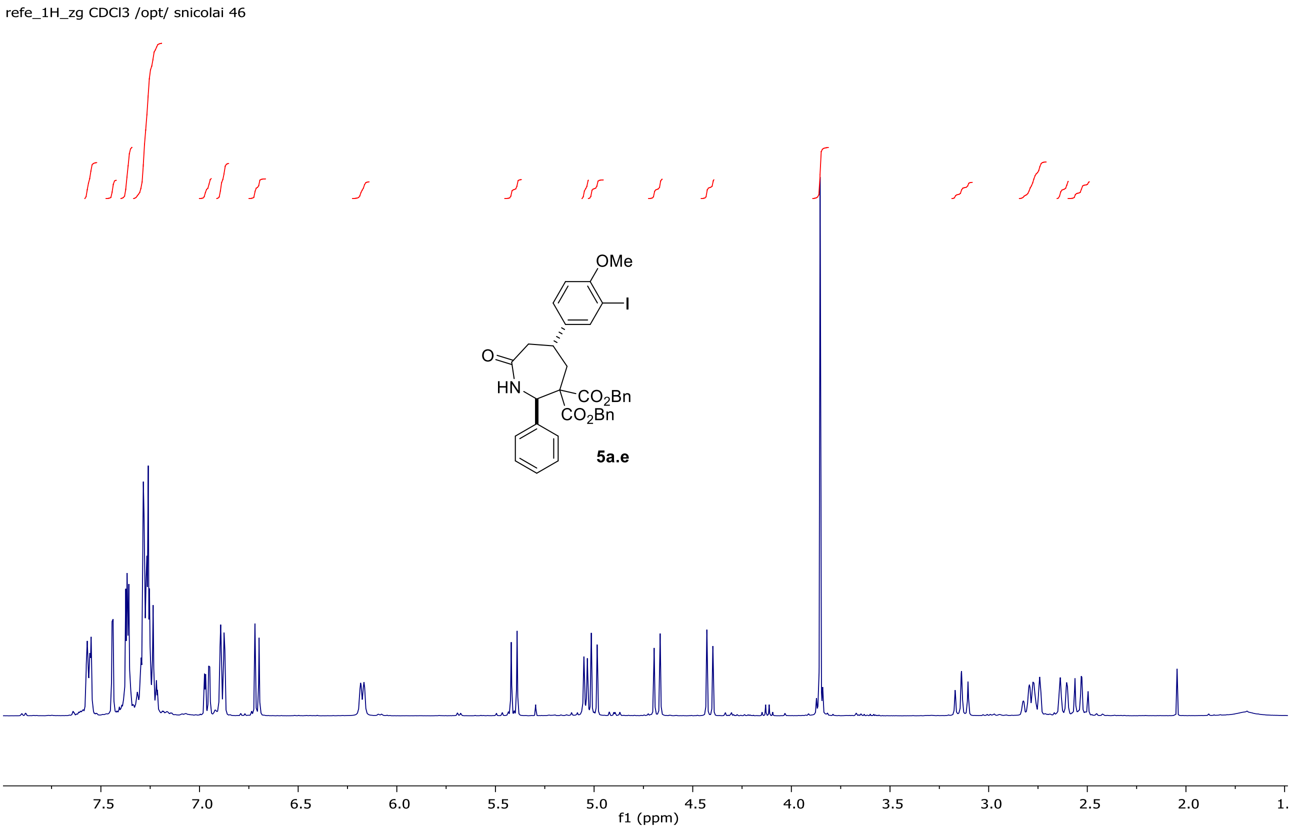

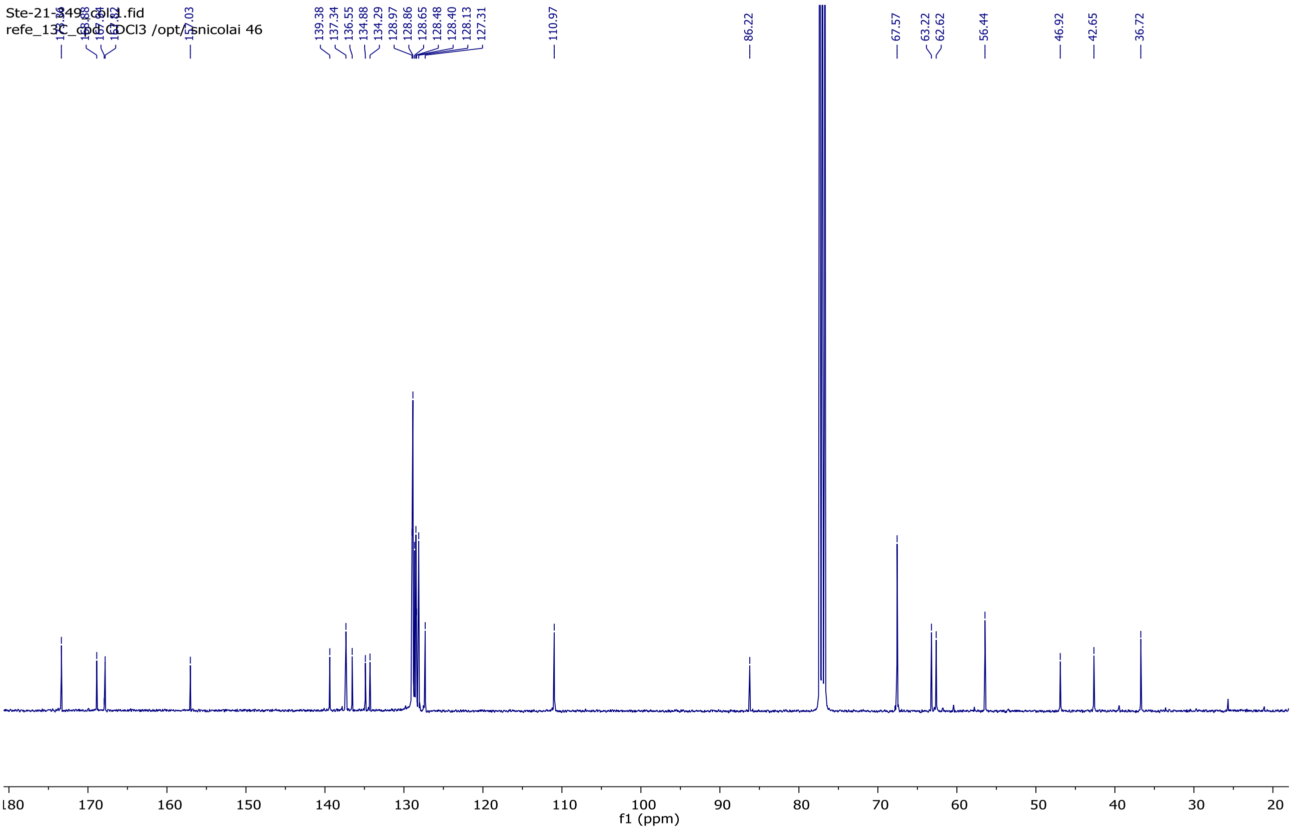

Dibenzyl 2,5-*trans*-7-oxo-2-phenyl-5-(*p*-tolyl)azepane-3,3-dicarboxylate (5a.f)

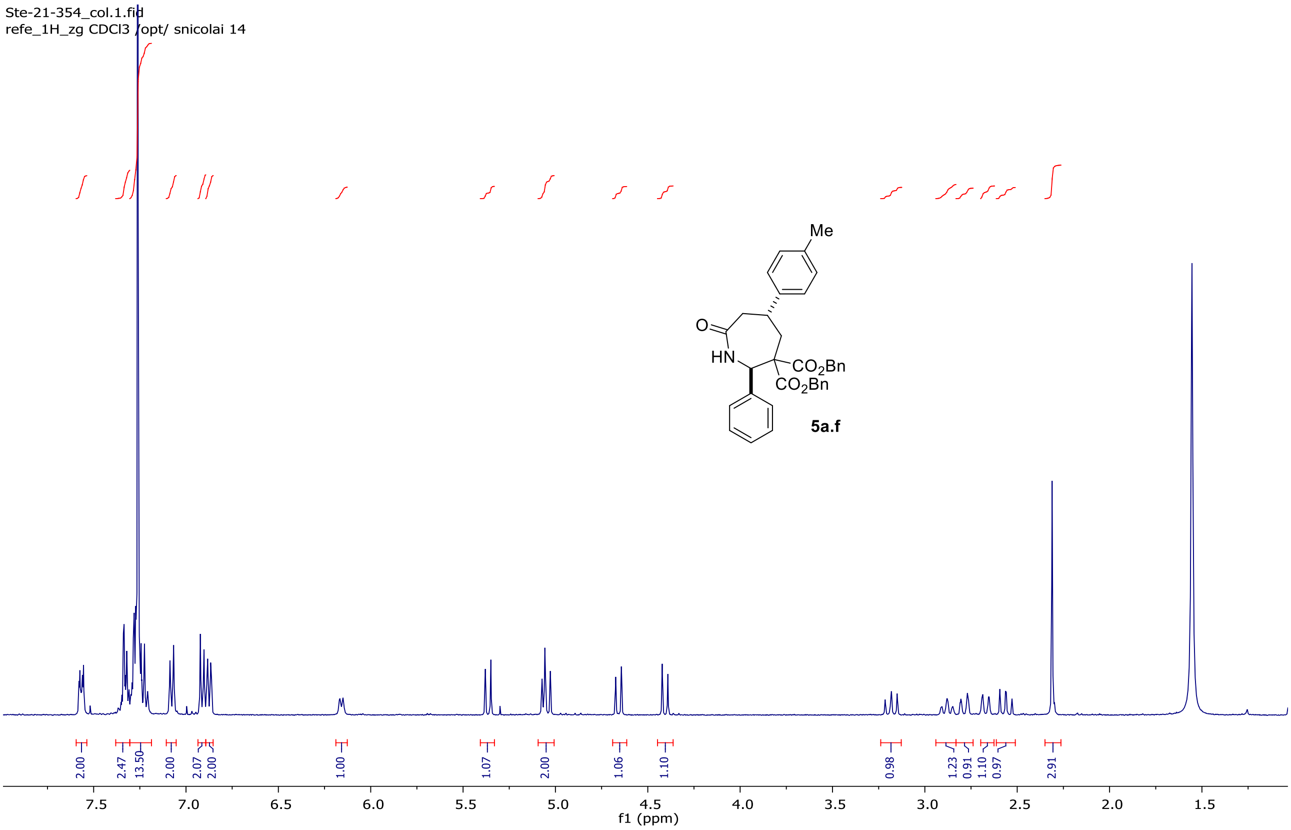

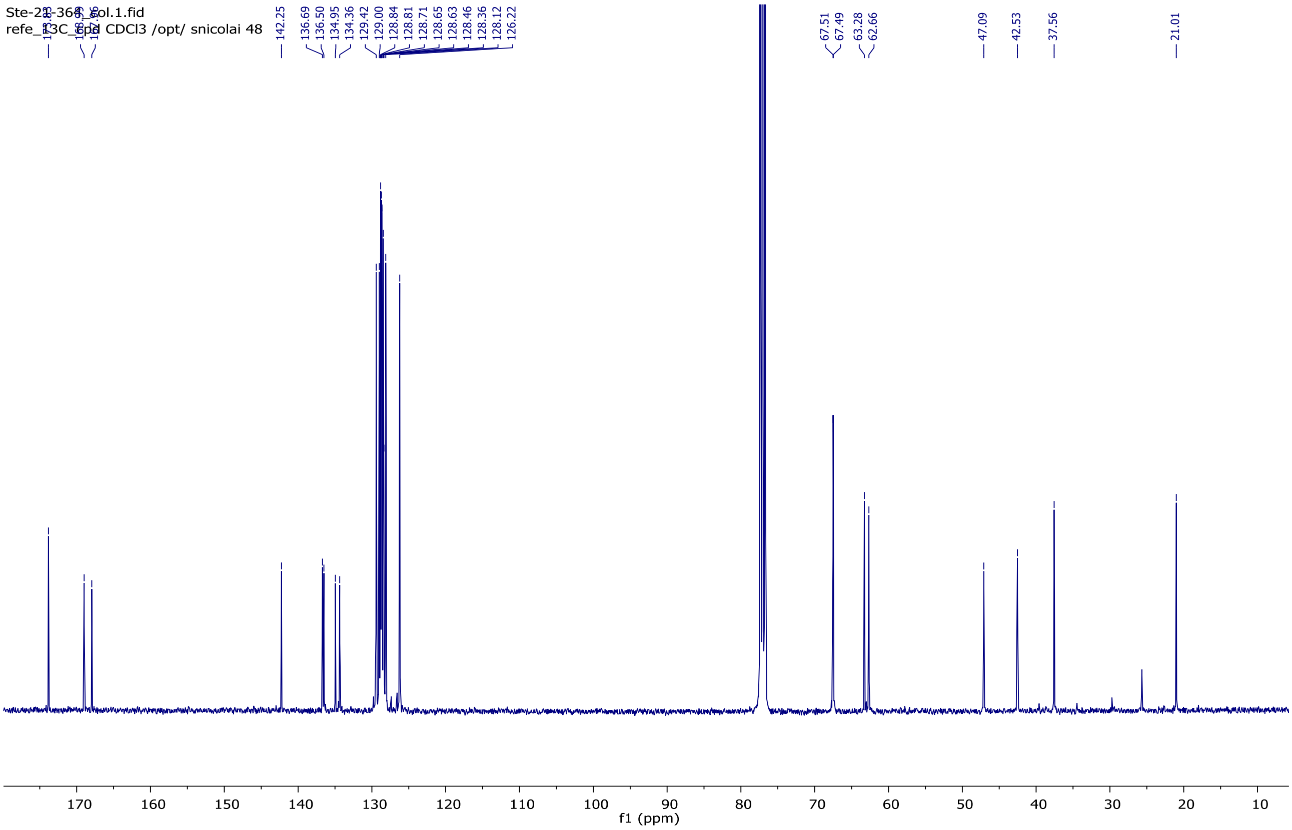

Dibenzyl 2,5-*trans*-7-oxo-2-phenyl-5-(1-tosyl-1H-indol-3-yl)azepane-3,3-dicarboxylate (5a.g)

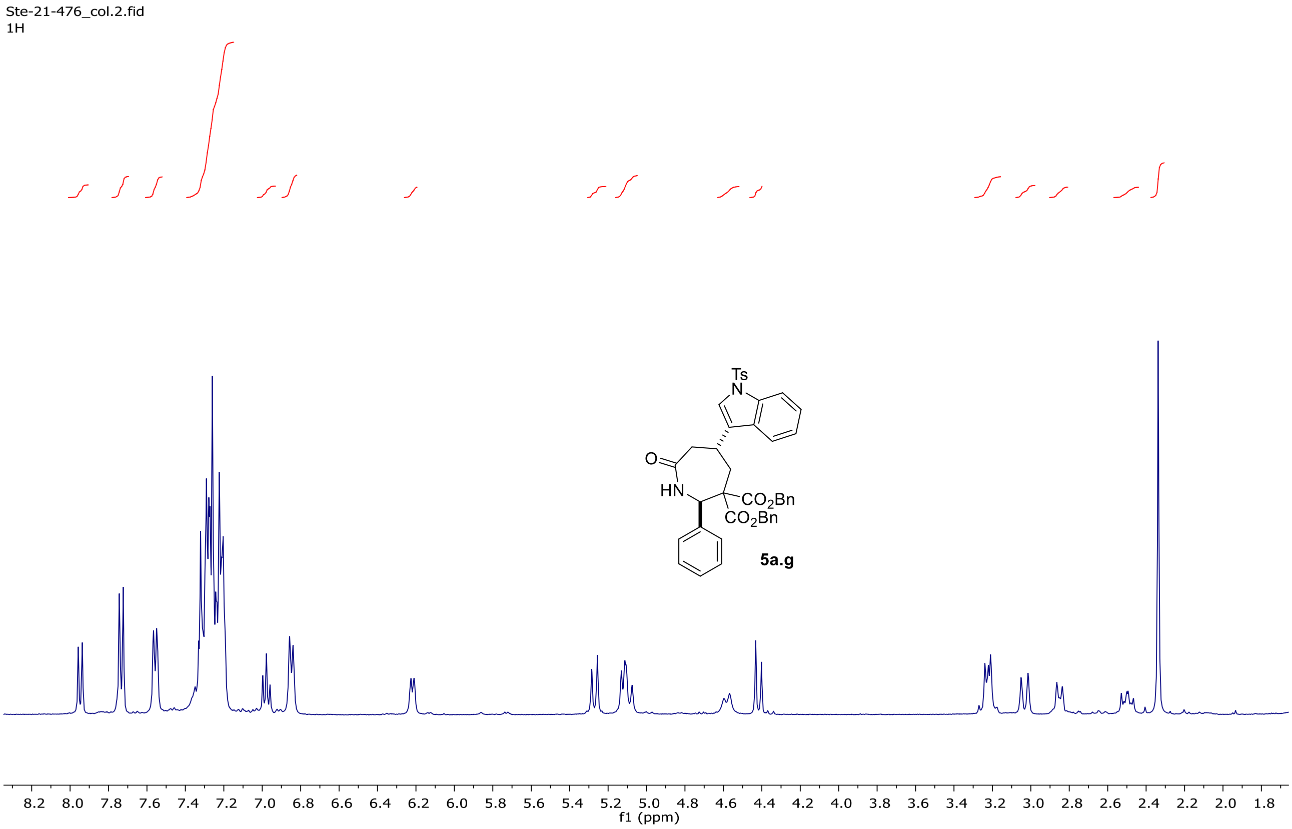

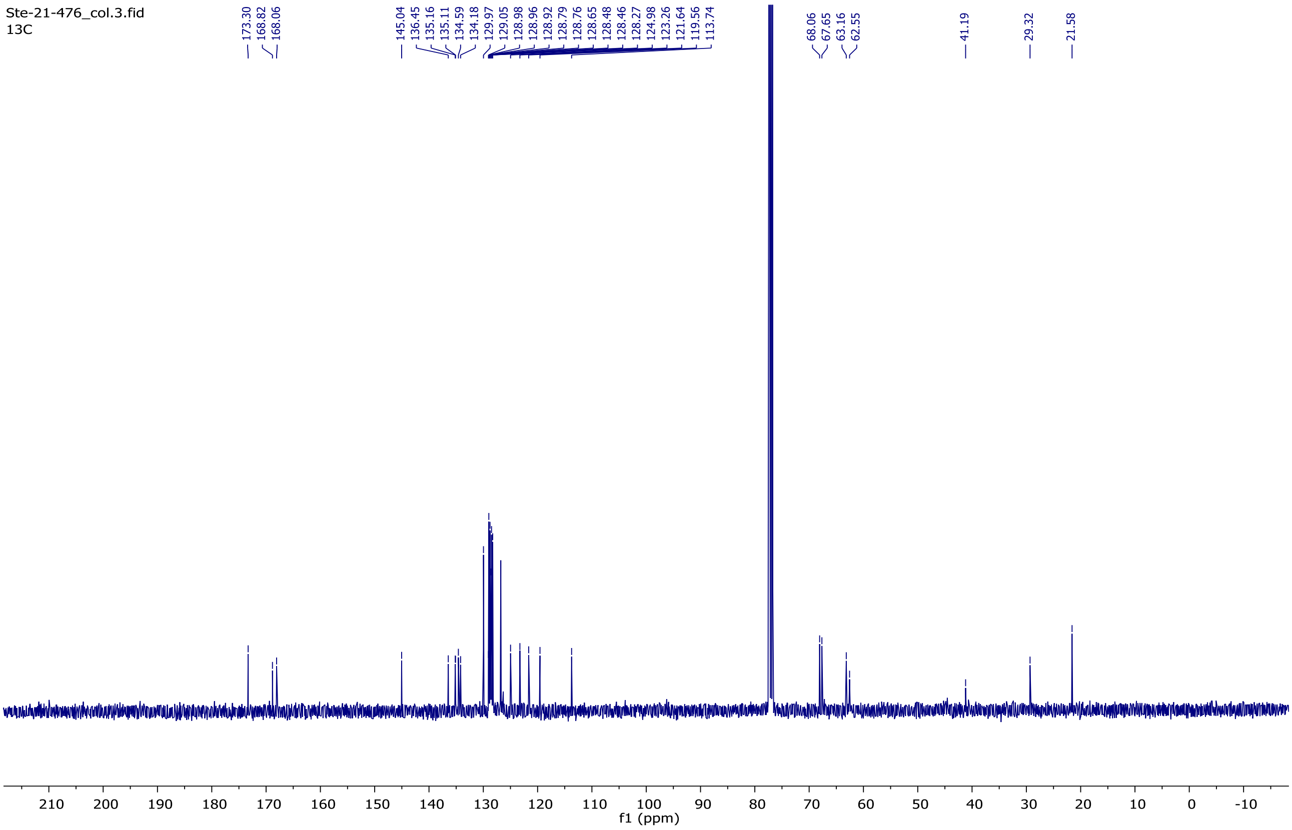

Dibenzyl 2,5-trans-5-(benzo[b]thiophen-2-yl)-7-oxo-2-phenylazepane-3,3-dicarboxylate (5a.h)

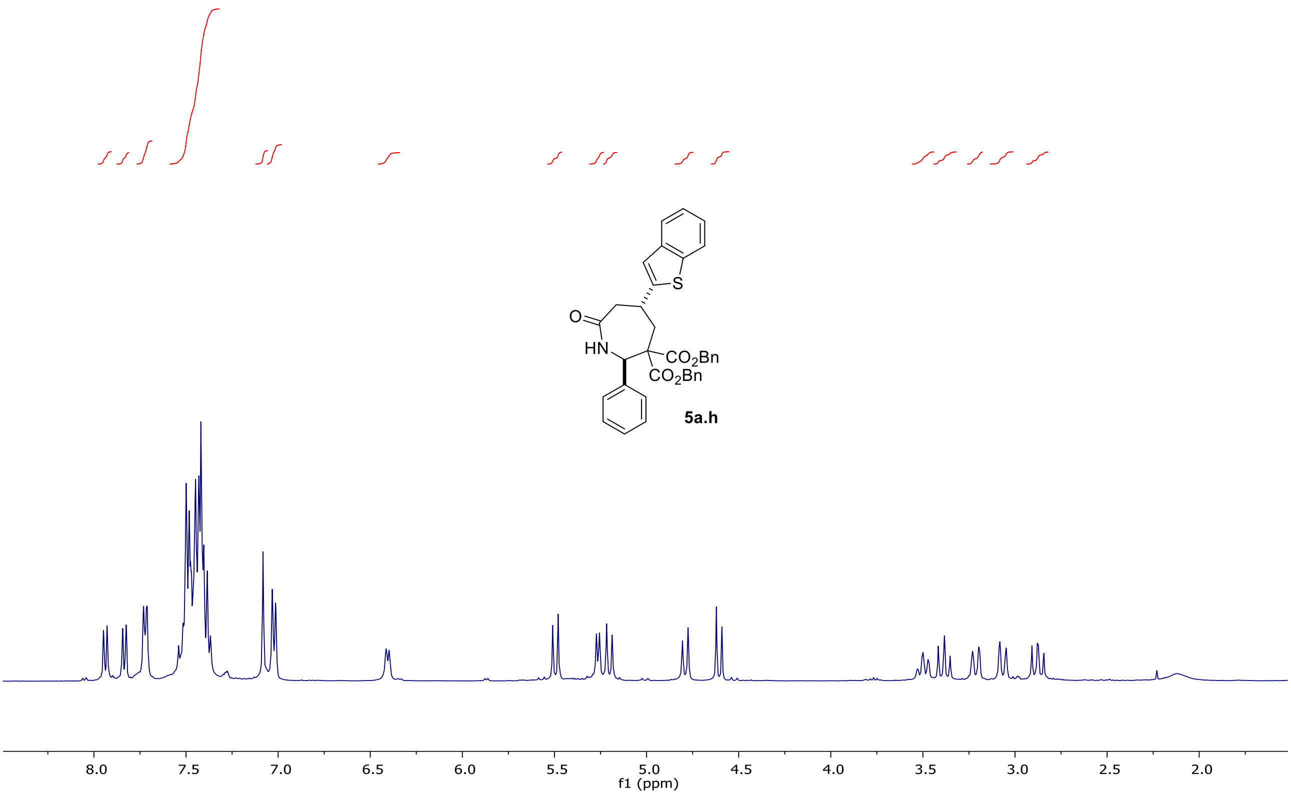

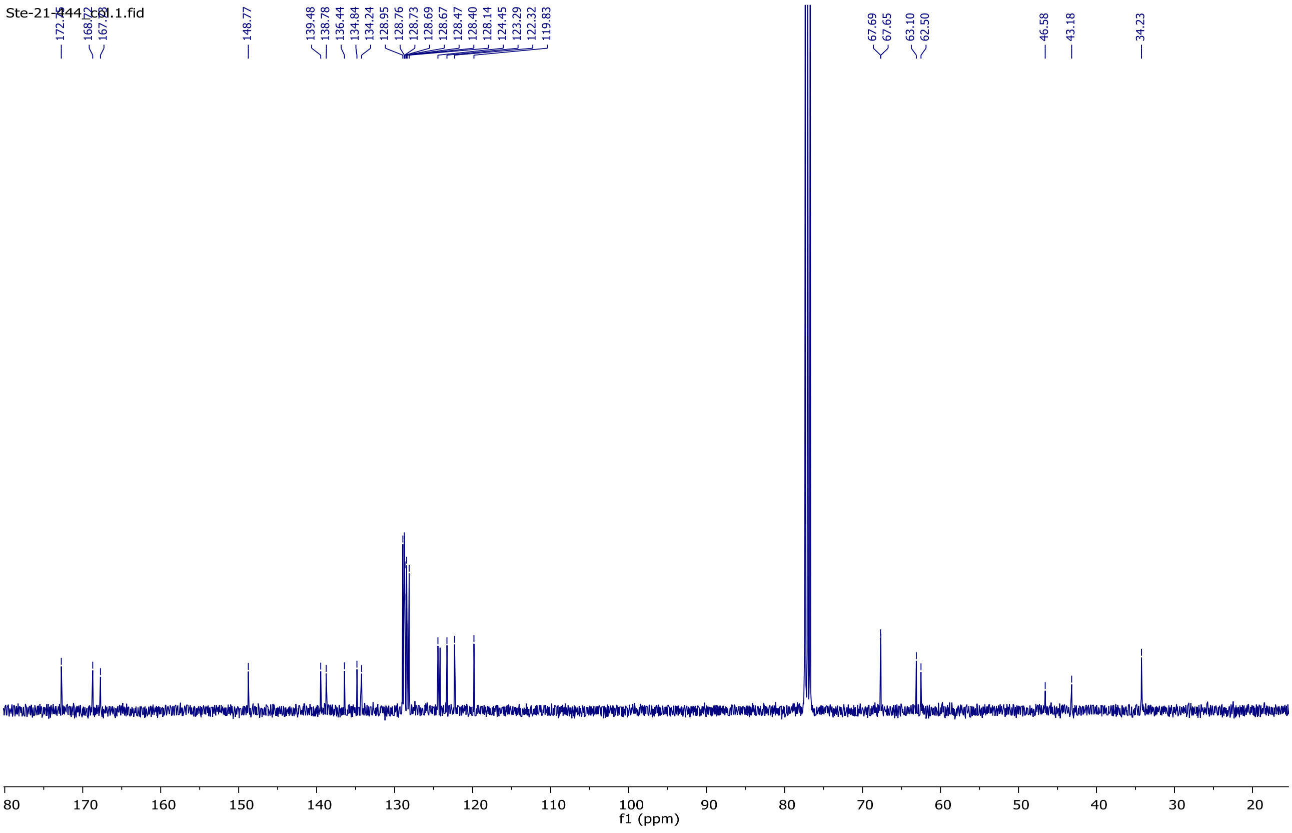

Dibenzyl 2,5-*trans* -7-oxo-2-phenyl-5-(1-phenyl-1*H*-pyrazol-4-yl)azepane-3,3-dicarboxylate (5a.i)

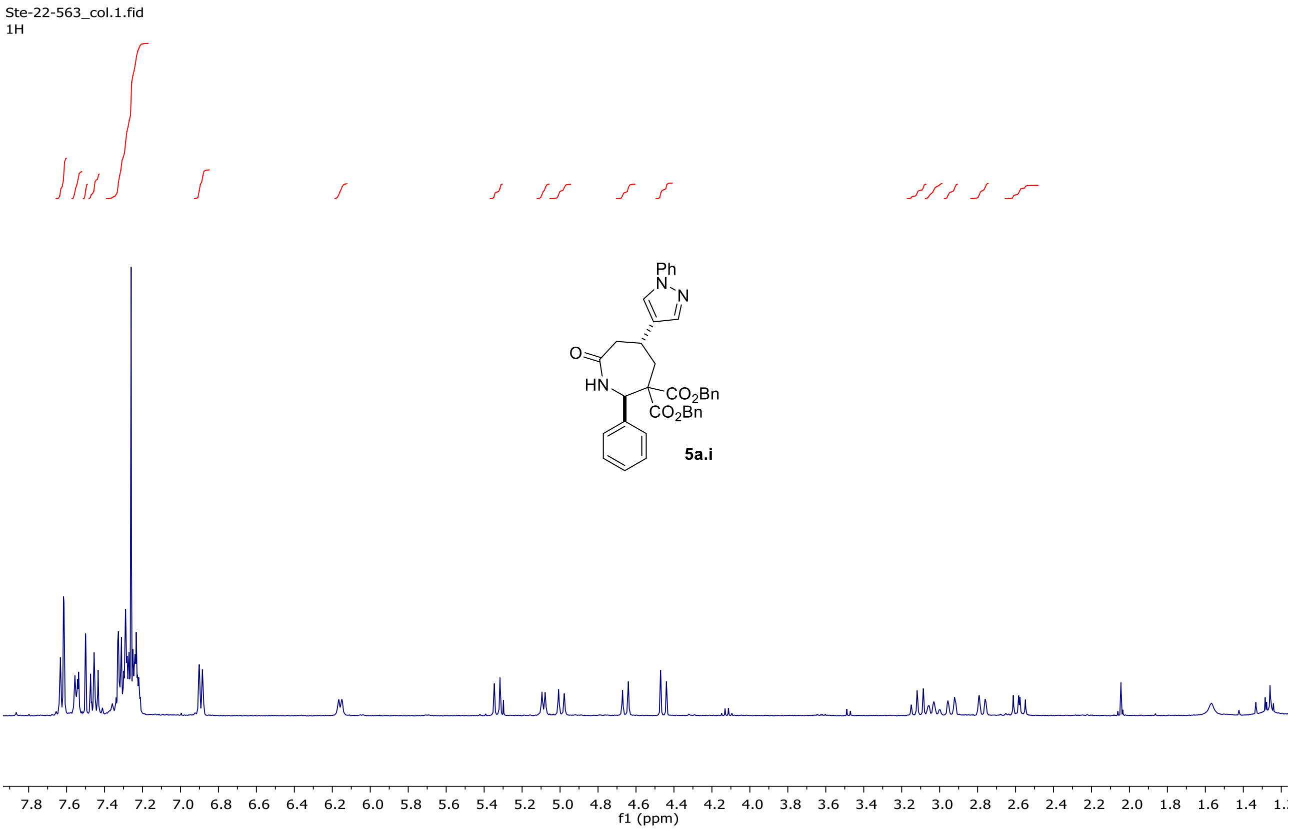

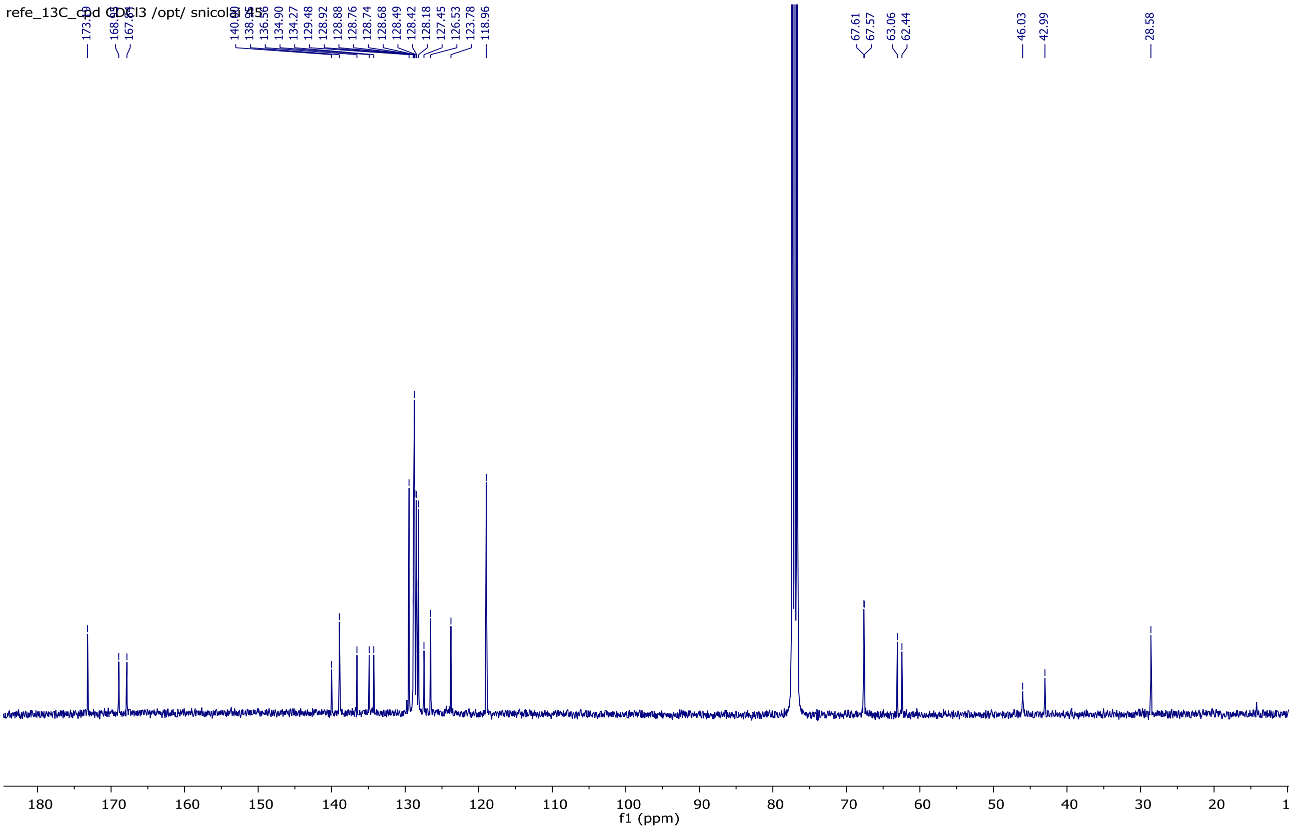

Dibenzyl 2,5-*trans*-7-oxo-2-phenyl-5-((E)-1-phenylprop-1-en-2-yl)azepane-3,3-dicarboxylate (5a.j)

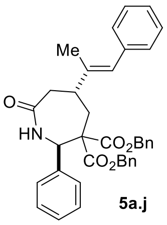

5a.j

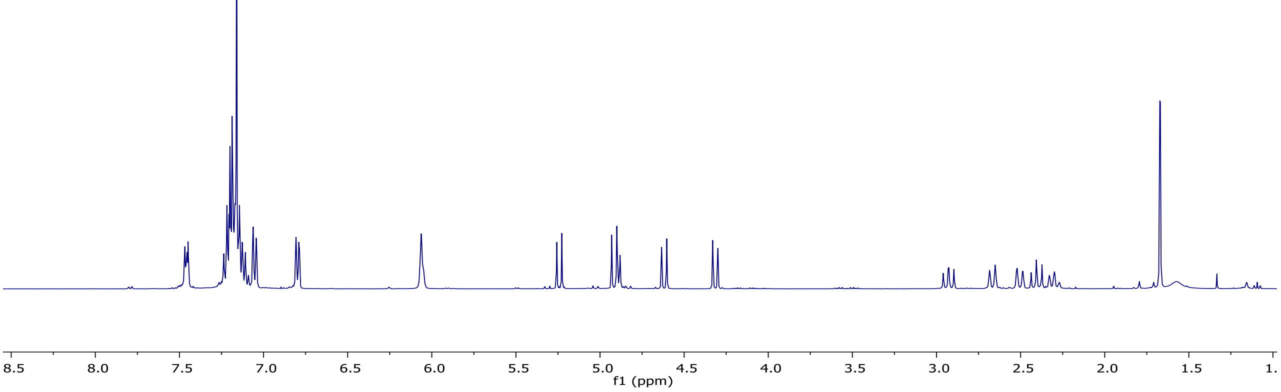

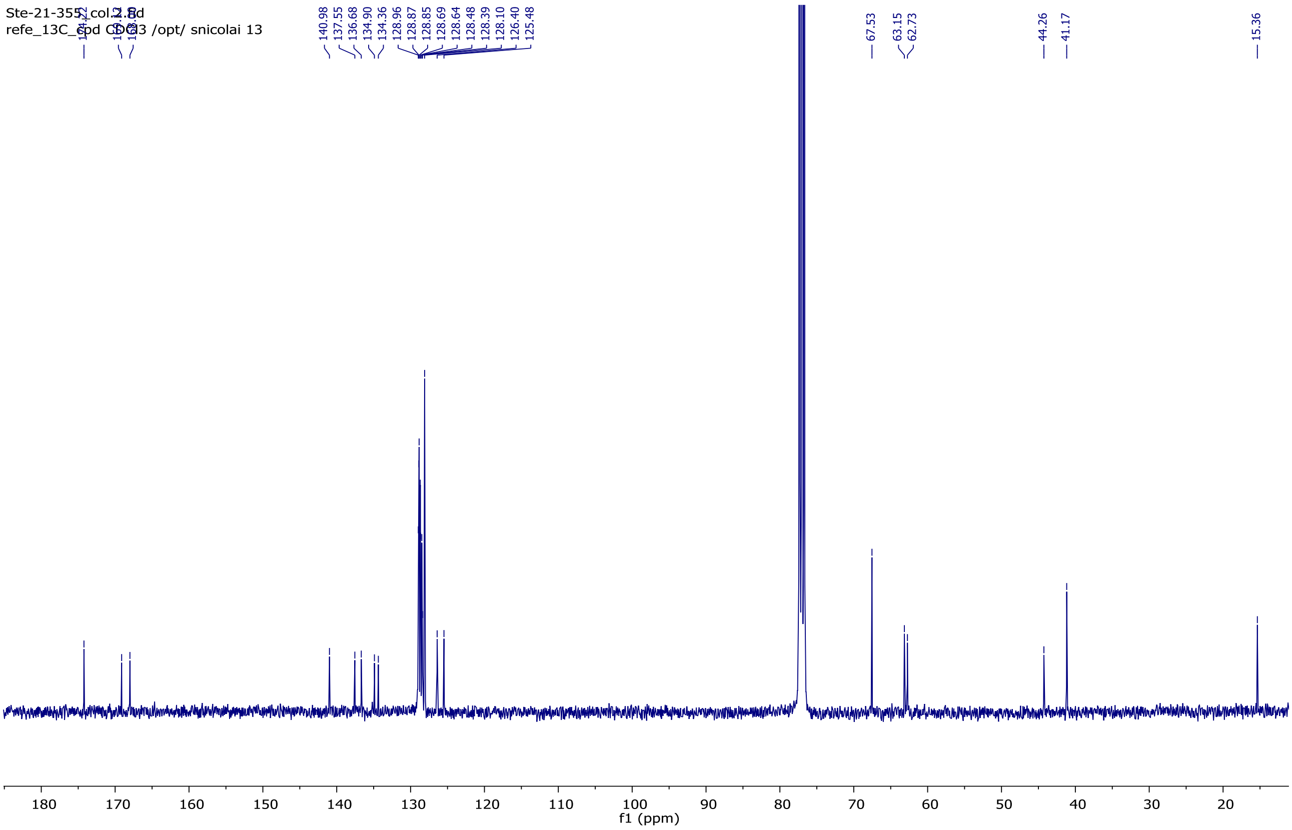

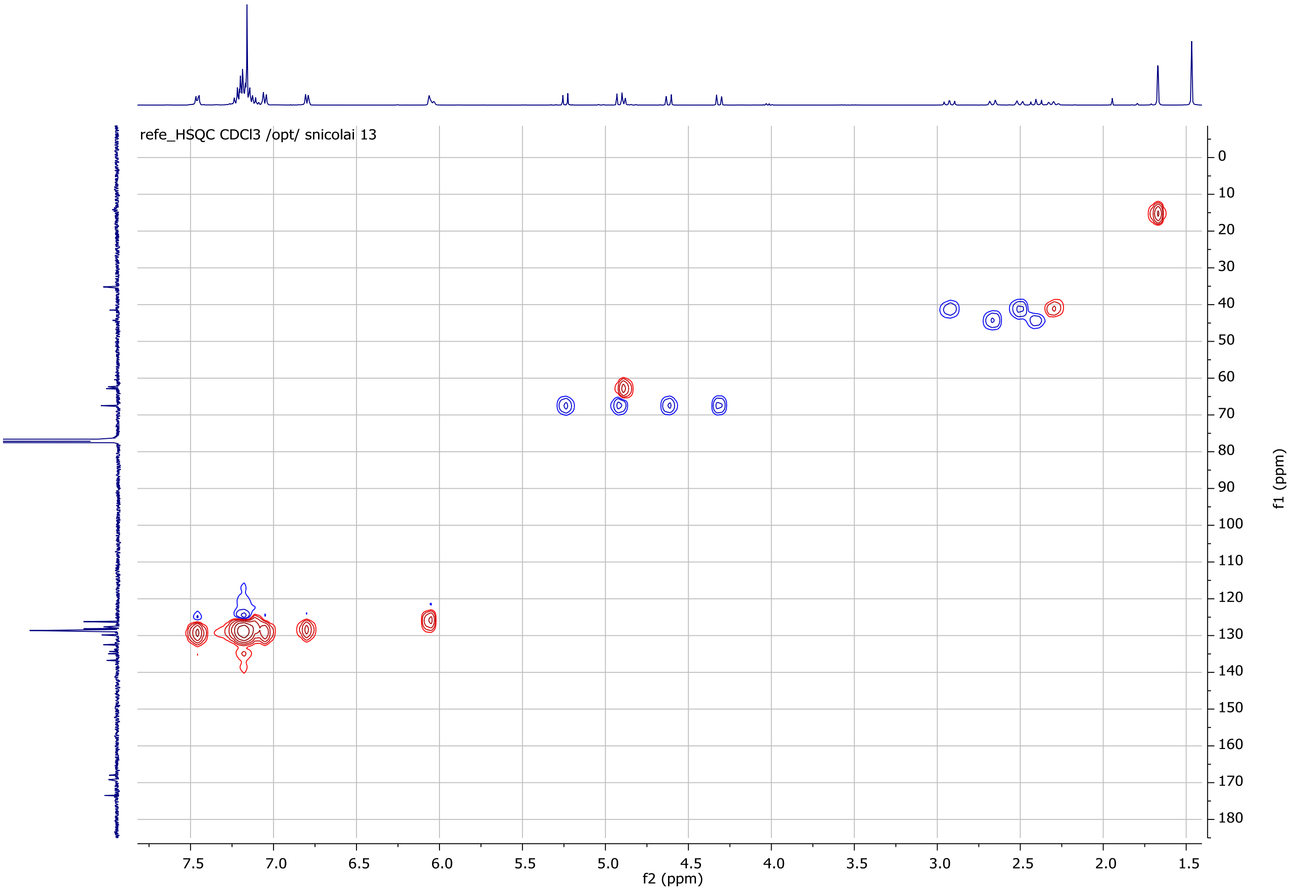
refe_HSQC CDCl3 /opt/ snicolai 13
f2 (ppm)
f1 (ppm)

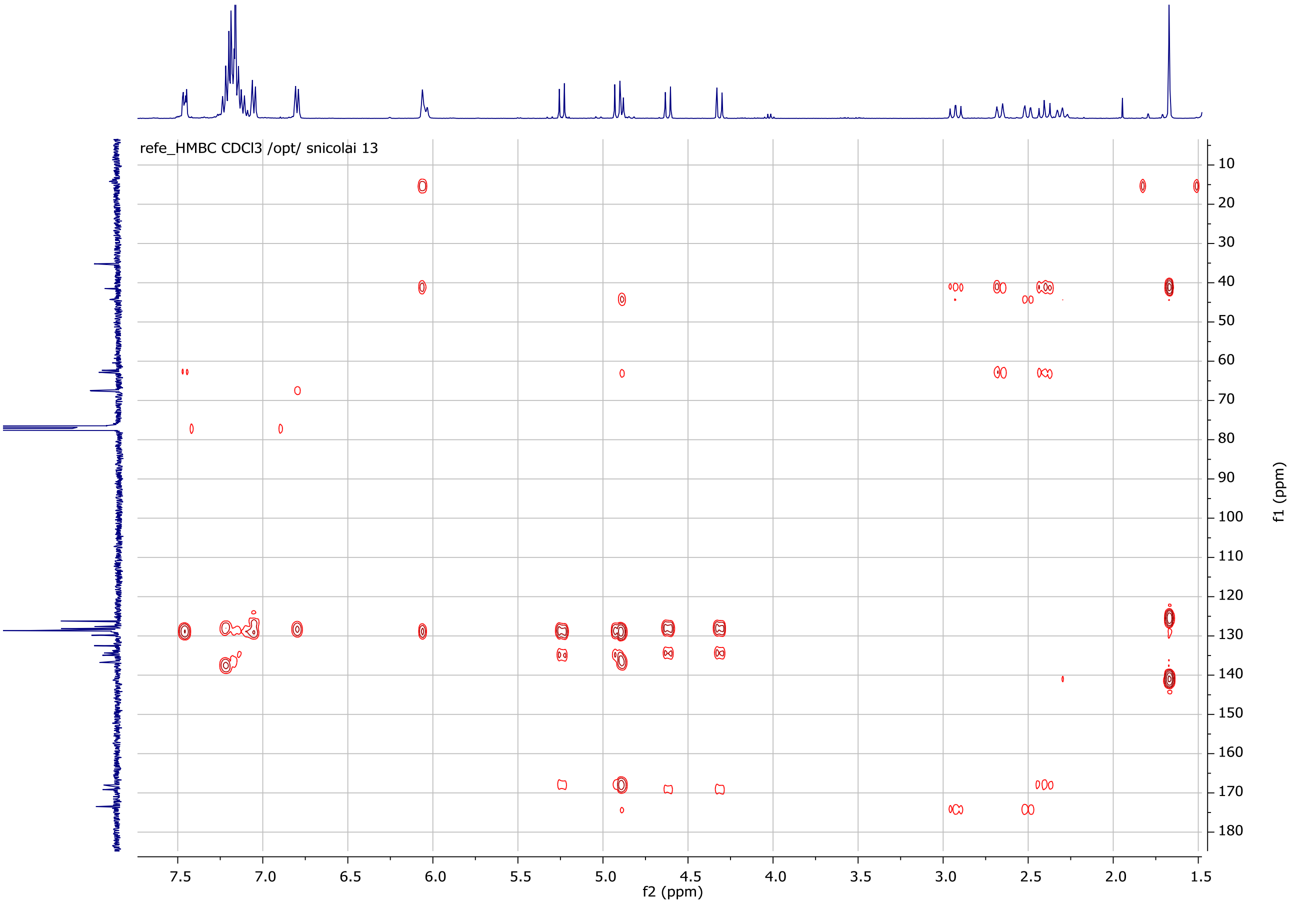
refe_HMBC CDCl3 /opt/ snicolai 13
f2 (ppm)
f1 (ppm)

Dibenzyl 2,5-*trans*-7-oxo-2-phenyl-5-((E)-styryl)azepane-3,3-dicarboxylate (5a.k)

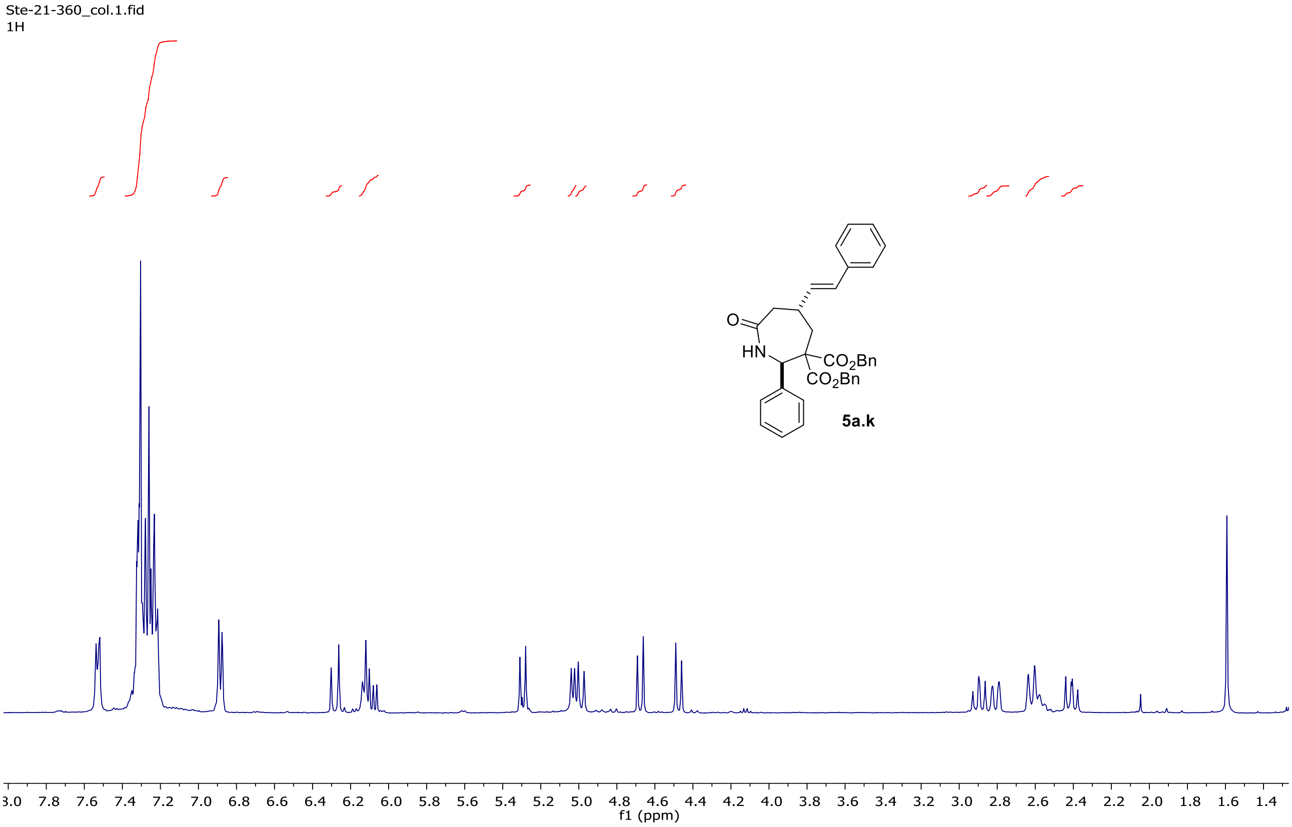

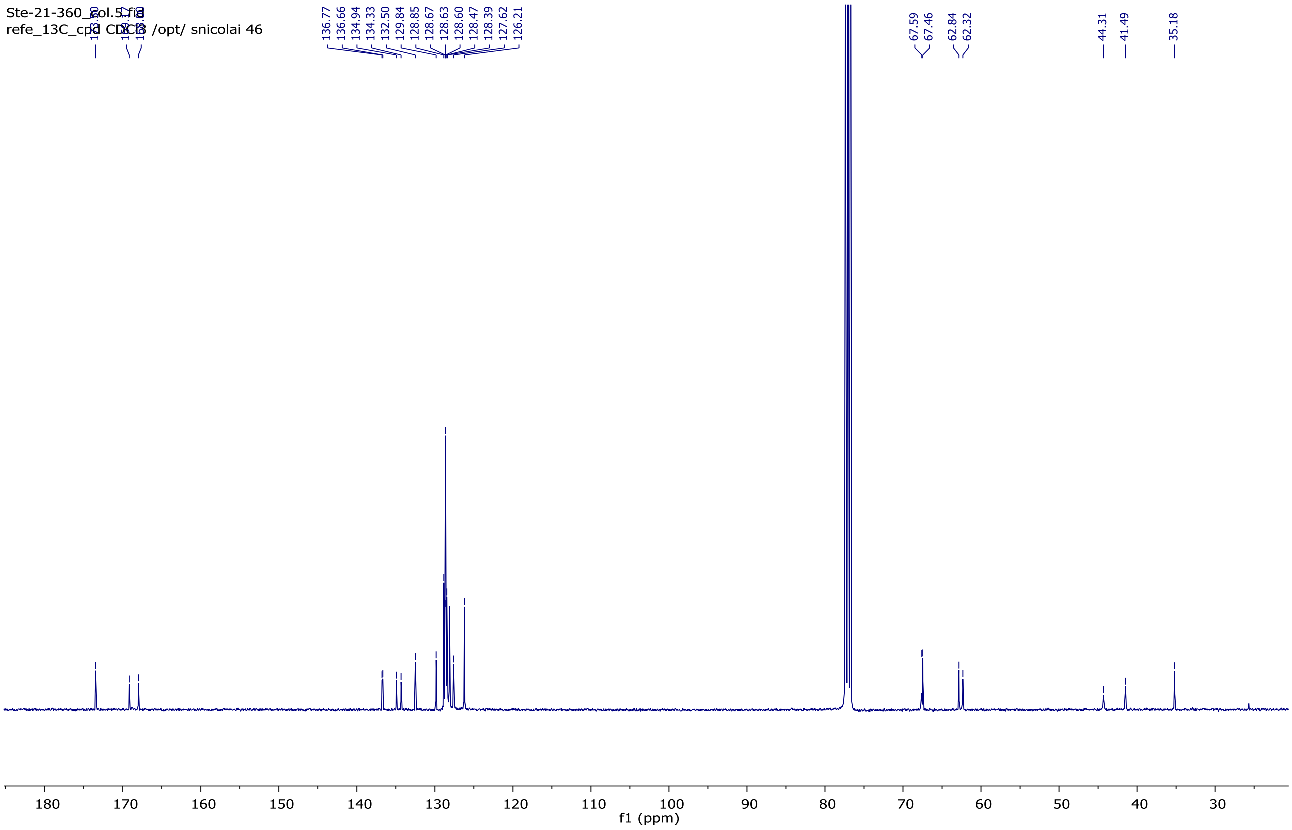

Dibenzyl 2,5-*trans*-5-(4-methoxyphenyl)-7-oxo-2-(4-(trifluoromethyl)phenyl)azepane-3,3-dicarboxylate (5b.a)

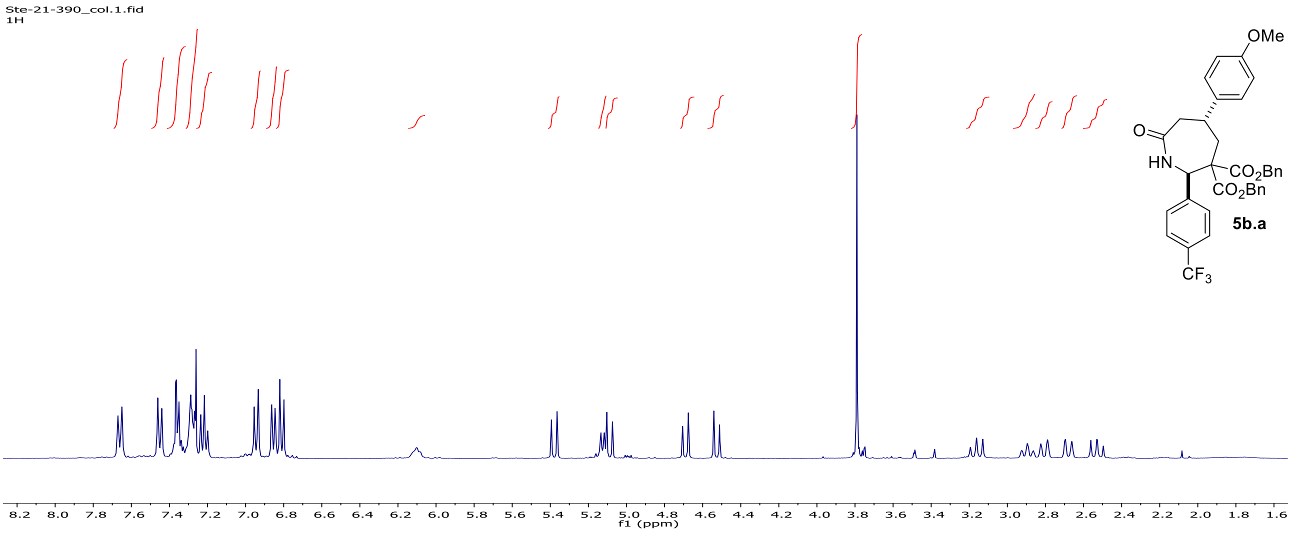

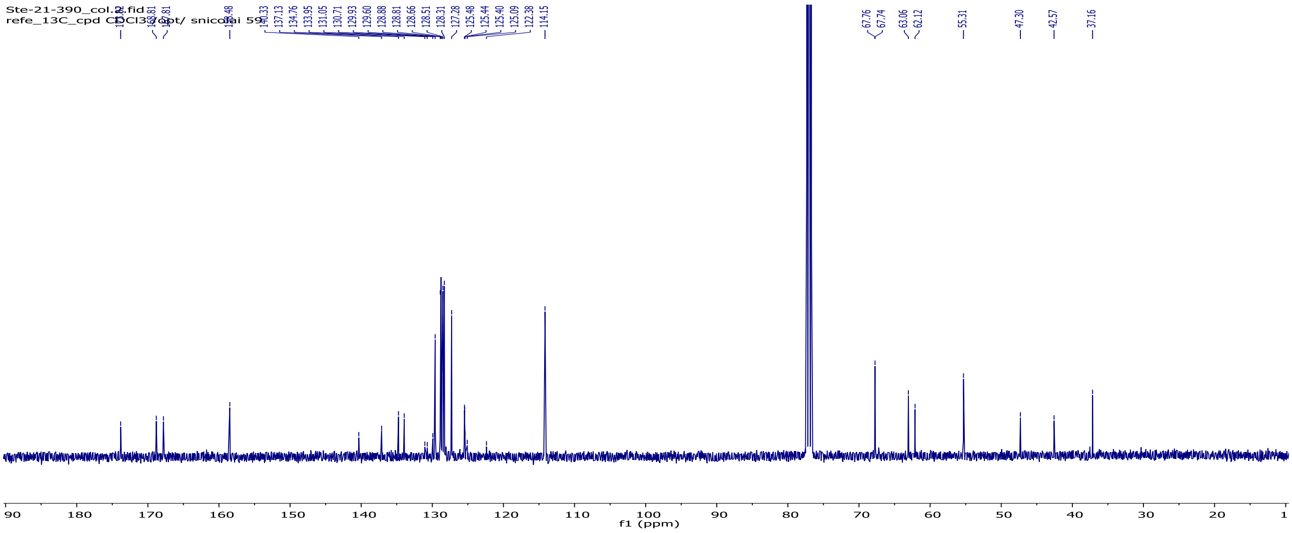

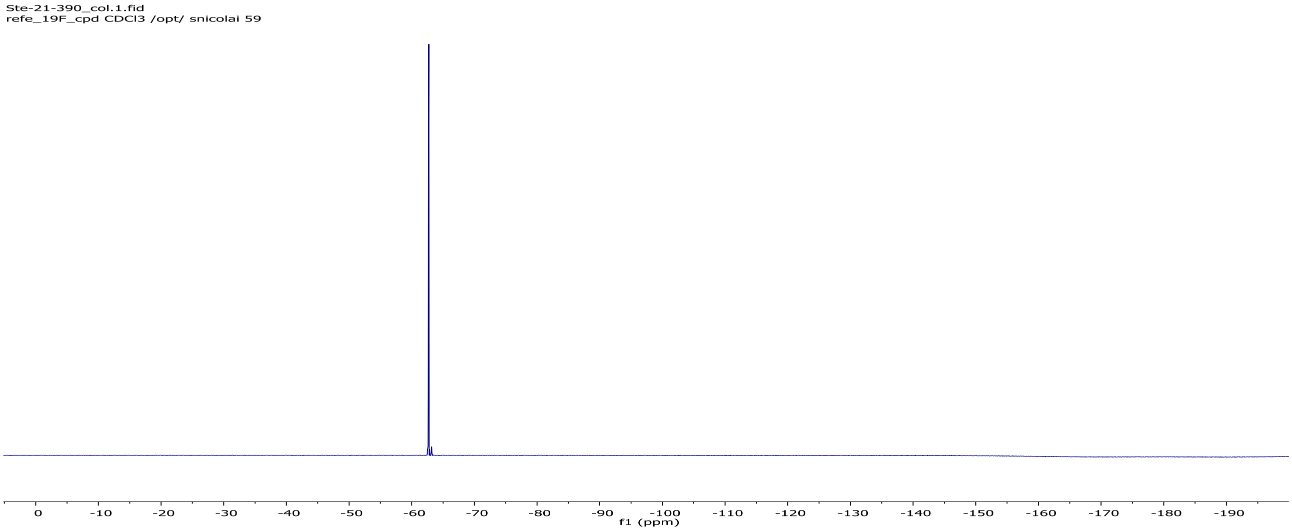

Dibenzyl 2,5-*trans*-2-(4-chlorophenyl)-5-(4-methoxyphenyl)-7-oxoazepane-3,3-dicarboxylate (5c.a)

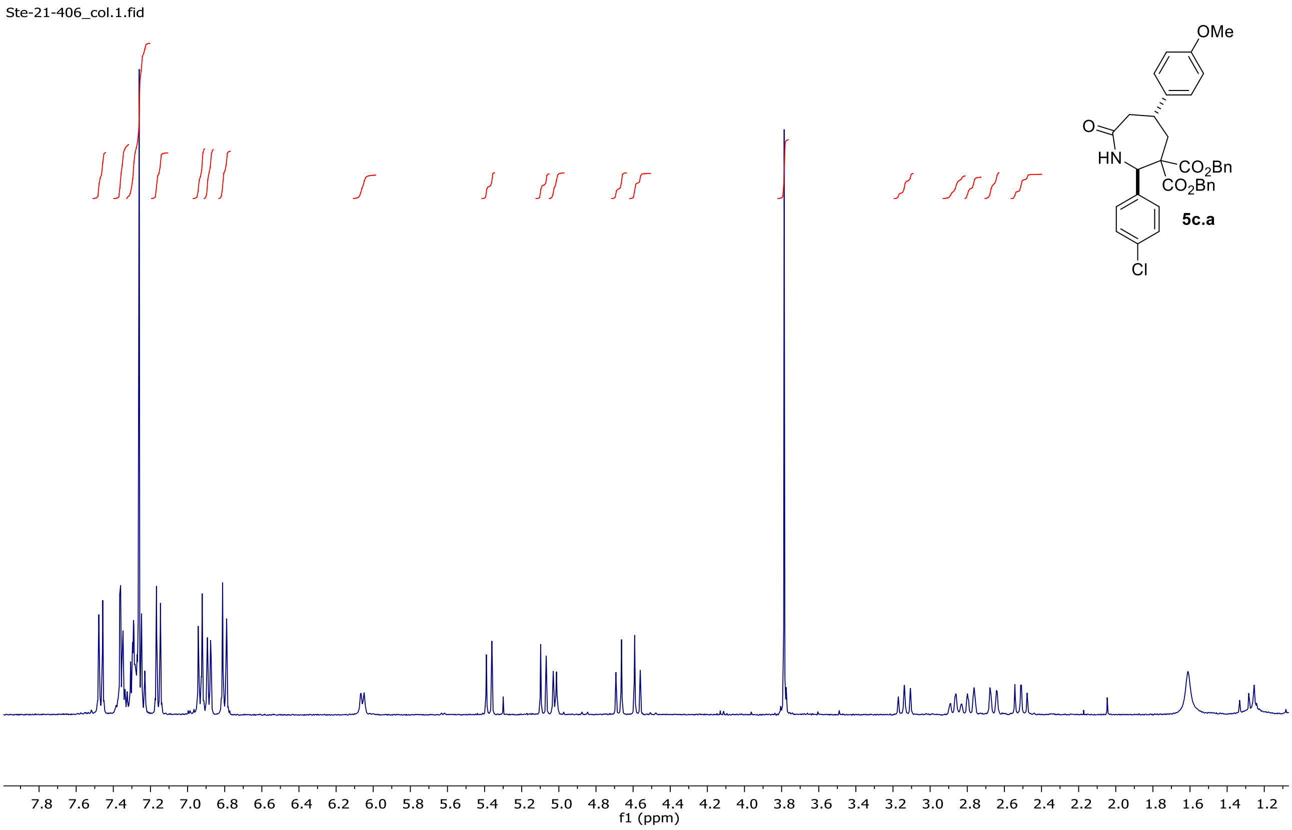

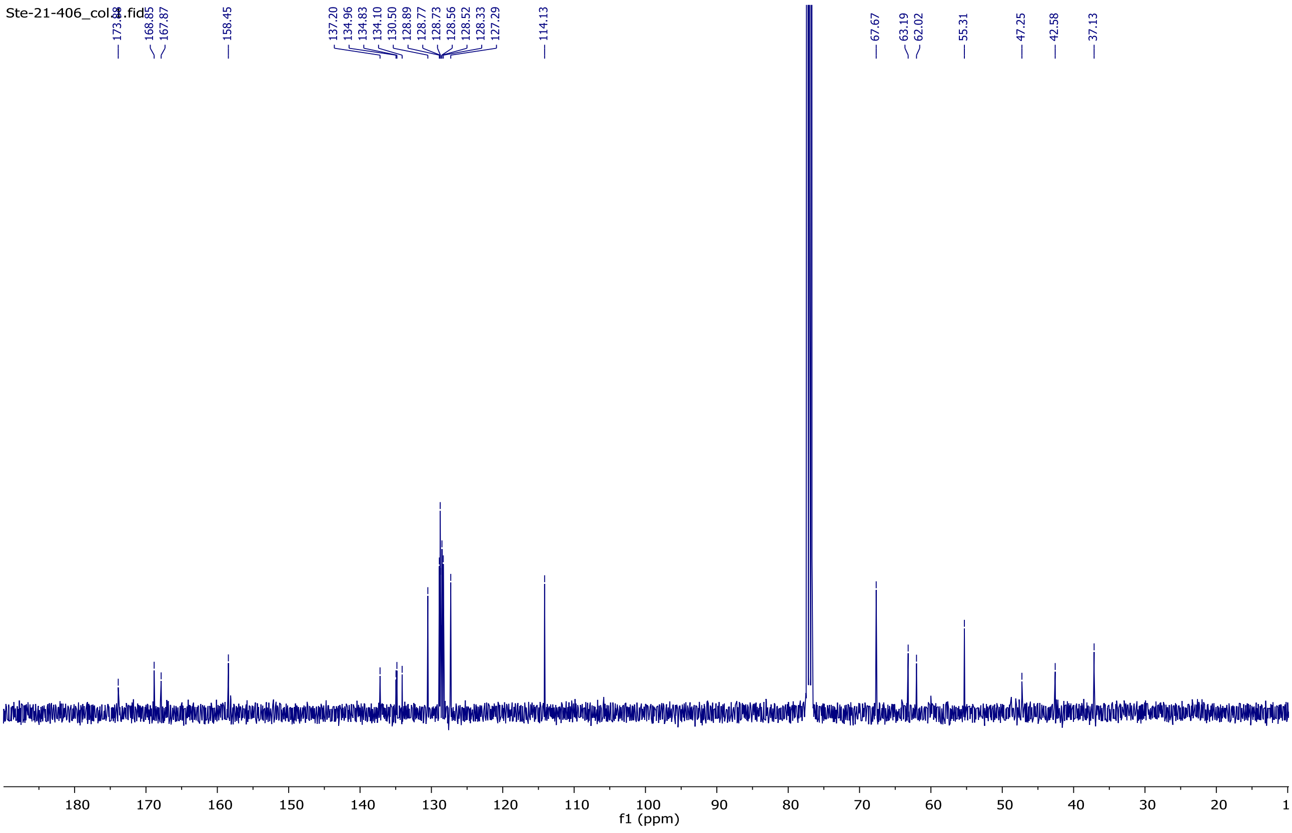

Dibenzyl 2,5-*trans*-2-(2-fluorophenyl)-5-(4-methoxyphenyl)-7-oxoazepane-3,3-dicarboxylate (5d.a)

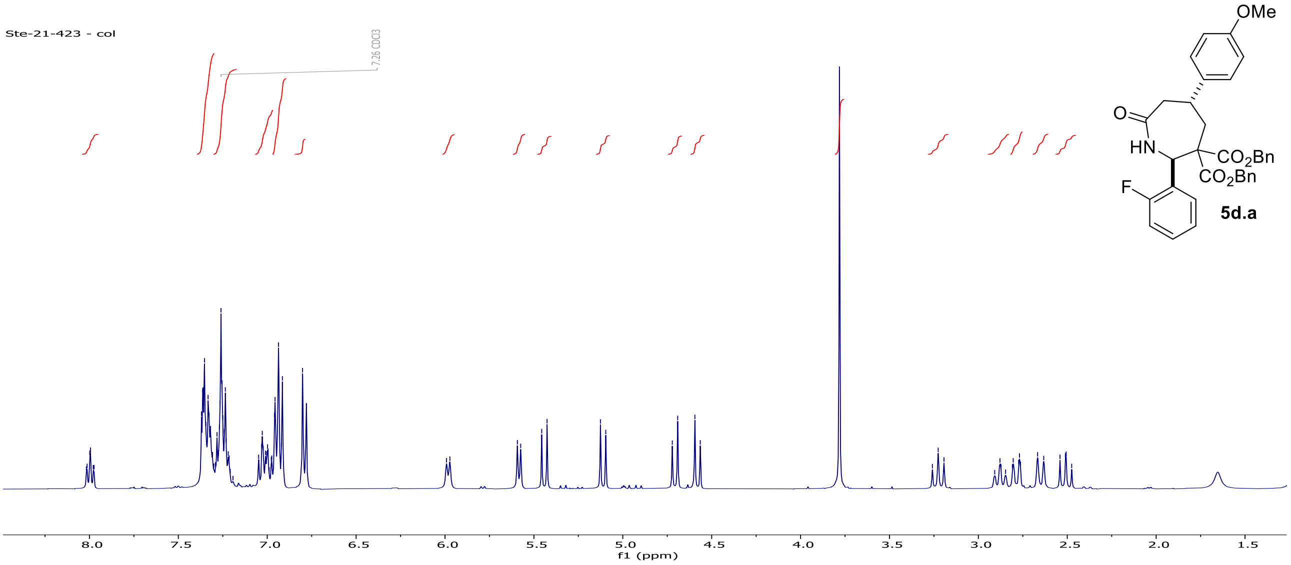

5d.a

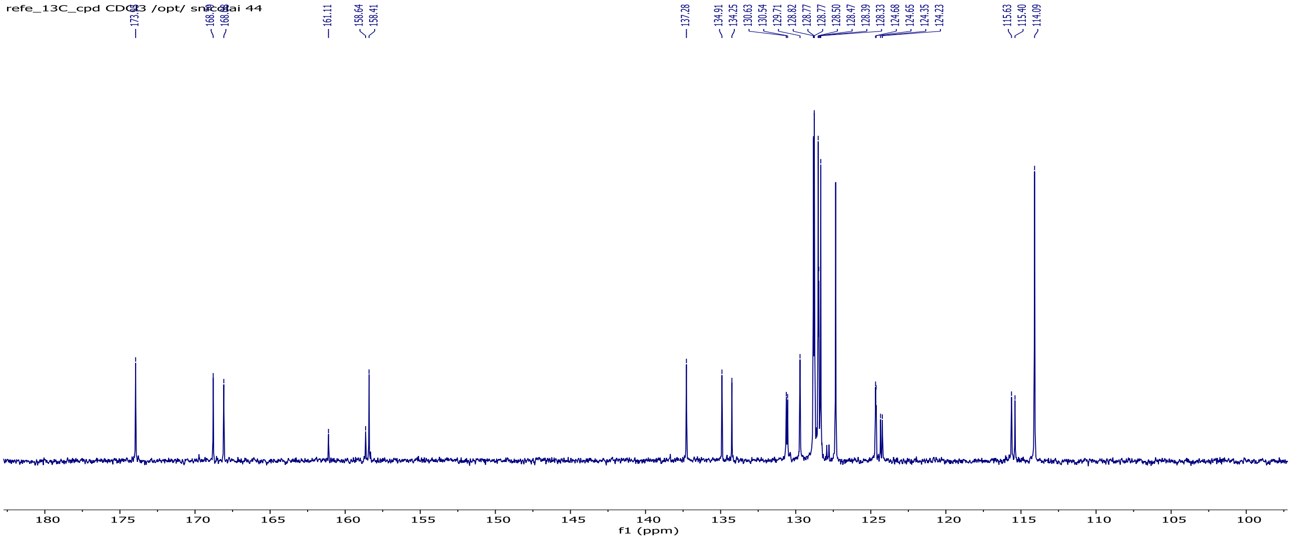

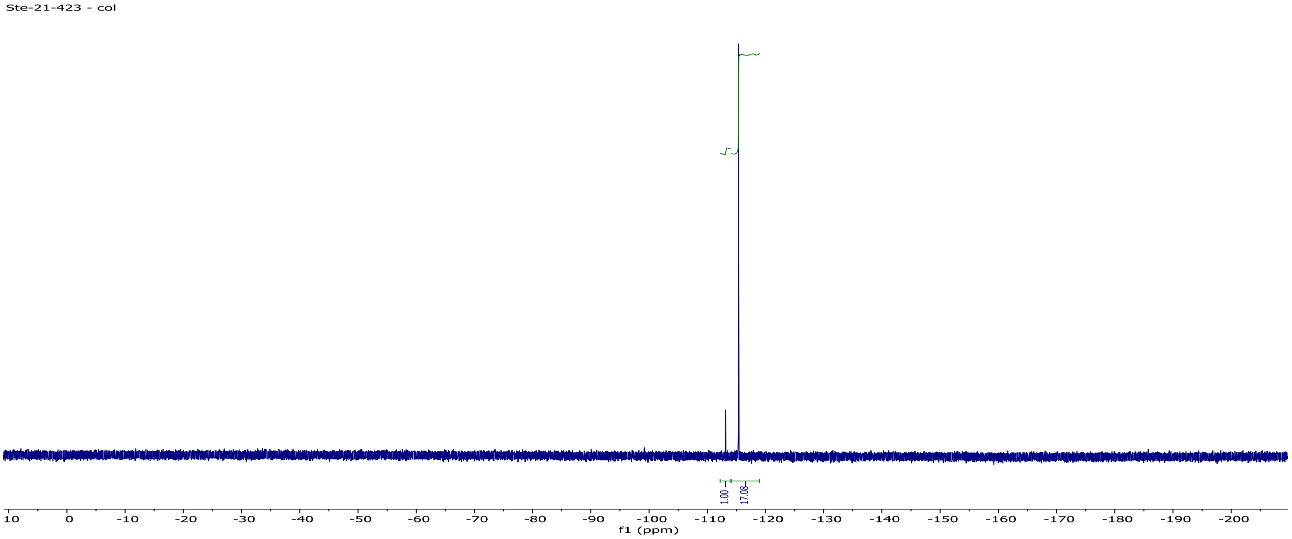

Dibenzyl 2,5-*trans*-2-(3-bromophenyl)-5-(4-methoxyphenyl)-7-oxoazepane-3,3-dicarboxylate (5e.a)

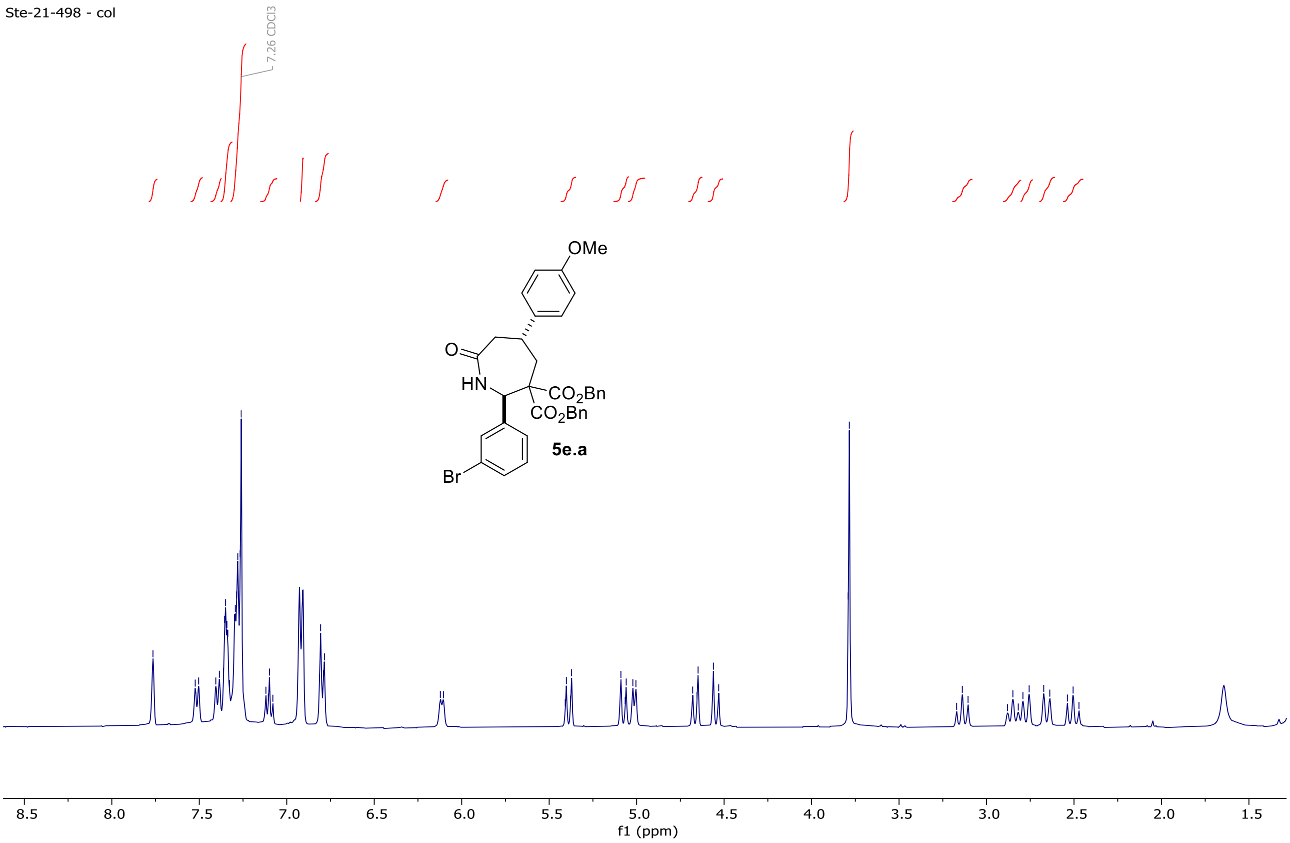

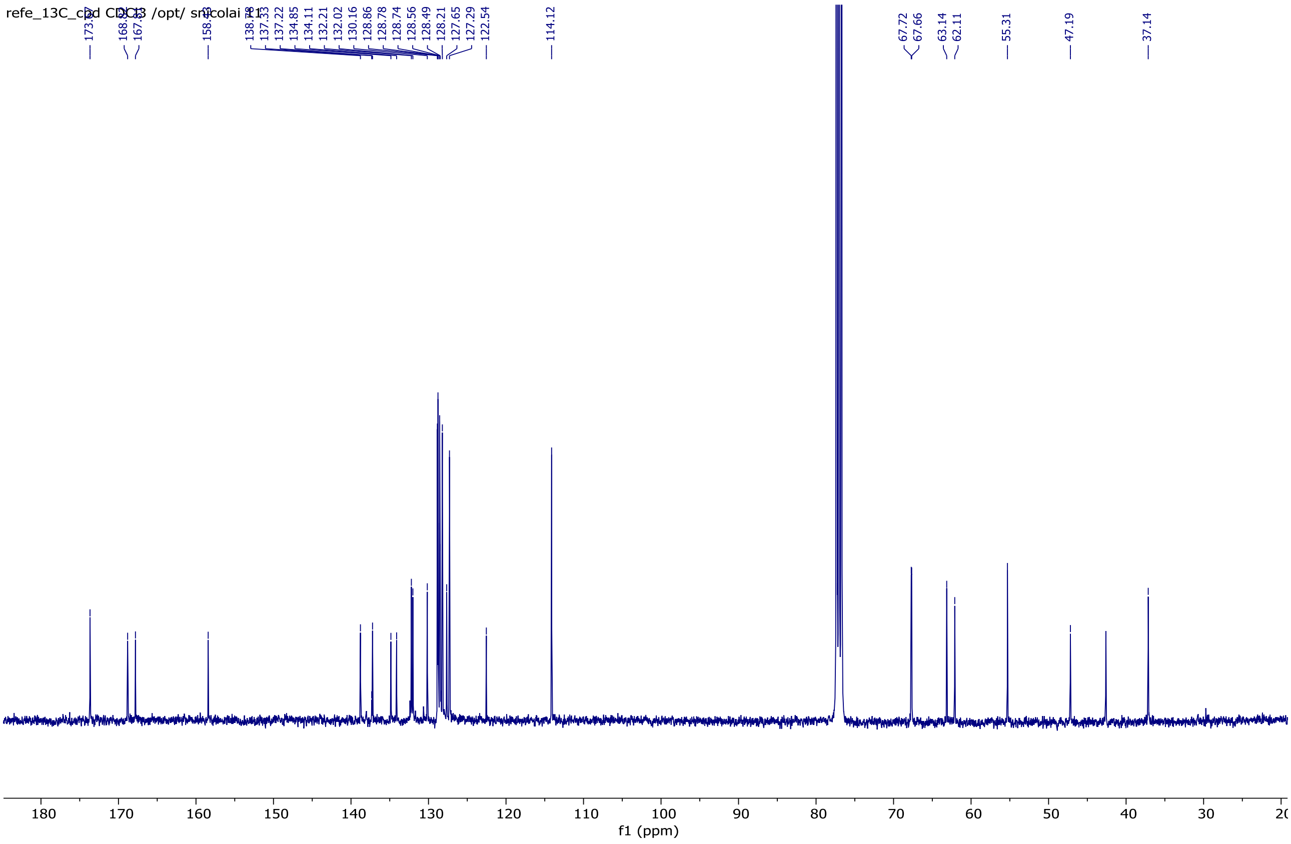

Dibenzyl 2,5-*trans*-2,5-bis(4-methoxyphenyl)-7-oxoazepane-3,3-dicarboxylate (5f.a)

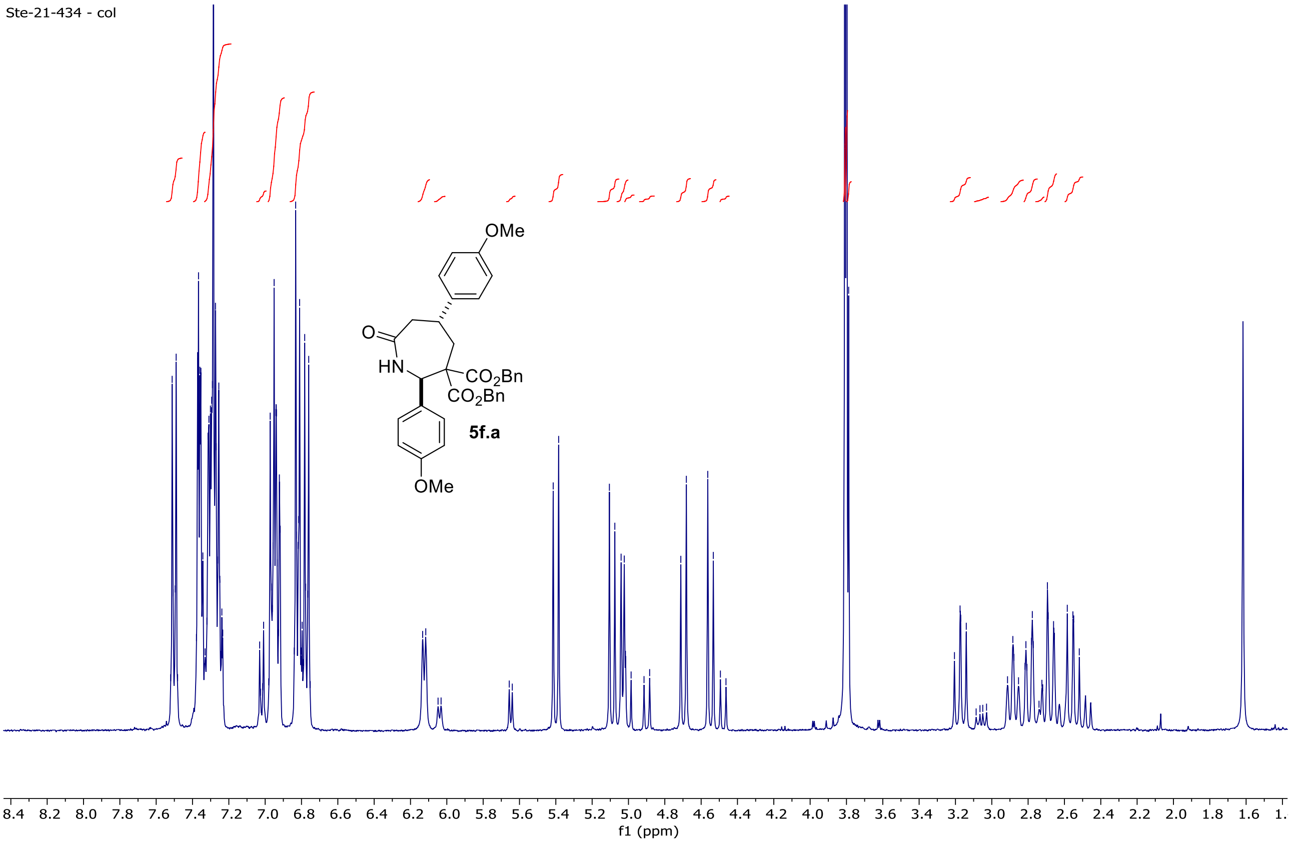

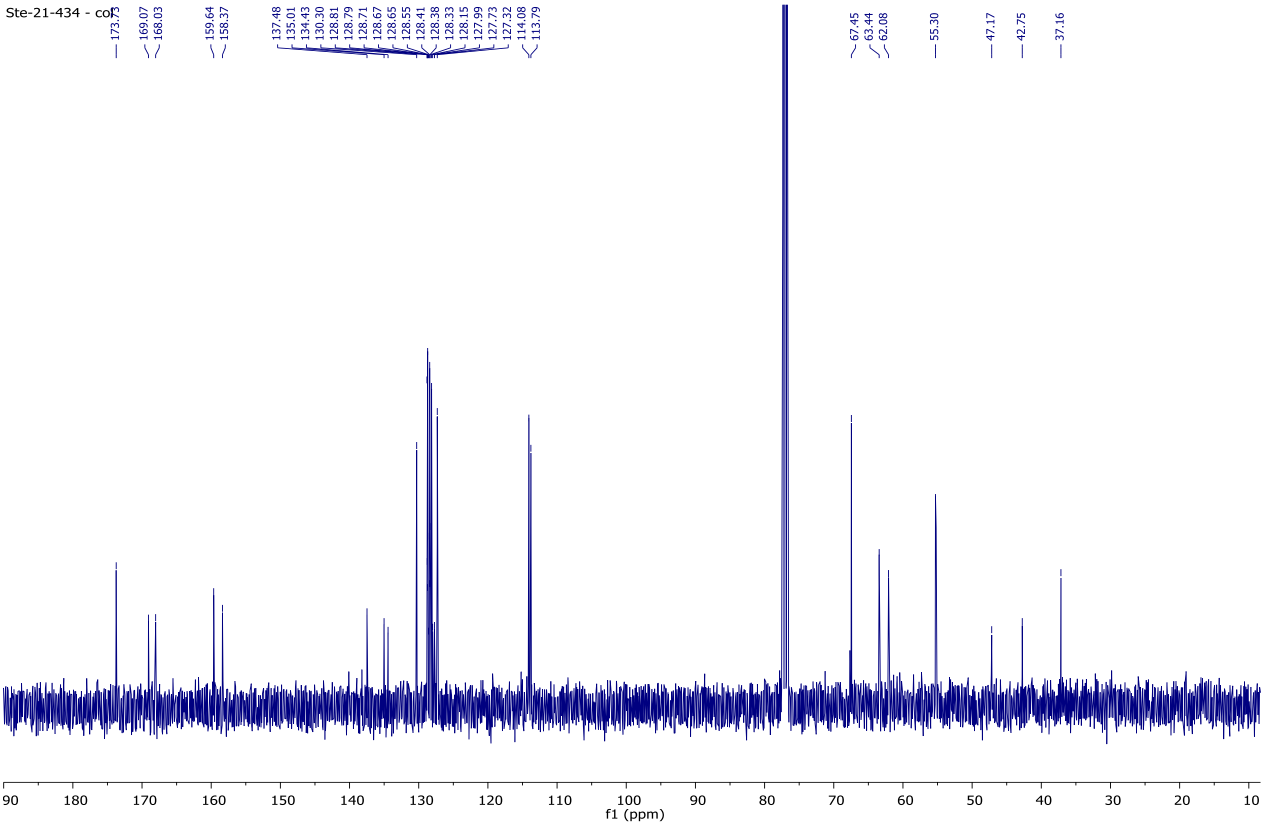

Dibenzyl 2,5-*trans*-5-(4-methoxyphenyl)-7-oxo-2-((E)-1-phenylprop-1-en-2-yl)azepane-3,3-dicarboxylate (5g.a)

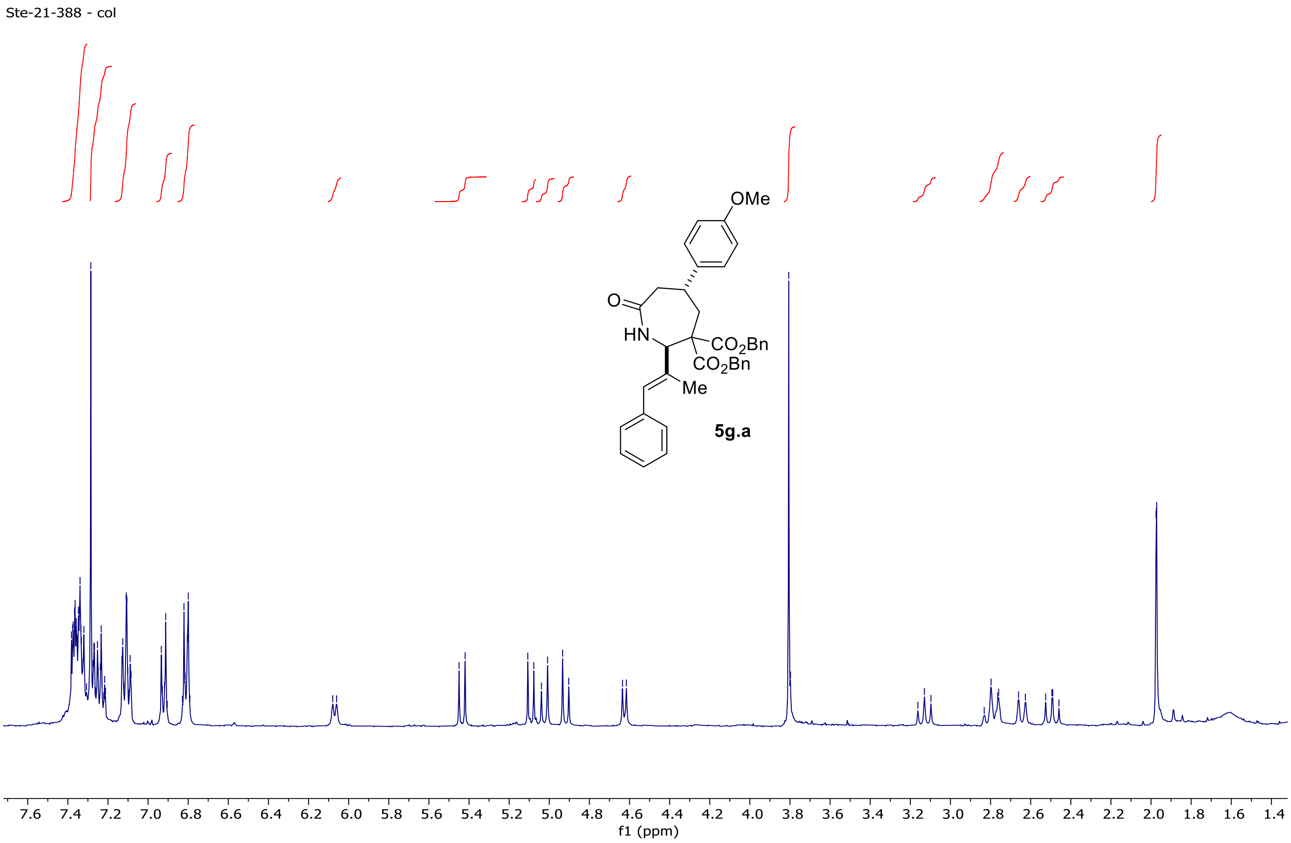

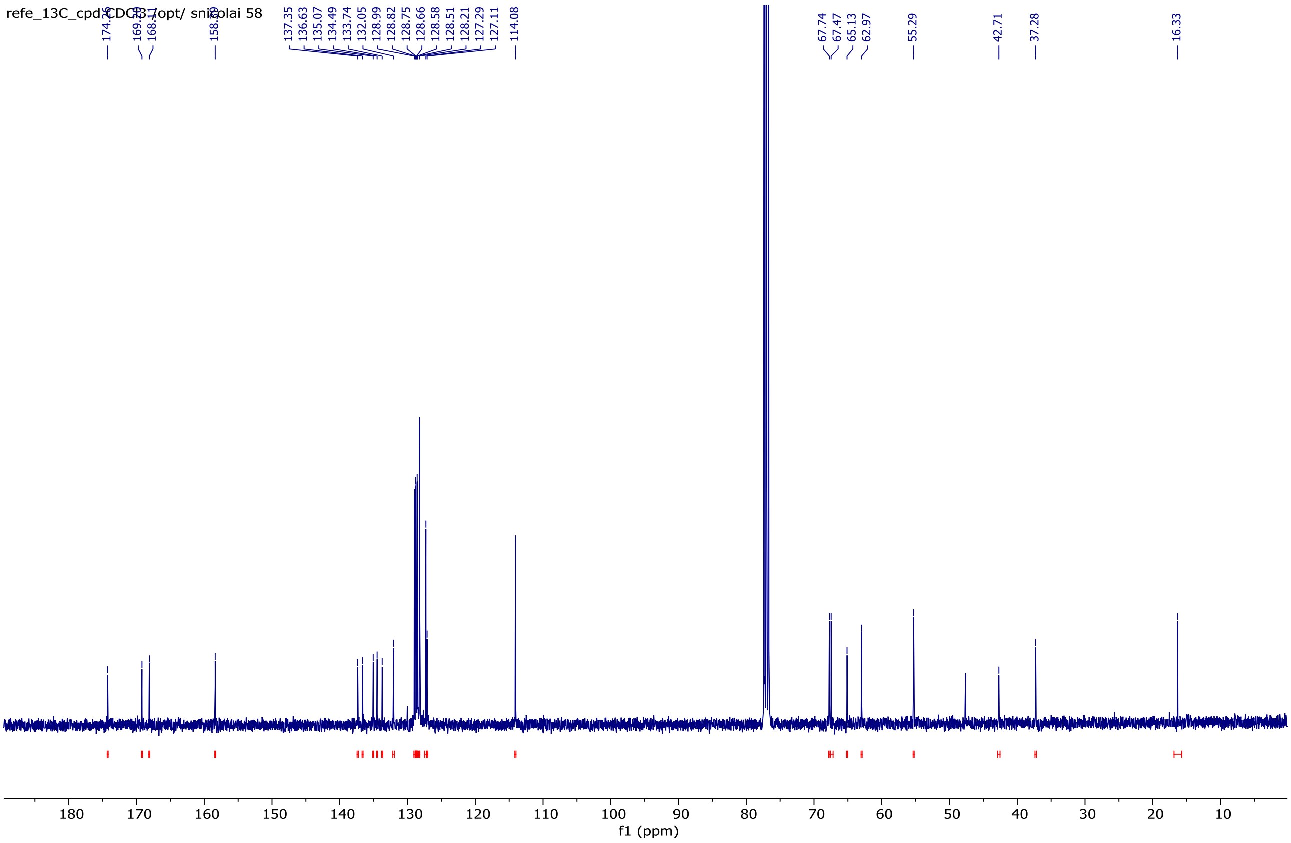

Dibenzyl 2,5-*trans*-5-(1,3-dioxoisoindolin-2-yl)-7-oxo-2-phenylazepane-3,3-dicarboxylate (5a.l)

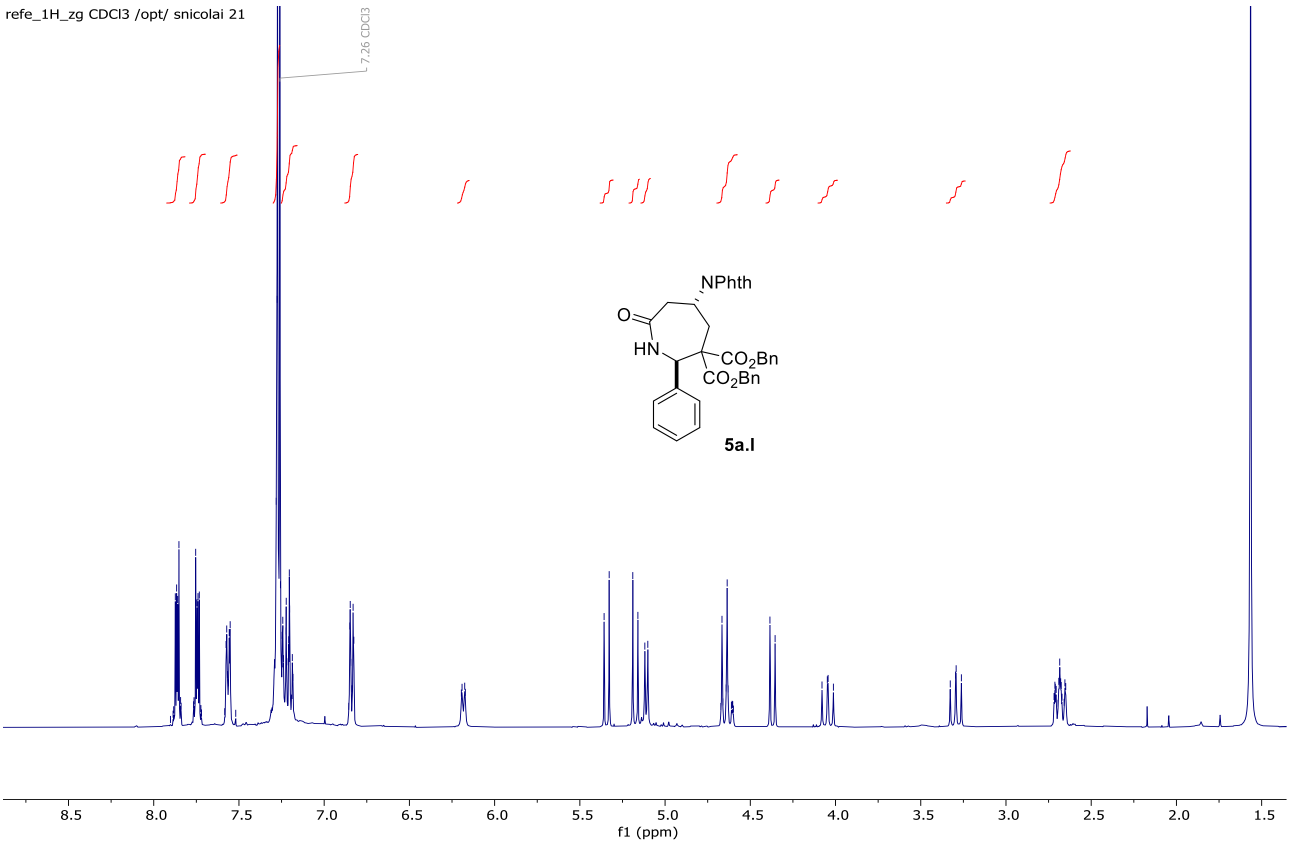

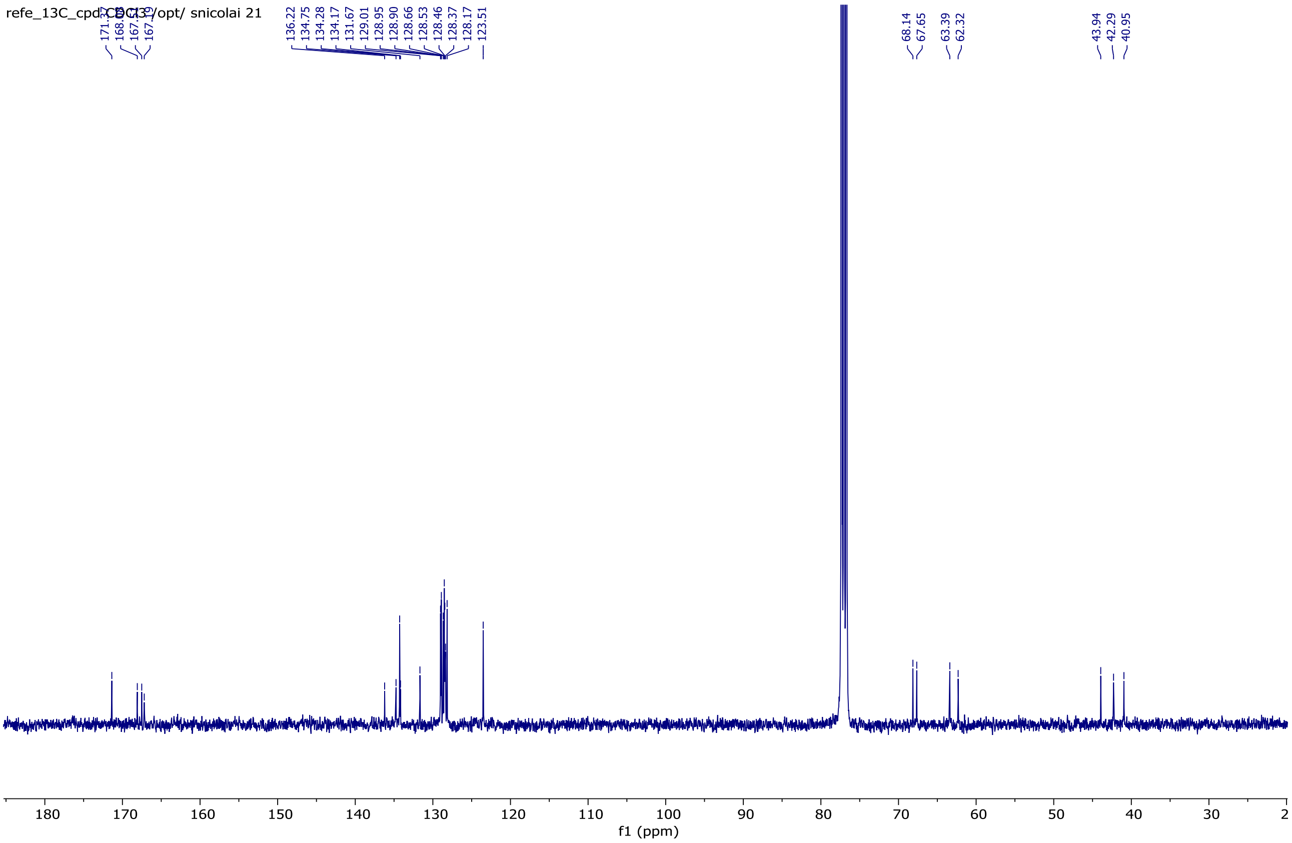

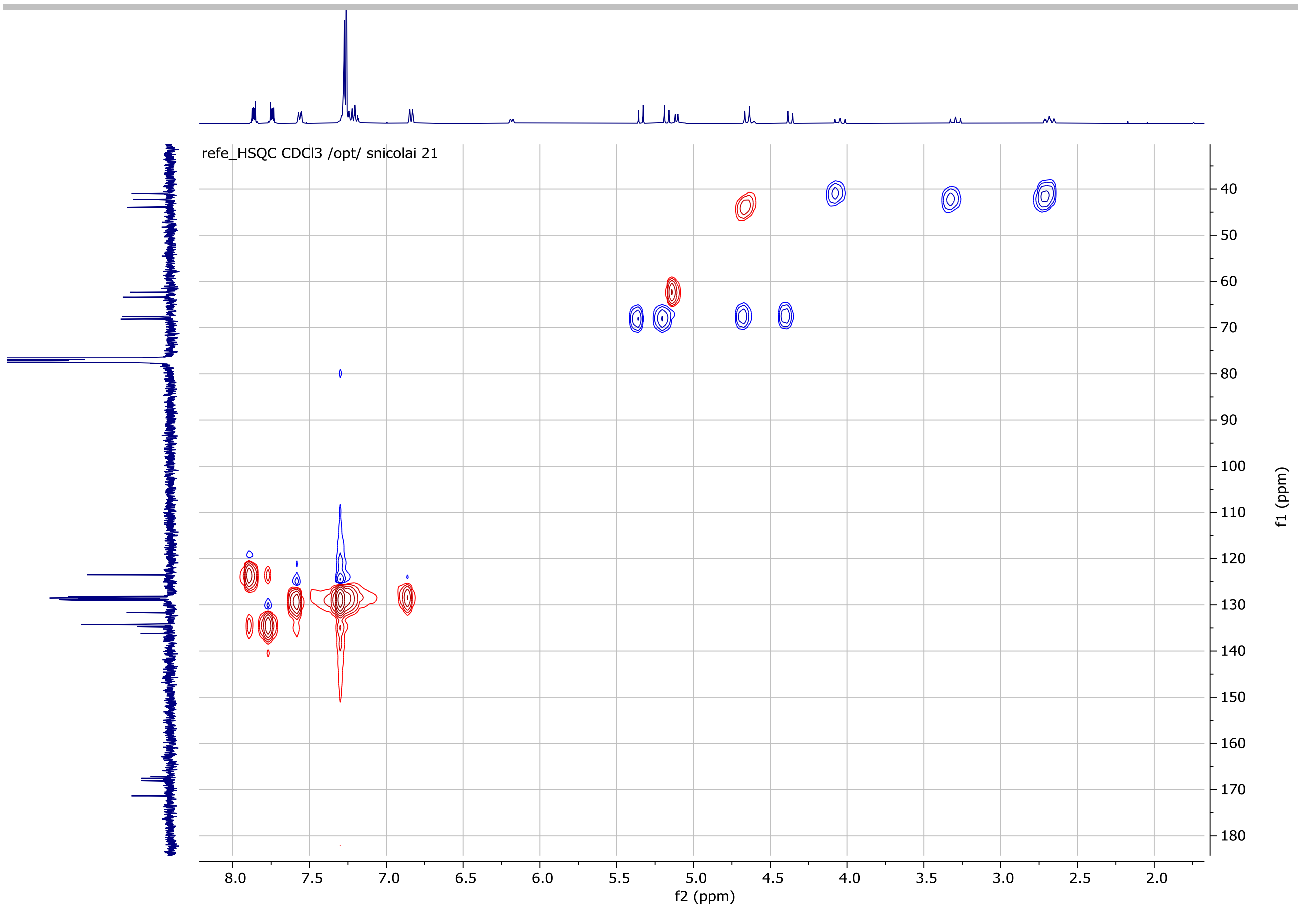
refe_HSQC CDCl3 /opt/ snicolai 21
f2 (ppm)
f1 (ppm)

Dibenzyl 2,5-*trans*-5-(1,3-dioxoisoindolin-2-yl)-7-oxo-2-(4-(trifluoromethyl)phenyl)azepane-3,3-dicarboxylate (5b.l)

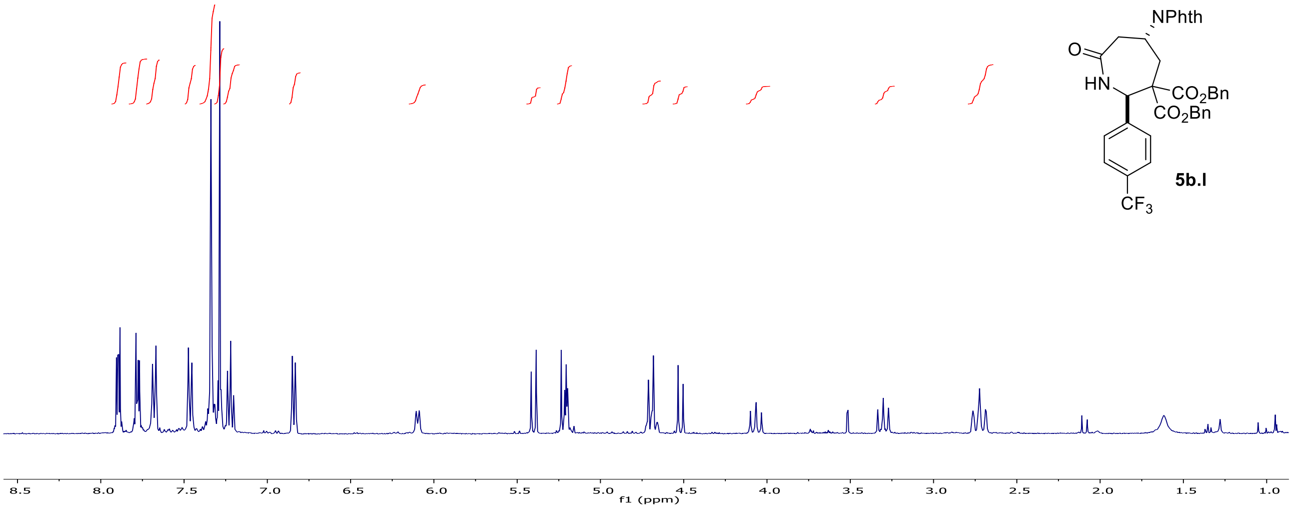

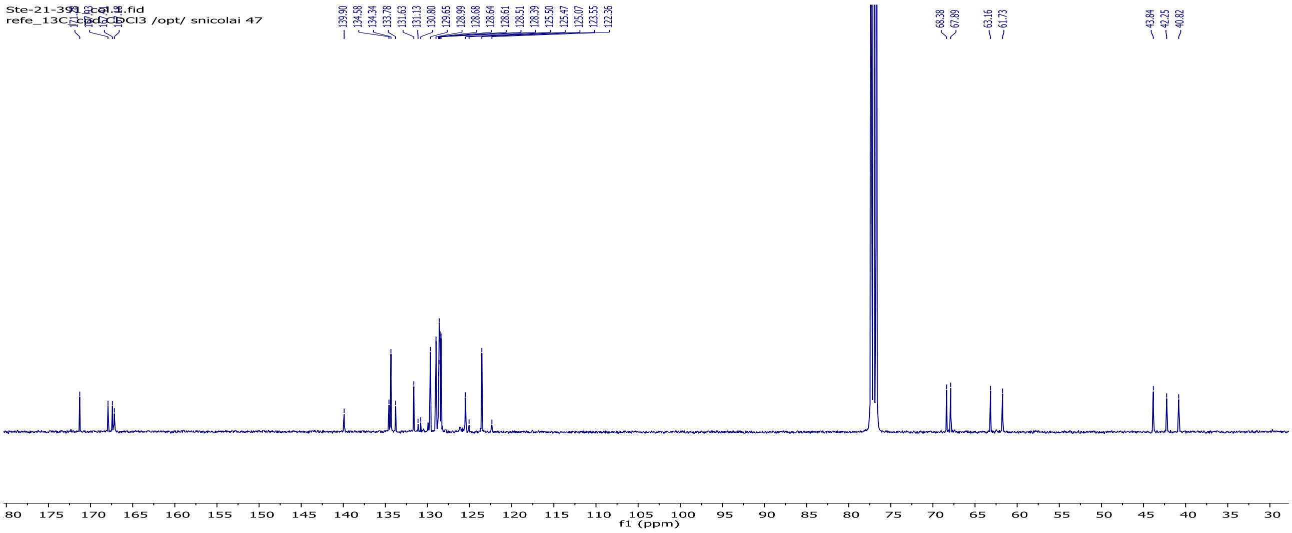

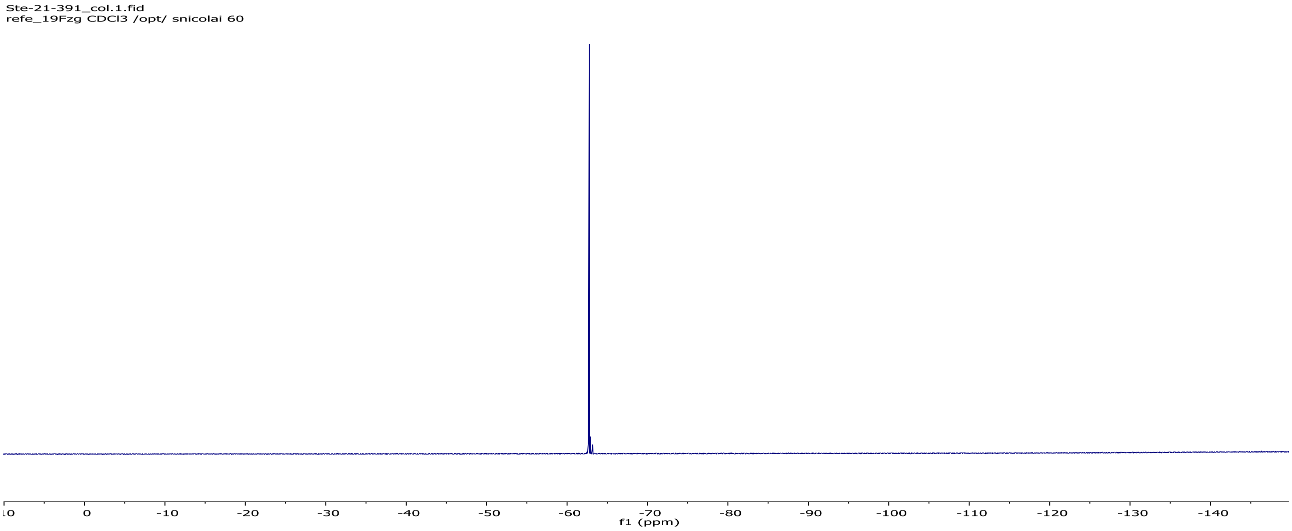

Dibenzyl 2,5-*trans*-2-(4-chlorophenyl)-5-(1,3-dioxoisoindolin-2-yl)-7-oxoazepane-3,3-dicarboxylate (5c.I)

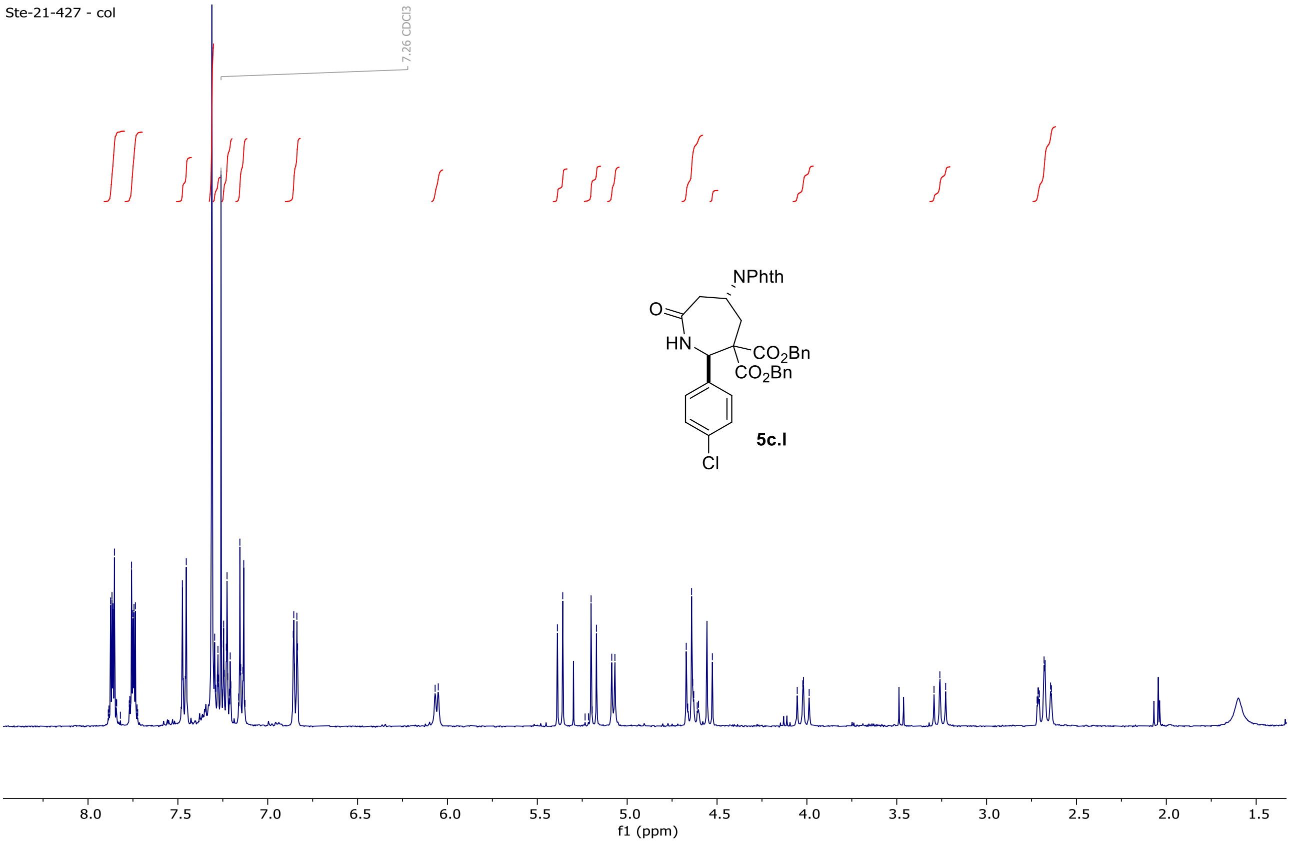

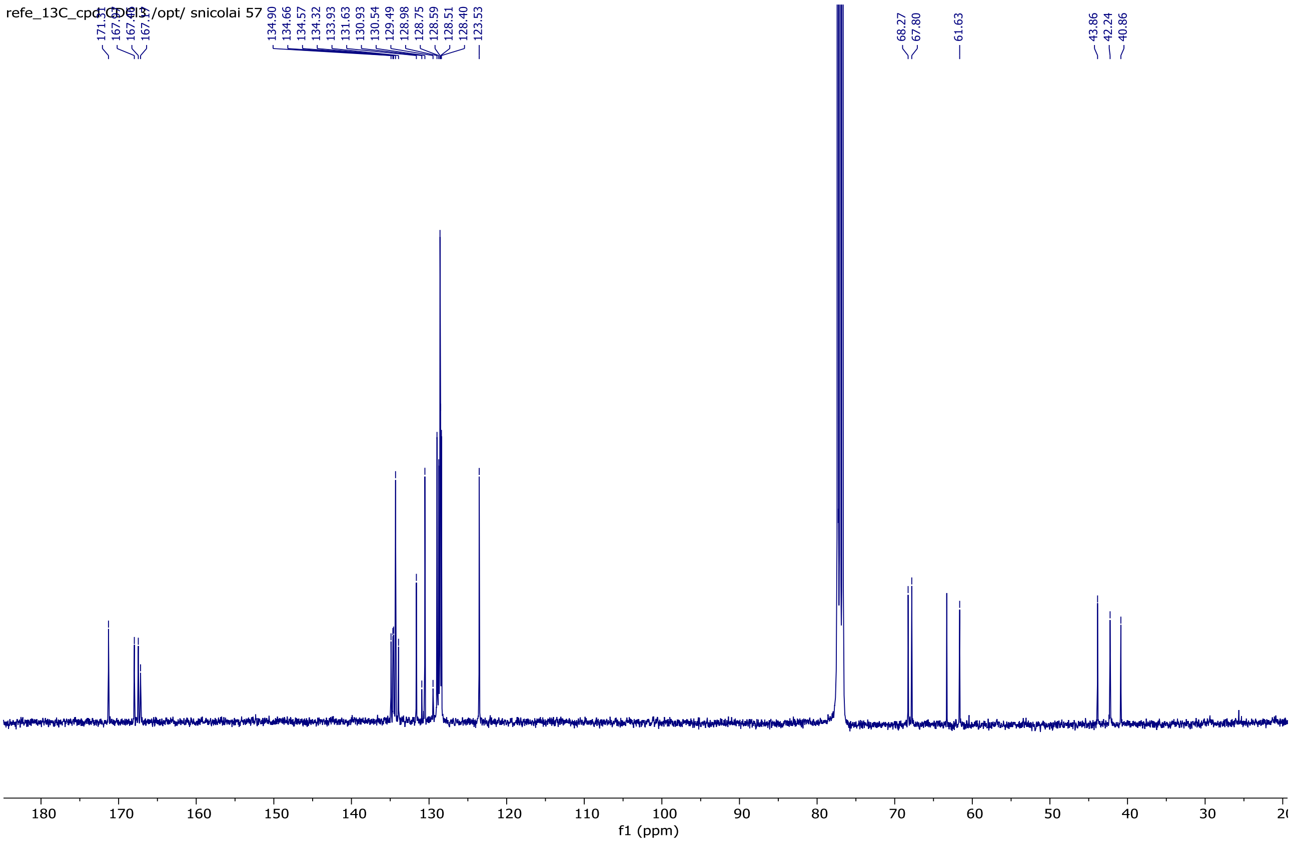

Dibenzyl 2,5-*trans*-5-(1,3-dioxoisoindolin-2-yl)-2-(2-fluorophenyl)-7-oxoazepane-3,3-dicarboxylate (5d.l)

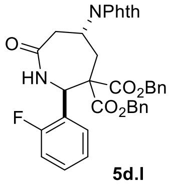

5d.l

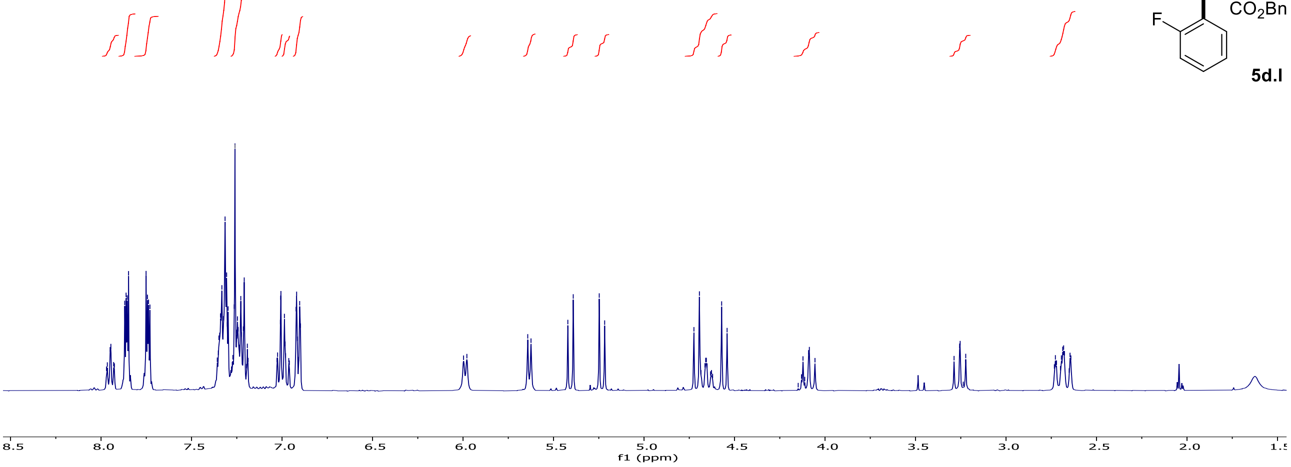

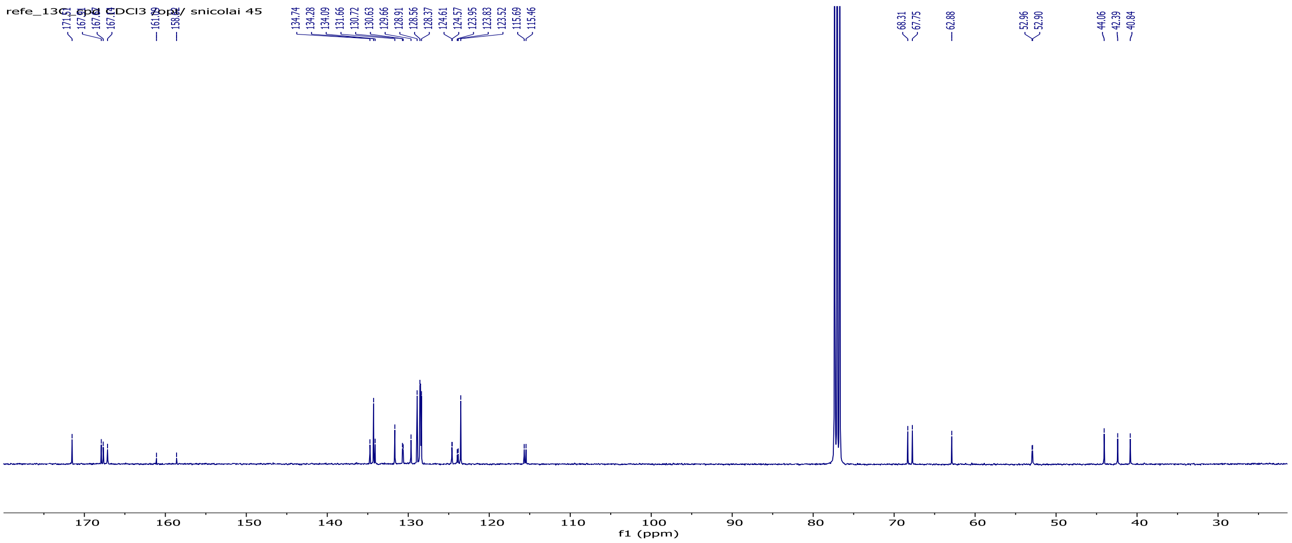

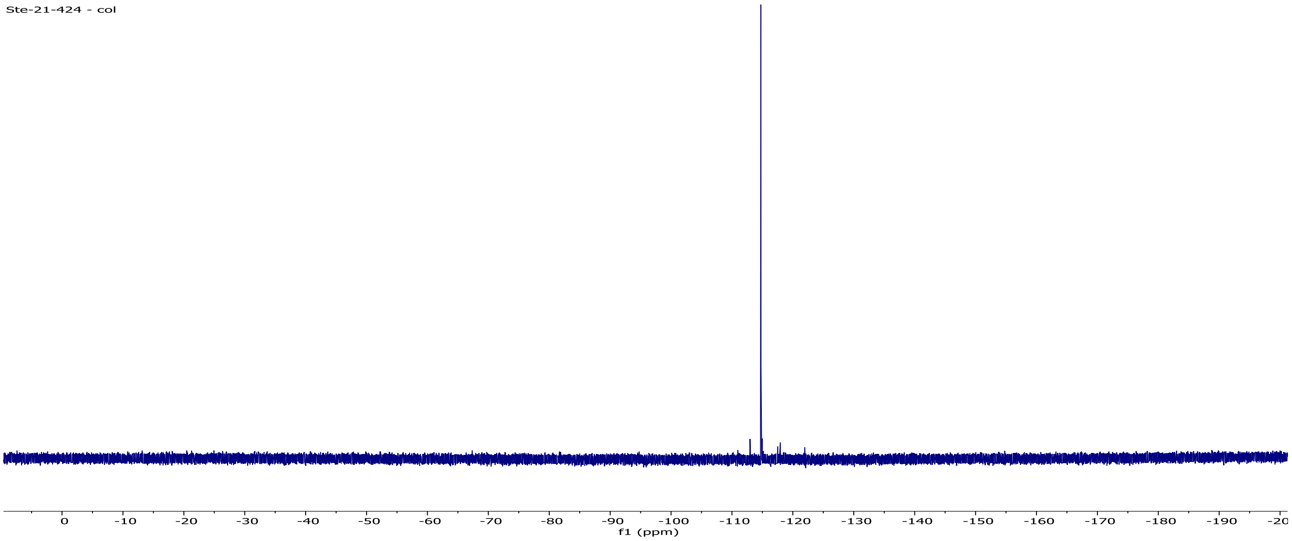

Dibenzyl 2,5-*trans*-2-(3-bromophenyl)-5-(1,3-dioxoisoindolin-2-yl)-7-oxoazepane-3,3-dicarboxylate (5e.l)

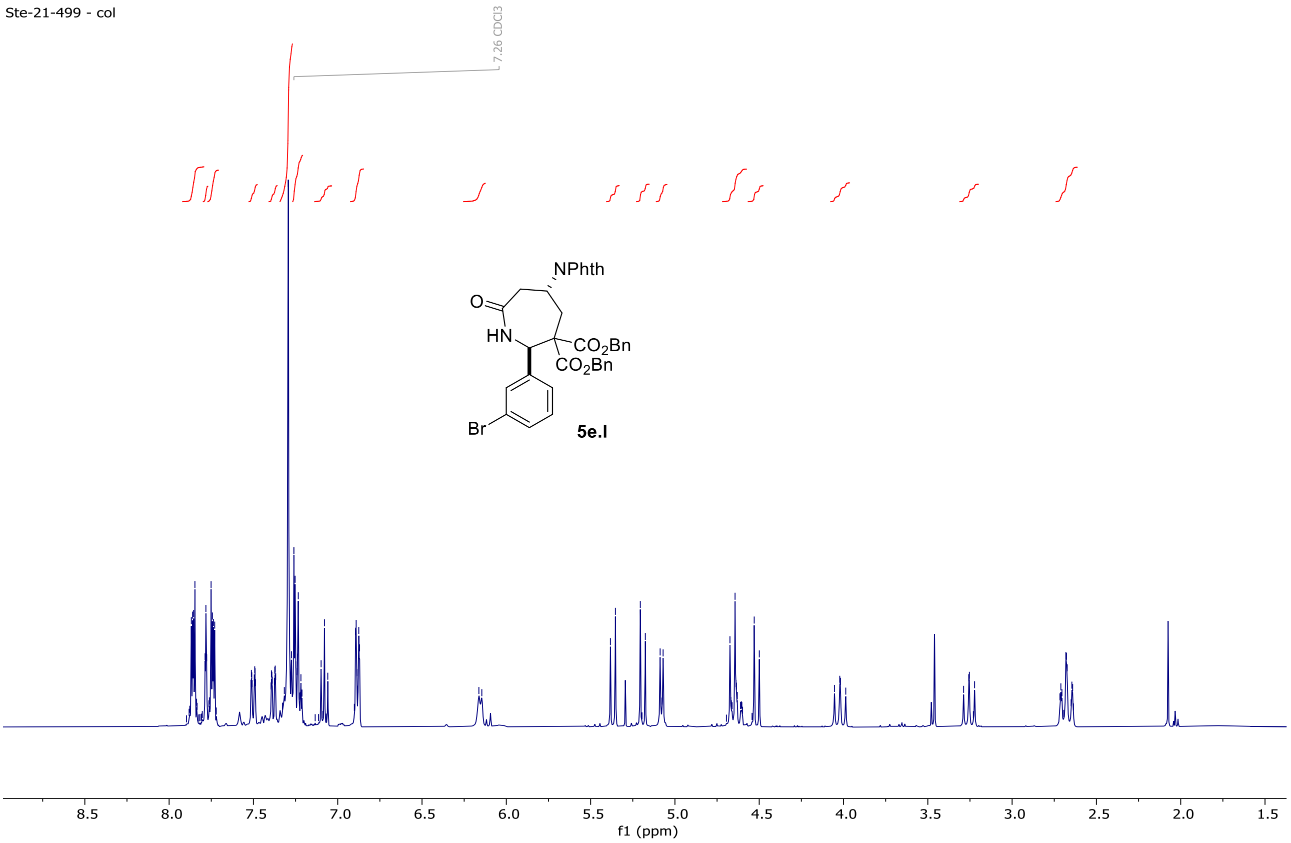

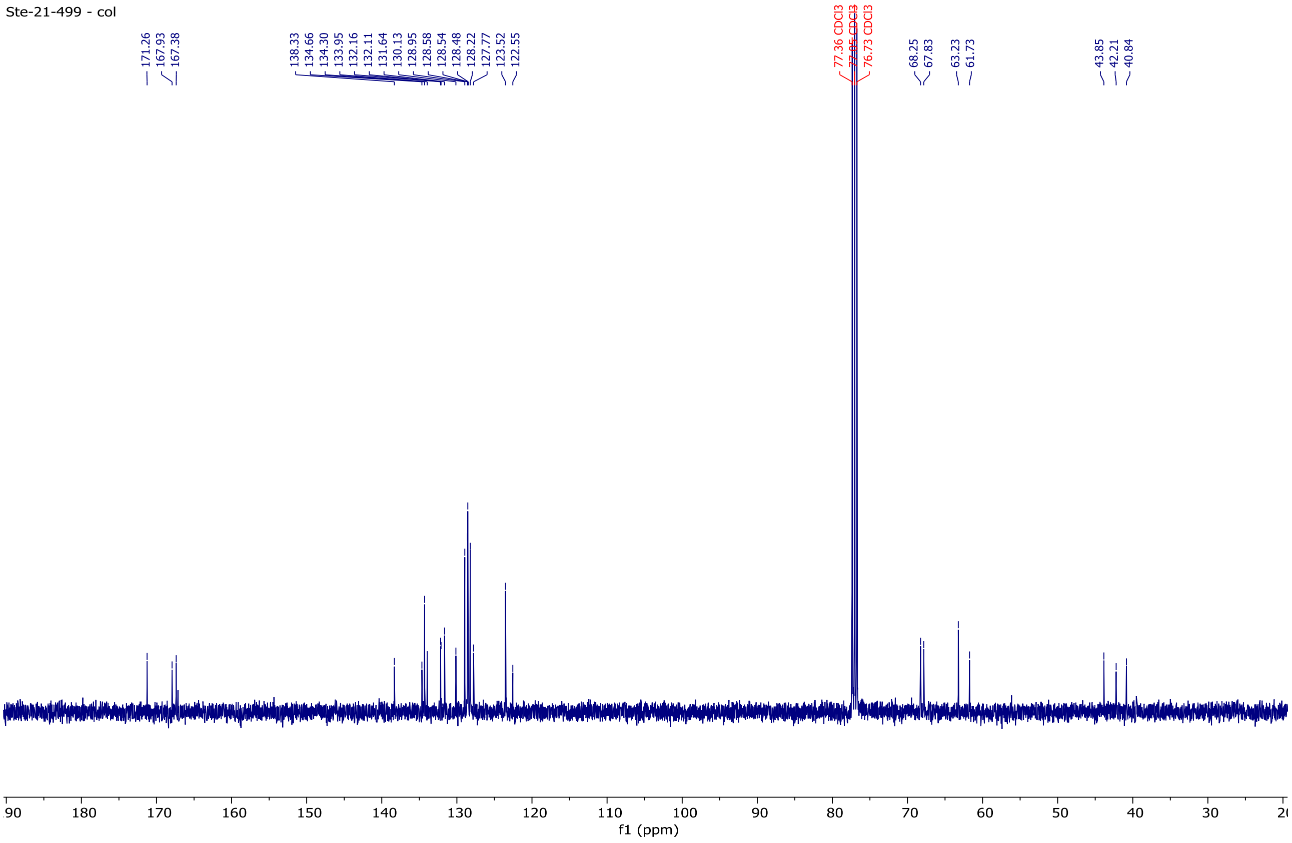

Dibenzyl 2,5-*trans*-5-(1,3-dioxoisoindolin-2-yl)-2-(4-methoxyphenyl)-7-oxoazepane-3,3-dicarboxylate (5f.l)

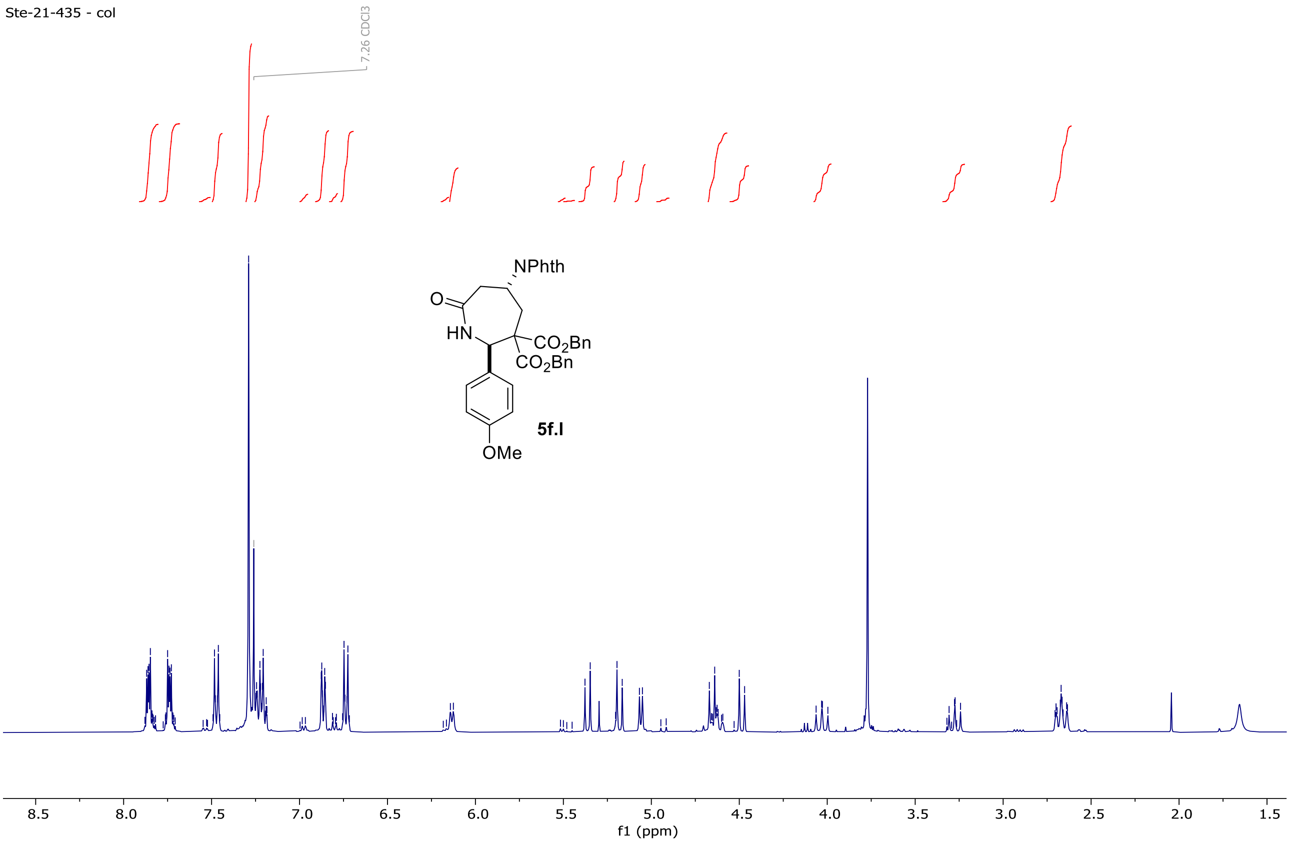

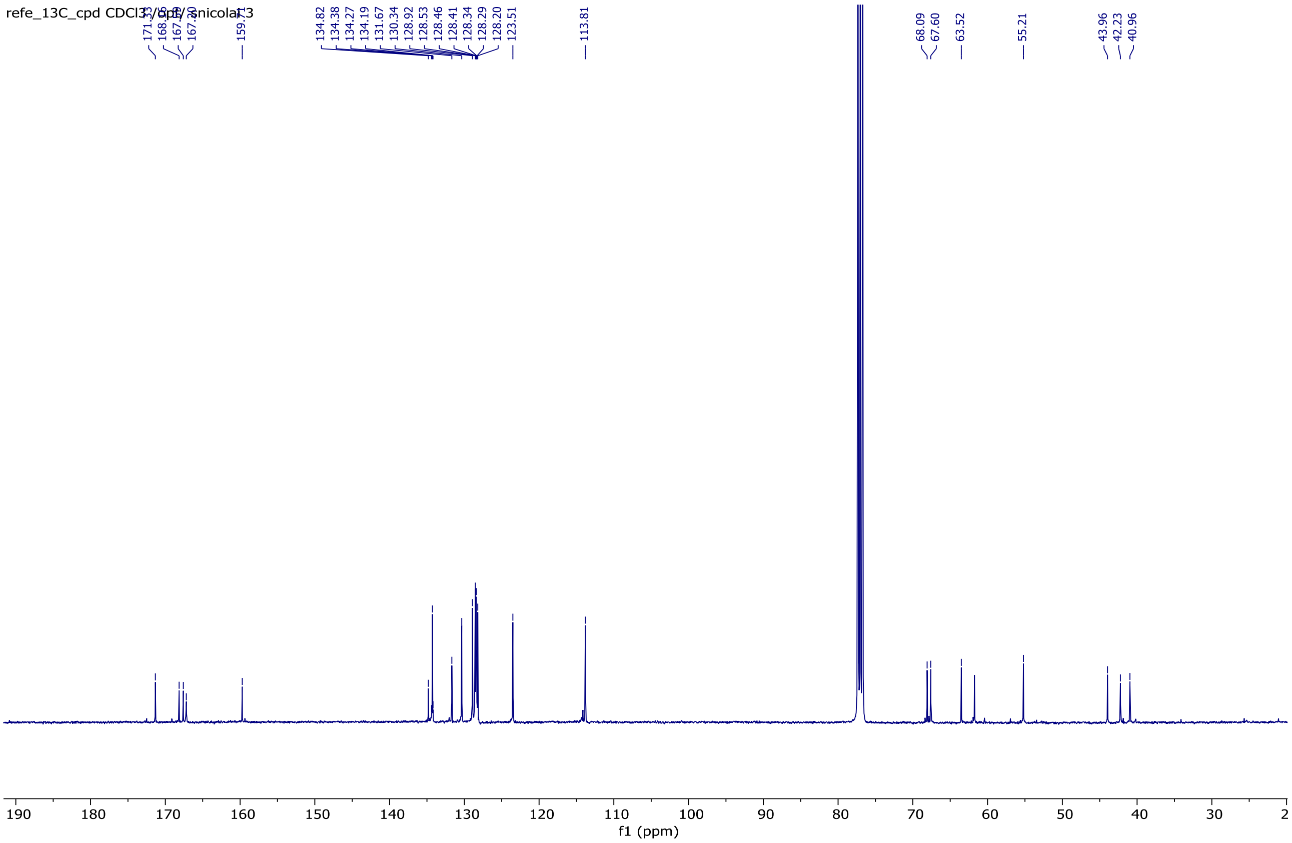

Dibenzyl 2,5-*trans*-5-(1,3-dioxoisoindolin-2-yl)-7-oxo-2-((E)-1-phenylprop-1-en-2-yl)azepane-3,3-dicarboxylate (5g.l)

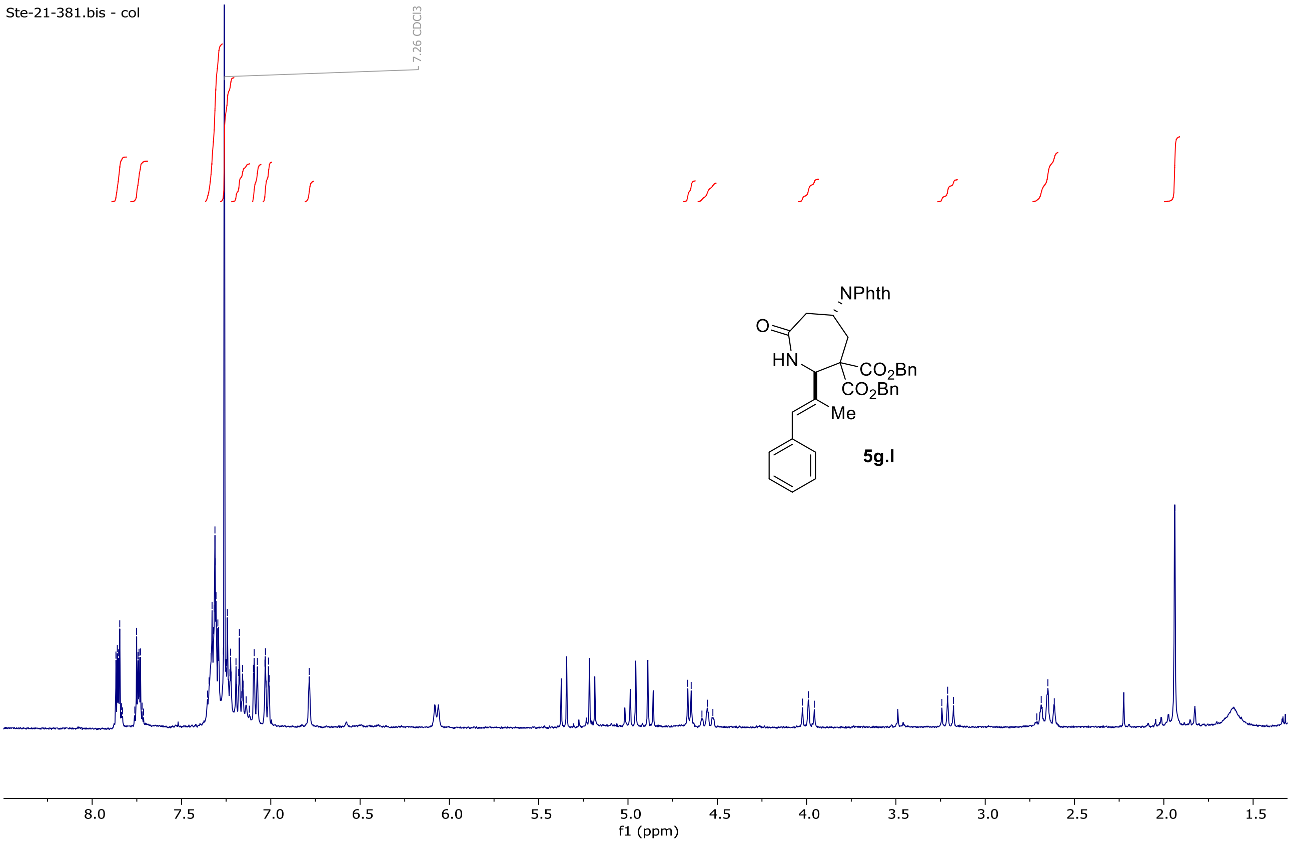

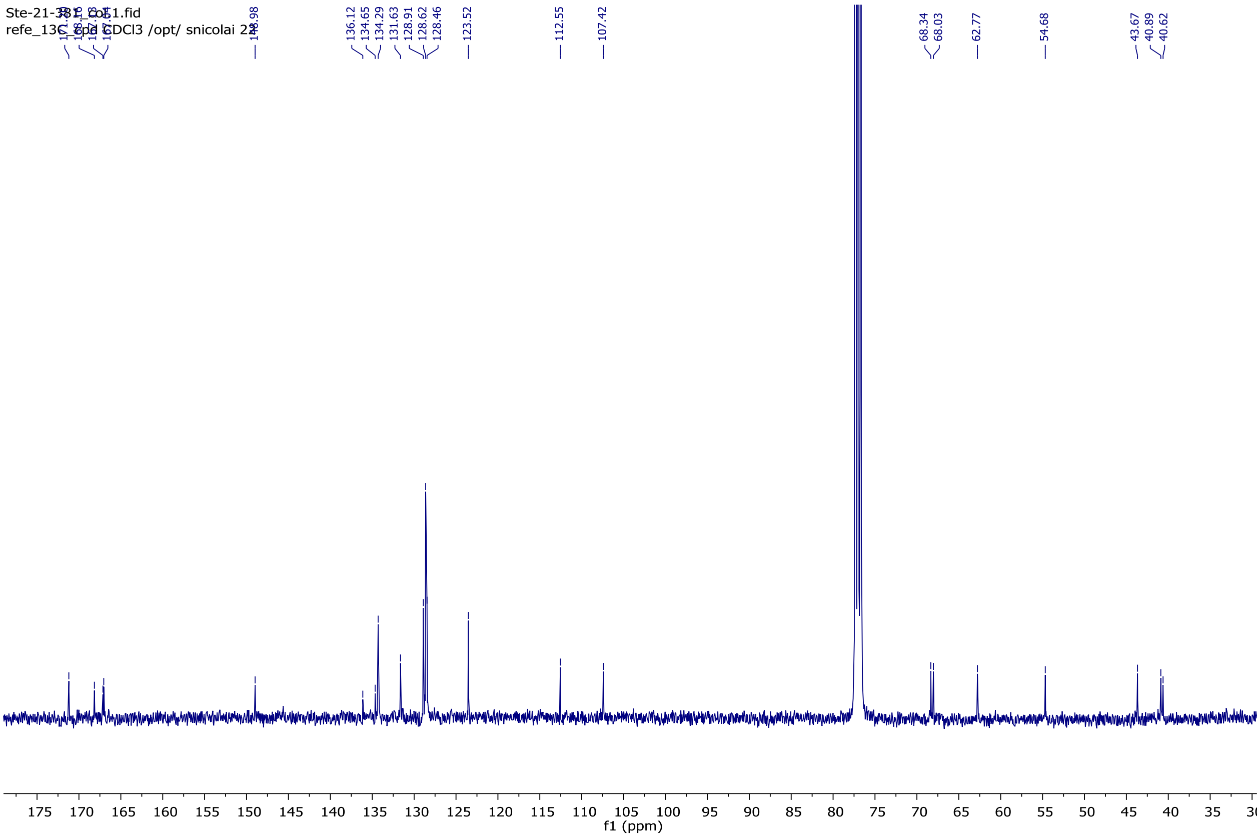

Dibenzyl 2,5-*trans*-5-(3-(4-(*tert*-butyl)benzyl)-5-methyl-2,4-dioxo-3,4-dihydropyrimidin-1(2*H*)-yl)-7-oxo-2-phenylazepane-3,3-dicarboxylate (5a.m)

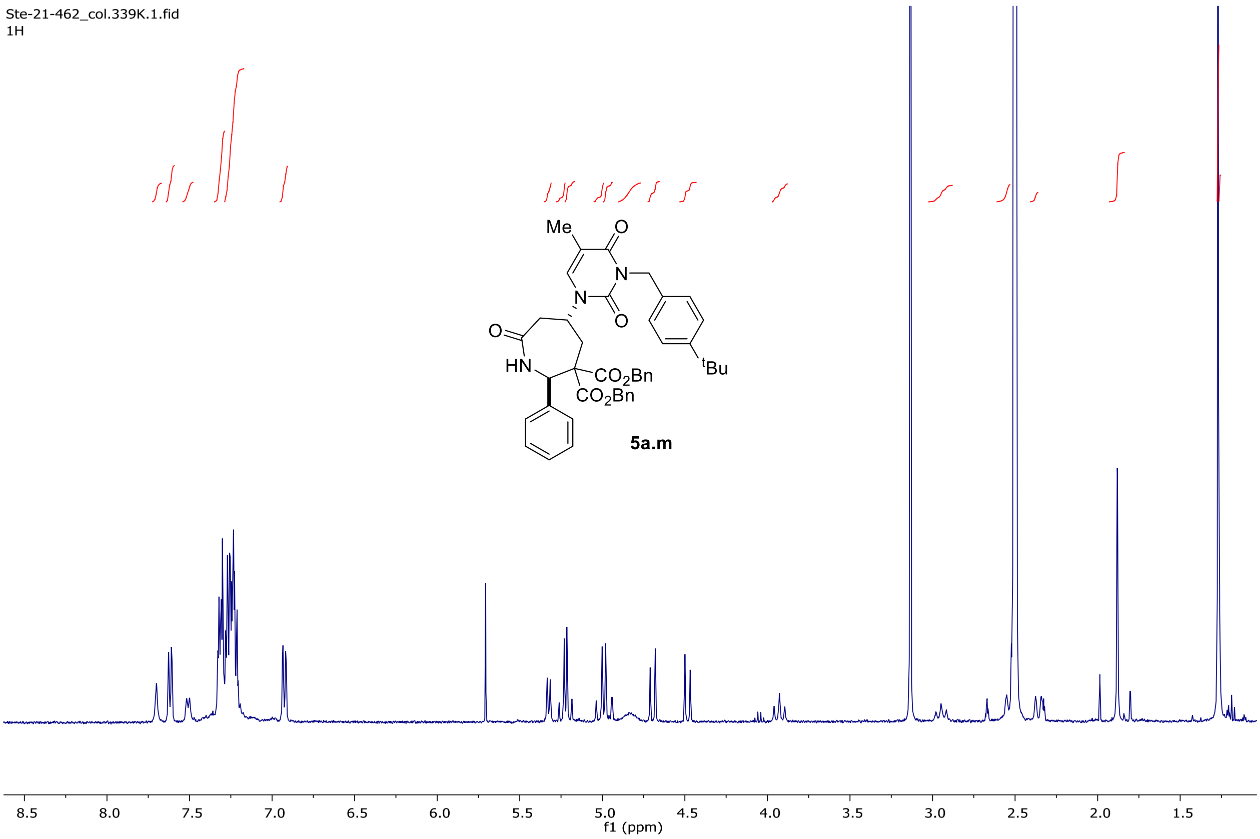

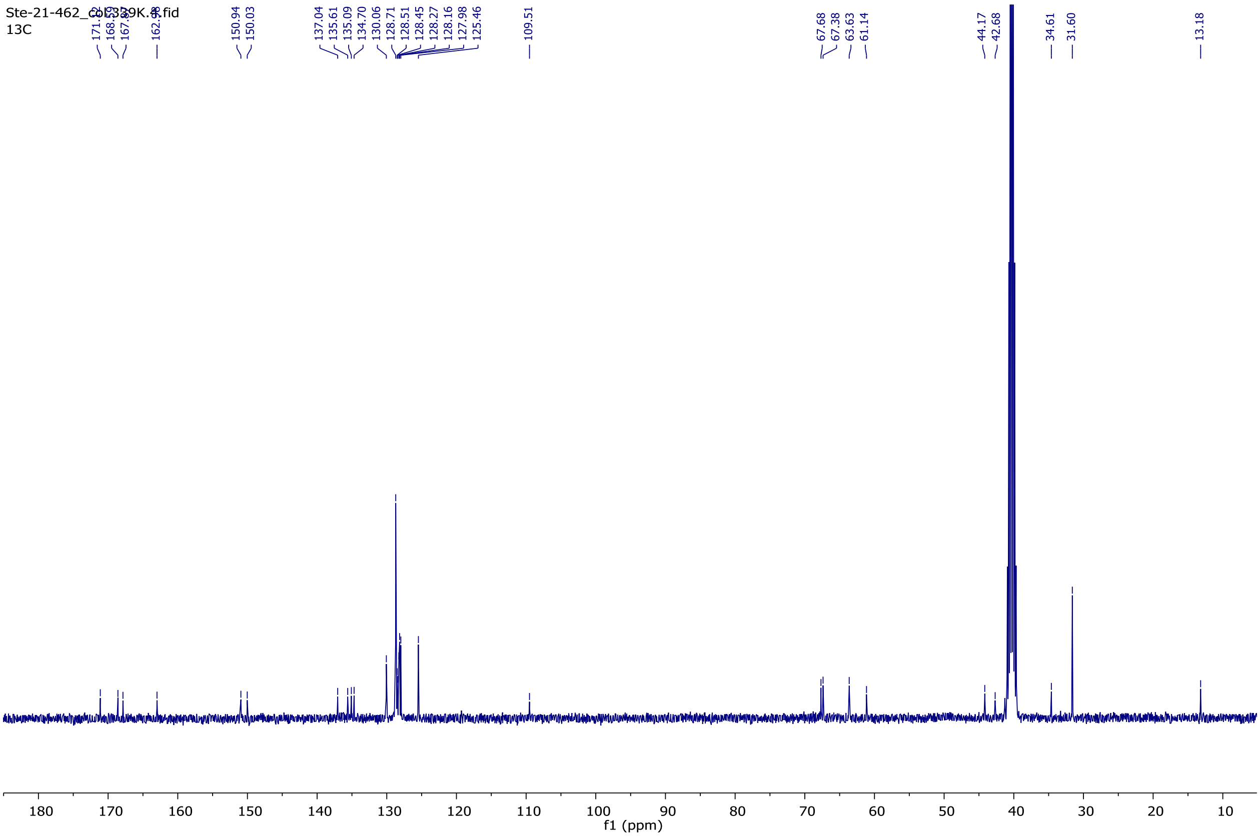

(4S,4'S)-2,2'-(Pentane-3,3-diyl)bis(4-cyclohexyl-4,5-dihydrooxazole) (Et_2-(S)-CyTox) (L4)

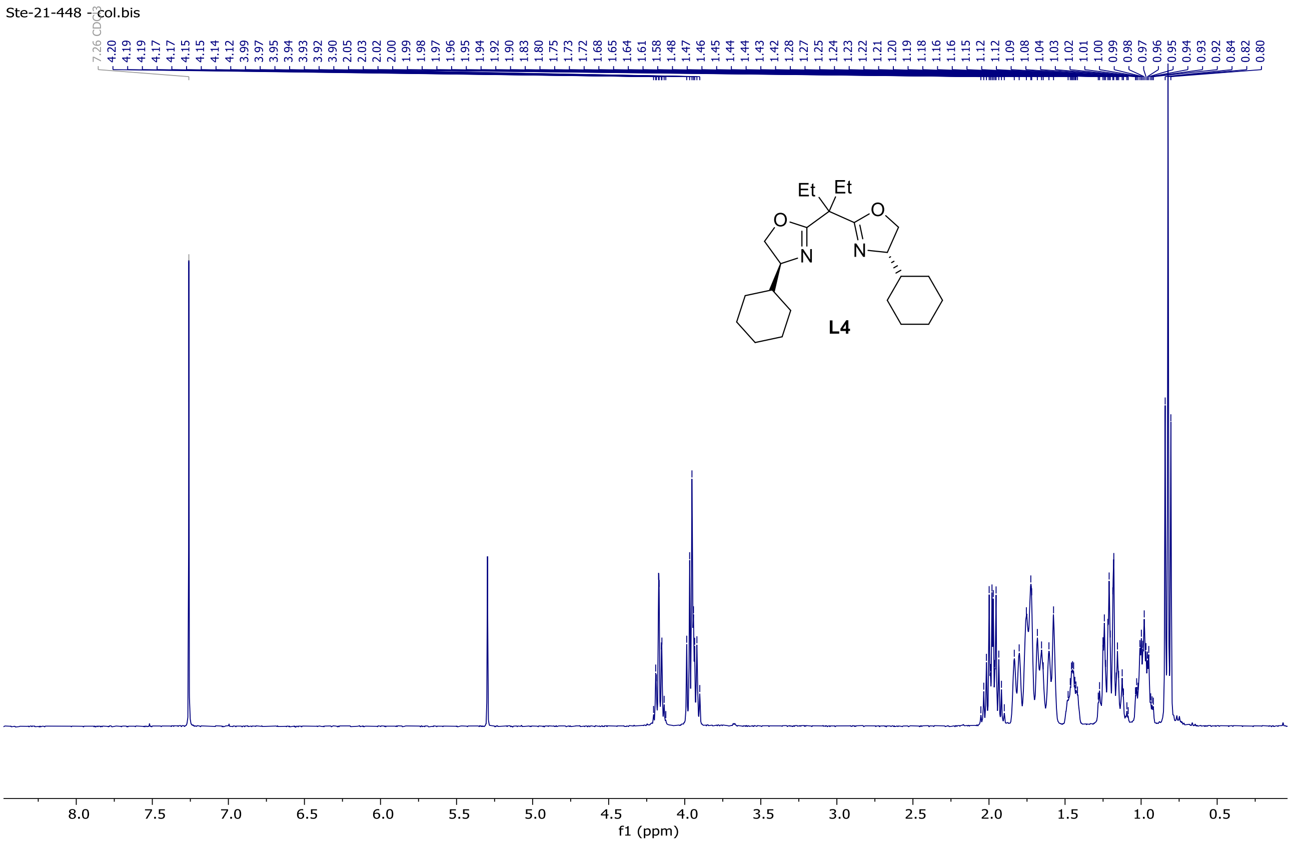

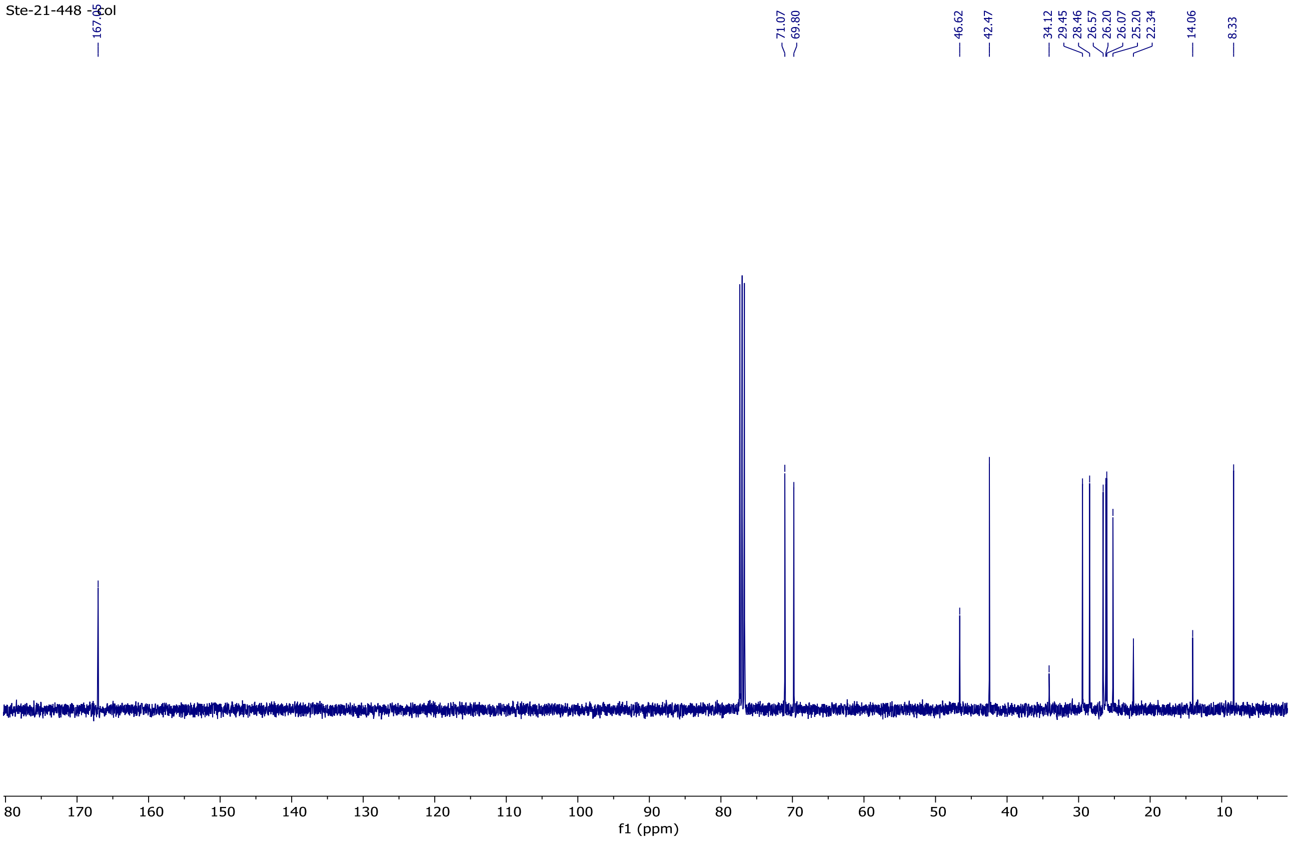

(4S,4'S)-2,2'-(propane-2,2-diyl)bis(4-cyclopentyl-4,5-dihydrooxazole) ((S)-Cyp-Box) (L13)

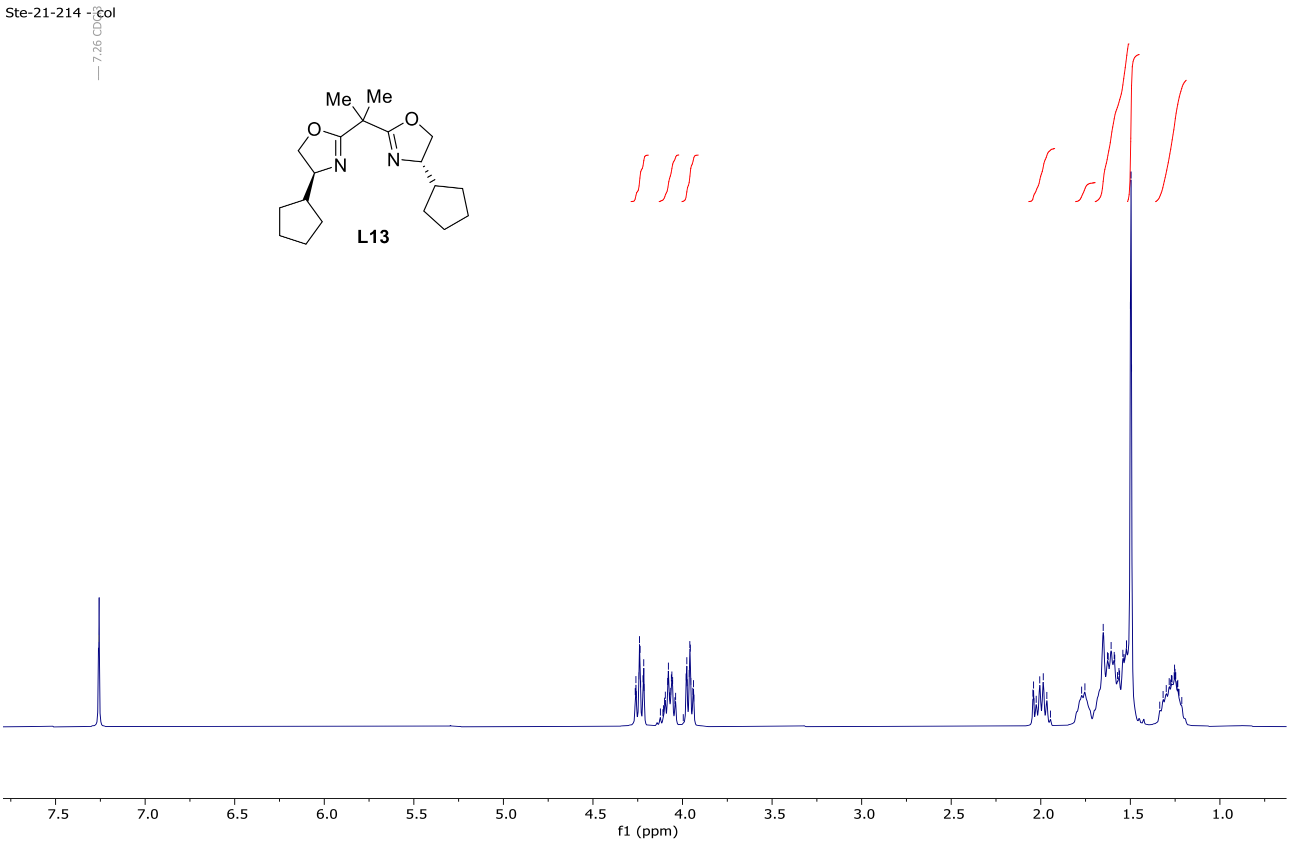

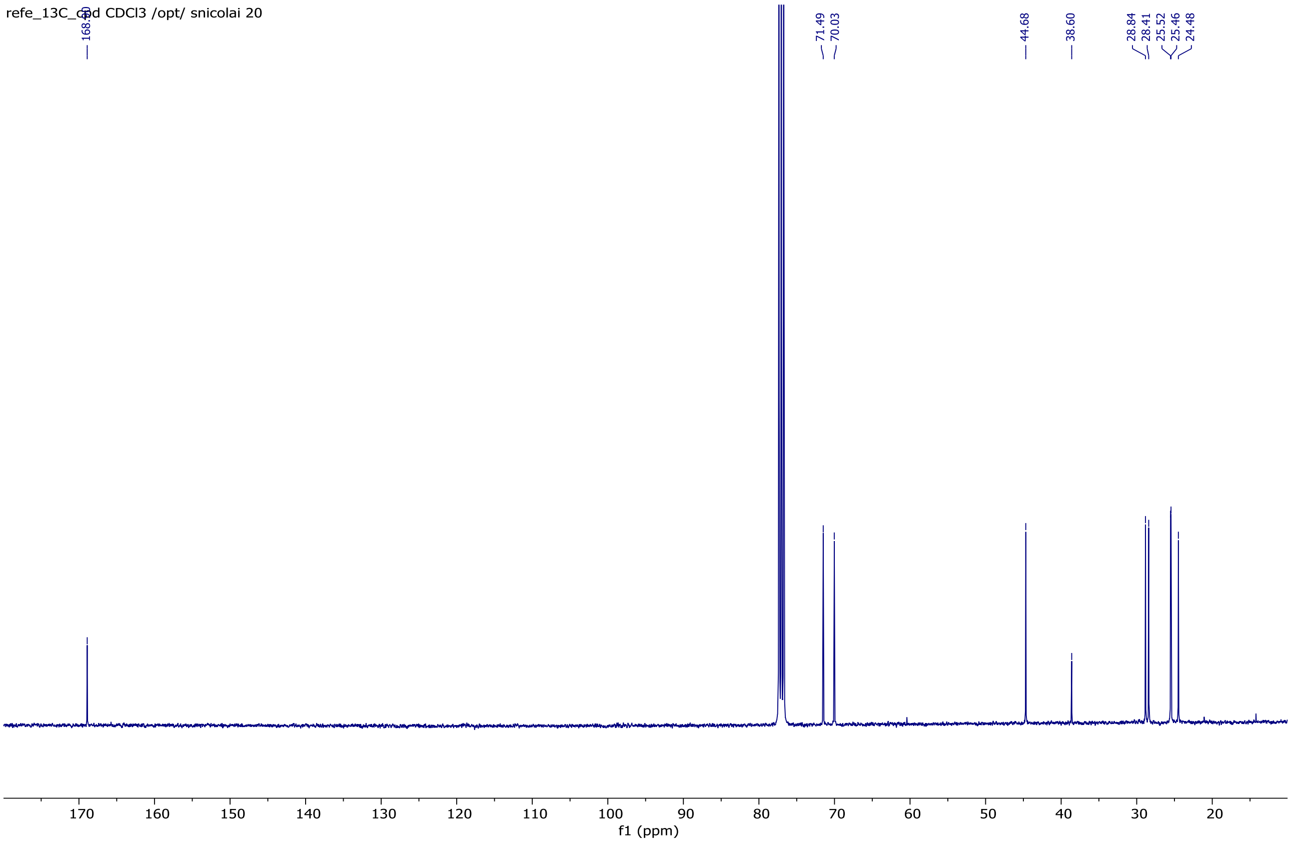

(4S,4'S,4''S)-2,2',2''-(Propane-1,2,2-triyl)tris(4-benzyl-4,5-dihydrooxazole) ((S)-BnTox) (L14)

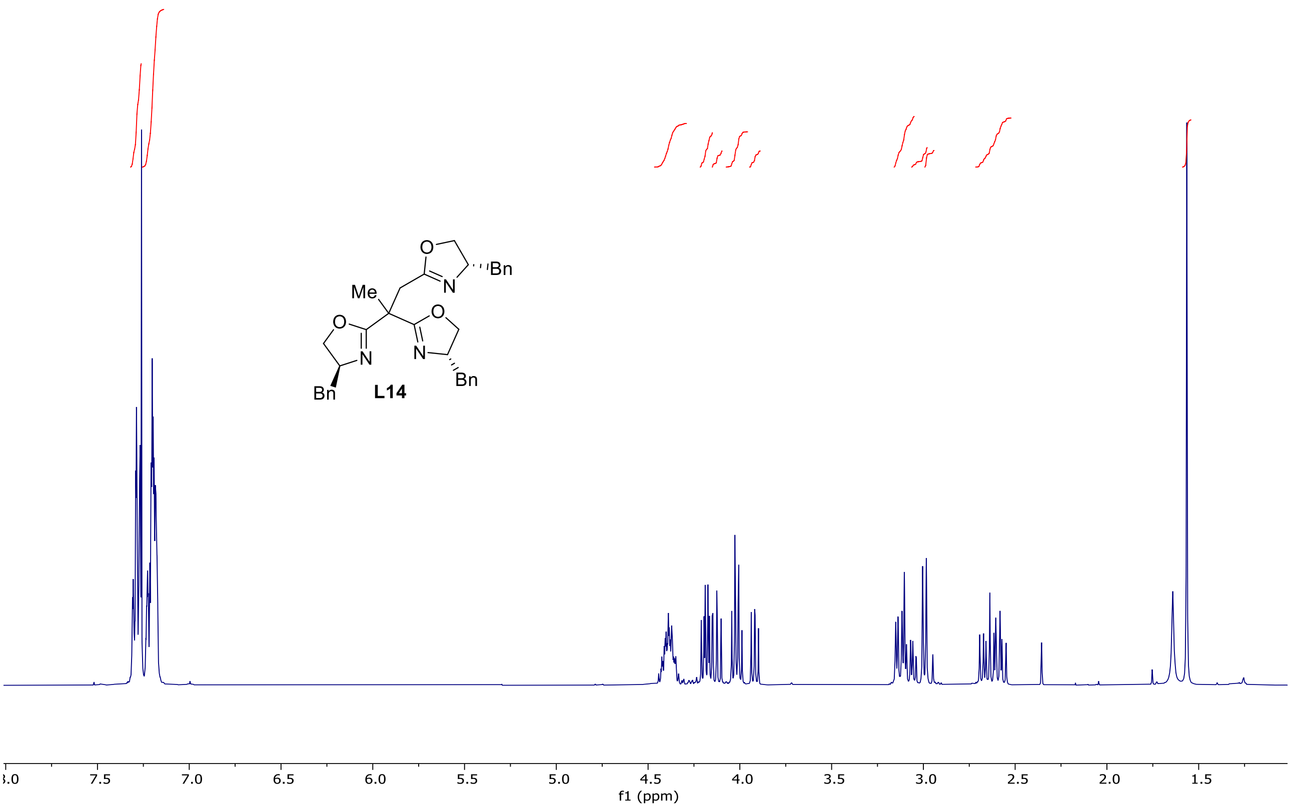

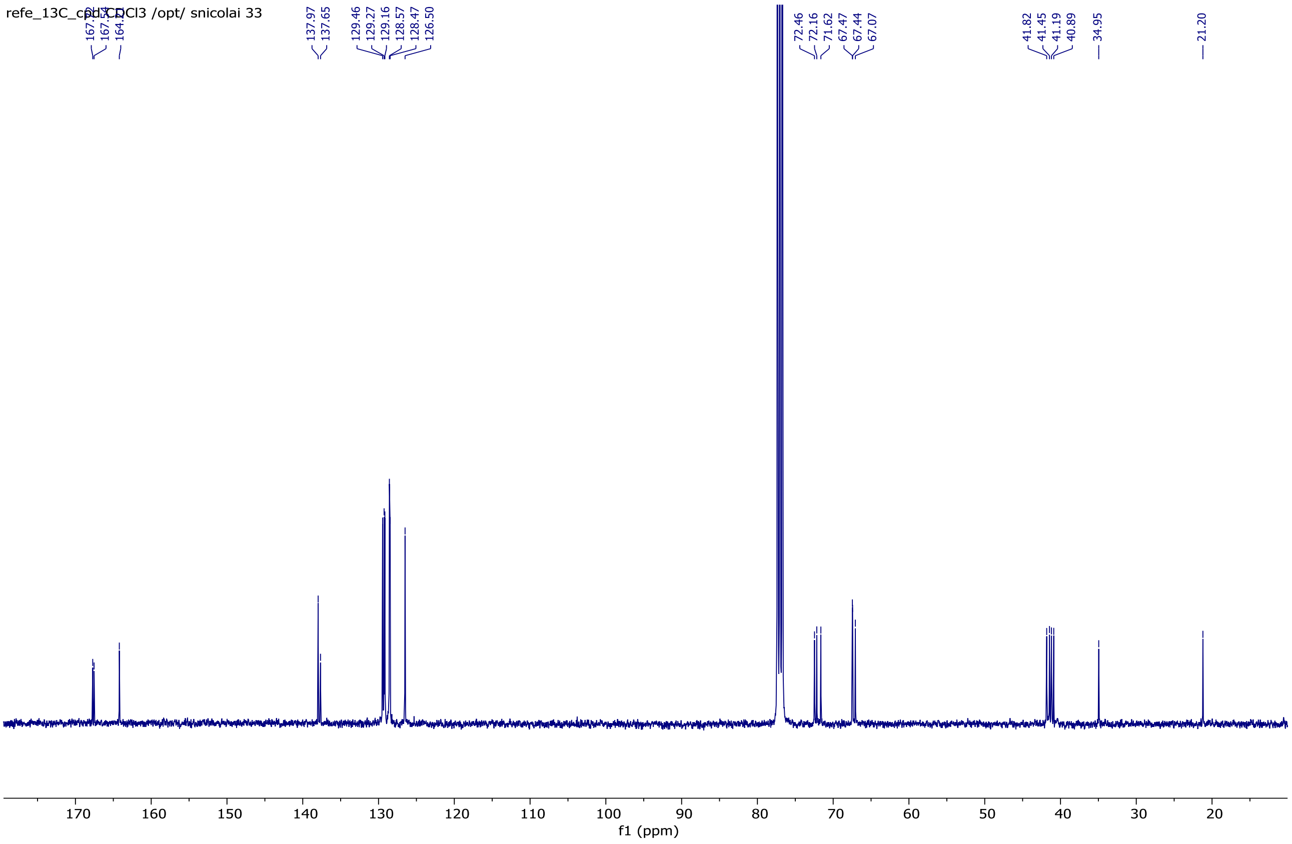

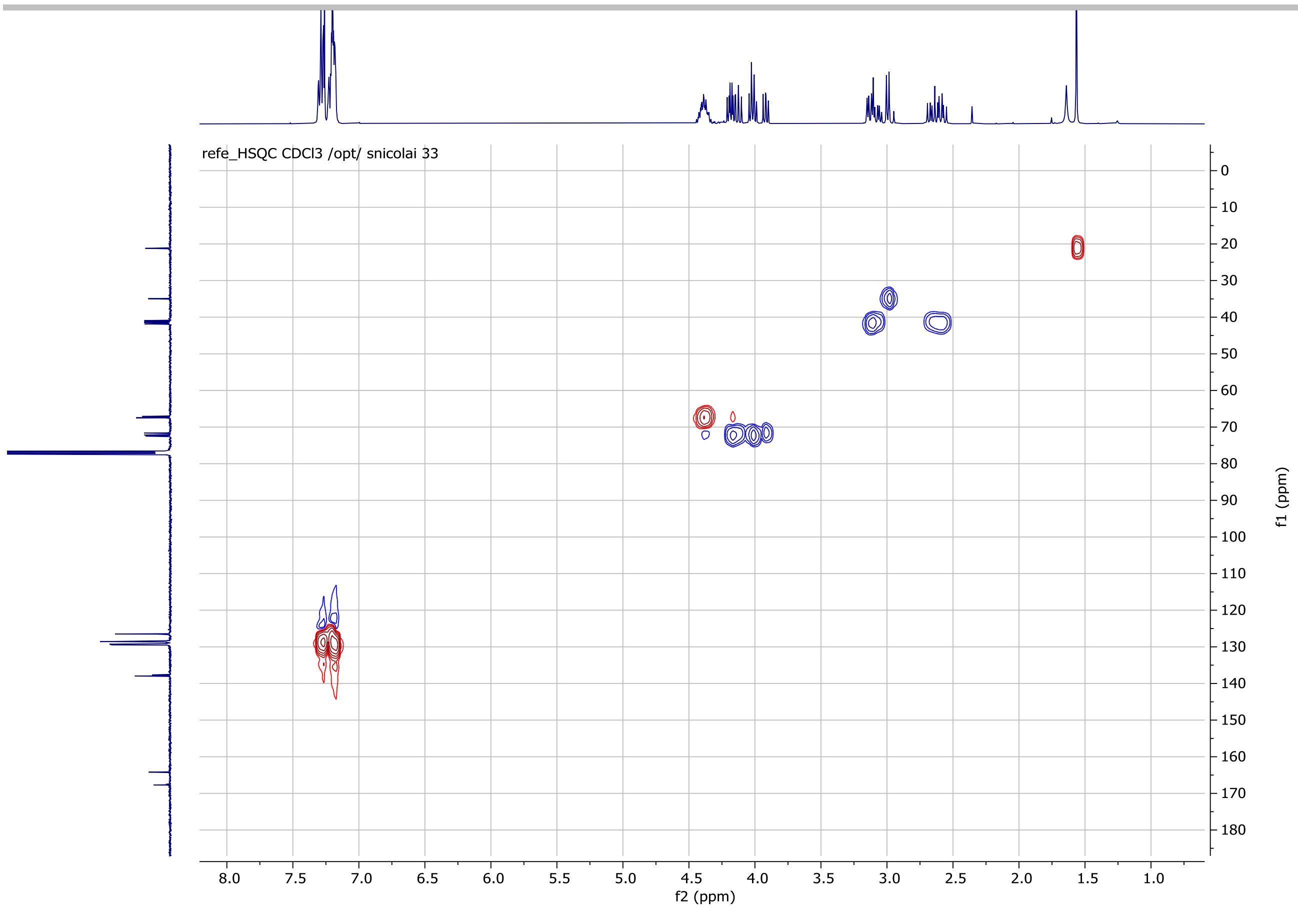
refe_HSQC CDCl3 /opt/ snicolai 33
f2 (ppm)
f1 (ppm)

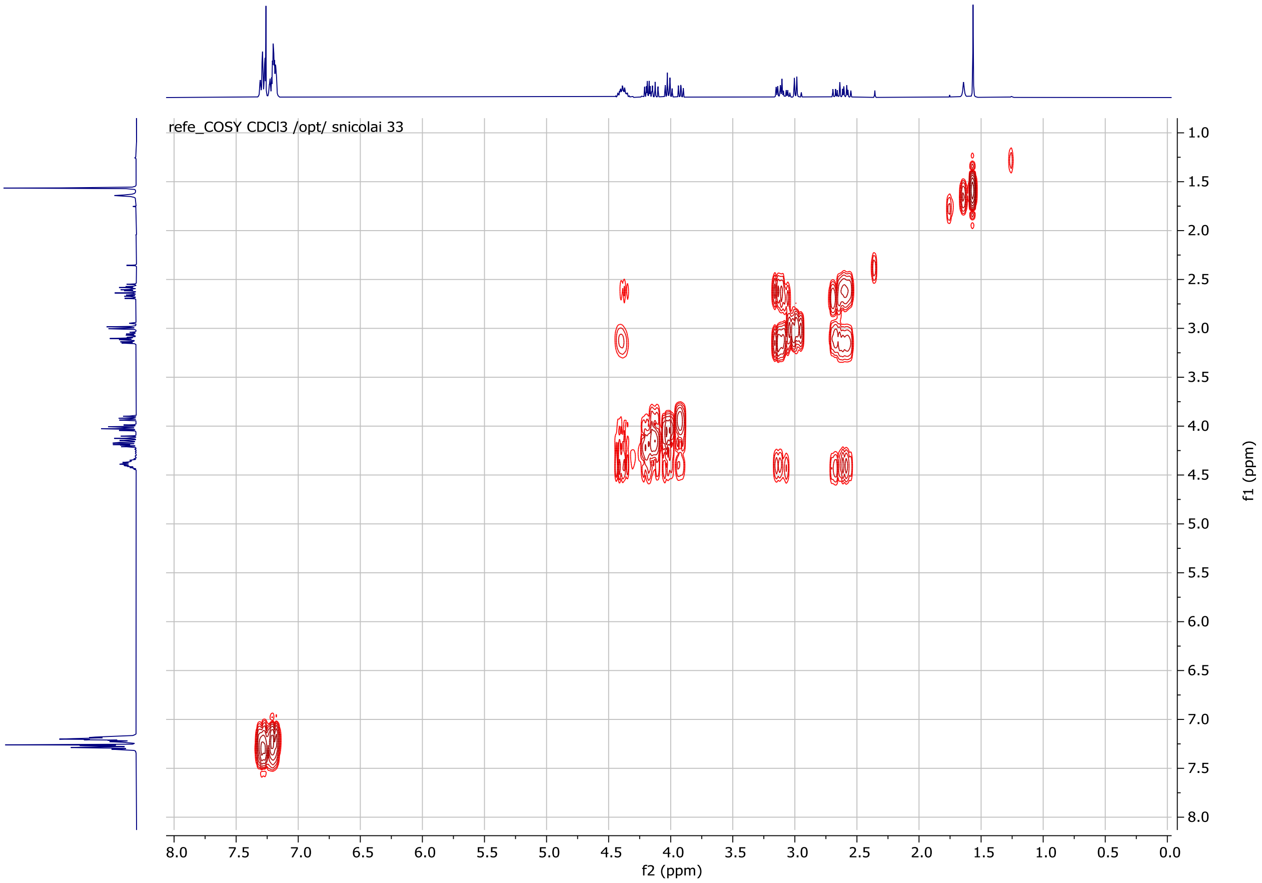
refe_COSY CDCl3 /opt/ snicolai 33
f2 (ppm)
f1 (ppm)

5-(4-methoxyphenyl)-7-oxo-2-phenylazepane-3-carboxylic acid (6)

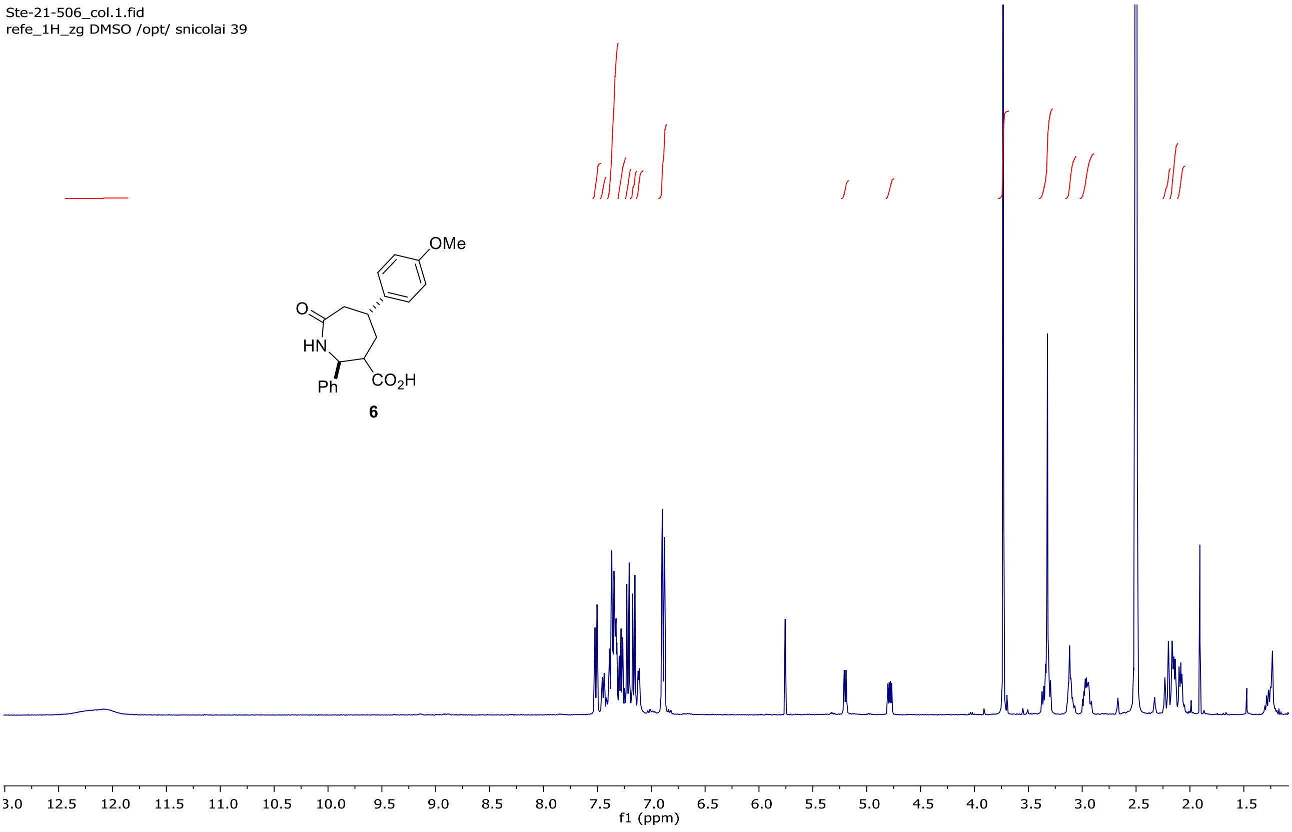

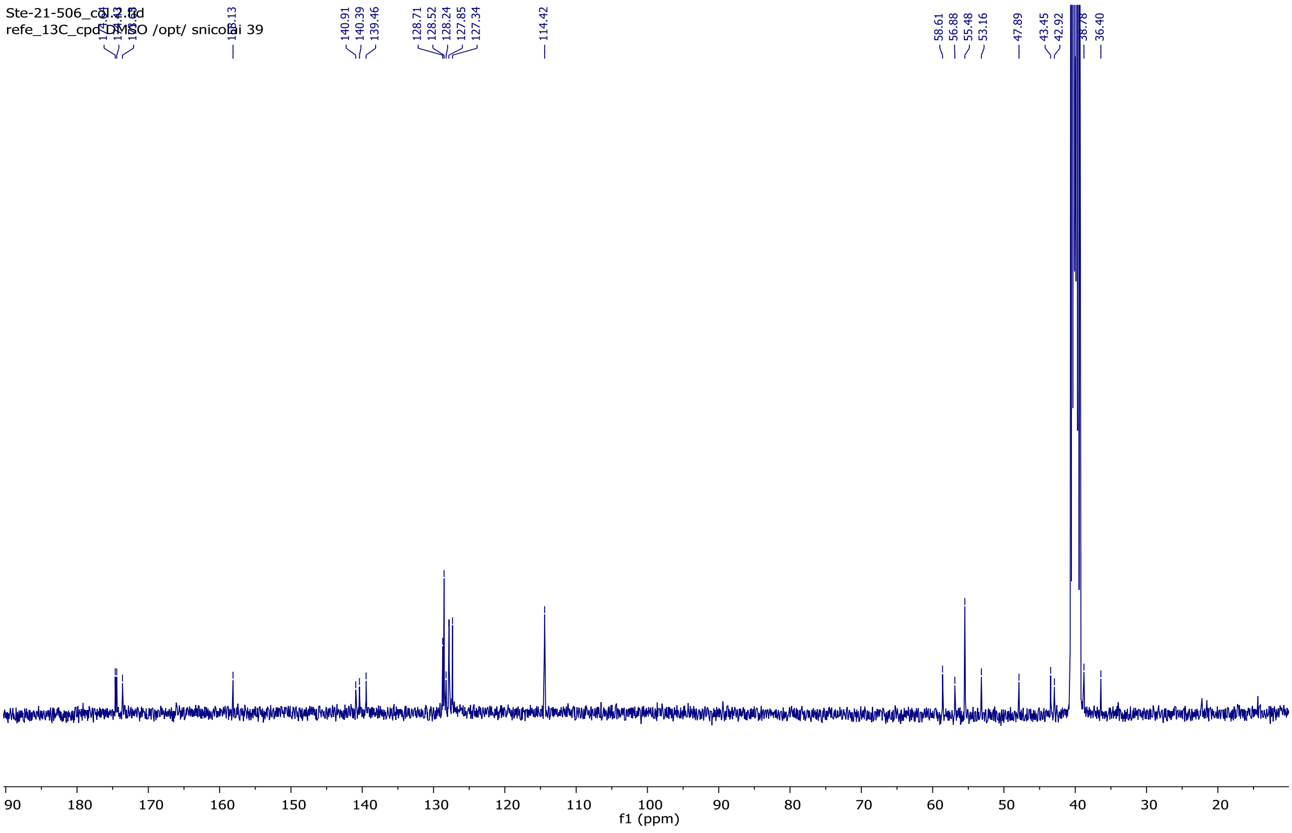

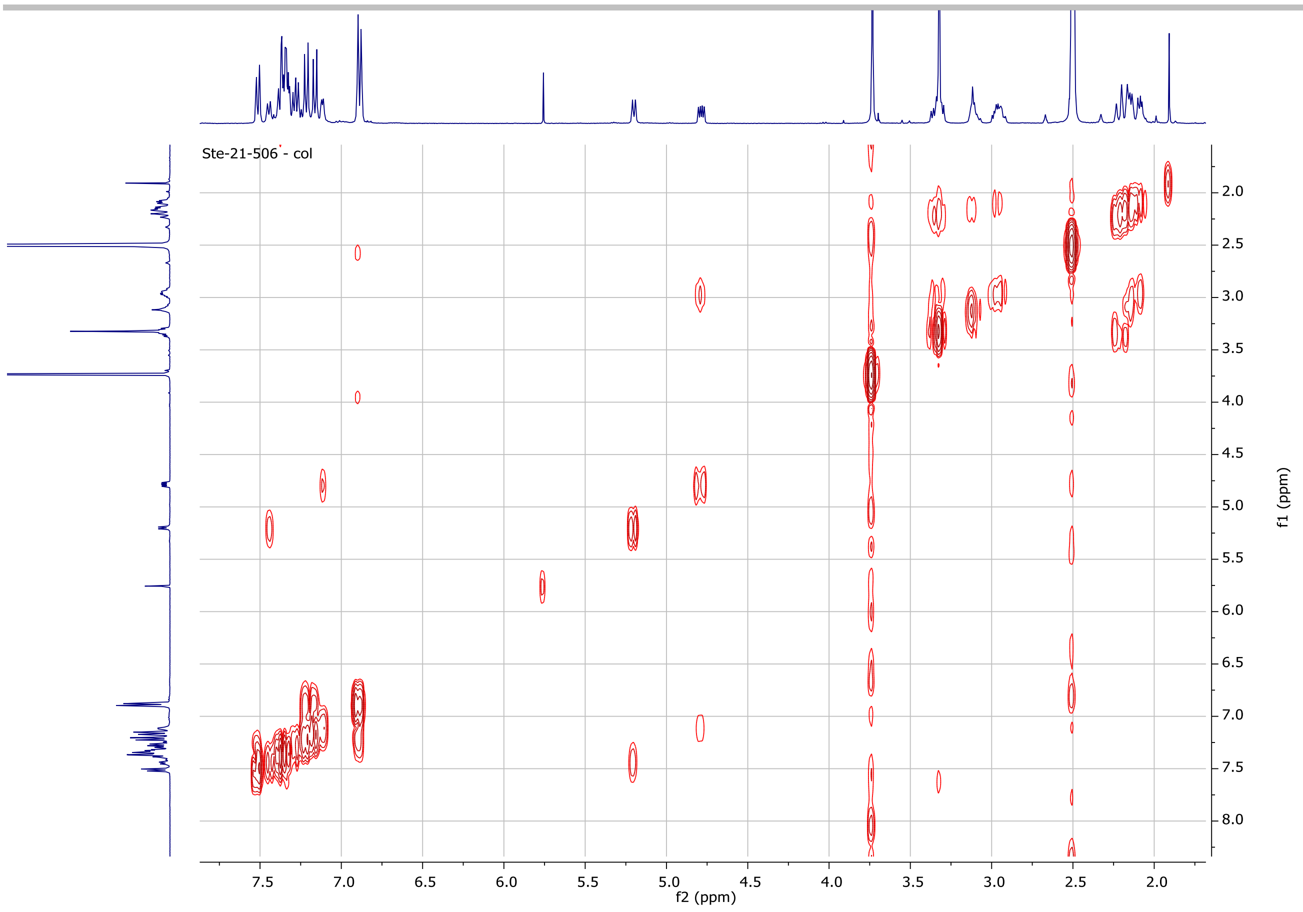
Ste-21-506 - col
f2 (ppm)
f1 (ppm)

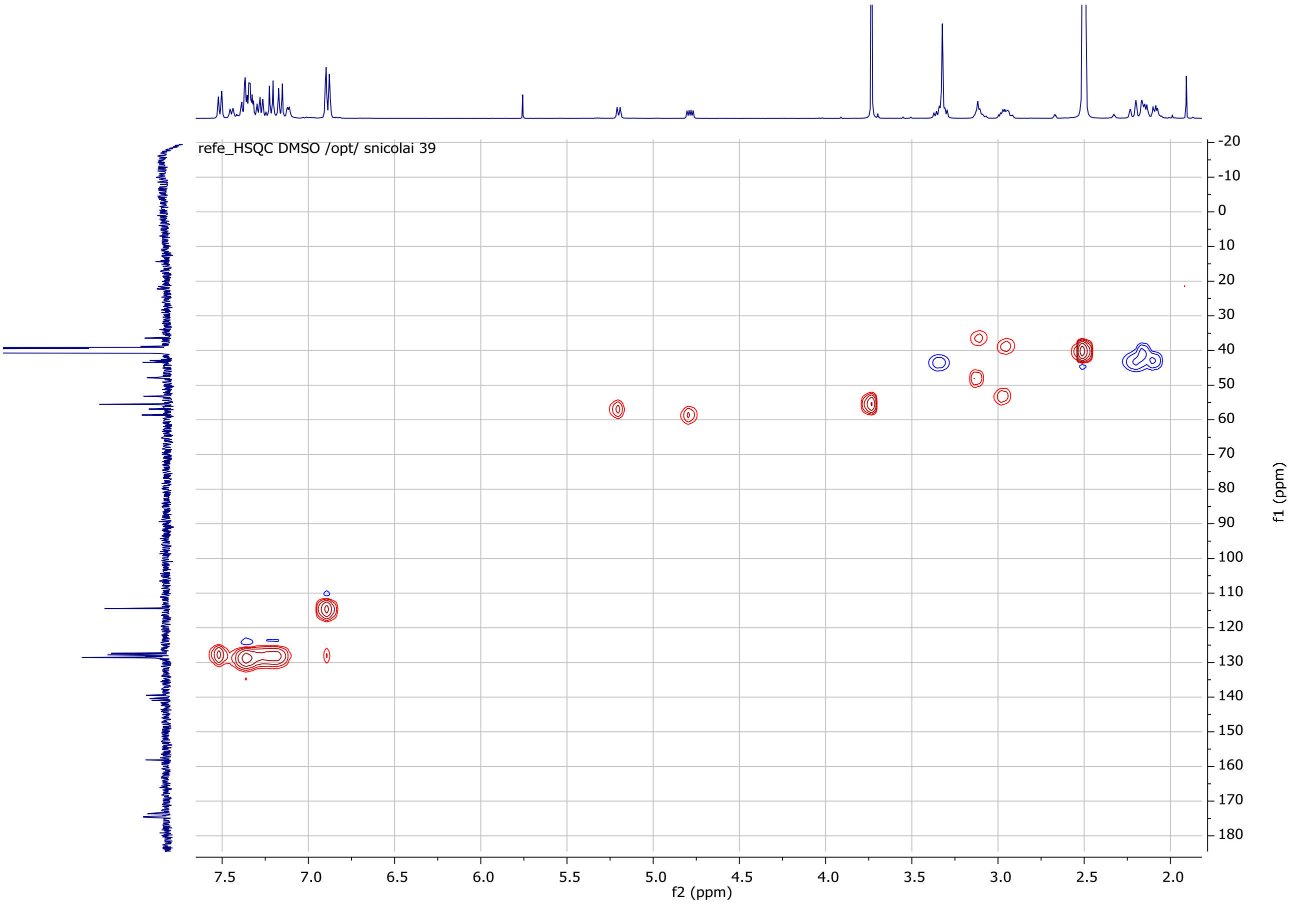
refe_HSQC DMSO /opt/ snicolai 39
f2 (ppm)
f1 (ppm)

4-(4-Methoxyphenyl)-7-phenyl-6-(phenylethynyl)azepan-2-one (7)

4-(4-Methoxyphenyl)-7-phenyl-6-(phenylethynyl)azepan-2-one (XX).1.fid
1H

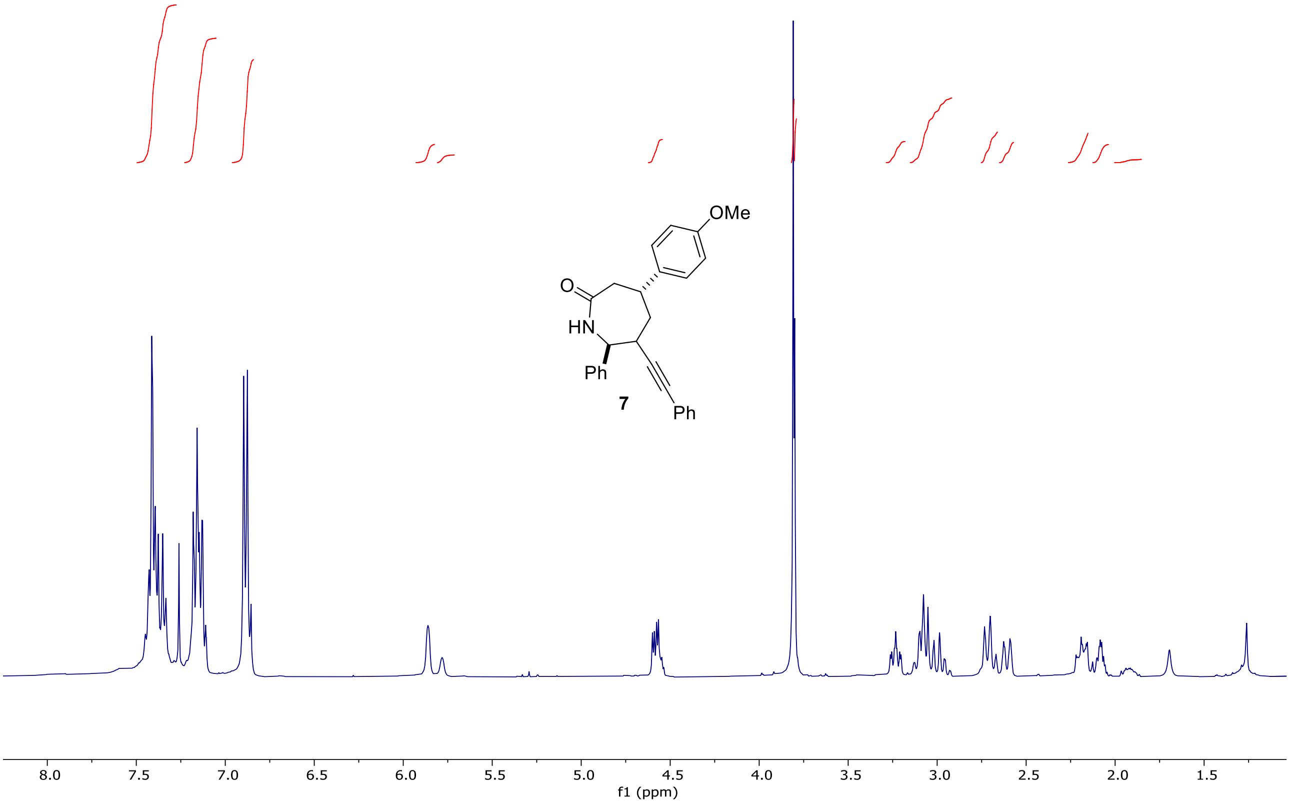

4-(4-Methoxyphenyl)-7-phenyl-6-(phenylethynyl)azepan-2-one (XX).2.fid
13C

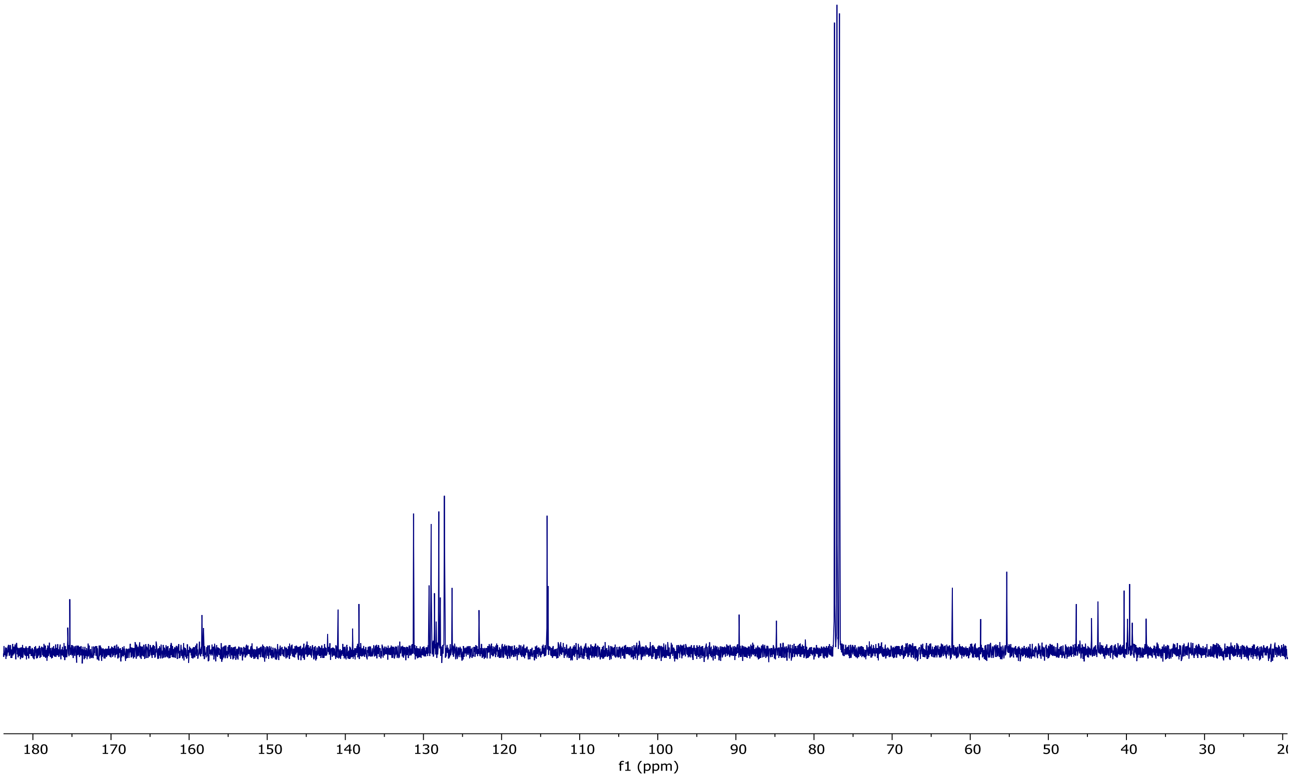

4,7-*trans*-4-(4-Methoxyphenyl)-7-phenylazepan-2-one (8)

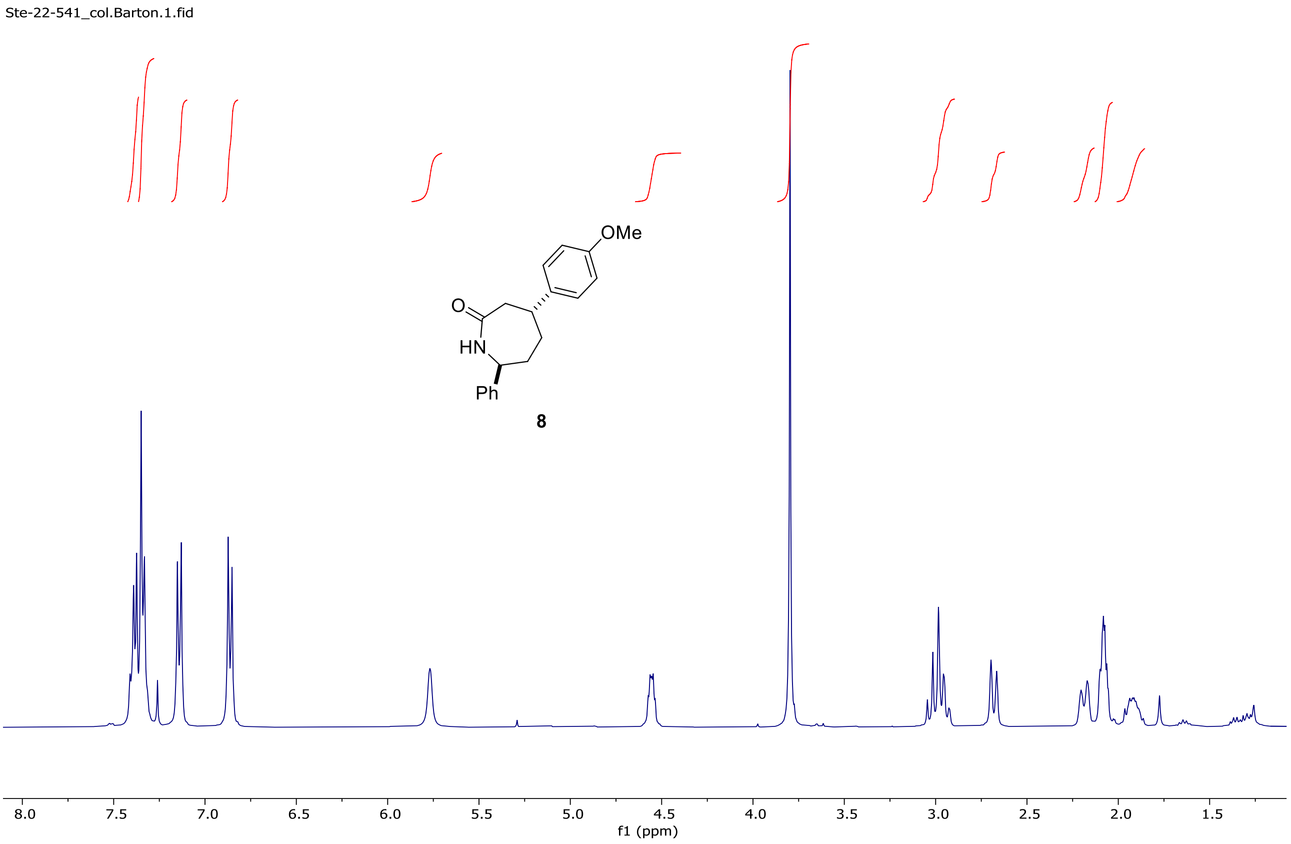

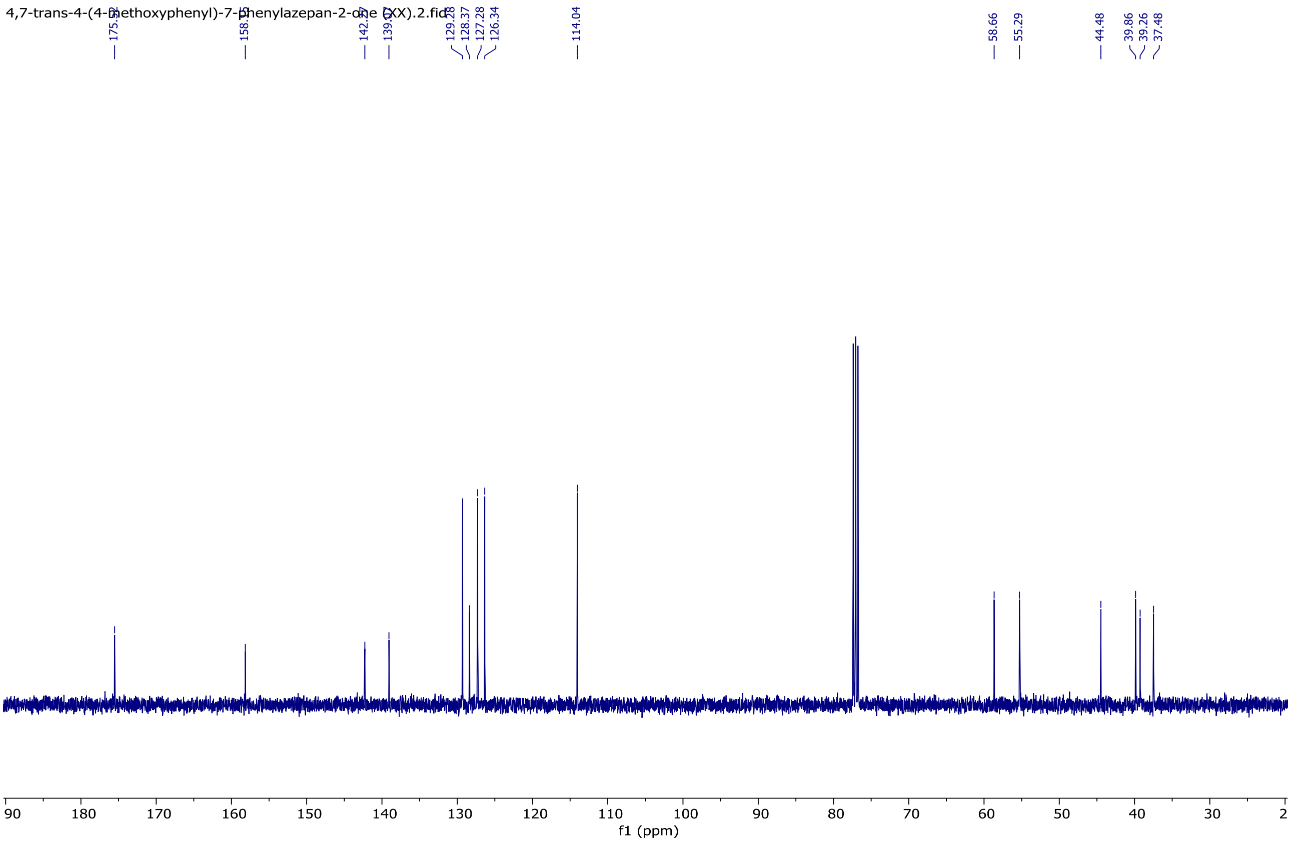

Dibenzyl 2-(4-(benzyl(methyl)amino)-2-(4-methoxyphenyl)butyl)malonate (S94)

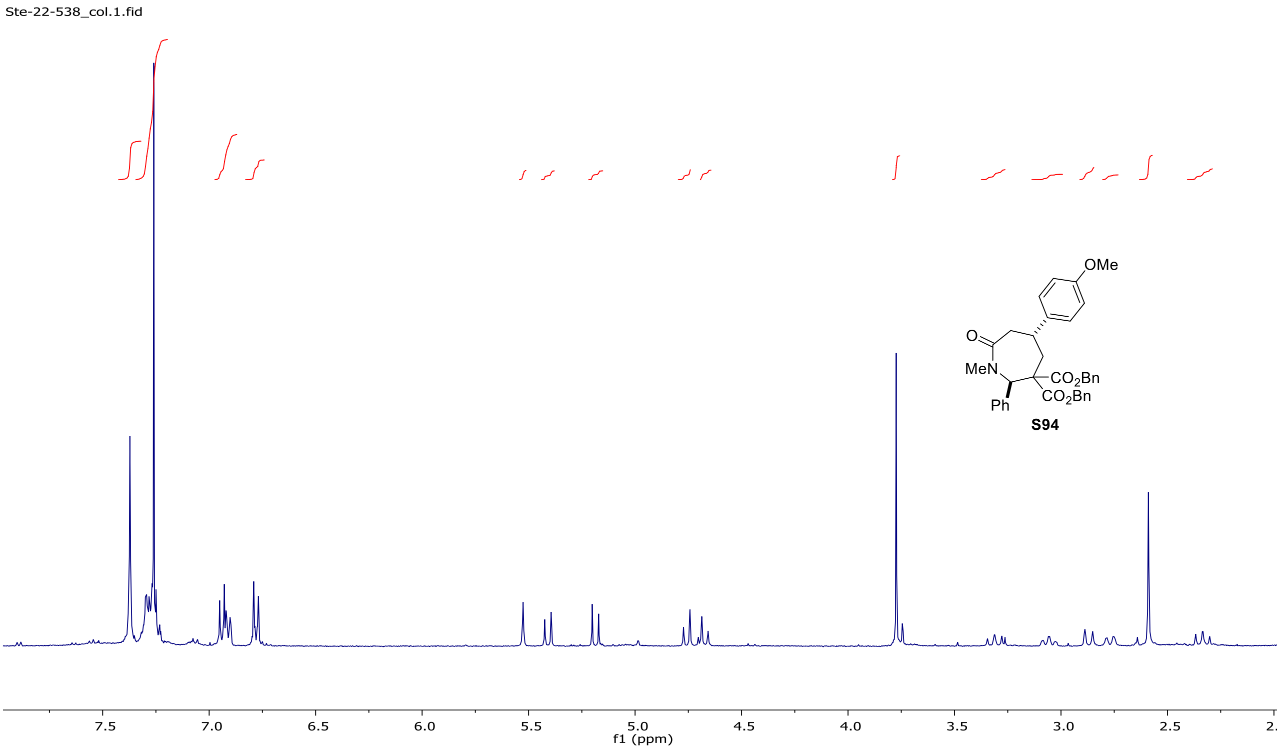

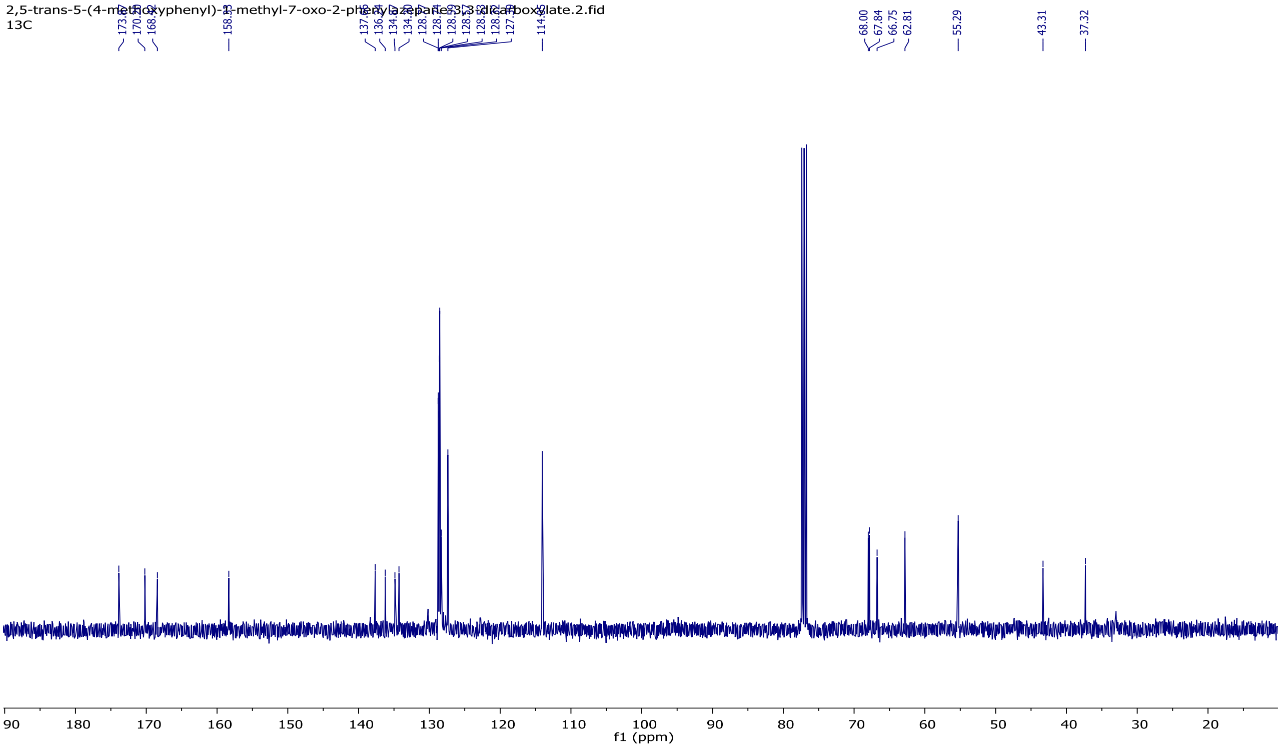

Dibenzyl 2-(4-(benzyl(methyl)amino)-2-(4-methoxyphenyl)butyl)malonate (9)

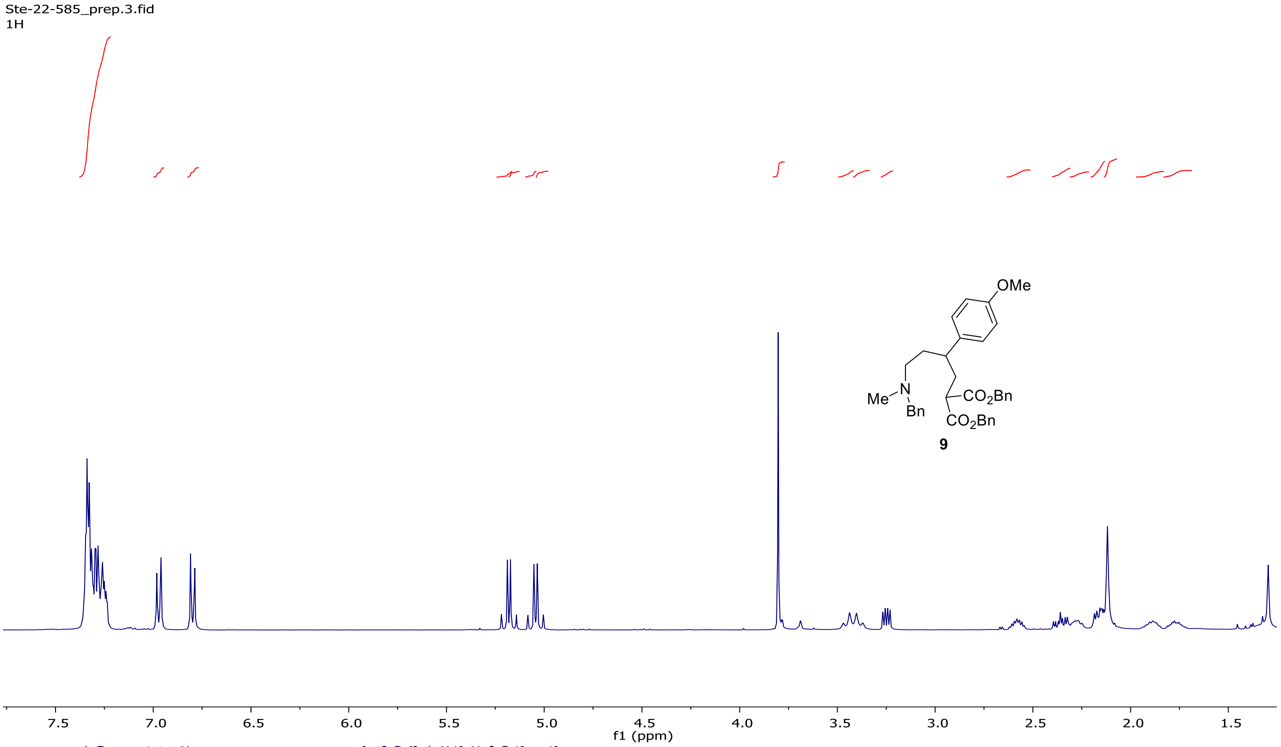

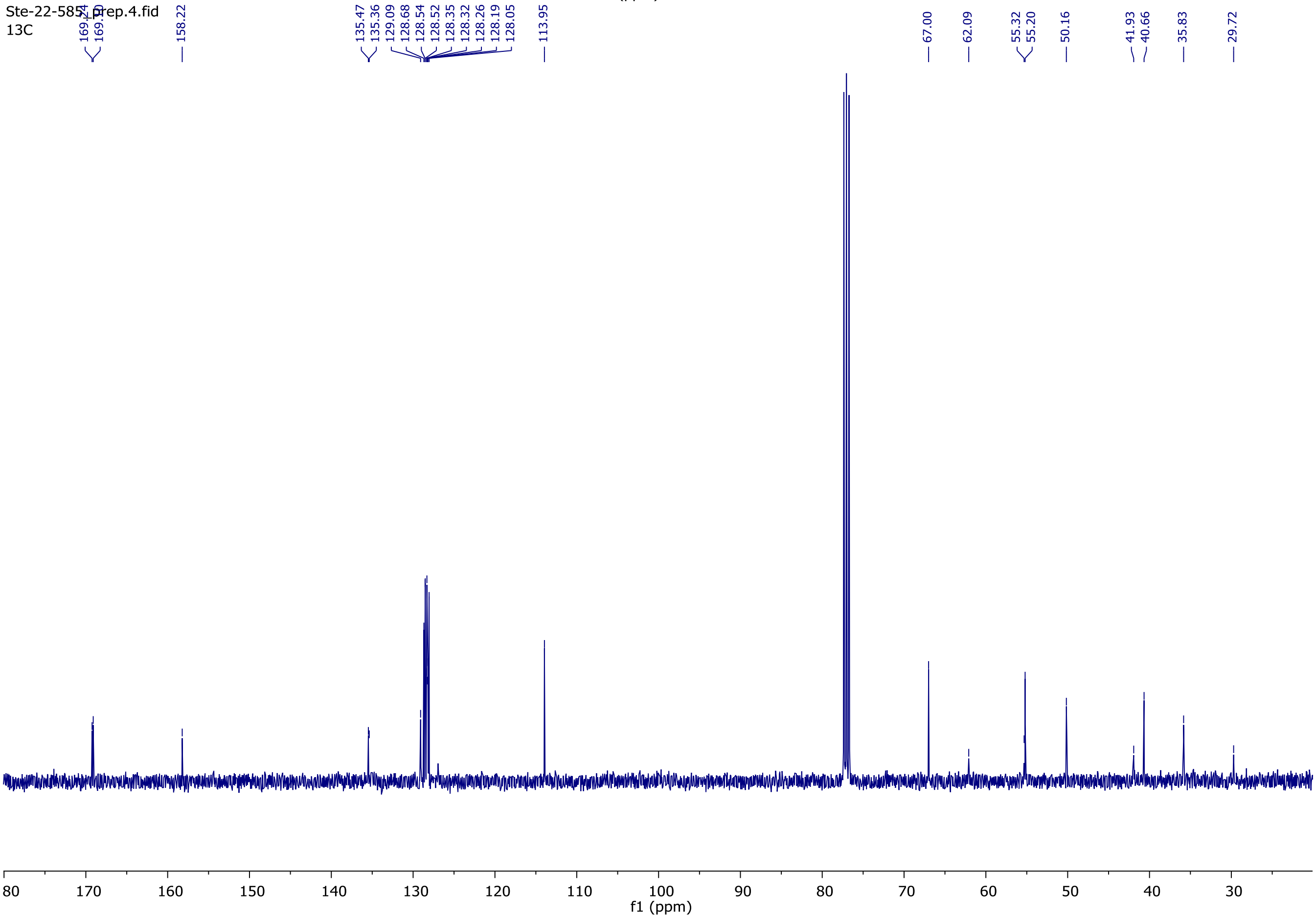

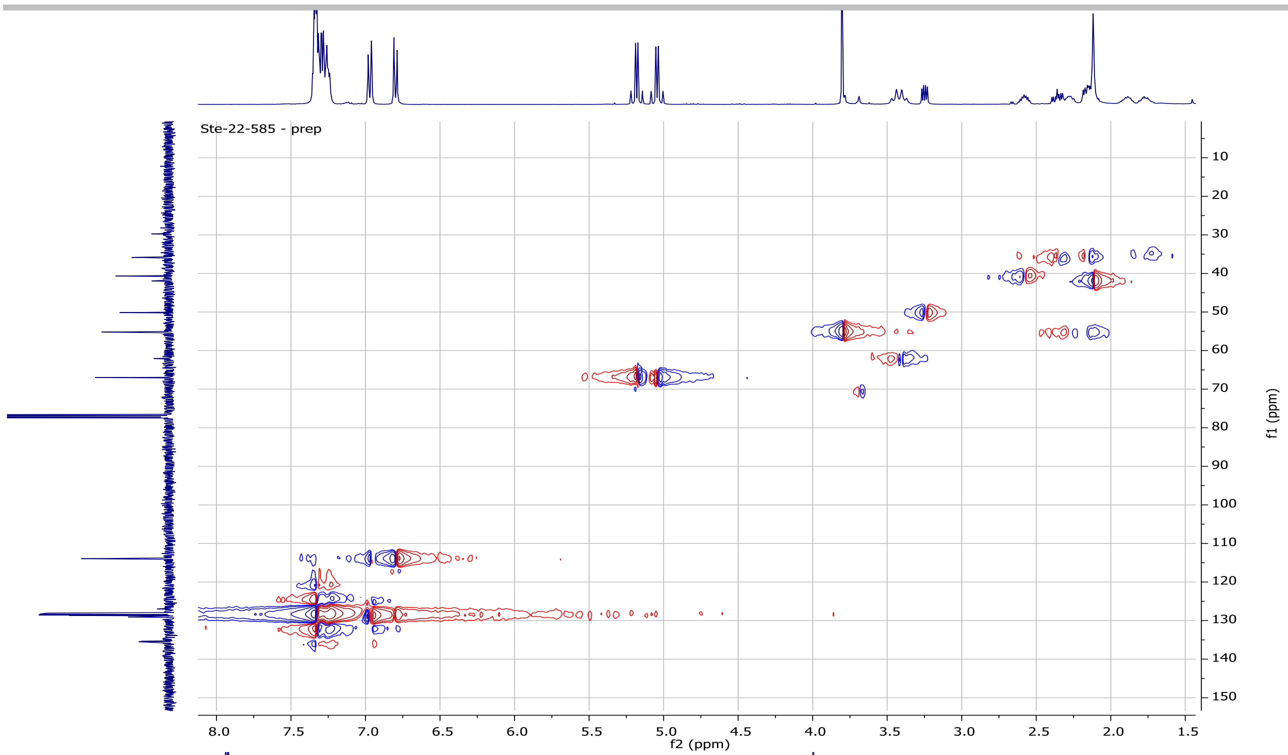
Ste-22-585 - prep
f2 (ppm)
f1 (ppm)

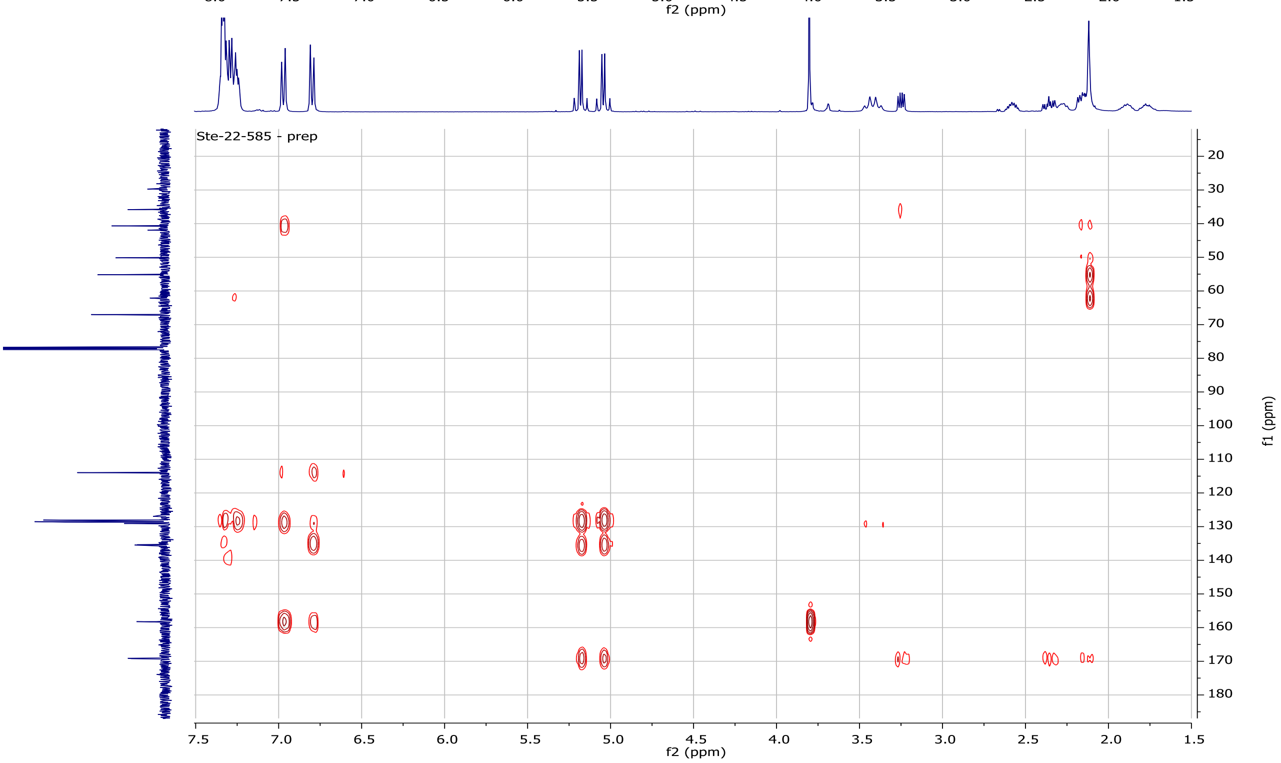
Ste-22-585 - prep
f2 (ppm)
f1 (ppm)

2,5-*trans*-5-(4-methoxyphenyl)-2-phenylazepane (10)

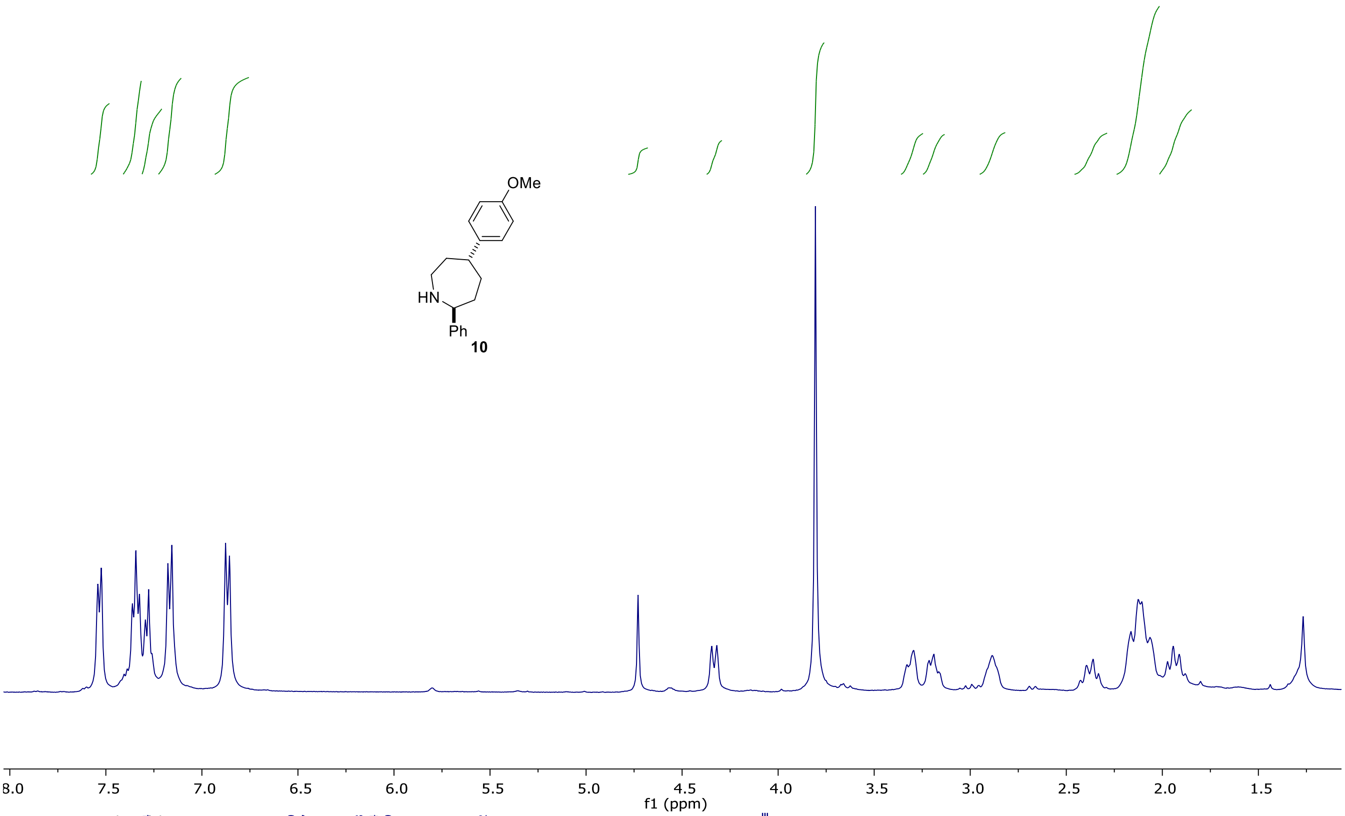

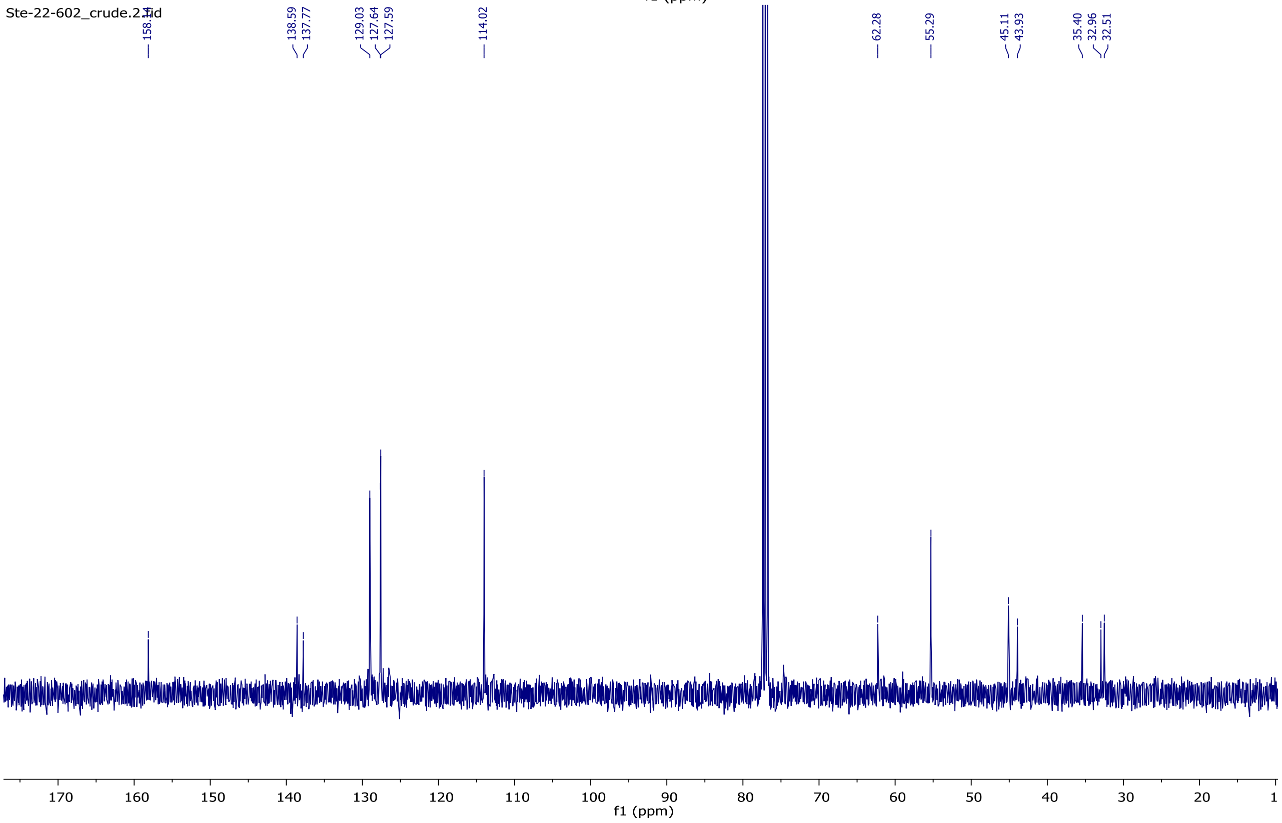

Benzyl-4,8-dioxo-2-phenyl-3,7-diazabicyclo[4.2.1]nonane-1-carboxylate (11)

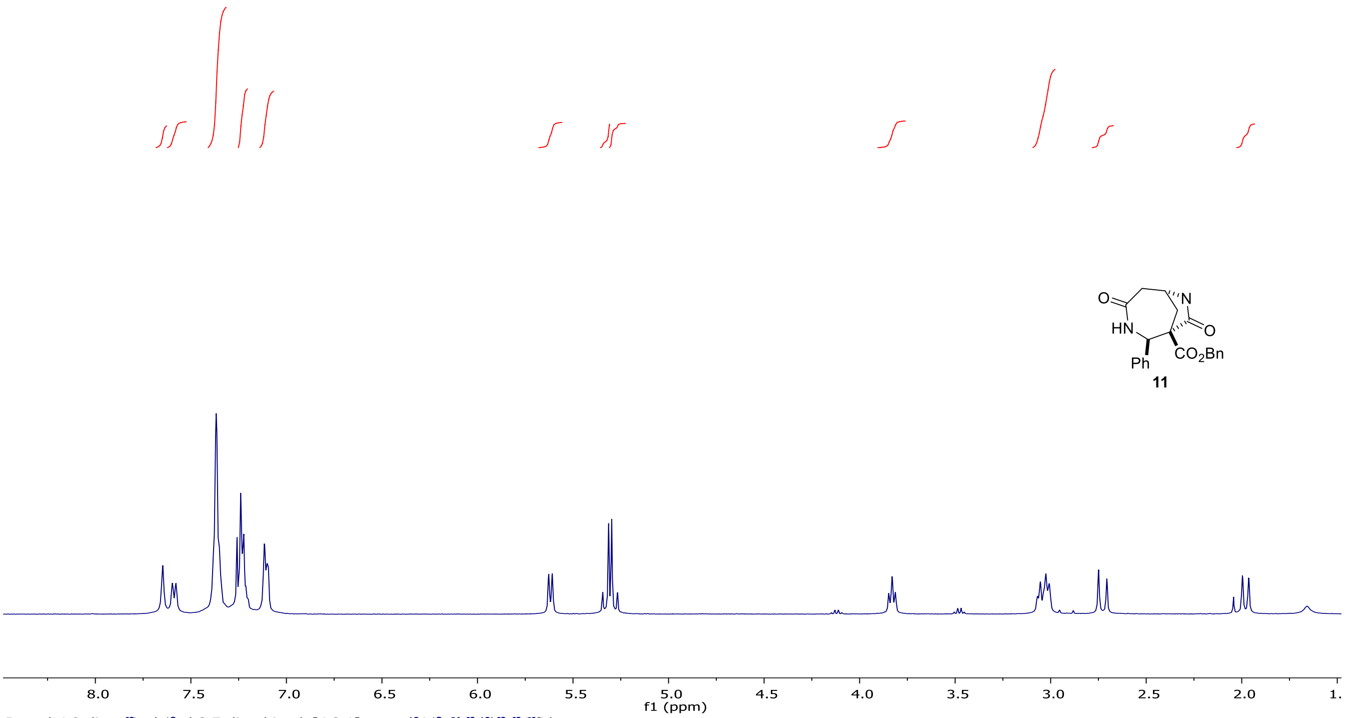

11

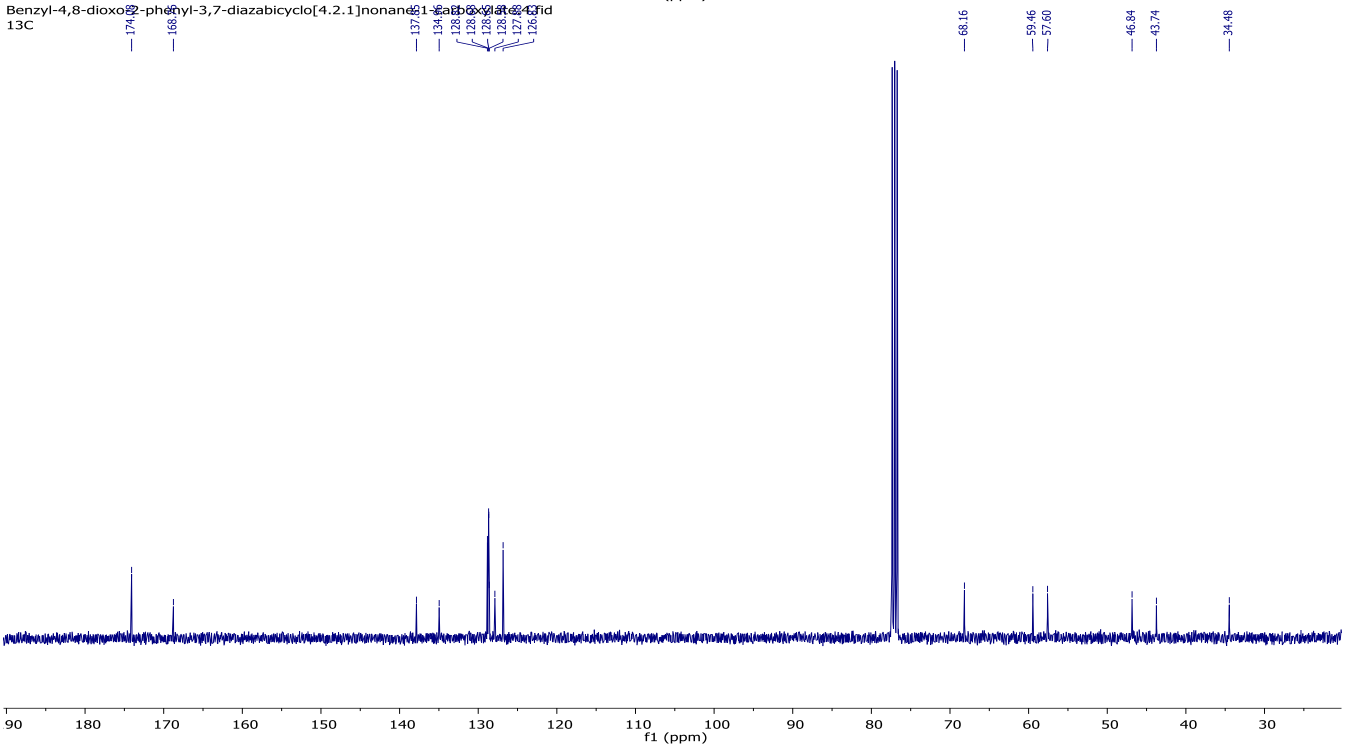

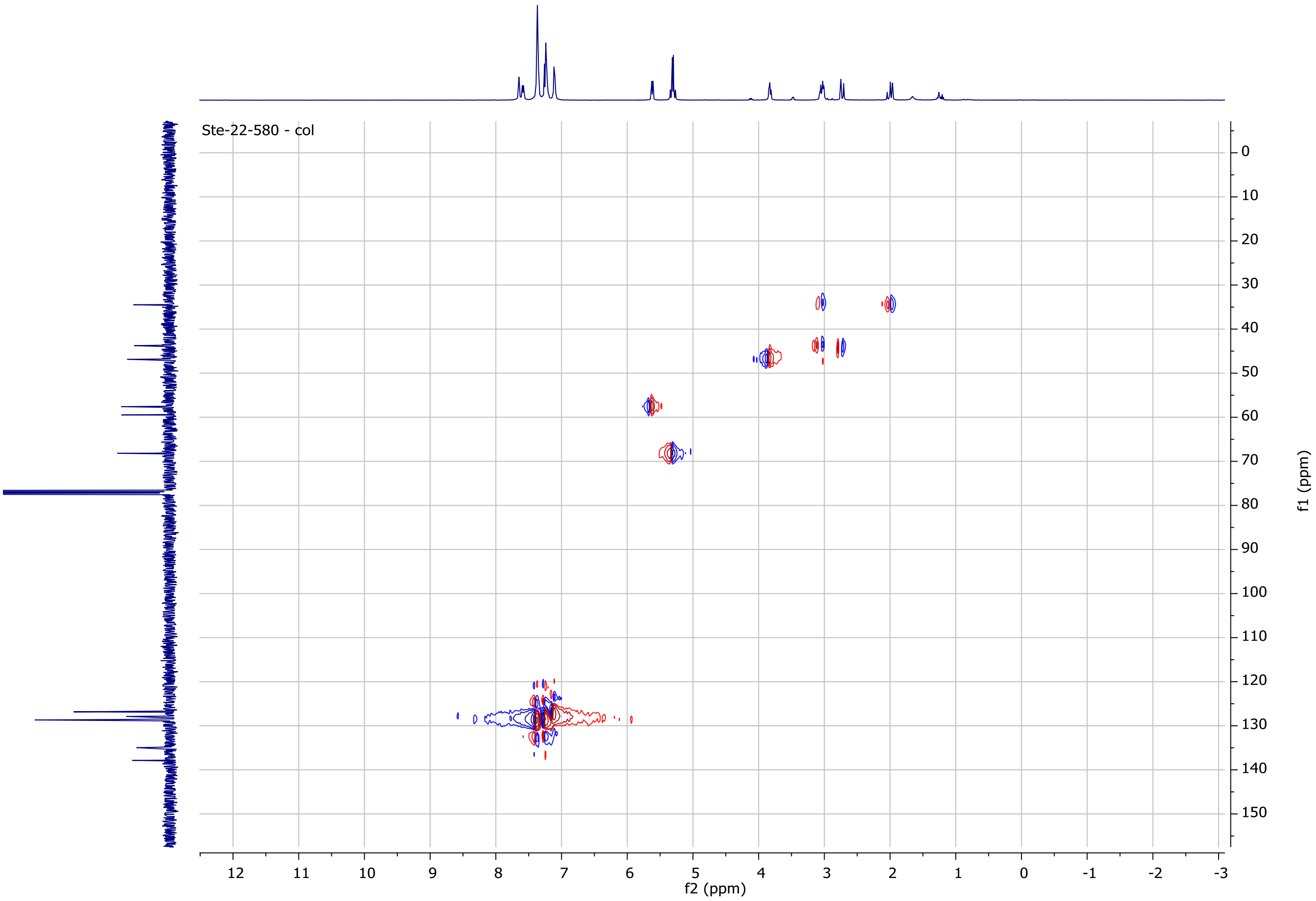
Ste-22-580 - col
f2 (ppm)
f1 (ppm)
12
11
10
9
8
7
6
5
4
3
2
1
0
-1
-2
-3
0
10
20
30
40
50
60
70
80
90
100
110
120
130
140
150
